# Supplementary material for: Classifying ball trajectories in invasion sports using dynamic time warping: A basketball case study
Source: PLoS One. 2022 Oct 20;17(10):e0272848. doi: 10.1371/journal.pone.0272848 (PMC9584368; doi:10.1371/journal.pone.0272848)
Supplement: S1 Appendix — Dynamic time warping algorithm. Dynamic time warping barycentre averaging. Validation indices and results. Silhouette index plot for K-means. Silhouette index plot for K-means (Australia Area 3). (DOCX) [file pone.0272848.s001.docx]

# **Appendices**

## **Appendix: Computer Software**

Morgan, S. Pattern Plotter (Version 4.5x) [36]. Australian Institute of Sport.

## Appendix: Rstudio Libraries

- - dplyr -> main use to modify and extract data
  - dtwclust -> to perform hierarchical clustering and other dtw related operations
  - ape -> dendro
  - ggplot2 -> trajectory + court plotting
  - ggdendro -> dendro
  - dendextend -> dendro
  - sparcl -> dendro
  - ggforce -> trajectory + court plotting
  - gtable -> trajectory + court plotting

## **Appendix: Dynamic Time Warping Algorithm**

$$\boldsymbol{\# DTW Algorithim}$$

$$DTW(s_{1}, s_{2})($$

$$T_{1} \leftarrow length(s_{1})$$

$$T_{2} \leftarrow length(s_{2})$$

$$matrix M\left[ 1:T_{1},1:T_{2} \right] \leftarrow0$$

$$M[1:T_{1},1] \leftarrow Inf$$

$$M[1,1:T_{2}]\leftarrow Inf$$

$$\boldsymbol{for} i from 2 to T_{1} \mathbf{do}$$

$$\boldsymbol{for} j from 2 to T_{2} \boldsymbol{do}$$

$$M[i][j]\leftarrow d_{e}(s_{1}[i],s_{2}[j])$$

$+ minimum(M[i-1][j-1],M\left[ i \right]\left[ j-1 \right], M[i-1][j])$

$$\boldsymbol{end for}$$

$$\boldsymbol{end for}$$

$$\boldsymbol{Return} (M[T_{1} + 1][T_{2} + 1])$$

$$)$$

## **Appendix: Dynamic Time Warping Barycentre Averaging**

Let $\boldsymbol{S}$ be a set of trajectories within a cluster. Let $\boldsymbol{G=}\boldsymbol{\{}{\boldsymbol{s}_{\boldsymbol{c}}}_{\boldsymbol{1}}\boldsymbol{, \ldots}{\boldsymbol{s}_{\boldsymbol{c}}}_{\boldsymbol{T}_{\boldsymbol{c}}}\boldsymbol{\}}$ be a dummy Average Trajectory, with $\boldsymbol{G'}$ as the updated mean trajectory and $\boldsymbol{aM}$ be an associate Matrix with elements as a set of coordinates associated to $\boldsymbol{G}$. $\boldsymbol{aM}$ elements are established during the DTW calculations, when optimal pathing and aligned coordinate pairs are defined.

$$\boldsymbol{DBA Algorithim}$$

$$DBA(S)($$

$$matrix aM\left[ 1:T,1:T' \right] \leftarrow0$$

$$\boldsymbol{for} T in S \boldsymbol{do}$$

$$M\leftarrow DTW(G,S)$$

$$i\leftarrow T^{'}$$

$$j\leftarrow T$$

$$\boldsymbol{while} i\geq1 and j \geq1 \boldsymbol{do}$$

$aM\left[ i \right]\leftarrow aM\left[ i \right]\cup S_{j}$

$\left( i,j \right)= second\left( m\left[ i,j \right] \right)$

$$\boldsymbol{end while}$$

$$\boldsymbol{end for}$$

$\boldsymbol{for}i=1 to T \boldsymbol{do}$

$G^{'}[i]=barycenter\left( aM\left[ i \right] \right)$ {See Equation 1}

$$\boldsymbol{end for}$$

$$\boldsymbol{Return}G’$$

$$)$$

## **Appendix: Validation indices and Results**

The CVI function (Clustering Validation Indices) in *‘dtwclust’* [35] can be used to assess cluster validity. In the following, $\boldsymbol{c}_{\boldsymbol{l}}$ is a unique second cluster:

- Dunn Index, measuring distance between clusters while ensuring sufficient distant between clusters (DI) [32]. This index identifies compact clusters with small variance of distance between data points while also ensuring clusters are sufficiently apart to be distinct. The index is to be maximised which denotes a cluster set to be well separated with small variance of datums. The mathematical definition is as follows:

$$D\left( C \right)=\frac{mi{n_{c}}_{k}\in c\{mi{n_{c}}_{1}\in C\backslash c_{k}\{\delta(c_{k},c_{l})\}\}}{ma{x_{c}}_{k}\in c\{\Delta(c_{k})\}}$$

Equation 4: Dunn Index Mathematical Equation

- Cop Index, measuring the distance of clusters relative to cluster centroid, and separation based on furthest neighbour distance (COP) [26]. This index is a ratio-type index where cohesion is calculated through estimating distance of cluster points to its centroid and separation is based on furthest neighbour distance. This index is to be minimised to indicate a good cluster. The mathematical definition is as follows:

$$COP\left( C \right)=\frac{1}{N}\sum_{c_{k}\in C} \left| c_{k} \right|\frac{1/\left| C_{k} \right|\sum_{{c_{k}}_{i}\in c_{k}} d_{e}\left( {c_{k}}_{i},\bar{c_{k}} \right)}{min_{{c_{k}}_{i}\notin c_{k}}d_{e}({c_{k}}_{i},{c_{k}}_{j})}$$

Equation 5: Cop Index Mathematical Equation

- Davies-Bouldin Index, measuring the ratio between within-cluster distance to between-cluster distances to indicate Davies-Bouldin Index (DB) [33]. This index is based on the ratio between within-cluster distance to between-cluster distances to indicate if the clusters are good fit. This index should be minimised. The Davies-Bouldin index is defined as:

$$DB\left( C \right)=\frac{1}{K}\sum_{c_{k} \in C} \max_{c_{1}\in C\backslash c_{k}} \{\frac{S\left( c_{k} \right)+S(c_{l})}{d_{e}(\bar{c_{k}},\bar{c_{l}})}\}$$

Equation 6: Davies-Bouldin Index Mathematical Equation

Where

$$S\left( C \right)=1/\left| c_{k} \right|\sum_{x_{i}\in c_{k}} d_{e}({c_{k}}_{i},\bar{c_{k}})$$

Equation 7: Sub-Equation for Davies-Bouldin Index

- Calinski-Harabasz Index, measures ratio of variance, with large between-cluster variance (SSB) and a small within-cluster variance (CH) [34]. The Calinski-harabasz index is a variance-type ratio index. Well-defined clusters are posed to have a large between-cluster variance (SSB) and a small within-cluster variance (SSW). The index is to be maximised to indicate a better partition. The mathematical definition is as follows:

Let $m$ be overall mean of sample data

$$CH\left( C \right)=\frac{SSB}{SSW}\times\frac{\left( U-K \right)}{\left( K-1 \right)}$$

Equation 8: Calinski-Harabasz Index Mathematical Equation

Where

$$SSB=\sum_{i=1}^{k} c_{k}\times d_{e}(\bar{c_{i}}-m )$$

$$SSW= \sum_{i=1}^{k} \sum_{c_{k}\in c_{i}} d_{e}(c_{k}-\bar{c_{i}})$$

Equation 9(a,b): Sub Equation for Calinski-Harabasz Index

Fig 9: Additional Validation Indices with Global Data

## **Appendix: Silhouette Index Plot for K-means**

The below SI Plot was used to determine the optimal number of clusters when partitioning the court.


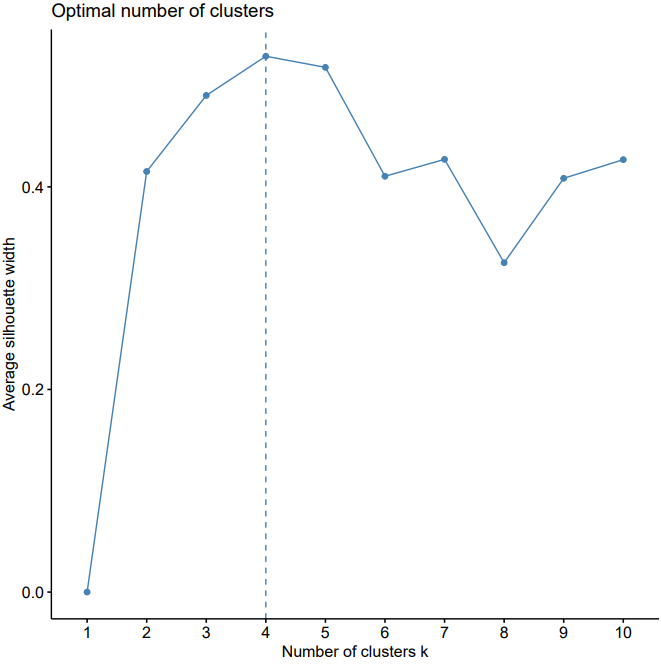


Fig 10: SI Plot to determine optimal clusters for K-Means

**Appendix: Silhouette Index Plot for K-means**


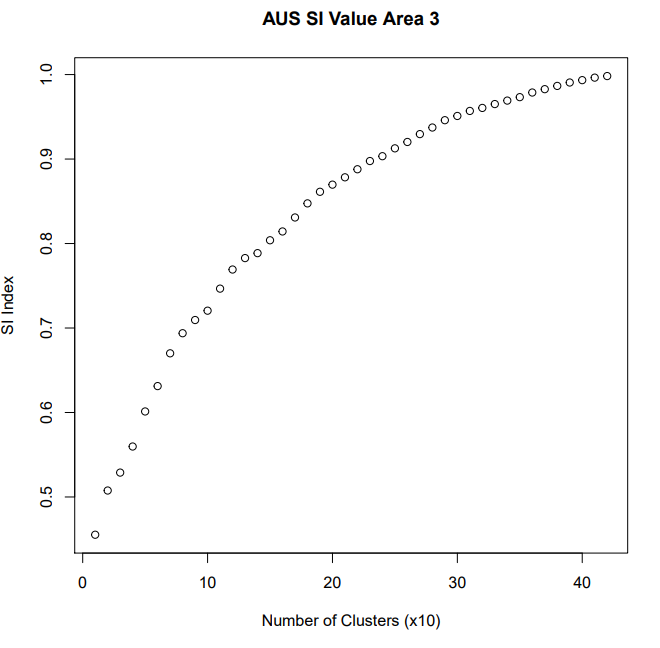


Fig 11: SI plot for Australia Dataset - Area 3

**References**

32. Dunn JC. A Fuzzy Relative of the ISODATA Process and Its Use in Detecting Compact Well-Separated Clusters. Journal of Cybernetics. ; 3: 32-57.

33. Davies DL, Bouldin D. A Cluster Separation Measure. IEEE Transactions on Pattern Analysis and Machine Intelligence. 1979;: 224-227.

34. Calinski T, Harabasz J. A dendrite method for Cluster Analysis. Communications in Statistics. 1974; 3(1): 1-27.

35. Sardá-Espinosa A. Comparing Time-Series Clustering Algorithms in R Using the dtwclust Package. ; 2018.

36. S. M. Pattern Plotter. 2018. Australian Institute of Sport.
