# Supplementary material for: Classifying ball trajectories in invasion sports using dynamic time warping: A basketball case study
Source: PLoS One. 2022 Oct 20;17(10):e0272848. doi: 10.1371/journal.pone.0272848 (PMC9584368; doi:10.1371/journal.pone.0272848)
Supplement: S2 Appendix — Table 3. Australia Summary Statistics. Table 4. Japan Summary Statistics. Table 5. USA Summary Statistics. (DOCX) [file pone.0272848.s002.docx]

## **Appendix: Australia Summary Statistics**

Table 3: Summary Statistics for Australia Data

| **Area** | **Cluster** | **Trajectories** | **Frequency of Execution (%)** | **Mean Trajectory Duration (sec)** | **SD Trajectory Duration (sec)** | **Mean Change in Heading (deg)** | **SD Change in Heading (deg)** | **Mean Heading (deg)** | **SD Heading (deg)** | **Scoring Rate (%)** | **Mean distance travelled (Foot)** | **SD distance travelled (Foot)** |
| --- | --- | --- | --- | --- | --- | --- | --- | --- | --- | --- | --- | --- |
| 1 | 1 | 16 | 1.7131 | 14.6875 | 3.1563 | 159.437 | 66.033 | -2.819 | 7.311 | 25 | 73.6106 | 11.3386 |
| 1 | 2 | 13 | 1.3919 | 7.6923 | 2.097 | 102.548 | 38.302 | 2 | 6.835 | 23.077 | 65.8444 | 12.7559 |
| 1 | 3 | 11 | 1.1777 | 8.3636 | 2.6181 | 106.57 | 71.431 | -14.513 | 8.354 | 18.182 | 54.26 | 9.0835 |
| 1 | 4 | 8 | 0.8565 | 17.125 | 2.5319 | 255.201 | 34.091 | -5.139 | 9.374 | 12.5 | 67.1471 | 13.3597 |
| 1 | 5 | 6 | 0.6424 | 5.5 | 4.8062 | 120.865 | 28.889 | -5.907 | 10.846 | 16.667 | 31.2364 | 12.3018 |
| 1 | 6 | 5 | 0.5353 | 12.4 | 7.3007 | 218.383 | 113.016 | -13.379 | 15.034 | 0 | 101.6524 | 11.5798 |
| 1 | 7 | 5 | 0.5353 | 3.4 | 0.5477 | 33.123 | 19.309 | -26.872 | 7.552 | 0 | 25.7431 | 5.4642 |
| 1 | 8 | 4 | 0.4283 | 13.5 | 2.6458 | 231.836 | 89.095 | 11.161 | 9.442 | 50 | 71.6423 | 6.1906 |
| 1 | 9 | 4 | 0.4283 | 14.5 | 3.3166 | 345.402 | 89.525 | 2.366 | 15.544 | 0 | 59.1317 | 24.1473 |
| 1 | 10 | 4 | 0.4283 | 6 | 2.1602 | 32.361 | 9.62 | -11.728 | 6.113 | 0 | 45.0015 | 9.5099 |
| 1 | 11 | 2 | 0.2141 | 18.5 | 3.5355 | 392.831 | 111.022 | -5.523 | 5.775 | 0 | 106.7572 | 21.6318 |
| 1 | 12 | 2 | 0.2141 | 15 | 1.4142 | 354.695 | 191.912 | 4.022 | 4.039 | 50 | 61.2794 | 8.2184 |
| 1 | 13 | 2 | 0.2141 | 18.5 | 0.7071 | 568.546 | 176.116 | -3.363 | 4.589 | 50 | 98.9059 | 11.5371 |
| 1 | 14 | 2 | 0.2141 | 8 | 2.8284 | 188.601 | 22.546 | -23.892 | 2.303 | 50 | 42.7977 | 8.1558 |
| 1 | 15 | 2 | 0.2141 | 3.5 | 2.1213 | 24.27 | 16.604 | -59.399 | 3.151 | 0 | 4.6638 | 3.5855 |
| 1 | 16 | 2 | 0.2141 | 23 | 1.4142 | 341.368 | 203.572 | 3.025 | 5.563 | 50 | 74.5373 | 22.2499 |
| 2 | 1 | 15 | 1.606 | 5.5333 | 1.7265 | 55.886 | 31.14 | 8.549 | 10.995 | 6.667 | 54.7851 | 9.395 |
| 2 | 2 | 10 | 1.0707 | 14.8 | 3.2592 | 272.504 | 82.867 | 2.607 | 10.921 | 0 | 88.1615 | 17.8932 |
| 2 | 3 | 9 | 0.9636 | 14.4444 | 3.0867 | 139.297 | 48.896 | -2.63 | 6.801 | 44.444 | 66.922 | 12.0043 |
| 2 | 4 | 9 | 0.9636 | 9.5556 | 2.0069 | 113.978 | 56.837 | 8.623 | 9.54 | 22.222 | 65.4537 | 5.2761 |
| 2 | 5 | 8 | 0.8565 | 16.875 | 2.7484 | 175.846 | 28.614 | 13.023 | 4.635 | 0 | 72.3997 | 8.388 |
| 2 | 6 | 7 | 0.7495 | 17.1429 | 3.0237 | 281.569 | 63.656 | -20.088 | 9.282 | 42.857 | 92.0492 | 20.2559 |
| 2 | 7 | 7 | 0.7495 | 11.2857 | 2.2147 | 190.107 | 22.42 | -21.222 | 11.127 | 42.857 | 62.9512 | 17.1626 |
| 2 | 8 | 5 | 0.5353 | 4.6 | 1.8166 | 119.118 | 167.046 | 6.566 | 10.239 | 20 | 23.6996 | 7.0664 |
| 2 | 9 | 5 | 0.5353 | 19.8 | 1.7889 | 225.568 | 76.455 | -3.879 | 8.027 | 0 | 89.8974 | 22.7043 |
| 2 | 10 | 4 | 0.4283 | 21.25 | 1.2583 | 391.542 | 81.813 | 2.974 | 9.775 | 25 | 106.9709 | 15.066 |
| 2 | 11 | 4 | 0.4283 | 6.75 | 3.304 | 145.044 | 46.192 | -18.547 | 26.012 | 50 | 23.4301 | 16.7585 |
| 2 | 12 | 4 | 0.4283 | 21 | 1.8257 | 425.307 | 40.829 | 13.092 | 13.677 | 25 | 84.248 | 11.4648 |
| 2 | 13 | 4 | 0.4283 | 9.5 | 2.0817 | 128.789 | 26.895 | -16.495 | 10.342 | 0 | 25.6583 | 2.2146 |
| 2 | 14 | 3 | 0.3212 | 20 | 1 | 185.988 | 4.561 | 23.325 | 16.759 | 0 | 80.3539 | 7.7715 |
| 2 | 15 | 1 | 0.1071 | 23 | NA | 349.126 | NA | 8.594 | NA | 0 | 122.184 | NA |
| 3 | 1 | 34 | 3.6403 | 5.5882 | 1.7773 | 96.331 | 60.413 | -3.667 | 18.621 | 44.118 | 60.8751 | 9.2206 |
| 3 | 2 | 31 | 3.3191 | 8 | 2.1602 | 119.353 | 61.198 | -11.333 | 13.591 | 29.032 | 74.5008 | 7.97 |
| 3 | 3 | 30 | 3.212 | 3.8333 | 1.3153 | 74.817 | 47.67 | 13.401 | 23.64 | 60 | 32.6436 | 10.9074 |
| 3 | 4 | 22 | 2.3555 | 12.0455 | 1.3965 | 128.944 | 48.386 | 8.938 | 8.417 | 27.273 | 74.4336 | 10.839 |
| 3 | 5 | 21 | 2.2484 | 12.0952 | 1.9724 | 176.448 | 59.307 | 2.613 | 9.918 | 57.143 | 74.0438 | 7.5899 |
| 3 | 6 | 20 | 2.1413 | 8.4 | 1.875 | 138.965 | 86.603 | 20.139 | 9.987 | 35 | 71.1293 | 7.1378 |
| 3 | 7 | 20 | 2.1413 | 14.75 | 2.4682 | 153.358 | 55.033 | 14.158 | 5.999 | 35 | 77.765 | 6.5939 |
| 3 | 8 | 20 | 2.1413 | 11.8 | 2.2618 | 237.296 | 174.976 | -12.347 | 9.958 | 30 | 72.6537 | 9.6632 |
| 3 | 9 | 17 | 1.8201 | 6.4706 | 1.2307 | 121.811 | 106.622 | 19.211 | 14.461 | 23.529 | 71.1083 | 6.2399 |
| 3 | 10 | 14 | 1.4989 | 15.3571 | 2.17 | 280.514 | 98.532 | 8.239 | 11.826 | 28.571 | 88.4606 | 11.3146 |
| 3 | 11 | 14 | 1.4989 | 10.3571 | 2.4371 | 202.203 | 97.242 | -2.699 | 14.536 | 42.857 | 41.3235 | 13.3192 |
| 3 | 12 | 13 | 1.3919 | 11.7692 | 2.4547 | 152.985 | 53.503 | 19.899 | 8.497 | 30.769 | 71.7065 | 15.6306 |
| 3 | 13 | 12 | 1.2848 | 16.4167 | 2.3533 | 322.506 | 174.678 | 13.253 | 9.672 | 33.333 | 82.3777 | 11.2804 |
| 3 | 14 | 11 | 1.1777 | 19.5455 | 3.1101 | 243.673 | 70.806 | 5.97 | 9.757 | 45.455 | 92.1455 | 12.9826 |
| 3 | 15 | 11 | 1.1777 | 8.4545 | 1.8635 | 112.586 | 32.974 | 6.303 | 21.497 | 36.364 | 46.5183 | 14.85 |
| 3 | 16 | 11 | 1.1777 | 15.1818 | 2.3587 | 304.739 | 124.148 | 11.837 | 9.557 | 72.727 | 91.1302 | 11.0612 |
| 3 | 17 | 10 | 1.0707 | 10.8 | 1.3984 | 197.722 | 57.187 | -19.727 | 6.52 | 30 | 78.8633 | 10.157 |
| 3 | 18 | 10 | 1.0707 | 18.1 | 2.4698 | 212.716 | 58.763 | 6.434 | 8.663 | 30 | 83.087 | 7.6823 |
| 3 | 19 | 10 | 1.0707 | 8.2 | 1.3984 | 161.729 | 68.766 | 1.57 | 34.183 | 50 | 28.939 | 6.3694 |
| 3 | 20 | 9 | 0.9636 | 3.1111 | 0.928 | 66.056 | 50.426 | 8.629 | 38.858 | 33.333 | 8.3526 | 6.401 |
| 3 | 21 | 9 | 0.9636 | 5.3333 | 1.9365 | 92.292 | 80.151 | -9.448 | 20.162 | 44.444 | 64.2681 | 11.8536 |
| 3 | 22 | 9 | 0.9636 | 14.3333 | 1.7321 | 192.21 | 74.089 | -3.146 | 9.941 | 33.333 | 77.7904 | 9.2834 |
| 3 | 23 | 8 | 0.8565 | 22.75 | 2.3755 | 310.589 | 111.669 | -4.314 | 8.365 | 37.5 | 95.5013 | 22.0408 |
| 3 | 24 | 8 | 0.8565 | 13.625 | 2.3261 | 218.211 | 91.341 | 1.266 | 15.533 | 0 | 60.368 | 16.4986 |
| 3 | 25 | 7 | 0.7495 | 19.2857 | 1.8898 | 197.315 | 86.058 | -4.927 | 5.46 | 14.286 | 89.2002 | 14.9324 |
| 3 | 26 | 7 | 0.7495 | 11.4286 | 1.5119 | 216.601 | 42.032 | -1.003 | 16.175 | 14.286 | 63.8742 | 16.2557 |
| 3 | 27 | 7 | 0.7495 | 10.5714 | 1.2724 | 100.583 | 38.898 | -8.199 | 8.961 | 42.857 | 68.3859 | 4.3706 |
| 3 | 28 | 6 | 0.6424 | 16.1667 | 2.4014 | 208.866 | 65.85 | -17.298 | 4.475 | 50 | 83.2987 | 20.7137 |
| 3 | 29 | 6 | 0.6424 | 13.3333 | 3.3267 | 215.209 | 150.212 | 27.903 | 10.451 | 16.667 | 80.7673 | 11.9831 |
| 3 | 30 | 5 | 0.5353 | 15.4 | 2.3022 | 344.938 | 93.936 | 0.917 | 16.524 | 0 | 89.5861 | 10.5482 |
| 3 | 31 | 5 | 0.5353 | 20.6 | 2.1909 | 351.057 | 59.381 | 9.603 | 11.557 | 40 | 111.2063 | 8.8634 |
| 3 | 32 | 5 | 0.5353 | 21.2 | 1.9235 | 165.831 | 52.179 | 16.914 | 11.694 | 40 | 75.4401 | 23.49 |
| 3 | 33 | 4 | 0.4283 | 16.25 | 1.7078 | 370.973 | 167.103 | 4.303 | 13.098 | 50 | 61.2936 | 8.4557 |
| 3 | 34 | 3 | 0.3212 | 20.6667 | 0.5774 | 388.334 | 113.268 | -3.782 | 19.314 | 0 | 98.8063 | 8.7553 |
| 3 | 35 | 2 | 0.2141 | 20.5 | 2.1213 | 366.086 | 132.485 | 7.5 | 2.911 | 0 | 80.8373 | 8.0229 |
| 3 | 36 | 2 | 0.2141 | 13.5 | 0.7071 | 470.433 | 204.036 | 21.171 | 10.789 | 50 | 90.6065 | 2.0039 |
| 3 | 37 | 2 | 0.2141 | 16.5 | 0.7071 | 508.609 | 67.105 | 2.366 | 11.322 | 50 | 128.1393 | 3.8052 |
| 3 | 38 | 1 | 0.1071 | 22 | NA | 726.298 | NA | -5.122 | NA | 0 | 204.9157 | NA |
| 4 | 1 | 14 | 1.4989 | 4.7857 | 1.8051 | 56.694 | 35.432 | 15.47 | 10.657 | 7.143 | 45.1501 | 10.2412 |
| 4 | 2 | 12 | 1.2848 | 10.9167 | 2.9987 | 165.459 | 85.846 | 1.455 | 12.702 | 16.667 | 67.1269 | 17.0026 |
| 4 | 3 | 9 | 0.9636 | 13 | 3.8406 | 119.542 | 59.055 | 2.361 | 7.649 | 11.111 | 68.2469 | 17.2648 |
| 4 | 4 | 9 | 0.9636 | 6.6667 | 3.6056 | 112.752 | 69.935 | 15.12 | 16.071 | 22.222 | 20.4638 | 17.8547 |
| 4 | 5 | 9 | 0.9636 | 6.7778 | 3.2702 | 35.718 | 16.249 | 14.095 | 5.042 | 0 | 43.8418 | 12.6757 |
| 4 | 6 | 8 | 0.8565 | 14.375 | 3.9619 | 220.715 | 63.369 | 14.754 | 10.164 | 25 | 72.1884 | 8.2675 |
| 4 | 7 | 7 | 0.7495 | 18.2857 | 1.7043 | 293.28 | 53.996 | 10.124 | 11.843 | 0 | 71.7605 | 20.0912 |
| 4 | 8 | 4 | 0.4283 | 10.5 | 2.3805 | 143.354 | 54.167 | -2.951 | 8.887 | 50 | 47.7048 | 9.897 |
| 4 | 9 | 3 | 0.3212 | 8.6667 | 3.0551 | 329.273 | 328.03 | 22.024 | 32.842 | 33.333 | 38.1562 | 7.094 |
| 4 | 10 | 3 | 0.3212 | 19.3333 | 4.1633 | 371.958 | 75.688 | -12.382 | 4.572 | 0 | 91.5157 | 26.1104 |
| 4 | 11 | 1 | 0.1071 | 24 | NA | 186.996 | NA | 0.51 | NA | 0 | 96.1183 | NA |
| 5 | 1 | 13 | 1.3919 | 6.9231 | 2.6287 | 143.285 | 105.504 | -15.67 | 12.296 | 38.462 | 59.0309 | 13.5513 |
| 5 | 2 | 9 | 0.9636 | 16.2222 | 3.1136 | 289.859 | 90.006 | -1.415 | 16.175 | 0 | 85.8931 | 13.6876 |
| 5 | 3 | 8 | 0.8565 | 12.125 | 2.5319 | 307.816 | 134.284 | -7.872 | 9.906 | 50 | 77.6212 | 28.7925 |
| 5 | 4 | 7 | 0.7495 | 13.2857 | 2.9277 | 196.576 | 41.081 | -17.544 | 17.361 | 0 | 43.8259 | 14.3365 |
| 5 | 5 | 7 | 0.7495 | 13.7143 | 3.2514 | 236.838 | 82.002 | 3.209 | 8.102 | 42.857 | 61.8186 | 16.2498 |
| 5 | 6 | 5 | 0.5353 | 6 | 2.3452 | 117.674 | 62.951 | -24.013 | 22.07 | 20 | 7.9599 | 4.4524 |
| 5 | 7 | 5 | 0.5353 | 8.6 | 1.9494 | 157.993 | 27.594 | 6.343 | 5.758 | 60 | 56.8272 | 2.2622 |
| 5 | 8 | 5 | 0.5353 | 19.8 | 3.3466 | 442.249 | 144.305 | 11.155 | 13.55 | 40 | 99.6063 | 17.018 |
| 5 | 9 | 5 | 0.5353 | 16.8 | 1.3038 | 299.342 | 49.538 | -12.765 | 6.257 | 20 | 79.5155 | 11.4838 |
| 5 | 10 | 3 | 0.3212 | 5 | 2 | 262.185 | 200.873 | -21.331 | 40.159 | 33.333 | 23.7387 | 19.9333 |
| 5 | 11 | 2 | 0.2141 | 20 | 1.4142 | 484.969 | 196.891 | -14.484 | 12.754 | 0 | 88.4316 | 15.1475 |
| 6 | 1 | 18 | 1.9272 | 7.3333 | 2.401 | 111.383 | 52.305 | 13.041 | 13.344 | 16.667 | 61.6212 | 16.0543 |
| 6 | 2 | 8 | 0.8565 | 18 | 2.4495 | 366.063 | 149.49 | -6.509 | 11.07 | 25 | 97.0491 | 14.6447 |
| 6 | 3 | 8 | 0.8565 | 13.375 | 2.1998 | 200.73 | 49.435 | 15.063 | 5.621 | 37.5 | 61.0325 | 11.9579 |
| 6 | 4 | 8 | 0.8565 | 12.75 | 2.8661 | 136.427 | 32.286 | 13.109 | 5.403 | 12.5 | 64.8604 | 14.0106 |
| 6 | 5 | 6 | 0.6424 | 6.3333 | 1.7512 | 121.639 | 69.001 | 8.359 | 9.007 | 33.333 | 63.782 | 12.3368 |
| 6 | 6 | 5 | 0.5353 | 11.4 | 1.9494 | 175.394 | 35.661 | 20.598 | 13.338 | 20 | 44.5171 | 18.973 |
| 6 | 7 | 5 | 0.5353 | 16.2 | 1.7889 | 280.268 | 49.893 | 11.253 | 8.004 | 40 | 73.5144 | 12.4166 |
| 6 | 8 | 5 | 0.5353 | 8.8 | 3.8987 | 251.551 | 210.207 | 15.533 | 28.522 | 20 | 36.2935 | 21.5249 |
| 6 | 9 | 3 | 0.3212 | 9.6667 | 2.0817 | 212.006 | 71.201 | -24.505 | 10.674 | 33.333 | 40.6282 | 25.4535 |
| 6 | 10 | 2 | 0.2141 | 21.5 | 0.7071 | 397.776 | 9.15 | 25.313 | 15.567 | 50 | 101.5301 | 29.8586 |
| 6 | 11 | 2 | 0.2141 | 17 | 1.4142 | 217.197 | 26.345 | 23.594 | 8.732 | 100 | 83.536 | 10.2005 |
| 6 | 12 | 2 | 0.2141 | 21.5 | 4.9497 | 299.073 | 49.017 | 9.173 | 2.492 | 0 | 70.6721 | 15.0891 |
| 6 | 13 | 1 | 0.1071 | 28 | NA | 152.539 | NA | 24.712 | NA | 0 | 50.5672 | NA |
| 6 | 14 | 1 | 0.1071 | 1 | NA | 8.531 | NA | 85.737 | NA | 100 | 3.2237 | NA |
| 7 | 1 | 17 | 1.8201 | 8.9412 | 2.8167 | 70.898 | 28.94 | 6.566 | 8.623 | 11.765 | 52.7424 | 9.6431 |
| 7 | 2 | 13 | 1.3919 | 8.6154 | 3.2542 | 145.084 | 97.346 | 0.052 | 20.214 | 38.462 | 22.3195 | 11.7075 |
| 7 | 3 | 12 | 1.2848 | 14.5833 | 2.3916 | 176.964 | 49.223 | -0.418 | 7.374 | 16.667 | 69.6455 | 11.5819 |
| 7 | 4 | 9 | 0.9636 | 6.2222 | 2.8186 | 80.856 | 58.659 | -13.751 | 6.028 | 33.333 | 47.7693 | 15.1245 |
| 7 | 5 | 7 | 0.7495 | 3.5714 | 0.9759 | 12.25 | 7.695 | -3.461 | 7.282 | 0 | 35.8094 | 10.349 |
| 7 | 6 | 7 | 0.7495 | 11.1429 | 2.4785 | 184.733 | 59.215 | 16.02 | 11.757 | 42.857 | 76.6566 | 14.0794 |
| 7 | 7 | 6 | 0.6424 | 14.3333 | 2.4221 | 206.139 | 40.525 | -6.033 | 9.397 | 0 | 48.3648 | 15.8391 |
| 7 | 8 | 4 | 0.4283 | 18 | 2.3094 | 297.388 | 73.814 | -16.249 | 11.207 | 50 | 82.1658 | 9.9791 |
| 7 | 9 | 4 | 0.4283 | 15.5 | 1.291 | 434.657 | 175.898 | -15.08 | 16.604 | 50 | 96.6427 | 37.4315 |
| 7 | 10 | 3 | 0.3212 | 19 | 2.6458 | 178.883 | 98.532 | -1.209 | 10.434 | 33.333 | 93.9244 | 27.516 |
| 7 | 11 | 3 | 0.3212 | 8.6667 | 4.0415 | 128.228 | 53.686 | -10.308 | 32.848 | 0 | 9.18 | 4.5491 |
| 7 | 12 | 2 | 0.2141 | 12 | 0 | 314.199 | 126.778 | 11.453 | 12.628 | 50 | 59.7442 | 3.5538 |
| 7 | 13 | 2 | 0.2141 | 23.5 | 2.1213 | 314.668 | 33.856 | 5.42 | 8.772 | 50 | 108.4152 | 11.8972 |
| 7 | 14 | 2 | 0.2141 | 24 | 1.4142 | 570.471 | 95.037 | -1.381 | 17.928 | 0 | 128.9506 | 49.1144 |
| 7 | 15 | 2 | 0.2141 | 18 | 0 | 336.882 | 127.403 | -12.708 | 15.006 | 50 | 99.271 | 14.1401 |

## **Appendix: Japan Summary Statistics**

Table 4: Summary Statistics for Japan Data

| **Area** | **Cluster** | **Trajectories** | **Frequency of Execution (%)** | **Mean Trajectory Duration (sec)** | **SD Trajectory Duration (sec)** | **Mean Change in Heading (deg)** | **SD Change in Heading (deg)** | **Mean Heading (deg)** | **SD Heading (deg)** | **Scoring Rate (%)** | **Mean distance travelled (Foot)** | **SD distance travelled (Foot)** |
| --- | --- | --- | --- | --- | --- | --- | --- | --- | --- | --- | --- | --- |
| 1 | 1 | 20 | 2.0942 | 6.95 | 1.9595 | 83.761 | 58.058 | 19.102 | 9.631 | 25 | 58.0314 | 10.6435 |
| 1 | 2 | 12 | 1.2565 | 7.4167 | 3.2879 | 156.721 | 55.268 | -11.31 | 8.892 | 58.333 | 64.4829 | 18.6768 |
| 1 | 3 | 11 | 1.1518 | 11.0909 | 3.0807 | 122.756 | 79.819 | 9.454 | 6.474 | 0 | 65.5561 | 10.1121 |
| 1 | 4 | 8 | 0.8377 | 19 | 2.0702 | 169.842 | 58.373 | -1.816 | 6.715 | 37.5 | 71.3556 | 16.2293 |
| 1 | 5 | 6 | 0.6283 | 9.5 | 2.8107 | 113.835 | 60.04 | 7.013 | 10.359 | 0 | 26.1994 | 14.8953 |
| 1 | 6 | 6 | 0.6283 | 10 | 2.8284 | 240.717 | 37.655 | -16.026 | 10.726 | 33.333 | 38.862 | 12.2669 |
| 1 | 7 | 5 | 0.5236 | 16.8 | 1.9235 | 283.964 | 94.252 | 7.729 | 6.973 | 60 | 93.0343 | 33.9927 |
| 1 | 8 | 4 | 0.4188 | 3.5 | 0.5774 | 25.428 | 19.223 | 50.088 | 12.198 | 0 | 8.0521 | 8.254 |
| 1 | 9 | 4 | 0.4188 | 4.5 | 1.7321 | 23.296 | 24.643 | 11.167 | 7.151 | 0 | 27.2147 | 14.0619 |
| 1 | 10 | 3 | 0.3141 | 20 | 3 | 268.047 | 83.039 | 5.586 | 9.609 | 0 | 92.2505 | 6.0719 |
| 1 | 11 | 2 | 0.2094 | 9.5 | 4.9497 | 91.347 | 20.626 | 9.351 | 28.533 | 50 | 36.813 | 2.9299 |
| 1 | 12 | 1 | 0.1047 | 26 | NA | 564.329 | NA | 19.412 | NA | 100 | 105.321 | NA |
| 2 | 1 | 13 | 1.3613 | 17.6154 | 3.2542 | 290.598 | 102.829 | -1.673 | 6.062 | 38.462 | 76.8935 | 16.5277 |
| 2 | 2 | 12 | 1.2565 | 7.6667 | 2.3868 | 184.79 | 61.473 | -5.008 | 14.857 | 50 | 76.3553 | 15.0664 |
| 2 | 3 | 11 | 1.1518 | 11.9091 | 1.9725 | 150.401 | 58.694 | 10.617 | 8.738 | 18.182 | 69.216 | 15.3692 |
| 2 | 4 | 10 | 1.0471 | 6.4 | 1.3499 | 66.245 | 13.654 | 21.016 | 6.457 | 10 | 59.6169 | 10.457 |
| 2 | 5 | 7 | 0.733 | 6.8571 | 2.7343 | 137.596 | 67.626 | 31.925 | 14.387 | 28.571 | 51.7922 | 10.948 |
| 2 | 6 | 7 | 0.733 | 7 | 2.8868 | 155.896 | 86.545 | 22.701 | 17.504 | 14.286 | 19.8191 | 15.1206 |
| 2 | 7 | 7 | 0.733 | 15.8571 | 1.7728 | 267.623 | 31.742 | 13.304 | 10.113 | 14.286 | 67.3562 | 16.1422 |
| 2 | 8 | 6 | 0.6283 | 14.3333 | 1.9664 | 303.238 | 73.659 | 10.101 | 13.591 | 0 | 92.355 | 14.7146 |
| 2 | 9 | 6 | 0.6283 | 17.8333 | 2.7869 | 203.4 | 84.649 | 17.469 | 4.71 | 16.667 | 72.0673 | 8.0092 |
| 2 | 10 | 4 | 0.4188 | 10.75 | 2.2174 | 188.142 | 73.957 | 13.888 | 16.822 | 0 | 53.1588 | 19.3855 |
| 2 | 11 | 4 | 0.4188 | 12.5 | 1.291 | 204.357 | 8.589 | -3.741 | 9.408 | 0 | 64.6124 | 11.4571 |
| 2 | 12 | 3 | 0.3141 | 3 | 1 | 42.617 | 63.661 | 44.307 | 34.005 | 66.667 | 2.3852 | 2.997 |
| 3 | 1 | 22 | 2.3037 | 12 | 1.48 | 162.078 | 49.481 | 12.748 | 7.03 | 31.818 | 74.1285 | 8.1566 |
| 3 | 2 | 17 | 1.7801 | 6.2353 | 1.855 | 104.175 | 39.414 | -2.401 | 9.591 | 41.176 | 60.4755 | 5.6904 |
| 3 | 3 | 16 | 1.6754 | 9.125 | 1.7842 | 112.752 | 44.003 | 6.045 | 8.01 | 18.75 | 75.063 | 11.2184 |
| 3 | 4 | 13 | 1.3613 | 3.7692 | 0.9268 | 64.521 | 33.61 | 18.908 | 15.401 | 69.231 | 35.1587 | 12.6065 |
| 3 | 5 | 12 | 1.2565 | 9.4167 | 2.8431 | 219.477 | 142.053 | 8.955 | 22.03 | 41.667 | 30.1187 | 8.8344 |
| 3 | 6 | 10 | 1.0471 | 14.2 | 3.6148 | 166.141 | 79.068 | 14.662 | 6.973 | 60 | 63.1539 | 17.06 |
| 3 | 7 | 10 | 1.0471 | 22.1 | 1.912 | 299.674 | 54.695 | 2.269 | 7.764 | 10 | 87.9383 | 10.4291 |
| 3 | 8 | 10 | 1.0471 | 9.1 | 1.9692 | 152.808 | 174.987 | 22.66 | 13.911 | 20 | 61.9988 | 7.2226 |
| 3 | 9 | 10 | 1.0471 | 14.9 | 2.5144 | 245.701 | 120.401 | 2.045 | 12.662 | 20 | 81.8856 | 13.1176 |
| 3 | 10 | 9 | 0.9424 | 4 | 2.2361 | 101.654 | 183.753 | 35.959 | 32.693 | 55.556 | 17.1655 | 11.6233 |
| 3 | 11 | 8 | 0.8377 | 20.125 | 1.4577 | 287.636 | 59.358 | 13.178 | 6.669 | 25 | 93.5877 | 14.4063 |
| 3 | 12 | 8 | 0.8377 | 16.625 | 1.7678 | 270.247 | 66.383 | 6.28 | 10.846 | 50 | 79.4314 | 7.945 |
| 3 | 13 | 8 | 0.8377 | 5.25 | 2.1213 | 64.595 | 23.411 | 30.287 | 9.202 | 0 | 69.1427 | 18.1704 |
| 3 | 14 | 8 | 0.8377 | 15.375 | 1.598 | 219.059 | 56.688 | 12.29 | 15.487 | 12.5 | 69.2121 | 8.4614 |
| 3 | 15 | 8 | 0.8377 | 11.625 | 2.3261 | 192.273 | 78.965 | -4.057 | 12.794 | 62.5 | 49.2876 | 15.5277 |
| 3 | 16 | 7 | 0.733 | 15.1429 | 2.6726 | 281.185 | 57.794 | -3.936 | 7.214 | 42.857 | 83.8661 | 13.8516 |
| 3 | 17 | 7 | 0.733 | 11.5714 | 2.3705 | 219.03 | 38.938 | 11.608 | 12.88 | 57.143 | 36.7444 | 13.6497 |
| 3 | 18 | 7 | 0.733 | 19.8571 | 2.9114 | 371.397 | 161.992 | 21.709 | 6.732 | 42.857 | 100.4178 | 21.1259 |
| 3 | 19 | 6 | 0.6283 | 5 | 2.0976 | 86.952 | 33.283 | 10.273 | 12.582 | 50 | 65.9638 | 6.1094 |
| 3 | 20 | 4 | 0.4188 | 11.5 | 4.3589 | 196.777 | 91.112 | -26.562 | 11.276 | 50 | 86.1451 | 24.7014 |
| 3 | 21 | 4 | 0.4188 | 13.5 | 2.0817 | 282.783 | 56.27 | -4.922 | 17.441 | 25 | 61.568 | 17.0848 |
| 3 | 22 | 2 | 0.2094 | 16.5 | 4.9497 | 239.938 | 11.167 | -10.204 | 20.615 | 50 | 108.4476 | 30.3612 |
| 3 | 23 | 2 | 0.2094 | 23 | 0 | 513.622 | 127.363 | 13.545 | 16.908 | 100 | 130.7046 | 10.6183 |
| 3 | 24 | 2 | 0.2094 | 23.5 | 0.7071 | 512.156 | 97.197 | 1.329 | 8.841 | 50 | 124.4757 | 6.5855 |
| 3 | 25 | 2 | 0.2094 | 22 | 1.4142 | 119.026 | 25.245 | 17.624 | 12.536 | 50 | 76.4435 | 4.9785 |
| 3 | 26 | 1 | 0.1047 | 19 | NA | 359.64 | NA | 7.832 | NA | 0 | 120.2375 | NA |
| 4 | 1 | 20 | 2.0942 | 7.45 | 2.6651 | 92.642 | 53.193 | 1.261 | 9.551 | 25 | 58.981 | 13.8371 |
| 4 | 2 | 11 | 1.1518 | 13 | 3.5777 | 149.513 | 94.847 | -6.663 | 7.672 | 27.273 | 65.8902 | 11.3733 |
| 4 | 3 | 10 | 1.0471 | 6.2 | 3.5214 | 91.473 | 80.965 | -17.865 | 16.369 | 30 | 23.8569 | 22.3373 |
| 4 | 4 | 8 | 0.8377 | 3.625 | 2.2638 | 24.425 | 15.871 | -15.573 | 4.102 | 0 | 33.1927 | 13.4875 |
| 4 | 5 | 6 | 0.6283 | 6.3333 | 3.2042 | 50.965 | 31.45 | -26.671 | 3.289 | 50 | 52.4274 | 18.3418 |
| 4 | 6 | 6 | 0.6283 | 16.3333 | 3.3862 | 293.469 | 53.067 | -13.843 | 12.187 | 0 | 97.1853 | 19.4075 |
| 4 | 7 | 5 | 0.5236 | 7.4 | 2.51 | 153.908 | 119.966 | 1.387 | 6.165 | 0 | 27.2995 | 26.0994 |
| 4 | 8 | 3 | 0.3141 | 13.3333 | 3.2146 | 210.969 | 51.709 | 23.761 | 10.25 | 66.667 | 90.1956 | 2.3085 |
| 4 | 9 | 3 | 0.3141 | 14.6667 | 3.2146 | 238.098 | 92.309 | 2.332 | 21.595 | 33.333 | 41.0326 | 24.4241 |
| 4 | 10 | 1 | 0.1047 | 13 | NA | 116.54 | NA | 21.354 | NA | 0 | 50.7851 | NA |
| 5 | 1 | 15 | 1.5707 | 6.8 | 2.1112 | 162.777 | 70.462 | -3.81 | 14.61 | 33.333 | 49.7201 | 19.4783 |
| 5 | 2 | 14 | 1.466 | 10.7857 | 2.2931 | 150.745 | 33.879 | 0.407 | 8.543 | 21.429 | 68.2804 | 12.6095 |
| 5 | 3 | 13 | 1.3613 | 4.9231 | 1.0377 | 72.565 | 24.018 | -13.803 | 17.109 | 30.769 | 50.0803 | 12.6078 |
| 5 | 4 | 9 | 0.9424 | 14.5556 | 1.8105 | 255.069 | 61.748 | 8.652 | 9.007 | 55.556 | 77.2978 | 14.7617 |
| 5 | 5 | 8 | 0.8377 | 13.625 | 2.8754 | 266.402 | 73.006 | -13.757 | 12.152 | 12.5 | 72.9674 | 15.7419 |
| 5 | 6 | 7 | 0.733 | 8.4286 | 2.3705 | 201.314 | 116.626 | -5.764 | 19.681 | 28.571 | 28.1431 | 16.8843 |
| 5 | 7 | 5 | 0.5236 | 16.6 | 1.5166 | 249.918 | 24.947 | -2.206 | 4.922 | 0 | 76.3022 | 8.3171 |
| 5 | 8 | 5 | 0.5236 | 16.4 | 1.9494 | 262.163 | 51.446 | -1.977 | 2.561 | 60 | 73.7579 | 23.3448 |
| 5 | 9 | 5 | 0.5236 | 22.6 | 2.3022 | 445.566 | 100.038 | 1.14 | 8.675 | 40 | 112.1036 | 19.9248 |
| 5 | 10 | 4 | 0.4188 | 10.25 | 2.63 | 327.95 | 129.311 | -14.633 | 23.056 | 25 | 65.1087 | 20.4409 |
| 5 | 11 | 4 | 0.4188 | 3.75 | 2.8723 | 89.038 | 64.08 | -34.102 | 22.54 | 0 | 6.8385 | 3.6578 |
| 5 | 12 | 4 | 0.4188 | 2.75 | 1.893 | 75.562 | 67.959 | -45.367 | 46.329 | 25 | 10.533 | 9.9382 |
| 5 | 13 | 3 | 0.3141 | 21.3333 | 4.5092 | 590.009 | 191.001 | 12.336 | 12.588 | 0 | 85.3258 | 23.8983 |
| 5 | 14 | 3 | 0.3141 | 17 | 3 | 362.396 | 203.538 | -13.992 | 5.168 | 0 | 70.4381 | 10.6669 |
| 5 | 15 | 3 | 0.3141 | 16.3333 | 1.1547 | 376.565 | 199.343 | -4.687 | 5.512 | 33.333 | 93.7717 | 3.9162 |
| 5 | 16 | 3 | 0.3141 | 22 | 2.6458 | 280.325 | 111.893 | -15.974 | 2.246 | 0 | 95.8693 | 38.523 |
| 5 | 17 | 2 | 0.2094 | 11.5 | 0.7071 | 243.937 | 52.088 | -9.941 | 19.349 | 50 | 87.2564 | 10.0026 |
| 5 | 18 | 1 | 0.1047 | 24 | NA | 425.438 | NA | -18.827 | NA | 0 | 124.637 | NA |
| 6 | 1 | 26 | 2.7225 | 7.1923 | 2.2631 | 89.697 | 47.997 | -25.159 | 11.196 | 57.692 | 62.1935 | 11.143 |
| 6 | 2 | 22 | 2.3037 | 7.4545 | 1.2622 | 109.114 | 51.085 | -11.82 | 10.141 | 40.909 | 68.0961 | 7.6078 |
| 6 | 3 | 22 | 2.3037 | 13.1364 | 2.7307 | 203.778 | 69.649 | 5.414 | 10.646 | 22.727 | 69.1447 | 13.6342 |
| 6 | 4 | 16 | 1.6754 | 6.875 | 2.6045 | 128.153 | 60.619 | 11.459 | 12.576 | 12.5 | 68.9786 | 11.4373 |
| 6 | 5 | 16 | 1.6754 | 15.9375 | 2.8628 | 216.051 | 67.391 | -17.183 | 7.408 | 25 | 81.2441 | 8.5415 |
| 6 | 6 | 13 | 1.3613 | 15.6923 | 1.7974 | 252.187 | 70.388 | 2.378 | 9.425 | 30.769 | 83.5987 | 13.6551 |
| 6 | 7 | 12 | 1.2565 | 5.0833 | 2.3533 | 101.694 | 53.629 | -9.482 | 26.018 | 41.667 | 28.3248 | 12.2788 |
| 6 | 8 | 11 | 1.1518 | 13.9091 | 3.113 | 194.639 | 55.422 | -10.737 | 7.477 | 45.455 | 65.4026 | 6.2544 |
| 6 | 9 | 11 | 1.1518 | 12.4545 | 2.4643 | 210.327 | 61.381 | -3.85 | 9.288 | 9.091 | 78.6293 | 9.0634 |
| 6 | 10 | 10 | 1.0471 | 13.6 | 2.3664 | 132.405 | 51.142 | -8.4 | 11.367 | 20 | 82.0838 | 13.6875 |
| 6 | 11 | 9 | 0.9424 | 3.7778 | 0.9718 | 43.361 | 22.569 | -9.139 | 11.178 | 44.444 | 60.1978 | 9.5652 |
| 6 | 12 | 9 | 0.9424 | 19 | 1.7321 | 234.133 | 140.489 | -4.899 | 13.39 | 55.556 | 83.0925 | 8.3709 |
| 6 | 13 | 9 | 0.9424 | 9 | 2 | 151.782 | 69.483 | 1.455 | 20.26 | 22.222 | 28.6877 | 8.3439 |
| 6 | 14 | 9 | 0.9424 | 18 | 1.8708 | 388.649 | 91.742 | -4.183 | 10.817 | 33.333 | 103.6526 | 22.4032 |
| 6 | 15 | 7 | 0.733 | 12.7143 | 1.6036 | 197.458 | 73.528 | 0.435 | 9.437 | 28.571 | 77.6559 | 7.8225 |
| 6 | 16 | 6 | 0.6283 | 16.6667 | 2.8048 | 308.996 | 69.677 | -11.178 | 14.335 | 16.667 | 93.5071 | 14.7382 |
| 6 | 17 | 6 | 0.6283 | 18.1667 | 2.7869 | 280.83 | 90.779 | 7.042 | 10.365 | 16.667 | 101.1694 | 25.0835 |
| 6 | 18 | 6 | 0.6283 | 9.5 | 1.7607 | 158.406 | 90.659 | -14.851 | 7.781 | 50 | 28.6844 | 10.0964 |
| 6 | 19 | 5 | 0.5236 | 14.6 | 1.8166 | 205.829 | 70.766 | 15.711 | 12.256 | 0 | 86.6092 | 26.346 |
| 6 | 20 | 5 | 0.5236 | 22.6 | 3.5071 | 223.459 | 94.509 | 2.057 | 13.591 | 0 | 83.7823 | 9.6707 |
| 6 | 21 | 5 | 0.5236 | 4 | 2.3452 | 69.07 | 61.031 | -38.474 | 23.824 | 60 | 12.8995 | 12.6395 |
| 6 | 22 | 5 | 0.5236 | 14 | 3.1623 | 285.178 | 93.329 | -22.088 | 15.682 | 20 | 55.4221 | 20.7081 |
| 6 | 23 | 4 | 0.4188 | 17.75 | 2.5 | 246.171 | 39.454 | -24.179 | 8.377 | 50 | 92.8551 | 11.5963 |
| 6 | 24 | 3 | 0.3141 | 19 | 1 | 394.625 | 195.293 | 18.787 | 12.382 | 66.667 | 87.6851 | 14.9409 |
| 6 | 25 | 3 | 0.3141 | 22.6667 | 1.1547 | 313.465 | 74.129 | 2.149 | 7.815 | 33.333 | 94.6835 | 4.2138 |
| 6 | 26 | 3 | 0.3141 | 18.6667 | 0.5774 | 465.442 | 80.707 | 8.422 | 15.424 | 33.333 | 123.6514 | 39.7907 |
| 6 | 27 | 2 | 0.2094 | 26 | 1.4142 | 636.957 | 233.629 | -5.042 | 1.255 | 0 | 111.732 | 8.5926 |
| 6 | 28 | 1 | 0.1047 | 22 | NA | 426.292 | NA | -2.693 | NA | 0 | 65.4593 | NA |
| 7 | 1 | 16 | 1.6754 | 6.625 | 2.4732 | 71.23 | 45.269 | -6.83 | 7.334 | 18.75 | 53.78 | 13.6563 |
| 7 | 2 | 14 | 1.466 | 7.7857 | 3.1422 | 109.888 | 59.754 | 6.119 | 12.823 | 35.714 | 21.1434 | 13.4451 |
| 7 | 3 | 12 | 1.2565 | 11.75 | 2.9271 | 143.686 | 62.47 | 6.79 | 5.724 | 25 | 69.9327 | 10.2889 |
| 7 | 4 | 11 | 1.1518 | 13.4545 | 2.5045 | 129.815 | 67.076 | 1.043 | 5.031 | 27.273 | 62.5511 | 10.1031 |
| 7 | 5 | 10 | 1.0471 | 12.2 | 2.0976 | 186.475 | 57.743 | -3.655 | 9.121 | 30 | 76.443 | 16.3516 |
| 7 | 6 | 9 | 0.9424 | 14.2222 | 4.1466 | 170.873 | 55.096 | -7.701 | 7.431 | 33.333 | 67.0959 | 18.0517 |
| 7 | 7 | 9 | 0.9424 | 10.2222 | 3.0322 | 185.845 | 68.68 | 7.701 | 10.995 | 44.444 | 82.4681 | 18.6799 |
| 7 | 8 | 8 | 0.8377 | 8 | 3.4641 | 124.292 | 51.921 | 5.793 | 35.157 | 50 | 22.3633 | 16.266 |
| 7 | 9 | 8 | 0.8377 | 6.75 | 0.4629 | 80.976 | 27.038 | 21.681 | 4.693 | 12.5 | 43.5821 | 10.0281 |
| 7 | 10 | 6 | 0.6283 | 20.5 | 1.5166 | 348.204 | 119.439 | 1.215 | 13.086 | 0 | 96.4678 | 23.1118 |
| 7 | 11 | 5 | 0.5236 | 14.8 | 3.9623 | 190.205 | 77.888 | 20.185 | 5.518 | 0 | 78.8375 | 7.6095 |
| 7 | 12 | 5 | 0.5236 | 18.4 | 1.5166 | 327.004 | 53.486 | -21.205 | 8.503 | 60 | 94.4203 | 13.4806 |
| 7 | 13 | 5 | 0.5236 | 9.6 | 3.5071 | 165.109 | 63.552 | -25.921 | 7.368 | 40 | 33.1299 | 13.137 |
| 7 | 14 | 5 | 0.5236 | 23 | 2.7386 | 313.952 | 49.263 | -3.581 | 9.66 | 60 | 99.9541 | 17.0172 |
| 7 | 15 | 4 | 0.4188 | 14 | 2.8284 | 228.203 | 79.303 | -26.866 | 14.593 | 50 | 93.7698 | 49.2257 |
| 7 | 16 | 4 | 0.4188 | 16 | 1.4142 | 361.983 | 148.556 | 6.406 | 7.666 | 25 | 98.9369 | 9.265 |
| 7 | 17 | 2 | 0.2094 | 19 | 0 | 304.67 | 144.397 | -4.469 | 3.965 | 0 | 75.6588 | 22.781 |

## **Appendix: USA Summary Statistics**

Table 5: Summary Statistics for USA Data

| **Area** | **Cluster** | **Trajectories** | **Frequency of Execution (%)** | **Mean Trajectory Duration (sec)** | **SD Trajectory Duration (sec)** | **Mean Change in Heading (deg)** | **SD Change in Heading (deg)** | **Mean Heading (deg)** | **SD Heading (deg)** | **Scoring Rate (%)** | **Mean distance travelled (Foot)** | **SD distance travelled (Foot)** |
| --- | --- | --- | --- | --- | --- | --- | --- | --- | --- | --- | --- | --- |
| 1 | 1 | 20 | 2.0243 | 6.05 | 2.6453 | 93.598 | 57.473 | -22.288 | 10.313 | 30 | 55.7012 | 11.8738 |
| 1 | 2 | 13 | 1.3158 | 9.9231 | 2.4311 | 172.827 | 79.154 | -2.28 | 11.786 | 61.538 | 72.0492 | 20.0137 |
| 1 | 3 | 11 | 1.1134 | 4.8182 | 2.0405 | 96.91 | 44.754 | -10.777 | 15.837 | 54.545 | 22.8386 | 13.544 |
| 1 | 4 | 8 | 0.8097 | 4 | 2.2678 | 64.251 | 45 | -52.07 | 29.175 | 50 | 9.6611 | 7.1282 |
| 1 | 5 | 7 | 0.7085 | 15.2857 | 2.8115 | 205.806 | 39.763 | -5.191 | 3.409 | 71.429 | 79.8459 | 21.6983 |
| 1 | 6 | 7 | 0.7085 | 15.2857 | 2.9841 | 109.326 | 24.036 | -16.581 | 6.486 | 28.571 | 73.463 | 14.3886 |
| 1 | 7 | 7 | 0.7085 | 7.5714 | 1.9024 | 203.411 | 53.887 | 13.287 | 5.856 | 57.143 | 71.8578 | 20.8619 |
| 1 | 8 | 6 | 0.6073 | 10.6667 | 1.8619 | 149.519 | 37.093 | -5.094 | 7.649 | 50 | 49.8177 | 10.9272 |
| 1 | 9 | 5 | 0.5061 | 14.6 | 1.6733 | 216.16 | 54.357 | -2.292 | 10.6 | 40 | 76.5825 | 12.3011 |
| 1 | 10 | 4 | 0.4049 | 18.25 | 3.5 | 403.517 | 90.734 | -11.494 | 16.948 | 50 | 106.3976 | 14.1122 |
| 1 | 11 | 3 | 0.3036 | 13.3333 | 2.0817 | 253.436 | 65.048 | 5.334 | 12.702 | 33.333 | 62.8103 | 27.2203 |
| 1 | 12 | 2 | 0.2024 | 19.5 | 3.5355 | 259.911 | 50.655 | -20.008 | 3.816 | 0 | 93.9236 | 10.8764 |
| 1 | 13 | 2 | 0.2024 | 15 | 4.2426 | 414.449 | 109.412 | -0.252 | 9.798 | 0 | 87.7541 | 22.5349 |
| 2 | 1 | 22 | 2.2267 | 9.9091 | 2.2659 | 111.979 | 34.458 | 8.382 | 7.815 | 36.364 | 59.3551 | 8.0743 |
| 2 | 2 | 19 | 1.9231 | 6.4737 | 1.7117 | 56.895 | 29.37 | -2.916 | 5.953 | 57.895 | 52.0954 | 7.7925 |
| 2 | 3 | 15 | 1.5182 | 5 | 1.4142 | 49.63 | 42.668 | -22.781 | 8.182 | 13.333 | 44.3366 | 9.6011 |
| 2 | 4 | 13 | 1.3158 | 3.7692 | 1.4233 | 22.861 | 12.175 | -15.39 | 9.437 | 0 | 31.5874 | 13.255 |
| 2 | 5 | 10 | 1.0121 | 14.3 | 3.1287 | 151.587 | 60.476 | -15.98 | 8.852 | 40 | 69.866 | 13.0882 |
| 2 | 6 | 9 | 0.9109 | 6.1111 | 3.4801 | 97.621 | 87.622 | -1.919 | 11.299 | 22.222 | 29.0282 | 23.9449 |
| 2 | 7 | 6 | 0.6073 | 7.5 | 1.9748 | 149.147 | 24.351 | -7.053 | 13.27 | 33.333 | 68.2018 | 5.2435 |
| 2 | 8 | 4 | 0.4049 | 3.25 | 0.9574 | 60.808 | 40.669 | -23.686 | 49.274 | 0 | 5.1094 | 3.0469 |
| 2 | 9 | 4 | 0.4049 | 15.5 | 3.6968 | 312.491 | 92.871 | -24.97 | 11.992 | 25 | 100.2426 | 13.6776 |
| 2 | 10 | 3 | 0.3036 | 16.3333 | 2.5166 | 220.245 | 31.203 | 9.672 | 6.205 | 33.333 | 72.4304 | 9.847 |
| 2 | 11 | 2 | 0.2024 | 22 | 0 | 347.843 | 89.147 | 13.361 | 19.349 | 50 | 115.934 | 12.1742 |
| 2 | 12 | 1 | 0.1012 | 25 | NA | 567.755 | NA | -2.252 | NA | 0 | 119.8761 | NA |
| 3 | 1 | 33 | 3.3401 | 7.2727 | 2.7417 | 113.308 | 50.558 | -1.627 | 13.086 | 48.485 | 61.2588 | 10.7061 |
| 3 | 2 | 27 | 2.7328 | 5.2963 | 1.9771 | 90.47 | 58.316 | -29.41 | 8.325 | 44.444 | 46.7105 | 13.9207 |
| 3 | 3 | 23 | 2.3279 | 5.087 | 1.24 | 65.942 | 27.124 | -19.228 | 15.344 | 34.783 | 62.6889 | 14.4552 |
| 3 | 4 | 20 | 2.0243 | 7.6 | 1.4654 | 88.207 | 33.701 | -28.493 | 6.996 | 30 | 69.1075 | 6.5341 |
| 3 | 5 | 18 | 1.8219 | 11.8333 | 1.9478 | 183.919 | 95.896 | -11.694 | 12.588 | 27.778 | 70.967 | 8.3356 |
| 3 | 6 | 16 | 1.6194 | 11.0625 | 2.2051 | 176.74 | 69.454 | 4.337 | 14.948 | 62.5 | 77.3471 | 7.6255 |
| 3 | 7 | 14 | 1.417 | 15.5 | 1.9115 | 222.491 | 48.237 | 0.183 | 6.36 | 28.571 | 75.2177 | 8.8949 |
| 3 | 8 | 12 | 1.2146 | 14.0833 | 2.811 | 266.443 | 97.798 | -18.335 | 12.29 | 58.333 | 87.698 | 13.8491 |
| 3 | 9 | 10 | 1.0121 | 19.1 | 2.6854 | 227.533 | 68.658 | -13.178 | 7.609 | 20 | 79.9283 | 7.9681 |
| 3 | 10 | 9 | 0.9109 | 14.7778 | 2.1667 | 189.053 | 101.981 | -12.462 | 8.182 | 66.667 | 72.3281 | 10.3511 |
| 3 | 11 | 8 | 0.8097 | 6.5 | 3.7417 | 152.831 | 93.054 | 2.613 | 16.868 | 25 | 23.8608 | 14.7192 |
| 3 | 12 | 7 | 0.7085 | 4.2857 | 2.0587 | 108.77 | 94.578 | -46.736 | 19.836 | 71.429 | 17.7603 | 18.3464 |
| 3 | 13 | 7 | 0.7085 | 12.7143 | 1.8898 | 228.117 | 68.749 | -5.787 | 12.301 | 85.714 | 72.642 | 18.8015 |
| 3 | 14 | 6 | 0.6073 | 21 | 0.6325 | 208.476 | 52.047 | 2.246 | 7.128 | 50 | 92.3284 | 10.1529 |
| 3 | 15 | 4 | 0.4049 | 17 | 3.8297 | 363.937 | 256.771 | 2.492 | 10.749 | 25 | 103.7426 | 43.5215 |
| 3 | 16 | 3 | 0.3036 | 12.6667 | 2.0817 | 240.585 | 78.014 | 19.051 | 9.924 | 33.333 | 51.903 | 6.2563 |
| 4 | 1 | 13 | 1.3158 | 12.6923 | 3.3263 | 135.694 | 63.799 | -3.856 | 8.165 | 7.692 | 68.0254 | 16.5048 |
| 4 | 2 | 12 | 1.2146 | 7 | 2.6285 | 85.915 | 45.298 | 2.086 | 6.704 | 33.333 | 46.9761 | 17.342 |
| 4 | 3 | 11 | 1.1134 | 6.9091 | 2.8445 | 50.18 | 24.356 | 21.154 | 7.328 | 9.091 | 50.2472 | 10.9365 |
| 4 | 4 | 8 | 0.8097 | 4.875 | 1.5526 | 24.798 | 14.937 | 14.318 | 10.136 | 0 | 28.6282 | 12.8686 |
| 4 | 5 | 7 | 0.7085 | 4.2857 | 1.3801 | 63.965 | 46.427 | 5.535 | 26.551 | 42.857 | 10.3818 | 5.9537 |
| 4 | 6 | 6 | 0.6073 | 11.3333 | 1.633 | 91.186 | 24.838 | 1.845 | 8.818 | 0 | 54.2332 | 17.1278 |
| 4 | 7 | 3 | 0.3036 | 16.6667 | 2.5166 | 262.867 | 79.423 | 11.144 | 10.892 | 33.333 | 75.8548 | 10.1051 |
| 4 | 8 | 3 | 0.3036 | 13 | 2.6458 | 244.183 | 40.187 | -11.007 | 29.536 | 66.667 | 95.9828 | 31.4395 |
| 4 | 9 | 3 | 0.3036 | 18 | 4.3589 | 204.678 | 76.822 | 2.137 | 8.331 | 0 | 67.3479 | 20.7807 |
| 4 | 10 | 1 | 0.1012 | 17 | NA | 257.545 | NA | -8.256 | NA | 0 | 92.835 | NA |
| 5 | 1 | 34 | 3.4413 | 5.1471 | 1.6168 | 96.412 | 42.015 | 4.28 | 10.605 | 58.824 | 57.2928 | 7.9352 |
| 5 | 2 | 28 | 2.834 | 7.4643 | 2.2523 | 98.526 | 55.892 | 27.044 | 8.984 | 46.429 | 58.1515 | 9.1316 |
| 5 | 3 | 23 | 2.3279 | 10.1739 | 2.2893 | 154.578 | 106.026 | 13.86 | 7.477 | 52.174 | 75.4856 | 11.9856 |
| 5 | 4 | 21 | 2.1255 | 9 | 1.9494 | 176.23 | 72.542 | -7.918 | 12.21 | 38.095 | 68.3068 | 9.6019 |
| 5 | 5 | 18 | 1.8219 | 7.3889 | 1.883 | 106.914 | 31.501 | -5.036 | 8.738 | 33.333 | 51.5385 | 10.6304 |
| 5 | 6 | 17 | 1.7206 | 5.8235 | 2.4299 | 124.979 | 87.439 | 15.264 | 18.243 | 52.941 | 41.0579 | 16.3921 |
| 5 | 7 | 13 | 1.3158 | 3.8462 | 1.9081 | 49.784 | 44.702 | 42.485 | 35.162 | 30.769 | 10.6777 | 9.8242 |
| 5 | 8 | 13 | 1.3158 | 8.7692 | 2.0878 | 106.547 | 41.156 | 28.31 | 7.024 | 69.231 | 72.4135 | 5.3765 |
| 5 | 9 | 13 | 1.3158 | 13.5385 | 1.8536 | 234.03 | 44.502 | 10.285 | 9.139 | 61.538 | 74.9482 | 8.1459 |
| 5 | 10 | 12 | 1.2146 | 15.8333 | 2.4802 | 247.988 | 65.781 | 5.712 | 7.007 | 41.667 | 87.2074 | 8.5617 |
| 5 | 11 | 12 | 1.2146 | 4.25 | 2.0944 | 80.598 | 59.192 | 26.946 | 15.584 | 66.667 | 20.9095 | 8.8992 |
| 5 | 12 | 9 | 0.9109 | 12.6667 | 1.5811 | 161.981 | 60.619 | 0.733 | 10.72 | 11.111 | 72.0821 | 7.2962 |
| 5 | 13 | 9 | 0.9109 | 16.2222 | 2.2236 | 204.724 | 46.719 | -3.621 | 9.047 | 44.444 | 83.0448 | 19.1891 |
| 5 | 14 | 9 | 0.9109 | 12.1111 | 1.2693 | 211.57 | 34.899 | 6.188 | 9.333 | 11.111 | 81.1608 | 13.1163 |
| 5 | 15 | 9 | 0.9109 | 14.3333 | 1.9365 | 192.325 | 62.424 | 19.423 | 9.07 | 55.556 | 73.4793 | 11.5675 |
| 5 | 16 | 7 | 0.7085 | 15.7143 | 2.2887 | 241.21 | 75.94 | -14.032 | 7.128 | 28.571 | 91.161 | 15.4217 |
| 5 | 17 | 7 | 0.7085 | 14.8571 | 1.5736 | 148.648 | 52.62 | 7.935 | 7.225 | 71.429 | 79.224 | 10.1024 |
| 5 | 18 | 7 | 0.7085 | 4.2857 | 1.1127 | 74.639 | 41.379 | 16.129 | 4.561 | 71.429 | 71.8472 | 5.0319 |
| 5 | 19 | 7 | 0.7085 | 19.1429 | 2.7946 | 335.73 | 70.394 | -2.802 | 11.608 | 42.857 | 91.8544 | 11.2609 |
| 5 | 20 | 7 | 0.7085 | 10.4286 | 1.3973 | 201.383 | 59.759 | -14.227 | 7.764 | 57.143 | 67.6905 | 15.155 |
| 5 | 21 | 7 | 0.7085 | 21.7143 | 1.7995 | 264.174 | 112.139 | 4.326 | 6.864 | 28.571 | 82.2302 | 8.7582 |
| 5 | 22 | 6 | 0.6073 | 11.8333 | 2.6394 | 201.062 | 42.811 | 24.253 | 7.133 | 50 | 83.8955 | 6.8451 |
| 5 | 23 | 6 | 0.6073 | 14.6667 | 2.1602 | 363.307 | 164.553 | -8.382 | 12.313 | 66.667 | 108.3385 | 12.9818 |
| 5 | 24 | 6 | 0.6073 | 11.6667 | 2.8048 | 243.547 | 141.343 | 2.974 | 23.938 | 16.667 | 36.3346 | 18.1364 |
| 5 | 25 | 5 | 0.5061 | 19.2 | 2.8636 | 166.066 | 36.337 | -0.052 | 12.296 | 20 | 86.6945 | 13.3099 |
| 5 | 26 | 5 | 0.5061 | 16 | 1.8708 | 184.263 | 49.761 | -13.155 | 3.644 | 20 | 52.3727 | 7.9208 |
| 5 | 27 | 3 | 0.3036 | 19 | 2 | 224.983 | 113.514 | 20.225 | 8.955 | 0 | 96.1516 | 1.7215 |
| 5 | 28 | 2 | 0.2024 | 21 | 0 | 493.701 | 238.608 | 18.232 | 3.73 | 50 | 103.2346 | 34.5304 |
| 5 | 29 | 2 | 0.2024 | 15.5 | 3.5355 | 458.234 | 97.477 | 11.167 | 6.692 | 50 | 70.0259 | 24.1521 |
| 6 | 1 | 23 | 2.3279 | 7.4783 | 2.4471 | 90.058 | 48.1 | -13.063 | 6.618 | 34.783 | 56.8576 | 8.7835 |
| 6 | 2 | 20 | 2.0243 | 6.9 | 2.125 | 93.077 | 45.802 | 5.999 | 11.86 | 10 | 46.7477 | 12.493 |
| 6 | 3 | 17 | 1.7206 | 7.7059 | 2.2849 | 92.498 | 46.604 | 5.707 | 9.311 | 23.529 | 57.5946 | 7.6458 |
| 6 | 4 | 9 | 0.9109 | 2.8889 | 1.9003 | 20.088 | 19.887 | -0.917 | 6.824 | 0 | 29.454 | 12.5681 |
| 6 | 5 | 9 | 0.9109 | 12.7778 | 2.5874 | 161.402 | 47.968 | 9.597 | 11.665 | 33.333 | 75.2188 | 15.24 |
| 6 | 6 | 6 | 0.6073 | 11.1667 | 2.7142 | 85.199 | 59.983 | 7.139 | 7.133 | 33.333 | 55.2405 | 7.0881 |
| 6 | 7 | 6 | 0.6073 | 10 | 2.8983 | 172.924 | 22.145 | -0.596 | 2.893 | 50 | 61.1075 | 8.9678 |
| 6 | 8 | 5 | 0.5061 | 20 | 1 | 219.529 | 75.722 | 0.579 | 5.523 | 0 | 78.5738 | 15.4205 |
| 6 | 9 | 5 | 0.5061 | 5.2 | 2.1679 | 35.667 | 19.549 | -1.22 | 17.183 | 20 | 8.0385 | 2.1023 |
| 6 | 10 | 4 | 0.4049 | 15.25 | 0.9574 | 245.14 | 41.499 | 14.507 | 9.036 | 25 | 86.351 | 26.0405 |
| 6 | 11 | 4 | 0.4049 | 6.5 | 2.3805 | 112.391 | 26.963 | -14.954 | 46.559 | 0 | 24.3667 | 12.688 |
| 6 | 12 | 3 | 0.3036 | 12.3333 | 2.5166 | 305.627 | 87.147 | -0.344 | 3.323 | 33.333 | 85.9555 | 8.339 |
| 6 | 13 | 3 | 0.3036 | 15.6667 | 1.5275 | 290.61 | 118.379 | -14.152 | 4.051 | 0 | 97.3754 | 11.0001 |
| 6 | 14 | 3 | 0.3036 | 13.3333 | 7.5719 | 42.559 | 35.036 | 2.108 | 6.176 | 0 | 52.1018 | 4.8089 |
| 6 | 15 | 1 | 0.1012 | 18 | NA | 302.9 | NA | -24.901 | NA | 0 | 130.9176 | NA |
| 7 | 1 | 13 | 1.3158 | 6.6923 | 3.4733 | 87.078 | 55.995 | 22.466 | 11.78 | 30.769 | 53.5965 | 19.8069 |
| 7 | 2 | 10 | 1.0121 | 11.6 | 2.4585 | 146.591 | 99.729 | 17.533 | 10.267 | 60 | 63.5848 | 12.3199 |
| 7 | 3 | 9 | 0.9109 | 14.4444 | 2.6034 | 172.598 | 44.622 | -1.18 | 6.096 | 55.556 | 72.8345 | 12.8563 |
| 7 | 4 | 6 | 0.6073 | 16.8333 | 1.9408 | 247.008 | 81.572 | 11.728 | 6.079 | 33.333 | 96.6024 | 11.07 |
| 7 | 5 | 6 | 0.6073 | 6.8333 | 1.7224 | 111.727 | 41.889 | 0.911 | 10.795 | 0 | 55.197 | 6.9028 |
| 7 | 6 | 5 | 0.5061 | 10.6 | 3.2863 | 174.609 | 123.364 | 12.003 | 15.951 | 60 | 30.8633 | 10.3655 |
| 7 | 7 | 5 | 0.5061 | 10.4 | 3.9115 | 308.366 | 84.729 | -11.316 | 15.201 | 80 | 85.2852 | 13.041 |
| 7 | 8 | 4 | 0.4049 | 13.5 | 4.1231 | 364.957 | 114.03 | 4.693 | 16.146 | 0 | 88.7557 | 17.4091 |
| 7 | 9 | 3 | 0.3036 | 2.6667 | 0.5774 | 32.573 | 15.424 | 66.675 | 6.4 | 66.667 | 10.3458 | 8.5221 |
| 7 | 10 | 3 | 0.3036 | 20 | 4.5826 | 146.013 | 44.857 | 7.391 | 15.59 | 33.333 | 68.6284 | 4.598 |
| 7 | 11 | 1 | 0.1012 | 16 | NA | 535.4 | NA | -2.372 | NA | 0 | 69.6511 | NA |
| 7 | 12 | 1 | 0.1012 | 19 | NA | 438.479 | NA | 2.618 | NA | 0 | 104.5046 | NA |
