# Supplementary material for: Classifying ball trajectories in invasion sports using dynamic time warping: A basketball case study
Source: PLoS One. 2022 Oct 20;17(10):e0272848. doi: 10.1371/journal.pone.0272848 (PMC9584368; doi:10.1371/journal.pone.0272848)

AUS Area 1 with cluster c(20, 20, 50, 50, 50, 20, 50) Cluster dendrogram

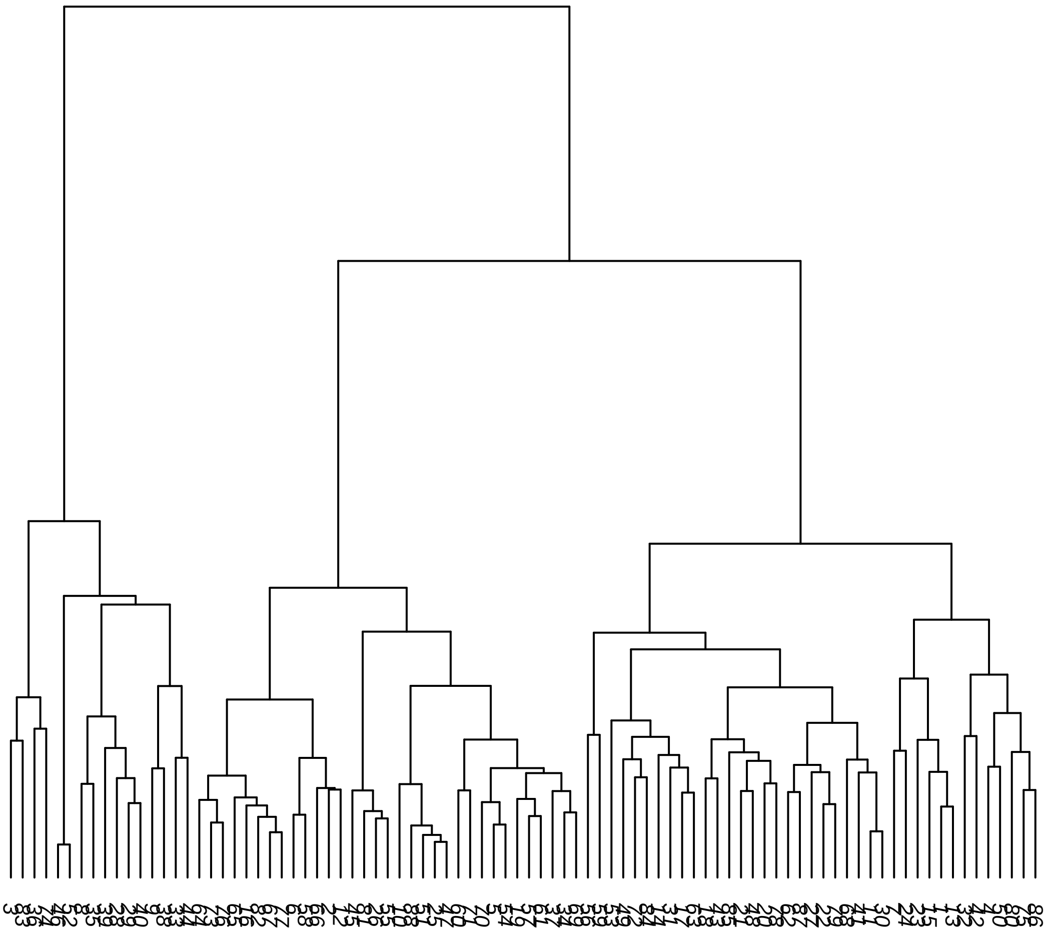

AUS Area 1 Unrooted Cluster dendrogram

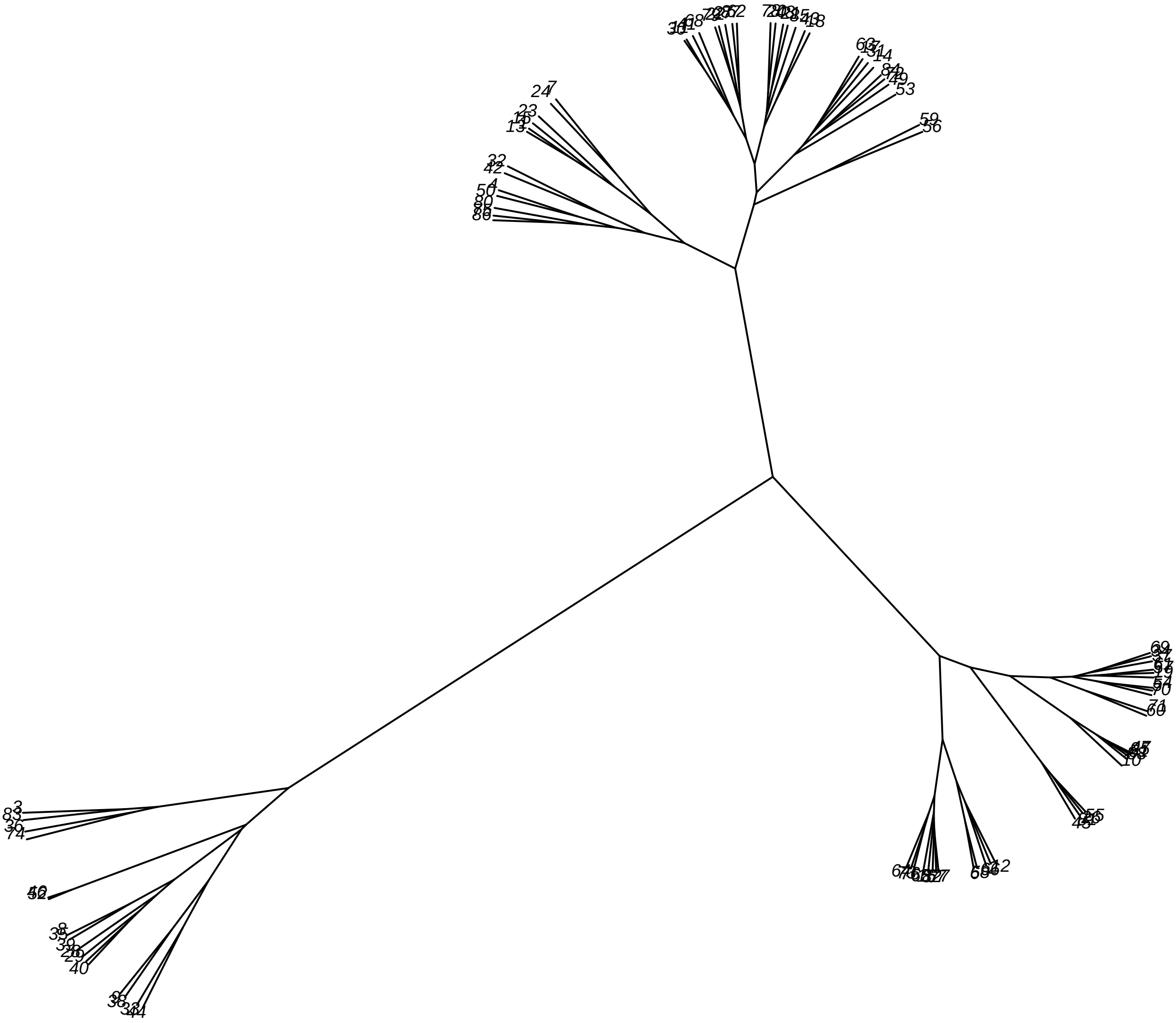

AUS Area 1 Fan Cluster dendrogram

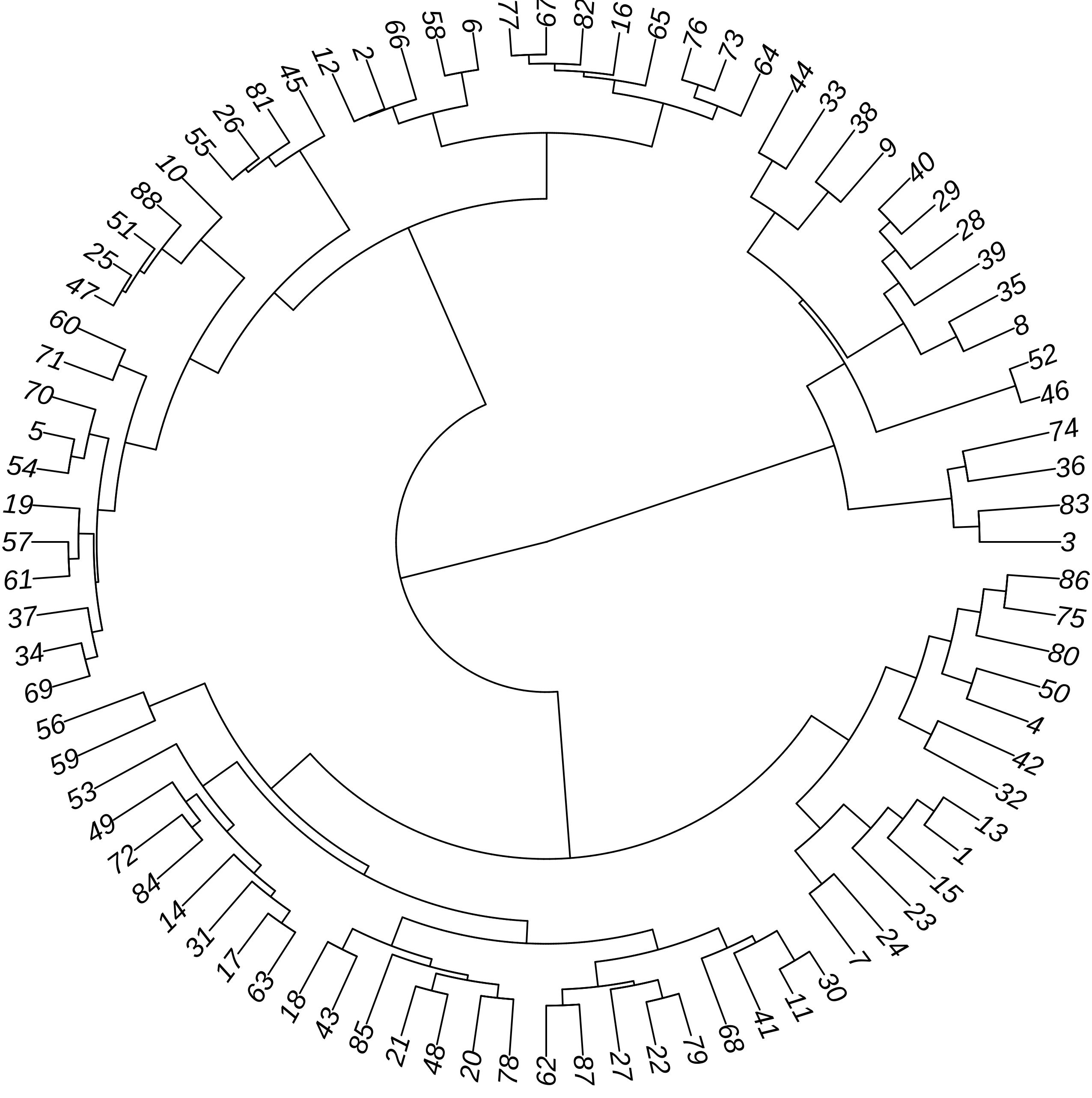

AUS Area 1 at h = 15 : Cluster dendrogram

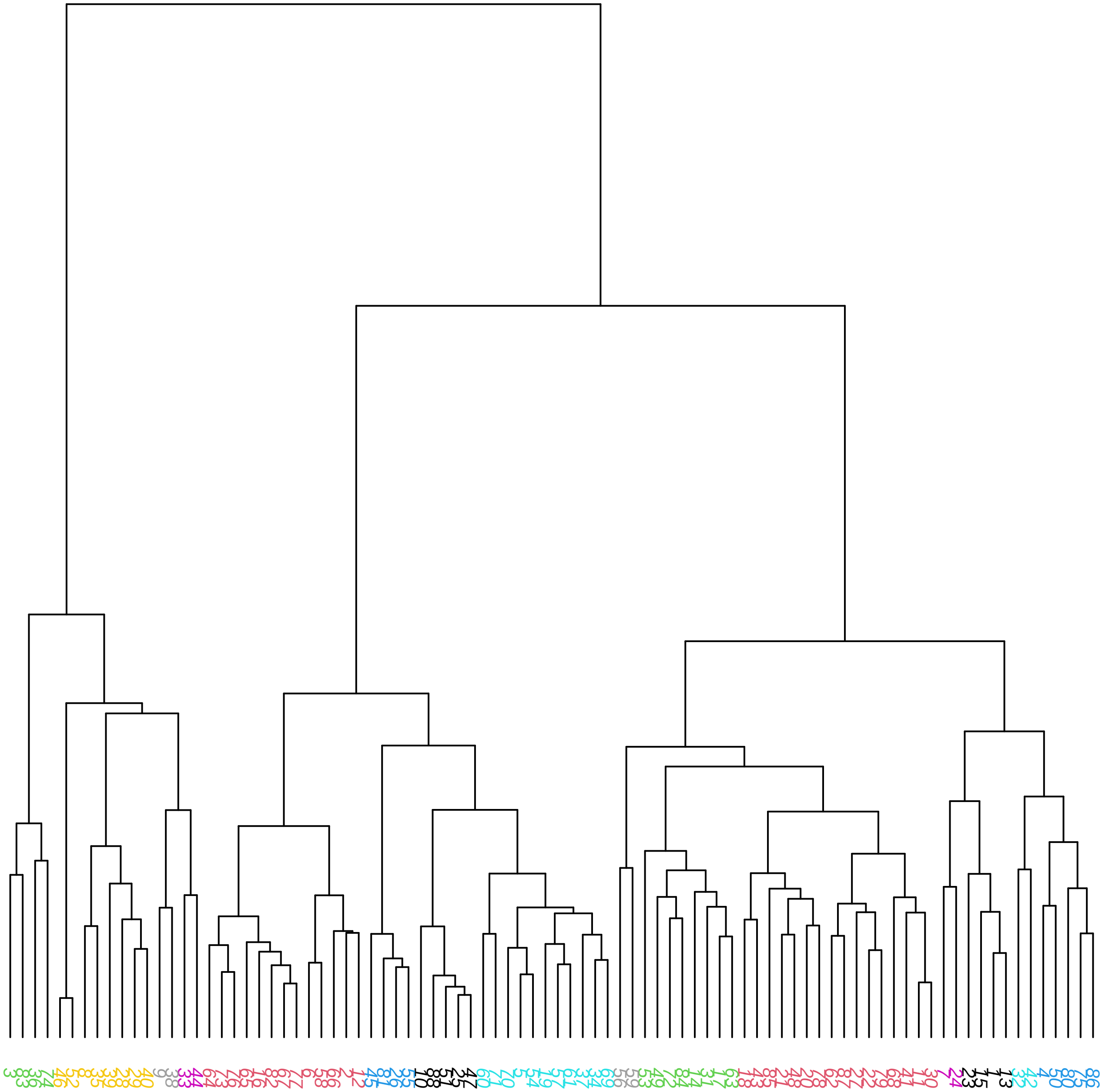

AUS Area 1 at h = 15 : Coloured Unrooted Cluster dendrogram

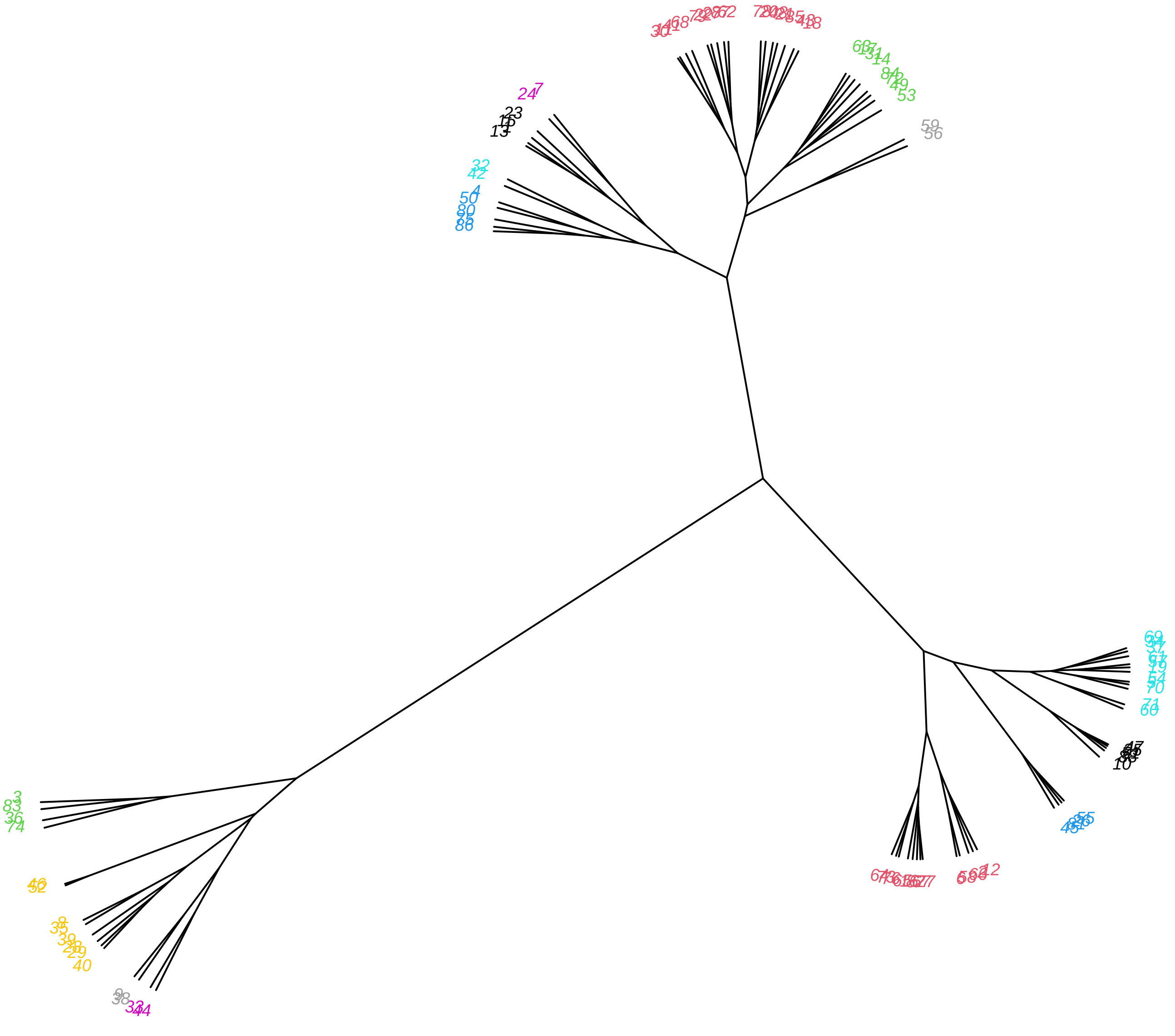

AUS Area 1 at h = 15 : Coloured Fan Cluster dendrogram

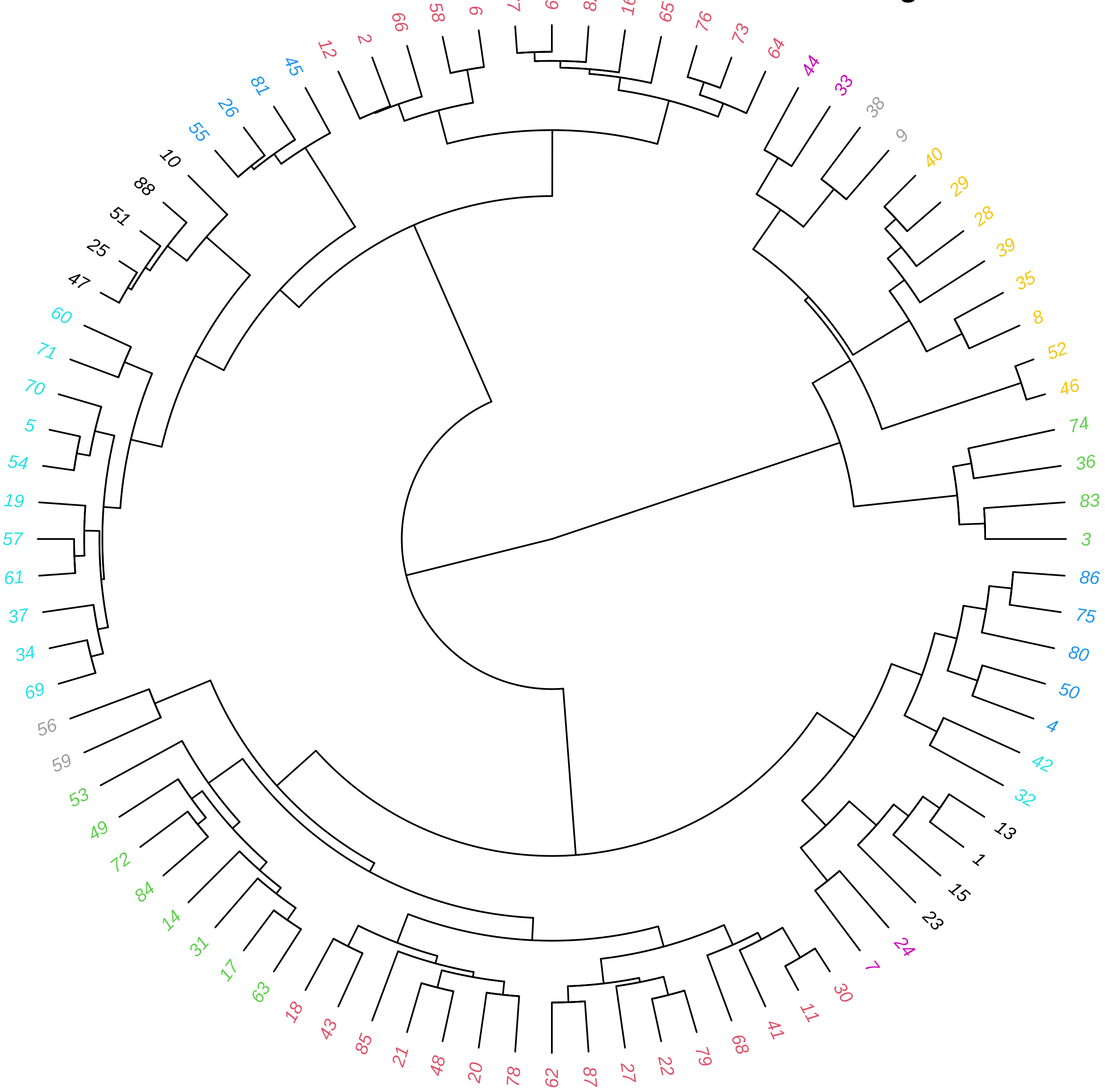

AUS Area 2 with cluster c(20, 20, 50, 50, 50, 20, 50) Cluster dendrogram

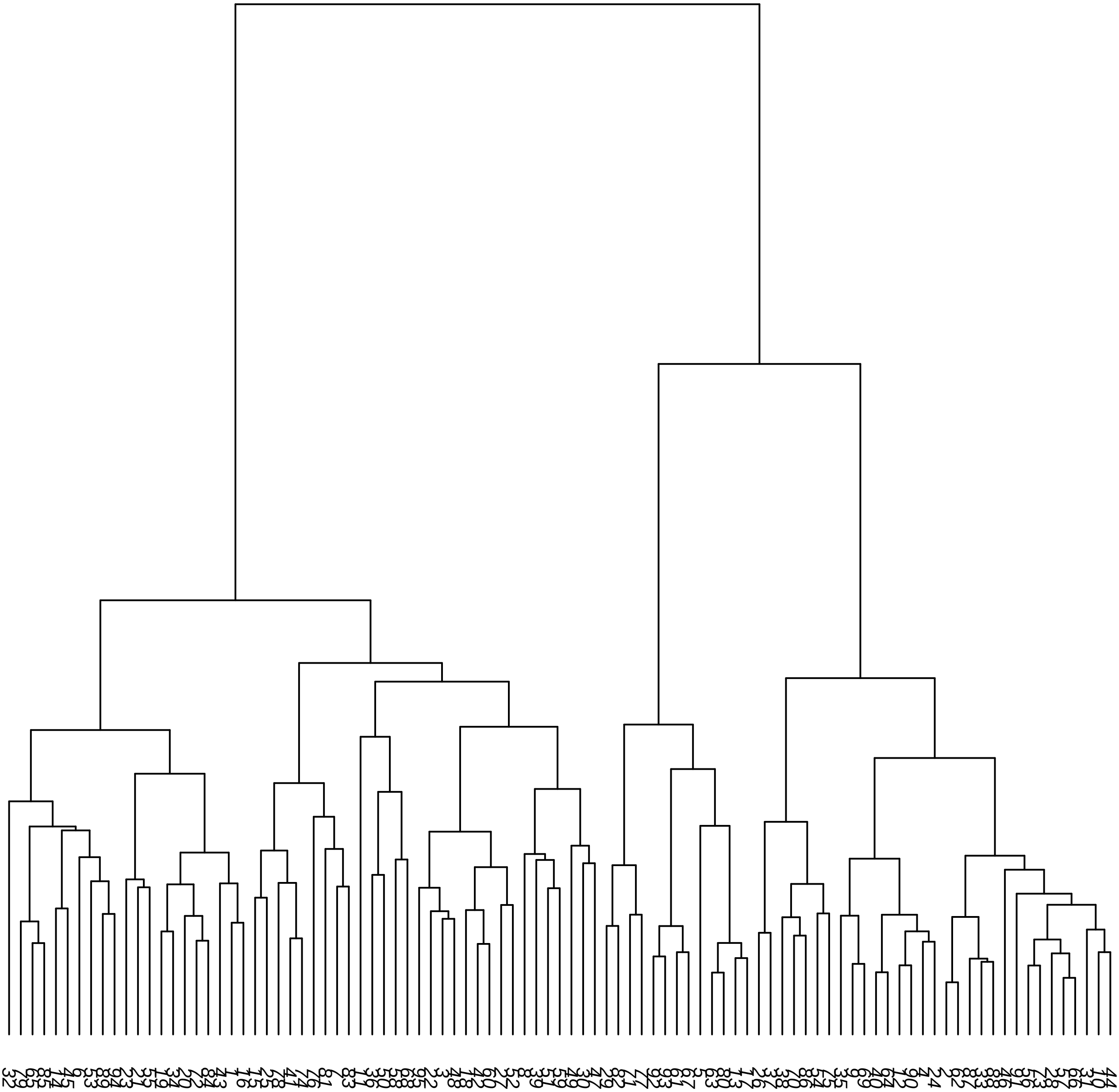

AUS Area 2 Unrooted Cluster dendrogram

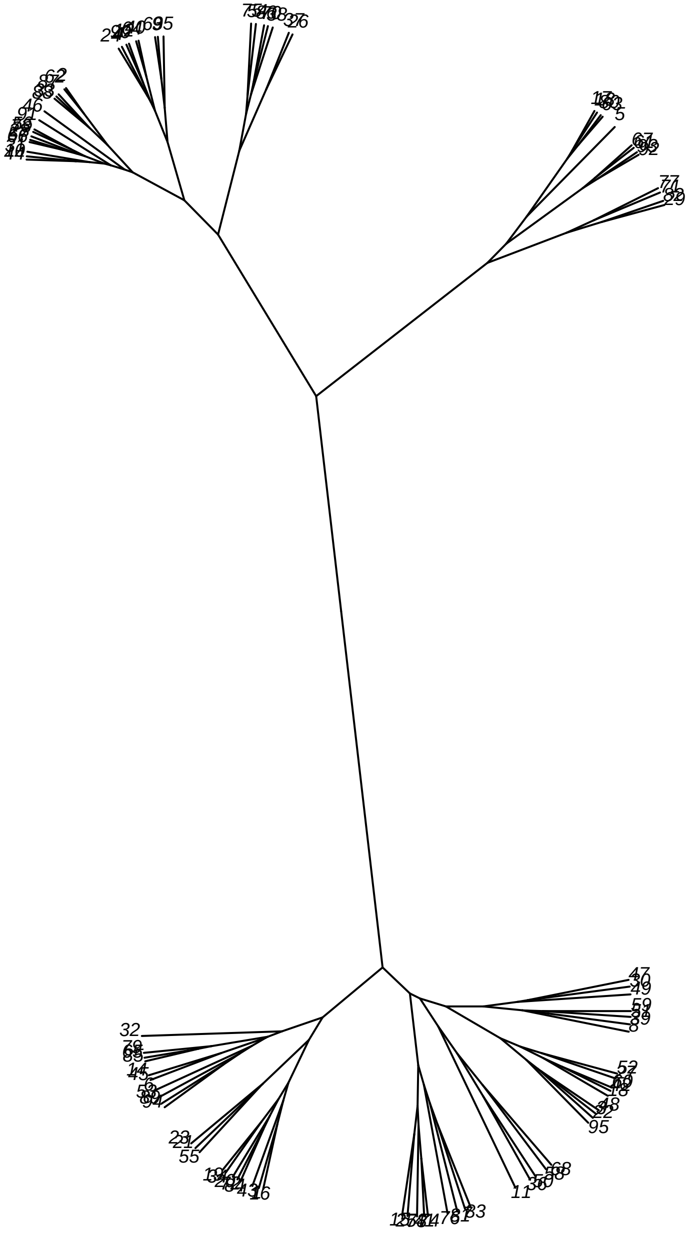

AUS Area 2 Fan Cluster dendrogram

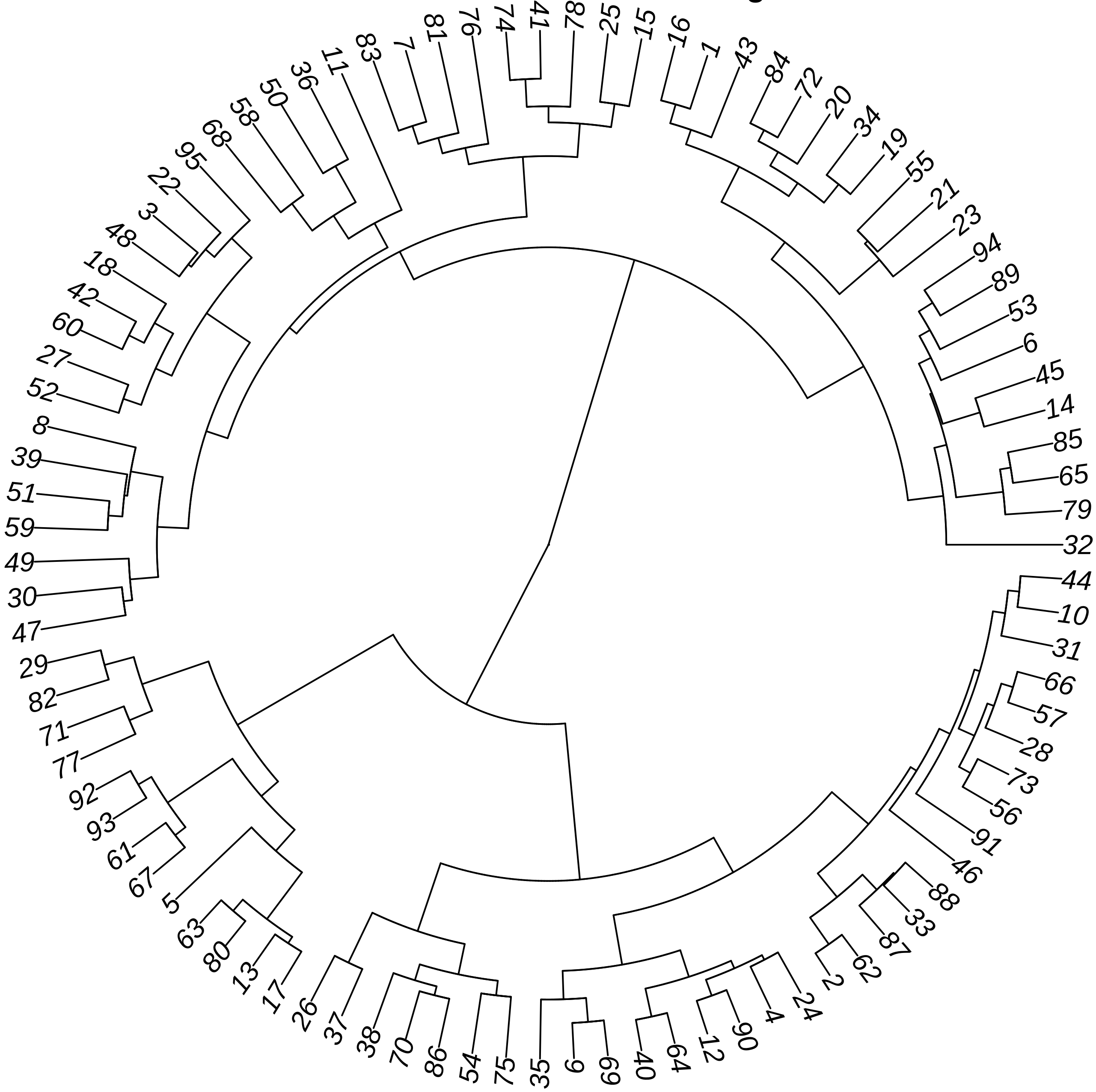

AUS Area 2 at h = 15 : Cluster dendrogram

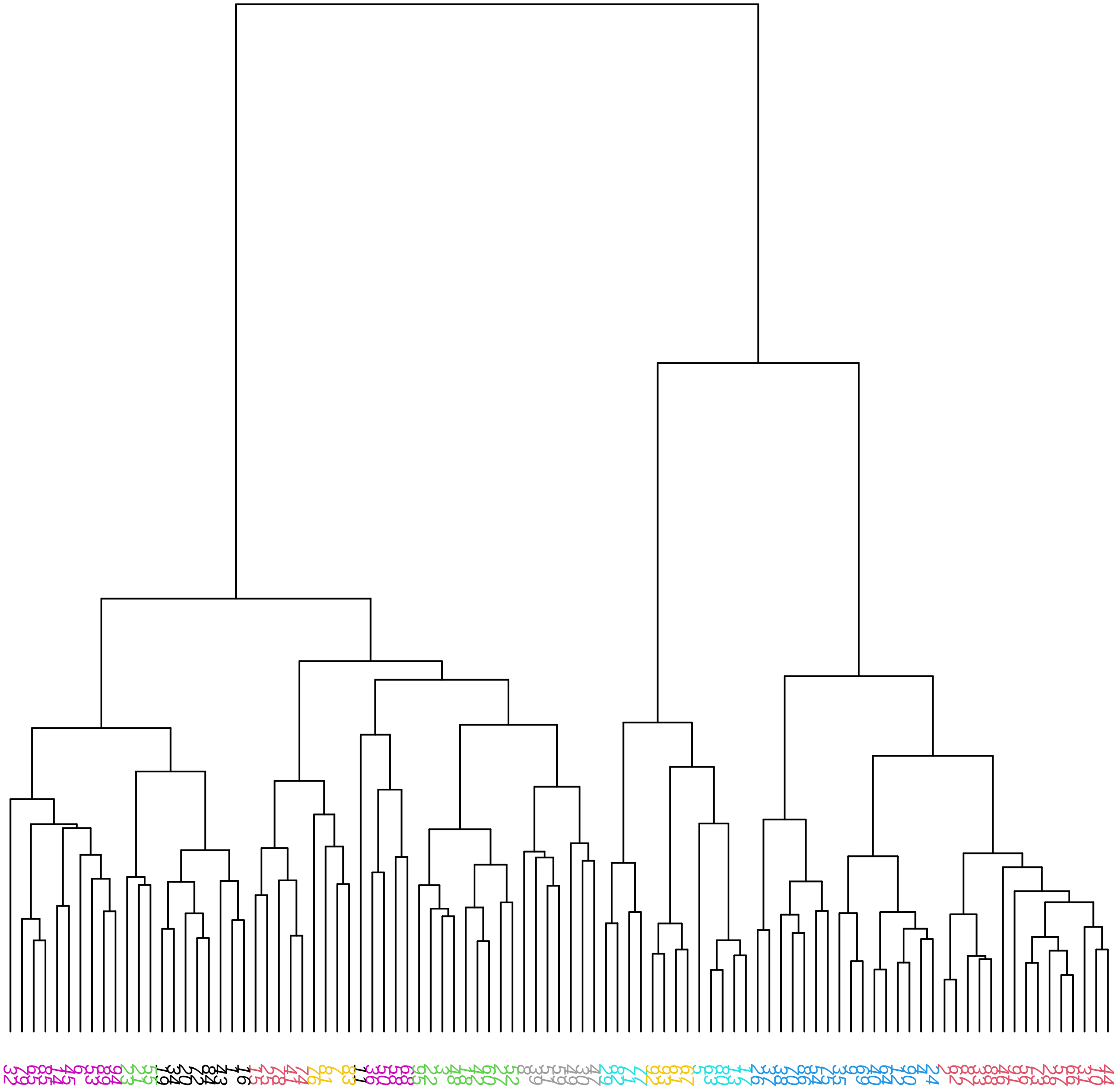

AUS Area 2 at h = 15 : Coloured Unrooted Cluster dendrogram

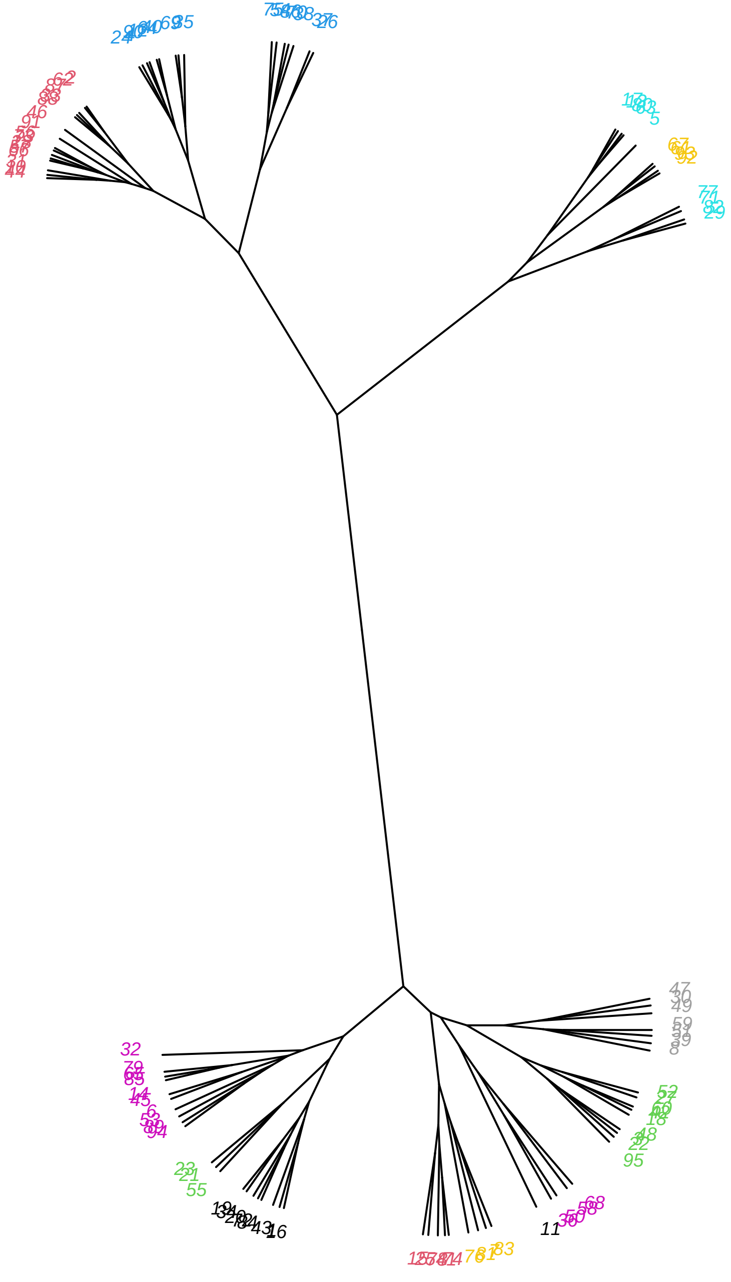

AUS Area 2 at h = 15 : Coloured Fan Cluster dendrogram

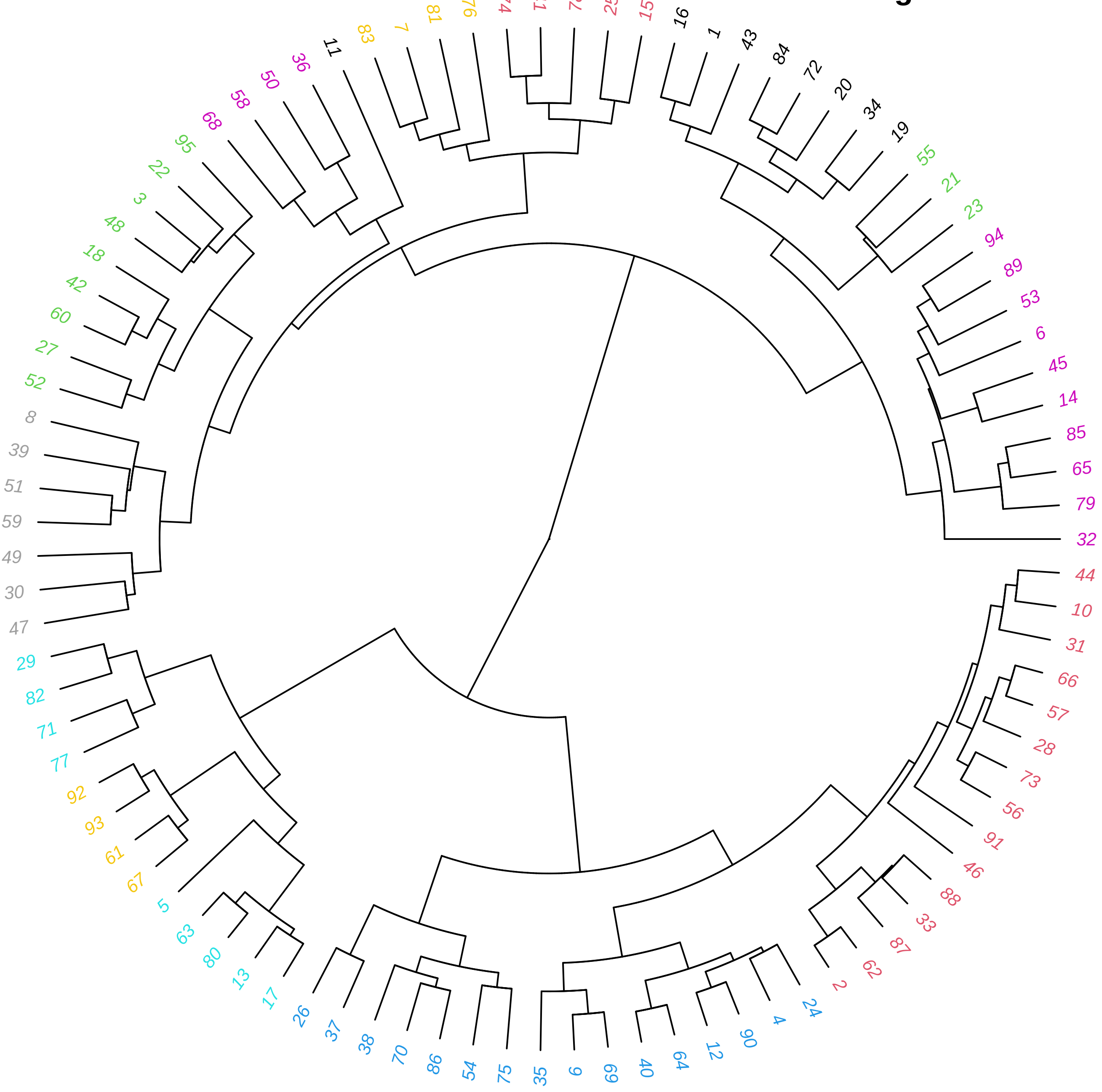

AUS Area 3 with cluster c(20, 20, 50, 50, 50, 20, 50) Cluster dendrogram

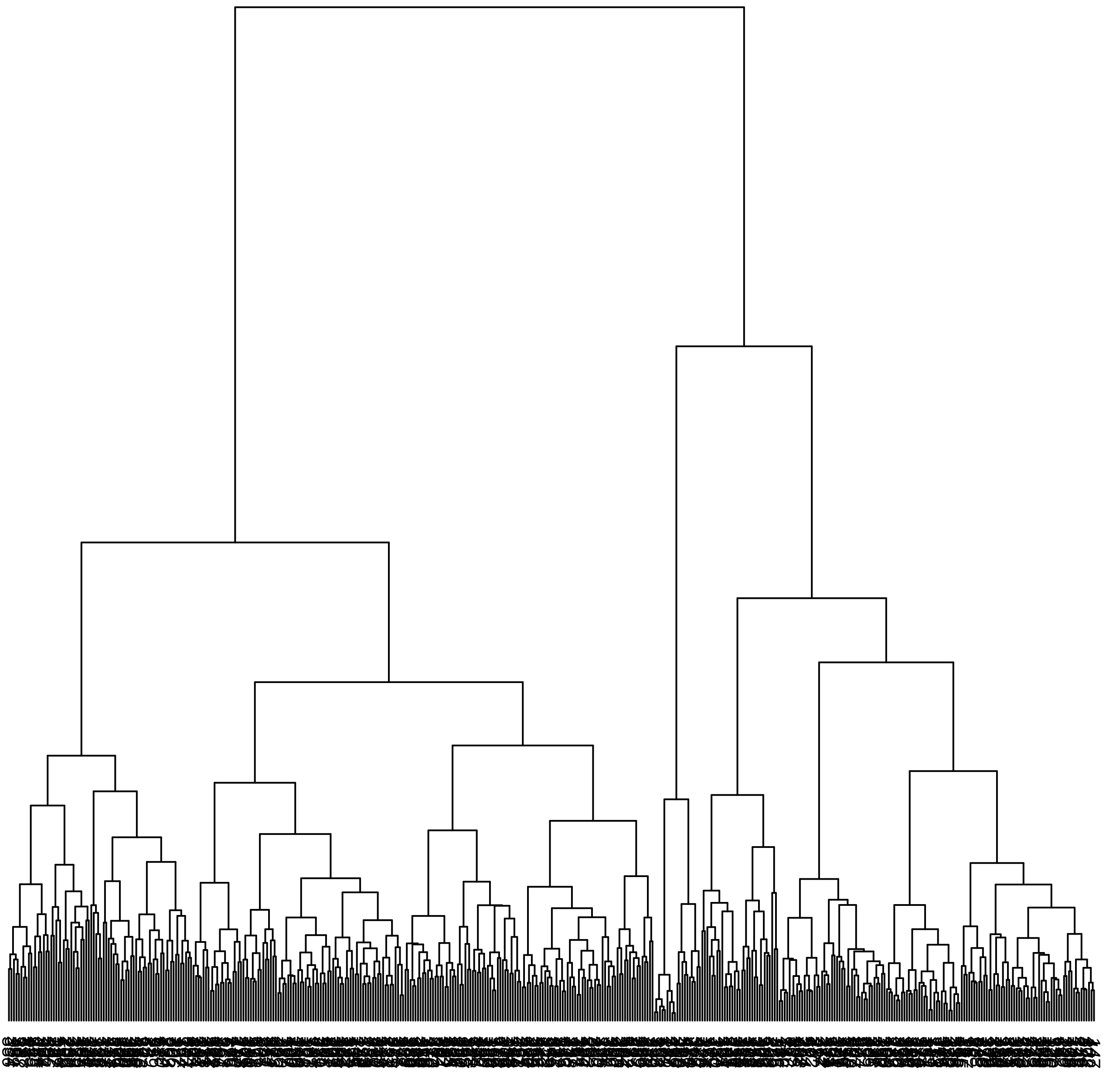

AUS Area 3 Unrooted Cluster dendrogram

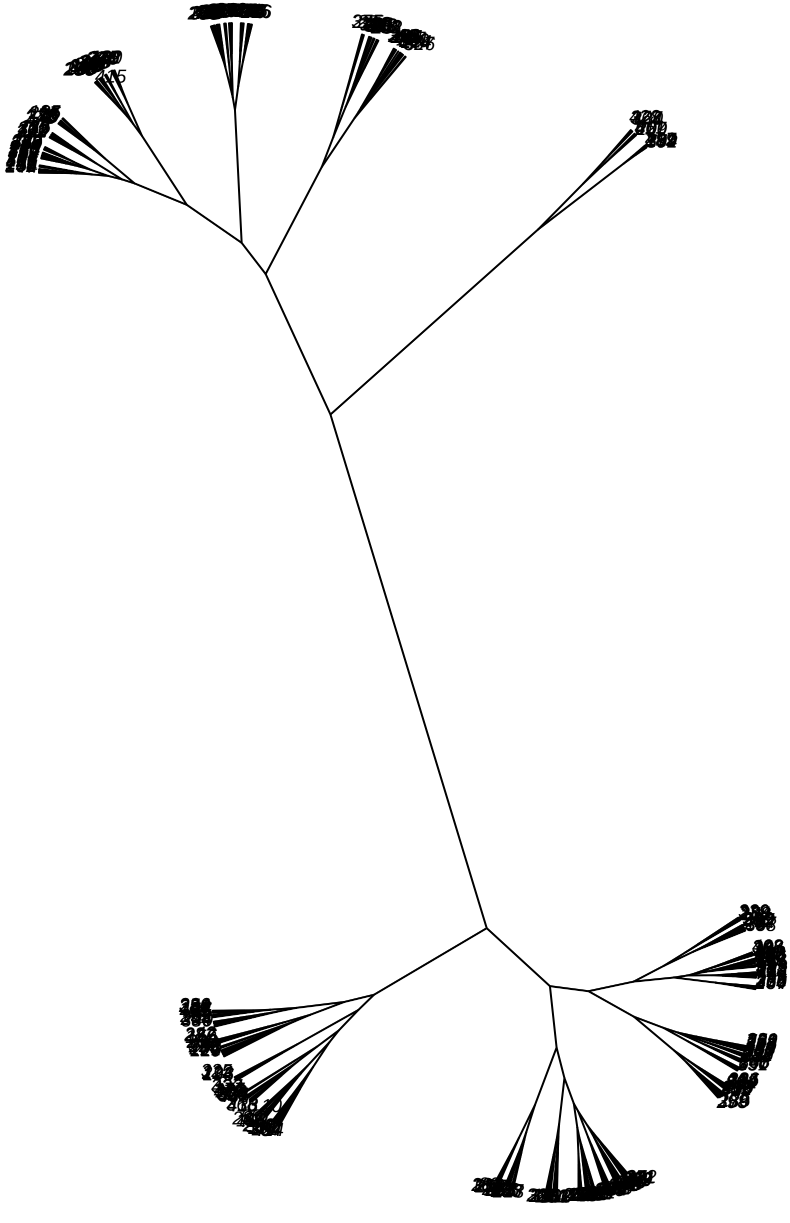

AUS Area 3 Fan Cluster dendrogram

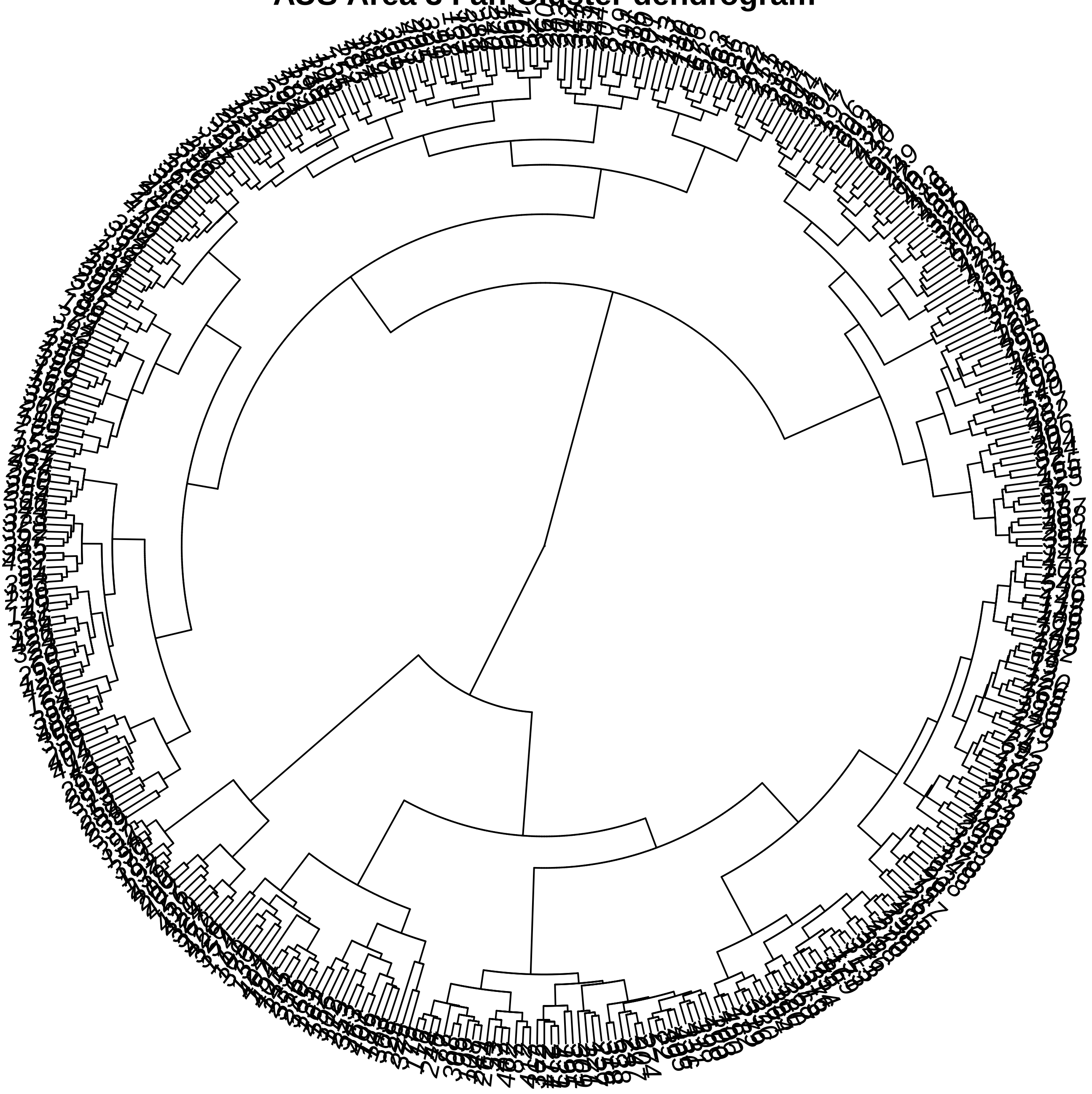

AUS Area 3 at h = 15 : Cluster dendrogram

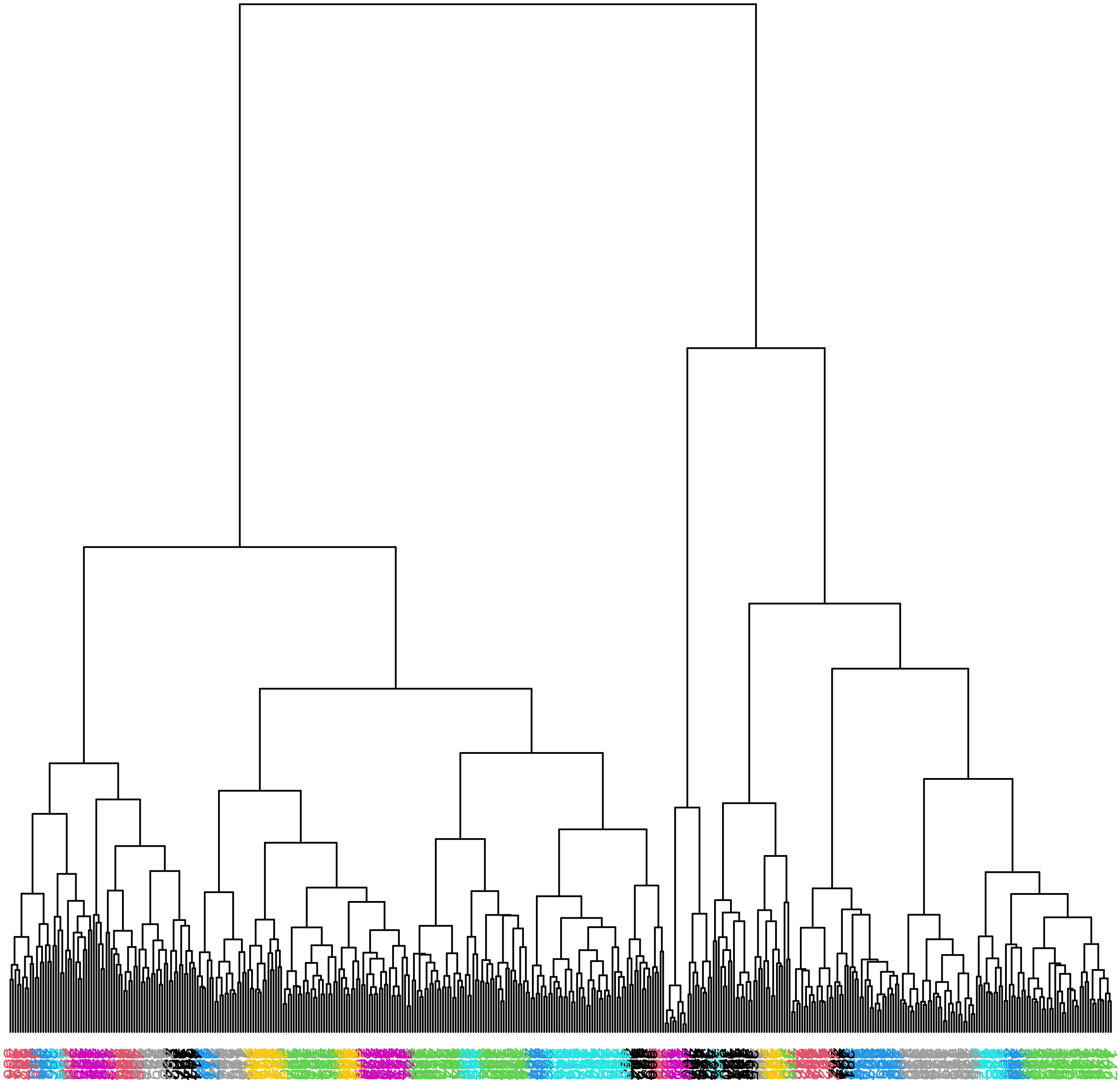

AUS Area 3 at h = 15 : Coloured Unrooted Cluster dendrogram

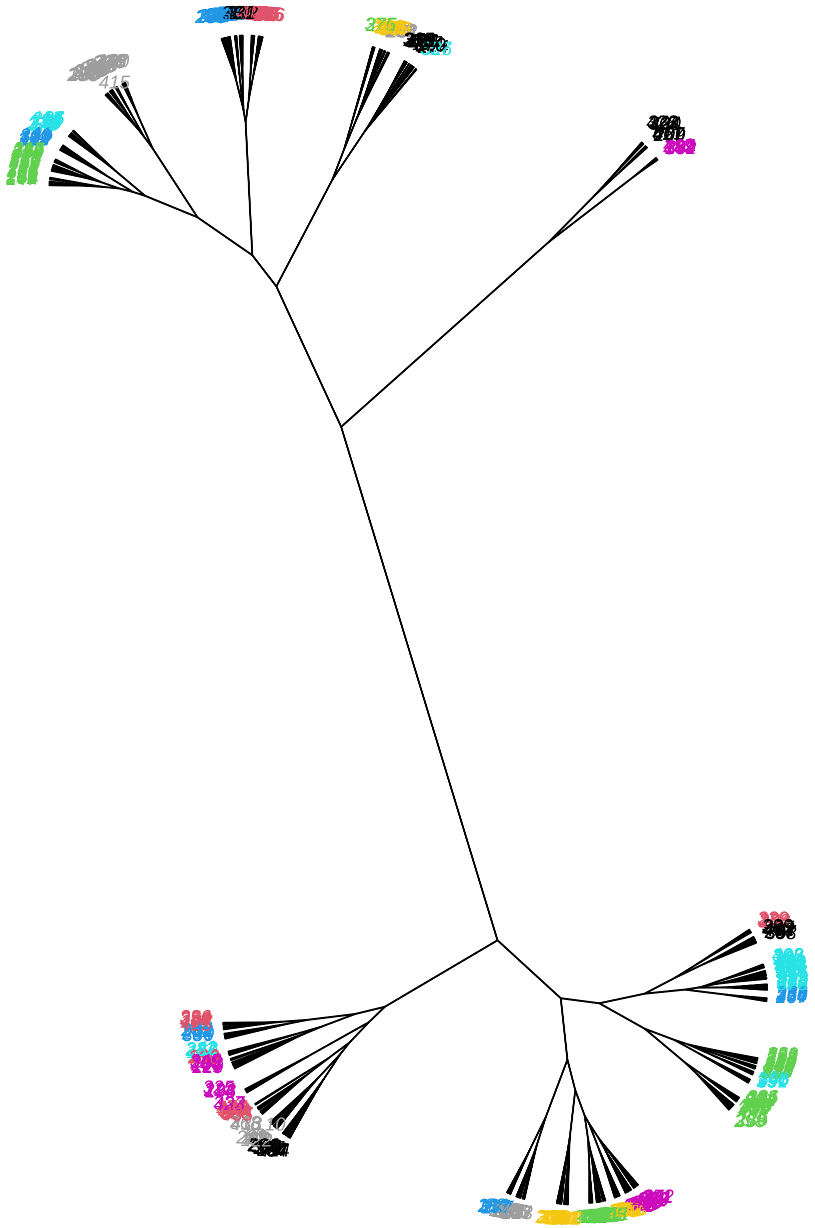

AUS Area 3 at h = 15 : Coloured Fan Cluster dendrogram

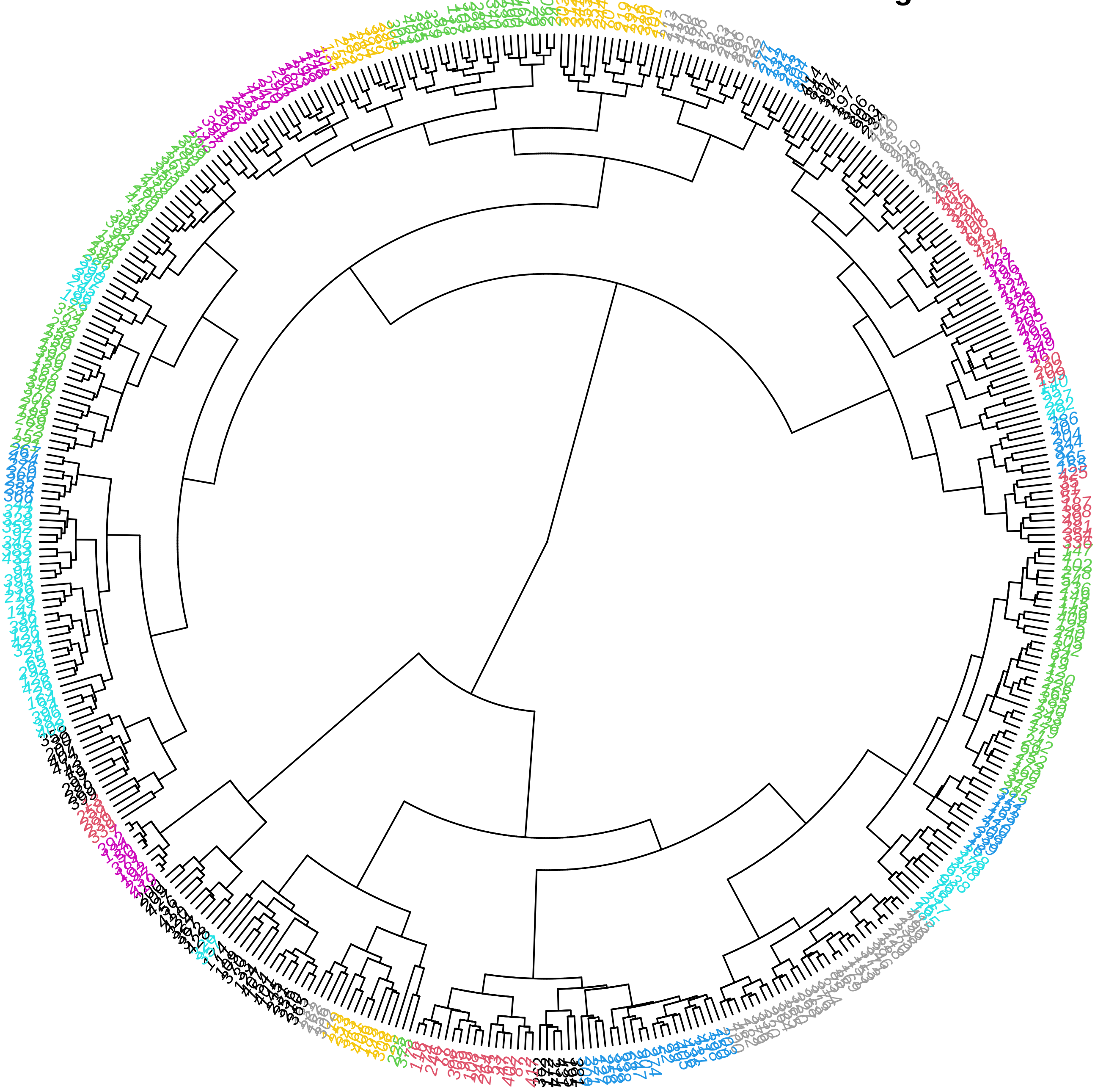

AUS Area 4 with cluster c(20, 20, 50, 50, 50, 20, 50) Cluster dendrogram

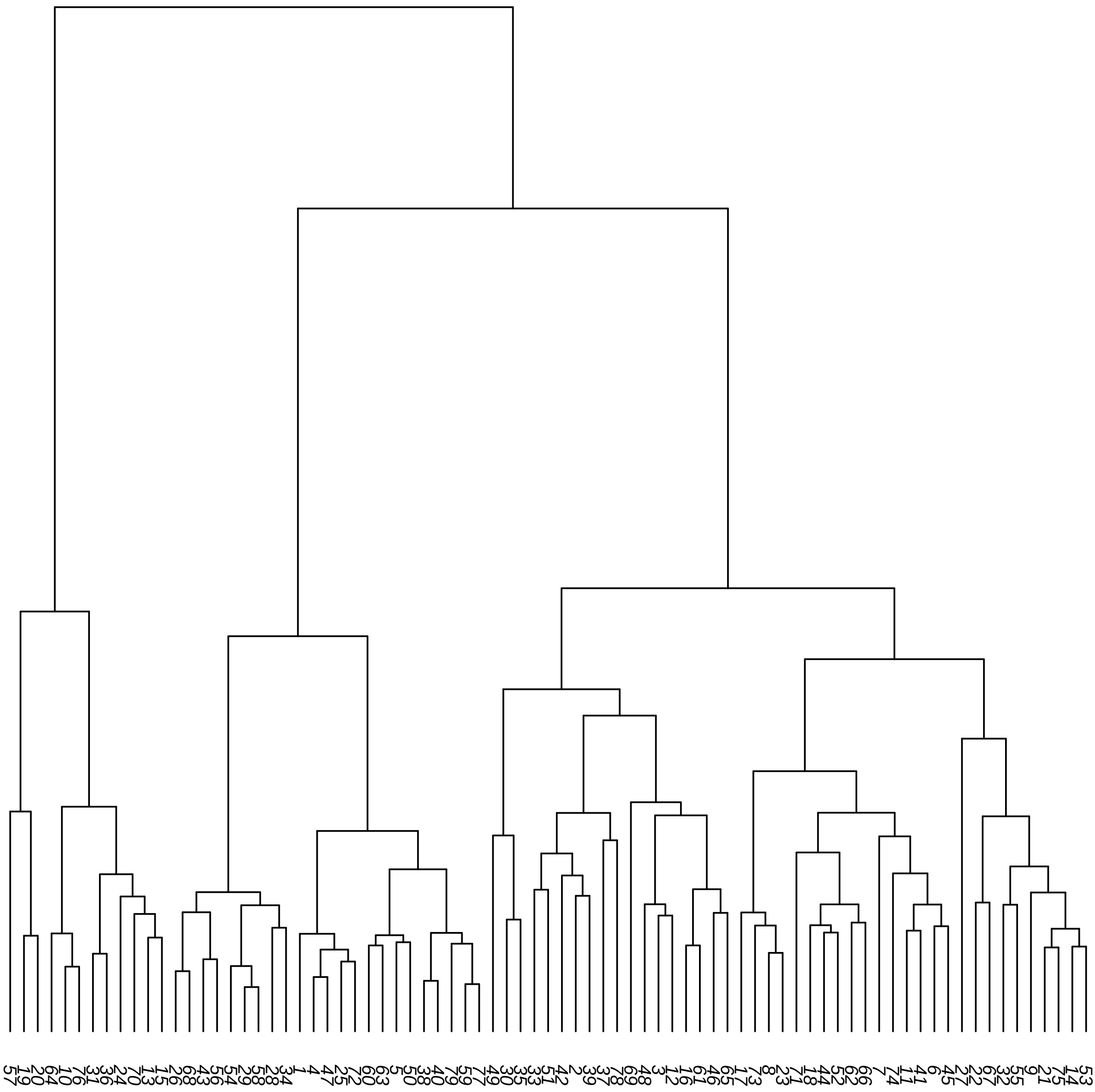

AUS Area 4 Unrooted Cluster dendrogram

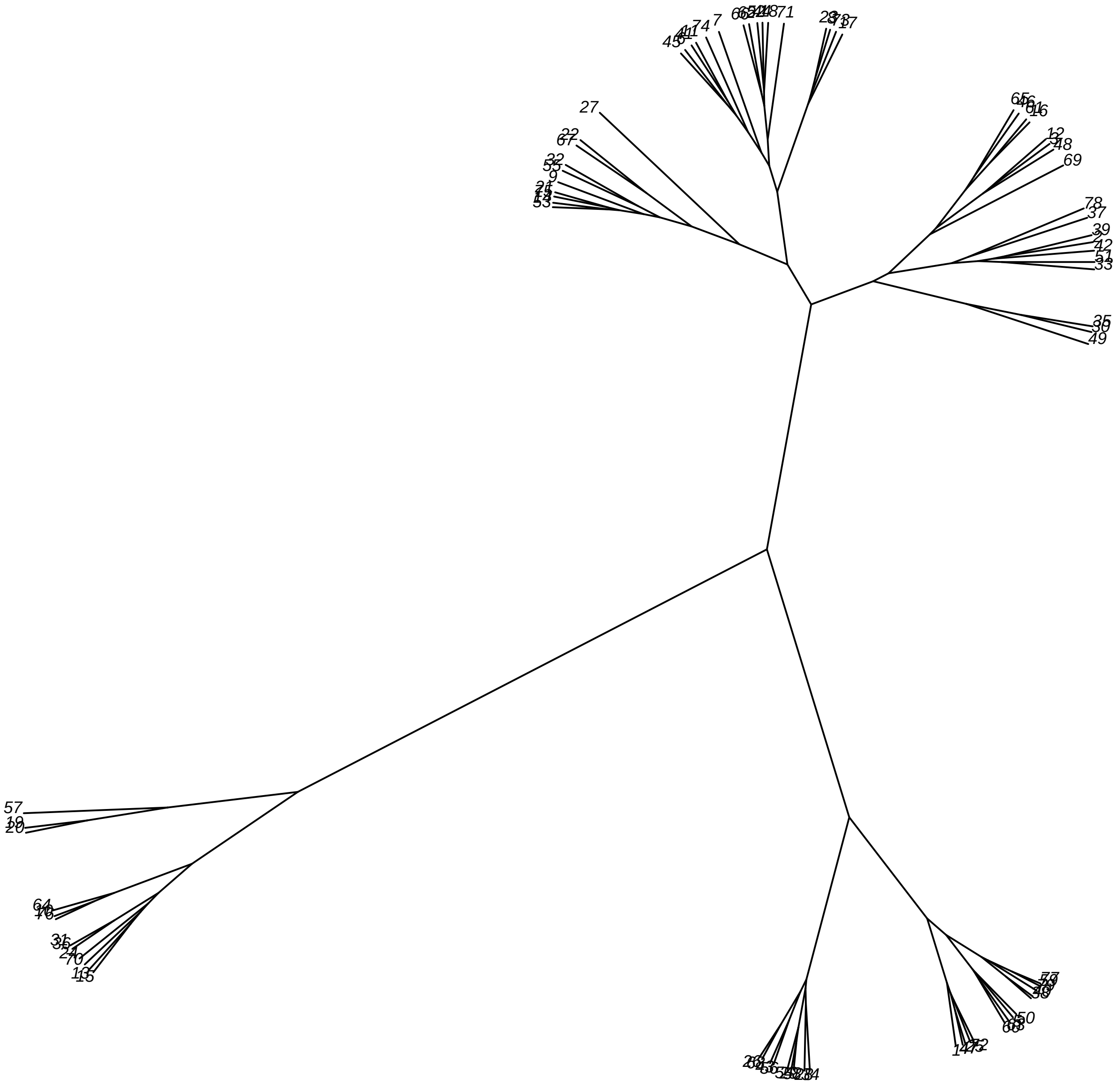

AUS Area 4 Fan Cluster dendrogram

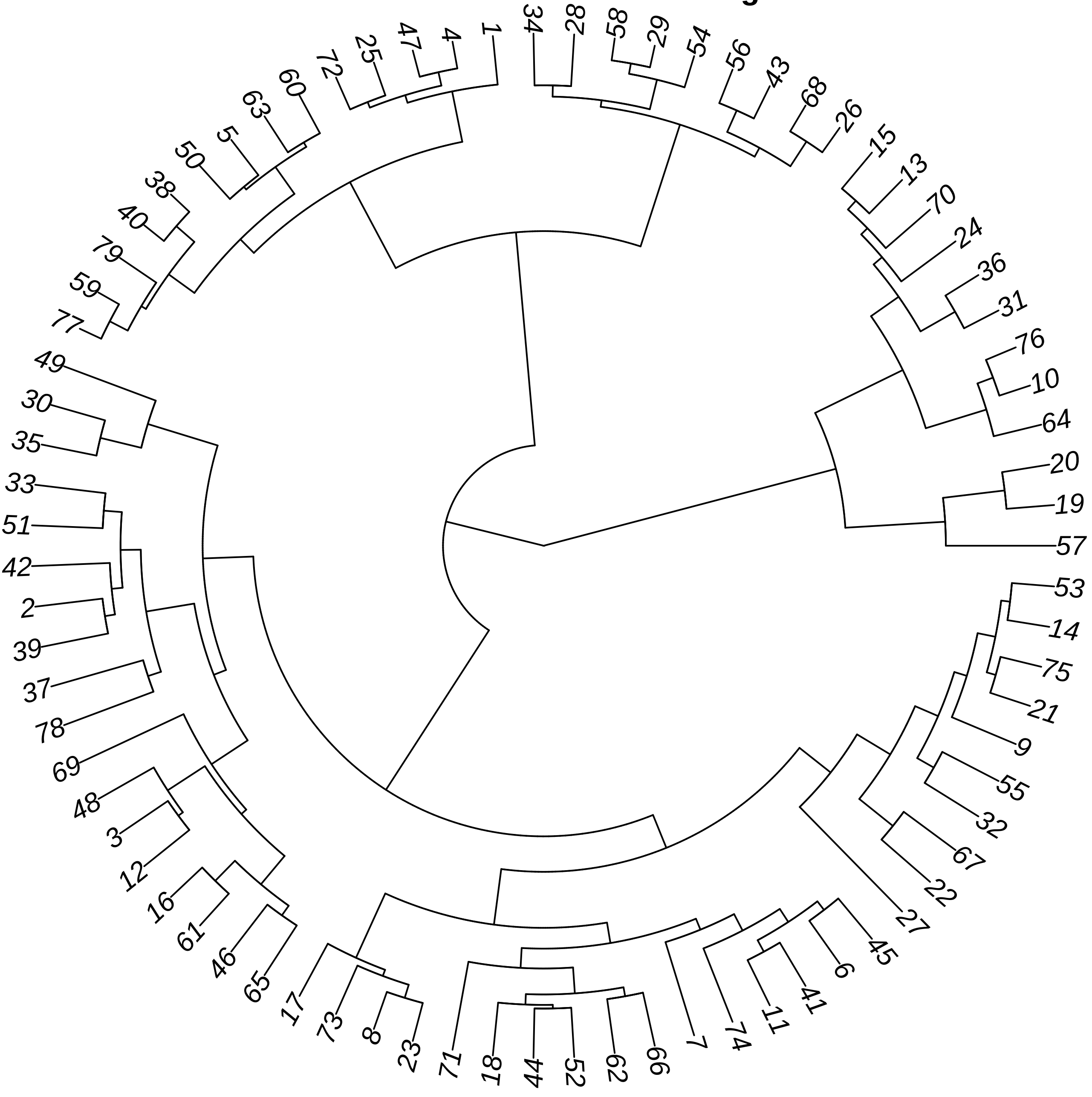

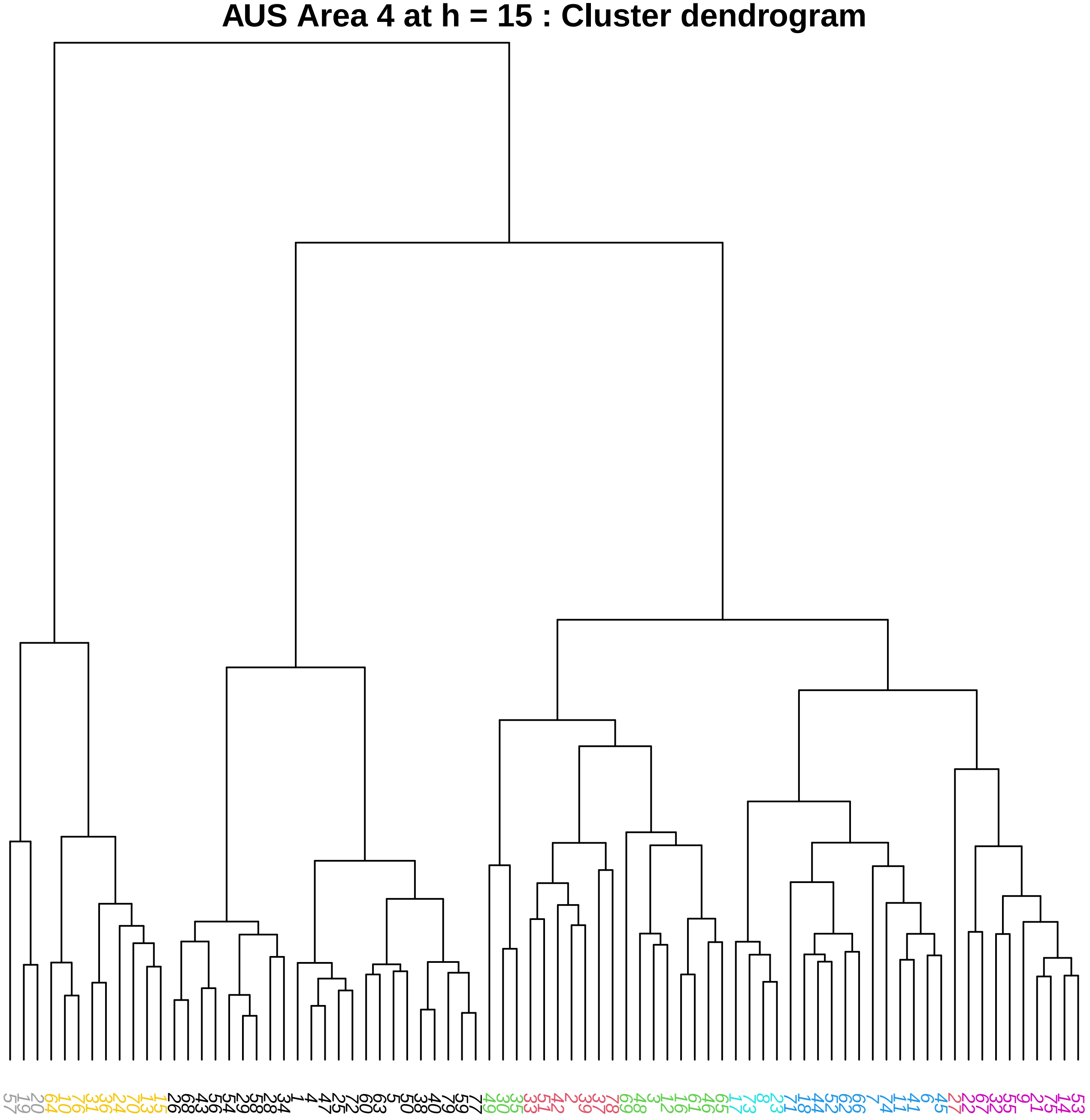
AUS Area 4 at h = 15 : Cluster dendrogram
57 19 20 64 10 76 31 36 24 70 13 15 26 68 43 56 54 29 58 28 34 1 4 47 25 72 60 63 5 50 38 40 79 59 77 49 30 35 33 51 42 2 39 37 78 69 48 3 12 16 61 46 65 17 73 8 23 71 18 44 52 62 66 7 74 11 41 6 45 27 22 67 32 55 9 21 75 14 53

AUS Area 4 at h = 15 : Coloured Unrooted Cluster dendrogram

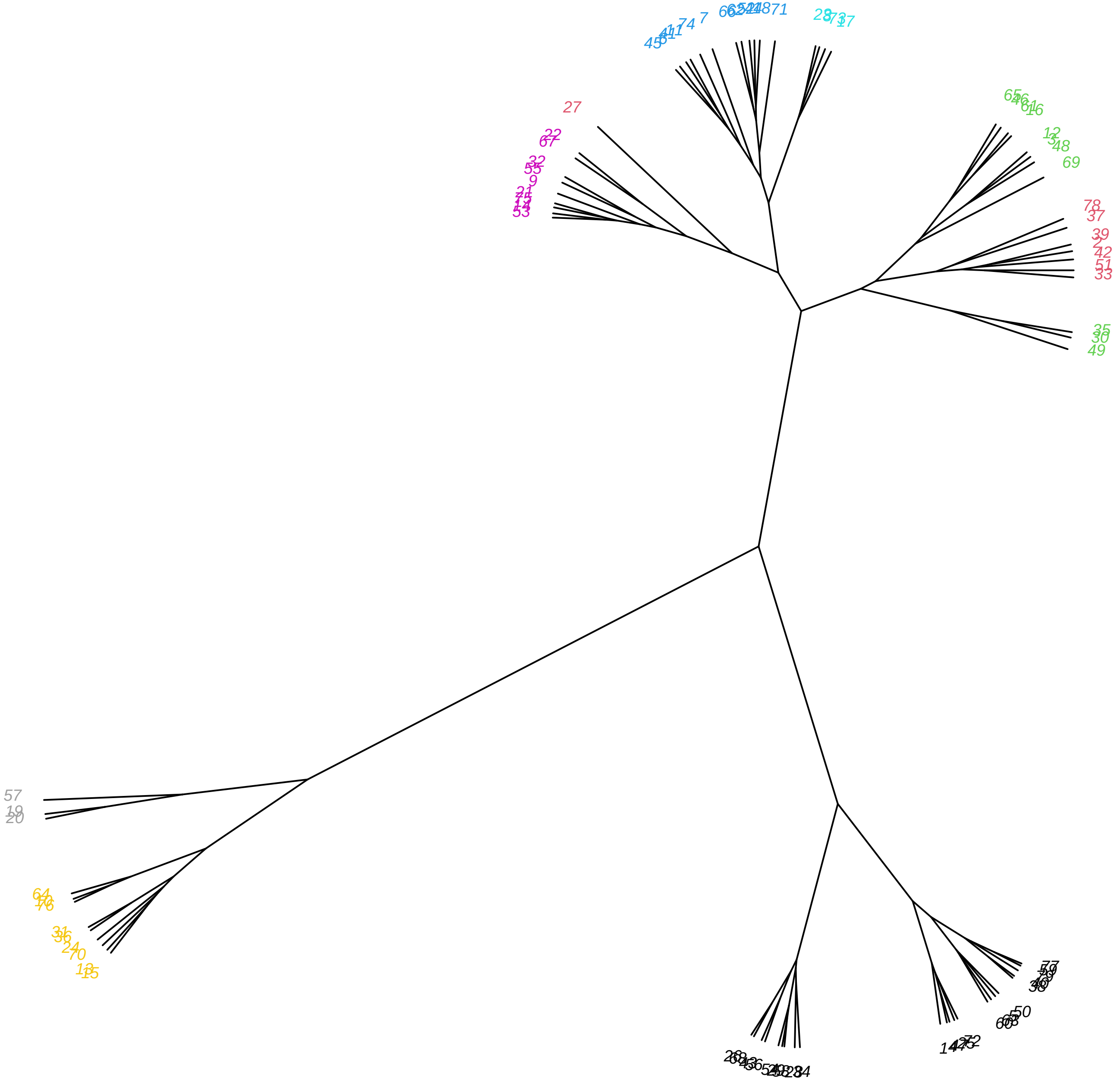

AUS Area 4 at h = 15 : Coloured Fan Cluster dendrogram

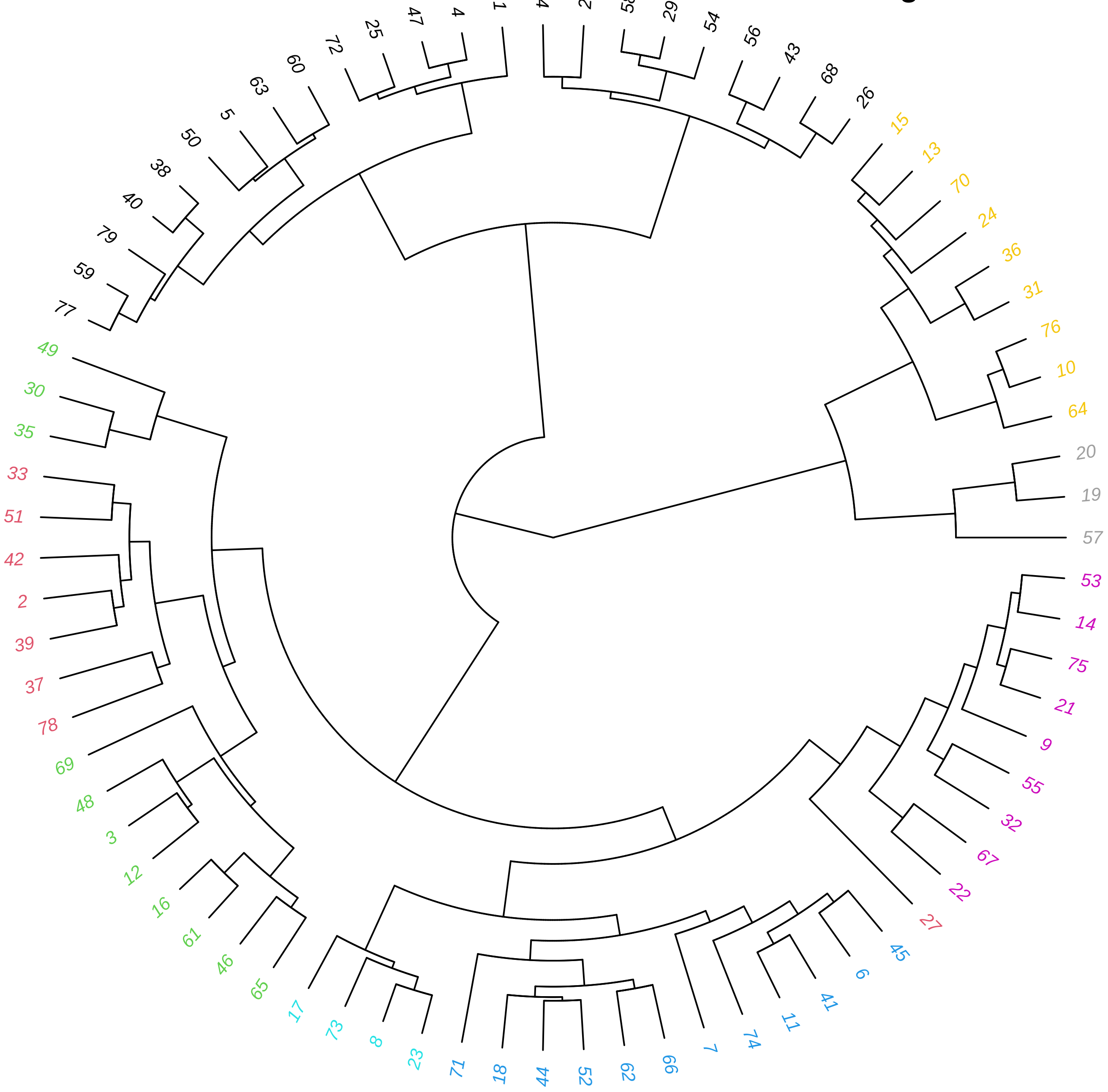

AUS Area 5 with cluster c(20, 20, 50, 50, 50, 20, 50) Cluster dendrogram

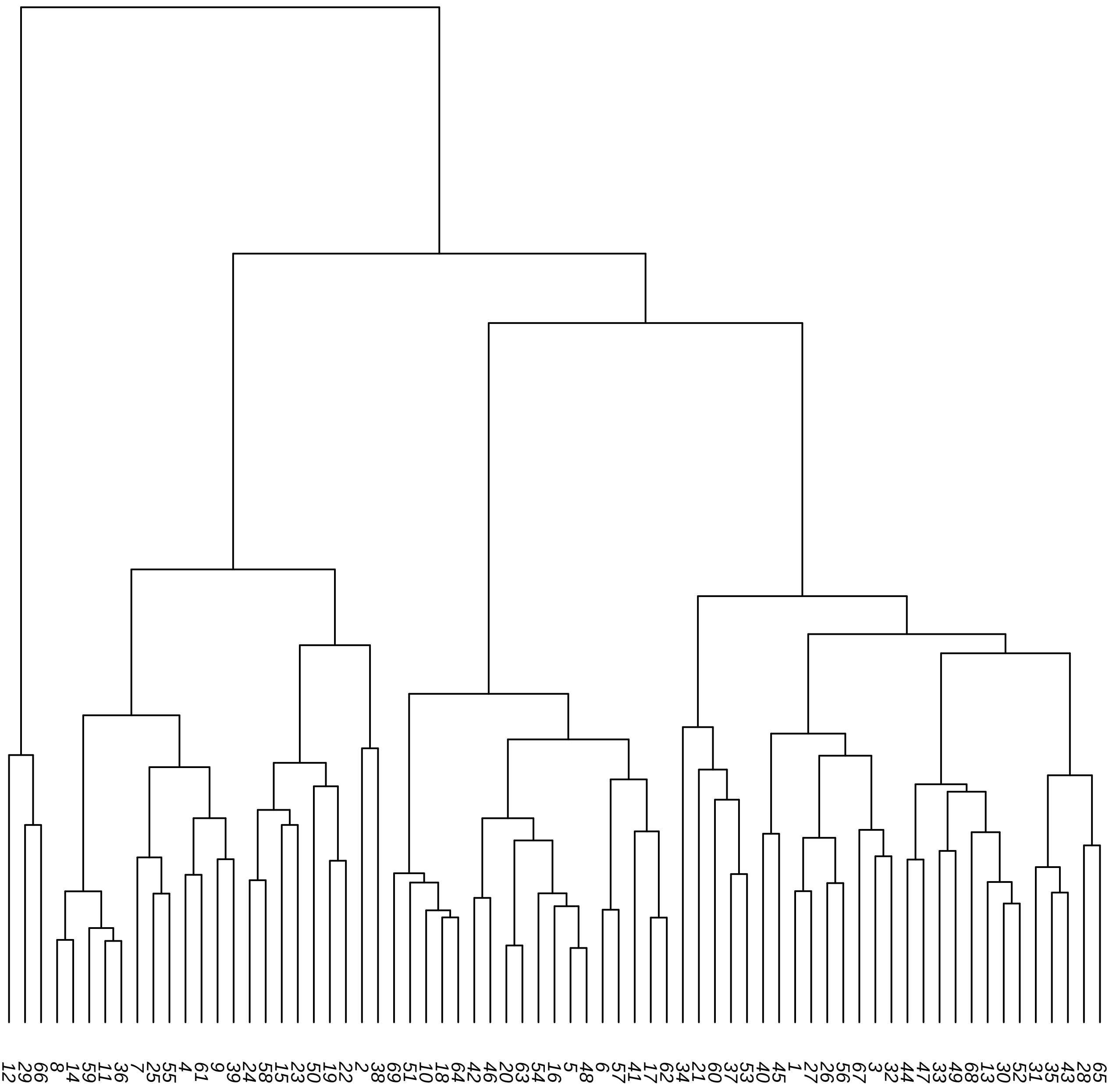

AUS Area 5 Unrooted Cluster dendrogram

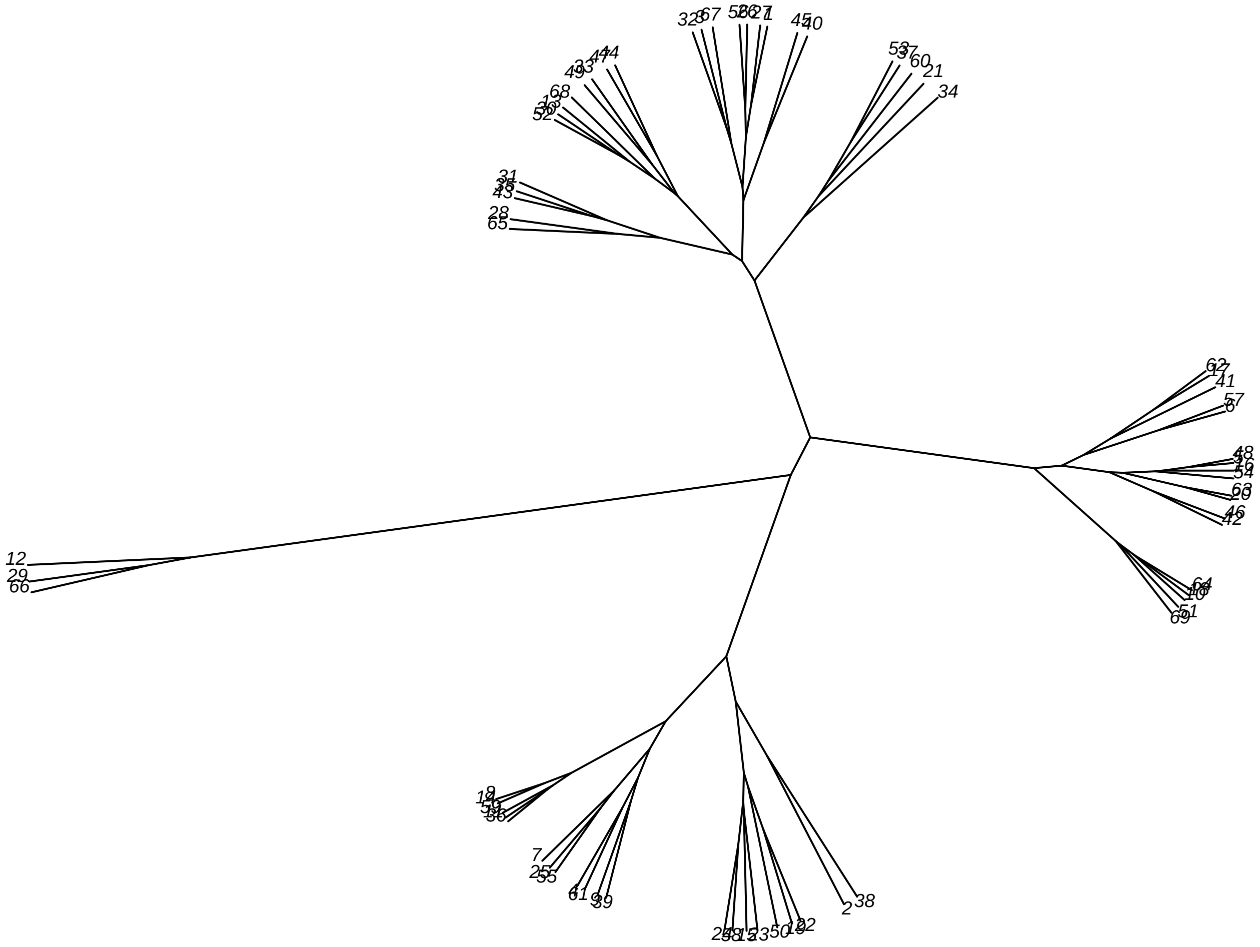

AUS Area 5 Fan Cluster dendrogram

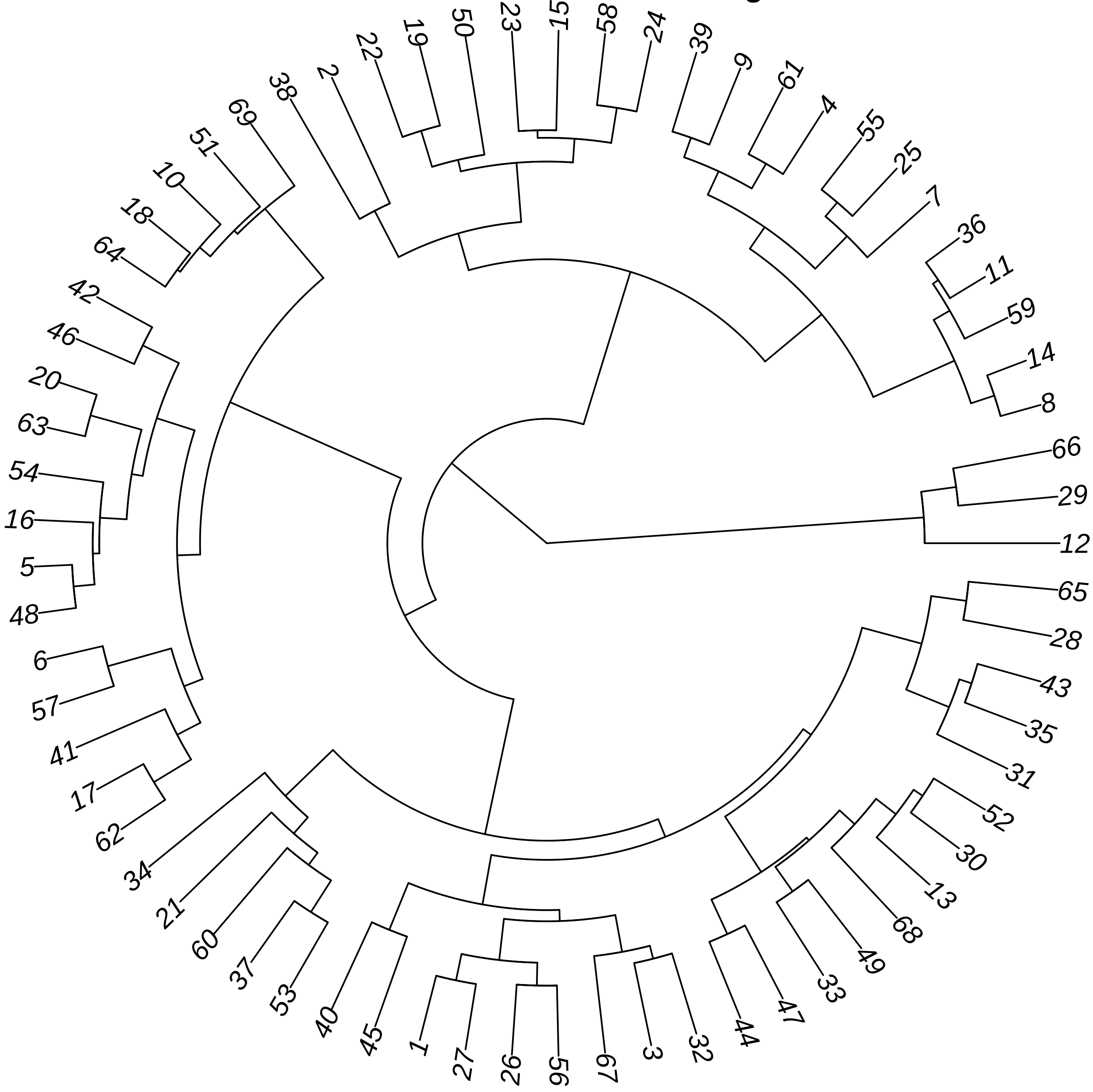

AUS Area 5 at h = 15 : Cluster dendrogram

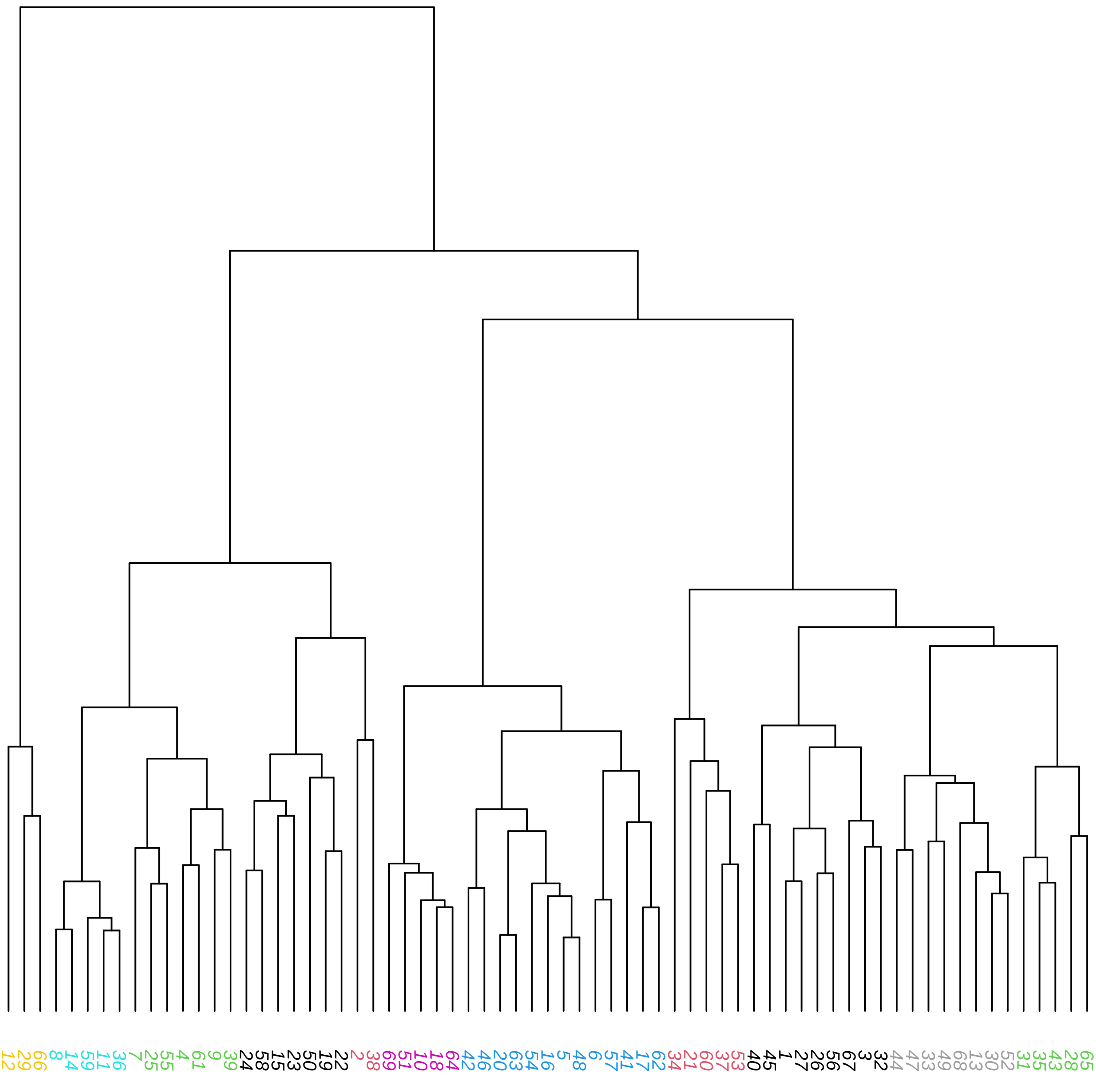

AUS Area 5 at h = 15 : Coloured Unrooted Cluster dendrogram

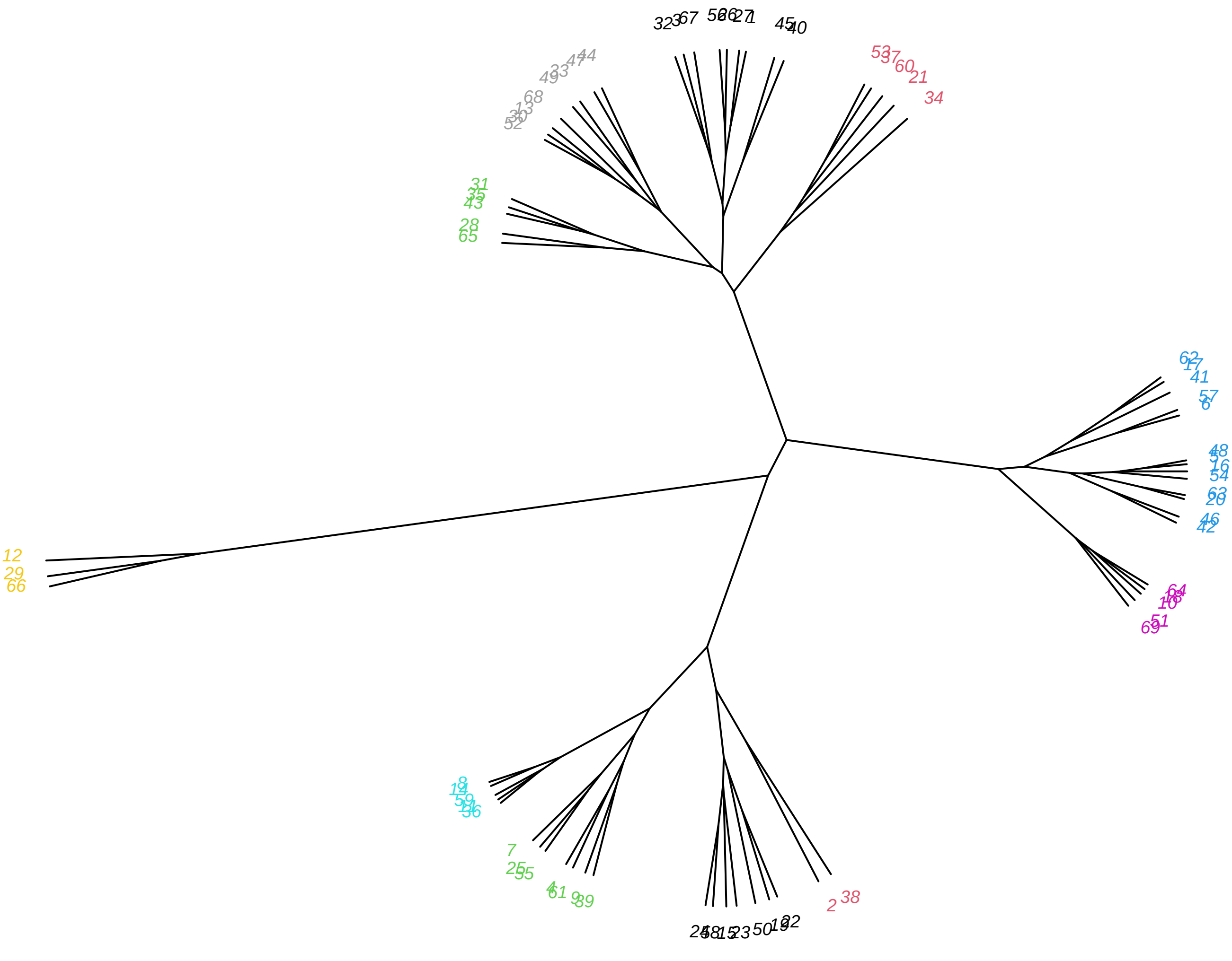

AUS Area 5 at h = 15 : Coloured Fan Cluster dendrogram

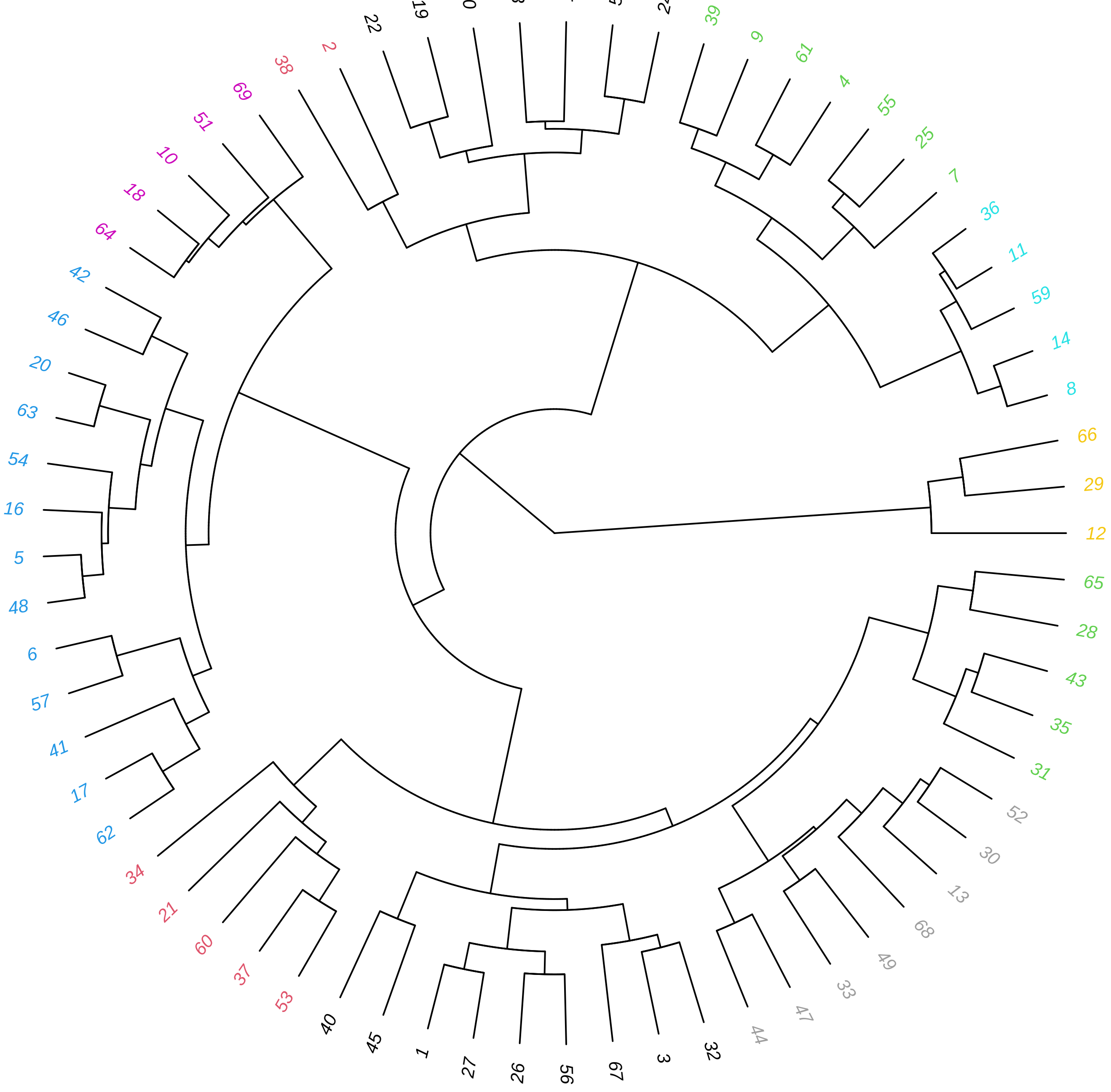

AUS Area 6 with cluster c(20, 20, 50, 50, 50, 20, 50) Cluster dendrogram

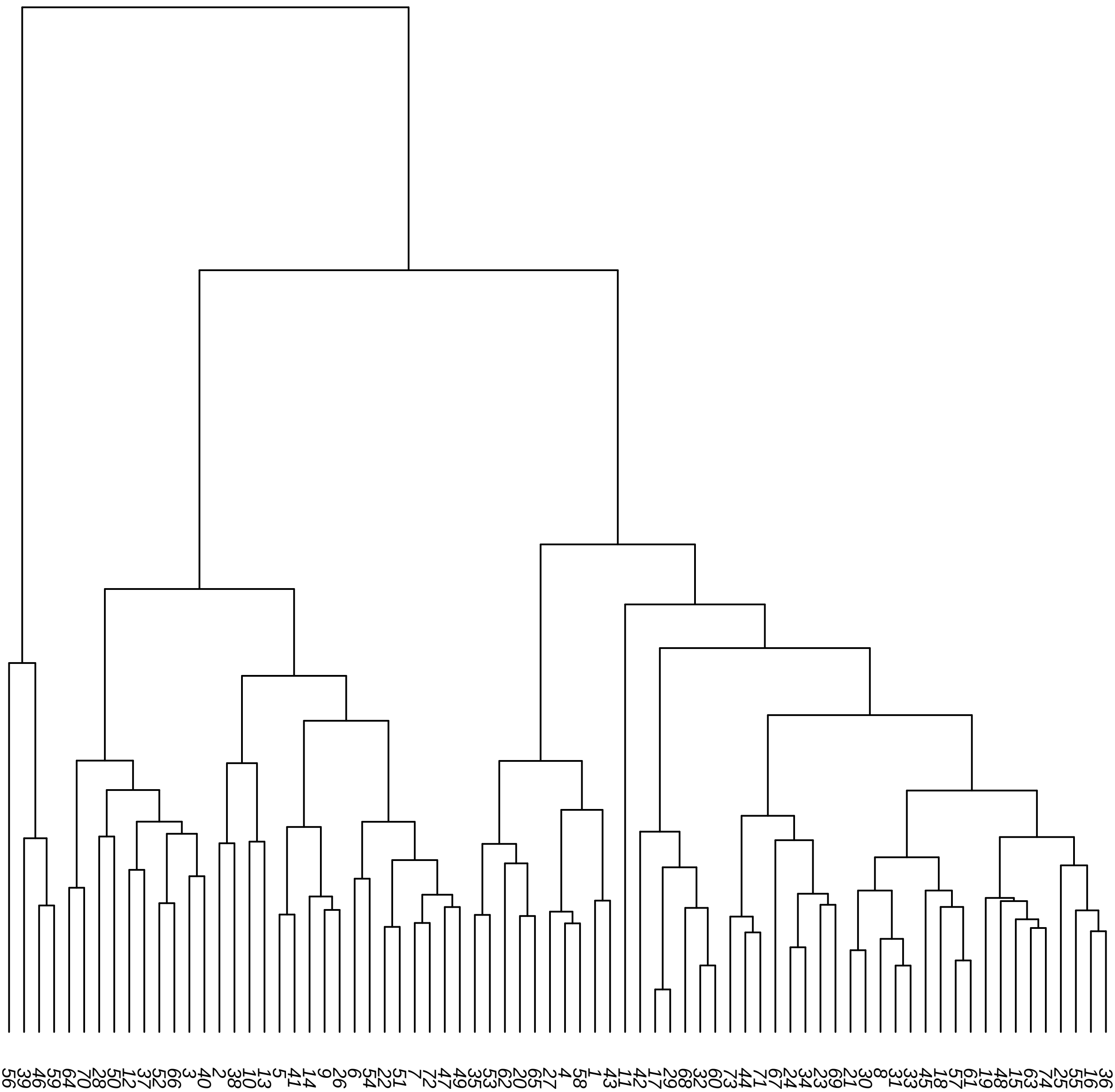

AUS Area 6 Unrooted Cluster dendrogram

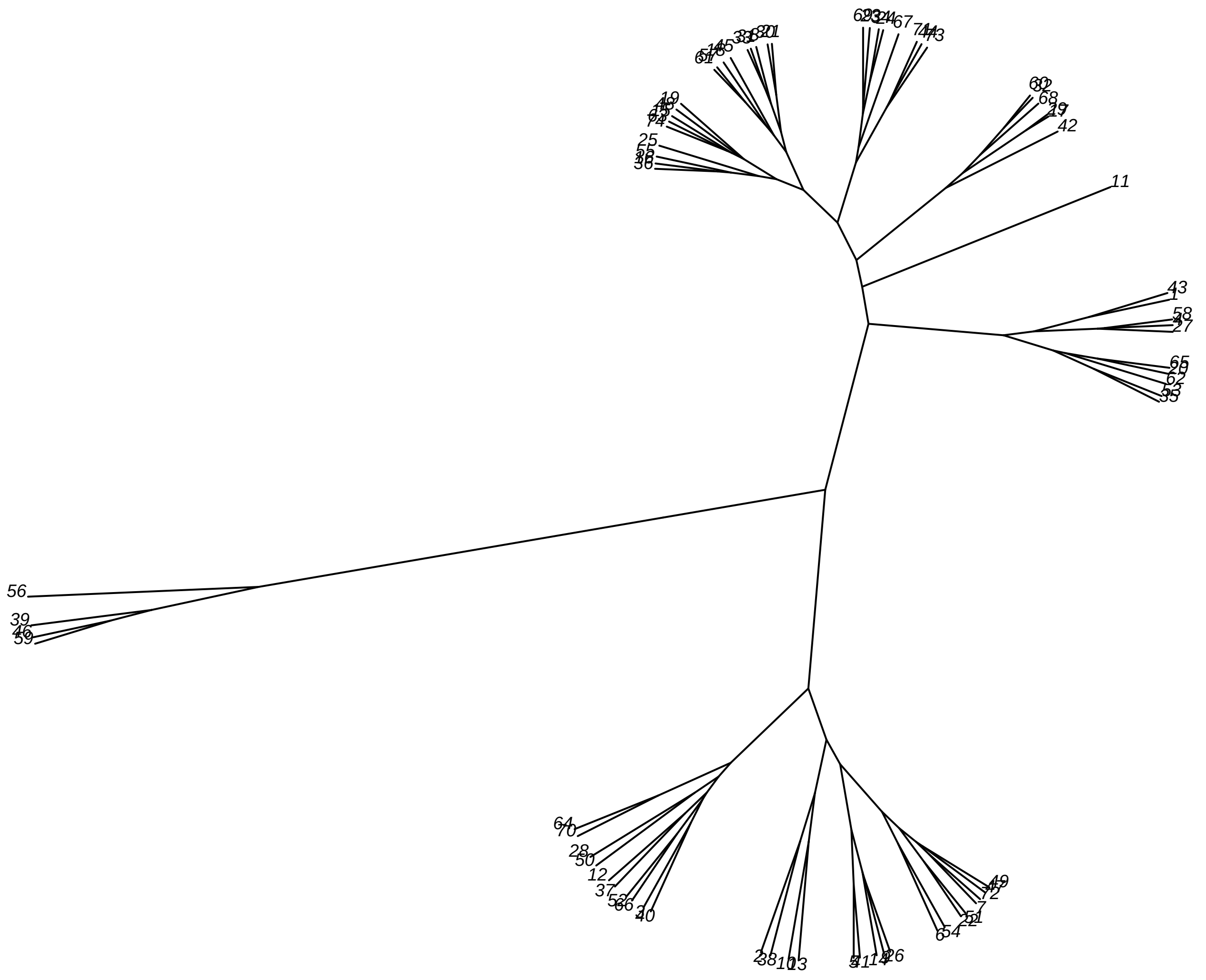

AUS Area 6 Fan Cluster dendrogram

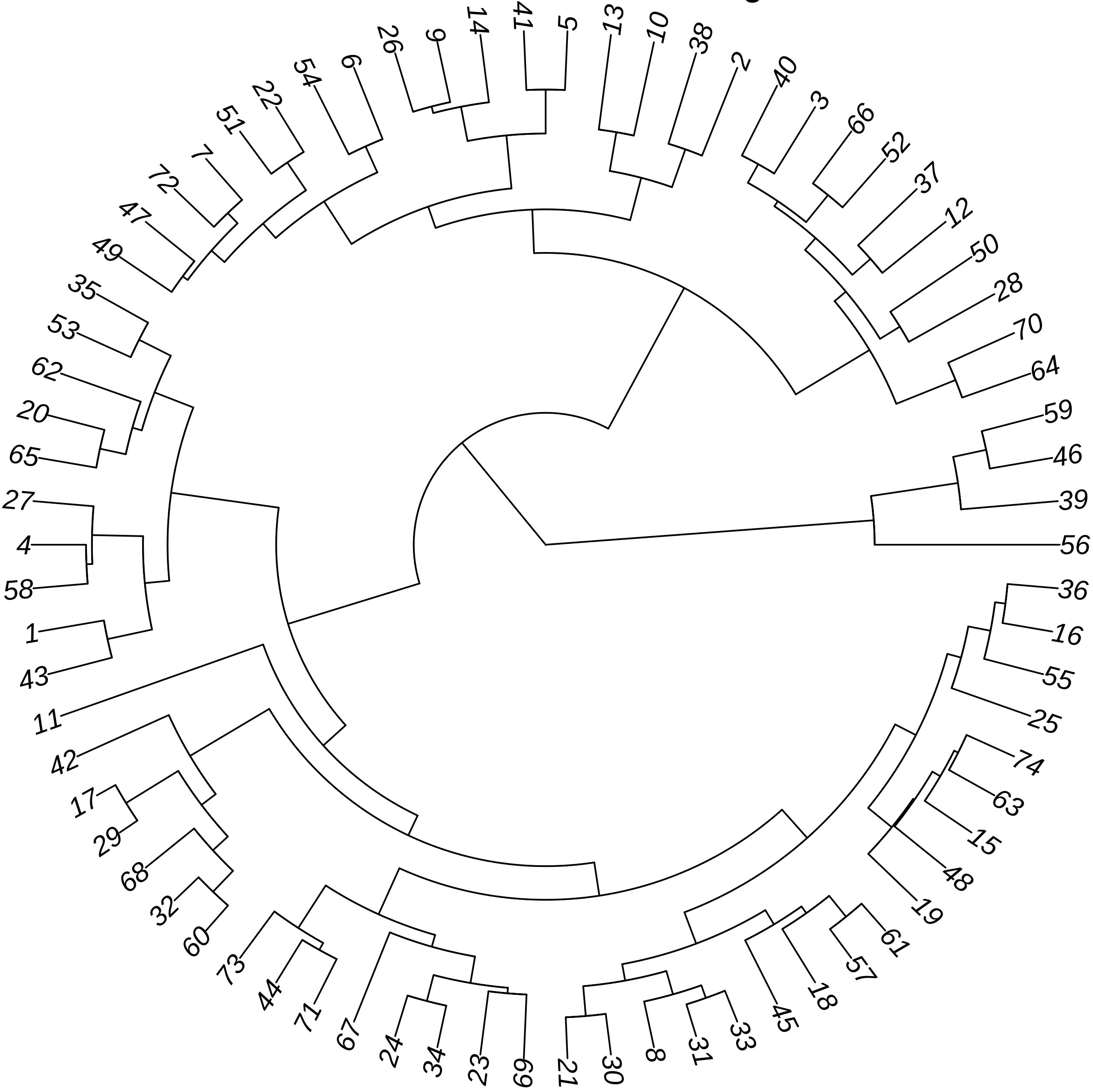

AUS Area 6 at h = 15 : Cluster dendrogram

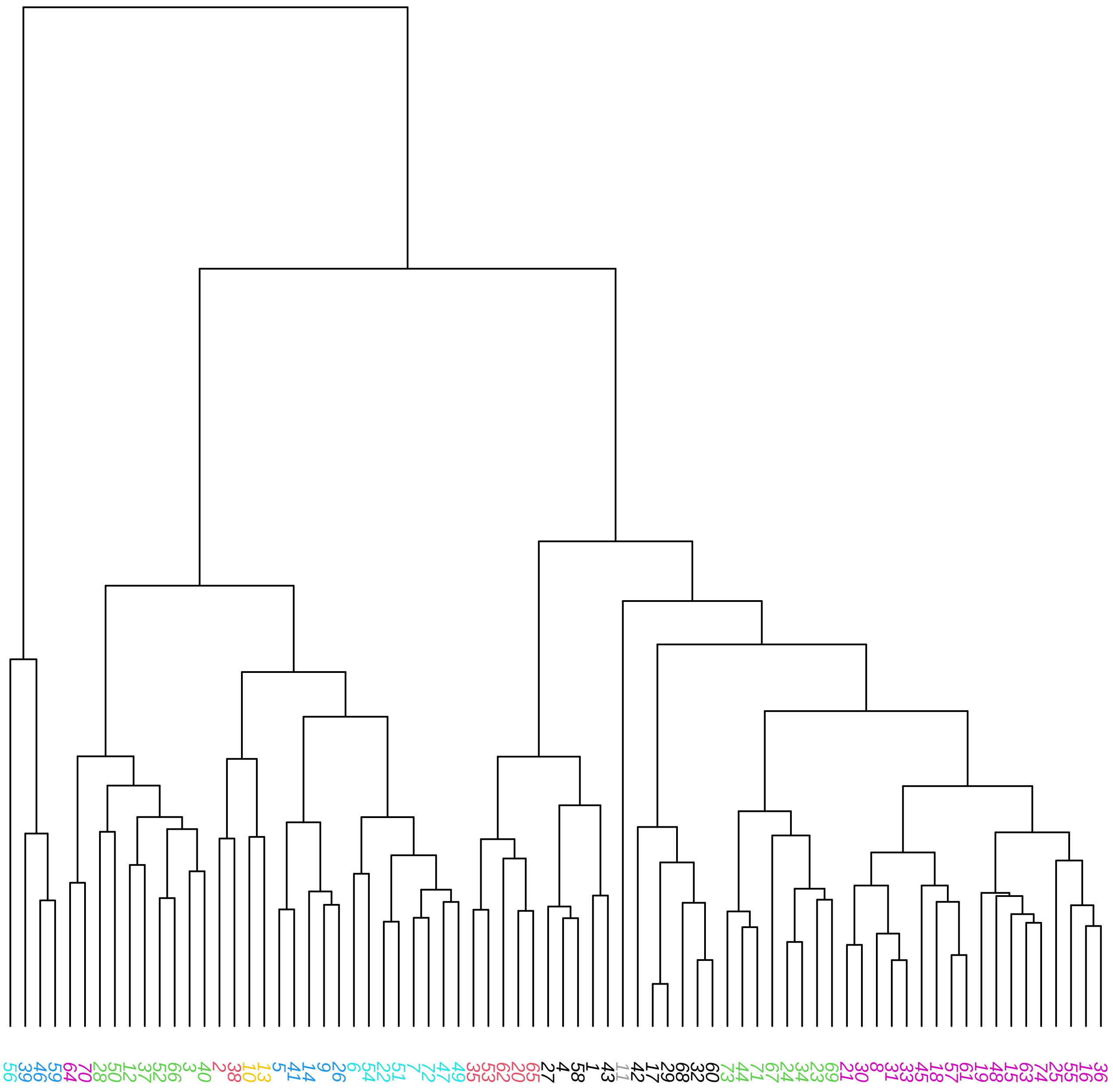

AUS Area 6 at h = 15 : Coloured Unrooted Cluster dendrogram

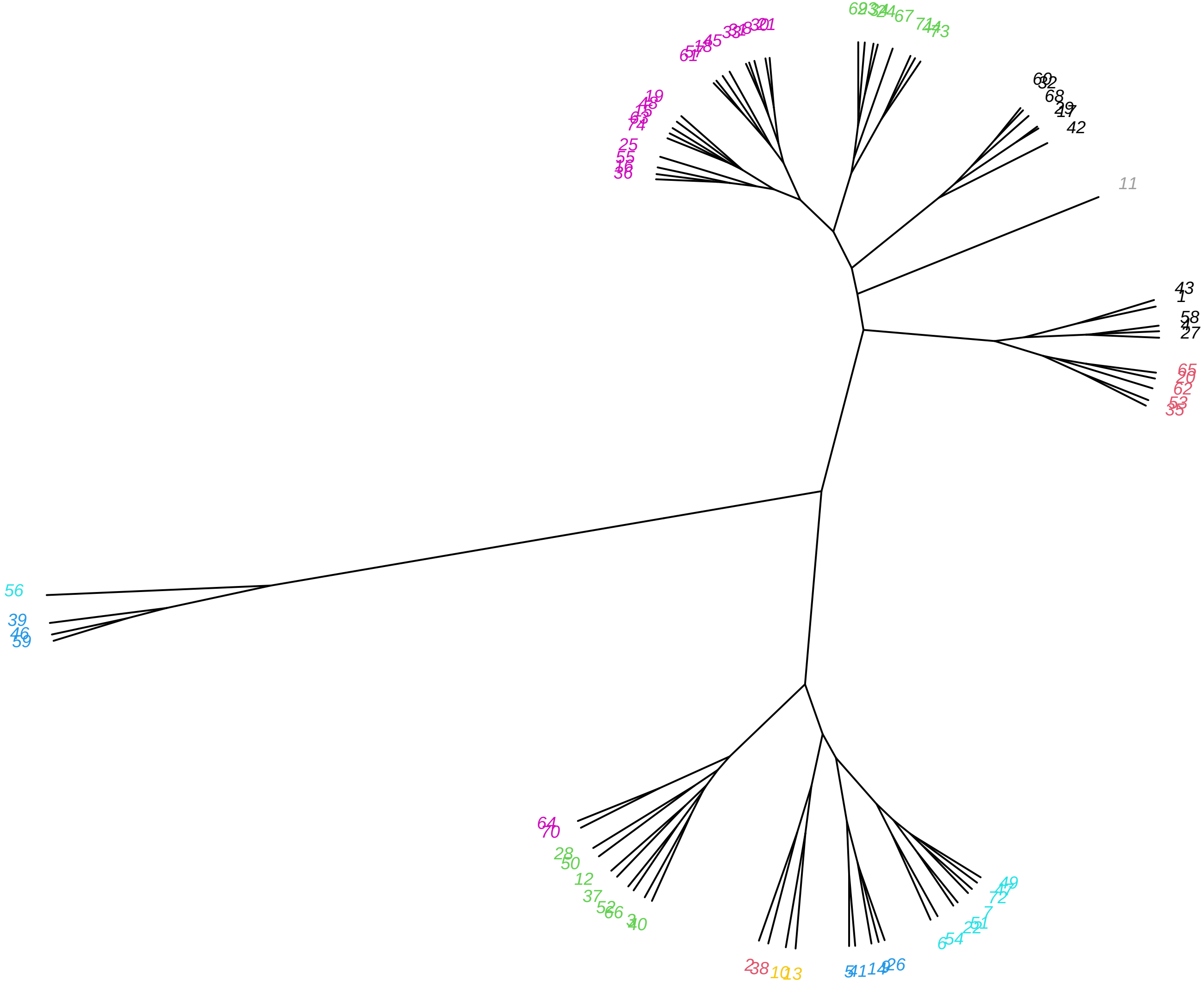

AUS Area 6 at h = 15 : Coloured Fan Cluster dendrogram

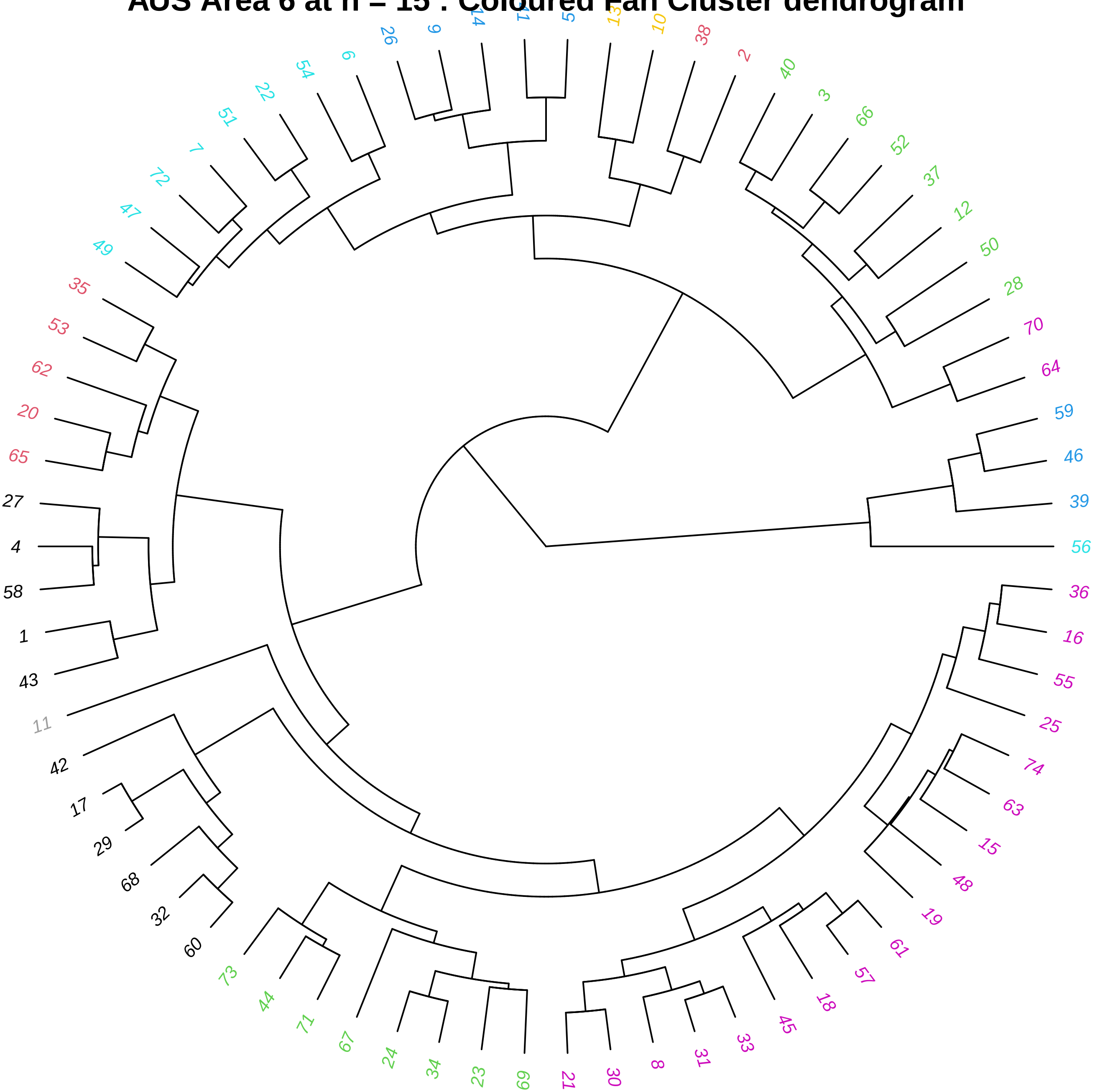

AUS Area 7 with cluster c(20, 20, 50, 50, 50, 20, 50) Cluster dendrogram

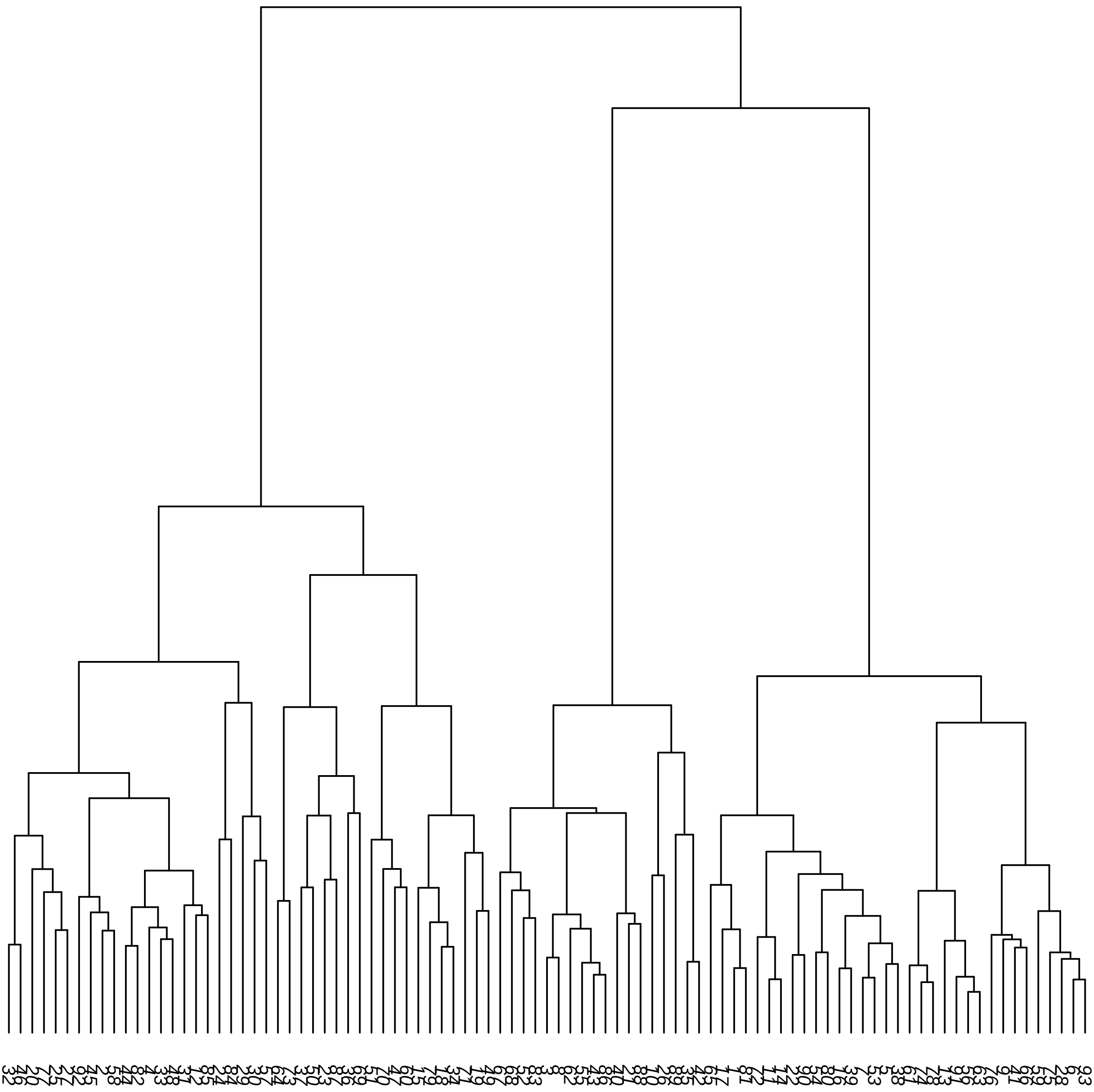

AUS Area 7 Unrooted Cluster dendrogram

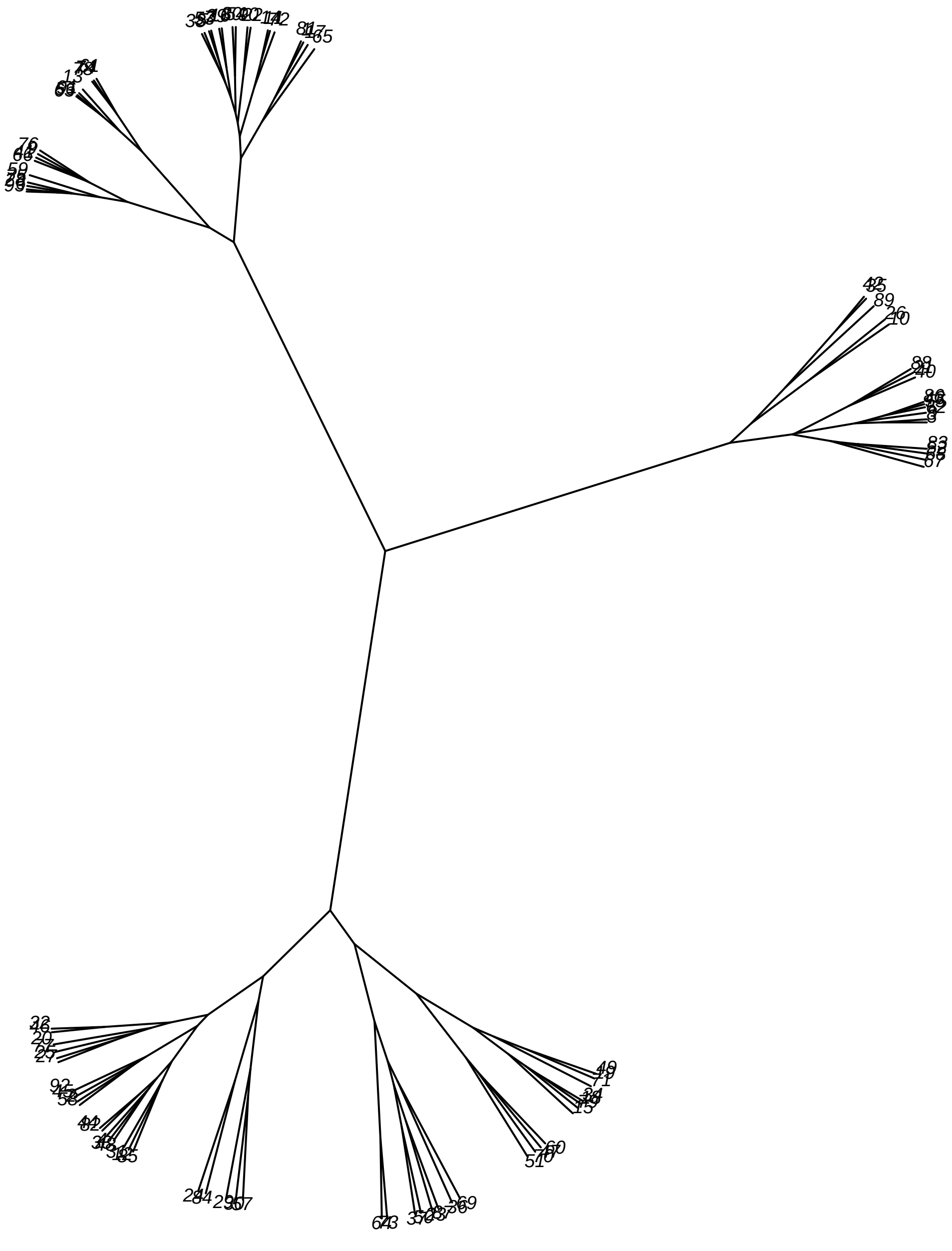

AUS Area 7 Fan Cluster dendrogram

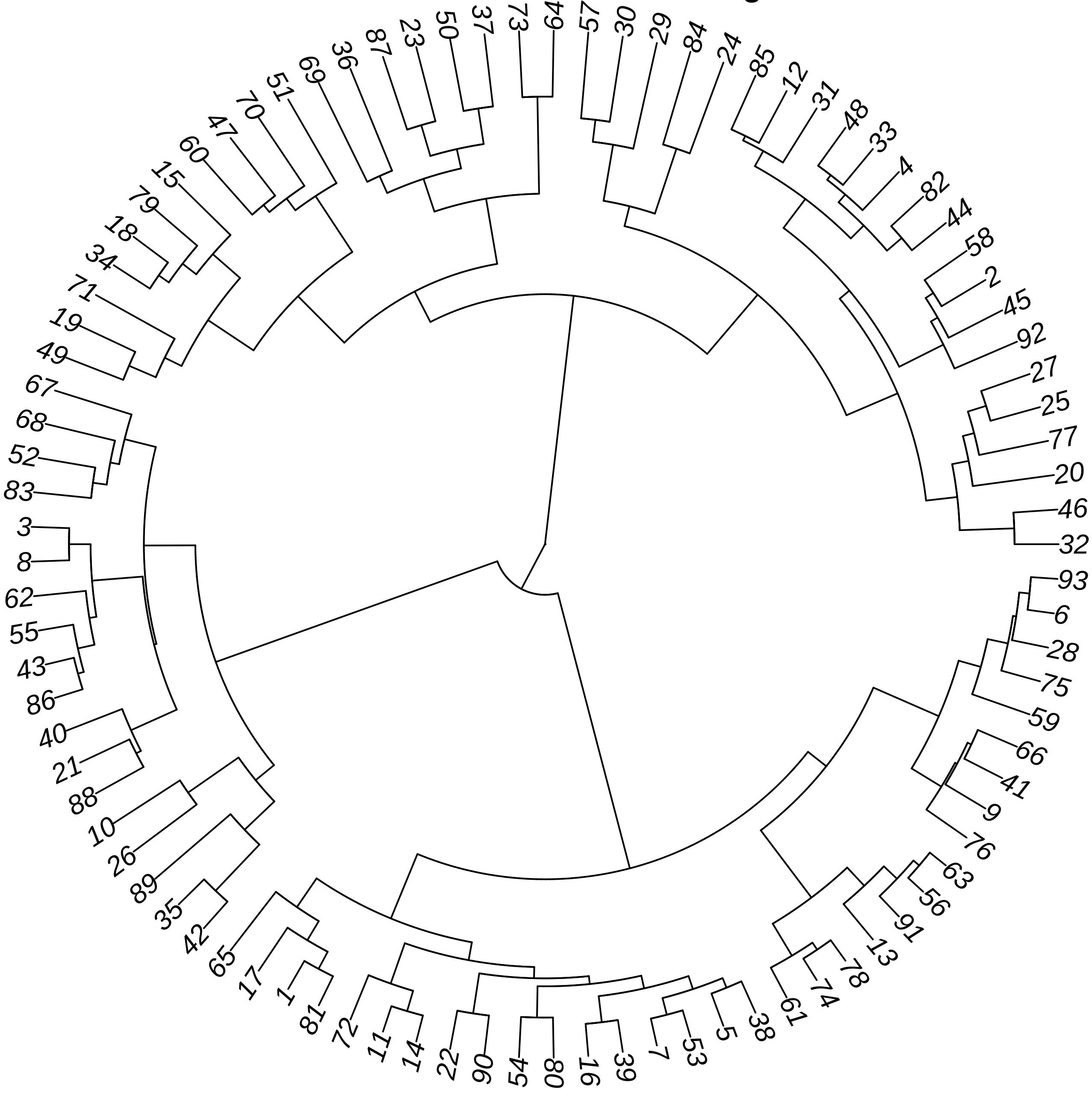

AUS Area 7 at h = 15 : Cluster dendrogram

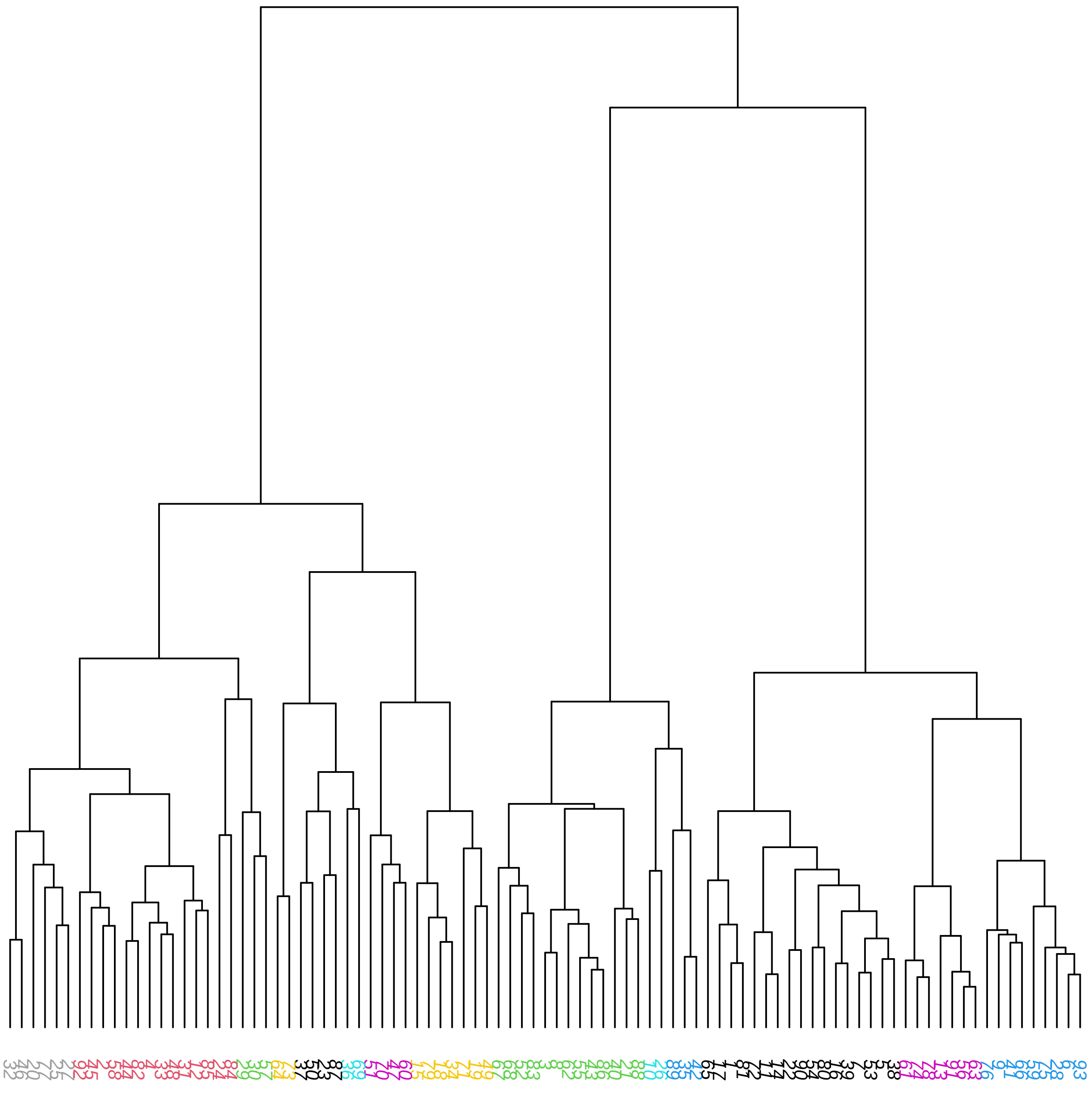

AUS Area 7 at h = 15 : Coloured Unrooted Cluster dendrogram

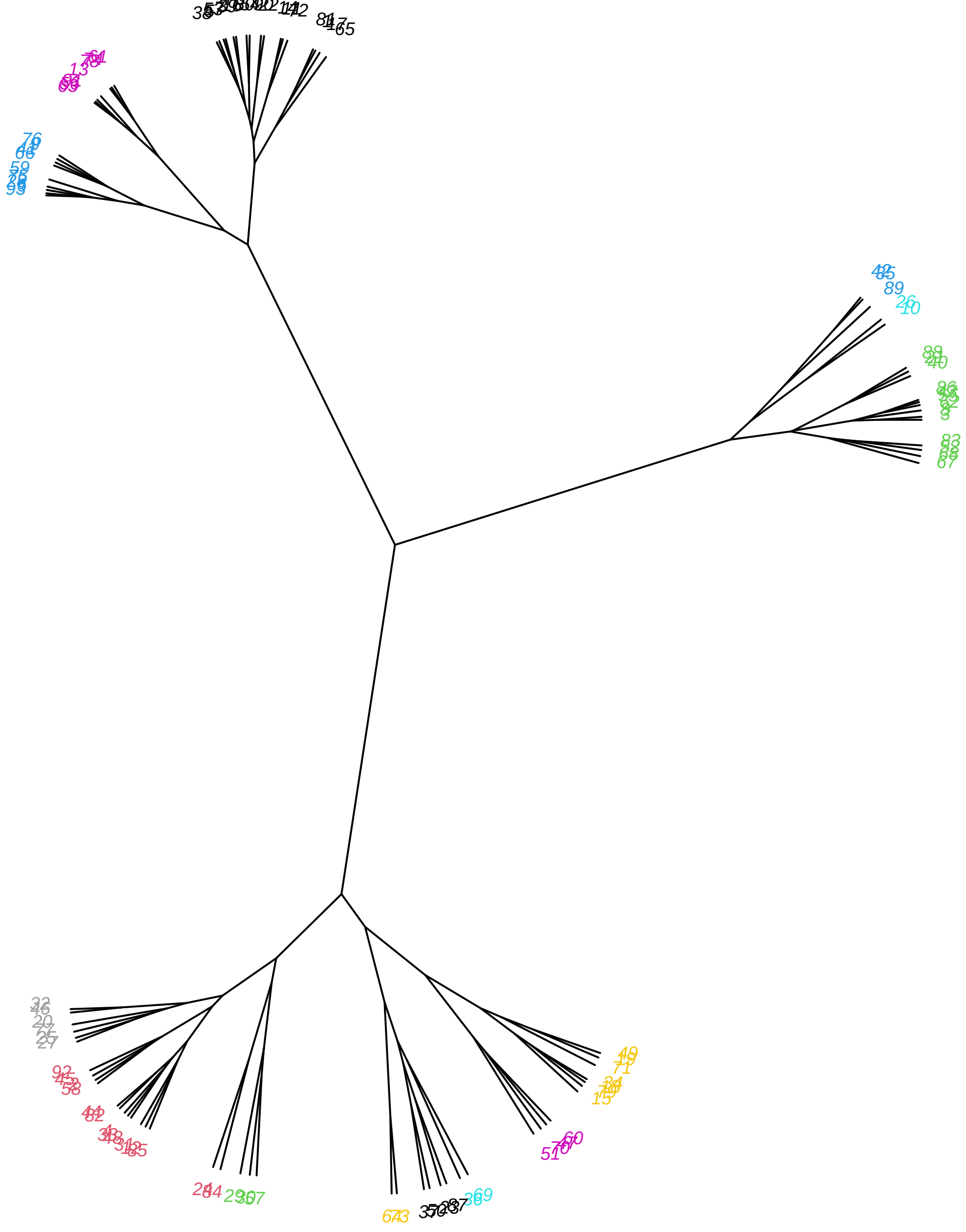

AUS Area 7 at h = 15 : Coloured Fan Cluster dendrogram

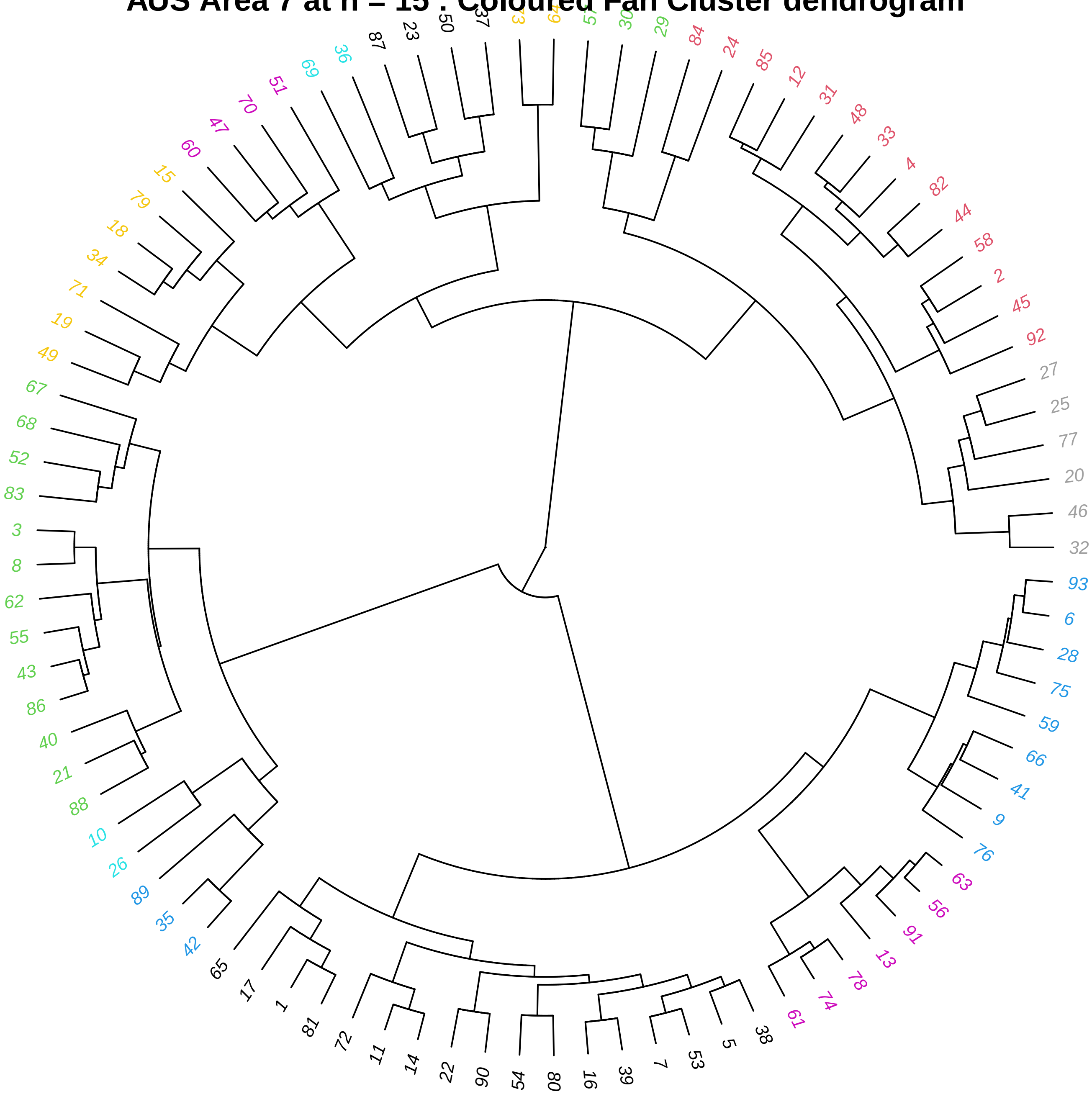

Supplement: S3 Appendix — (PDF) [file pone.0272848.s003.pdf]
