# Supplementary material for: Classifying ball trajectories in invasion sports using dynamic time warping: A basketball case study
Source: PLoS One. 2022 Oct 20;17(10):e0272848. doi: 10.1371/journal.pone.0272848 (PMC9584368; doi:10.1371/journal.pone.0272848)

AUS Area 1 Cluster 1 : SelectTrajectories

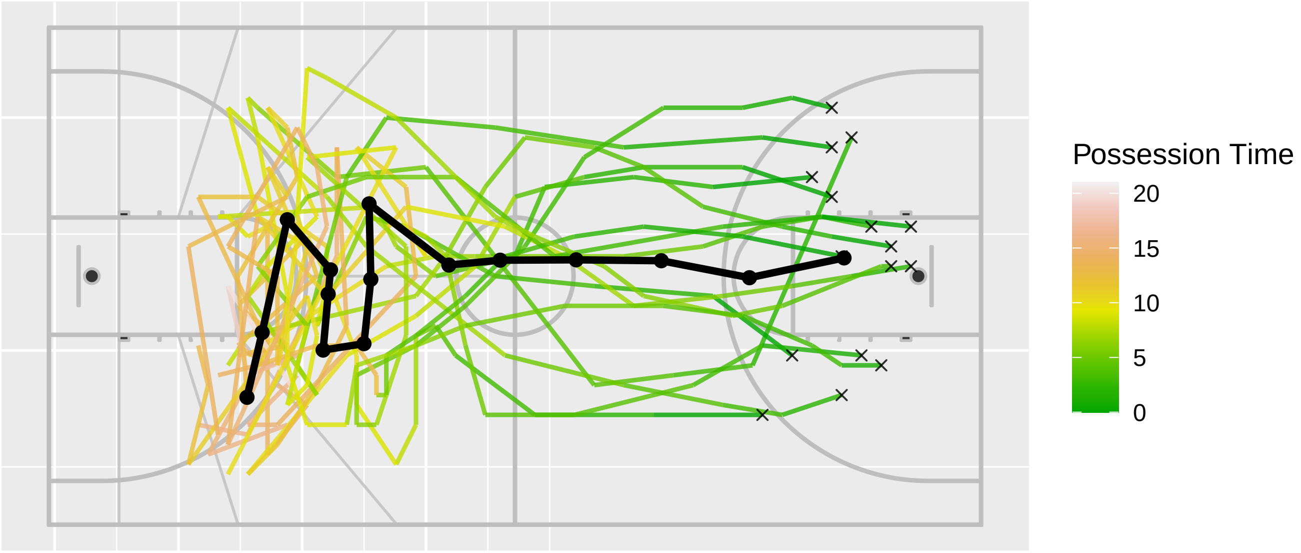

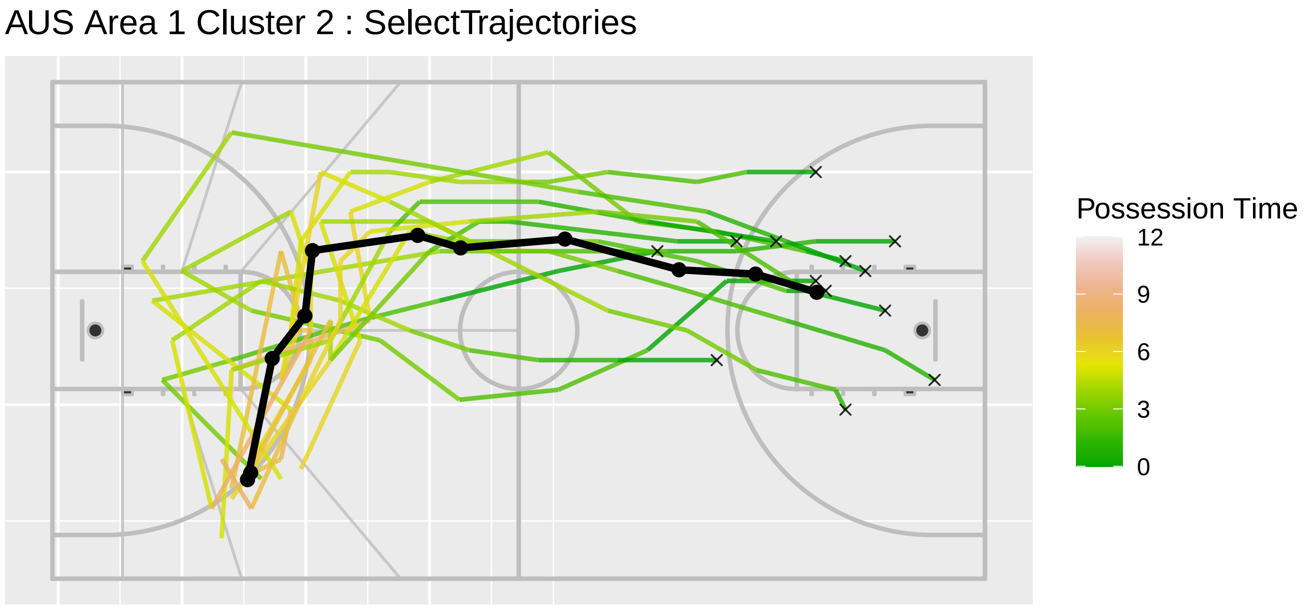

AUS Area 1 Cluster 2 : SelectTrajectories
Possession Time
12
9
6
3
0

AUS Area 1 Cluster 3 : SelectTrajectories

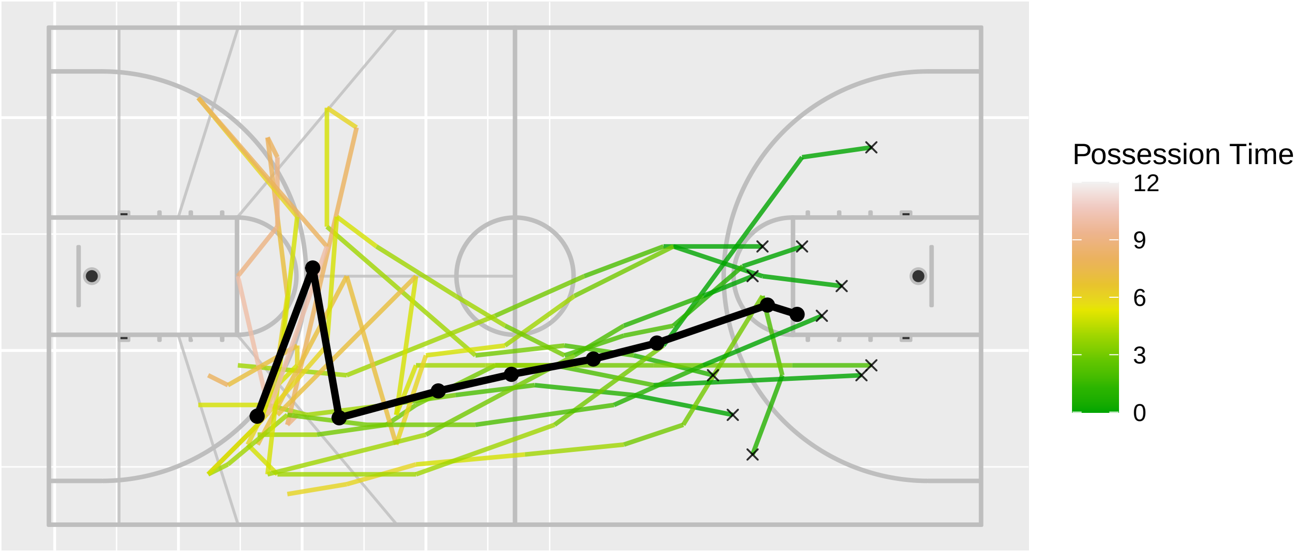

AUS Area 1 Cluster 4 : SelectTrajectories

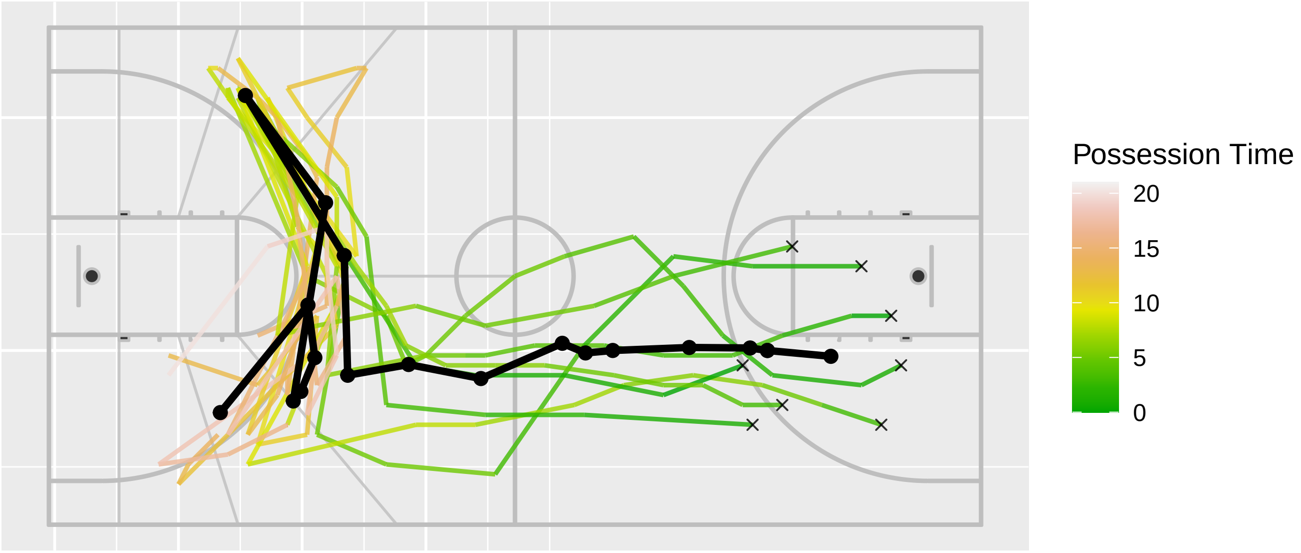

AUS Area 1 Cluster 5 : SelectTrajectories

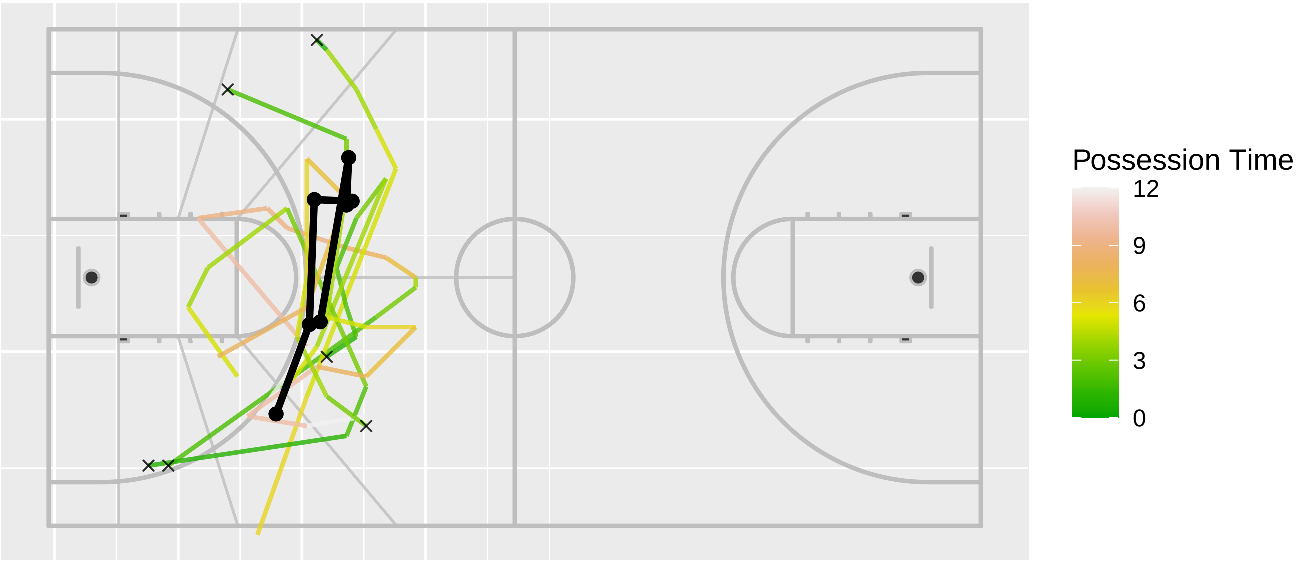

AUS Area 1 Cluster 6 : SelectTrajectories

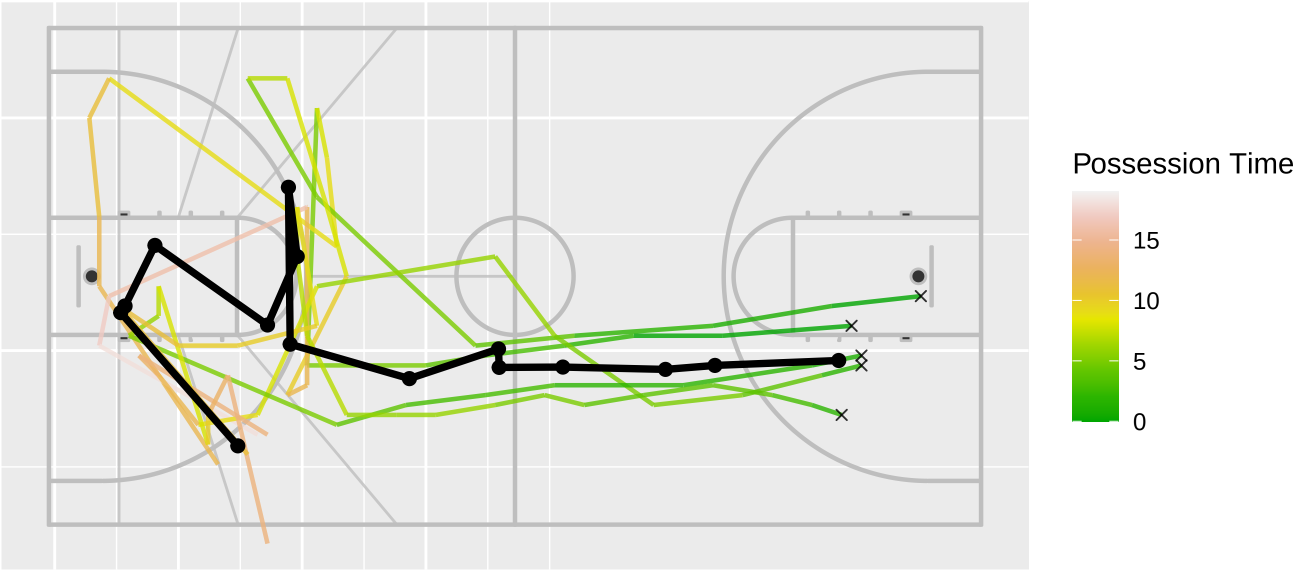

AUS Area 1 Cluster 7 : SelectTrajectories

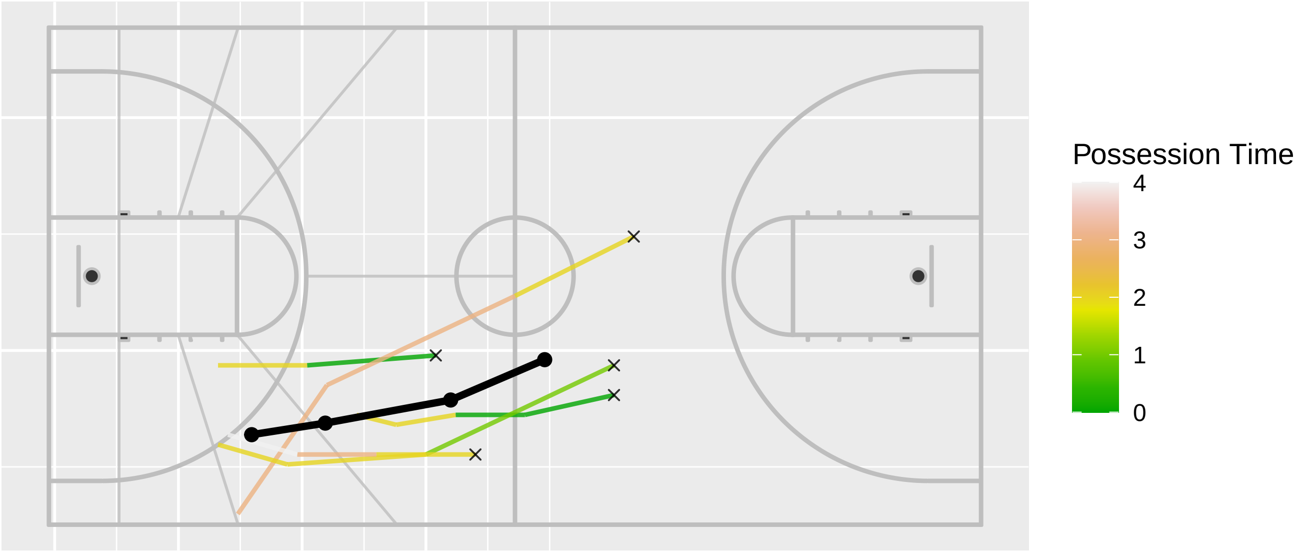

AUS Area 1 Cluster 8 : SelectTrajectories

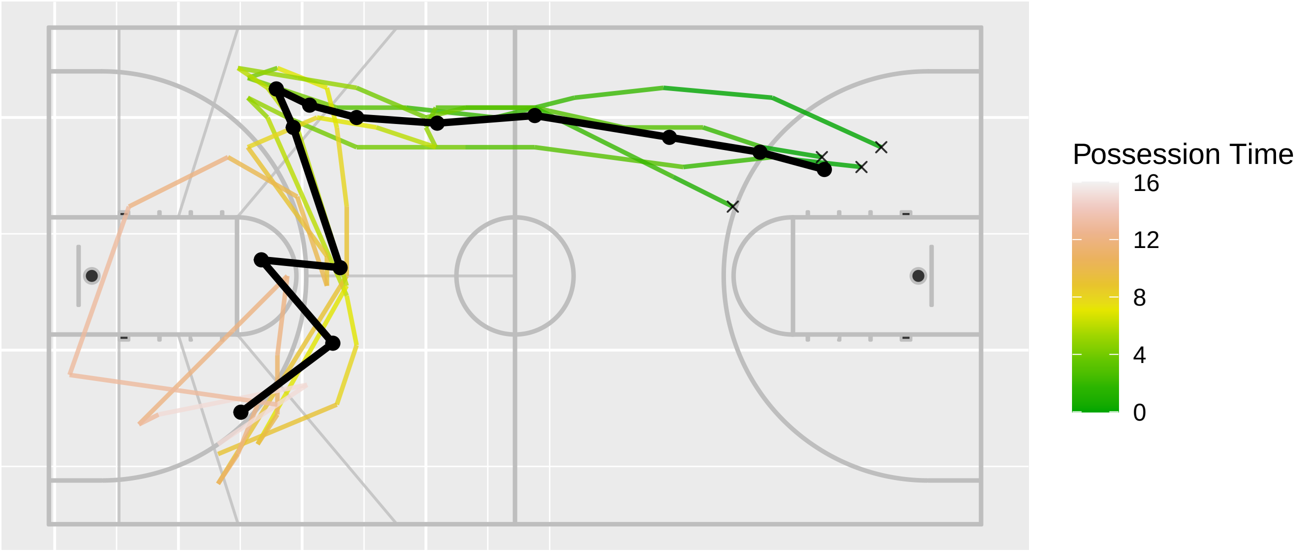

AUS Area 1 Cluster 9 : SelectTrajectories

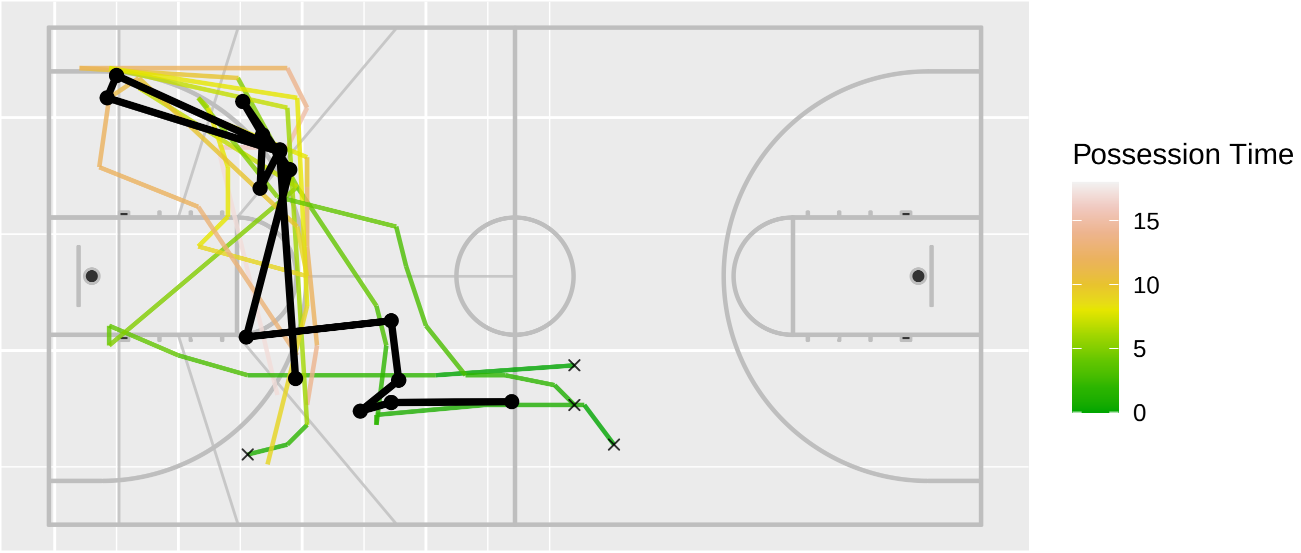

AUS Area 1 Cluster 10 : SelectTrajectories

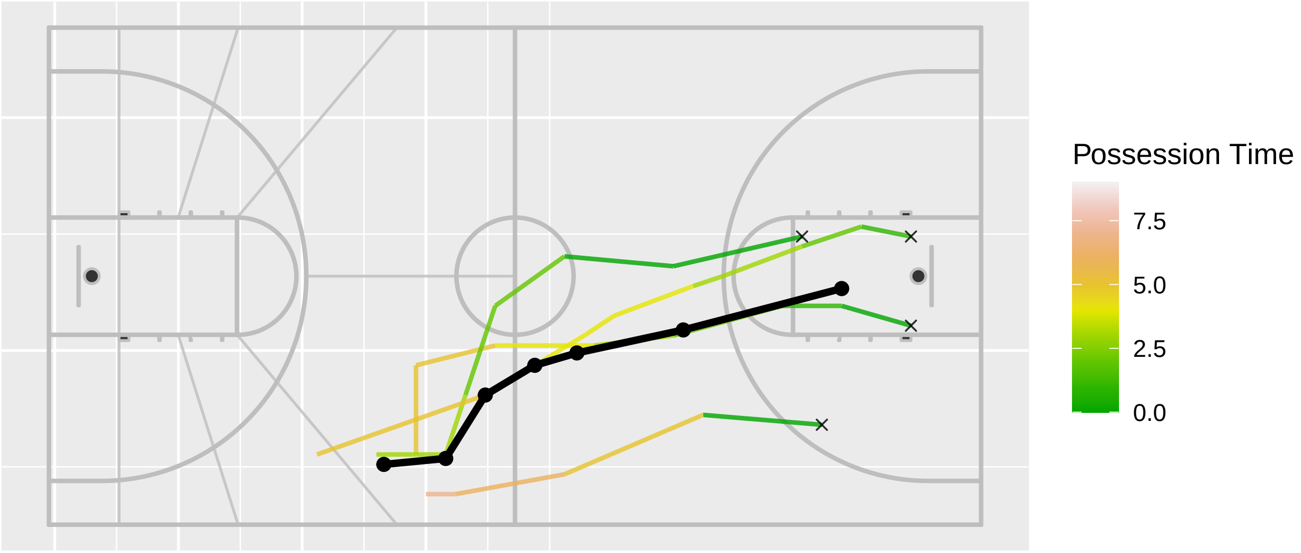

AUS Area 1 Cluster 11 : SelectTrajectories

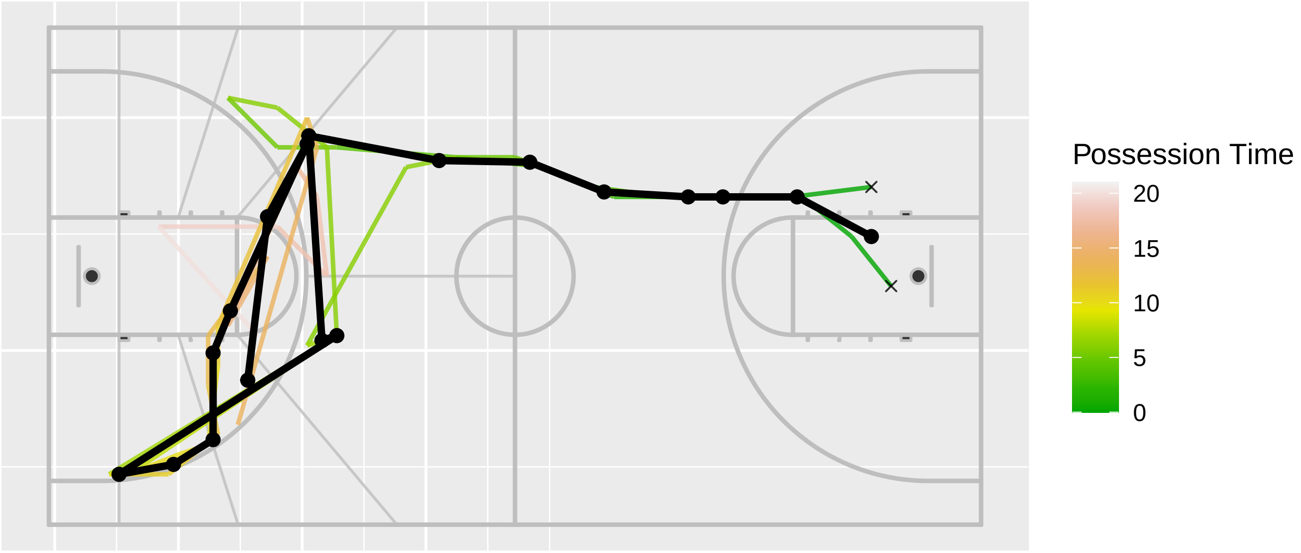

AUS Area 1 Cluster 12 : SelectTrajectories

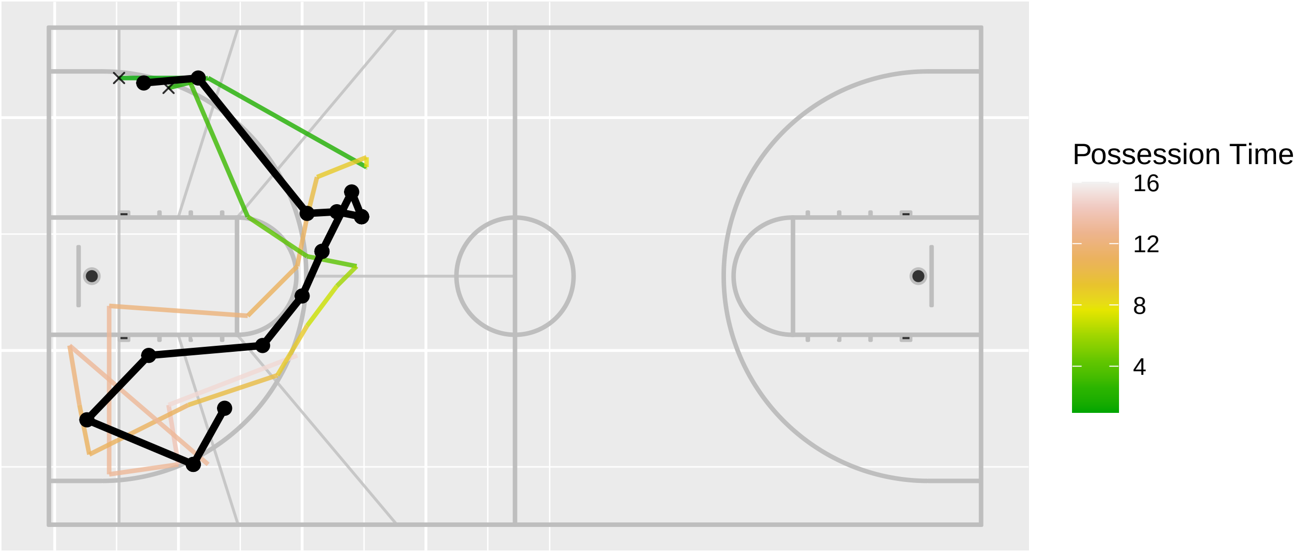

AUS Area 1 Cluster 13 : SelectTrajectories

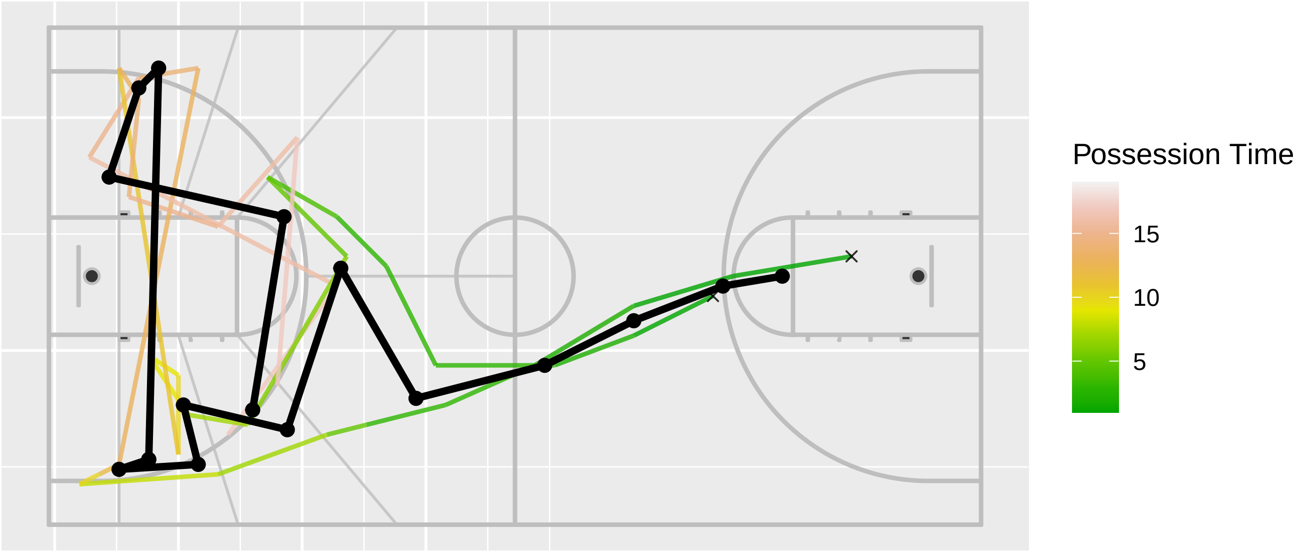

AUS Area 1 Cluster 14 : SelectTrajectories

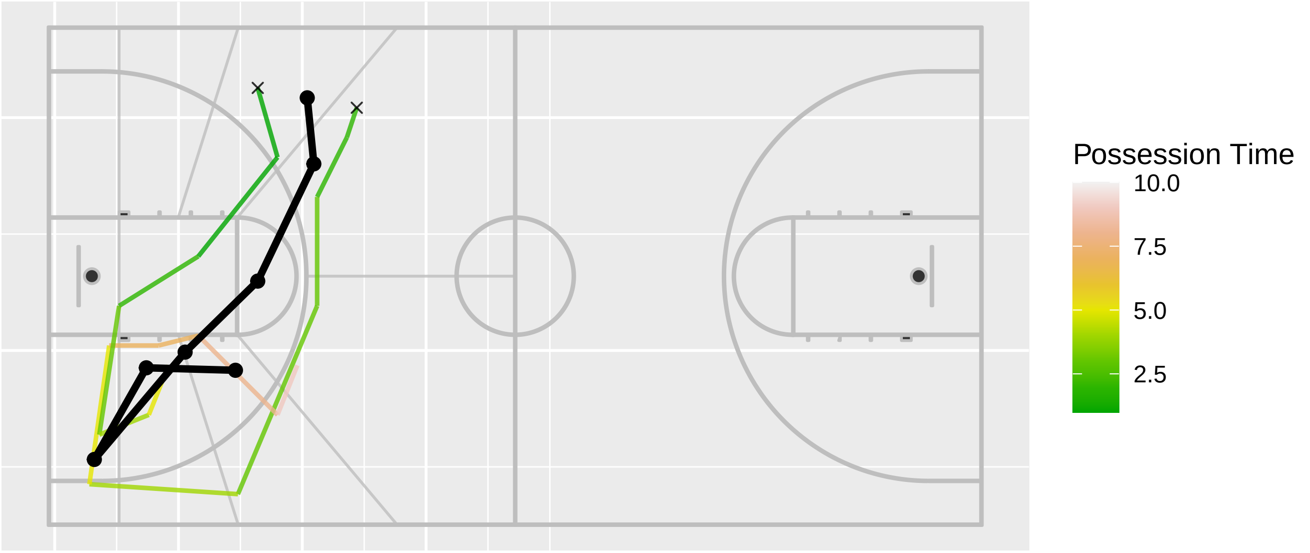

AUS Area 1 Cluster 15 : SelectTrajectories

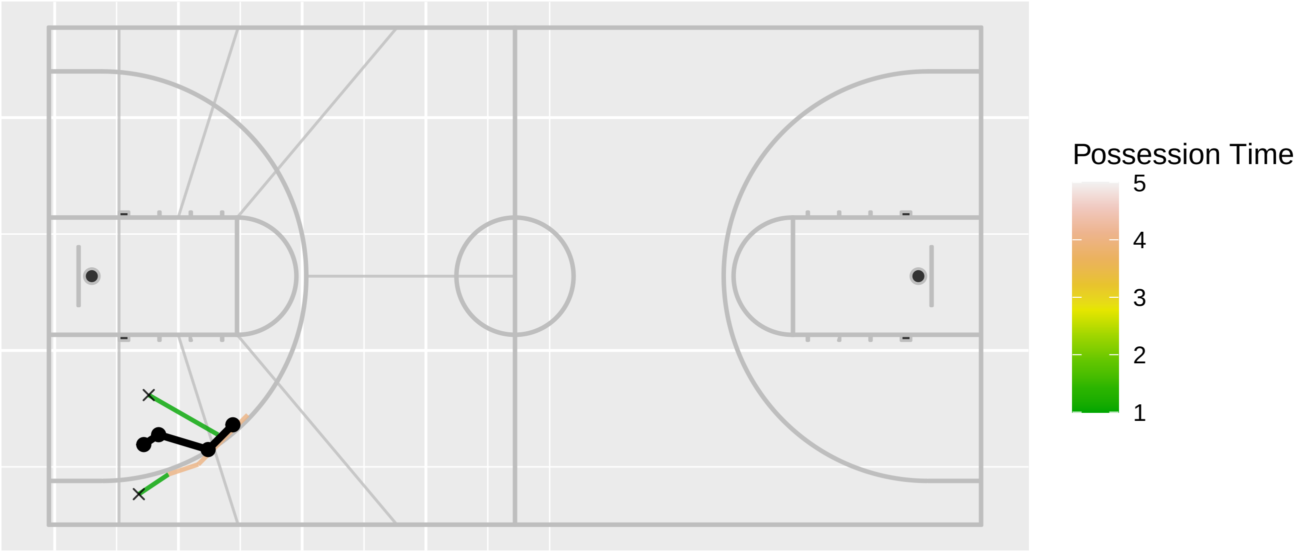

AUS Area 1 Cluster 16 : SelectTrajectories

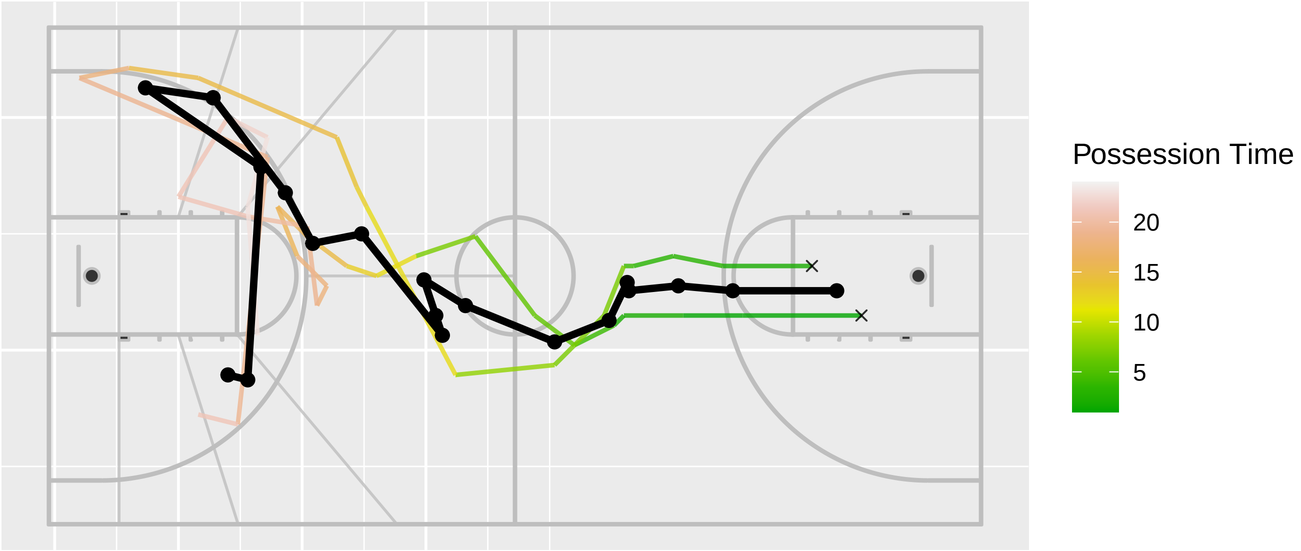

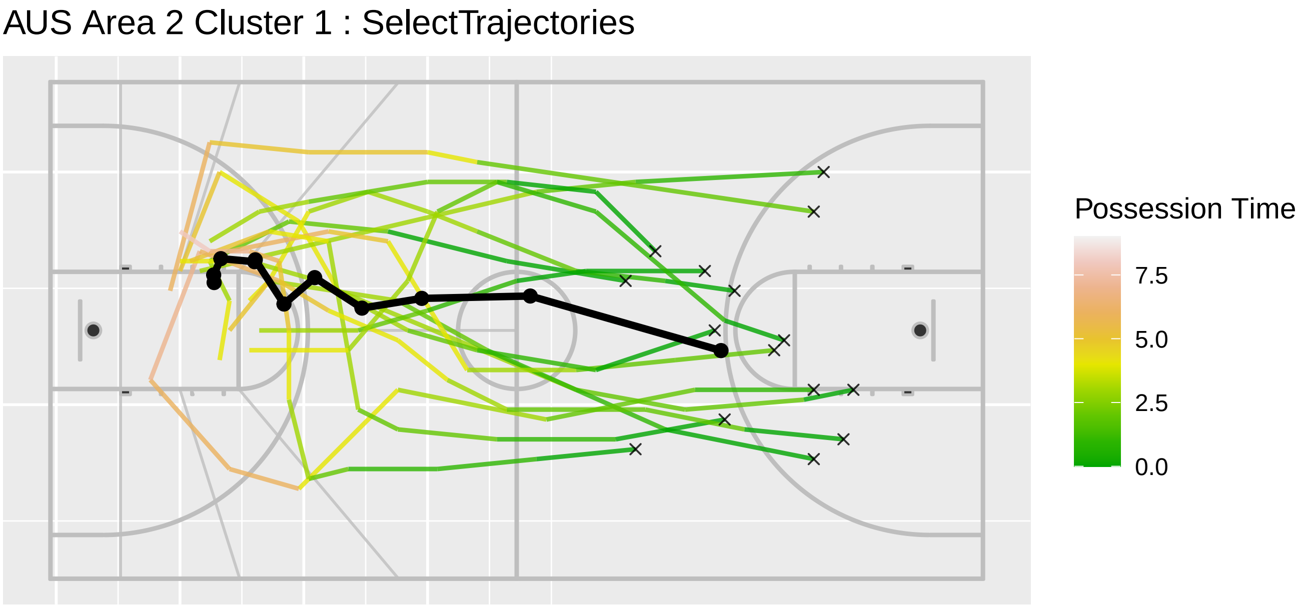

AUS Area 2 Cluster 1 : SelectTrajectories
Possession Time
7.5
5.0
2.5
0.0

AUS Area 2 Cluster 2 : SelectTrajectories

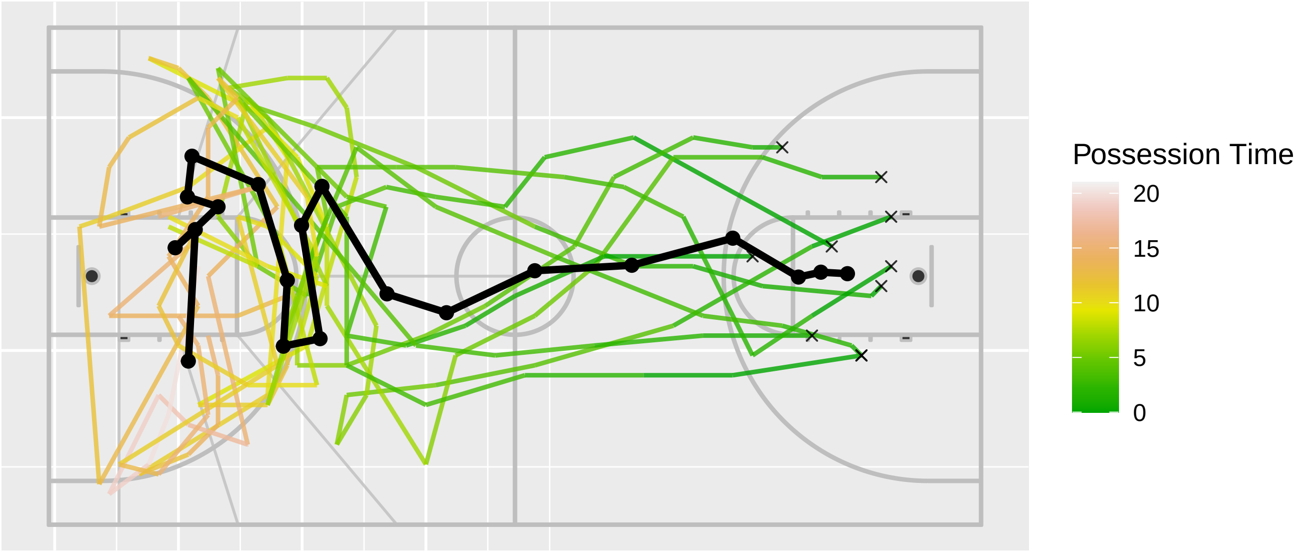

AUS Area 2 Cluster 3 : SelectTrajectories

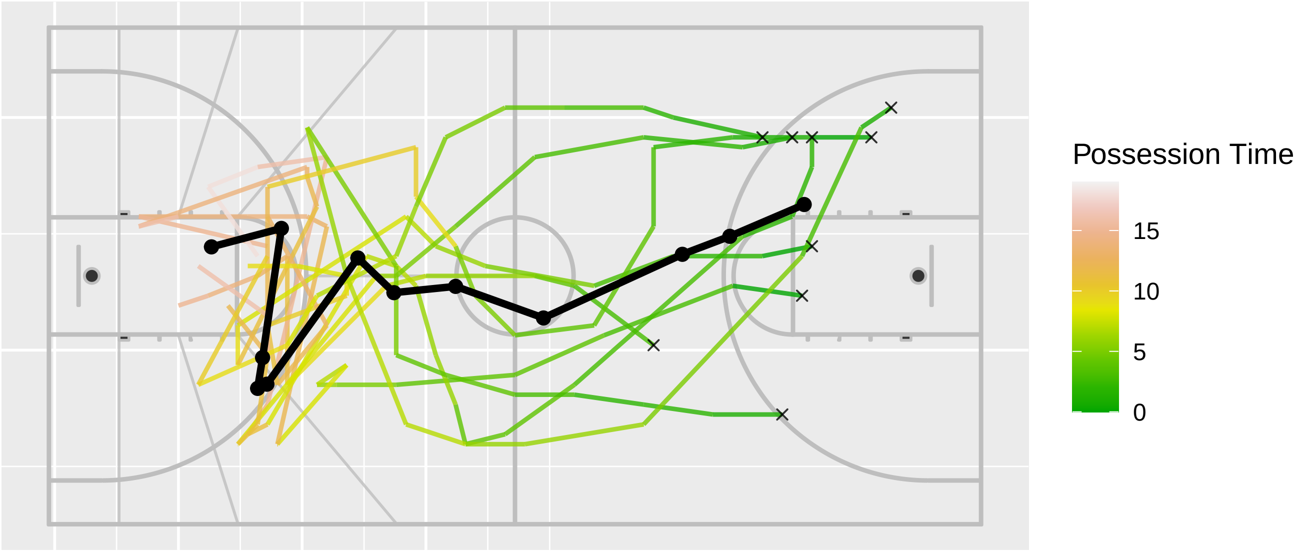

AUS Area 2 Cluster 4 : SelectTrajectories

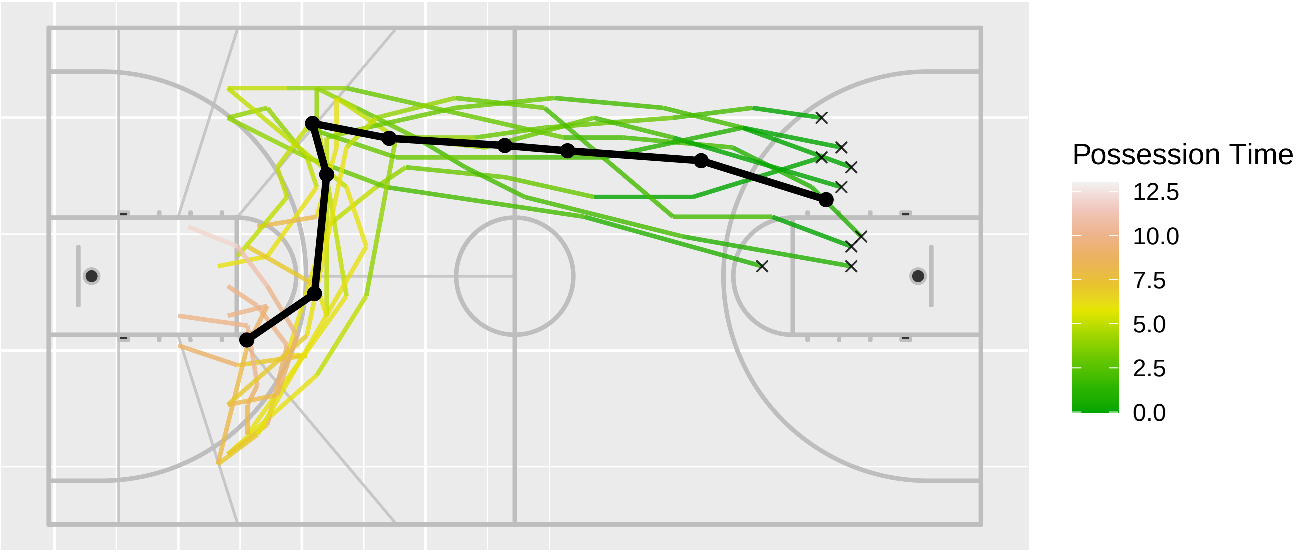

AUS Area 2 Cluster 5 : SelectTrajectories

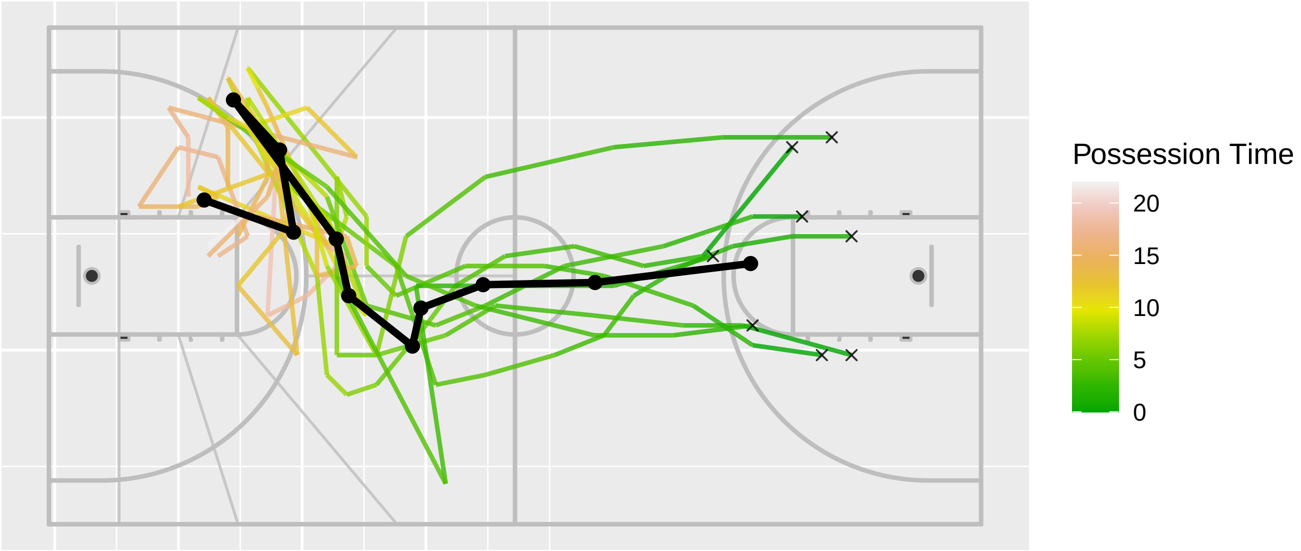

AUS Area 2 Cluster 6 : SelectTrajectories

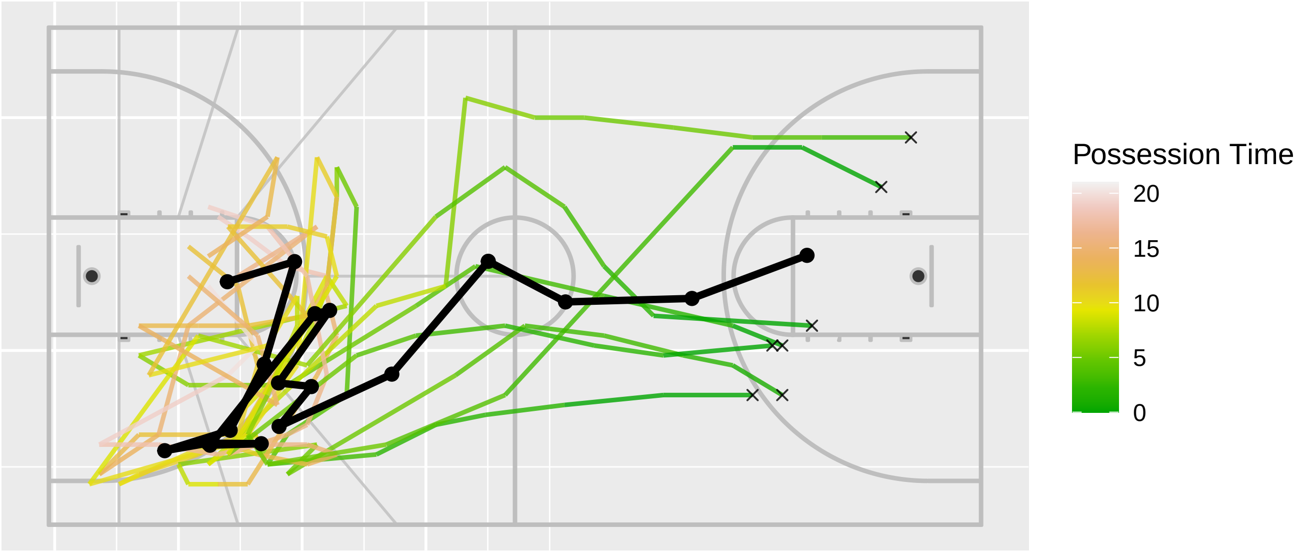

AUS Area 2 Cluster 7 : SelectTrajectories

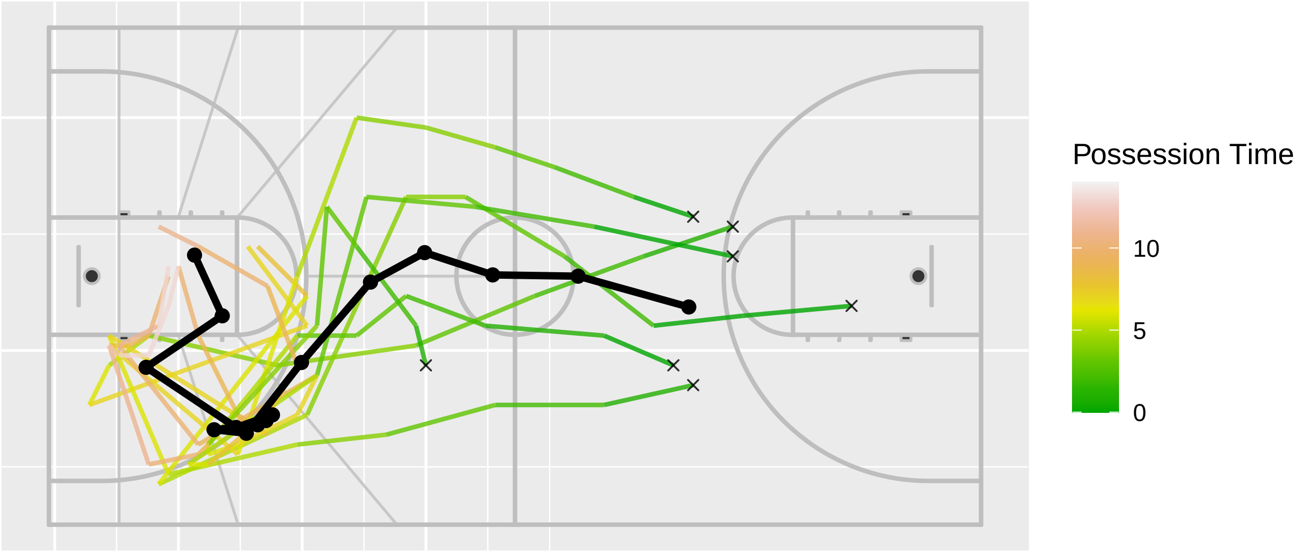

AUS Area 2 Cluster 8 : SelectTrajectories

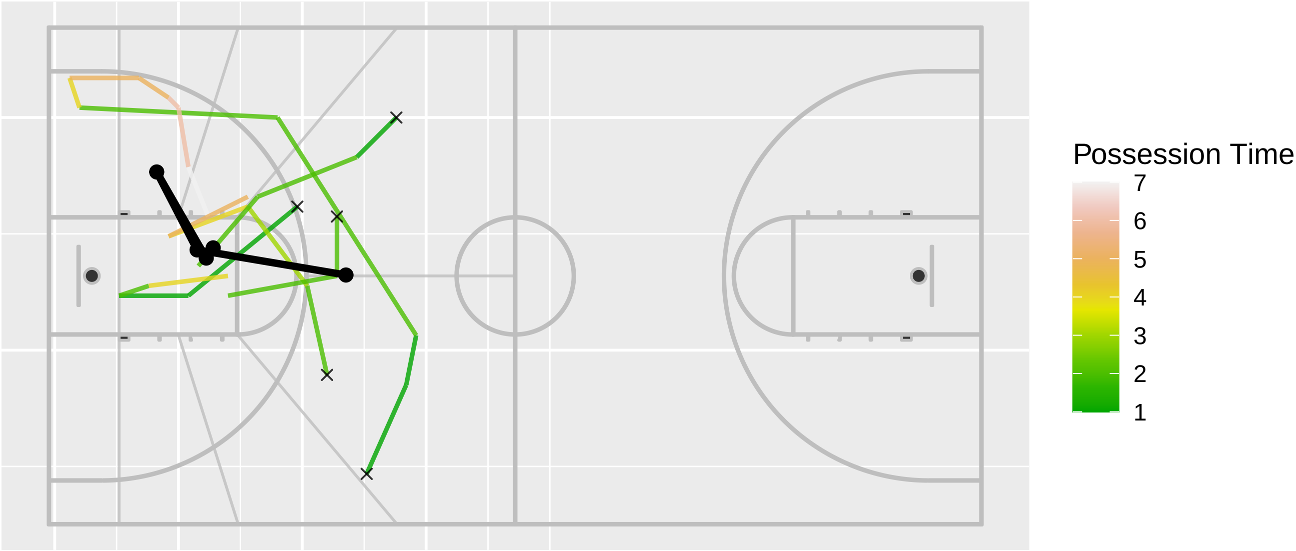

AUS Area 2 Cluster 9 : SelectTrajectories

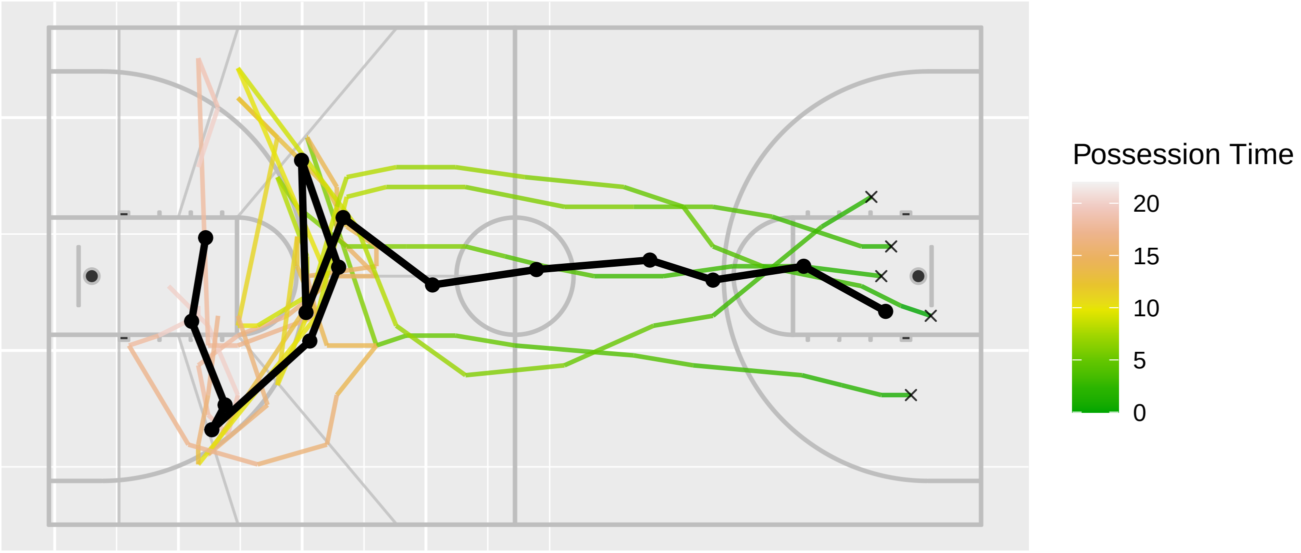

AUS Area 2 Cluster 10 : SelectTrajectories

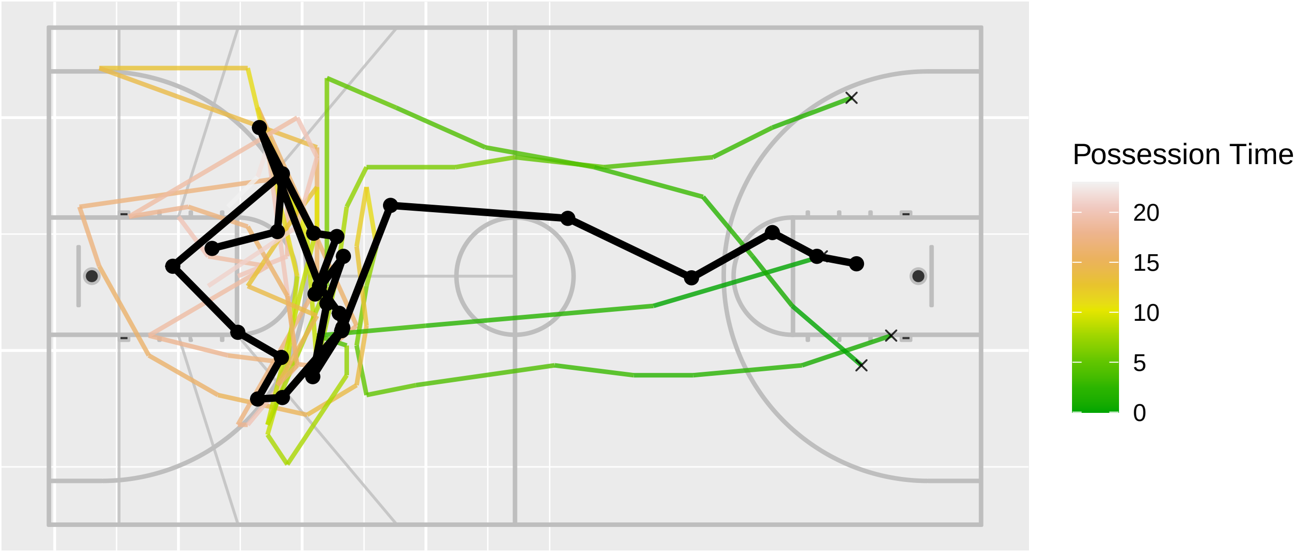

AUS Area 2 Cluster 11 : SelectTrajectories

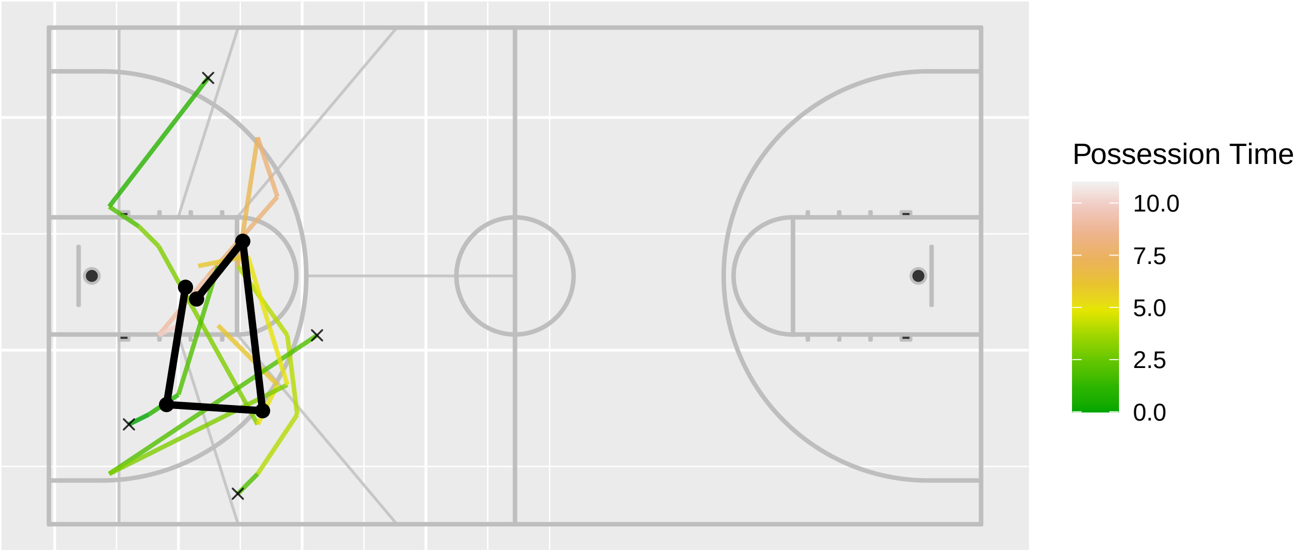

AUS Area 2 Cluster 12 : SelectTrajectories

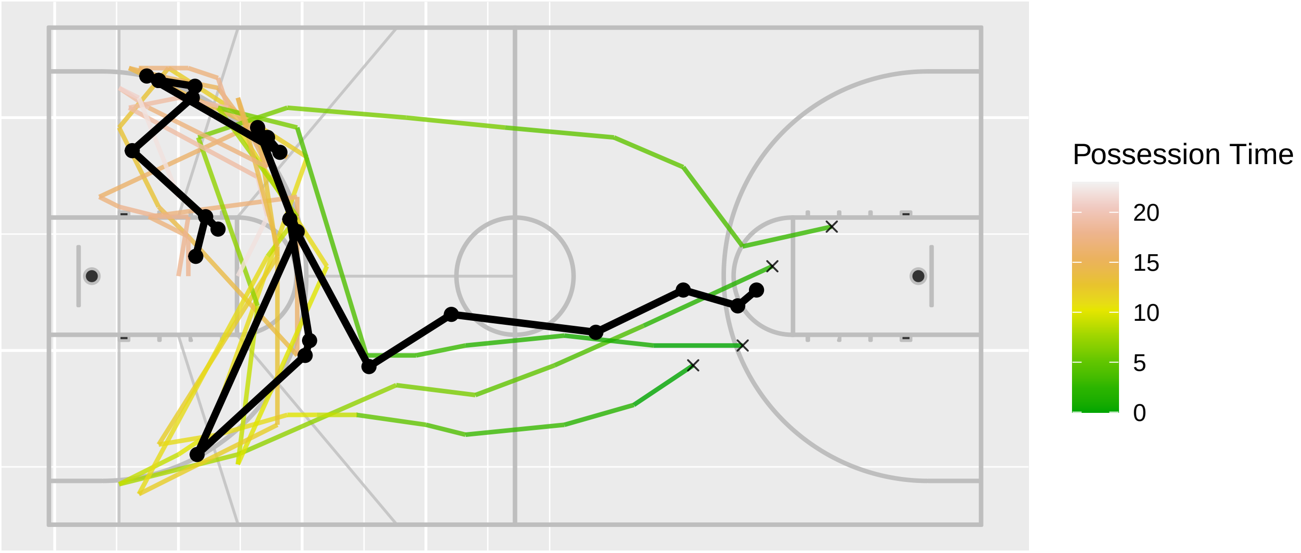

AUS Area 2 Cluster 13 : SelectTrajectories

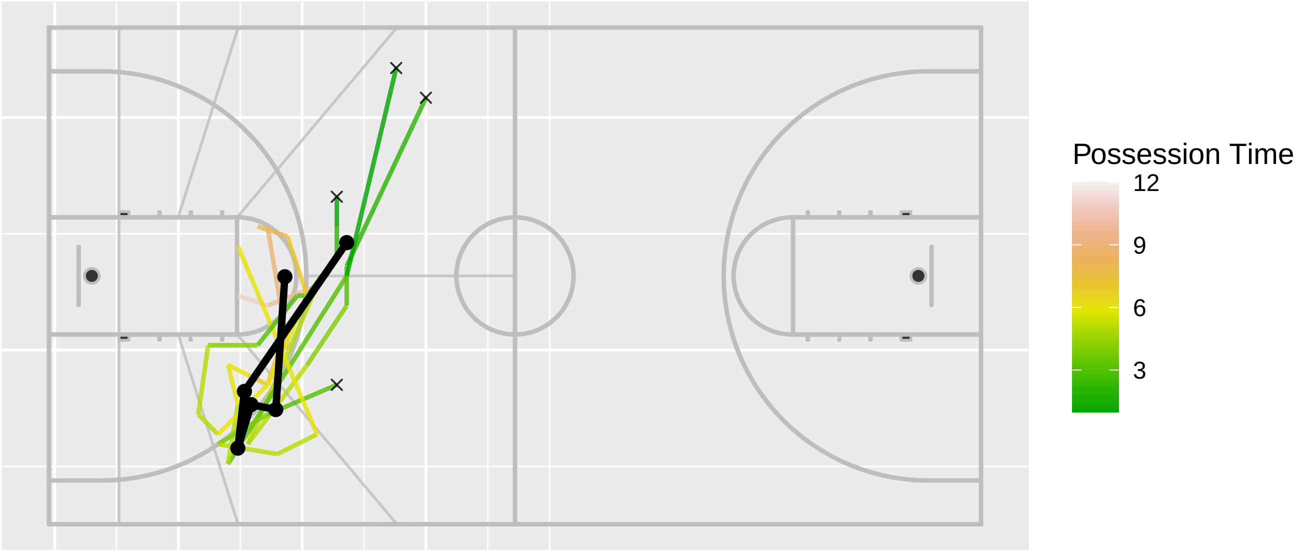

AUS Area 2 Cluster 14 : SelectTrajectories

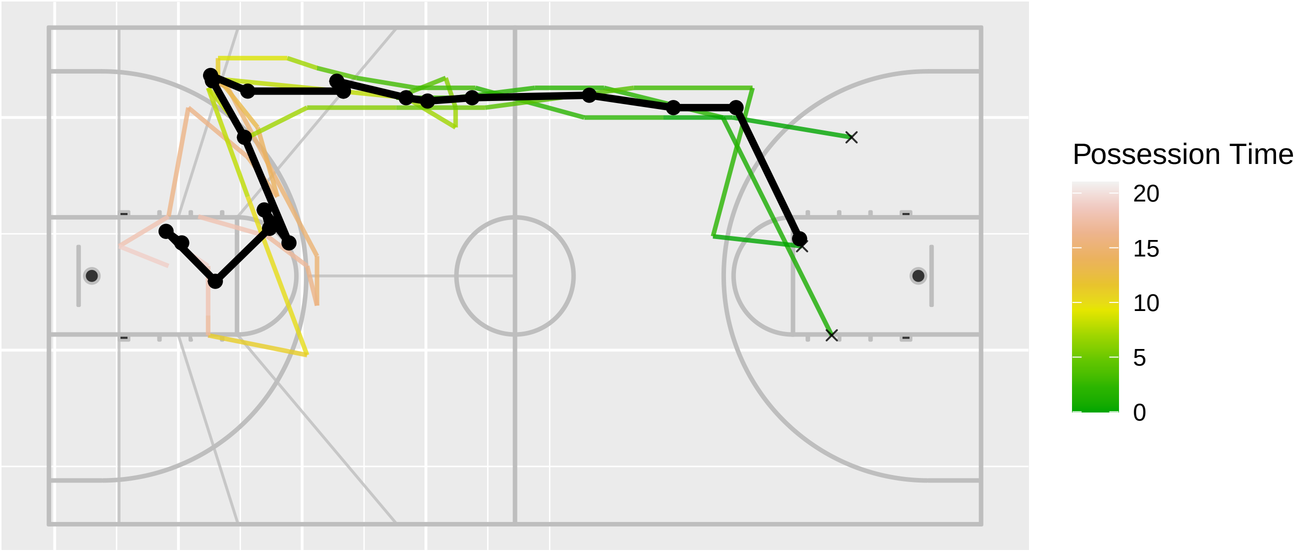

AUS Area 2 Cluster 15 : SelectTrajectories

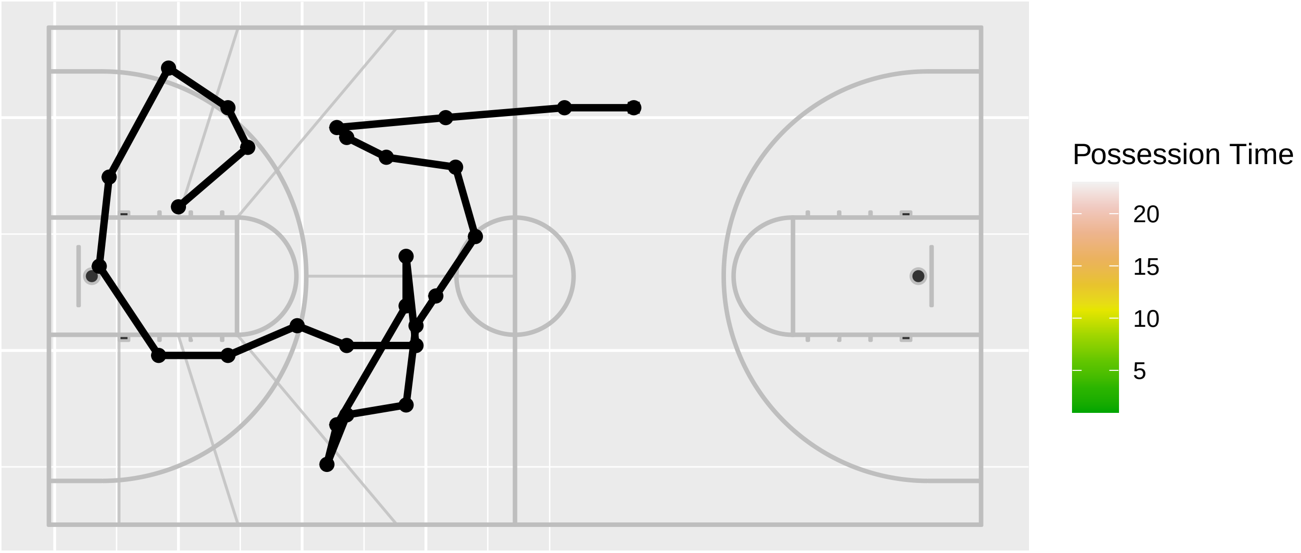

AUS Area 3 Cluster 1 : SelectTrajectories

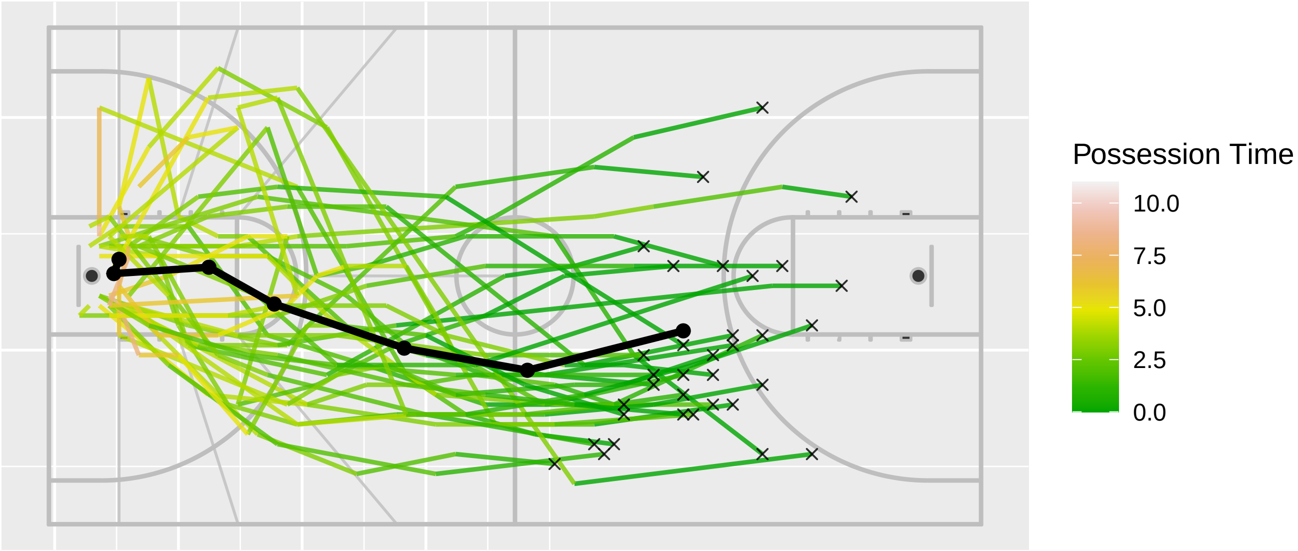

AUS Area 3 Cluster 2 : SelectTrajectories

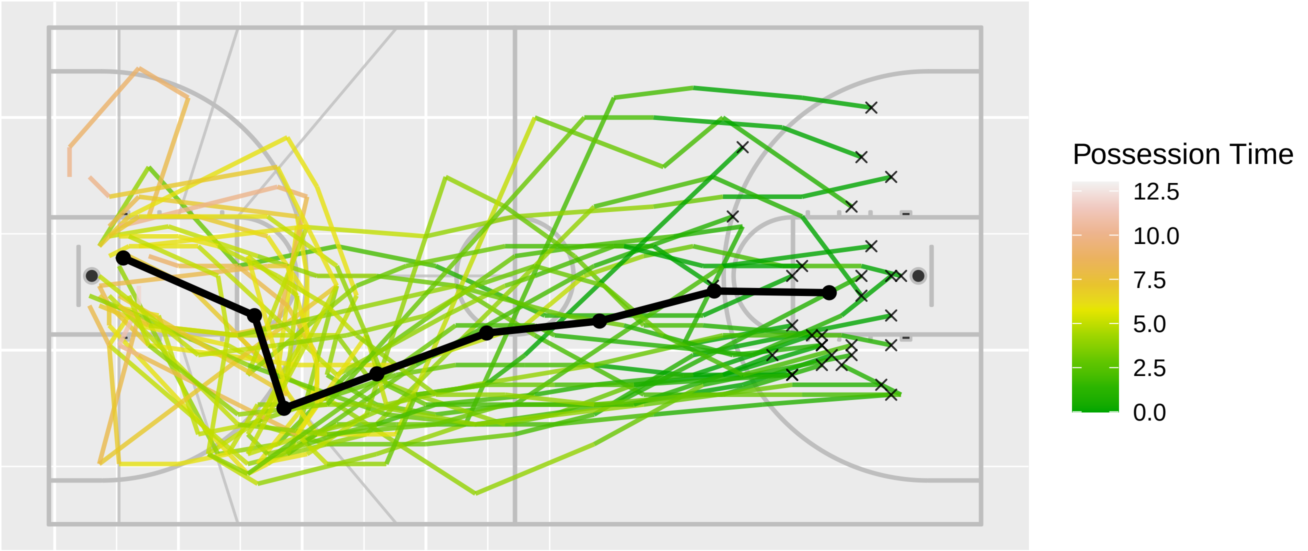

AUS Area 3 Cluster 3 : SelectTrajectories

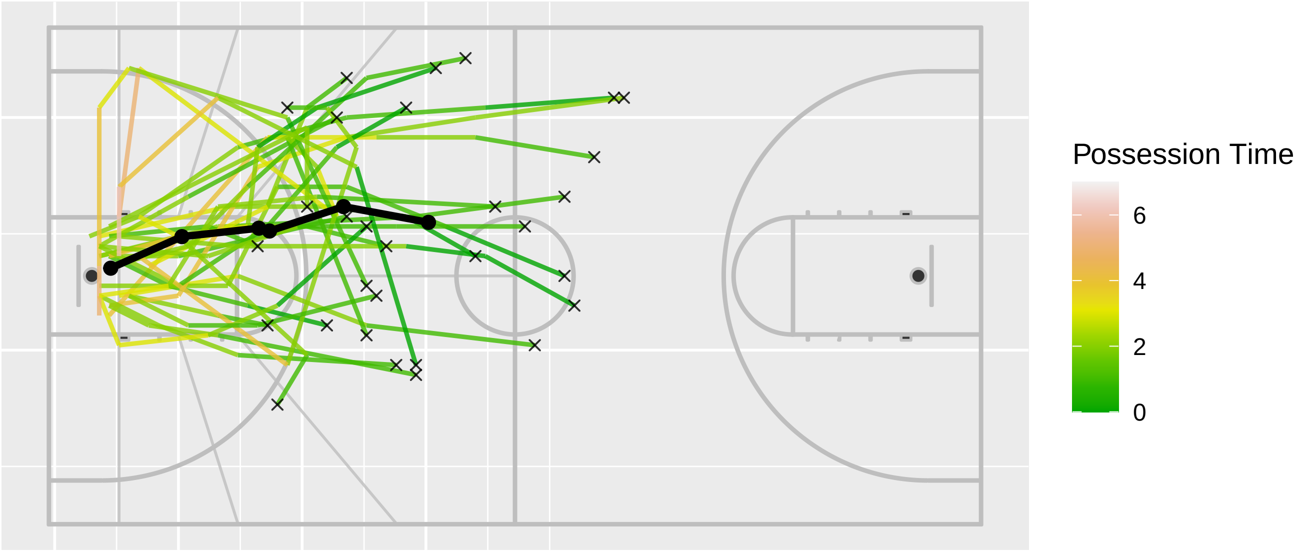

AUS Area 3 Cluster 4 : SelectTrajectories

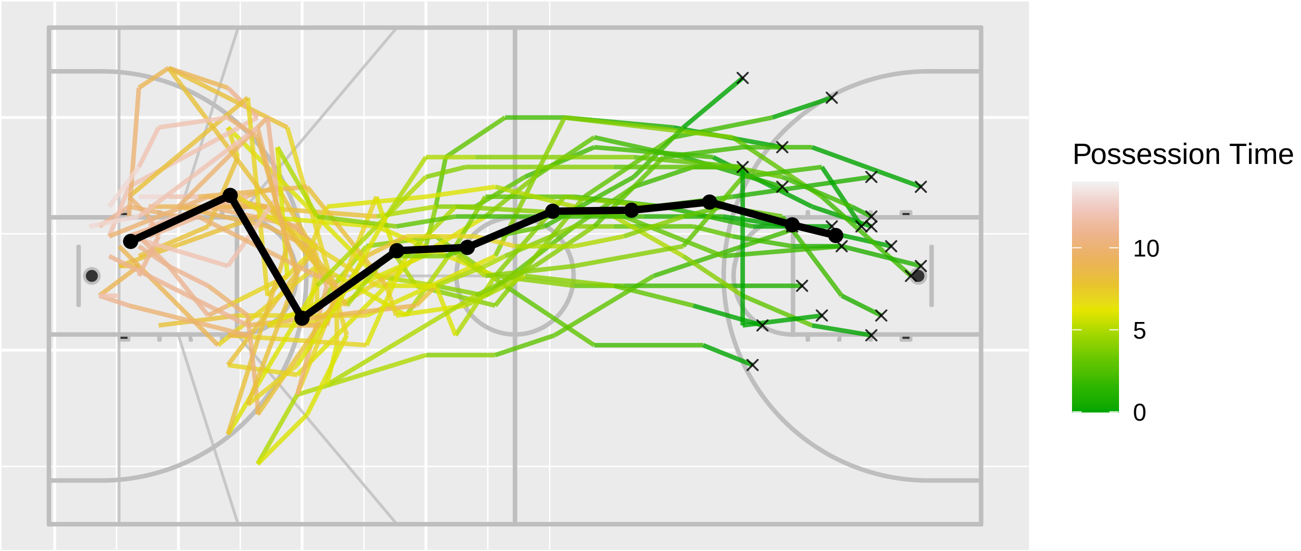

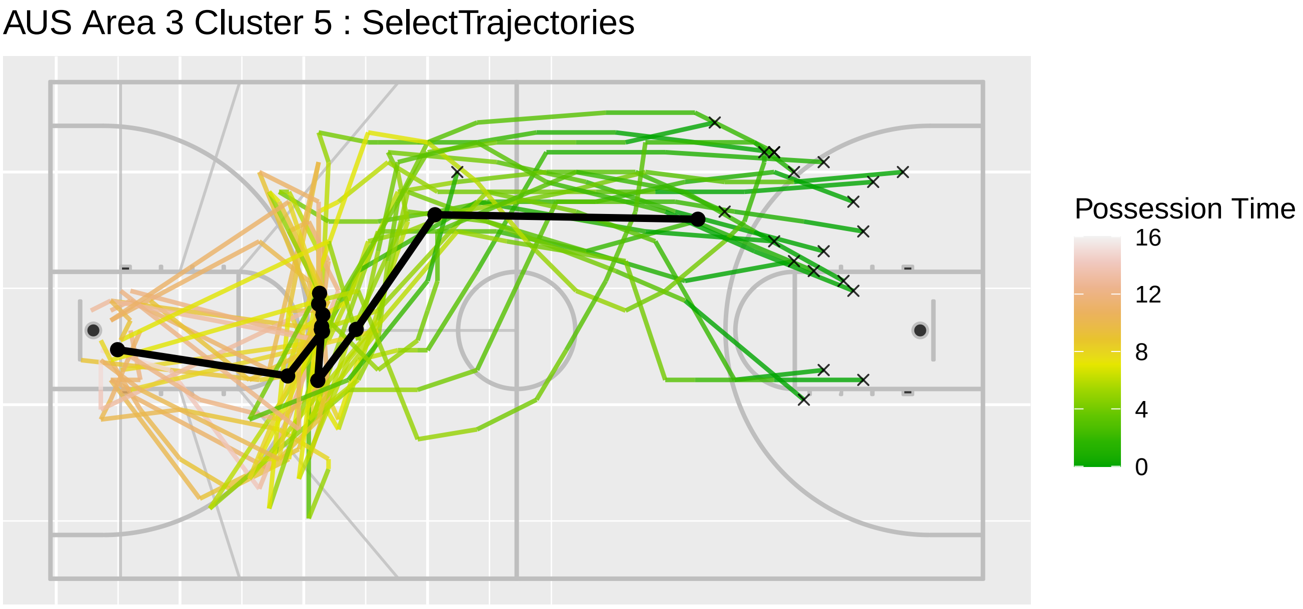
AUS Area 3 Cluster 5 : SelectTrajectories
Possession Time
16
12
8
4
0

AUS Area 3 Cluster 6 : SelectTrajectories

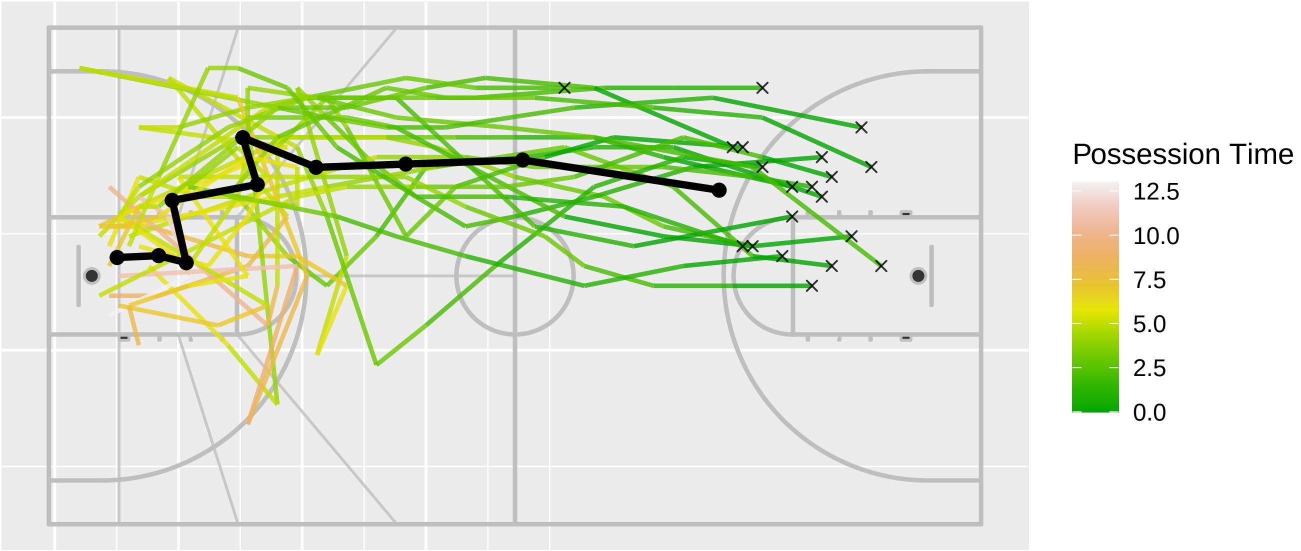

AUS Area 3 Cluster 7 : SelectTrajectories

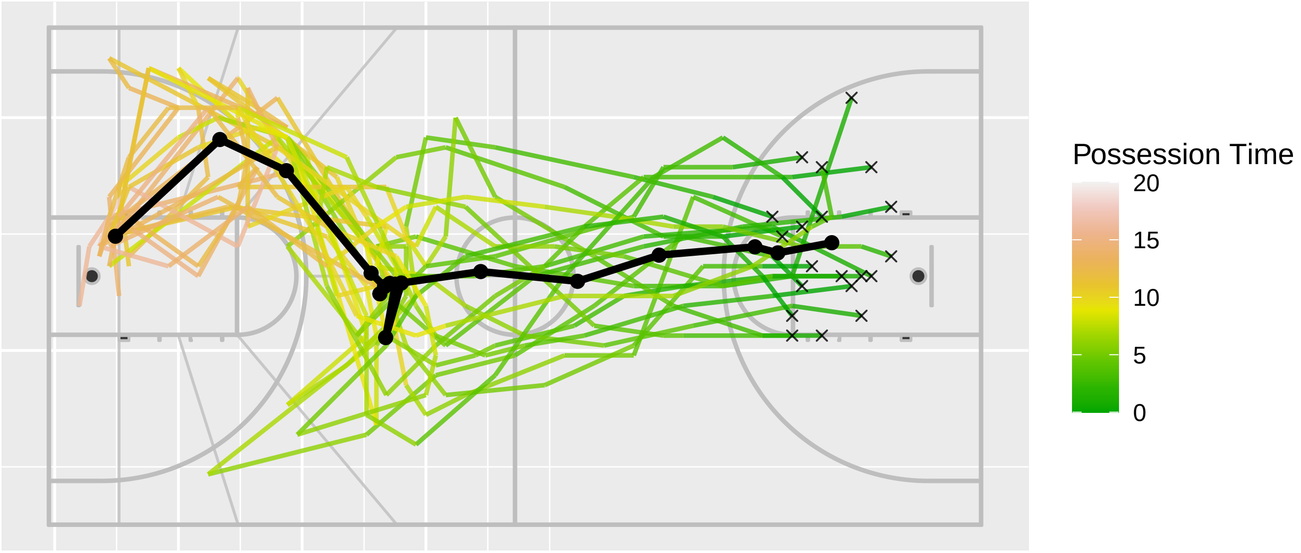

AUS Area 3 Cluster 8 : SelectTrajectories

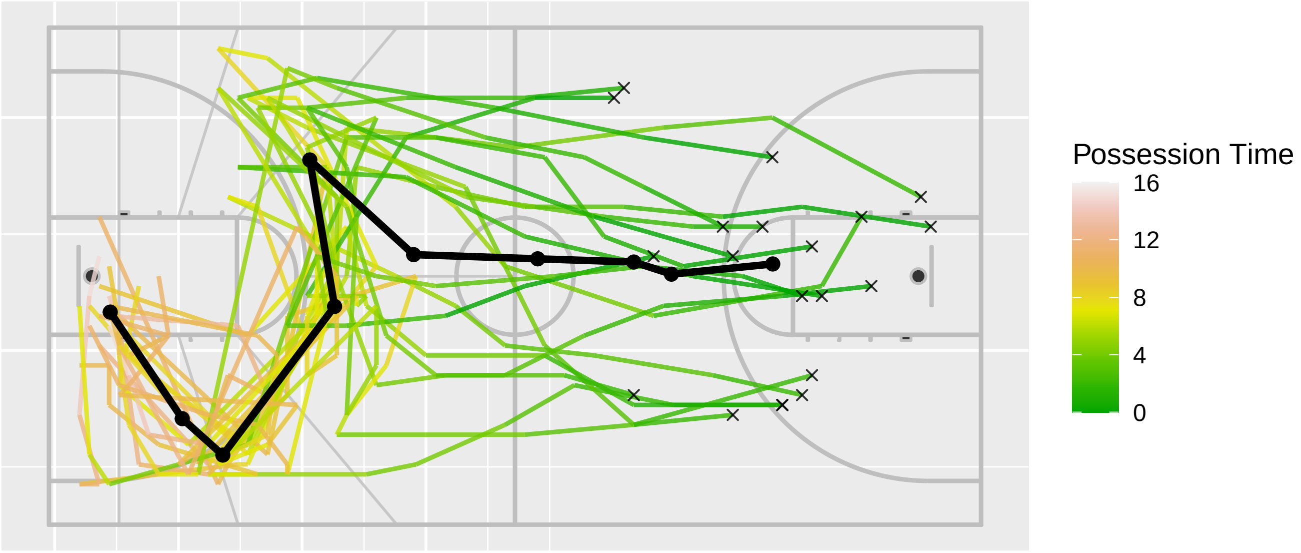

AUS Area 3 Cluster 9 : SelectTrajectories

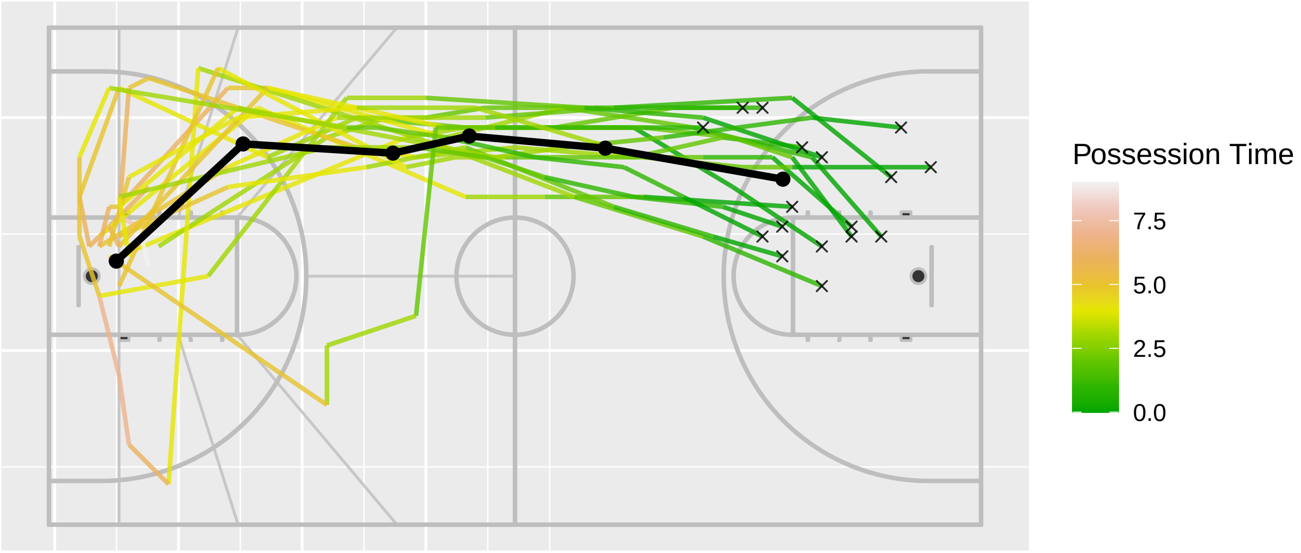

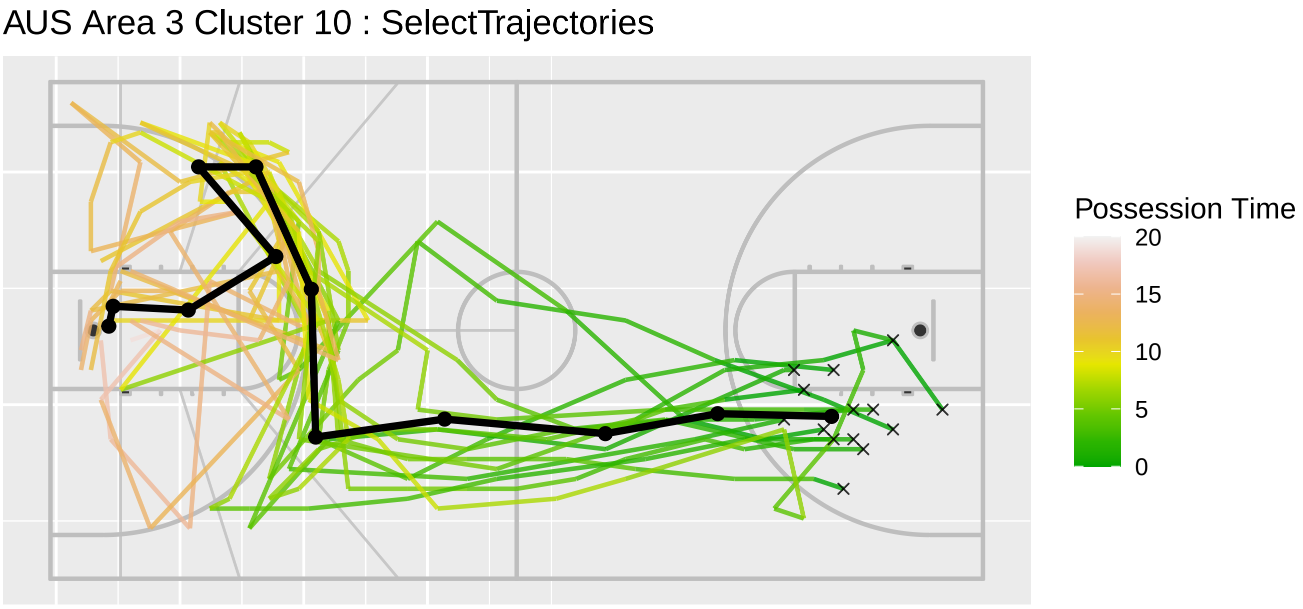
AUS Area 3 Cluster 10 : SelectTrajectories
Possession Time
20
15
10
5
0

AUS Area 3 Cluster 11 : SelectTrajectories

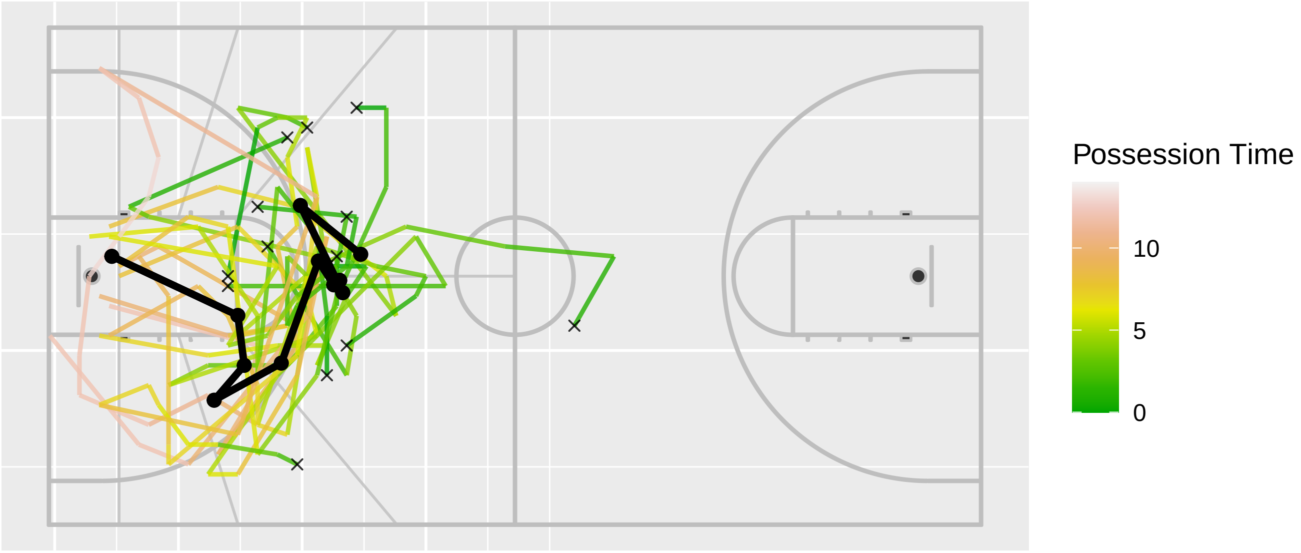

AUS Area 3 Cluster 12 : SelectTrajectories

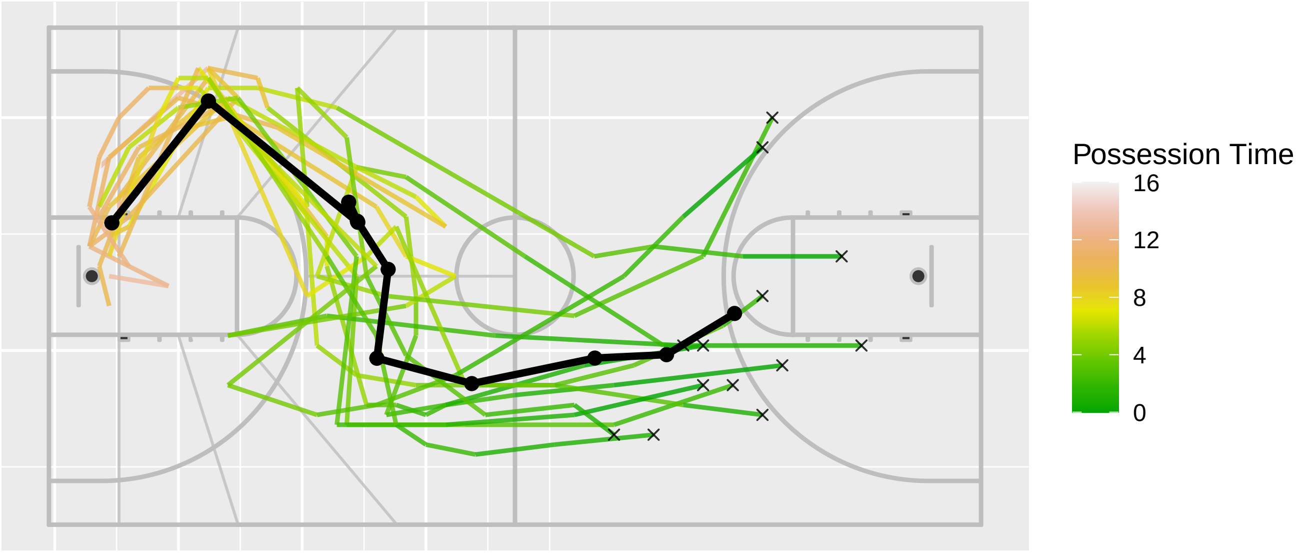

AUS Area 3 Cluster 13 : SelectTrajectories

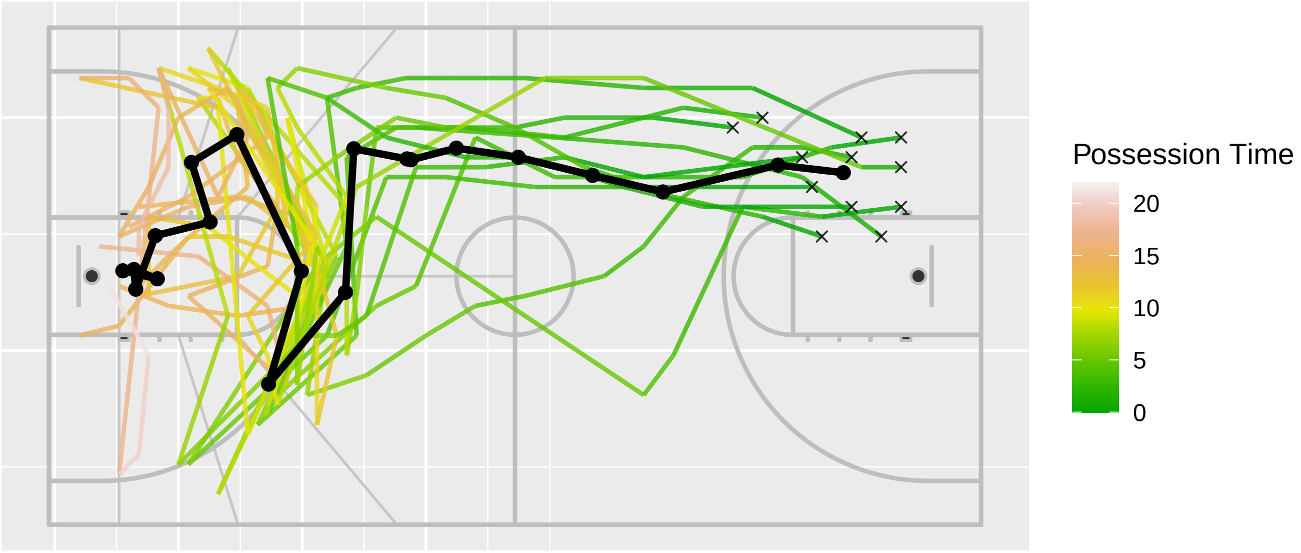

AUS Area 3 Cluster 14 : SelectTrajectories

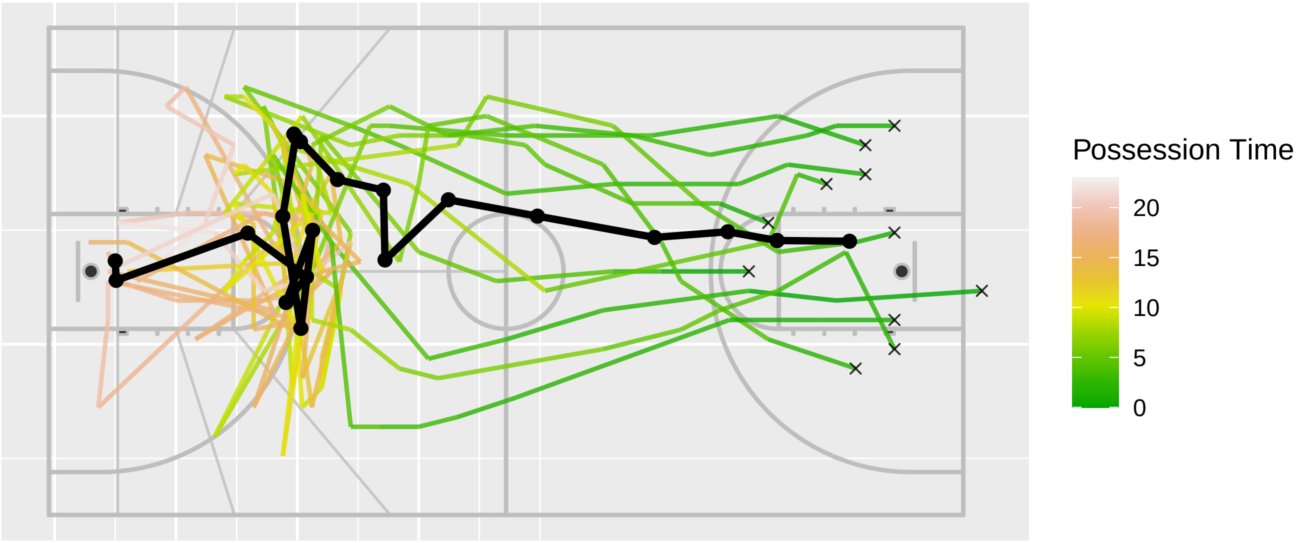

AUS Area 3 Cluster 15 : SelectTrajectories

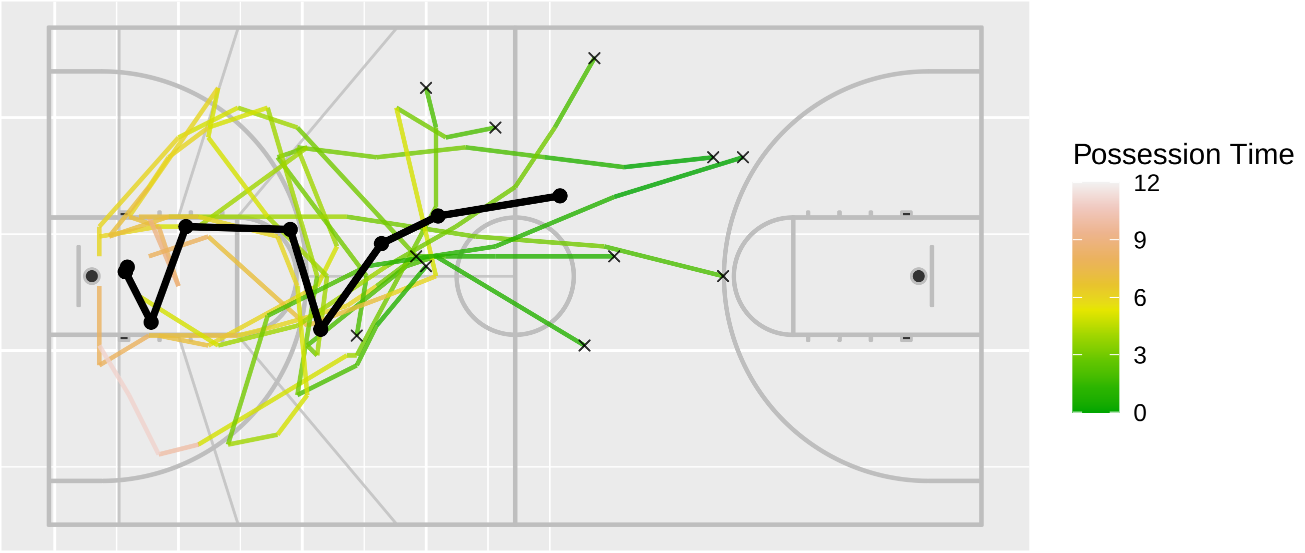

AUS Area 3 Cluster 16 : SelectTrajectories

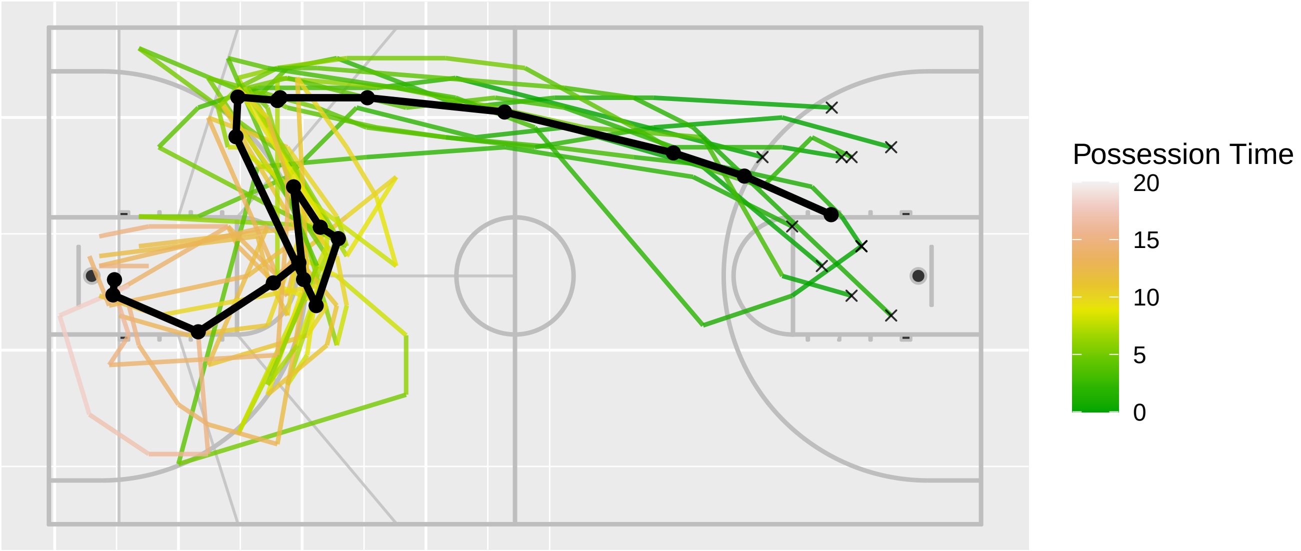

AUS Area 3 Cluster 17 : SelectTrajectories

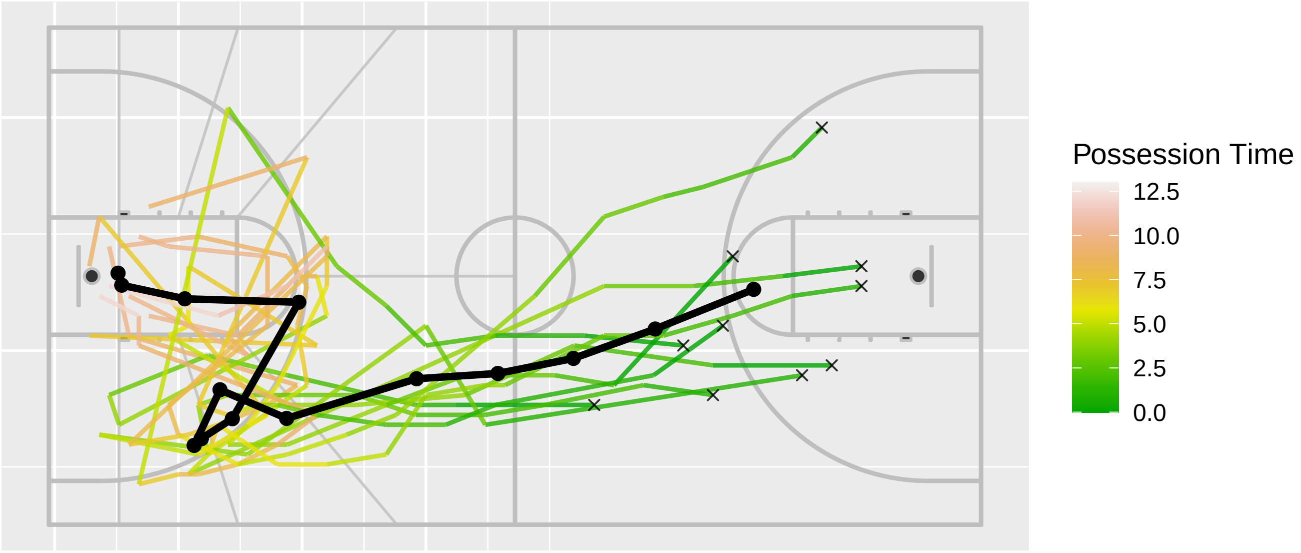

AUS Area 3 Cluster 18 : SelectTrajectories

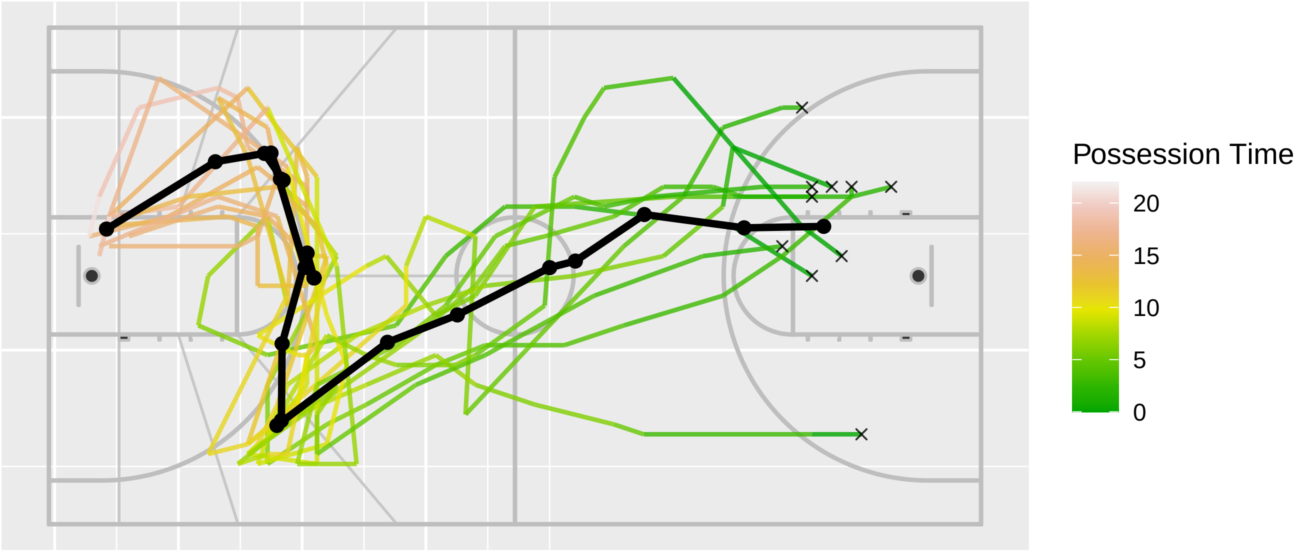

AUS Area 3 Cluster 19 : SelectTrajectories

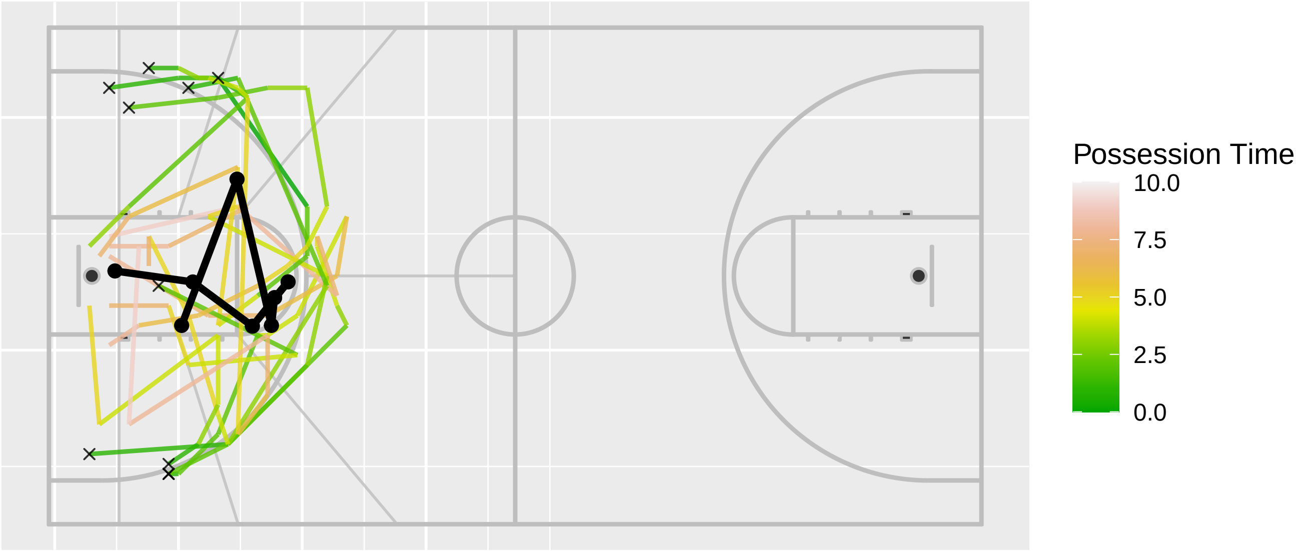

AUS Area 3 Cluster 20 : SelectTrajectories

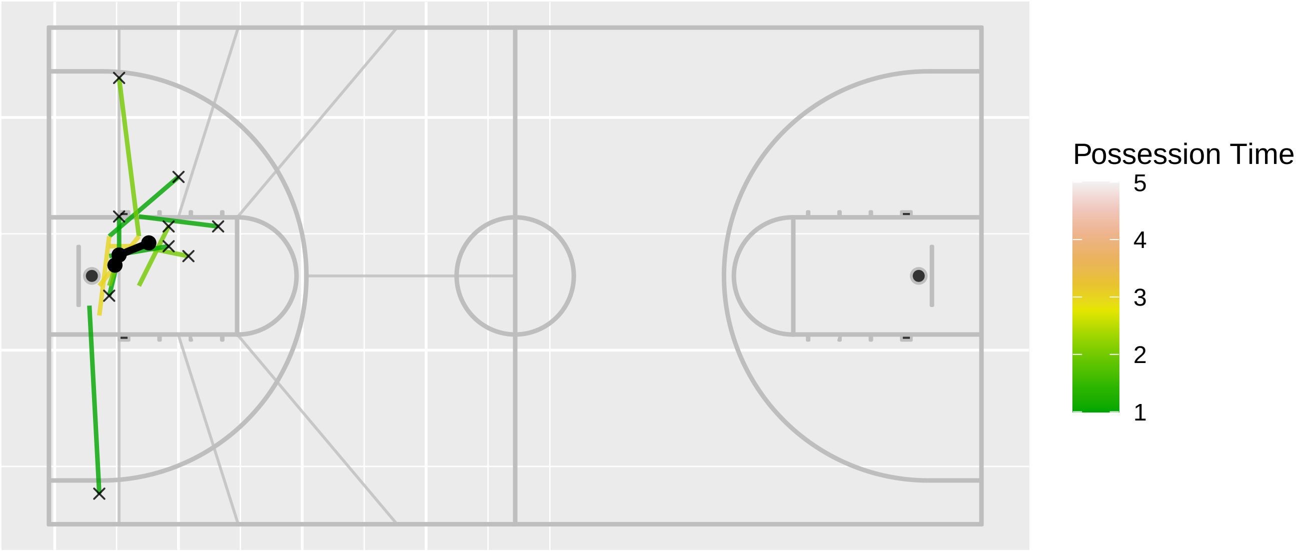

AUS Area 3 Cluster 21 : SelectTrajectories

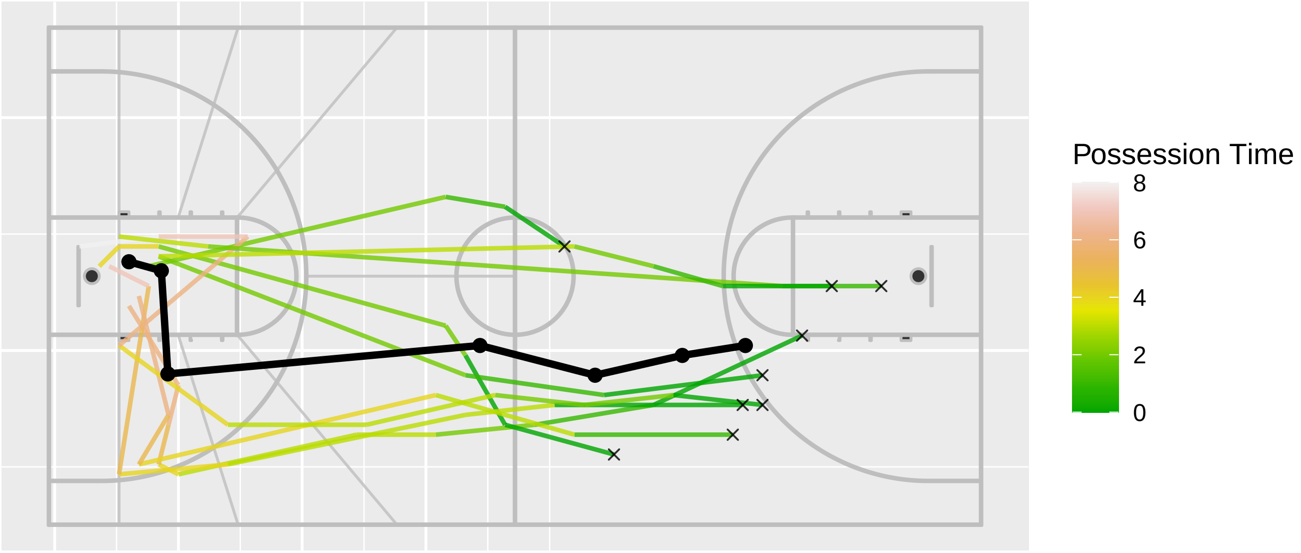

AUS Area 3 Cluster 22 : SelectTrajectories

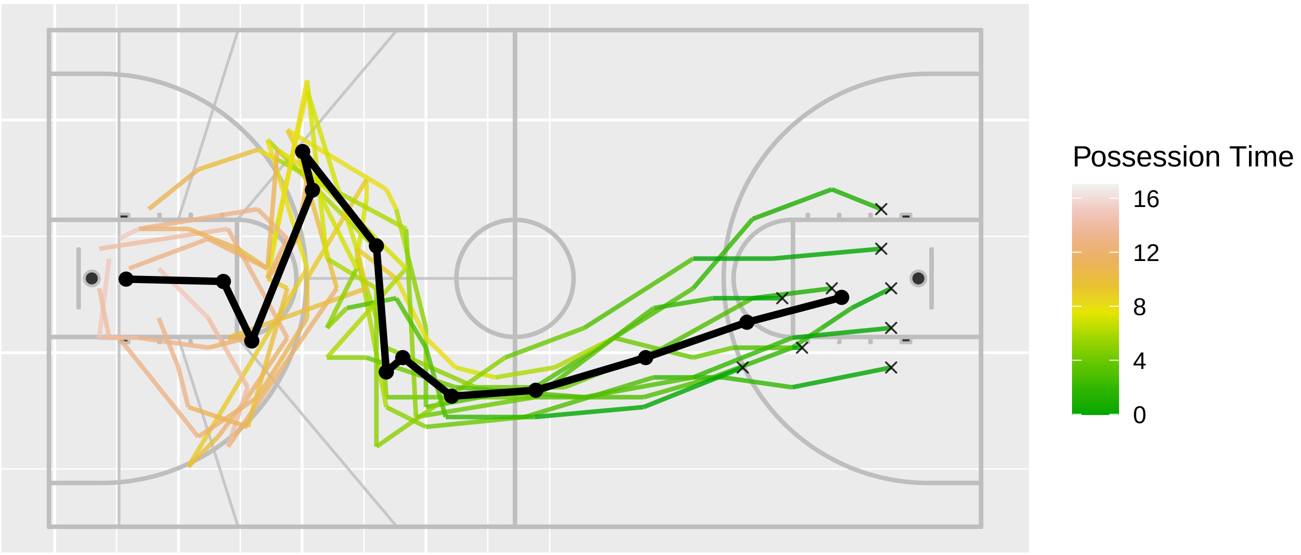

AUS Area 3 Cluster 23 : SelectTrajectories

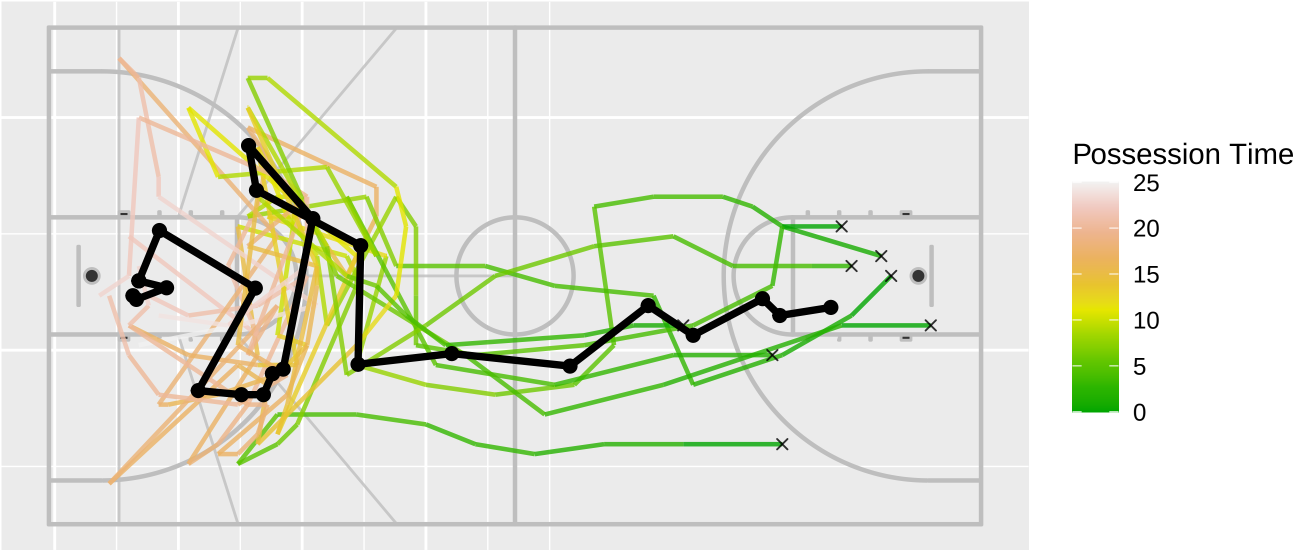

AUS Area 3 Cluster 24 : SelectTrajectories

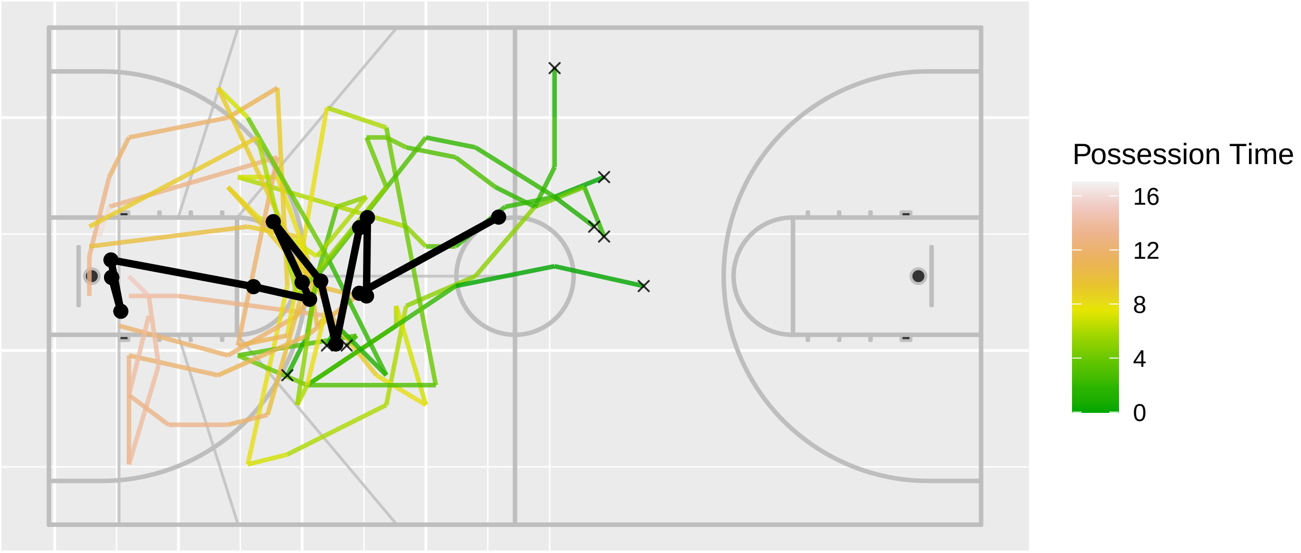

AUS Area 3 Cluster 25 : SelectTrajectories

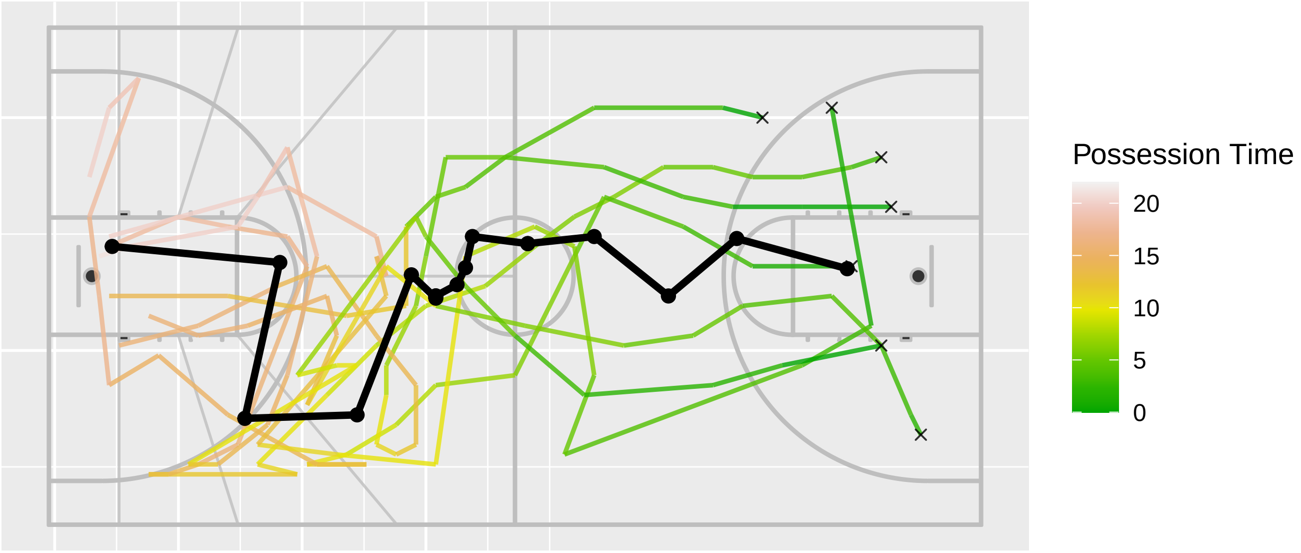

AUS Area 3 Cluster 26 : SelectTrajectories

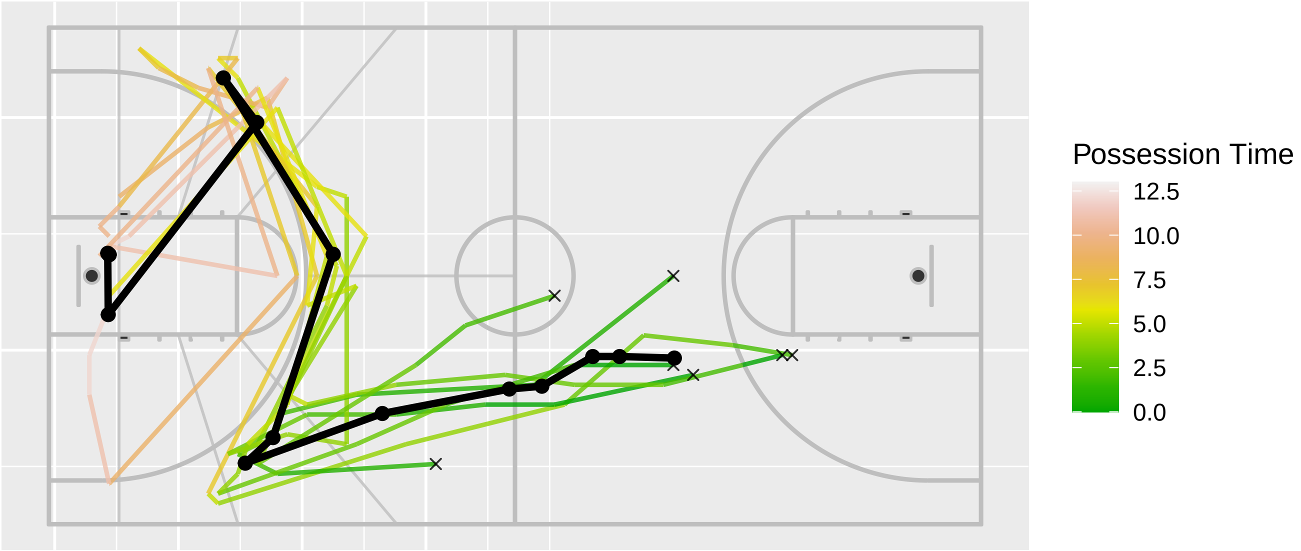

AUS Area 3 Cluster 27 : SelectTrajectories

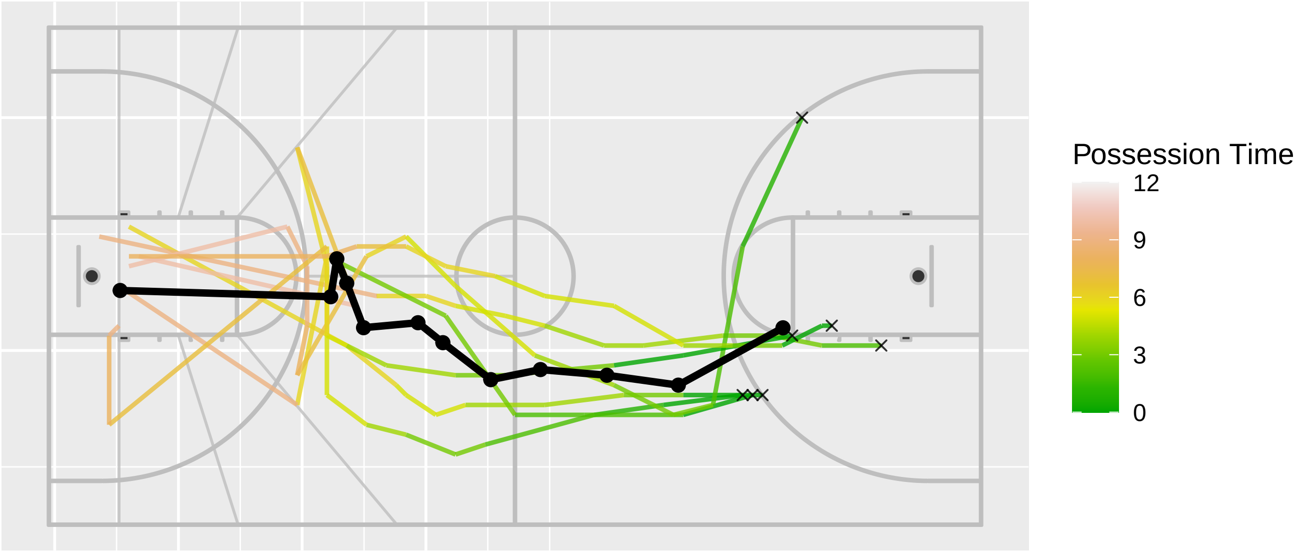

AUS Area 3 Cluster 28 : SelectTrajectories

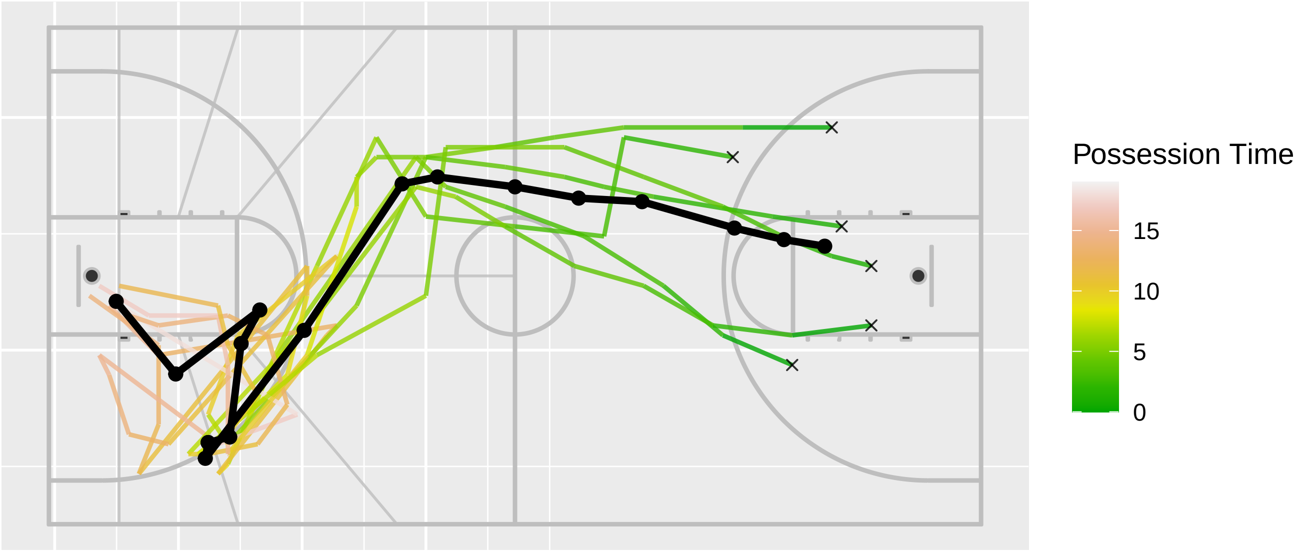

AUS Area 3 Cluster 29 : SelectTrajectories

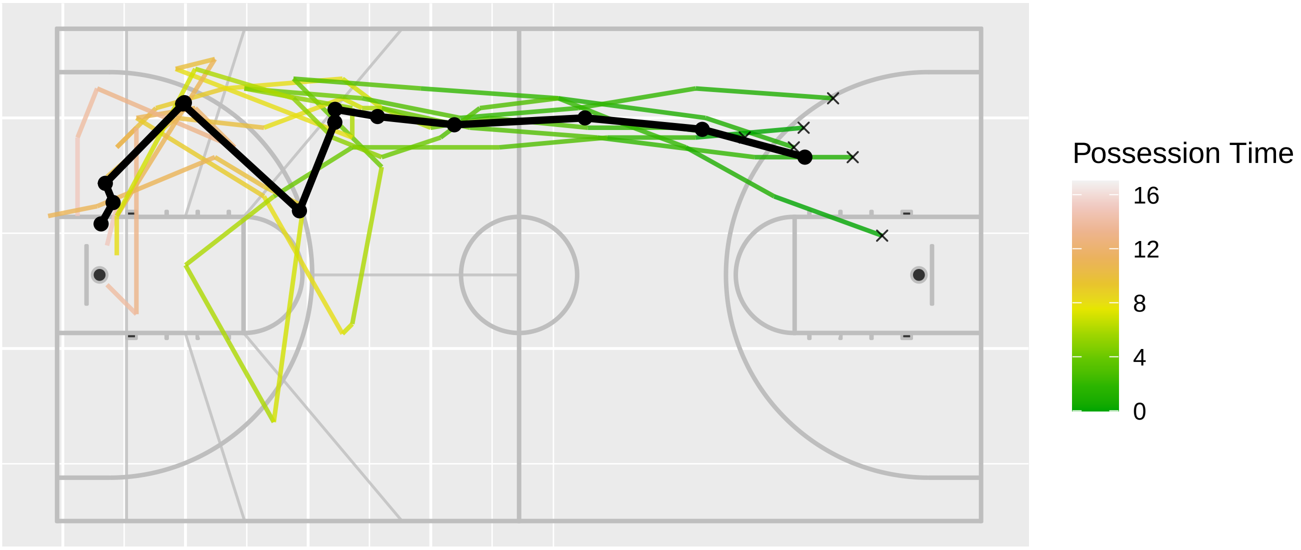

AUS Area 3 Cluster 30 : SelectTrajectories

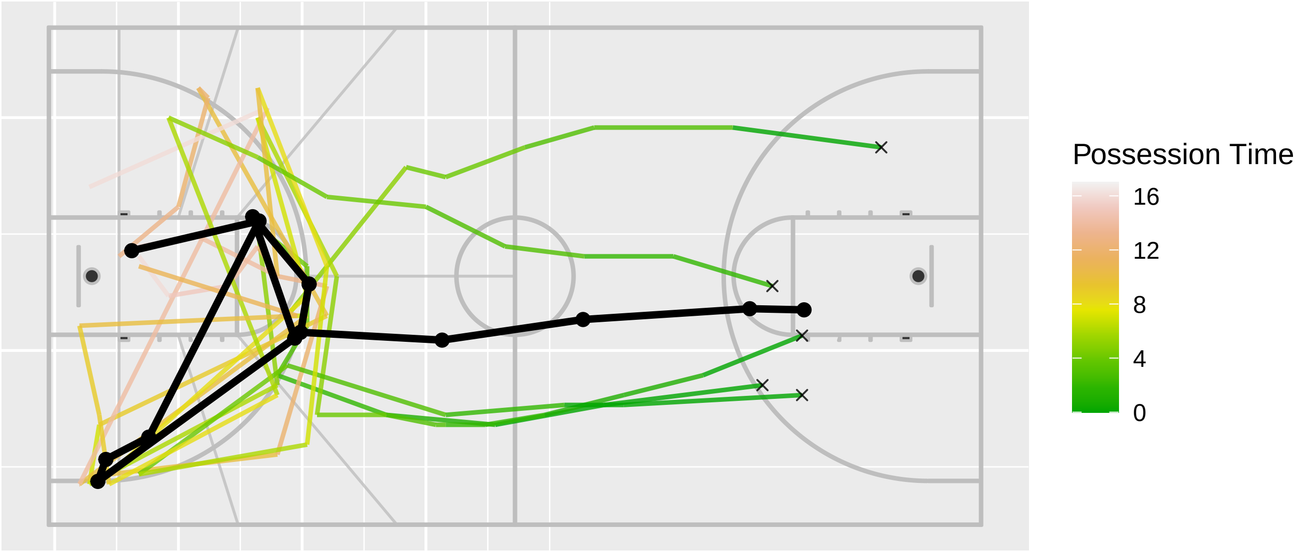

AUS Area 3 Cluster 31 : SelectTrajectories

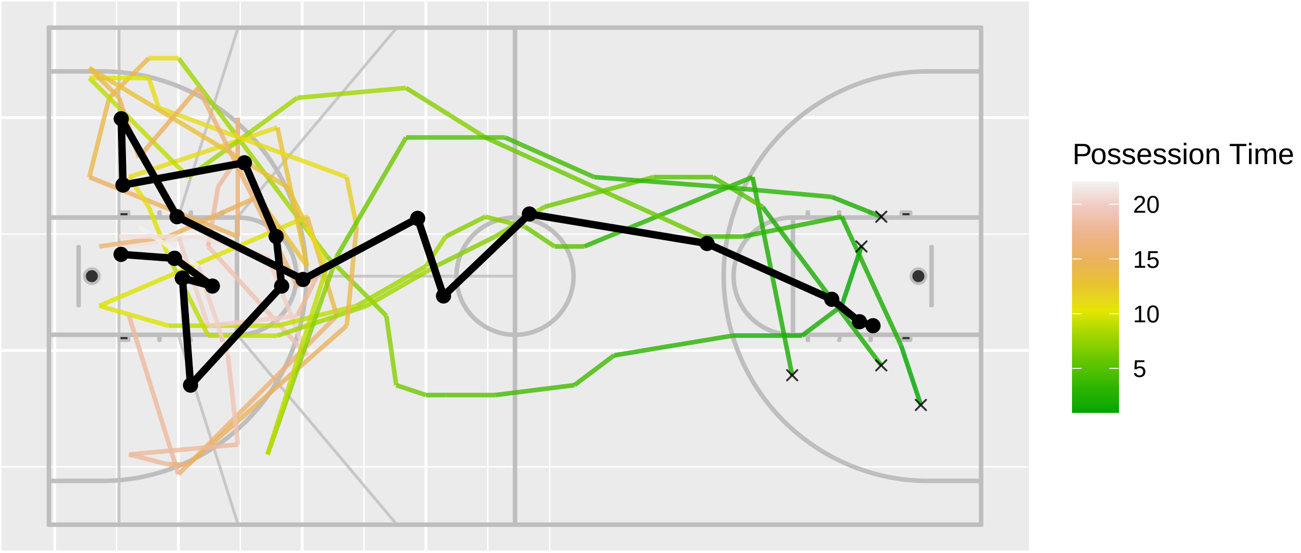

AUS Area 3 Cluster 32 : SelectTrajectories

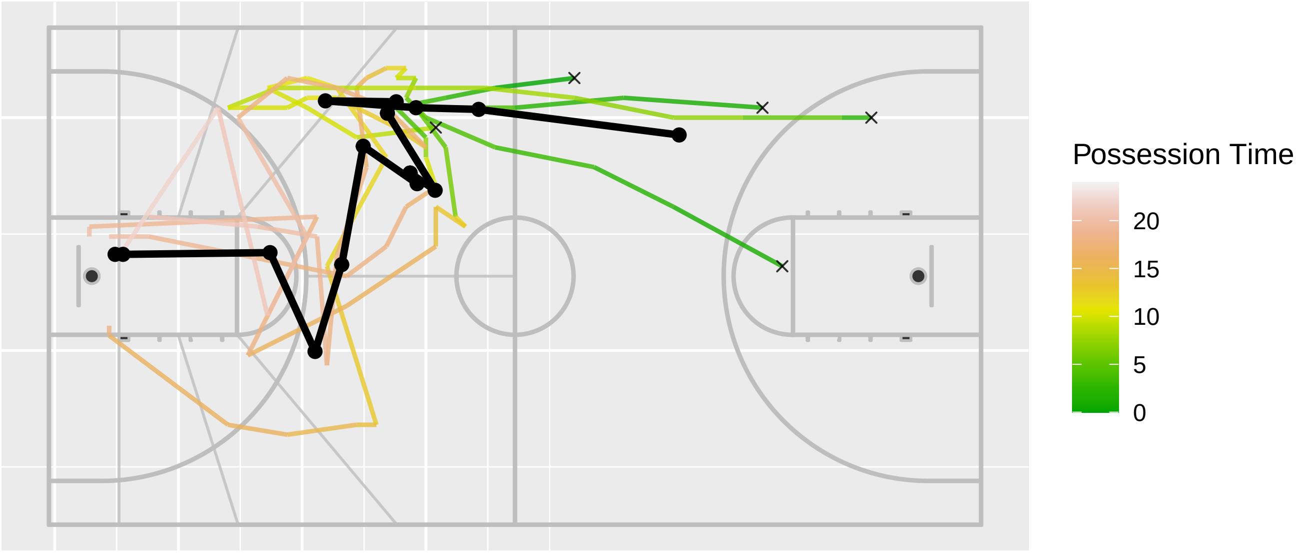

AUS Area 3 Cluster 33 : SelectTrajectories

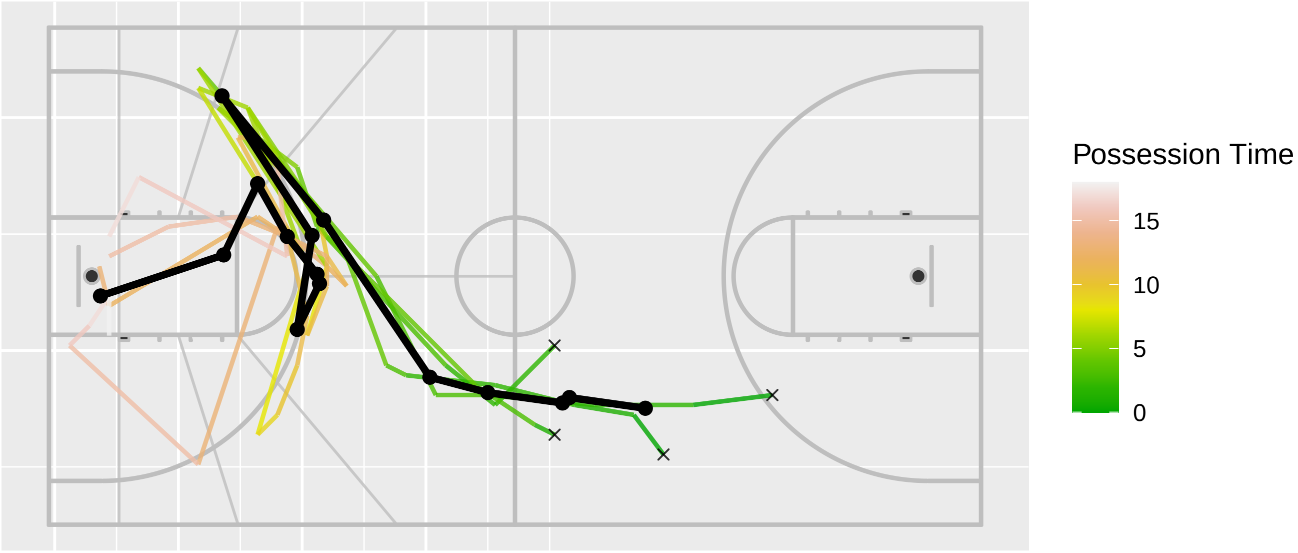

AUS Area 3 Cluster 34 : SelectTrajectories

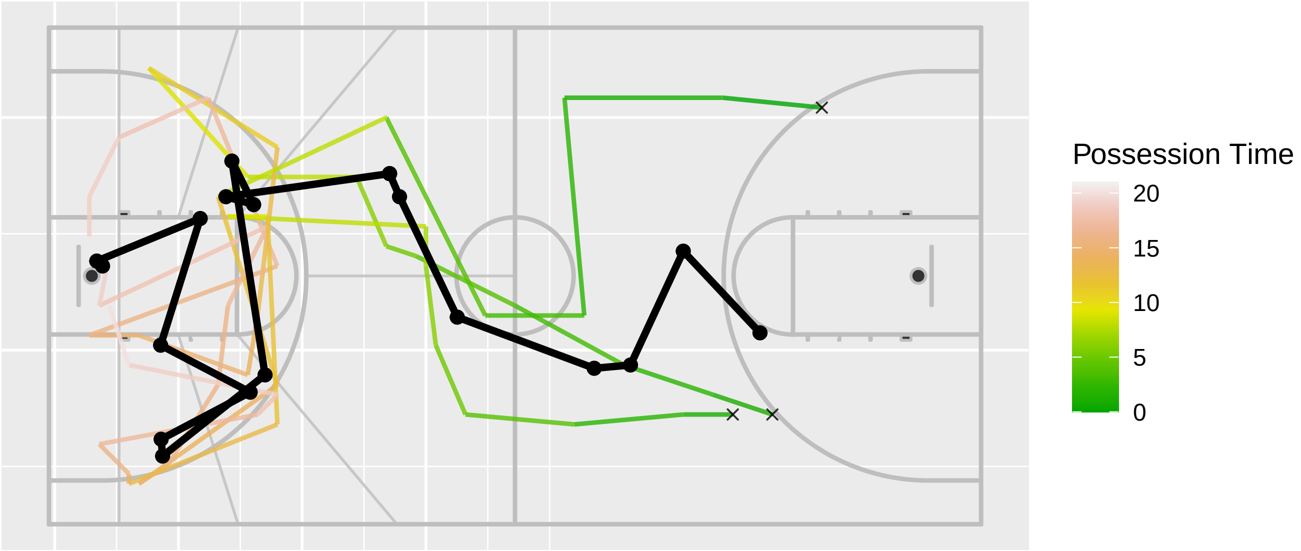

AUS Area 3 Cluster 35 : SelectTrajectories

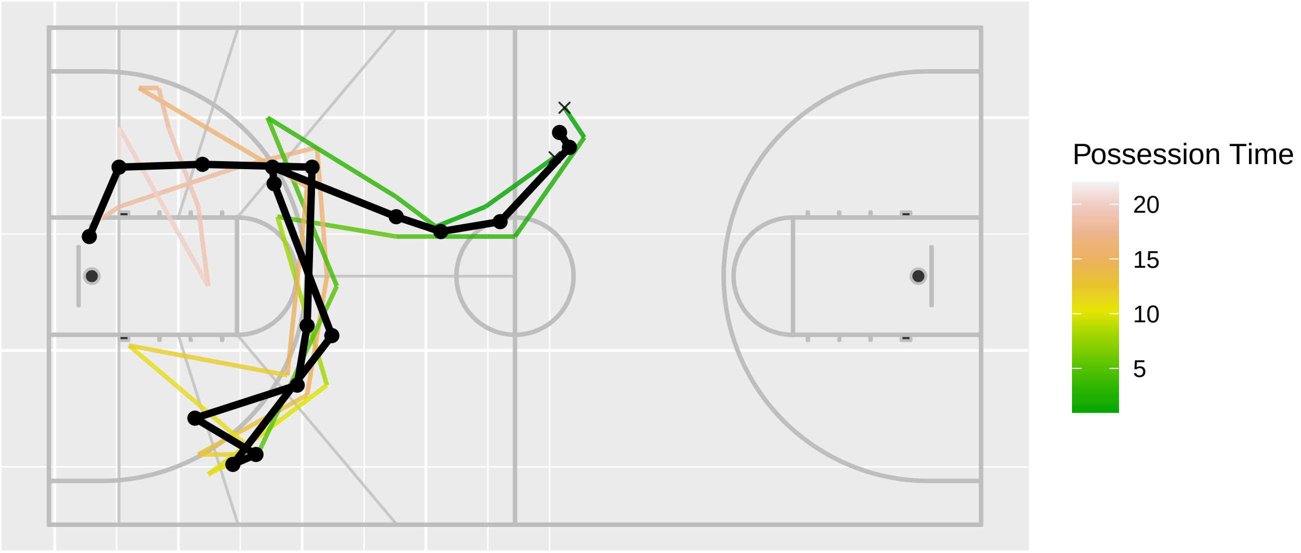

AUS Area 3 Cluster 36 : SelectTrajectories

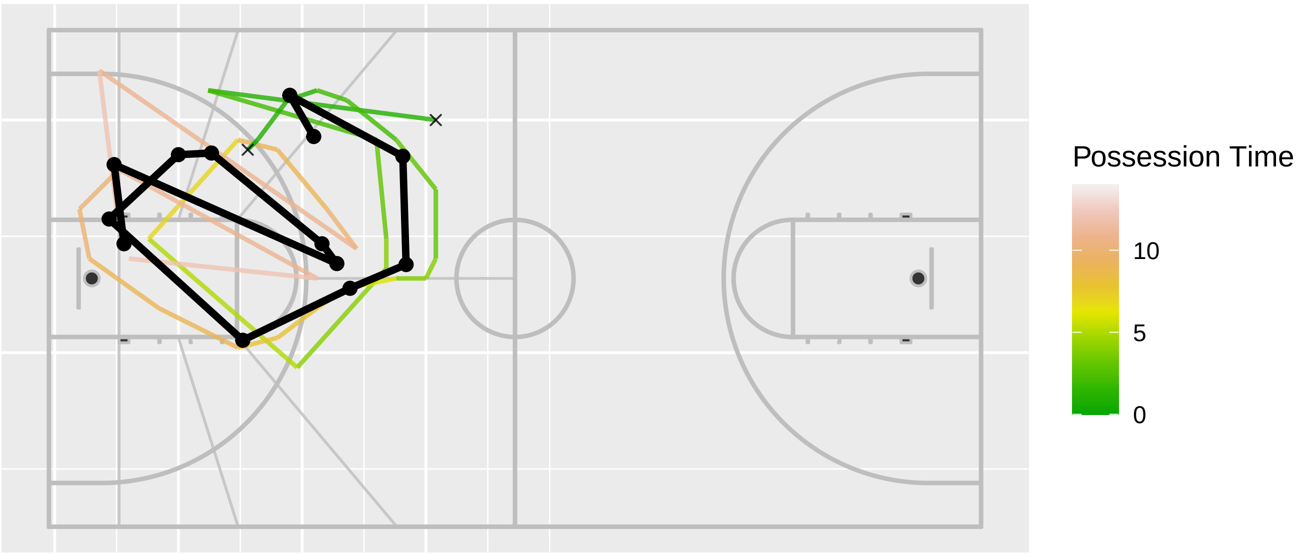

AUS Area 3 Cluster 37 : SelectTrajectories

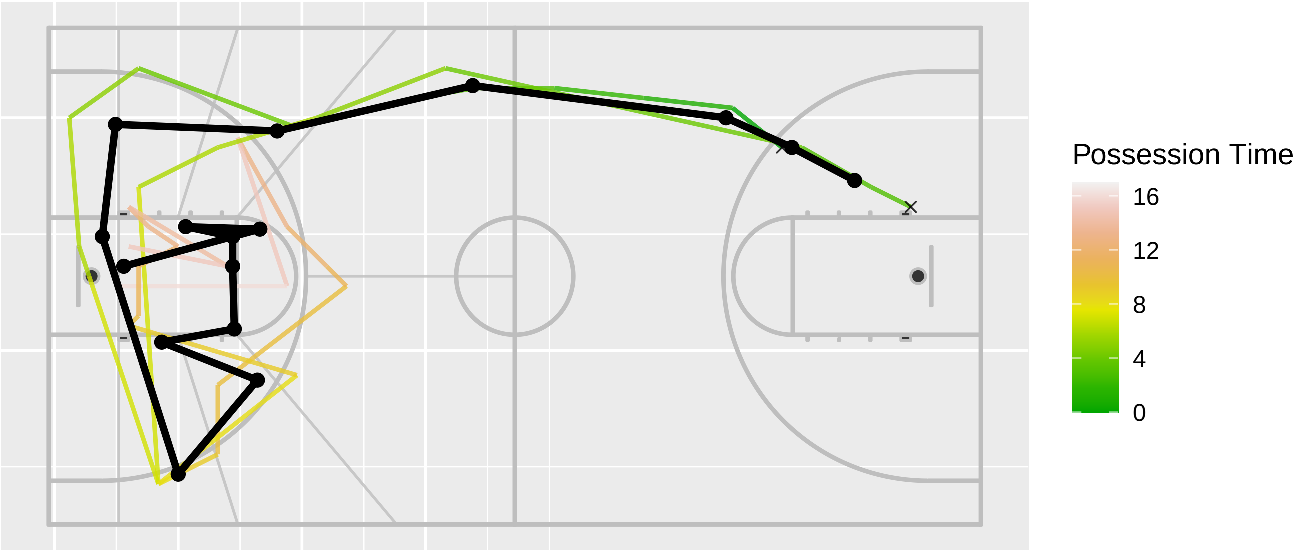

AUS Area 3 Cluster 38 : SelectTrajectories

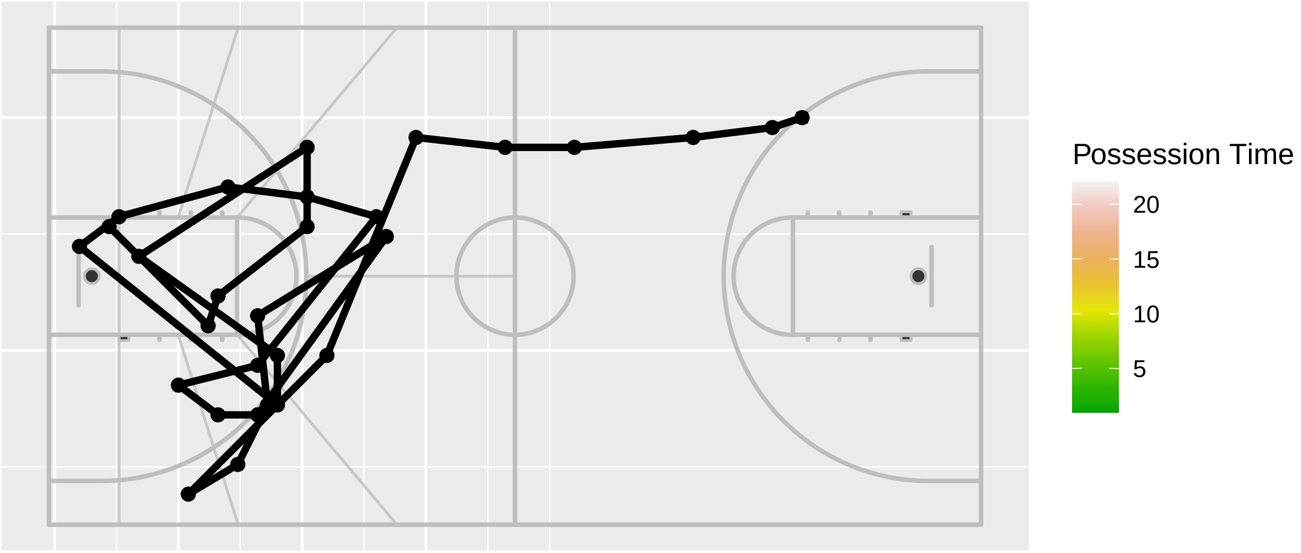

AUS Area 4 Cluster 1 : SelectTrajectories

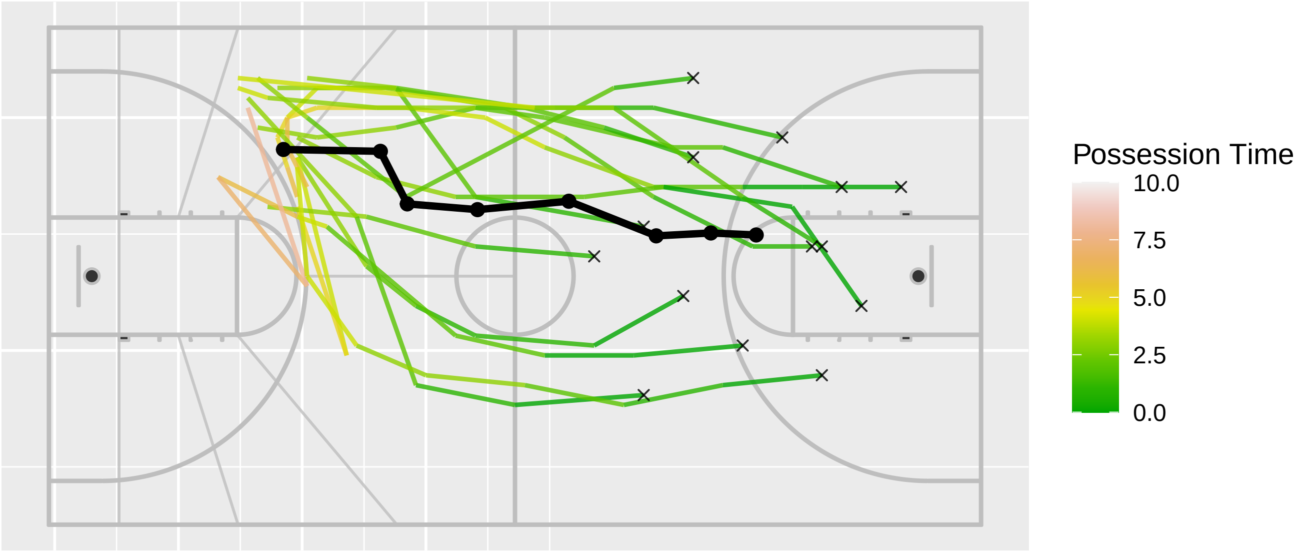

AUS Area 4 Cluster 2 : SelectTrajectories

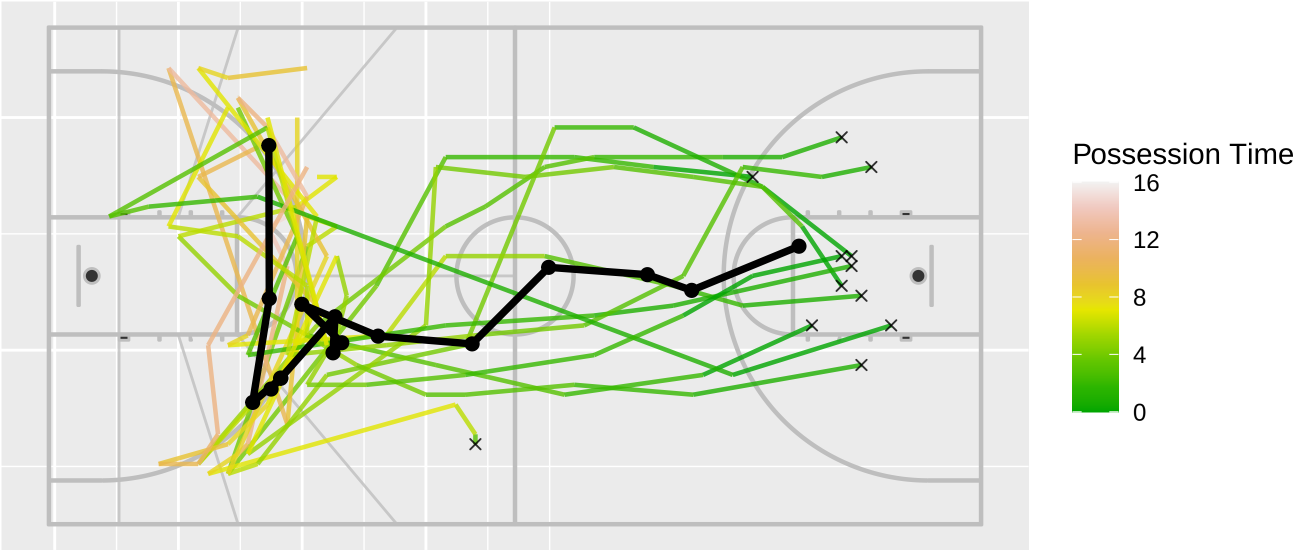

AUS Area 4 Cluster 3 : SelectTrajectories

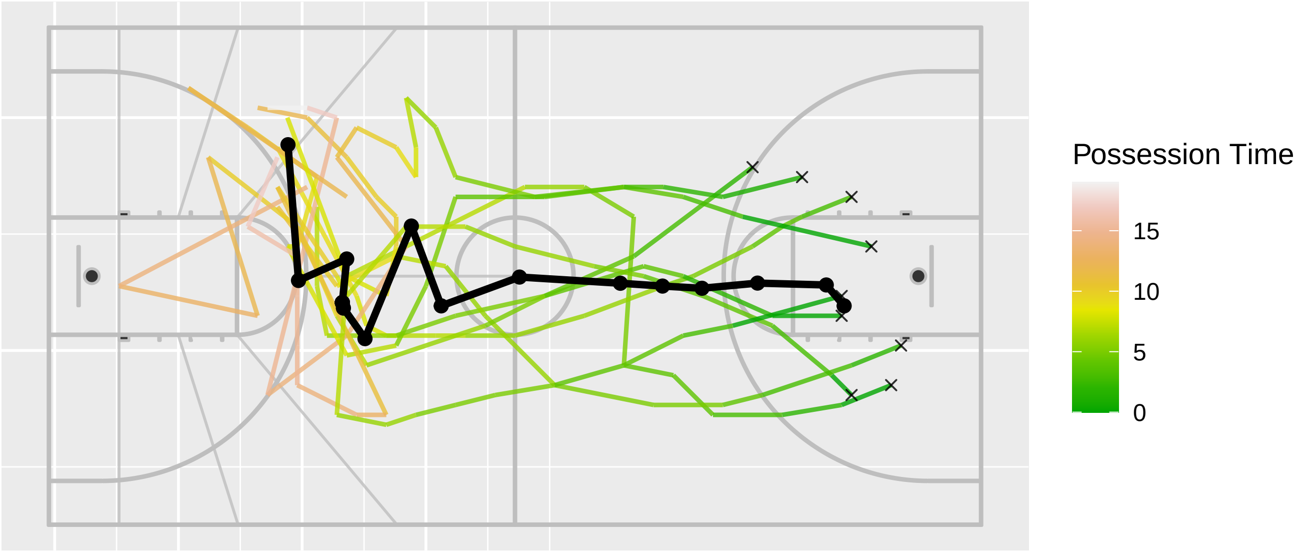

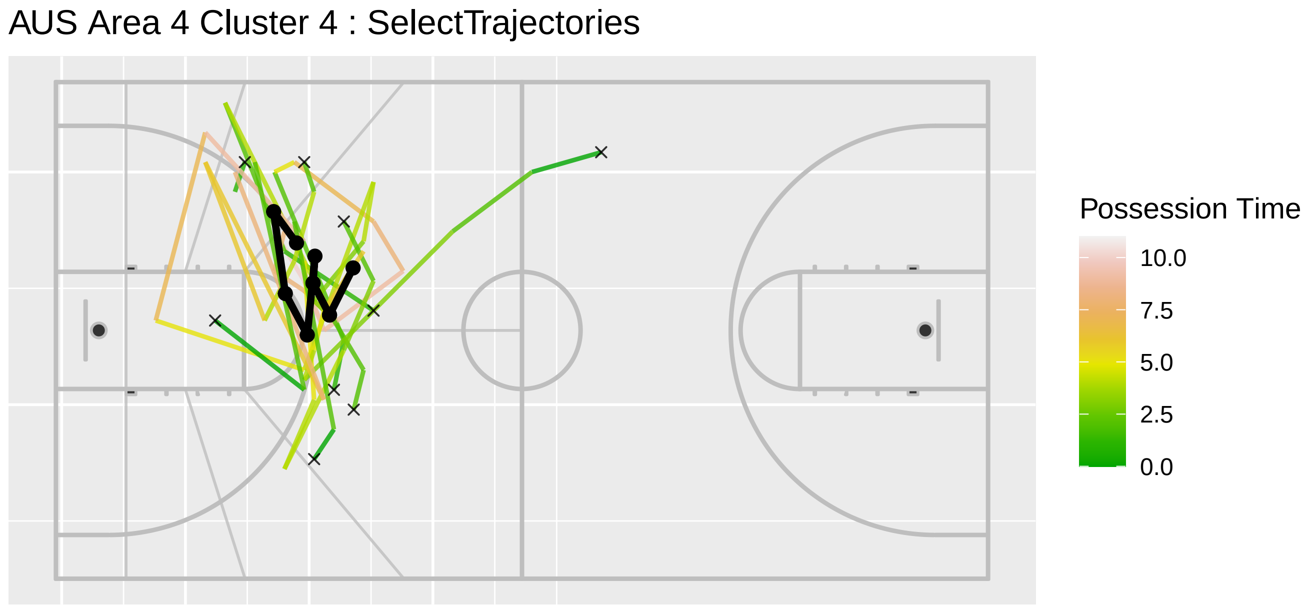
AUS Area 4 Cluster 4 : SelectTrajectories
Possession Time
10.0
7.5
5.0
2.5
0.0

AUS Area 4 Cluster 5 : SelectTrajectories

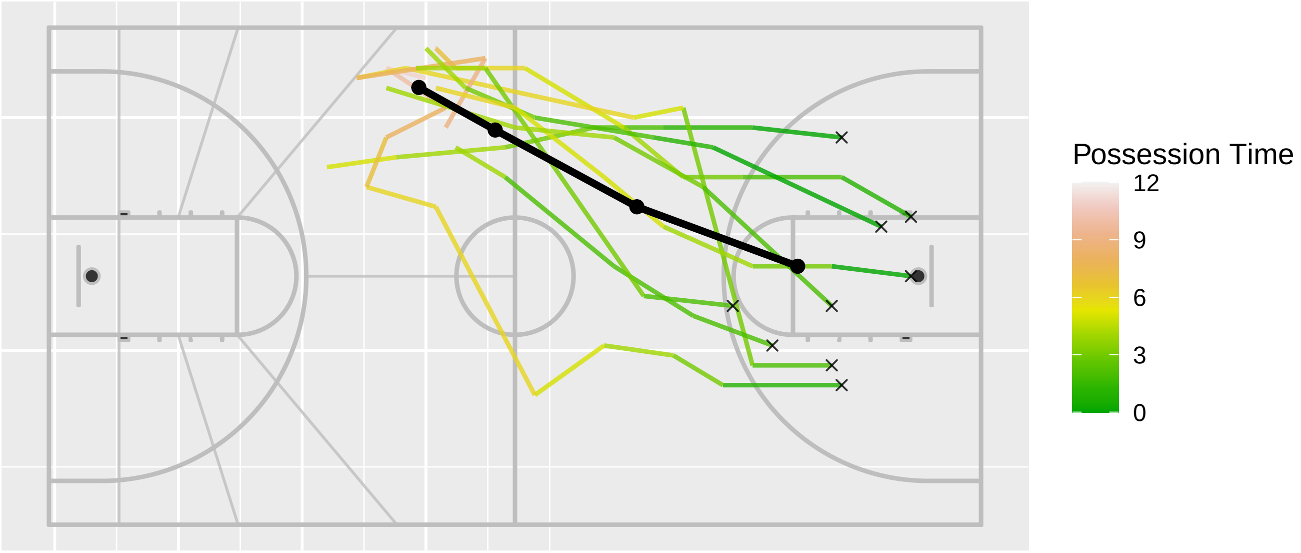

AUS Area 4 Cluster 6 : SelectTrajectories

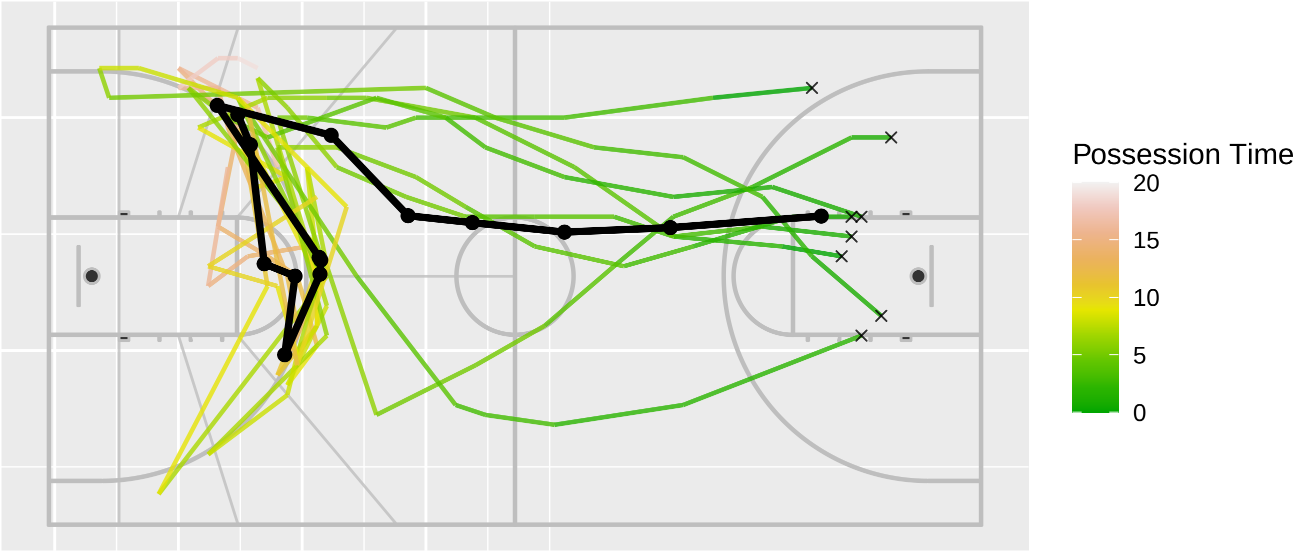

AUS Area 4 Cluster 7 : SelectTrajectories

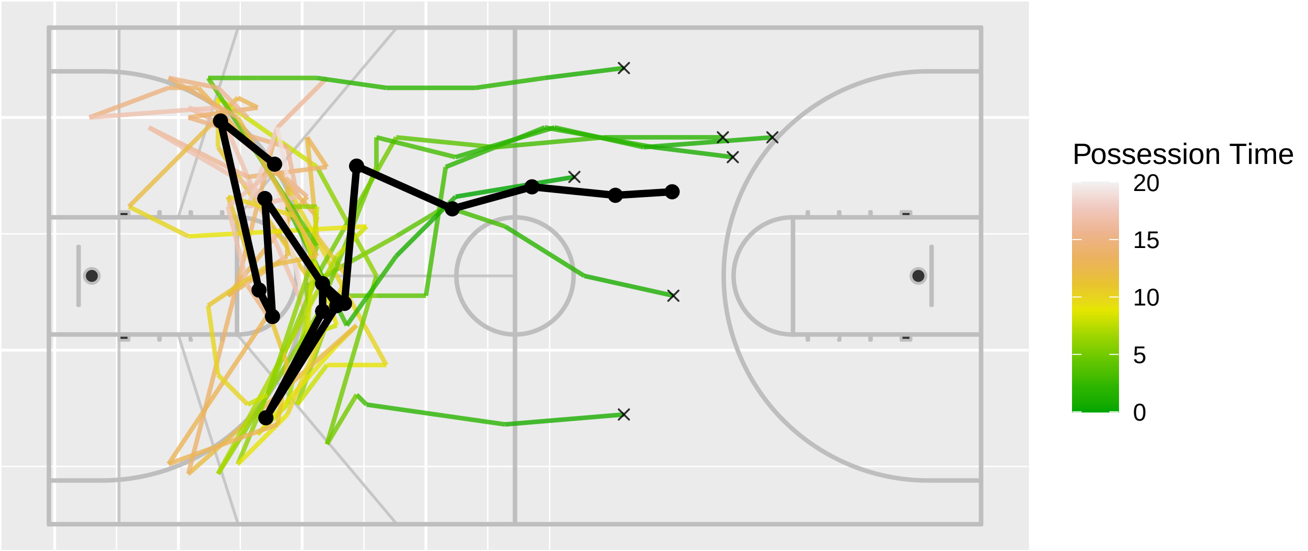

AUS Area 4 Cluster 8 : SelectTrajectories

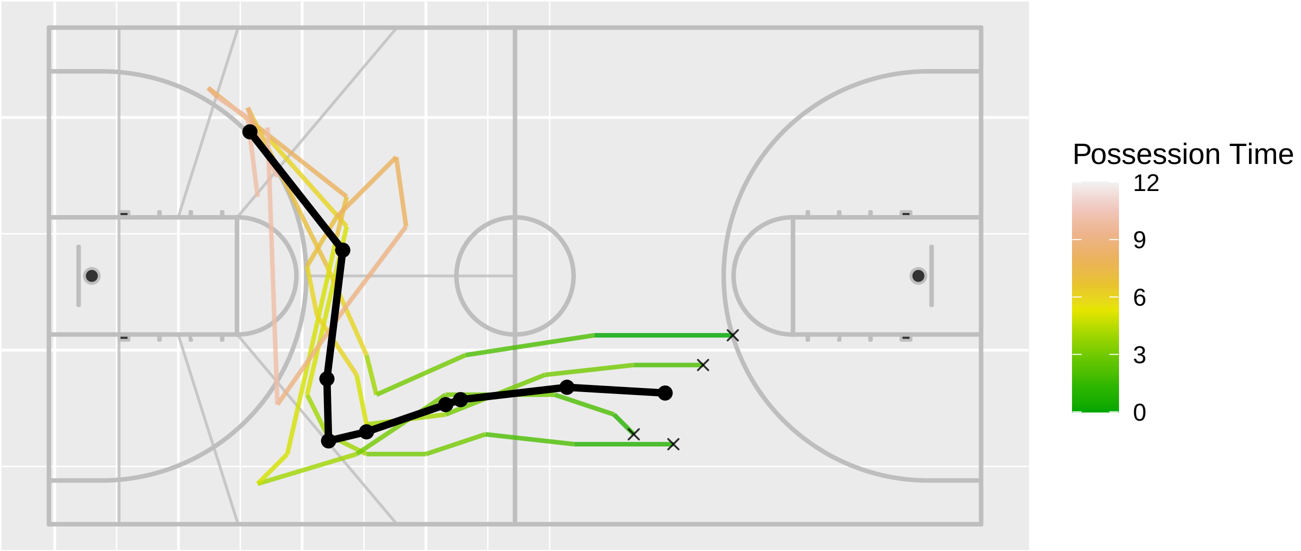

AUS Area 4 Cluster 9 : SelectTrajectories

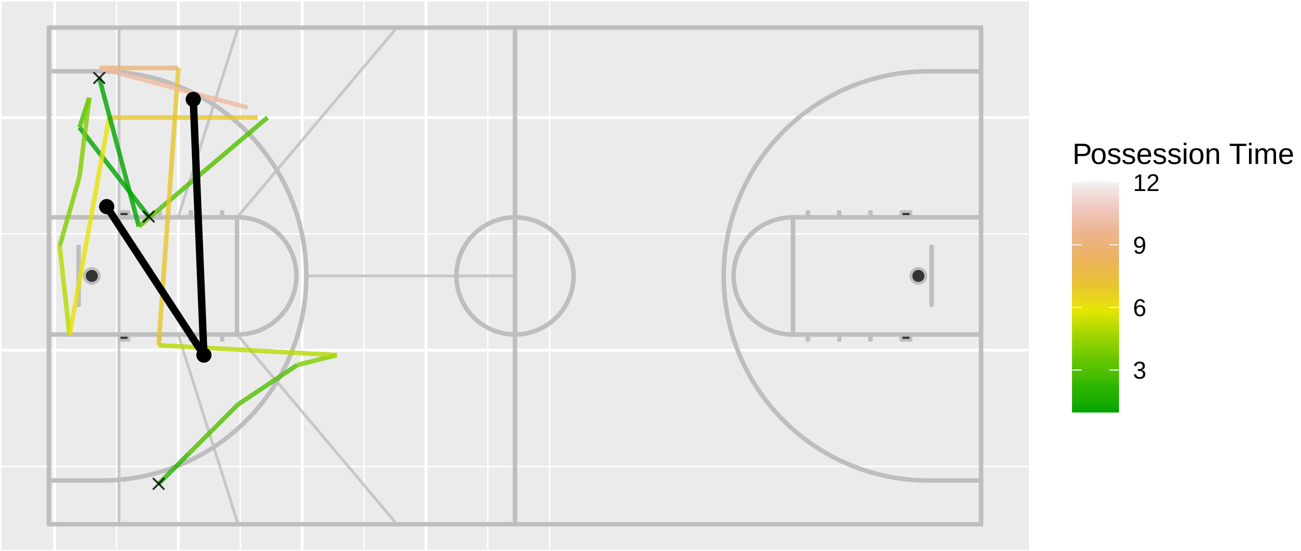

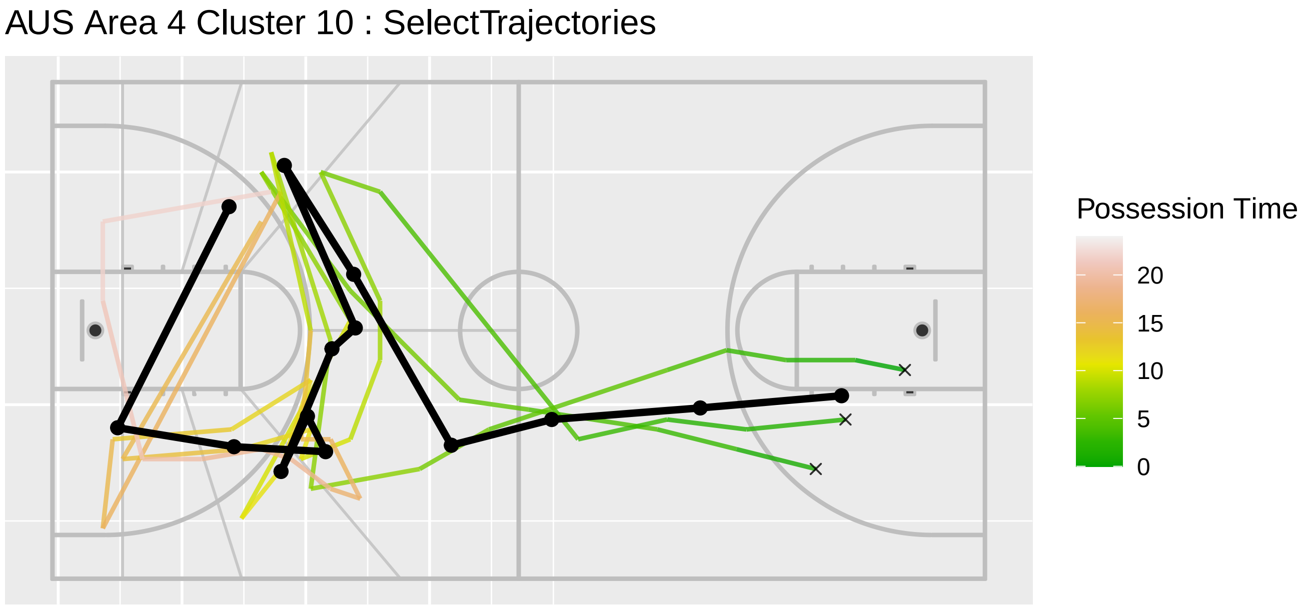
AUS Area 4 Cluster 10 : SelectTrajectories
Possession Time
20
15
10
5
0

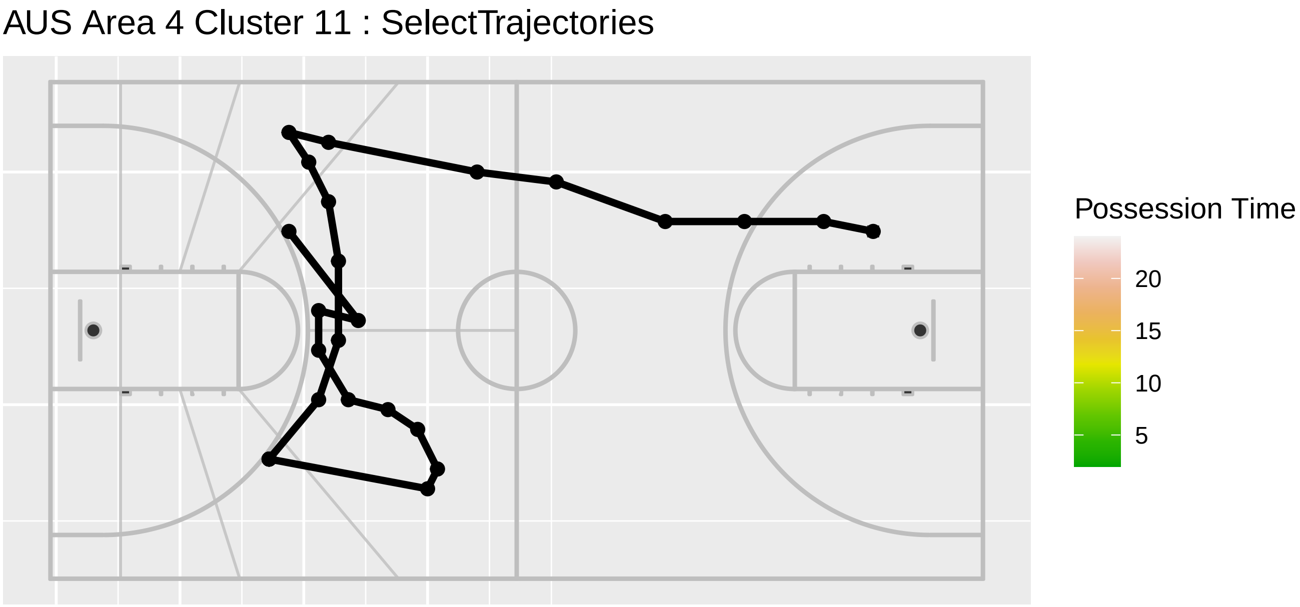
AUS Area 4 Cluster 11 : SelectTrajectories
Possession Time
20
15
10
5

AUS Area 5 Cluster 1 : SelectTrajectories

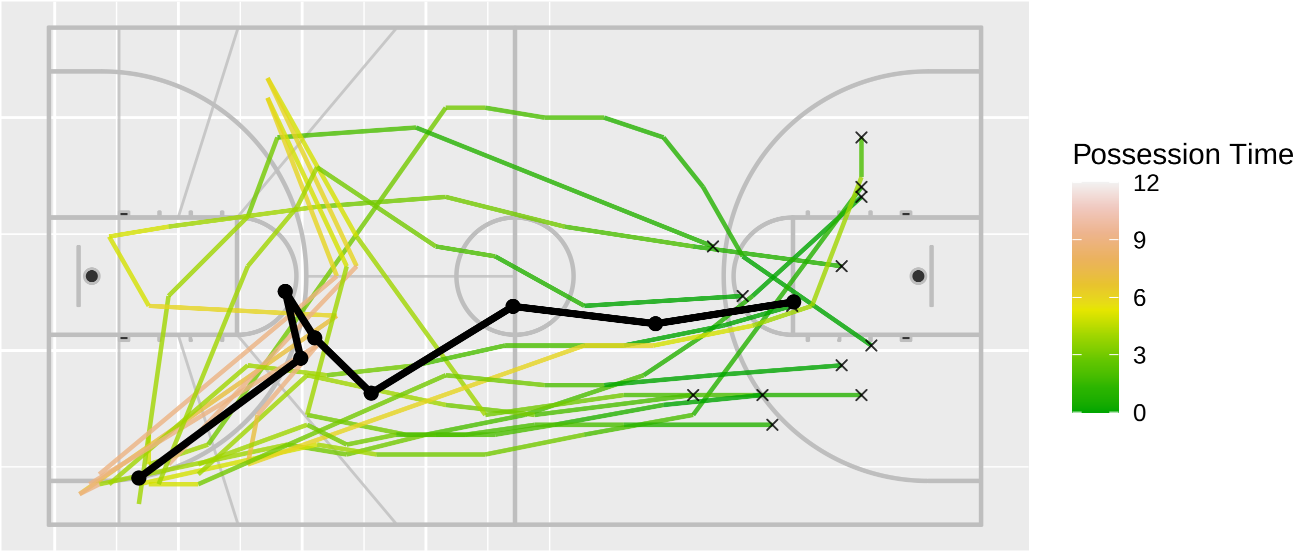

AUS Area 5 Cluster 2 : SelectTrajectories

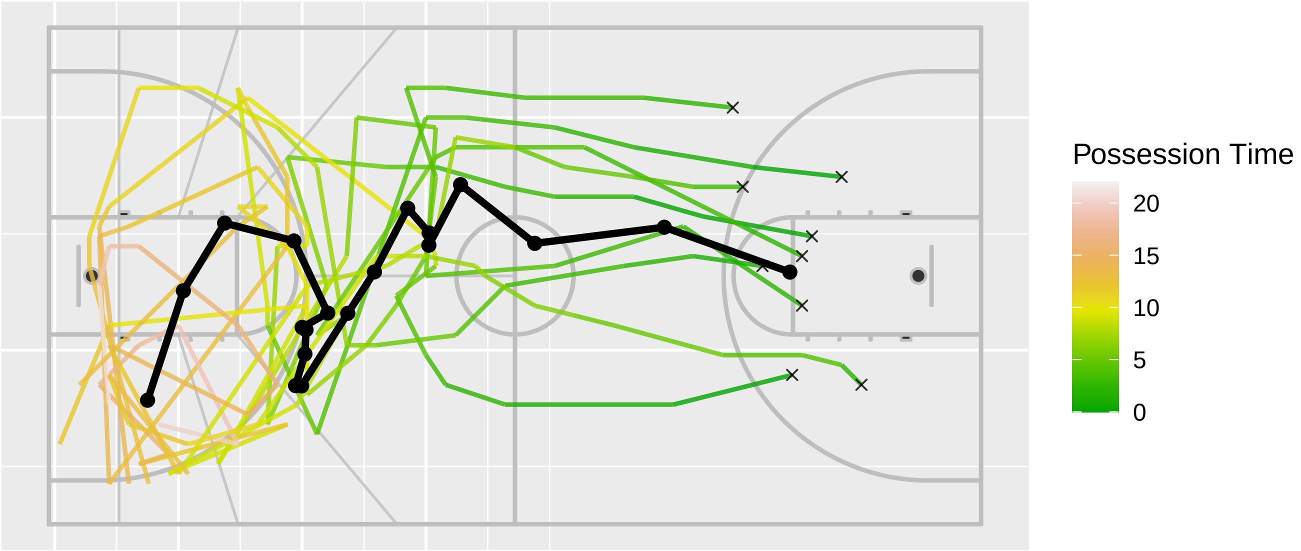

AUS Area 5 Cluster 3 : SelectTrajectories

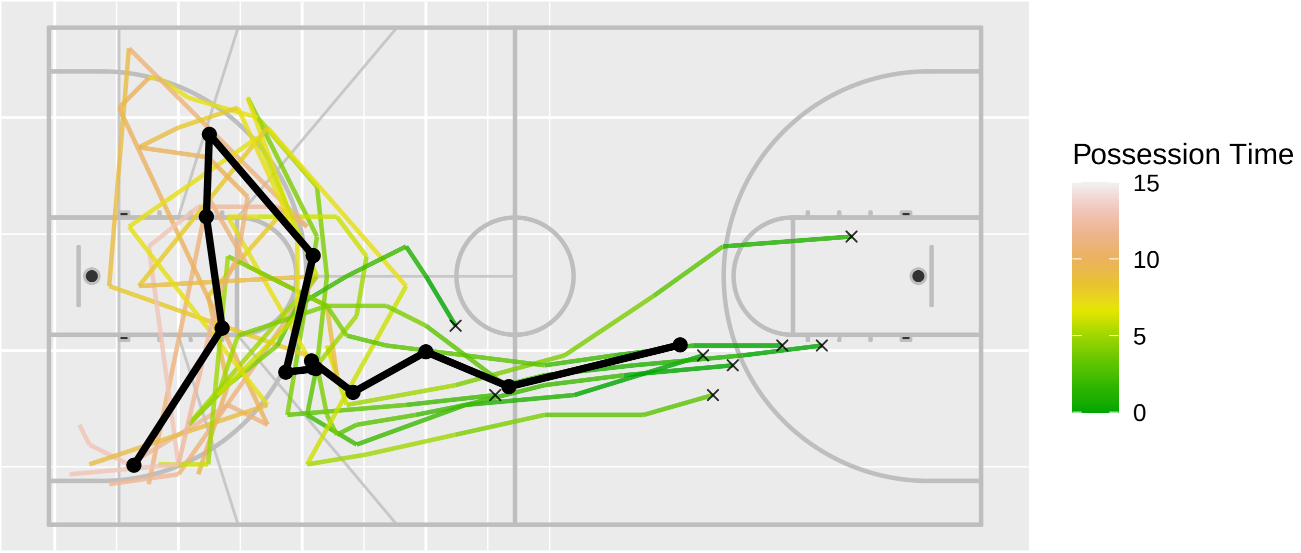

AUS Area 5 Cluster 4 : SelectTrajectories

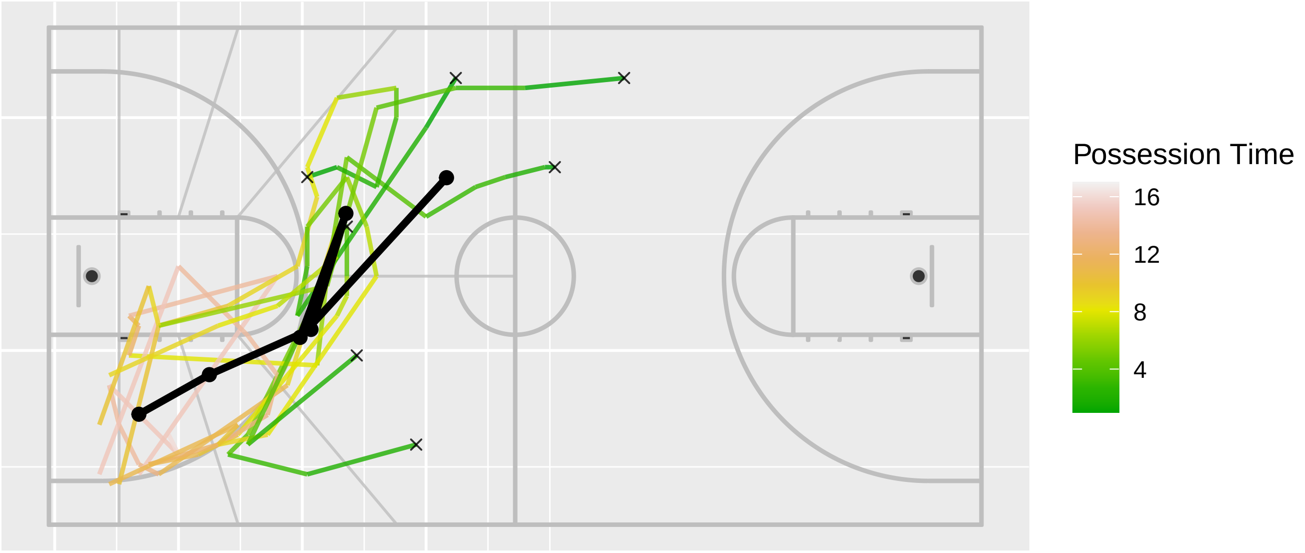

AUS Area 5 Cluster 5 : SelectTrajectories

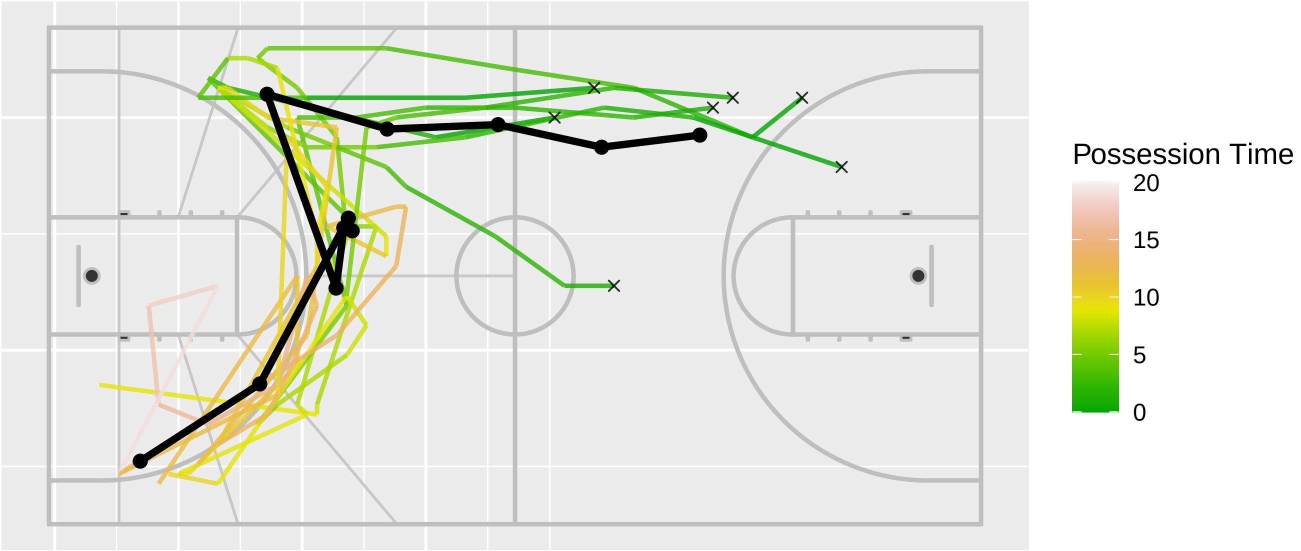

AUS Area 5 Cluster 6 : SelectTrajectories

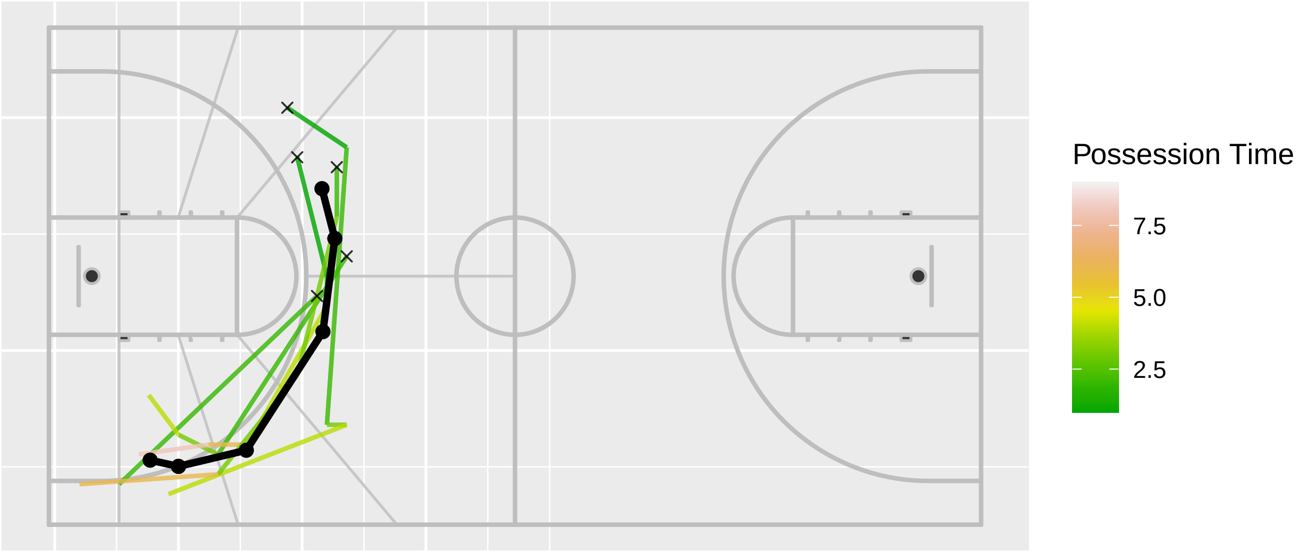

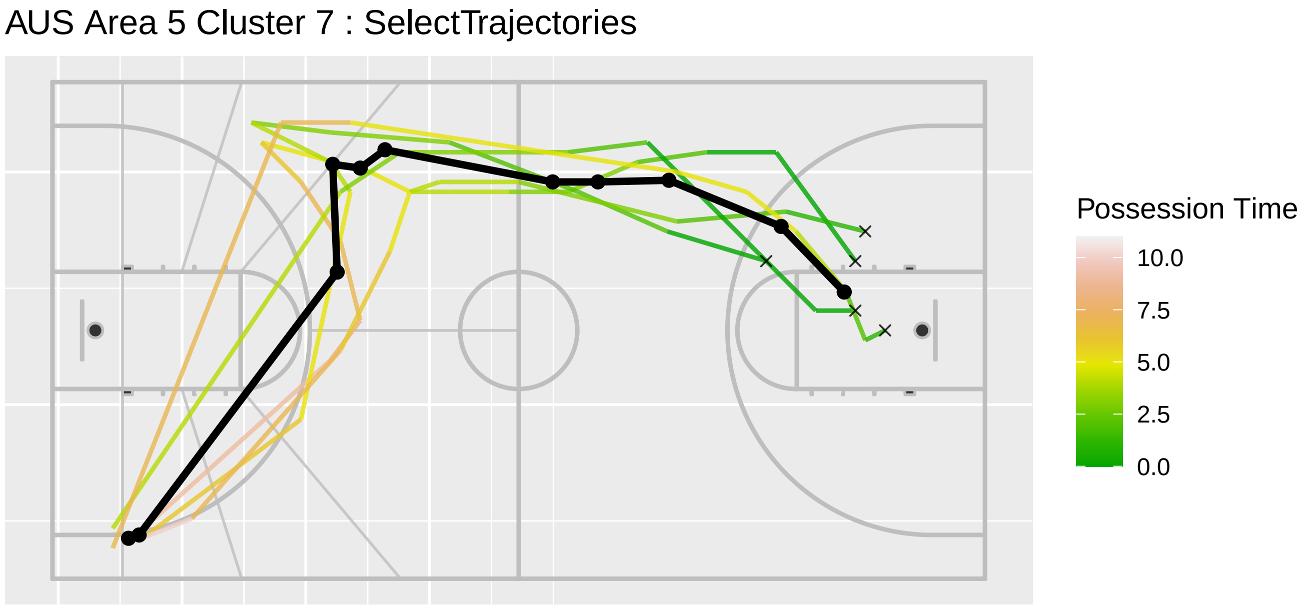
AUS Area 5 Cluster 7 : SelectTrajectories
Possession Time
10.0
7.5
5.0
2.5
0.0

AUS Area 5 Cluster 8 : SelectTrajectories

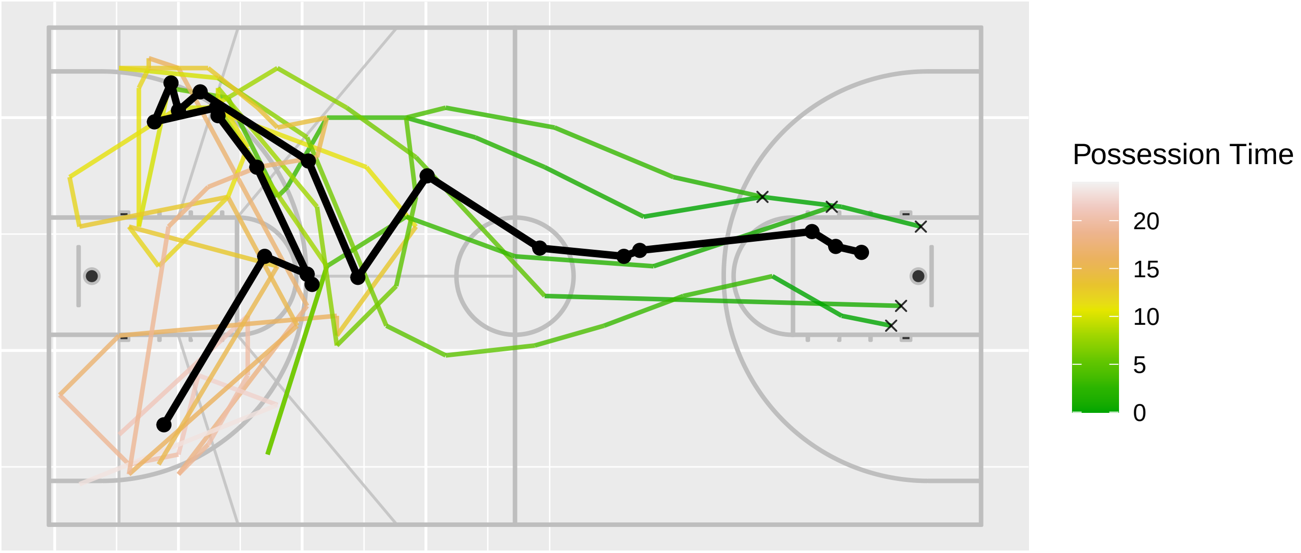

AUS Area 5 Cluster 9 : SelectTrajectories

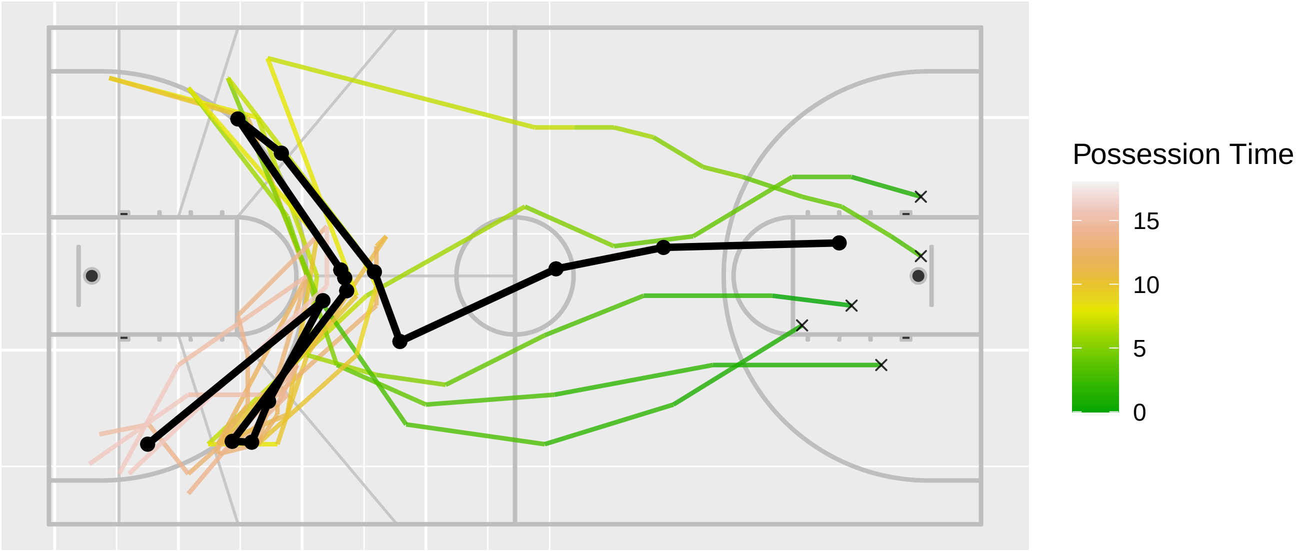

AUS Area 5 Cluster 10 : SelectTrajectories

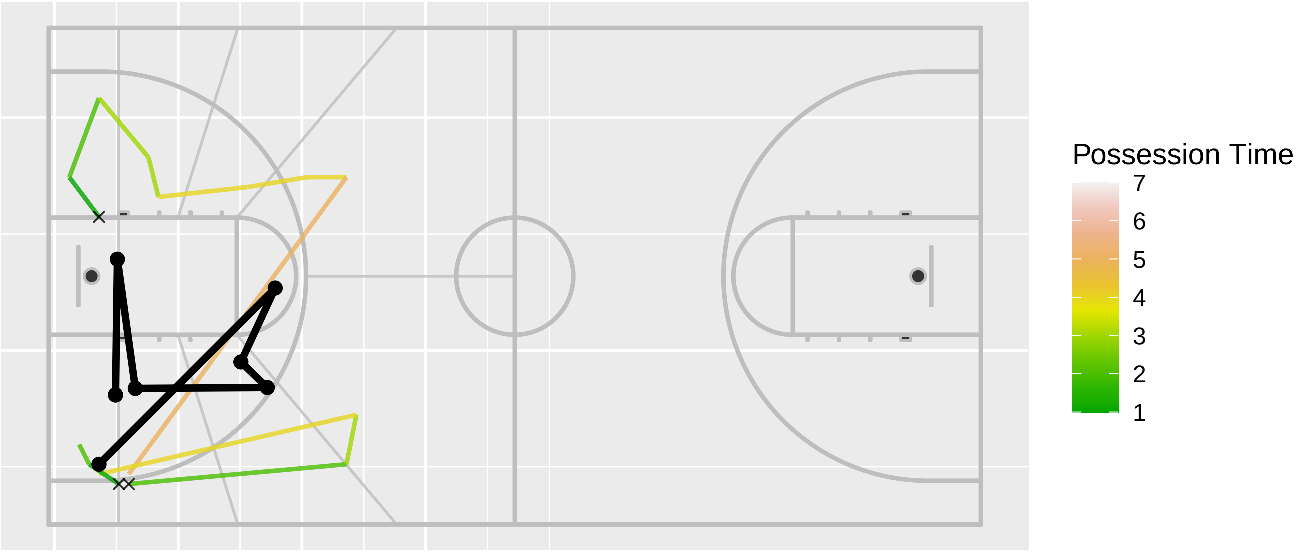

AUS Area 5 Cluster 11 : SelectTrajectories

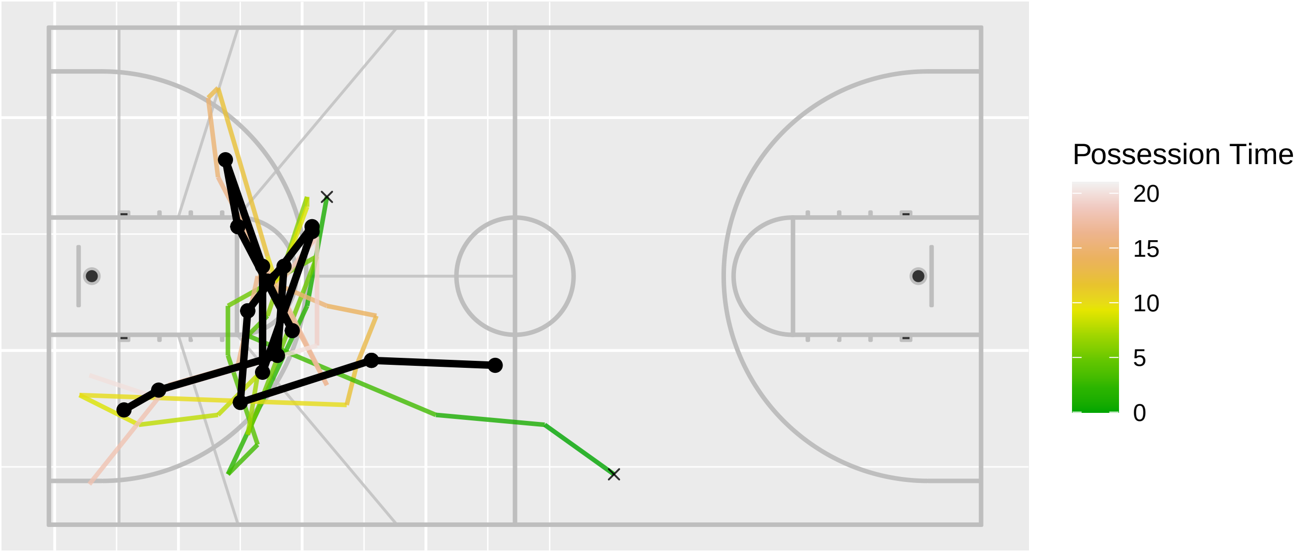

AUS Area 6 Cluster 1 : SelectTrajectories

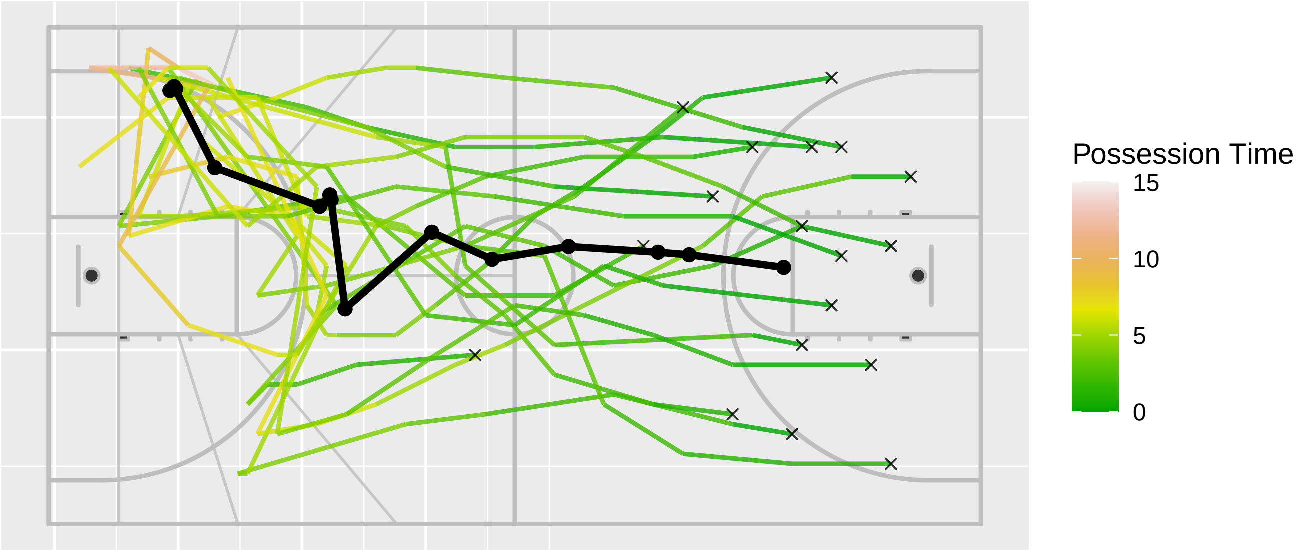

AUS Area 6 Cluster 2 : SelectTrajectories

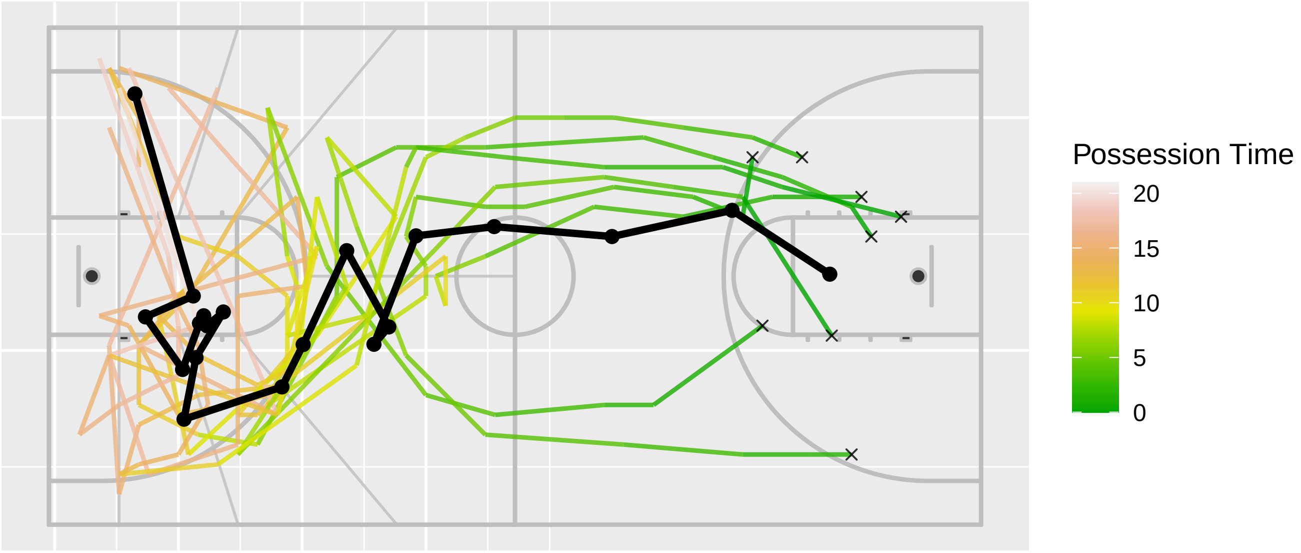

AUS Area 6 Cluster 3 : SelectTrajectories

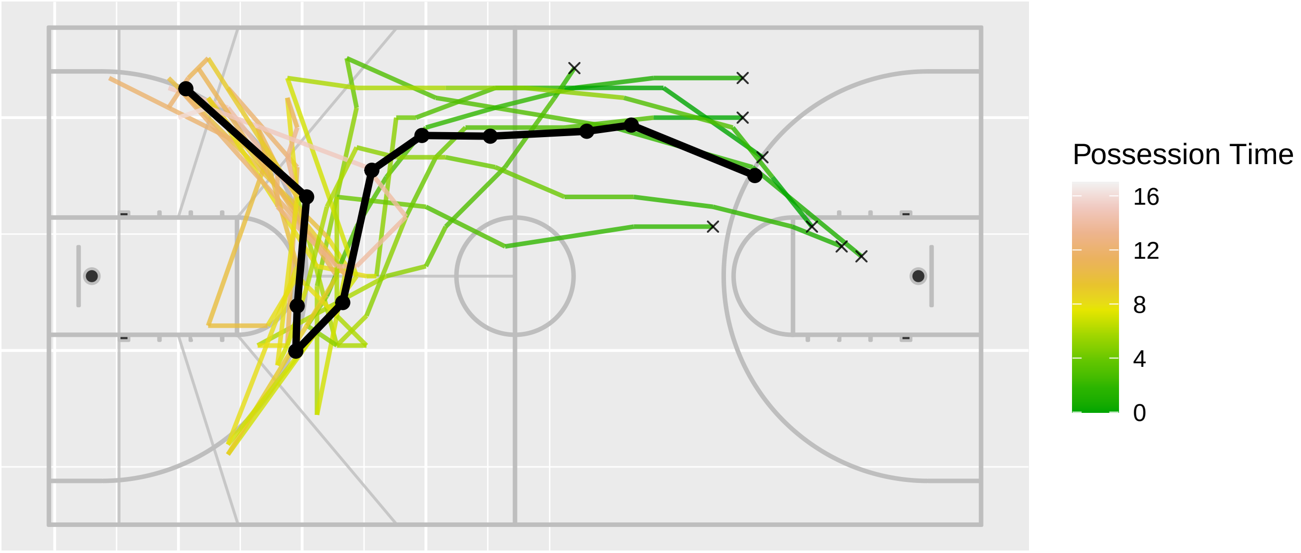

AUS Area 6 Cluster 4 : SelectTrajectories

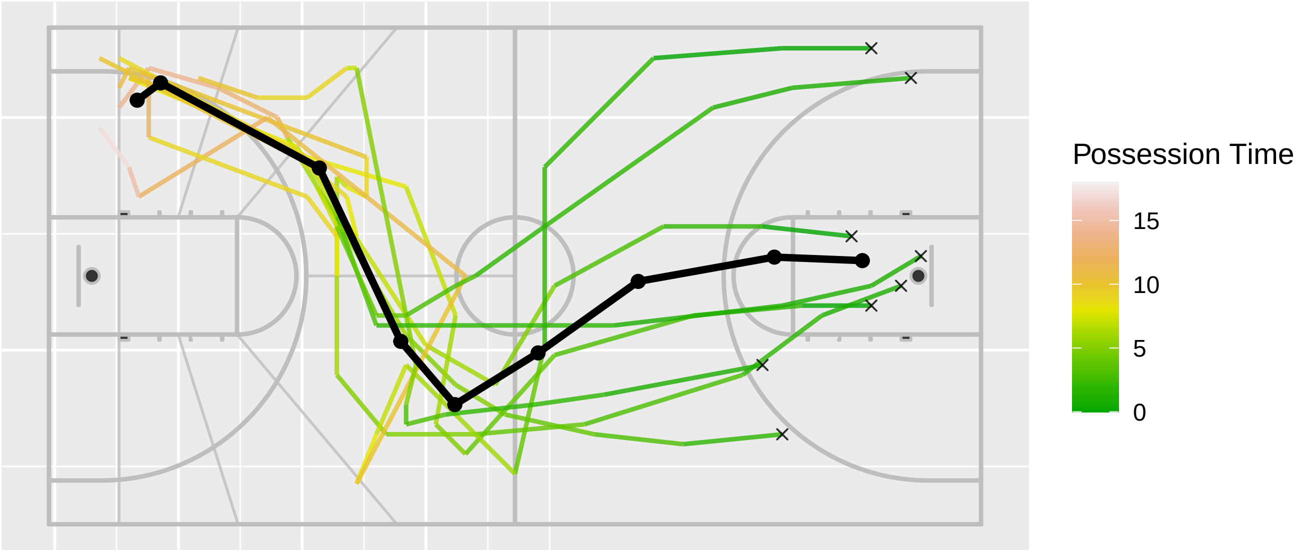

AUS Area 6 Cluster 5 : SelectTrajectories

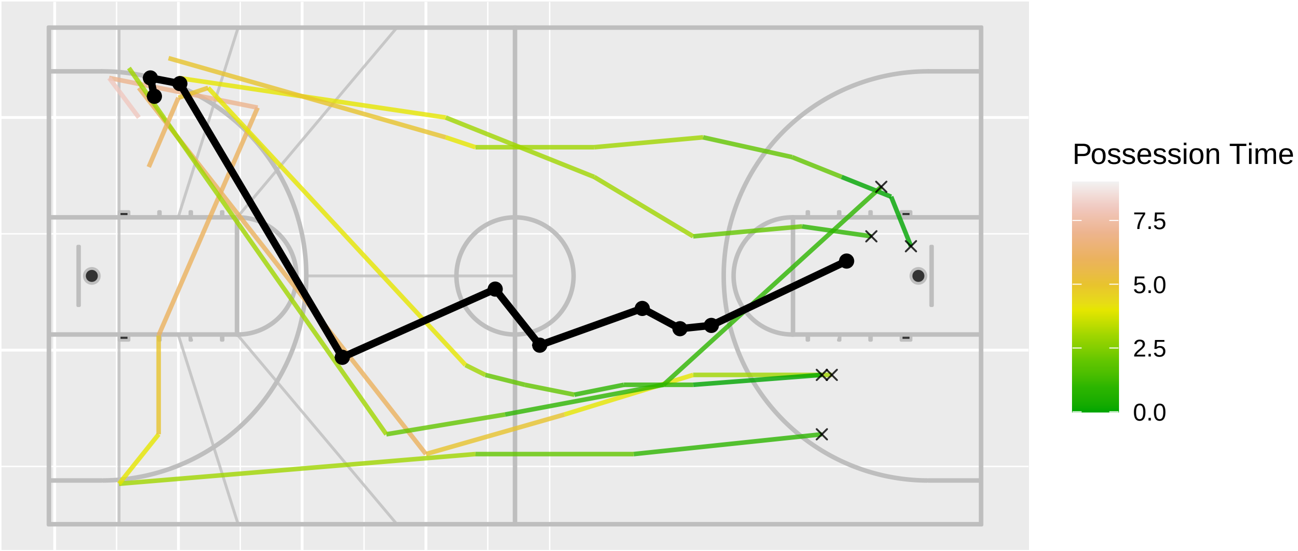

AUS Area 6 Cluster 6 : SelectTrajectories

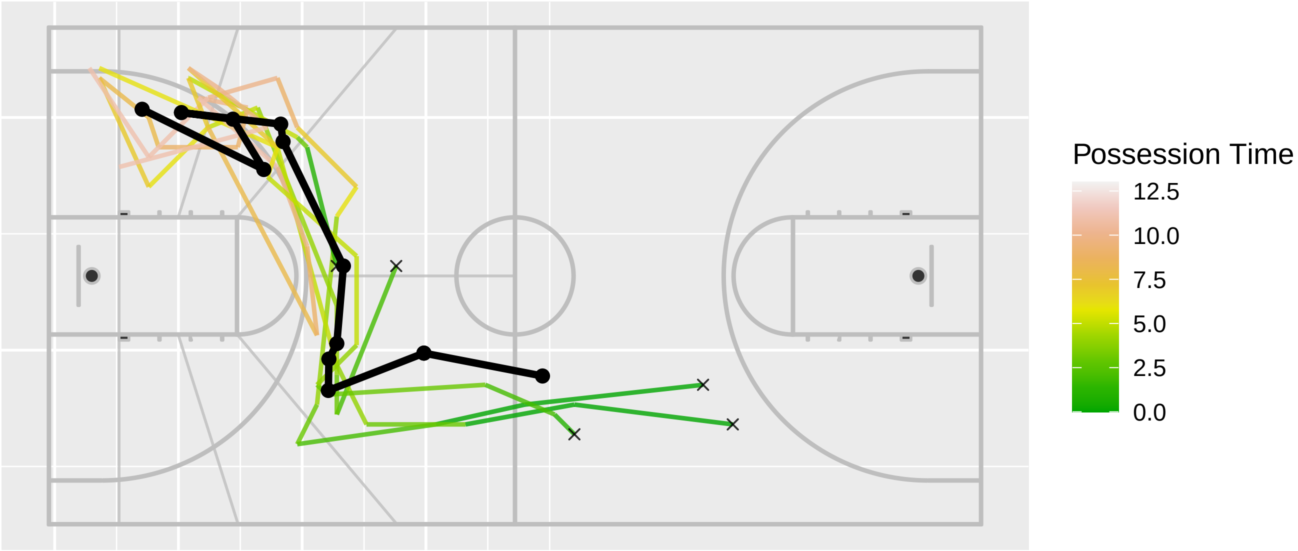

AUS Area 6 Cluster 7 : SelectTrajectories

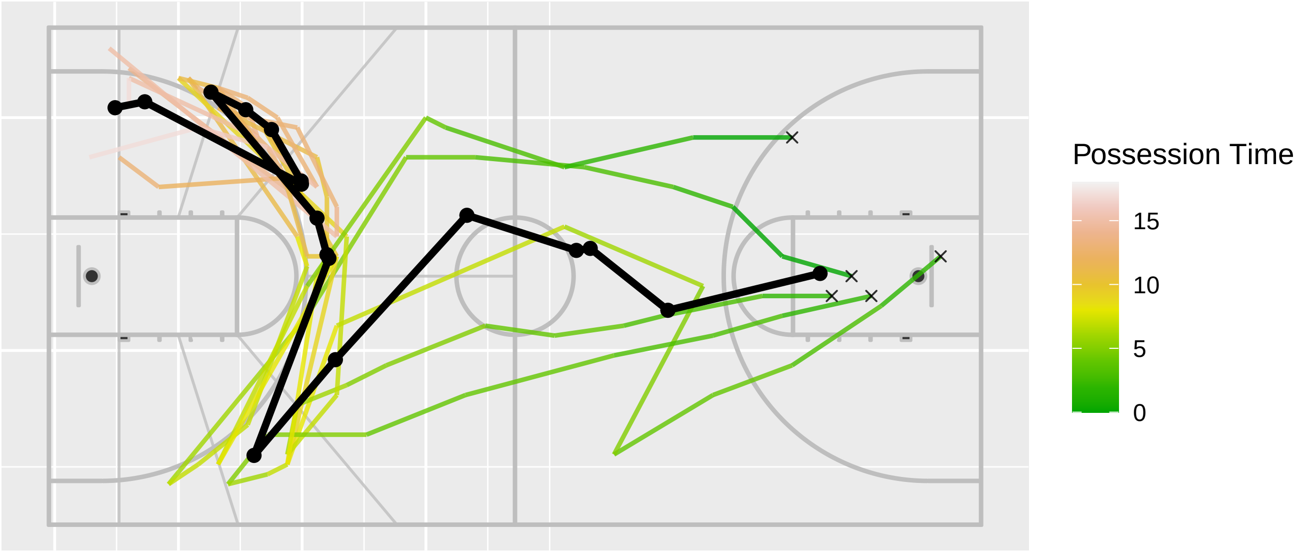

AUS Area 6 Cluster 8 : SelectTrajectories

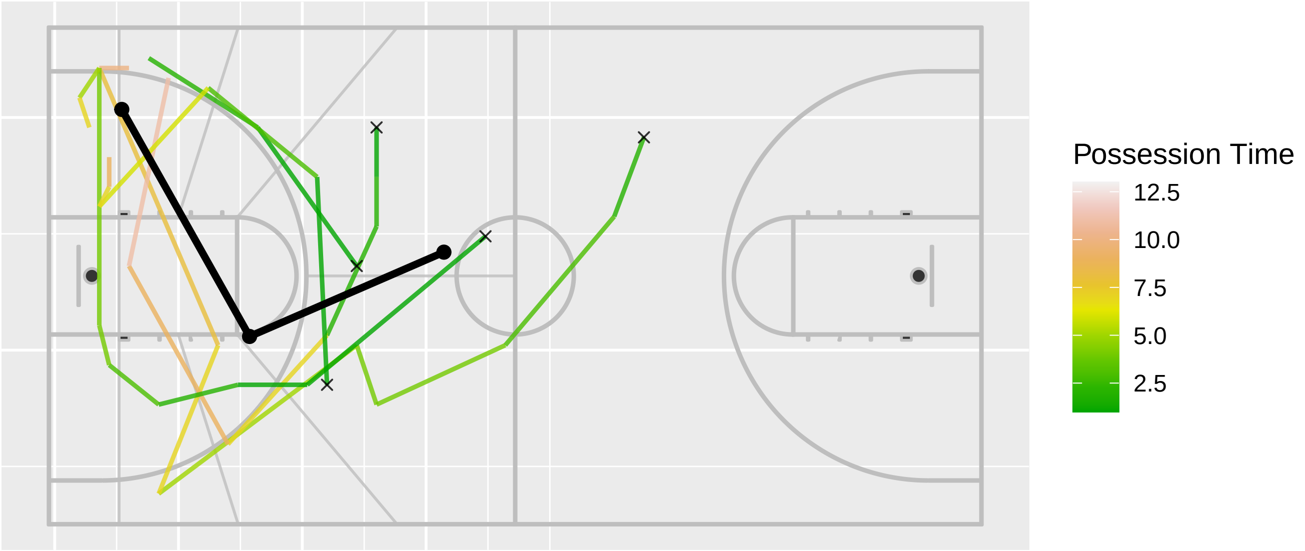

AUS Area 6 Cluster 9 : SelectTrajectories

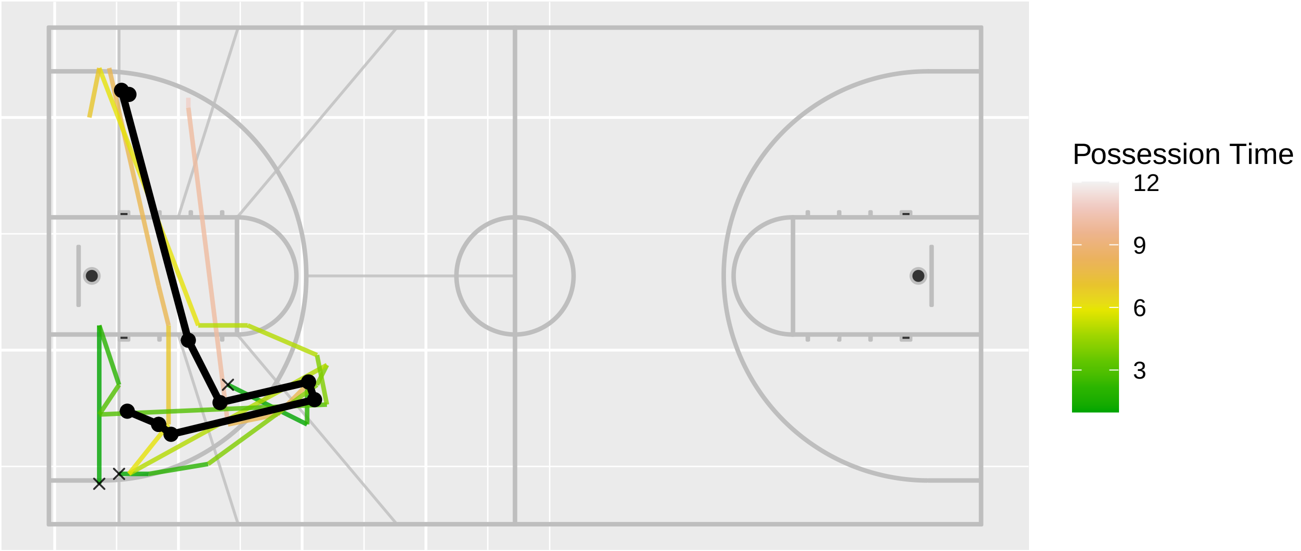

AUS Area 6 Cluster 10 : SelectTrajectories

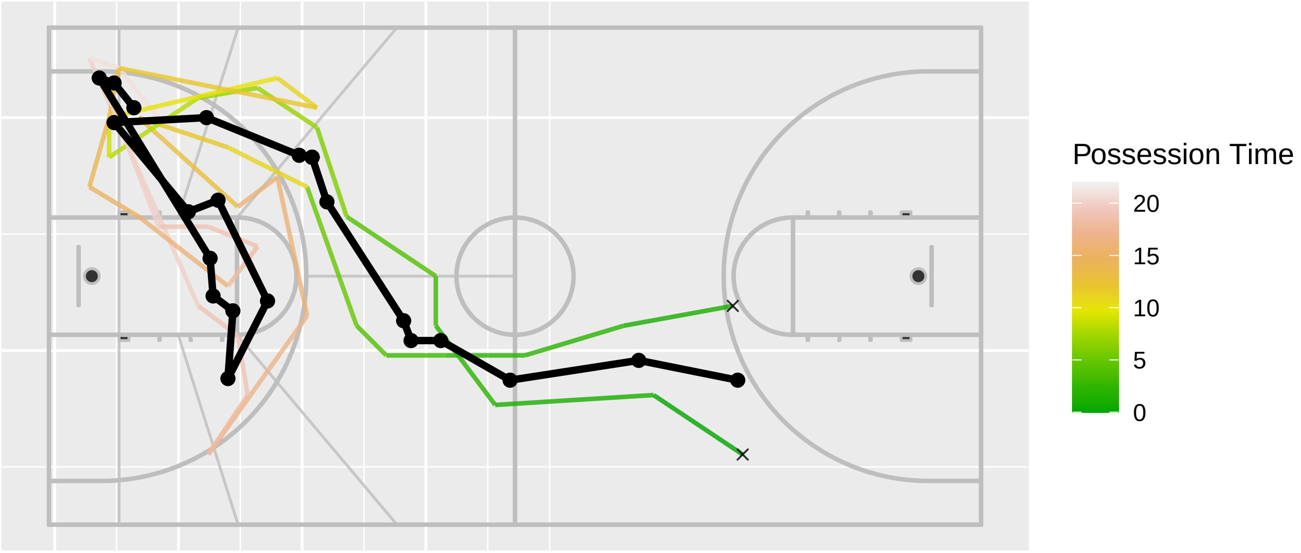

AUS Area 6 Cluster 11 : SelectTrajectories

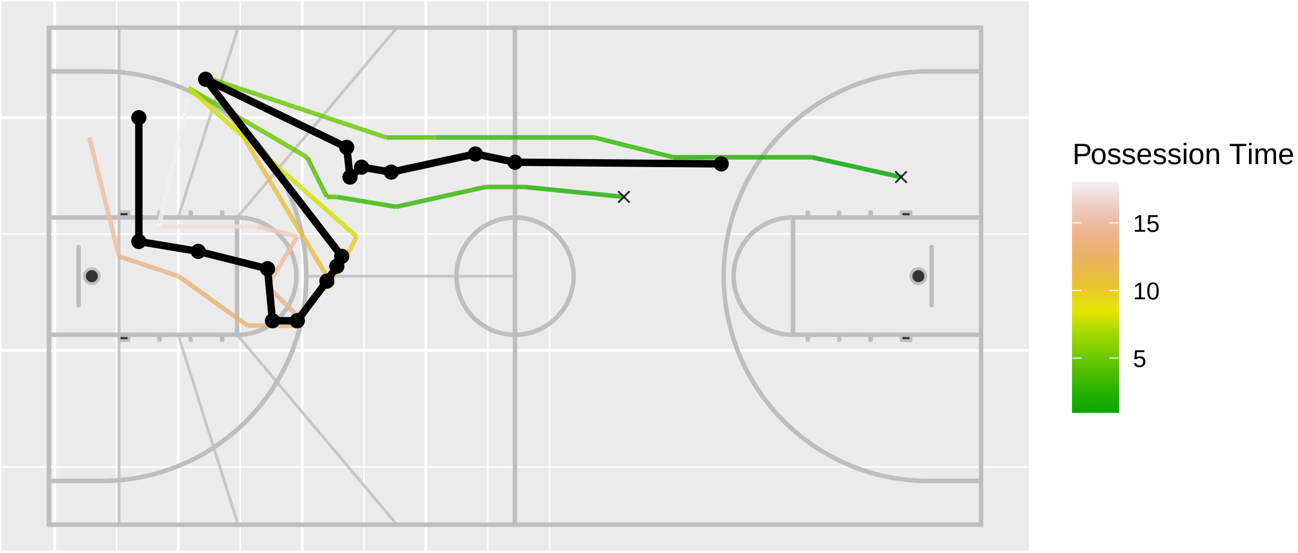

AUS Area 6 Cluster 12 : SelectTrajectories

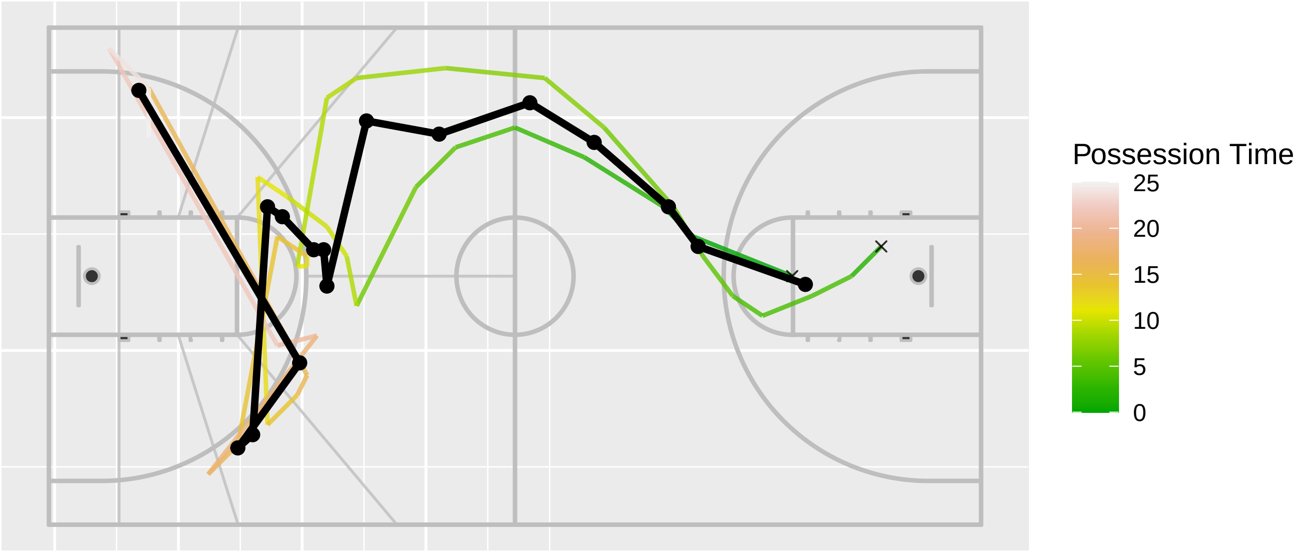

AUS Area 6 Cluster 13 : SelectTrajectories

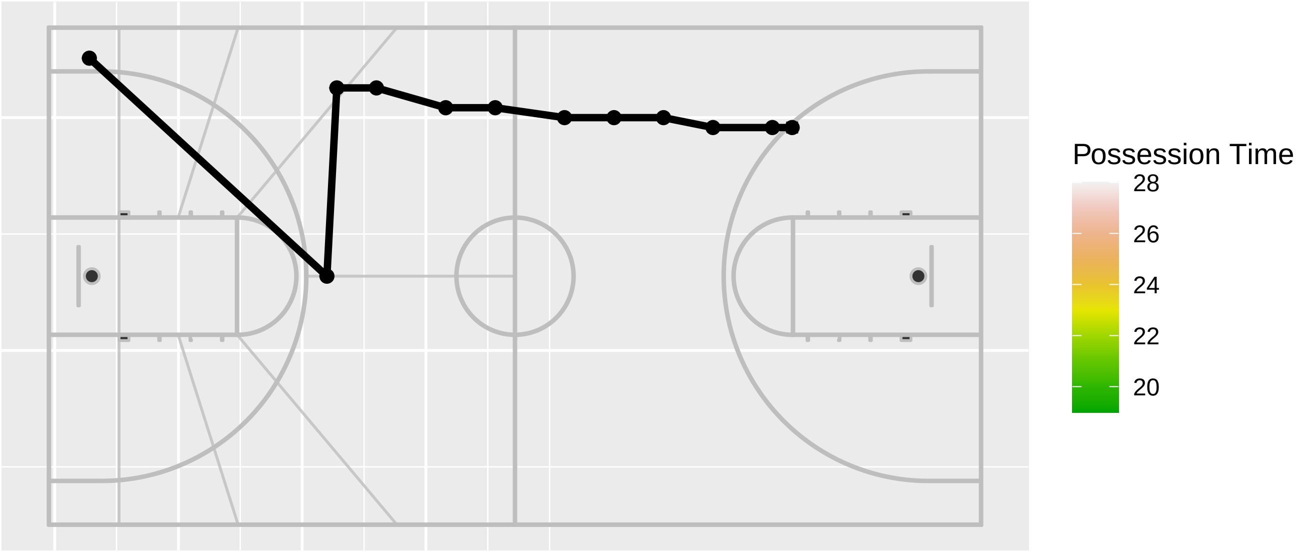

AUS Area 6 Cluster 14 : SelectTrajectories

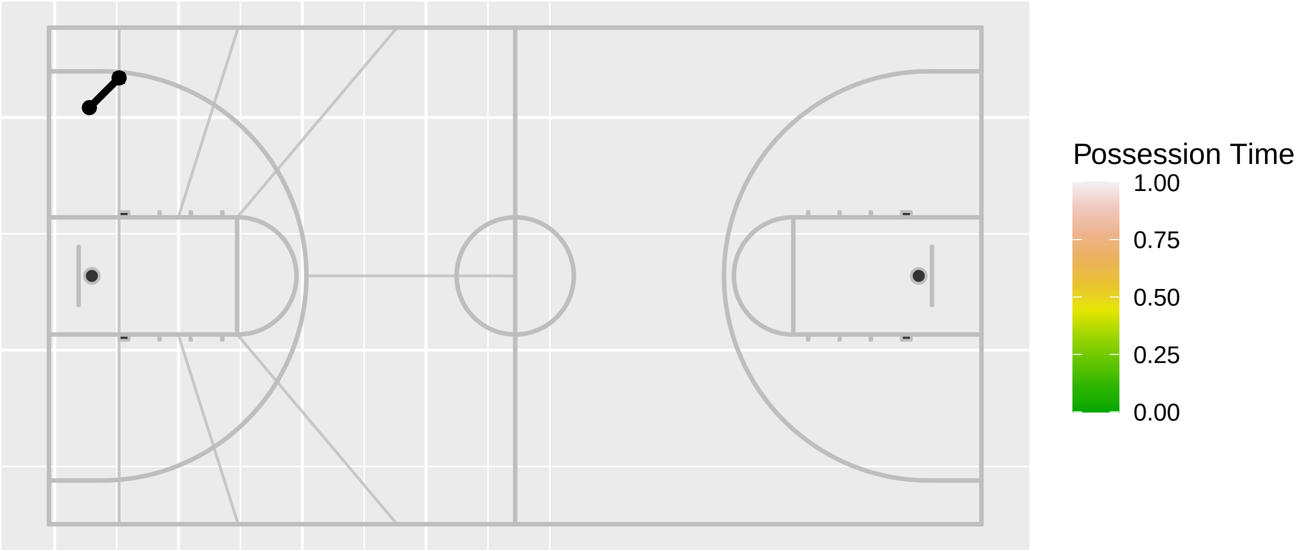

AUS Area 7 Cluster 1 : SelectTrajectories

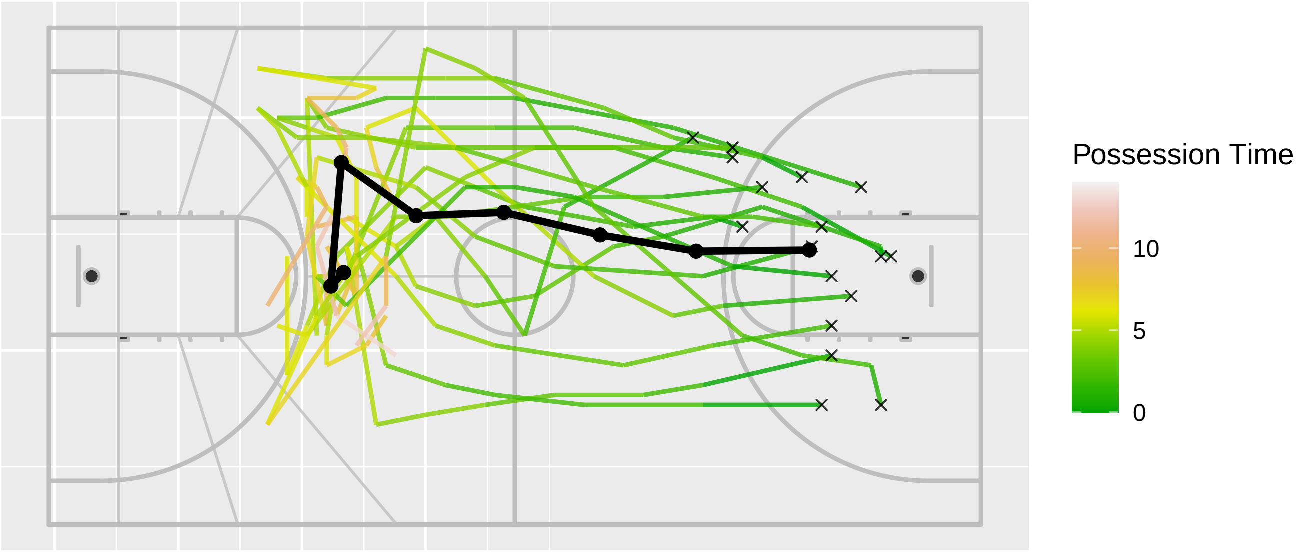

AUS Area 7 Cluster 2 : SelectTrajectories

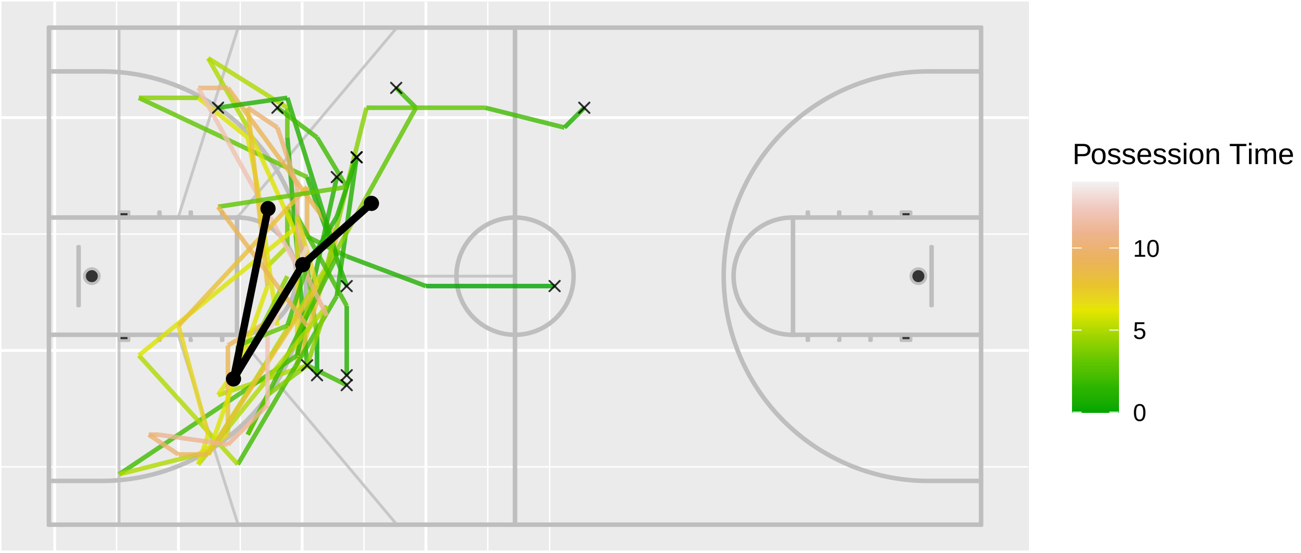

AUS Area 7 Cluster 3 : SelectTrajectories

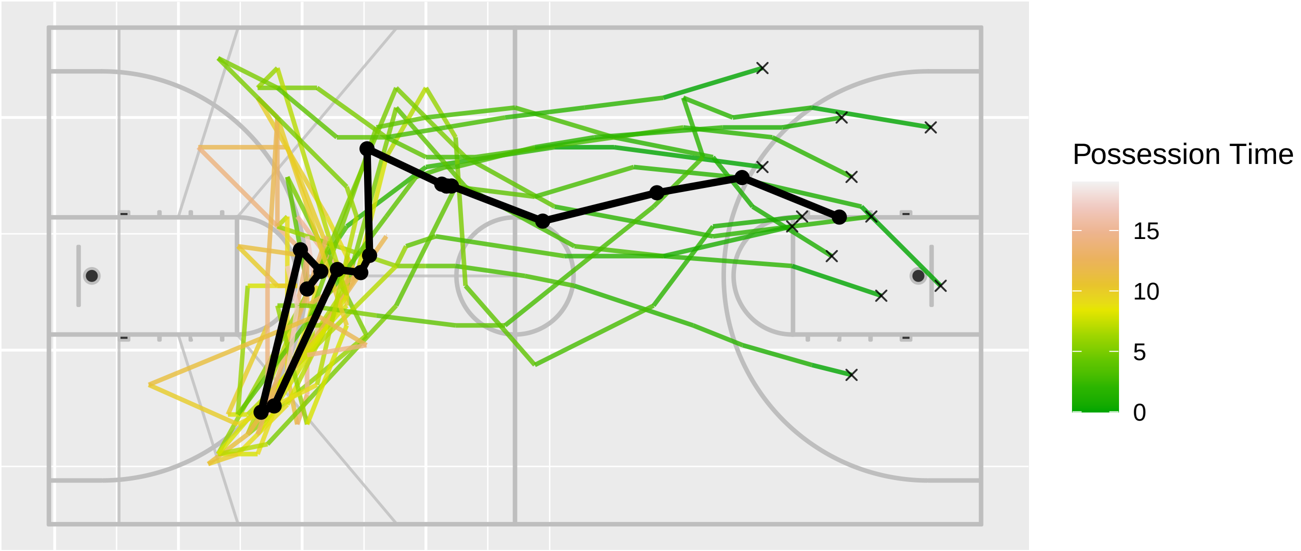

AUS Area 7 Cluster 4 : SelectTrajectories

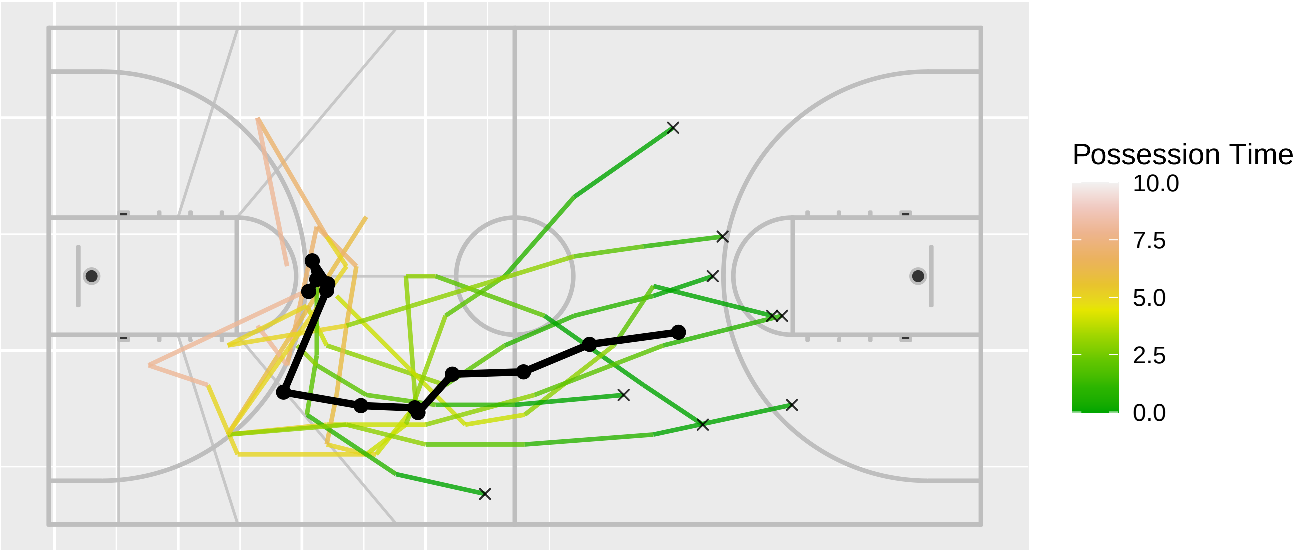

AUS Area 7 Cluster 5 : SelectTrajectories

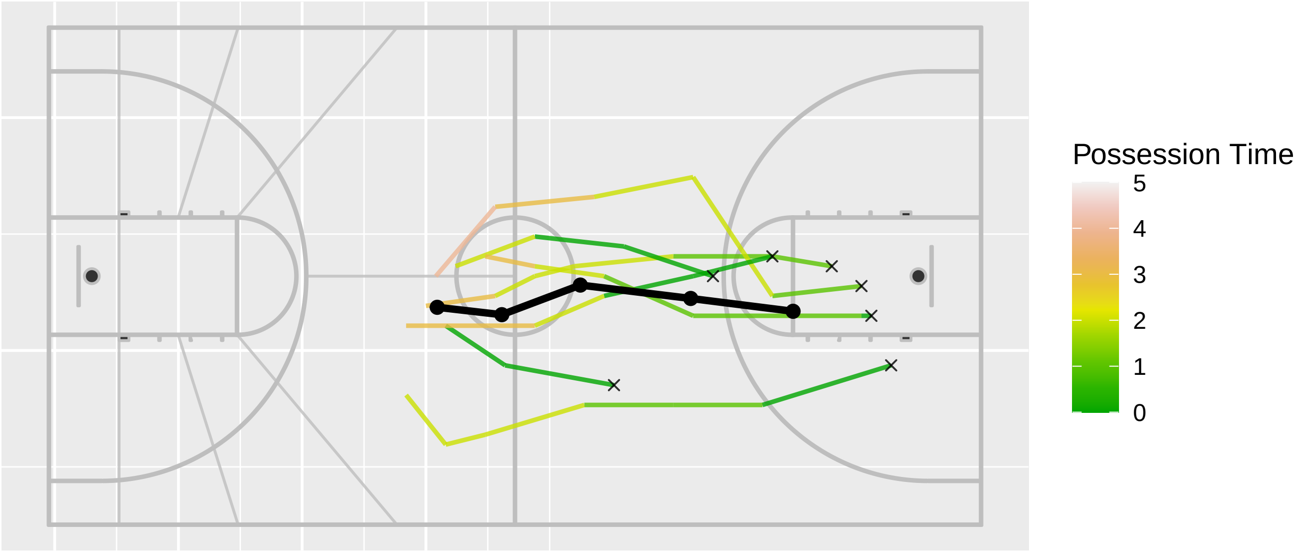

AUS Area 7 Cluster 6 : SelectTrajectories

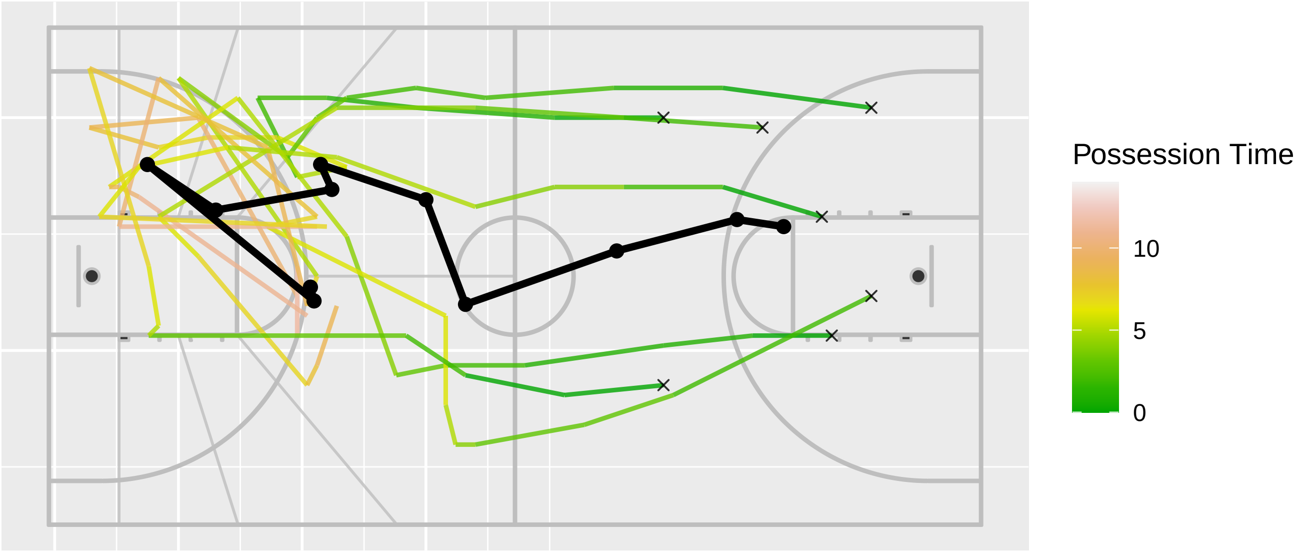

AUS Area 7 Cluster 7 : SelectTrajectories

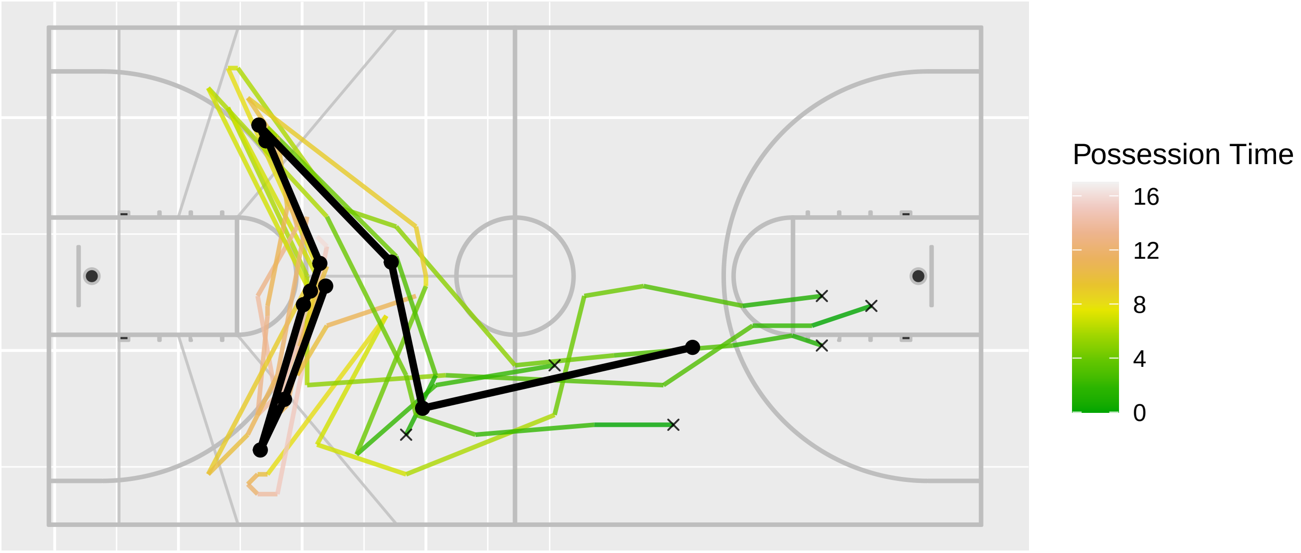

AUS Area 7 Cluster 8 : SelectTrajectories

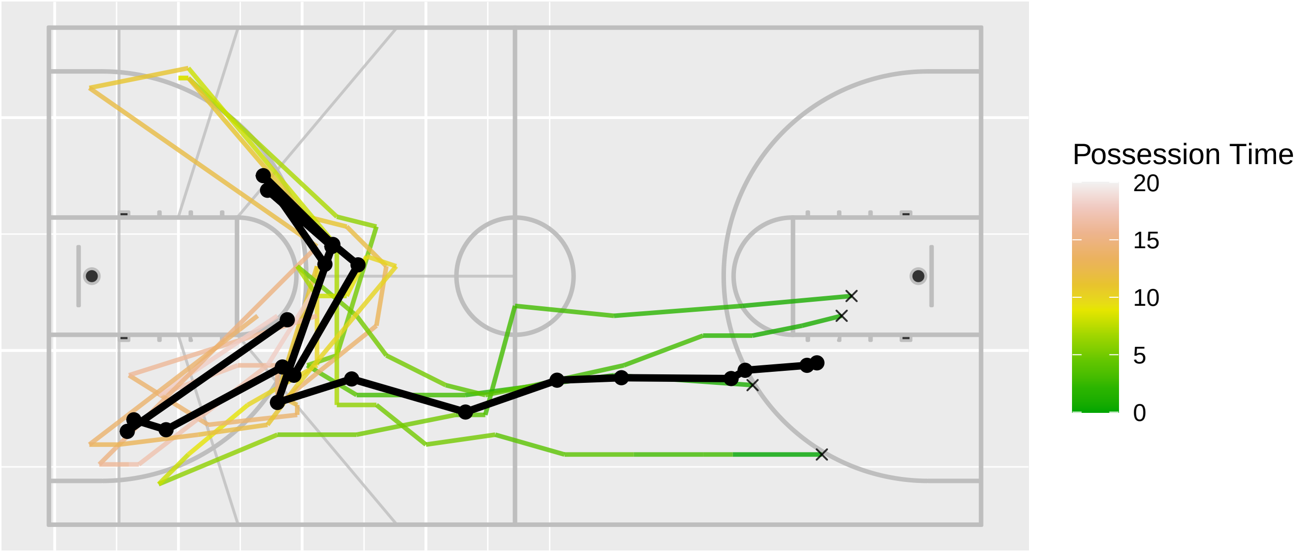

AUS Area 7 Cluster 9 : SelectTrajectories

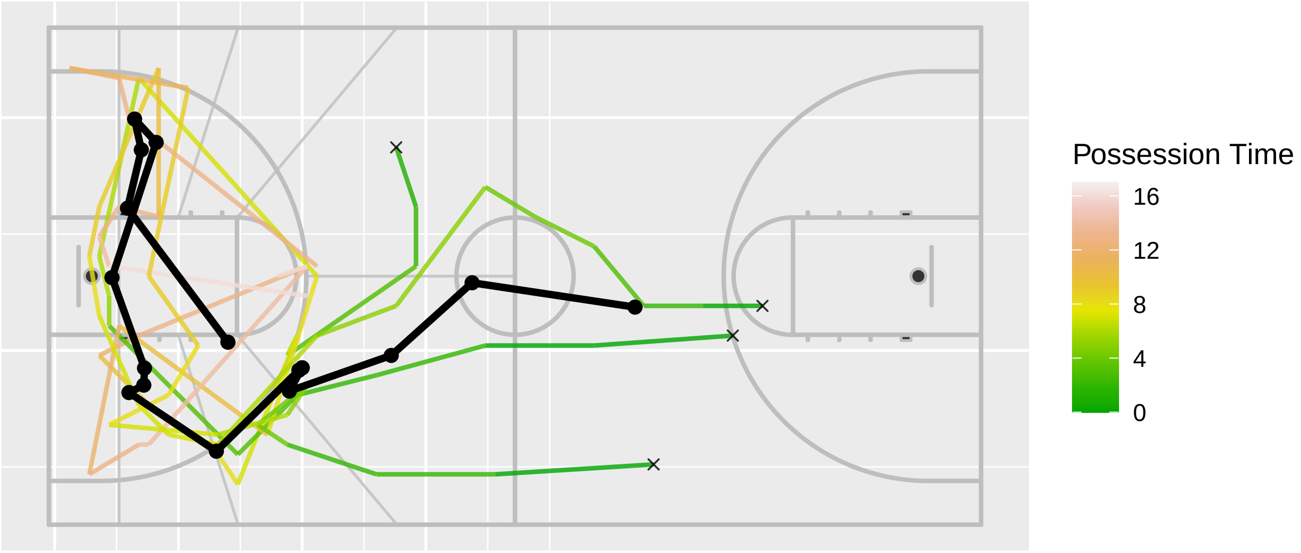

AUS Area 7 Cluster 10 : SelectTrajectories

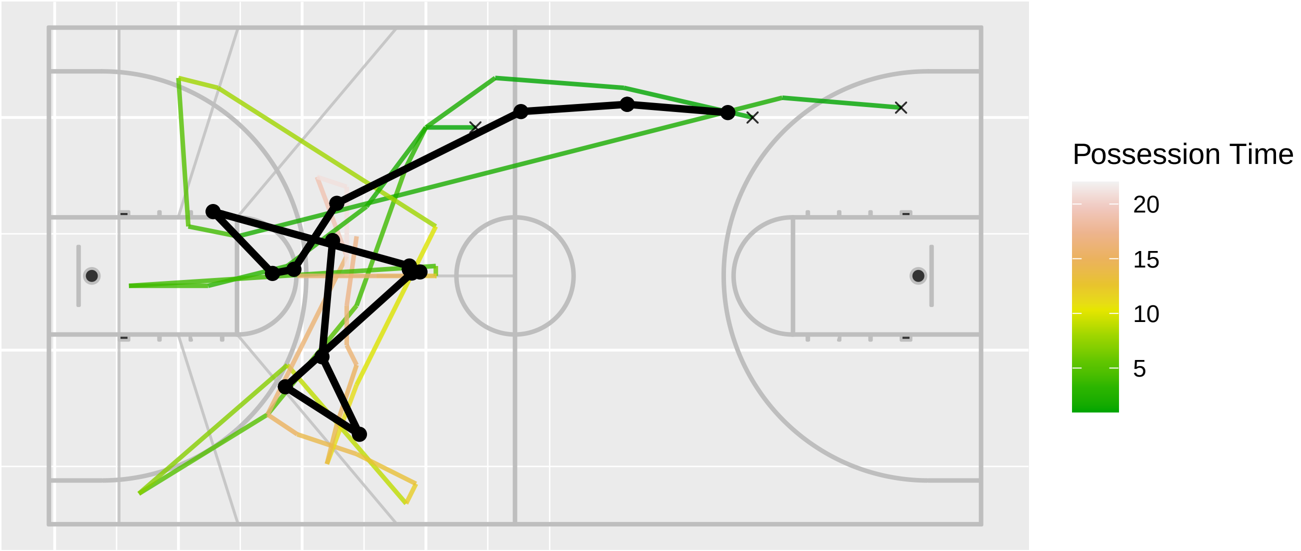

AUS Area 7 Cluster 11 : SelectTrajectories

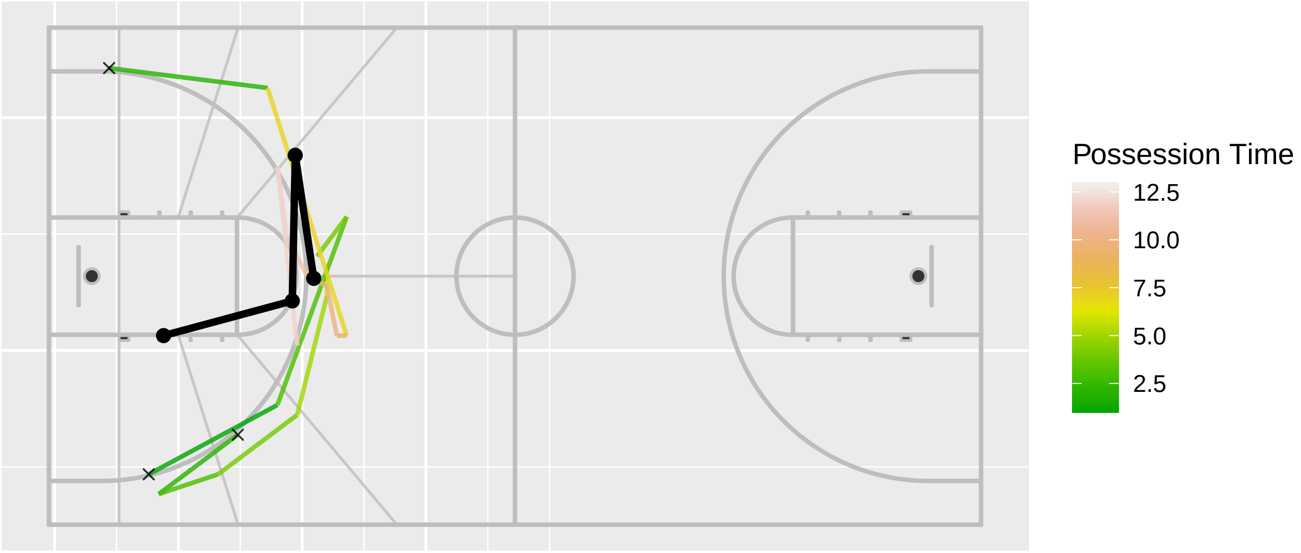

AUS Area 7 Cluster 12 : SelectTrajectories

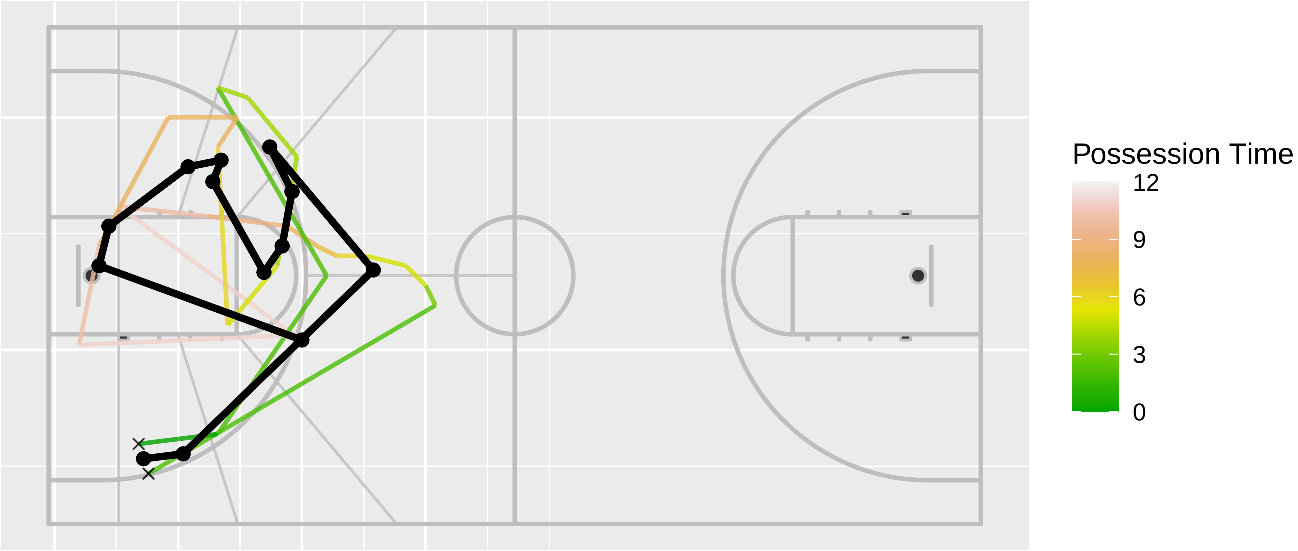

AUS Area 7 Cluster 13 : SelectTrajectories

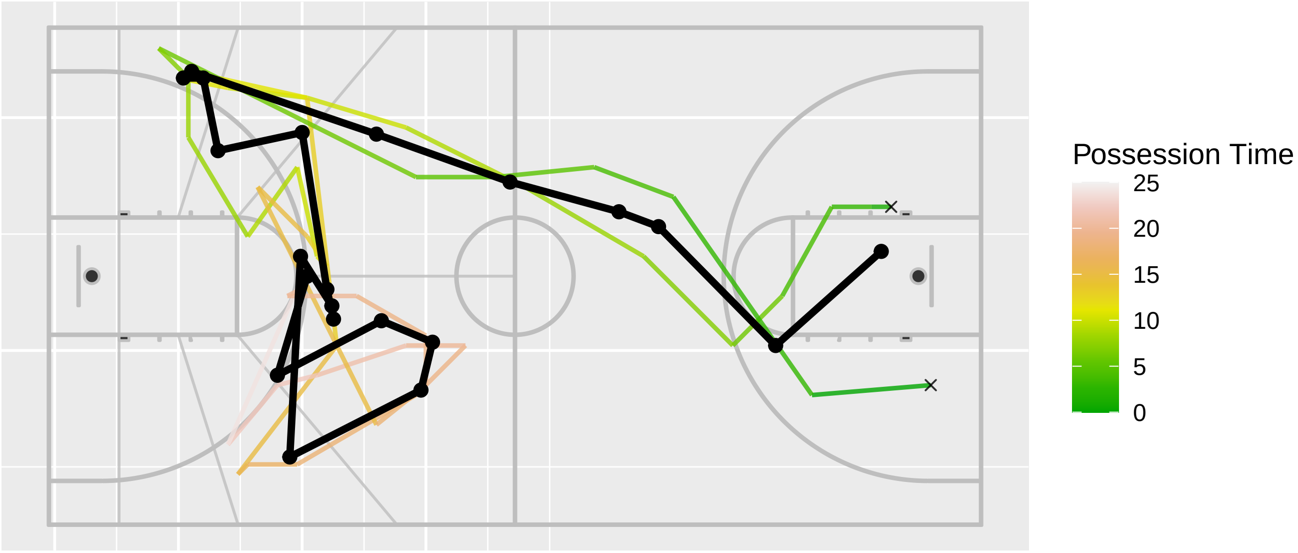

AUS Area 7 Cluster 14 : SelectTrajectories

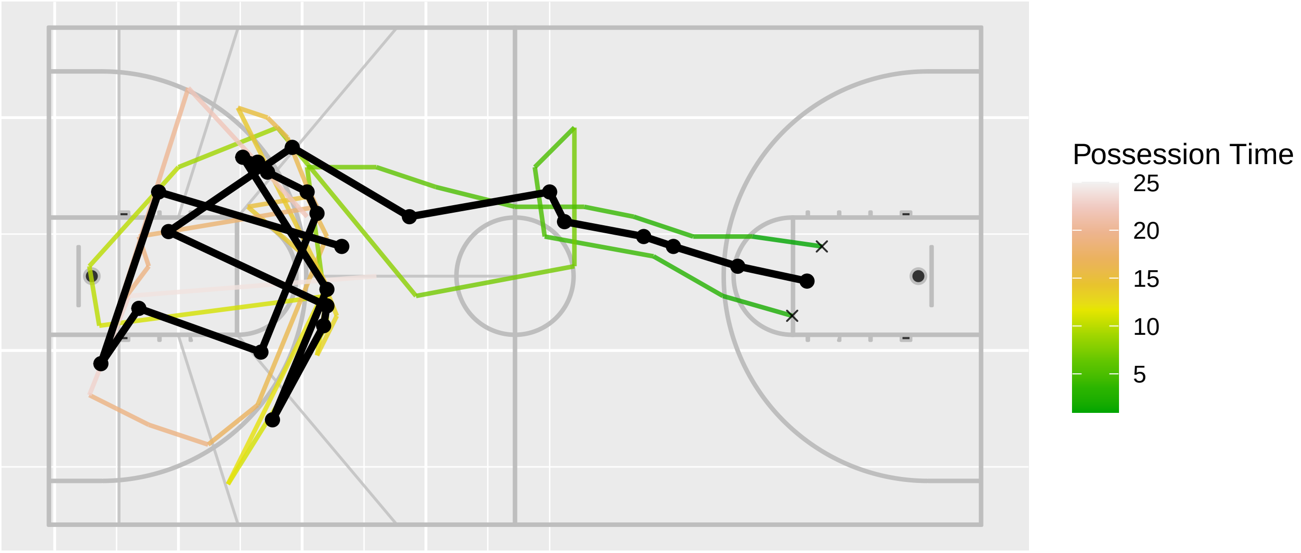

AUS Area 7 Cluster 15 : SelectTrajectories

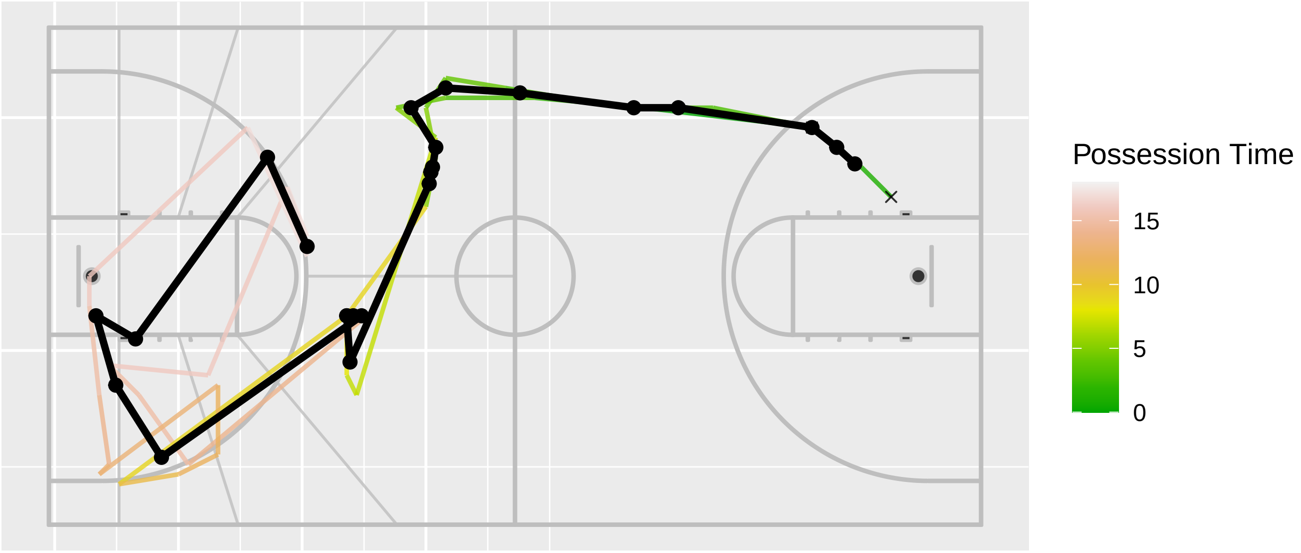

Supplement: S5 Appendix — (PDF) [file pone.0272848.s005.pdf]
