# Supplementary material for: Classifying ball trajectories in invasion sports using dynamic time warping: A basketball case study
Source: PLoS One. 2022 Oct 20;17(10):e0272848. doi: 10.1371/journal.pone.0272848 (PMC9584368; doi:10.1371/journal.pone.0272848)

JPN Area 1 with cluster c(20, 20, 50, 50, 50, 20, 50) Cluster dendrogram

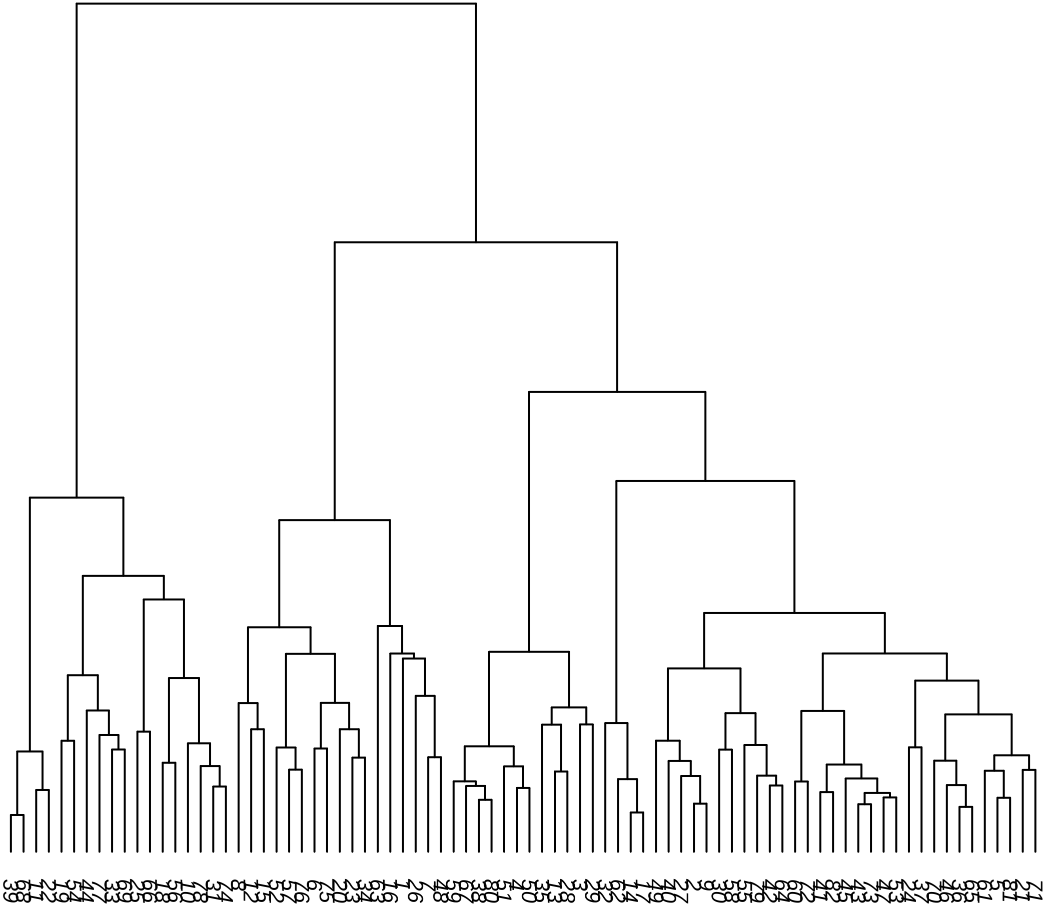

JPN Area 1 Unrooted Cluster dendrogram

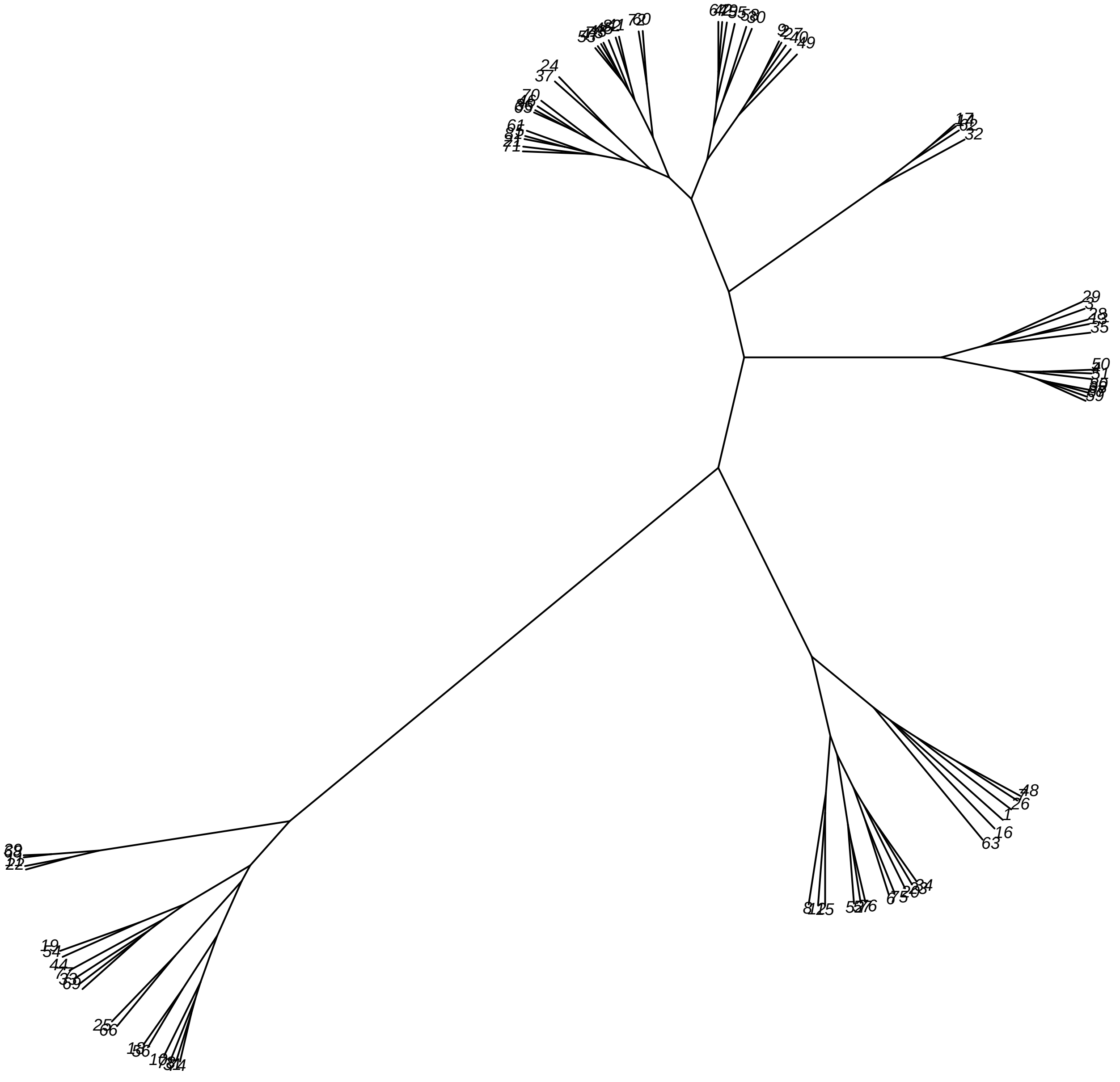

JPN Area 1 Fan Cluster dendrogram

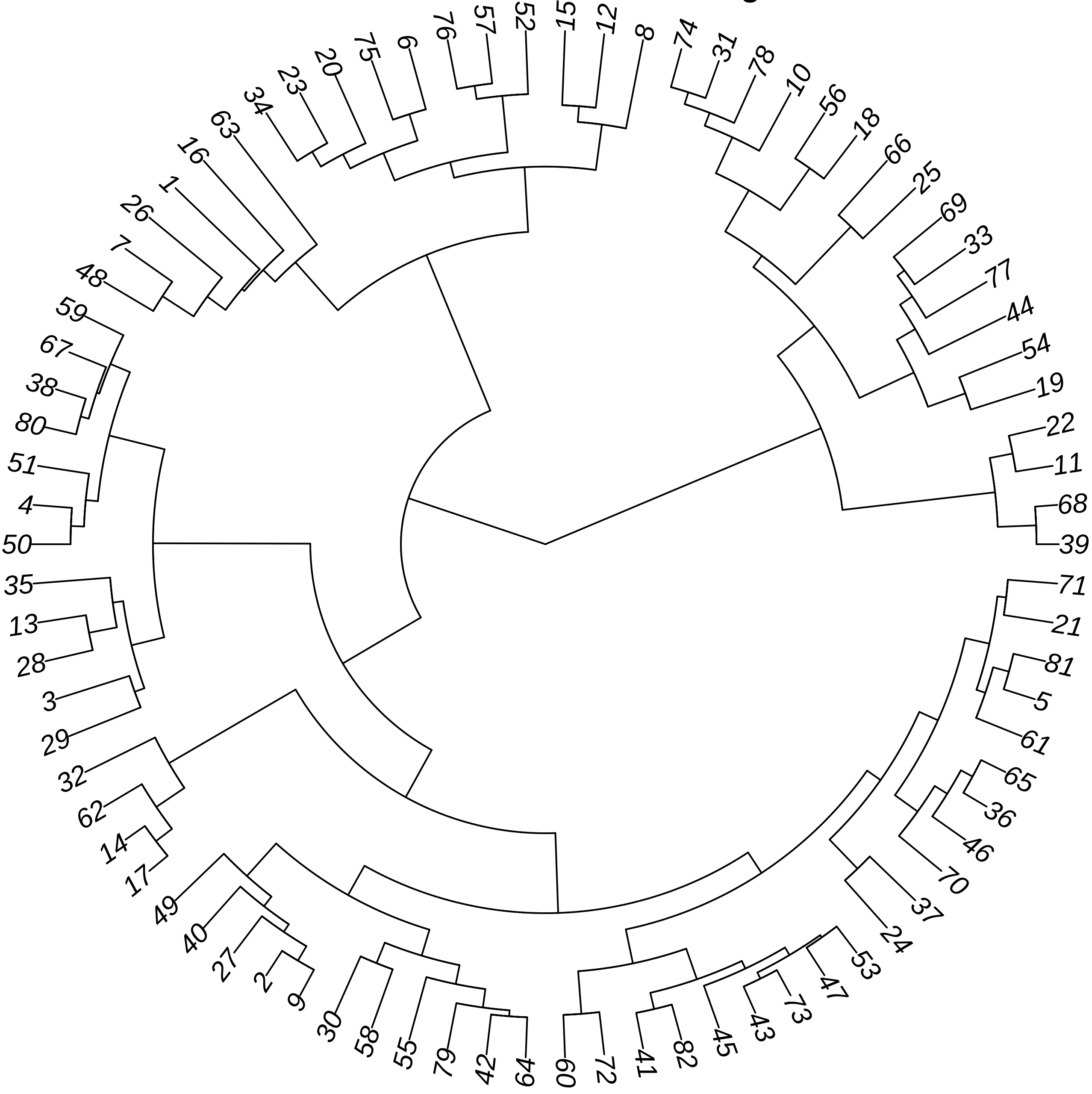

JPN Area 1 at h = 15 : Cluster dendrogram

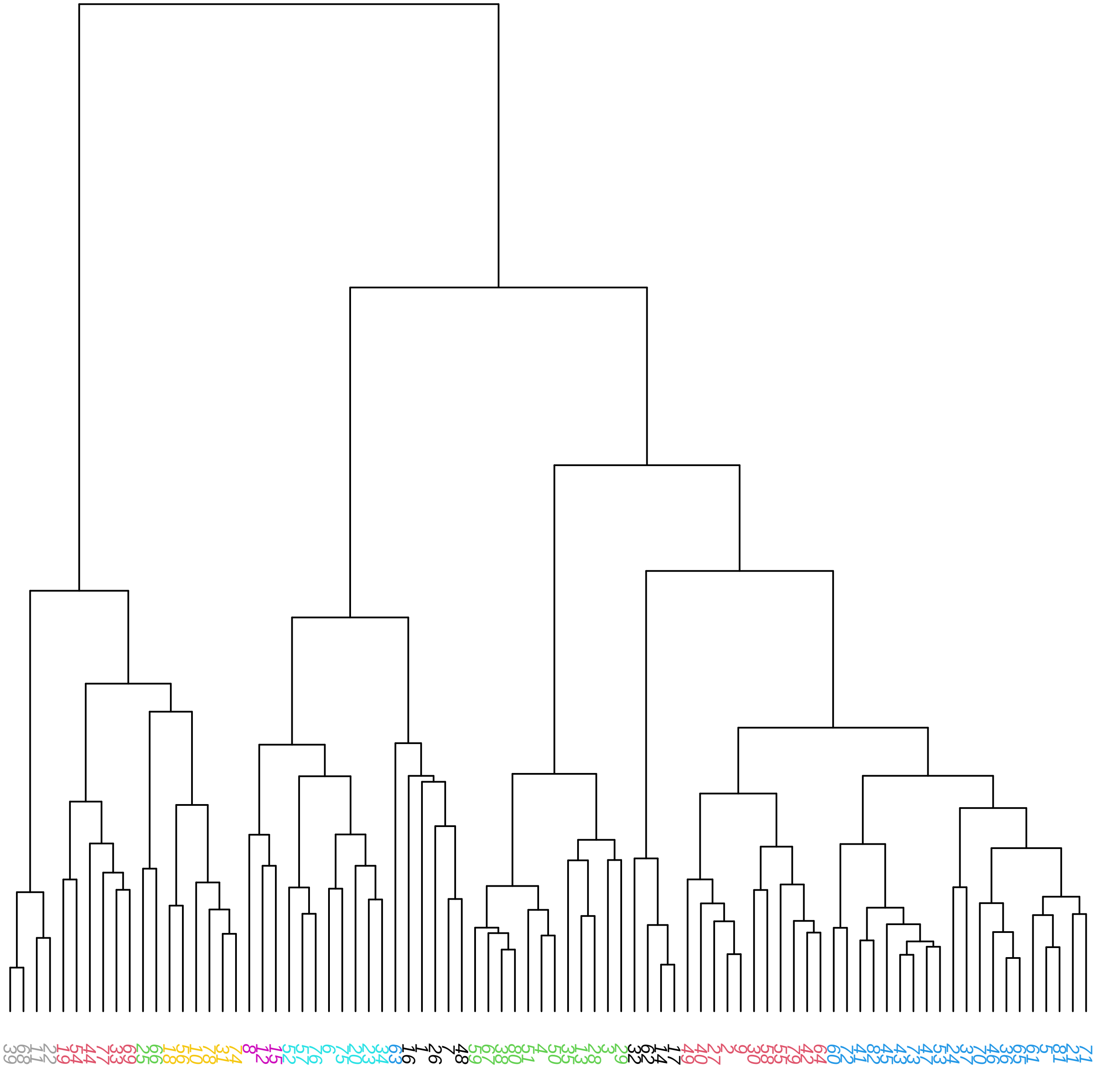

JPN Area 1 at h = 15 : Coloured Unrooted Cluster dendrogram

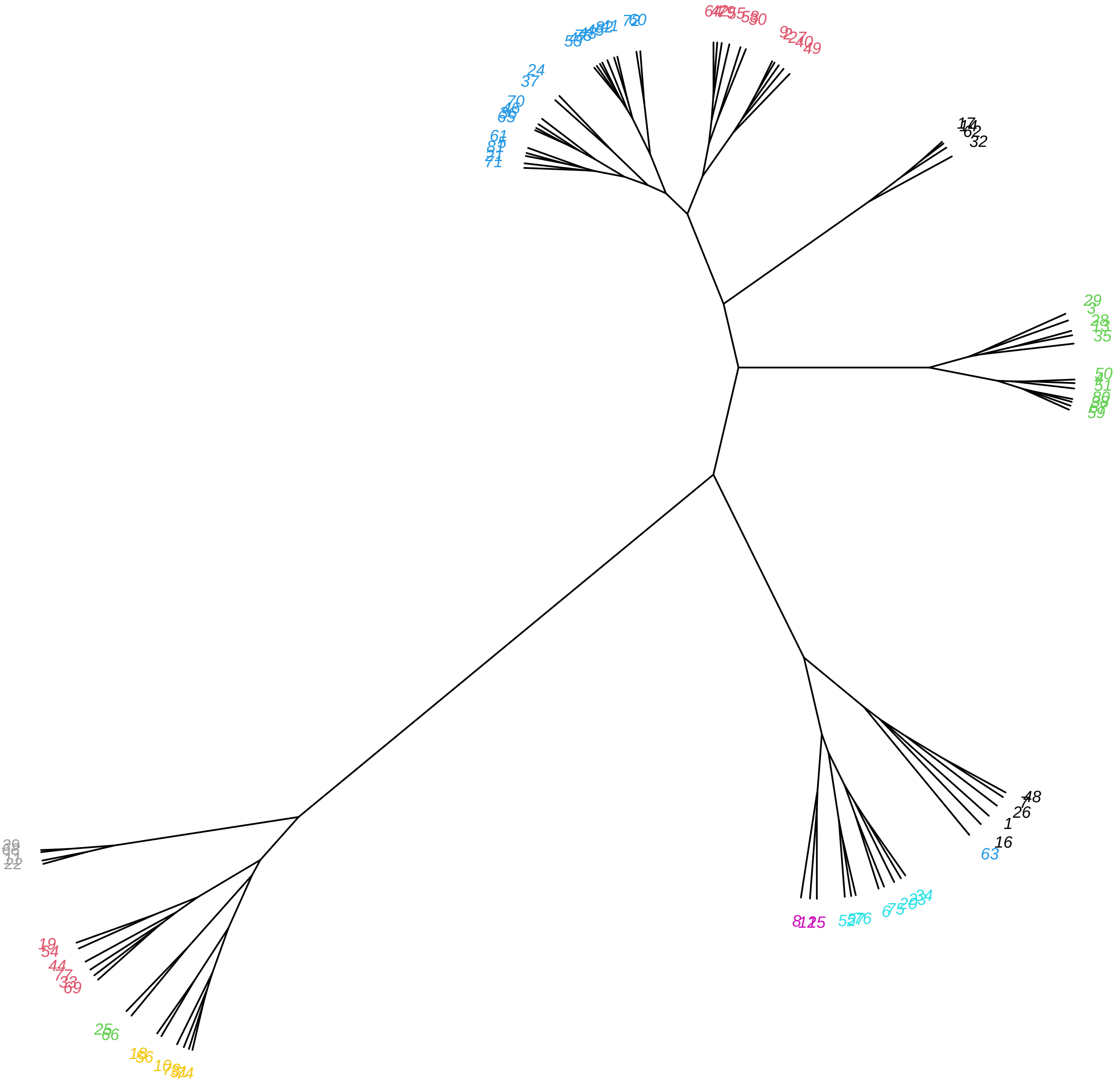

JPN Area 1 at h = 15 : Coloured Fan Cluster dendrogram

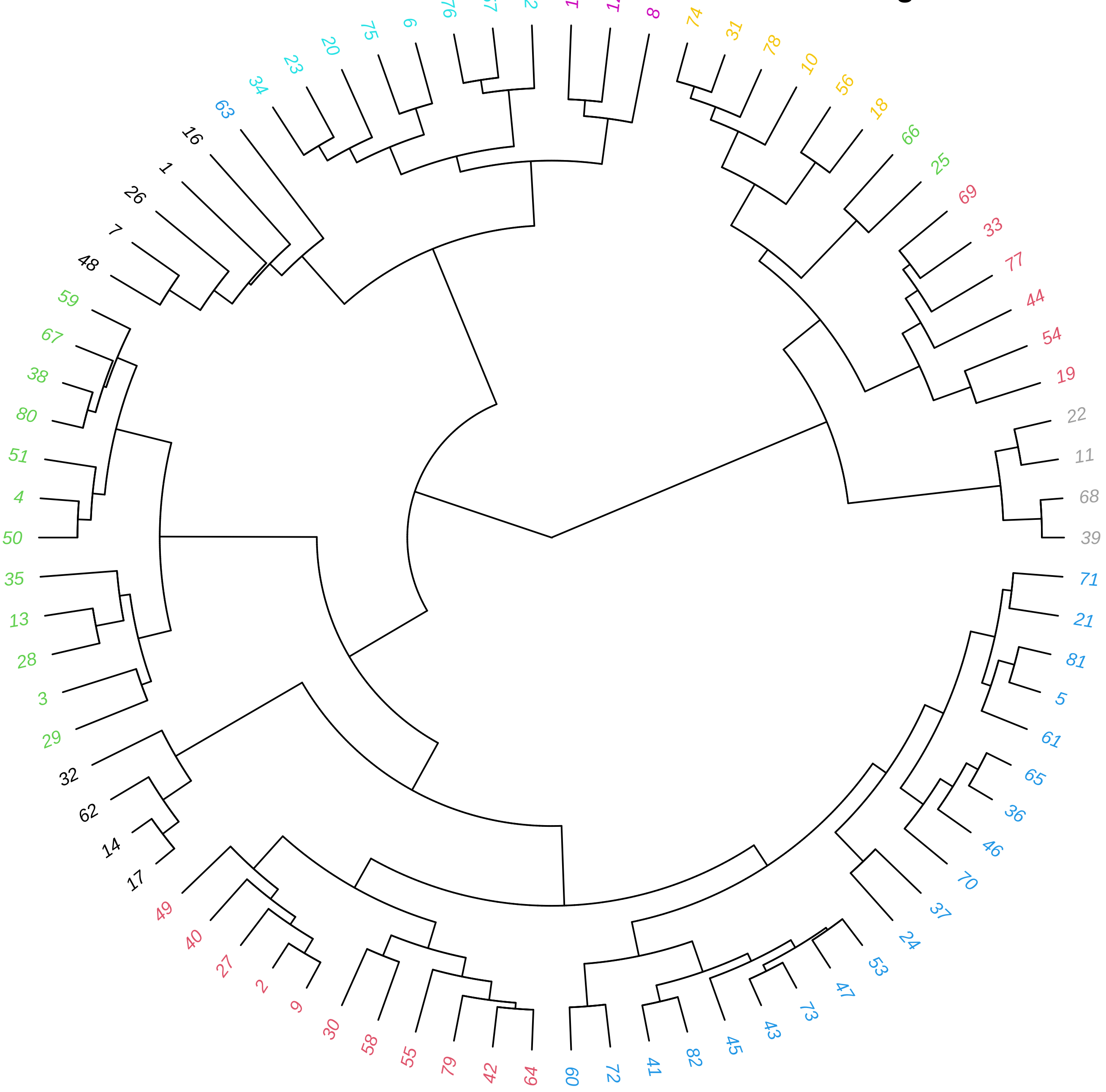

JPN Area 2 with cluster c(20, 20, 50, 50, 50, 20, 50) Cluster dendrogram

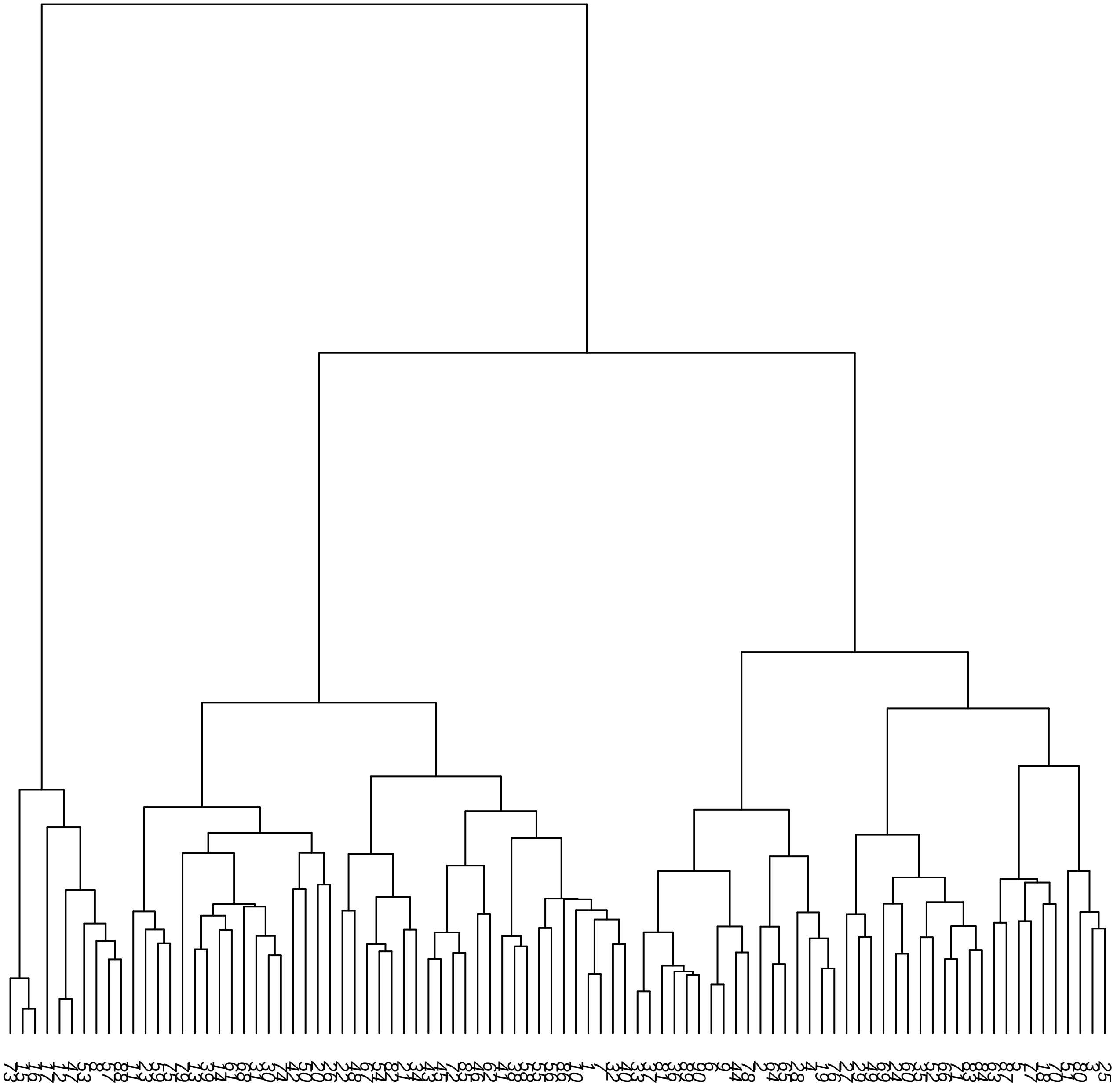

JPN Area 2 Unrooted Cluster dendrogram

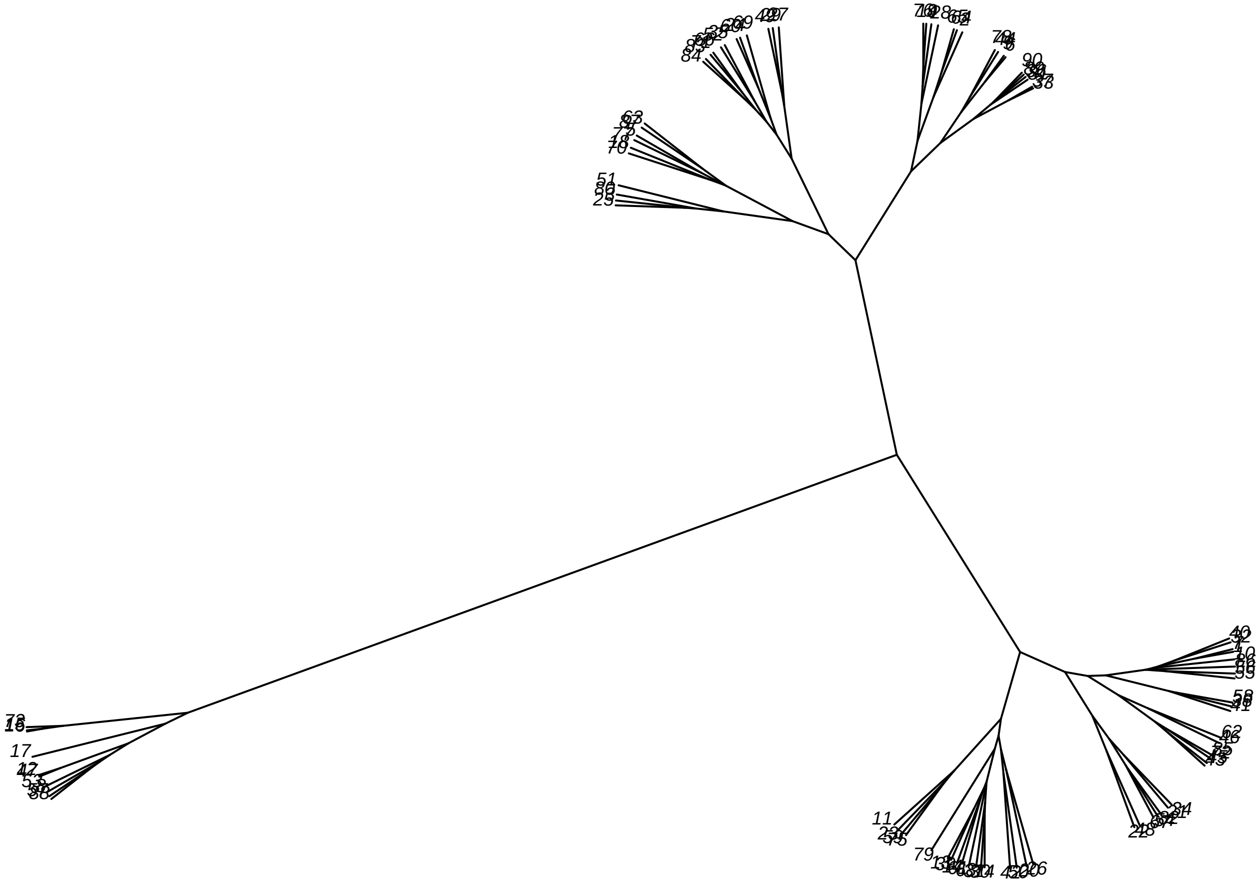

JPN Area 2 Fan Cluster dendrogram

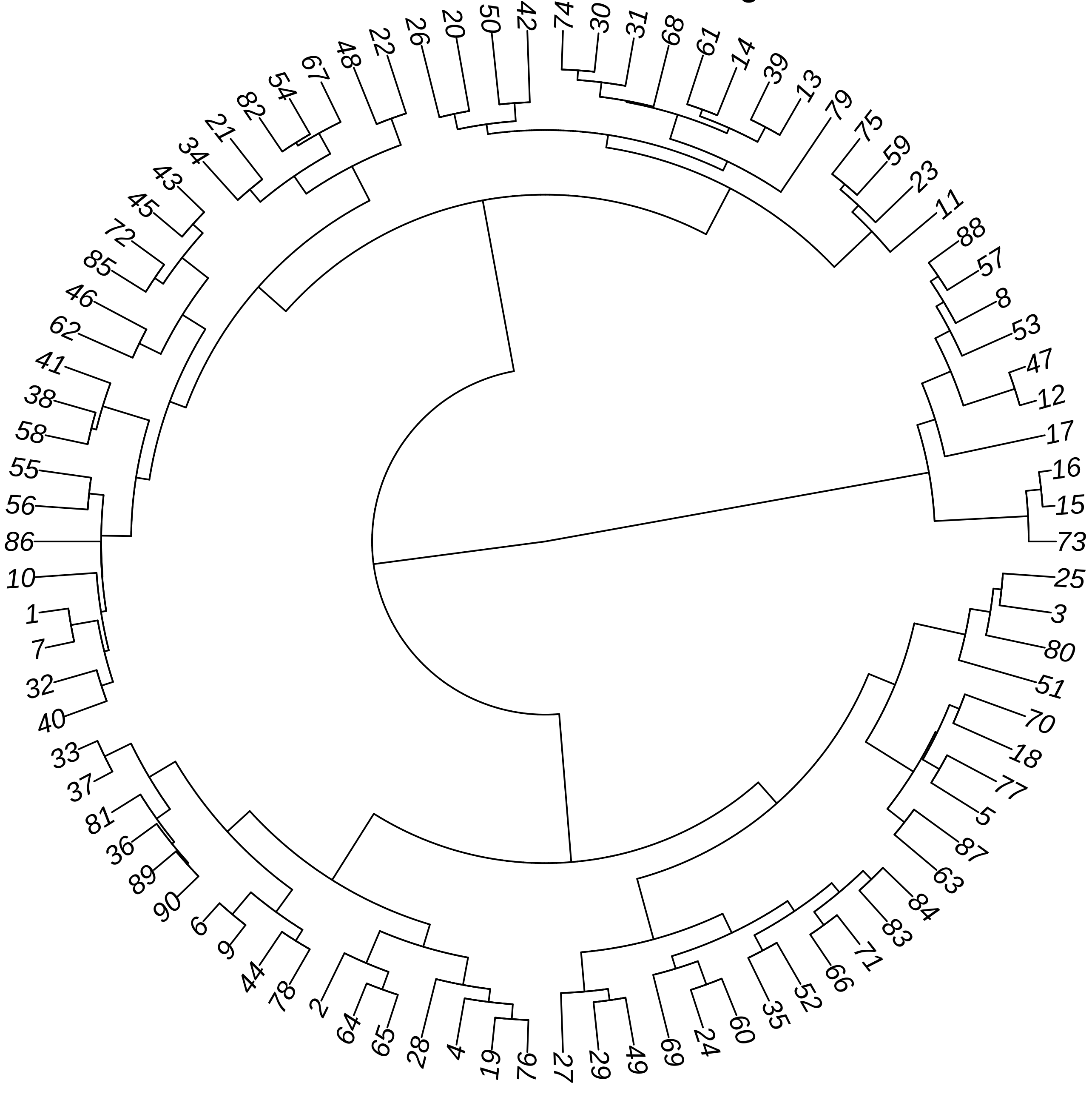

JPN Area 2 at h = 15 : Cluster dendrogram

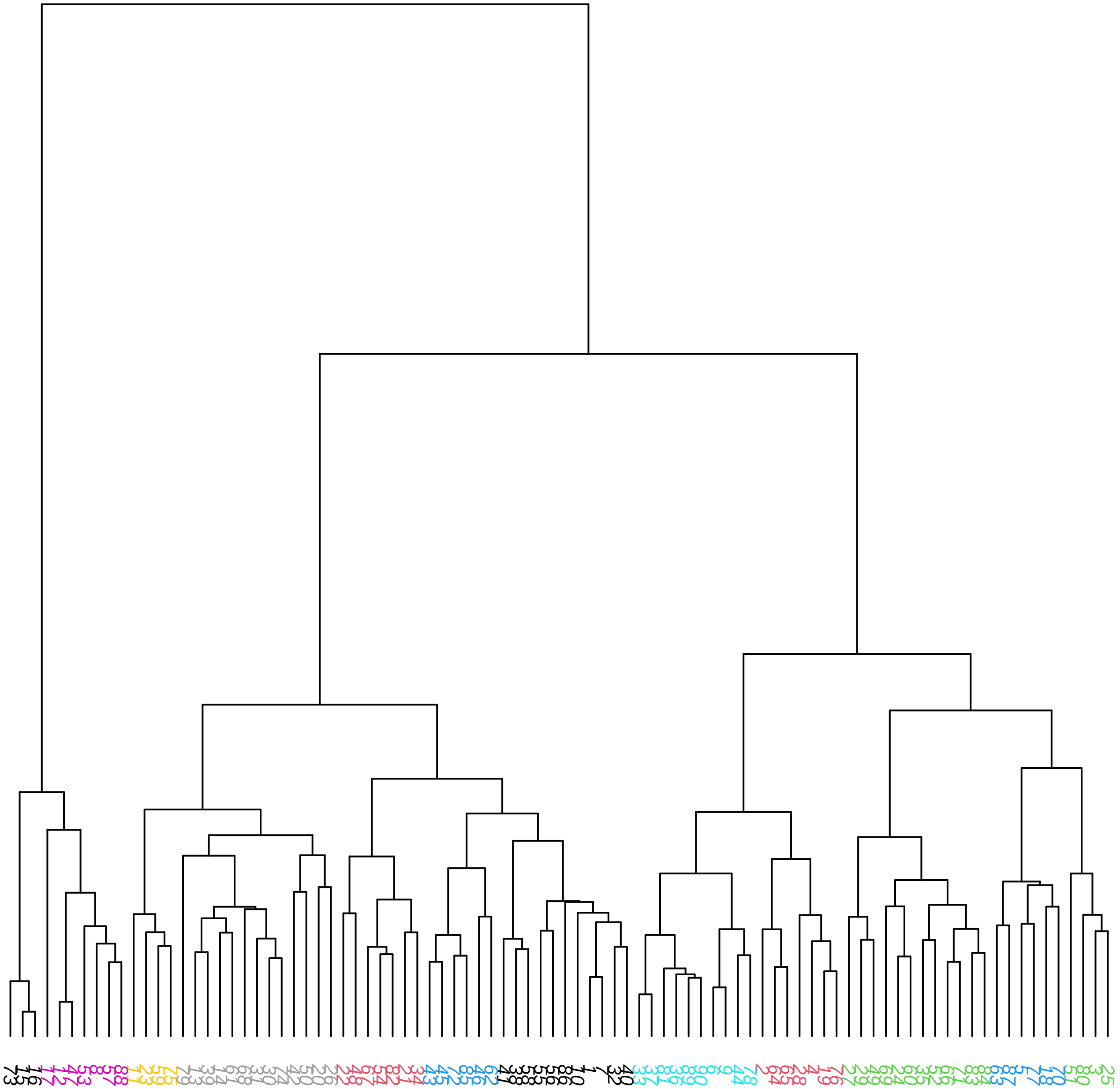

JPN Area 2 at h = 15 : Coloured Unrooted Cluster dendrogram

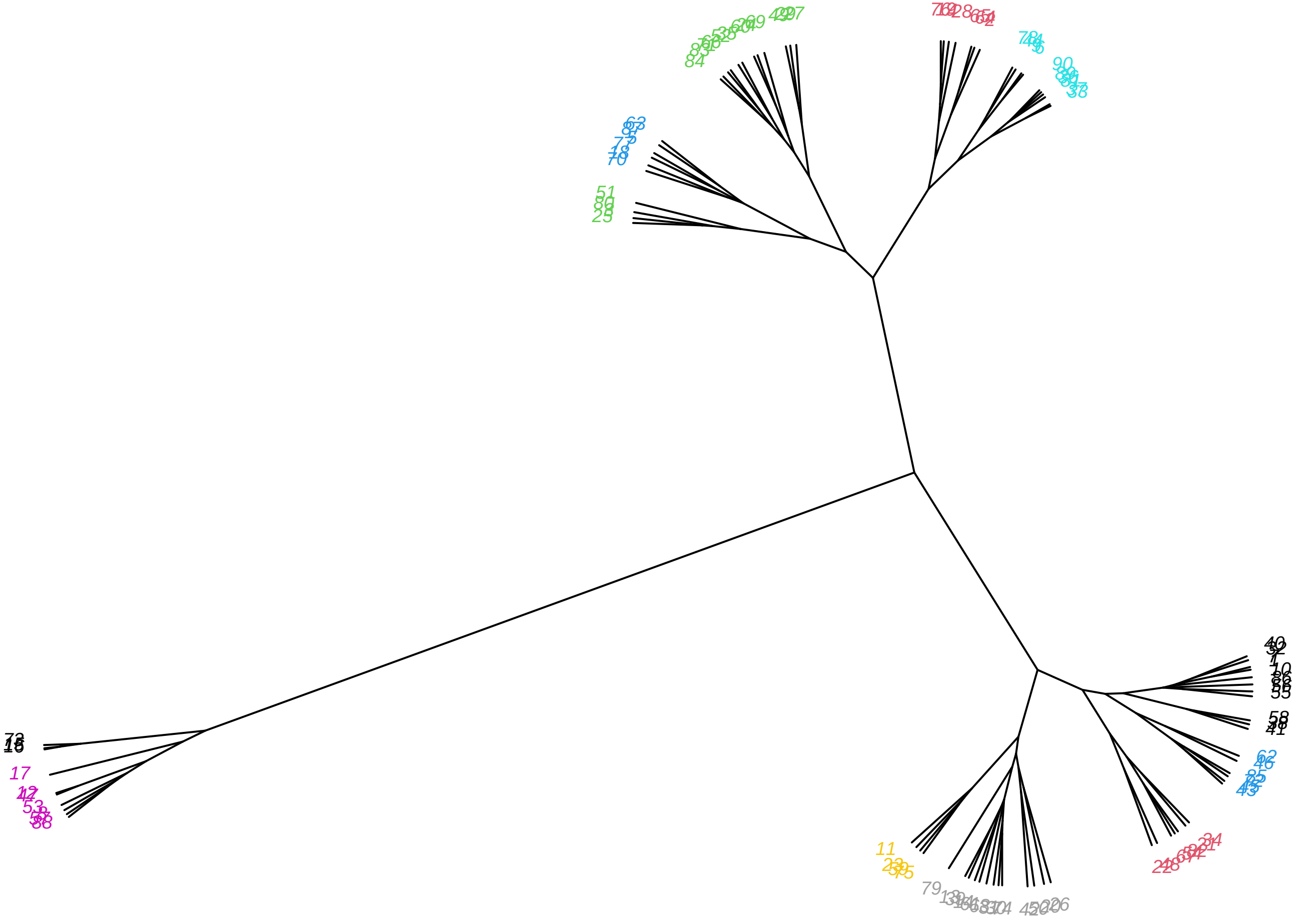

JPN Area 2 at h = 15 : Coloured Fan Cluster dendrogram

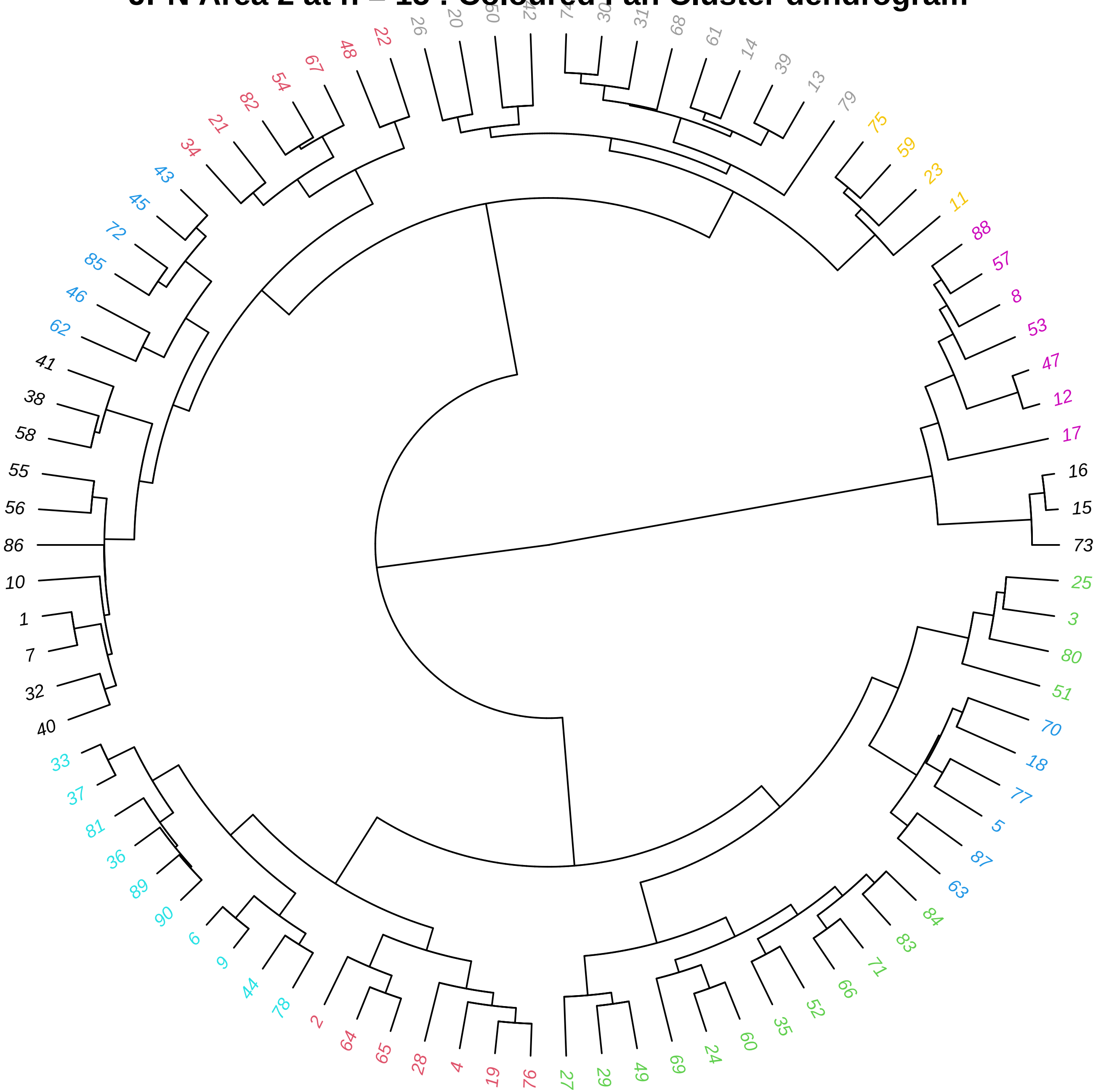

JPN Area 3 with cluster c(20, 20, 50, 50, 50, 20, 50) Cluster dendrogram

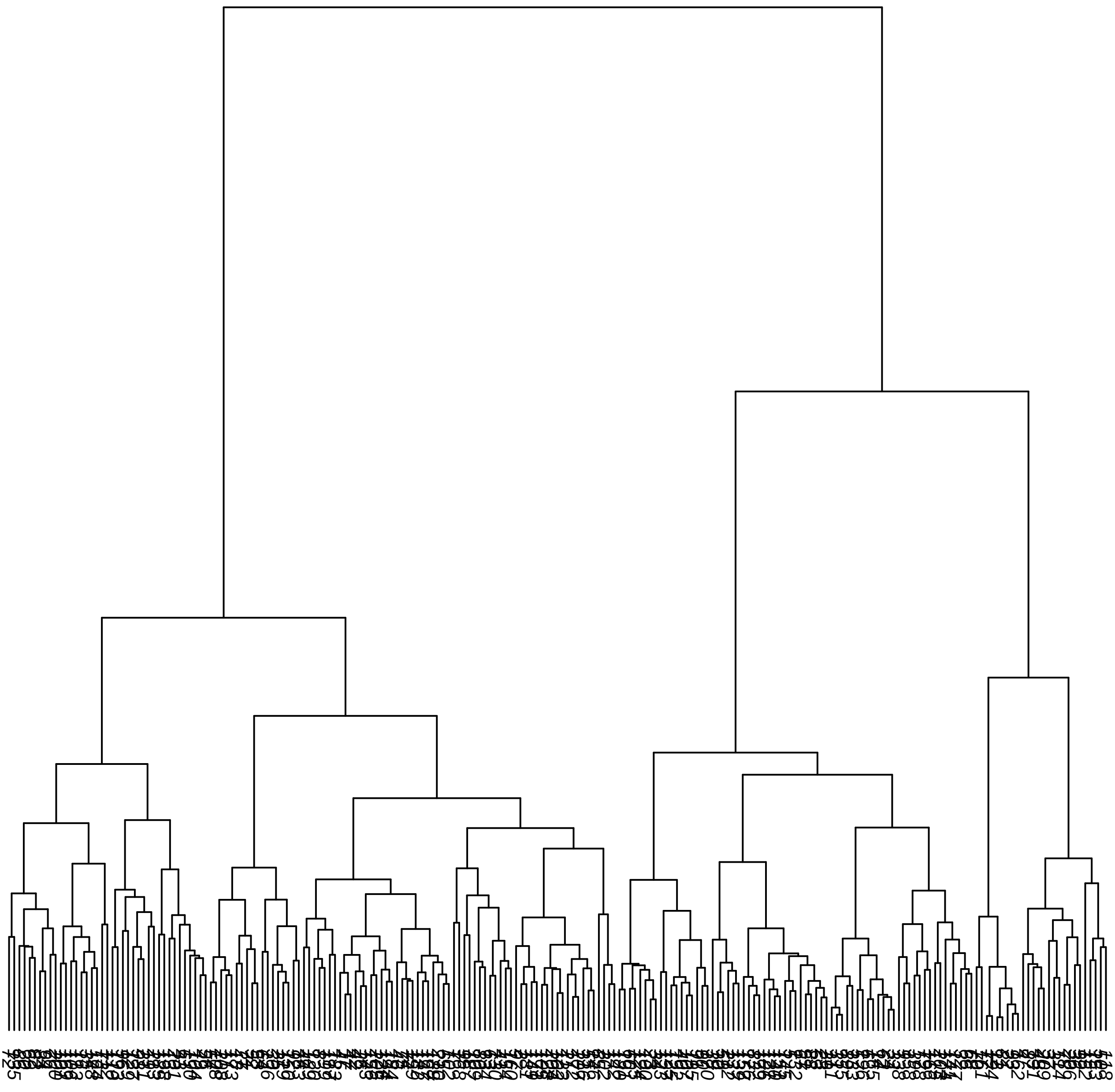

JPN Area 3 Unrooted Cluster dendrogram

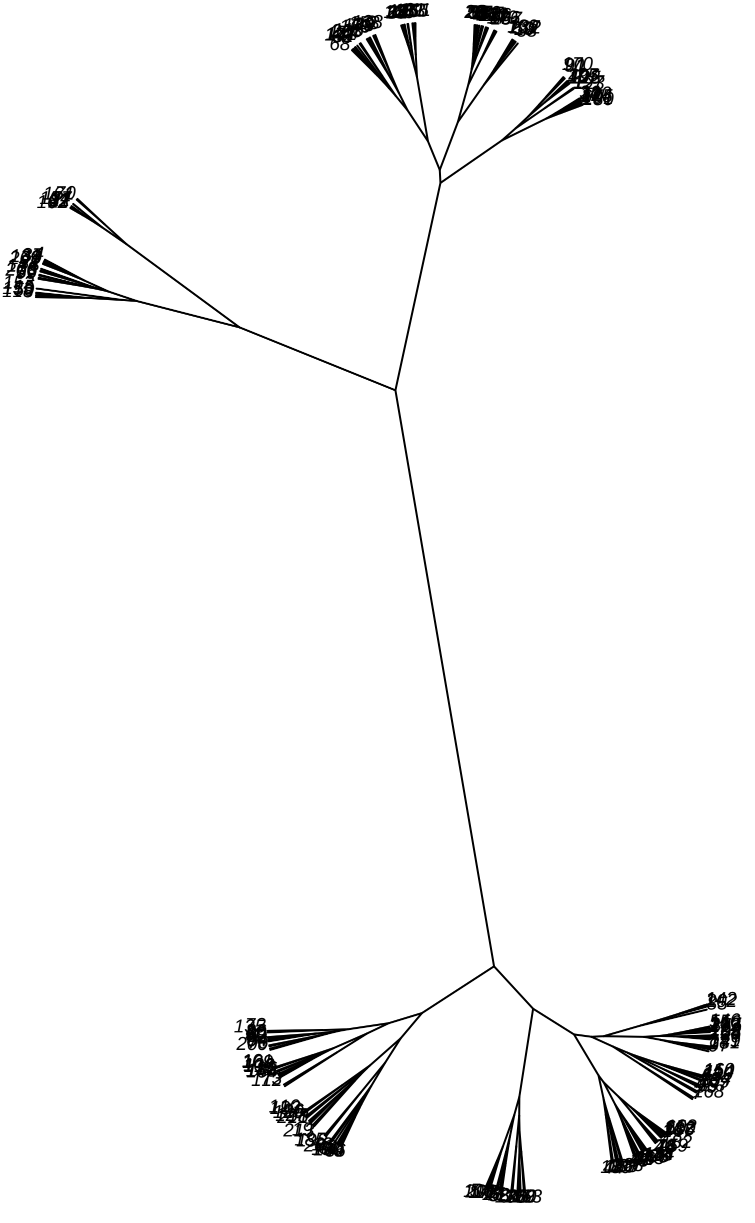

JPN Area 3 Fan Cluster dendrogram

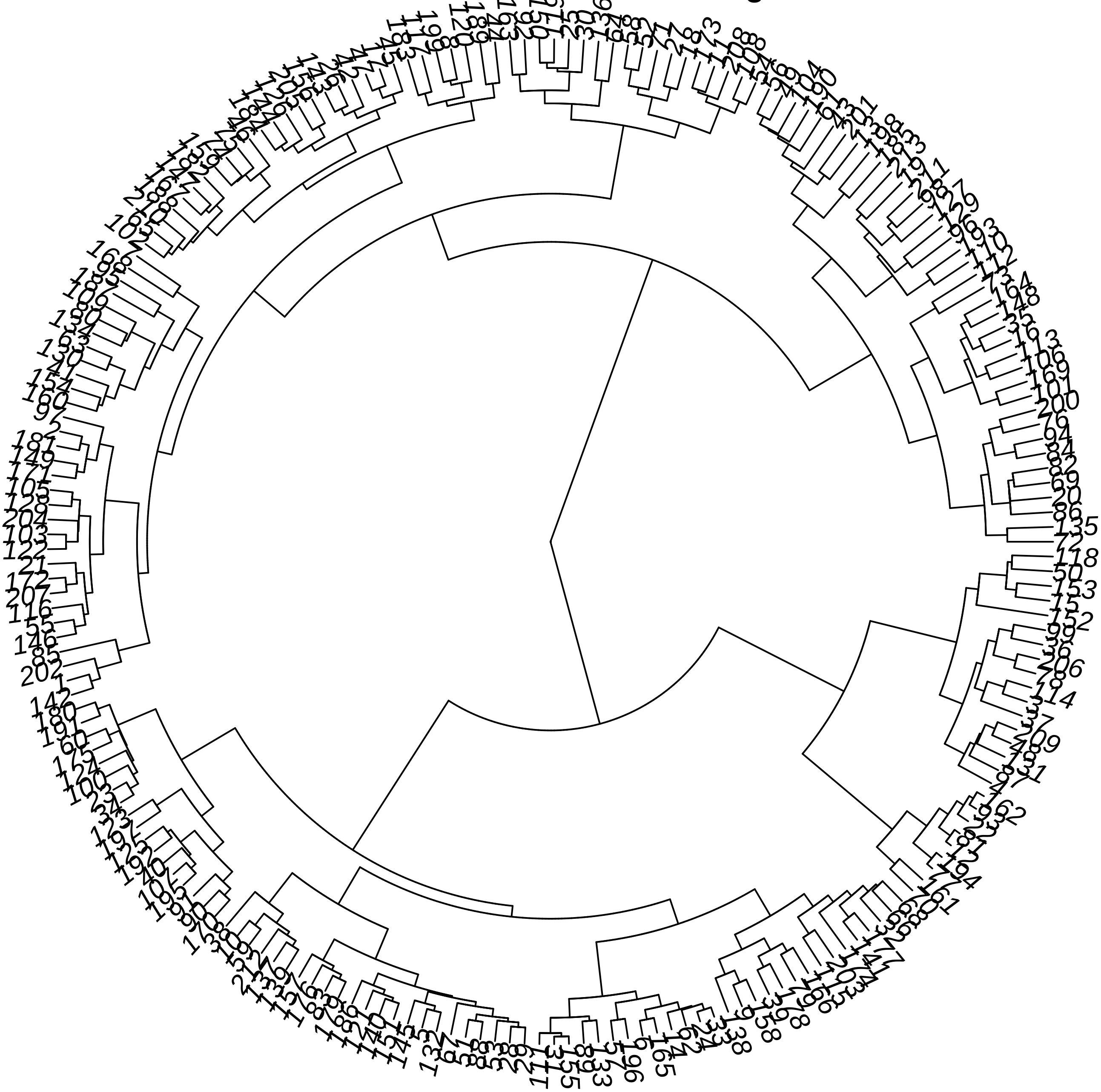

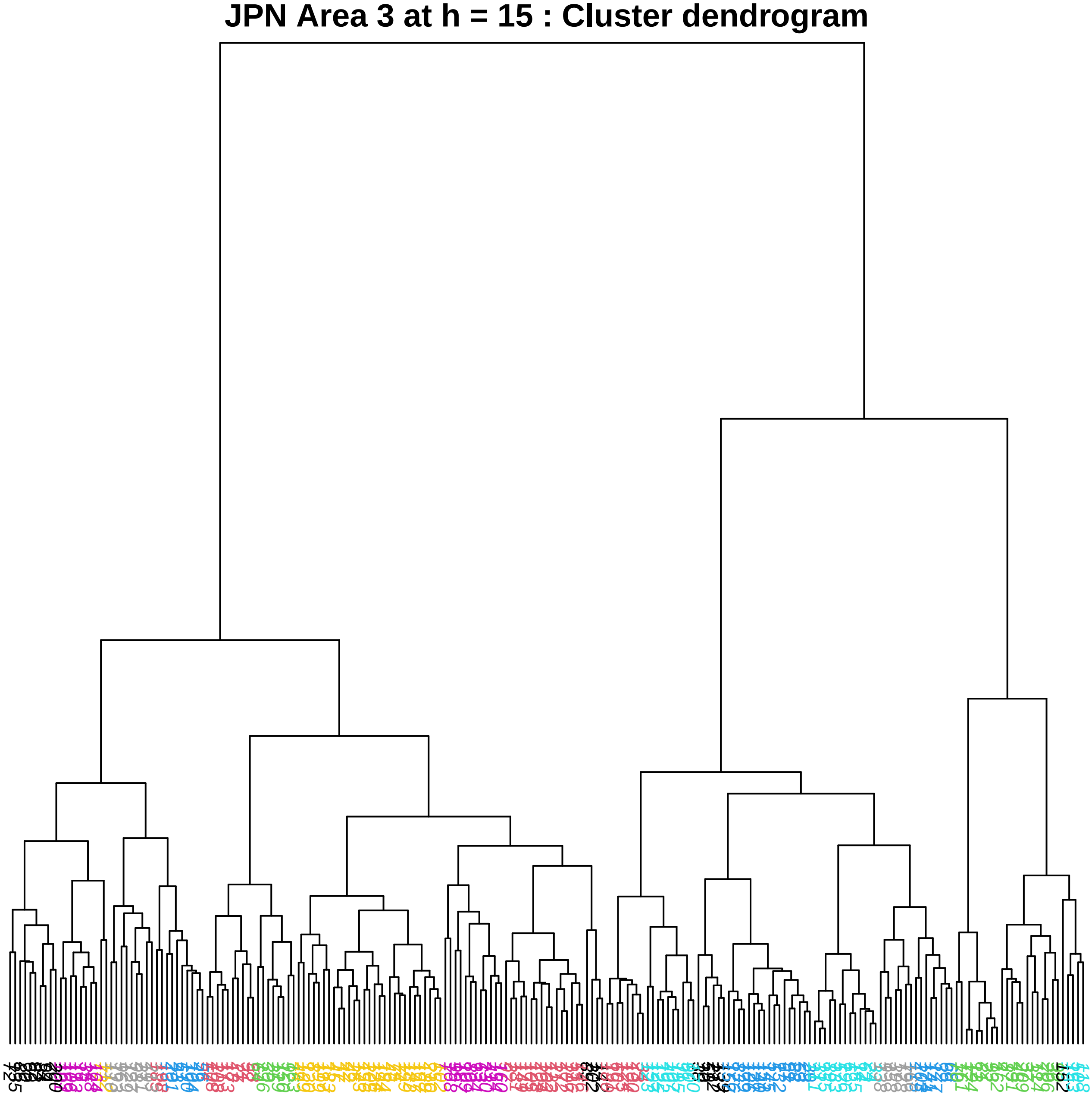
JPN Area 3 at h = 15 : Cluster dendrogram

JPN Area 3 at h = 15 : Coloured Unrooted Cluster dendrogram

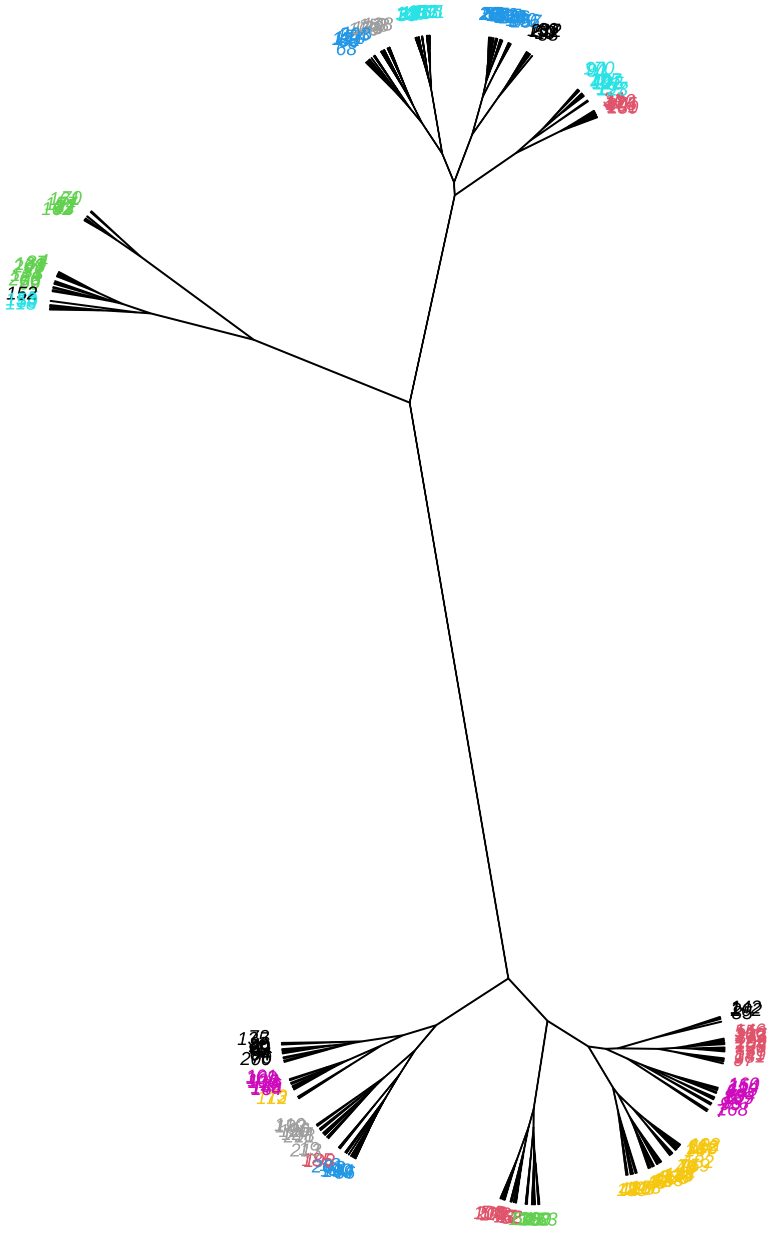

JPN Area 3 at h = 15 : Coloured Fan Cluster dendrogram

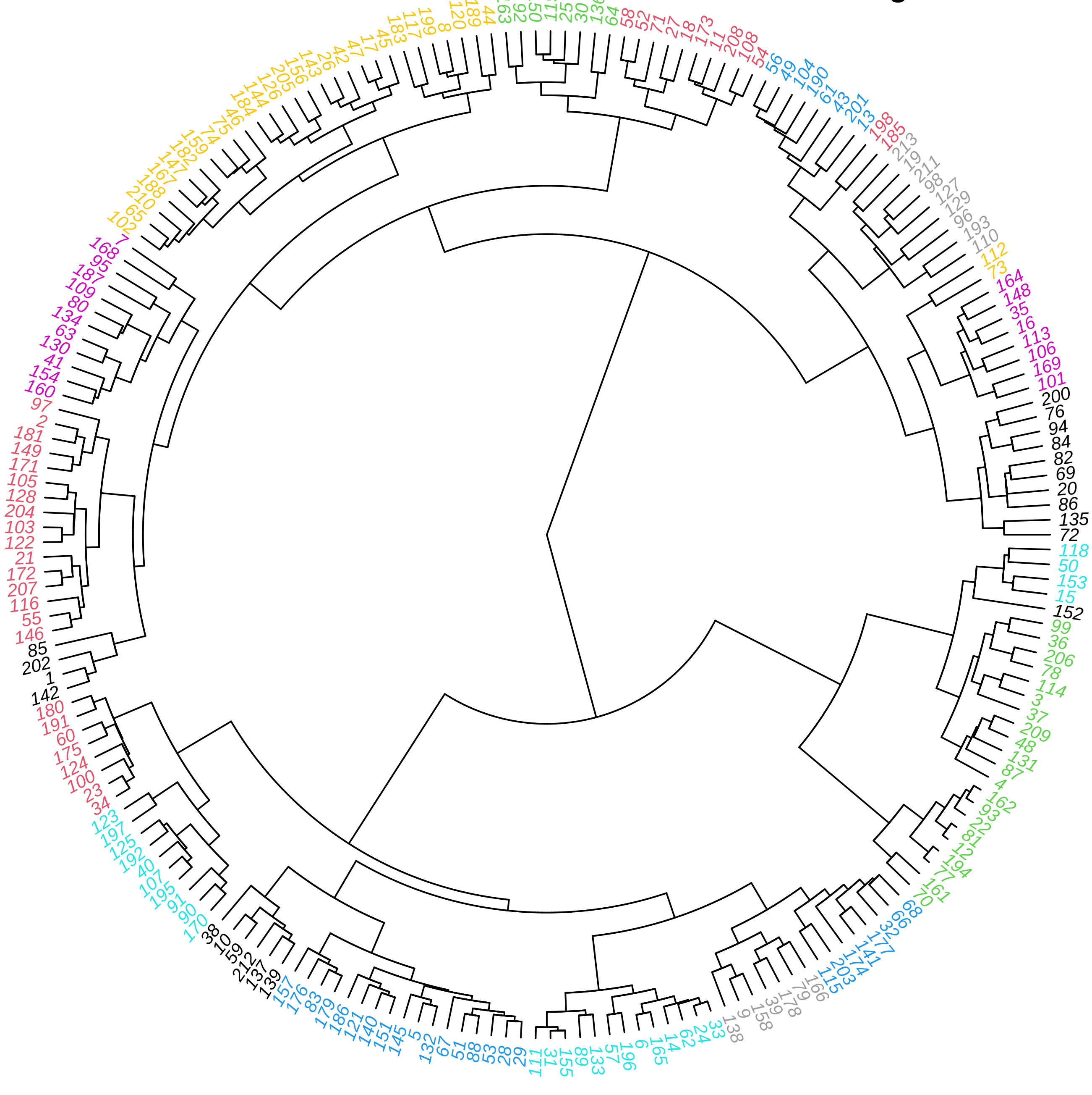

JPN Area 4 with cluster c(20, 20, 50, 50, 50, 20, 50) Cluster dendrogram

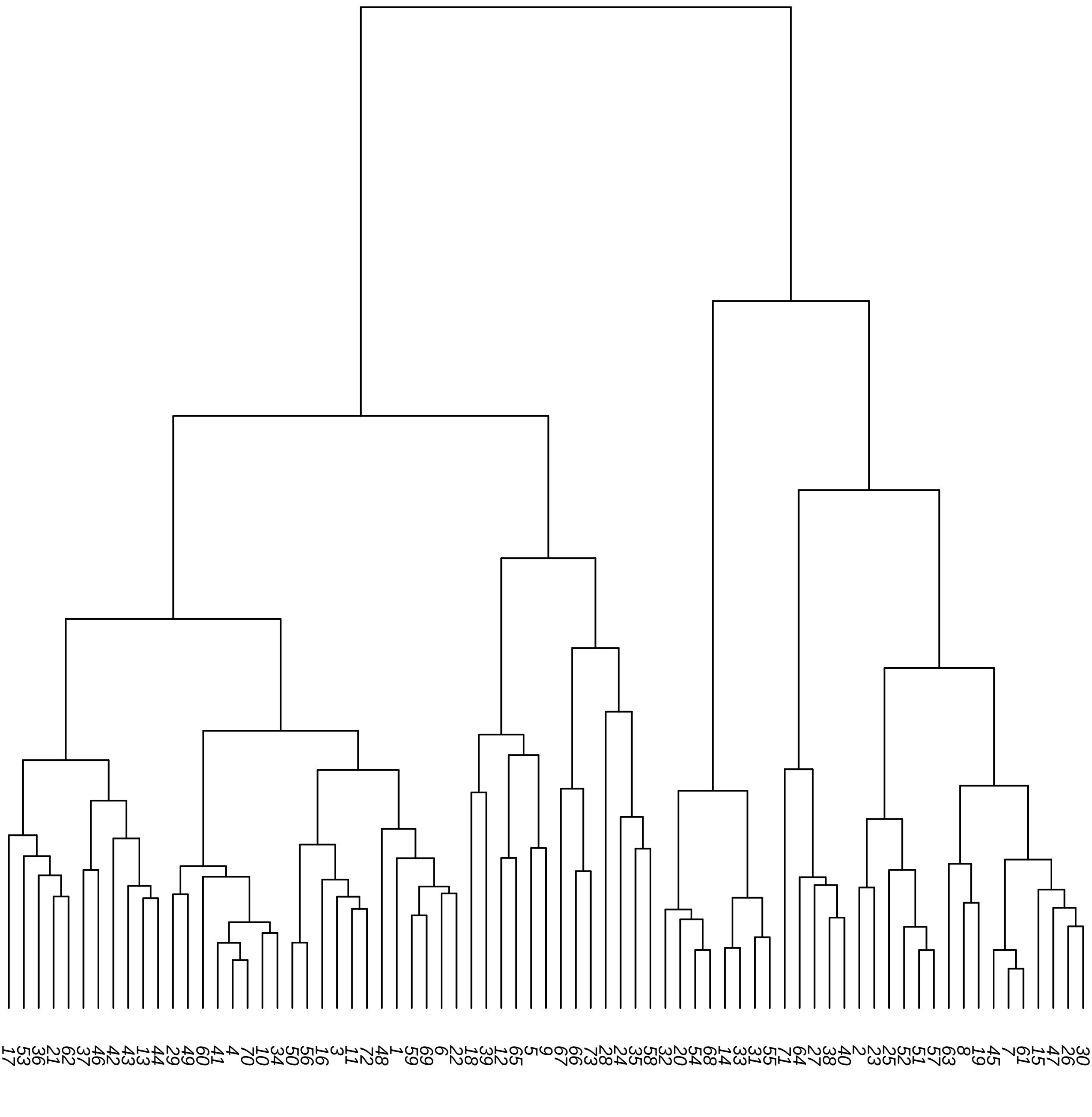

JPN Area 4 Unrooted Cluster dendrogram

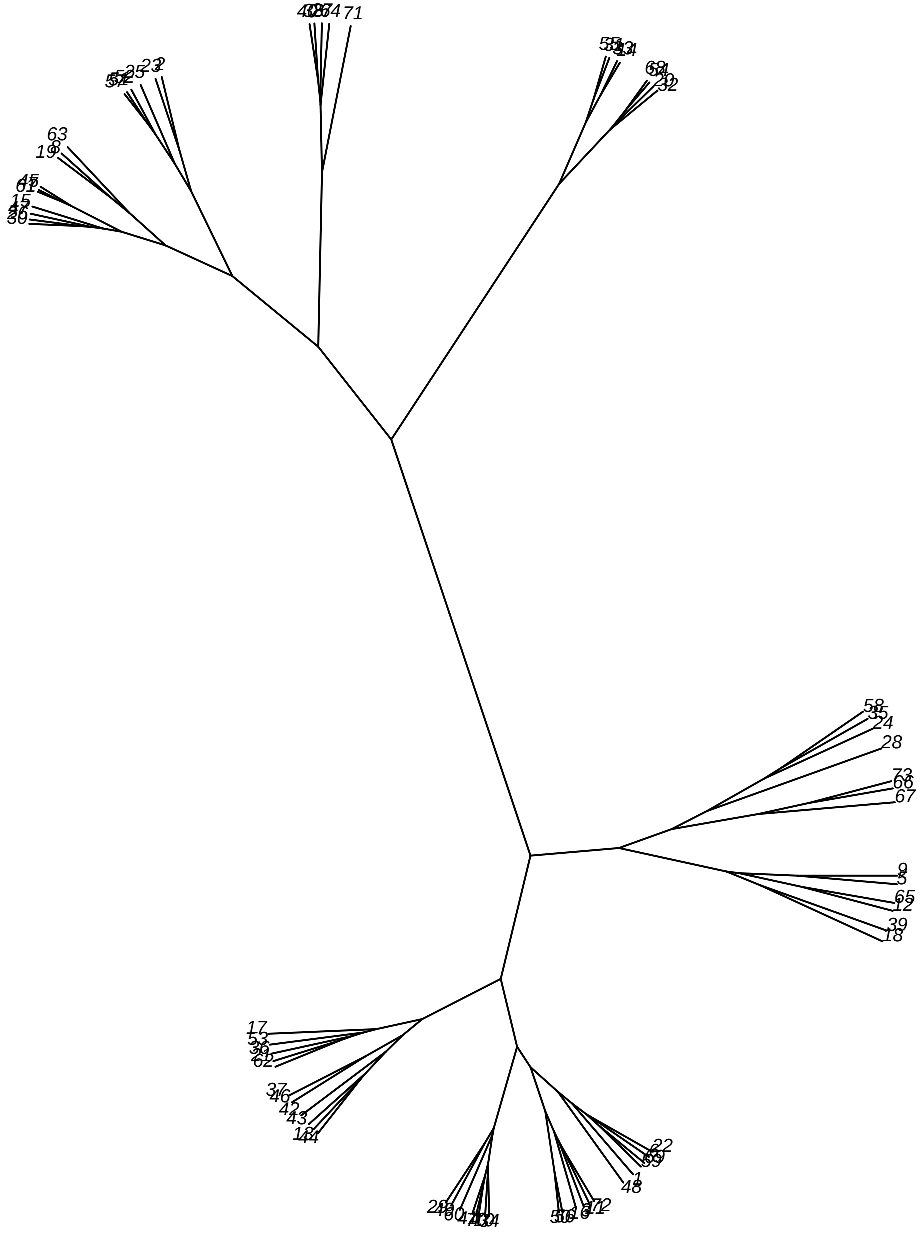

JPN Area 4 Fan Cluster dendrogram

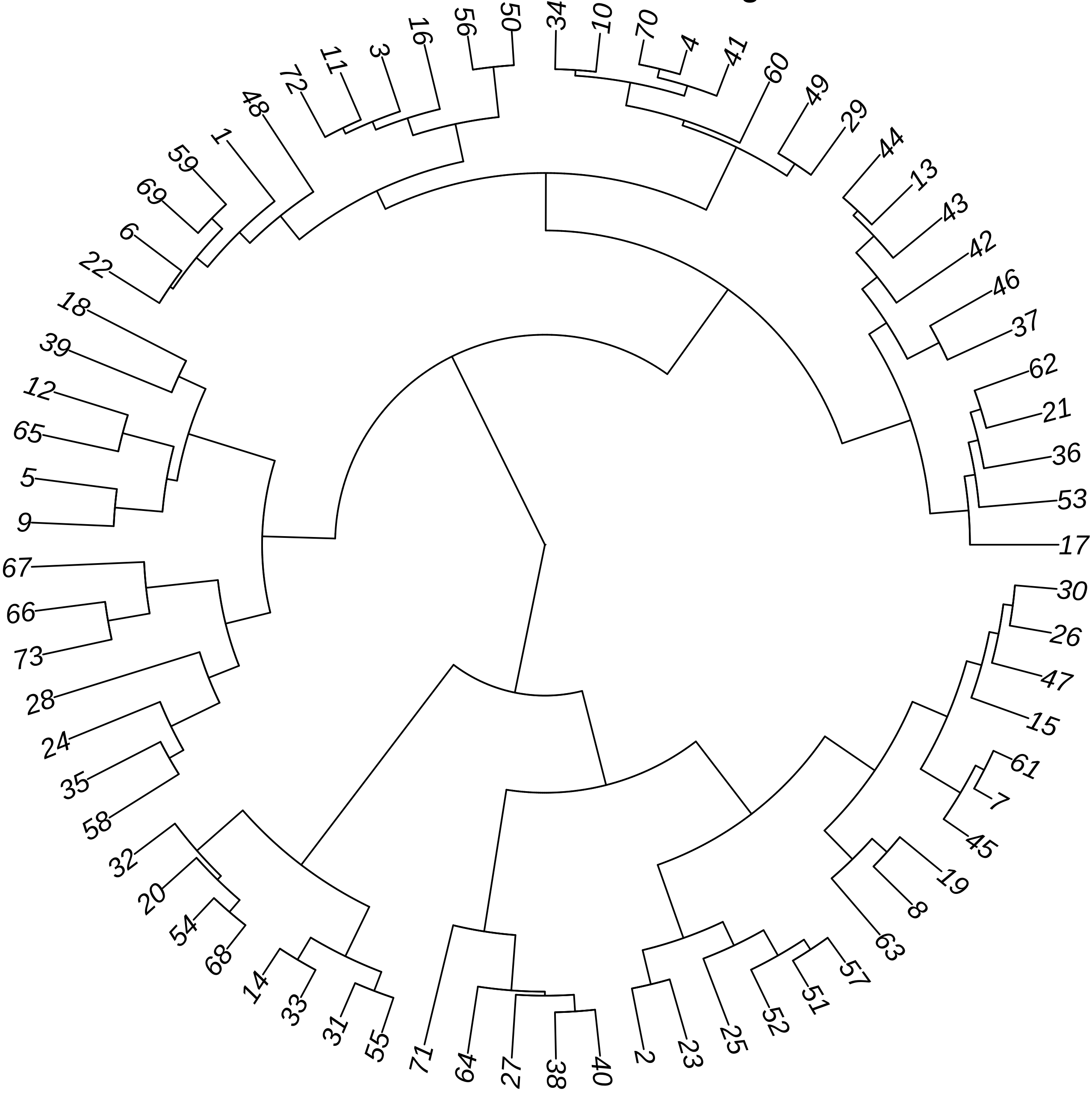

JPN Area 4 at h = 15 : Cluster dendrogram

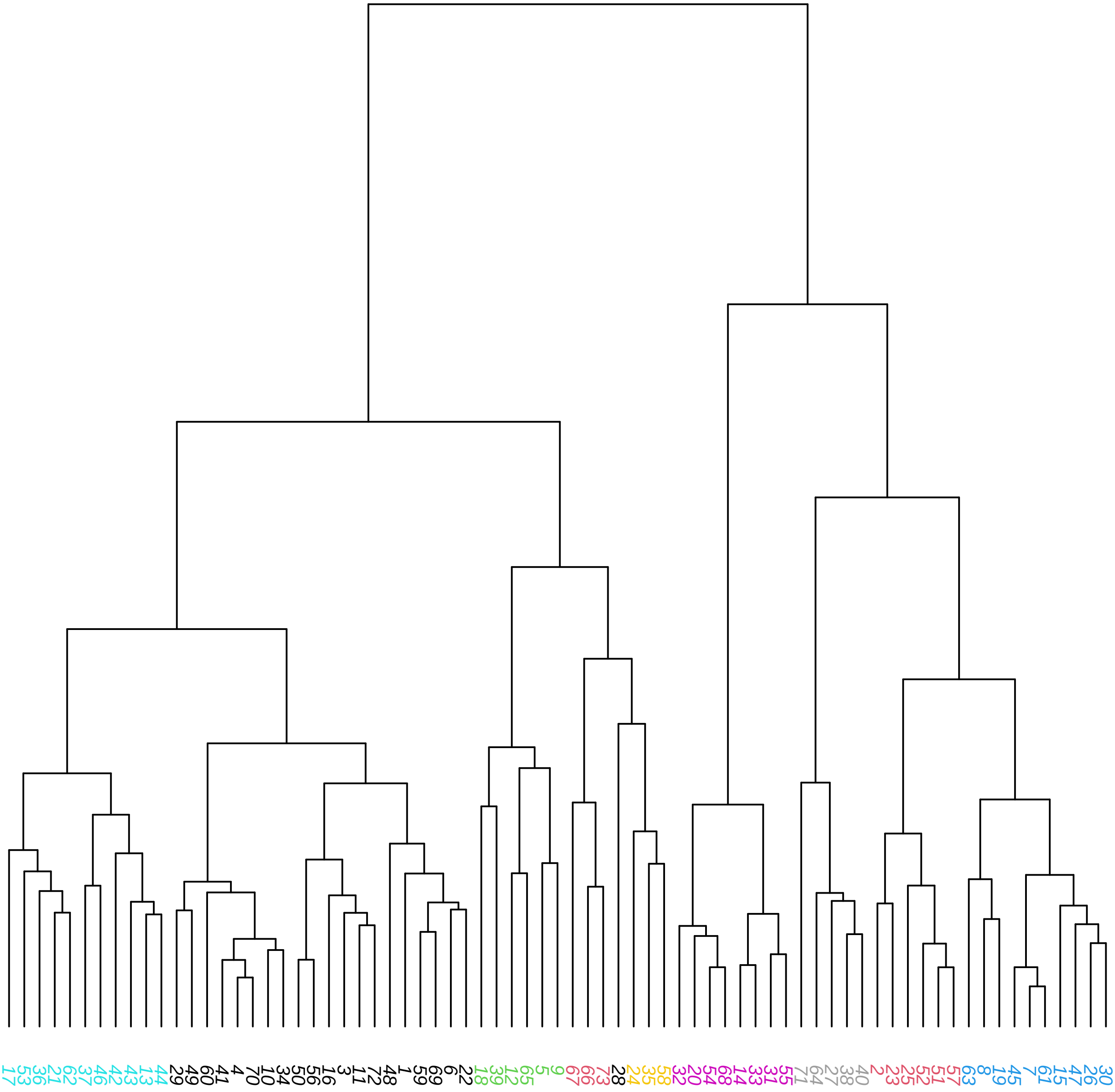

JPN Area 4 at h = 15 : Coloured Unrooted Cluster dendrogram

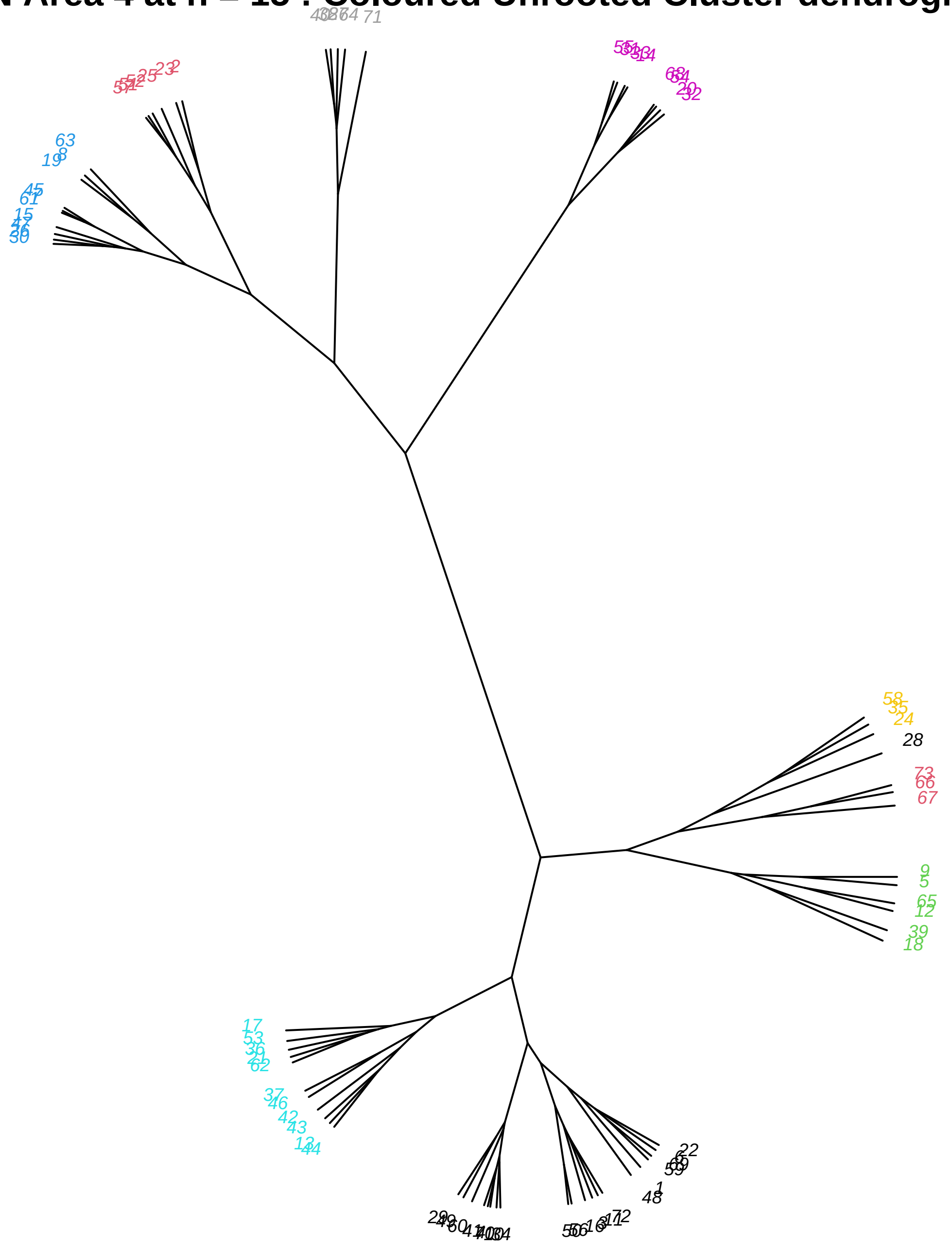

JPN Area 4 at h = 15 : Coloured Fan Cluster dendrogram

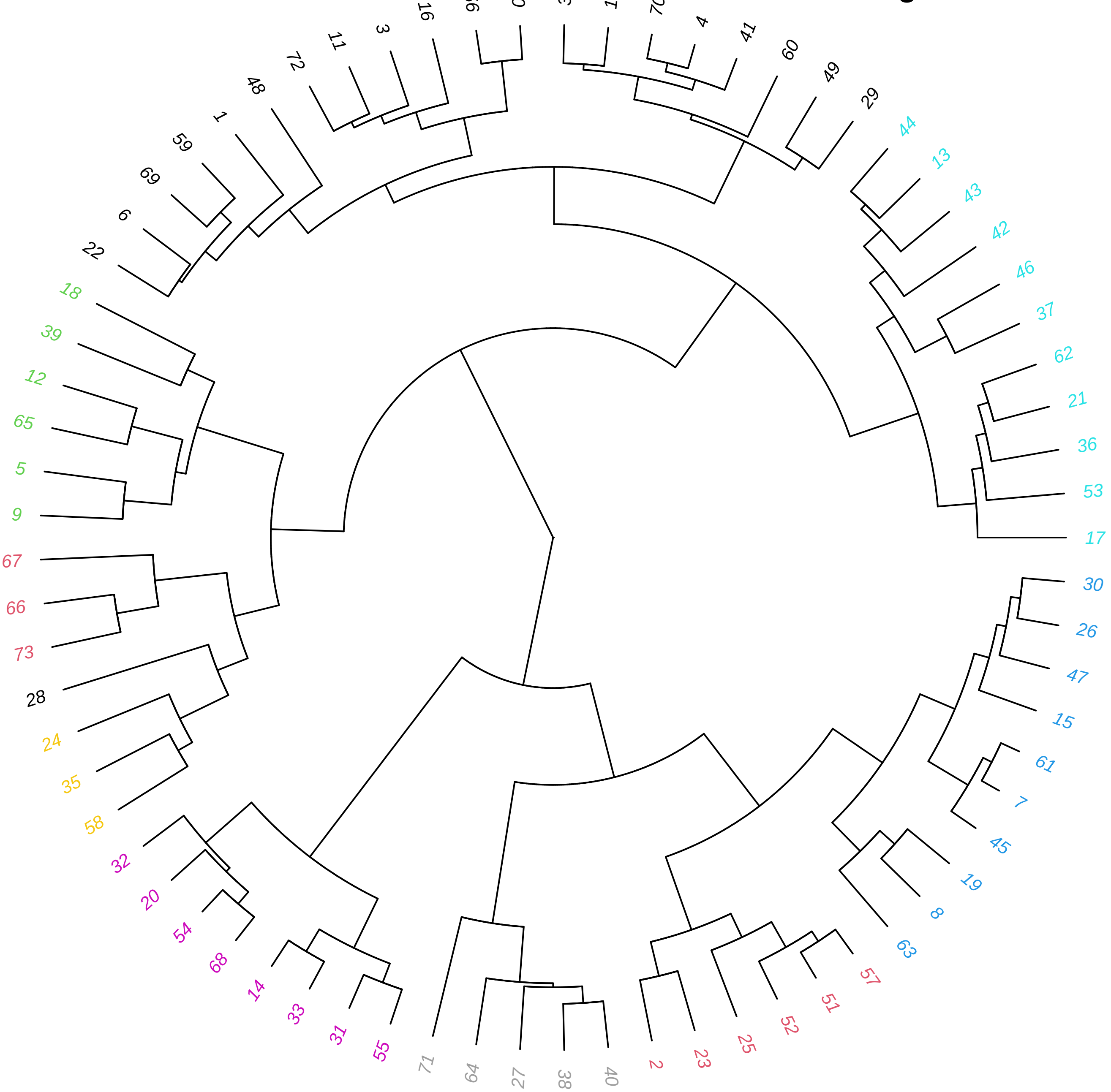

JPN Area 5 with cluster c(20, 20, 50, 50, 50, 20, 50) Cluster dendrogram

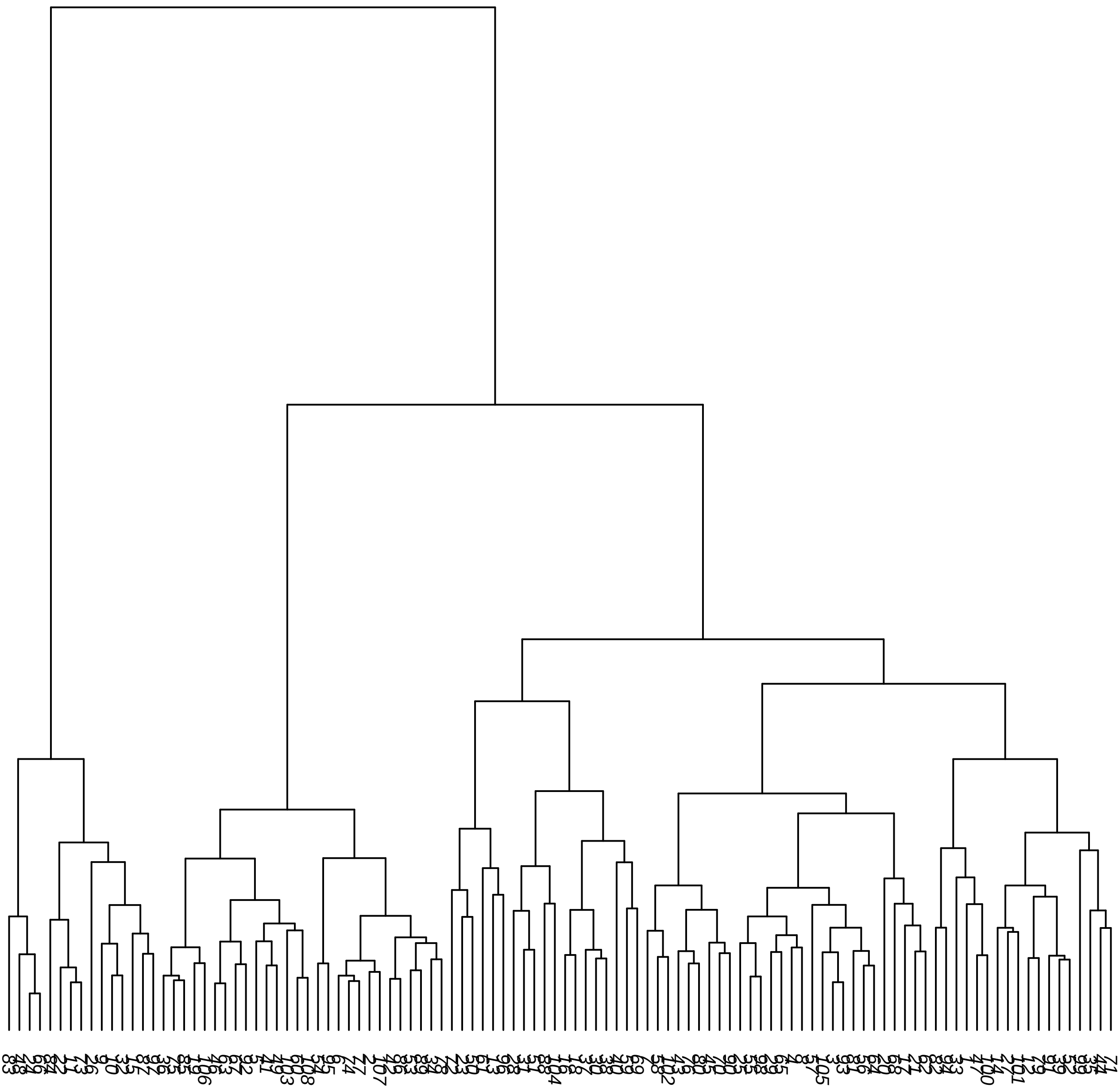

JPN Area 5 Unrooted Cluster dendrogram

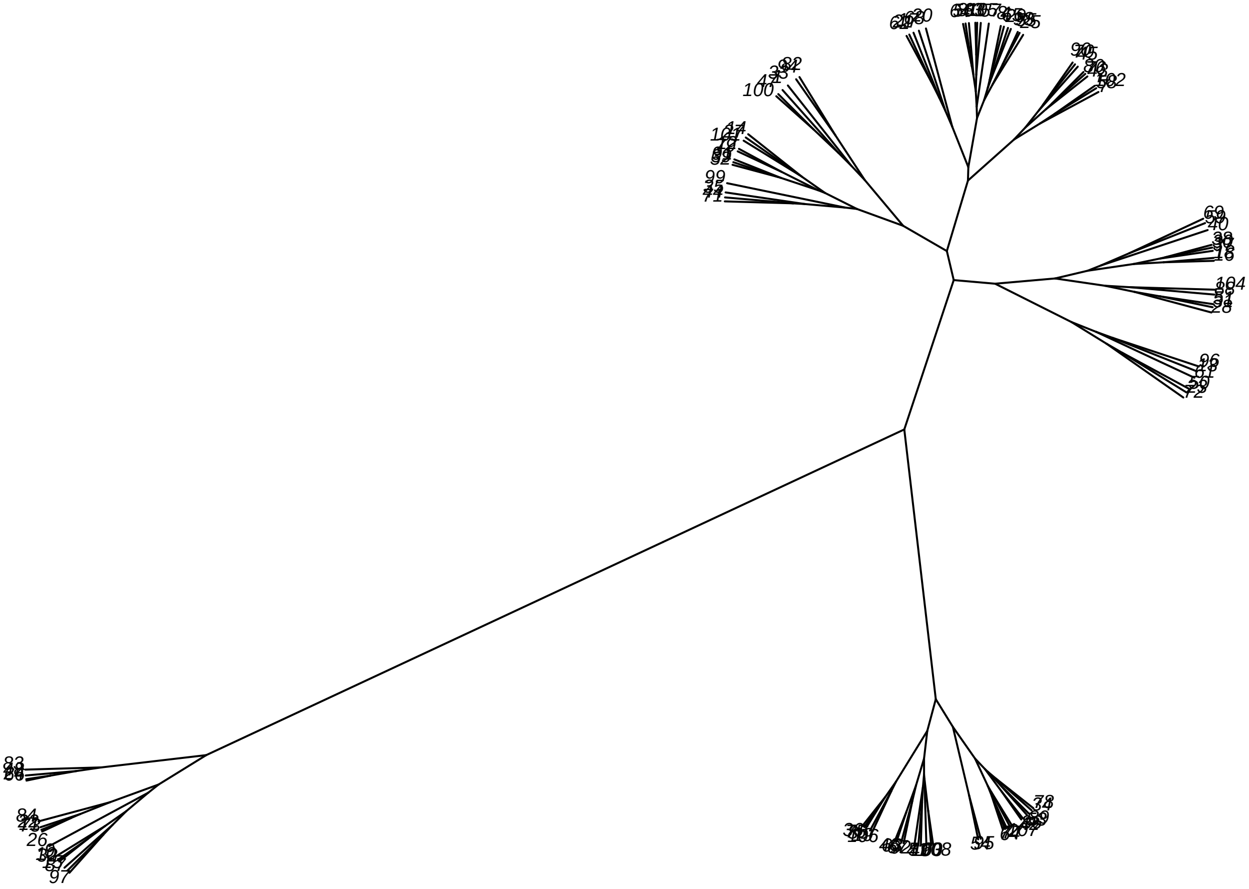

JPN Area 5 Fan Cluster dendrogram

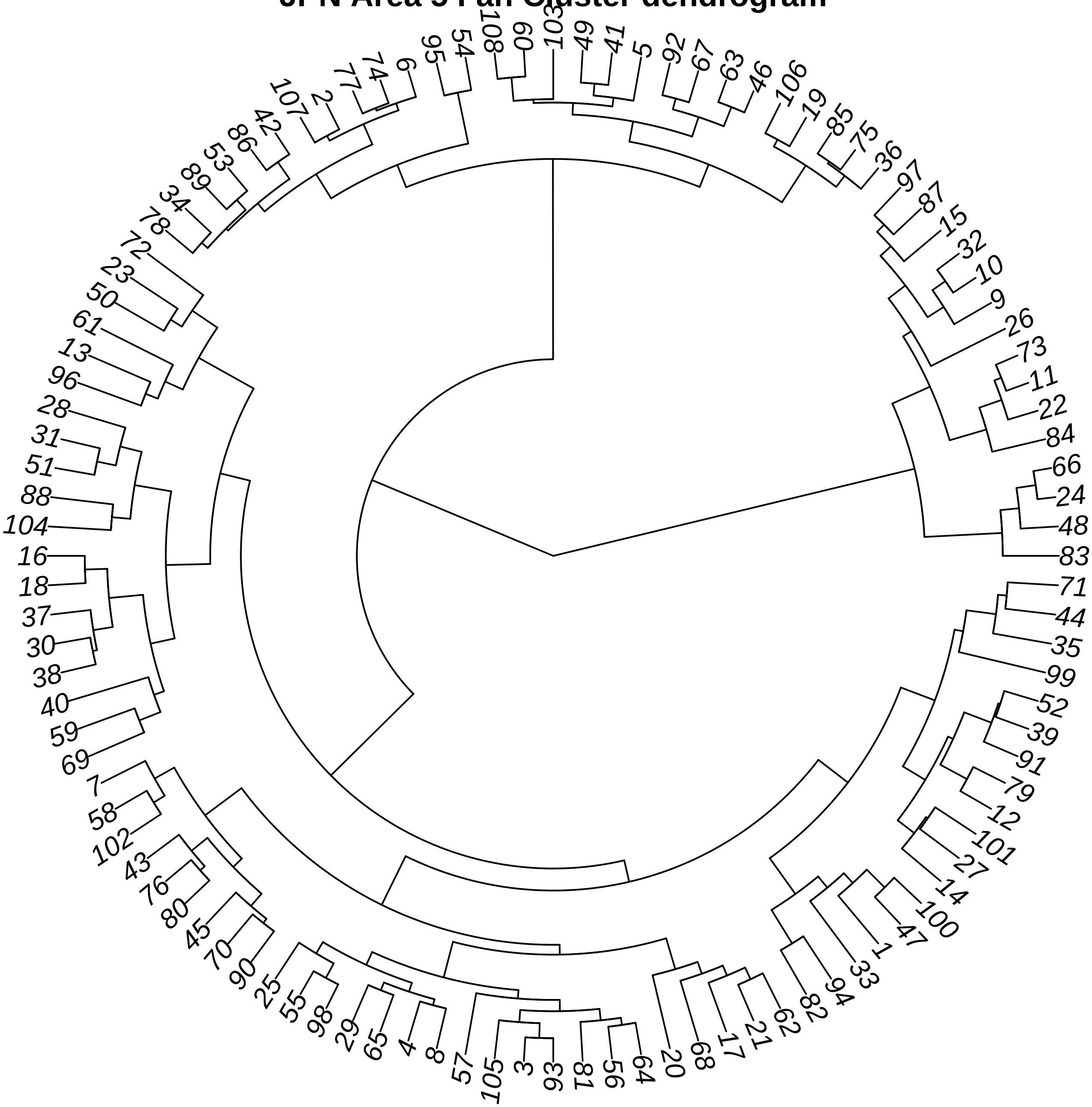

JPN Area 5 at h = 15 : Cluster dendrogram

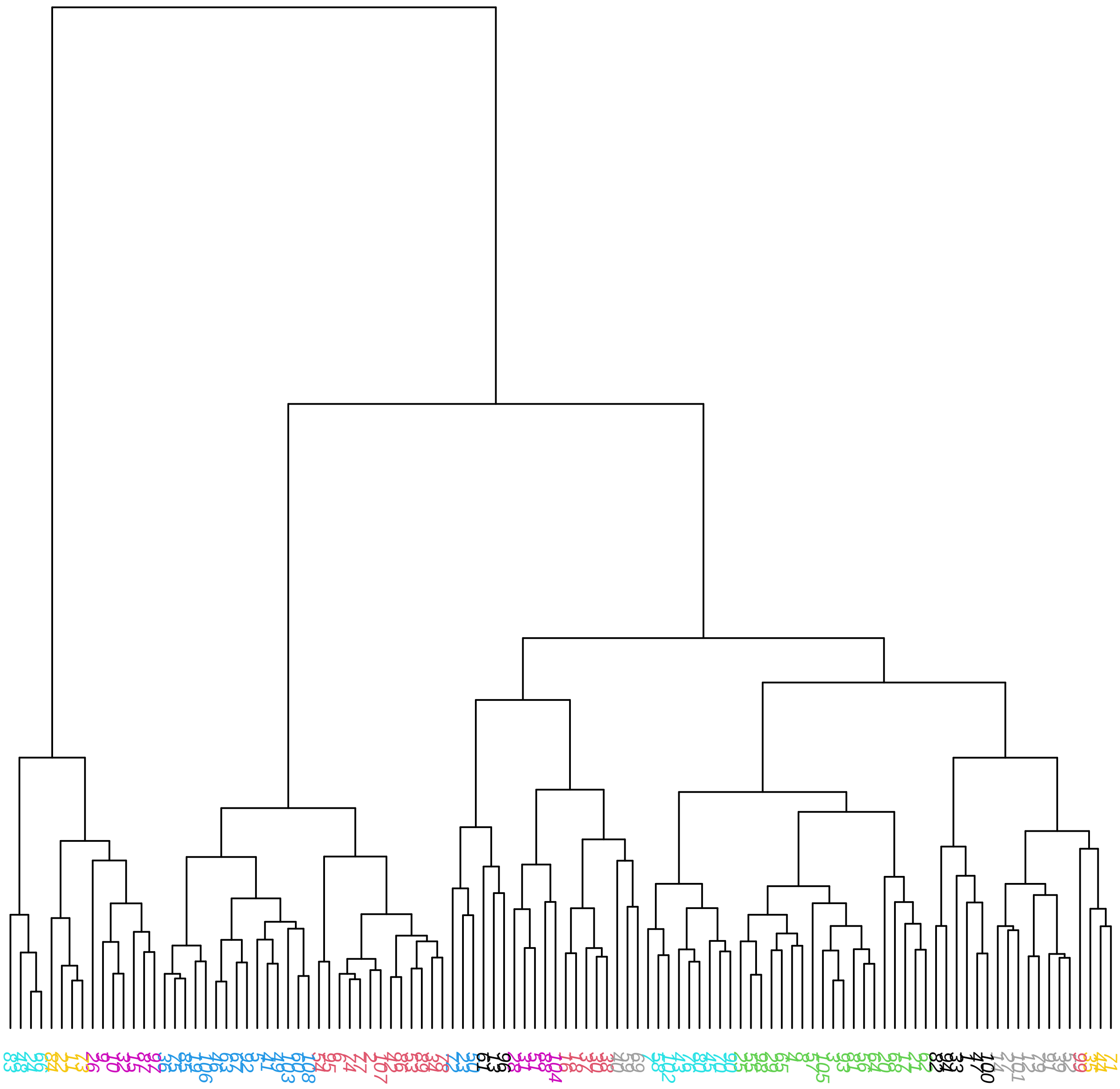

JPN Area 5 at h = 15 : Coloured Unrooted Cluster dendrogram

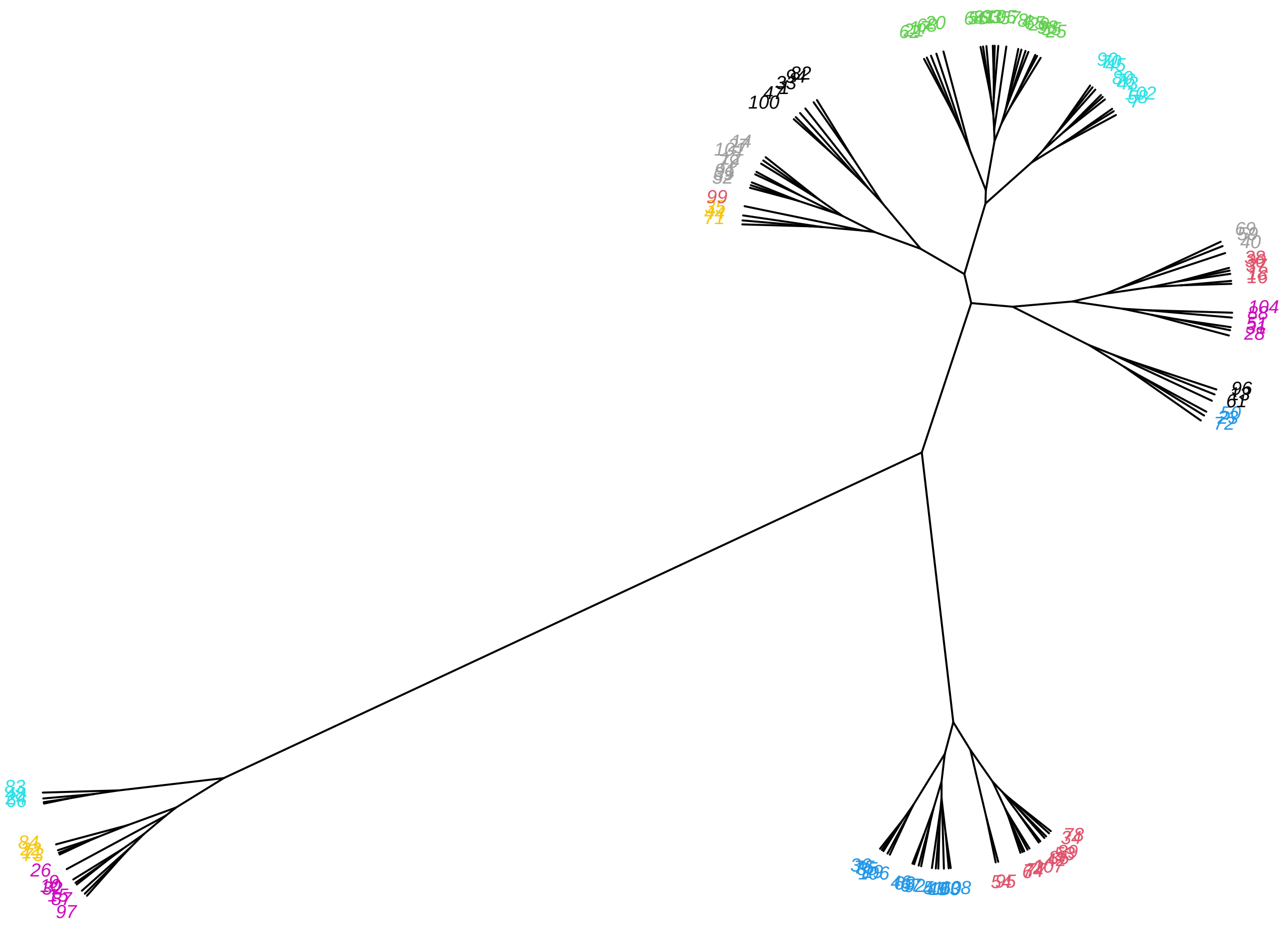

JPN Area 5 at h = 15 : Coloured Fan Cluster dendrogram

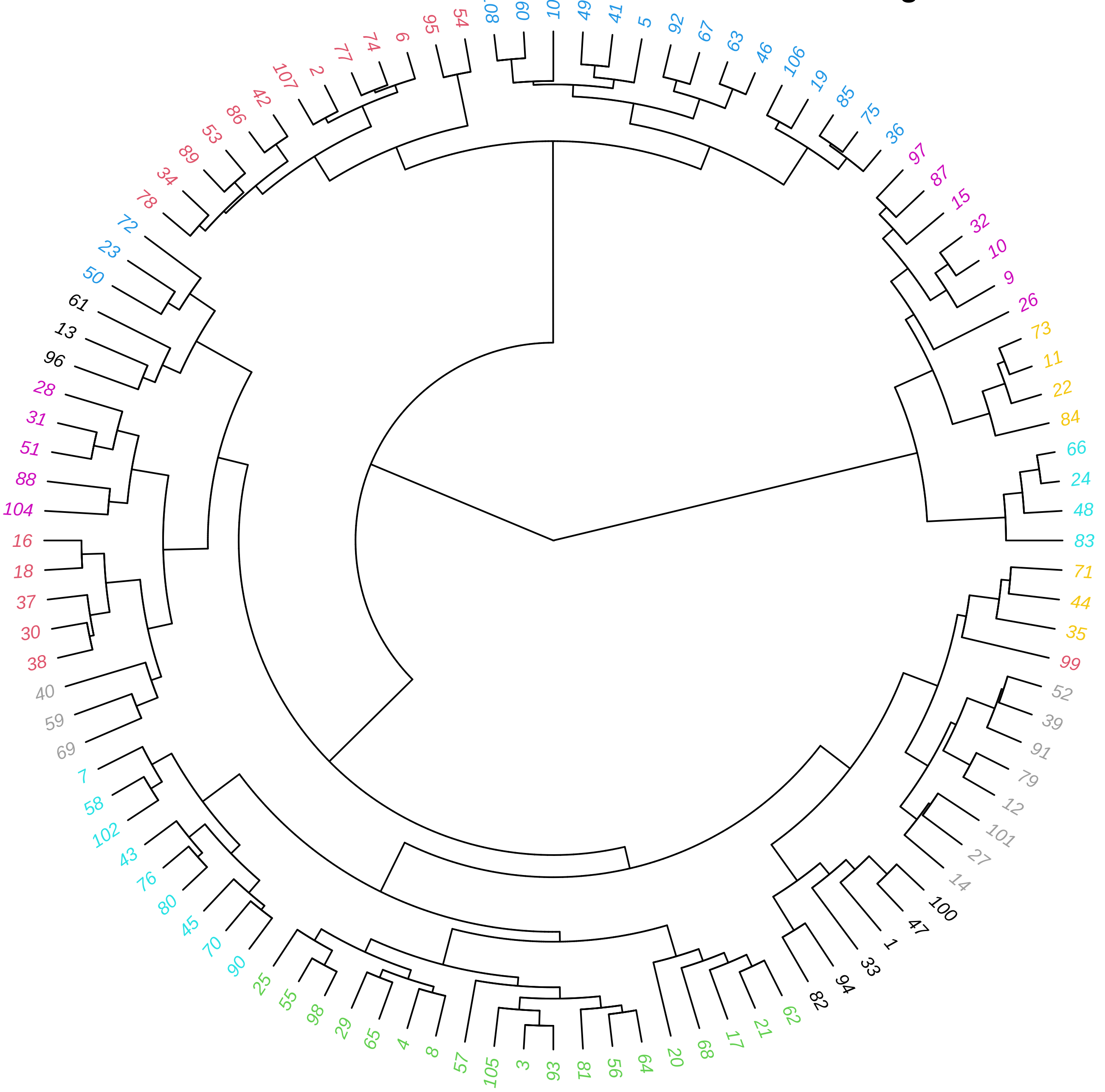

JPN Area 6 with cluster c(20, 20, 50, 50, 50, 20, 50) Cluster dendrogram

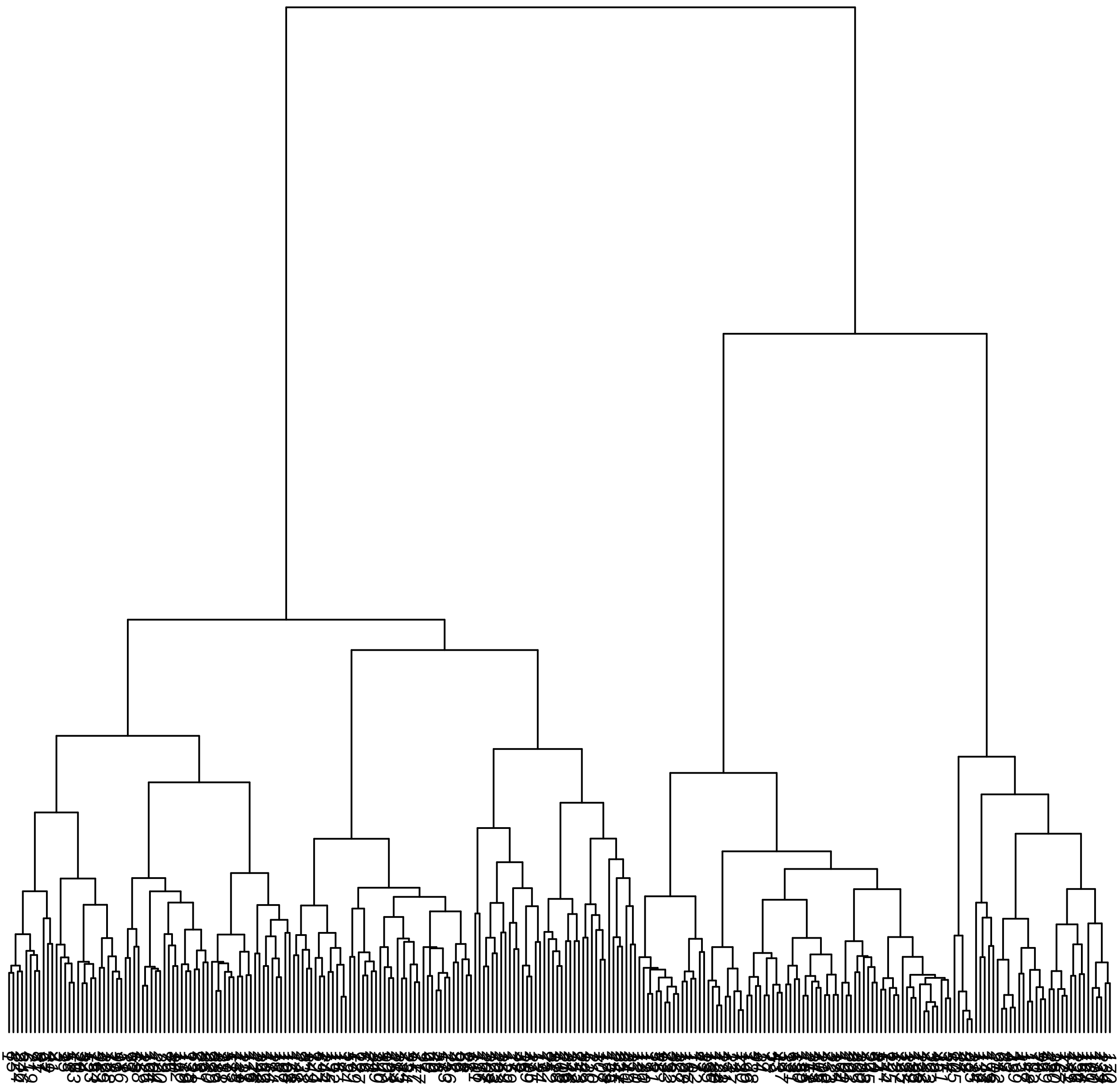

JPN Area 6 Unrooted Cluster dendrogram

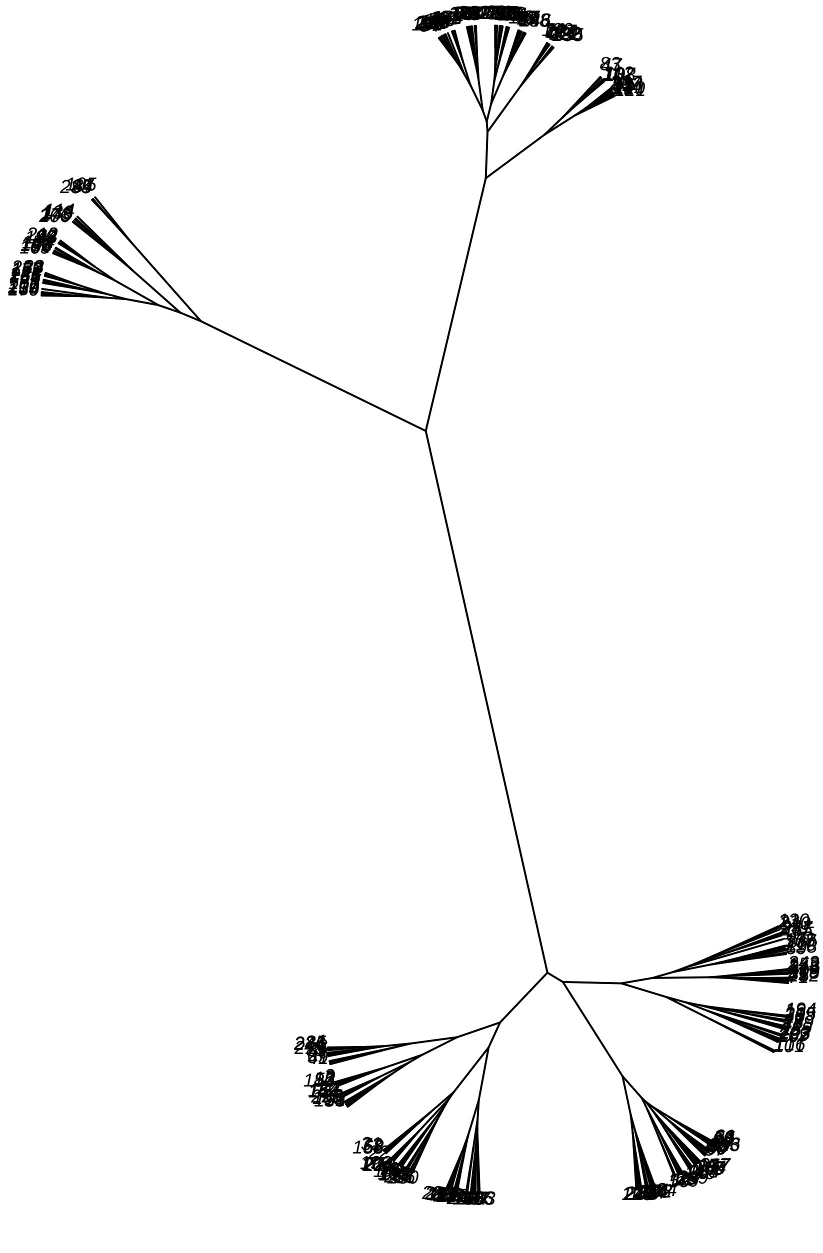

JPN Area 6 Fan Cluster dendrogram

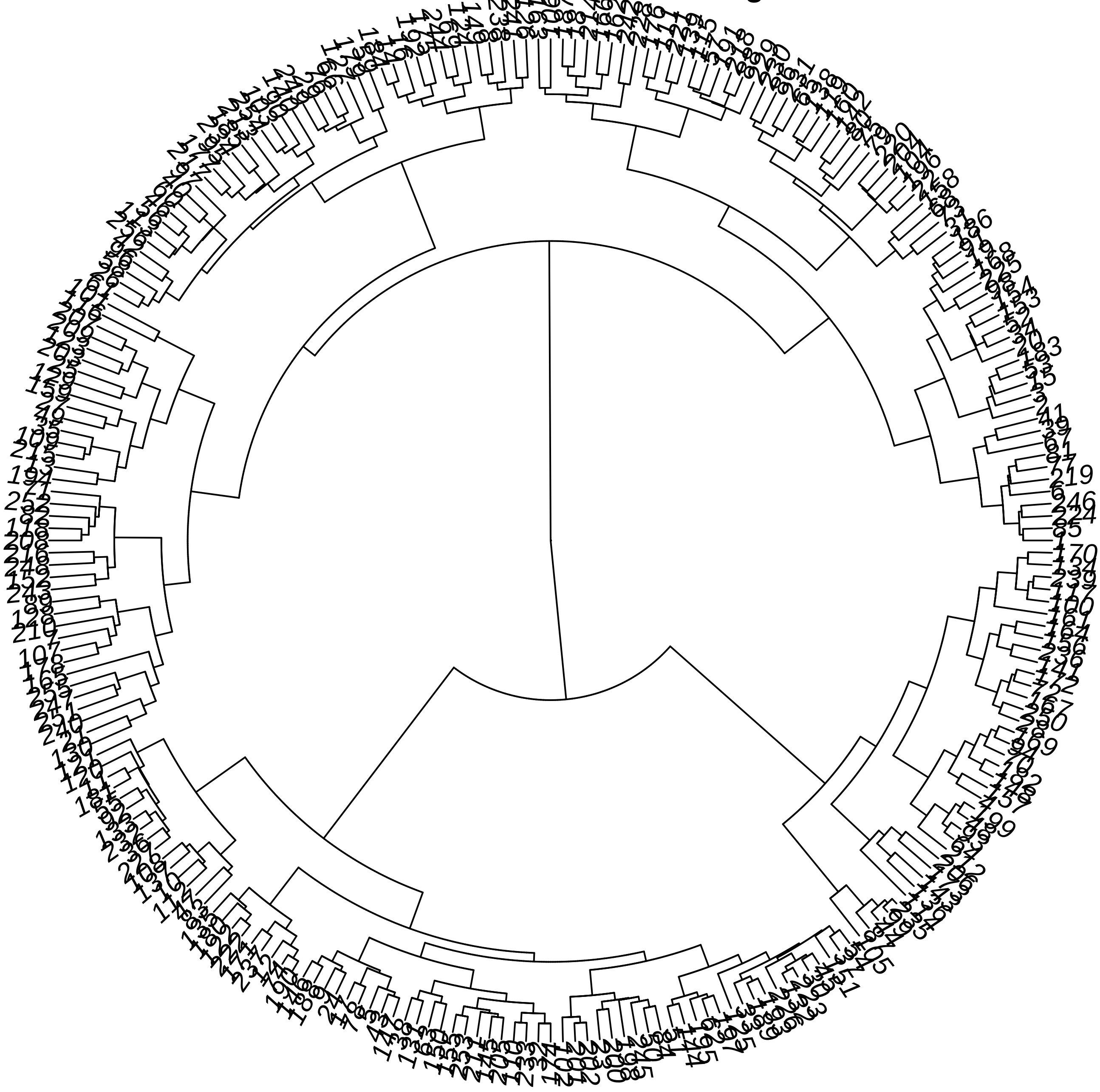

JPN Area 6 at h = 15 : Cluster dendrogram

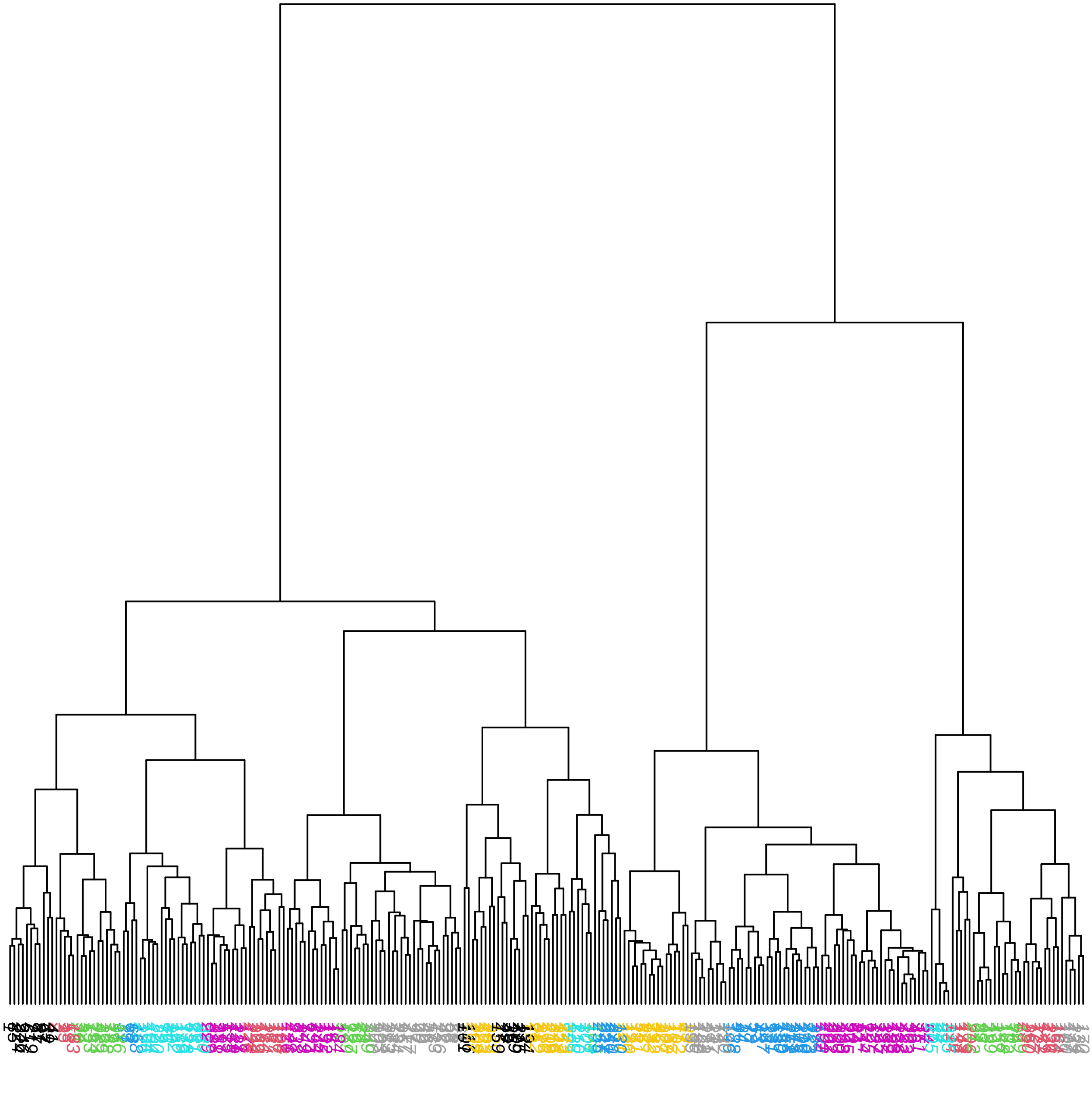

JPN Area 6 at h = 15 : Coloured Unrooted Cluster dendrogram

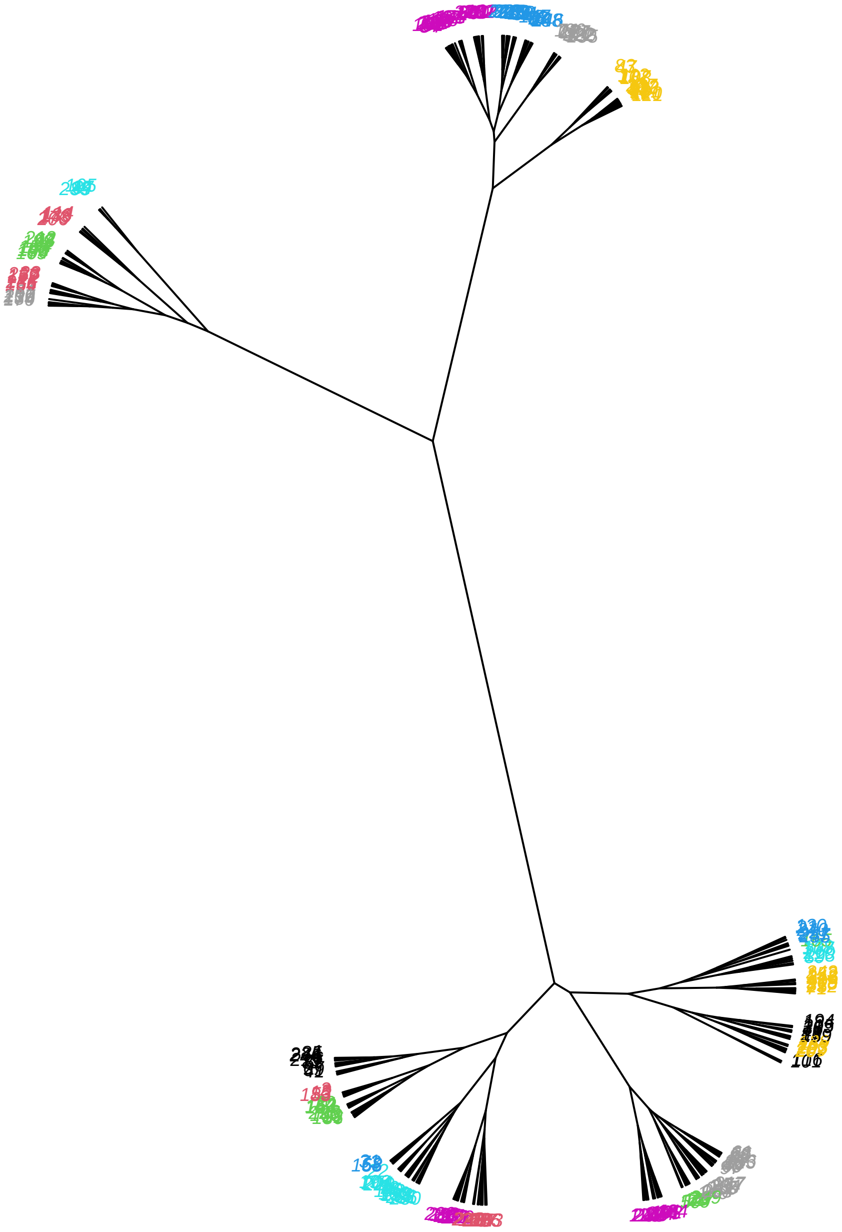

JPN Area 6 at h = 15 : Coloured Fan Cluster dendrogram

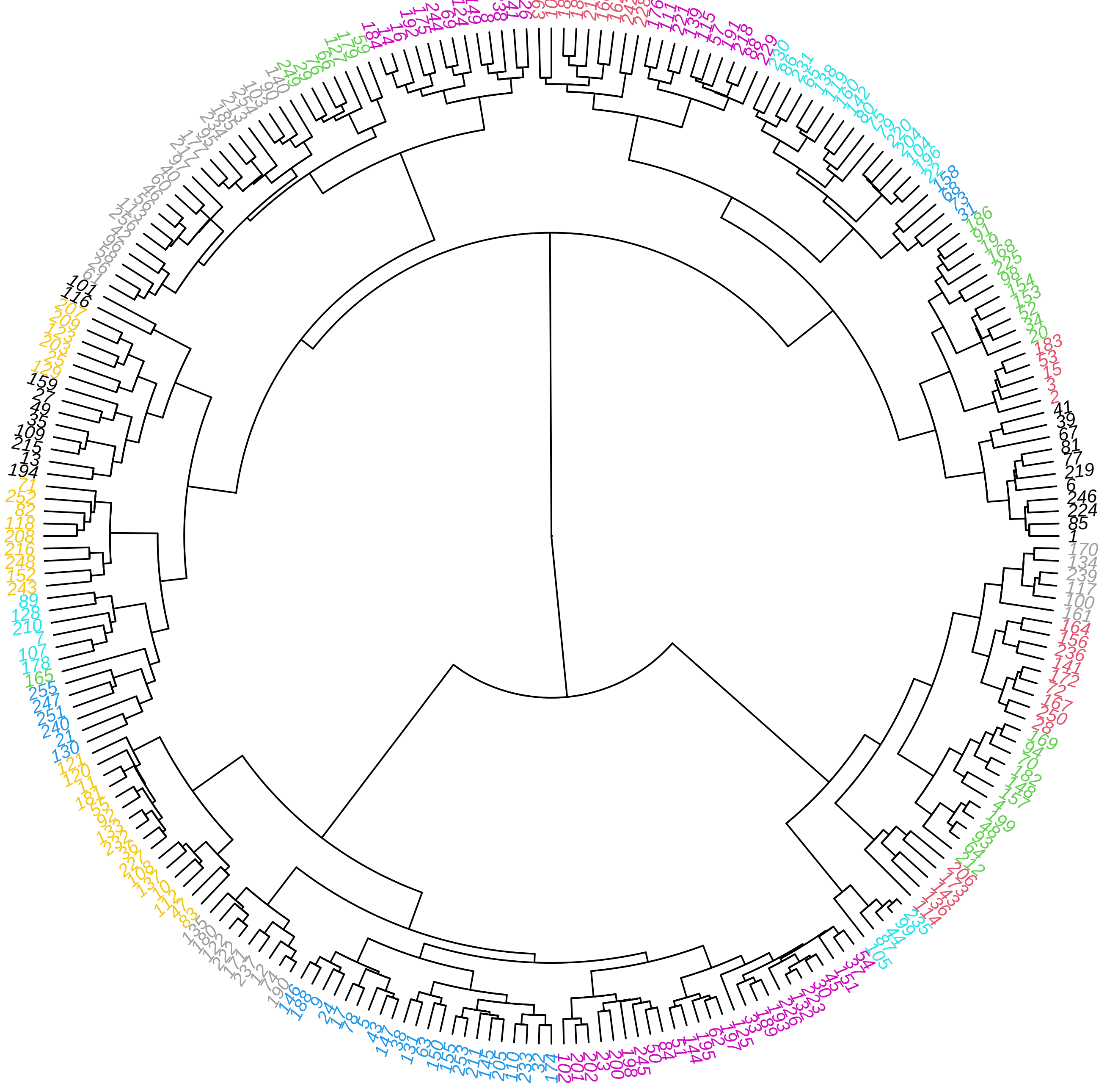

JPN Area 7 with cluster c(20, 20, 50, 50, 50, 20, 50) Cluster dendrogram

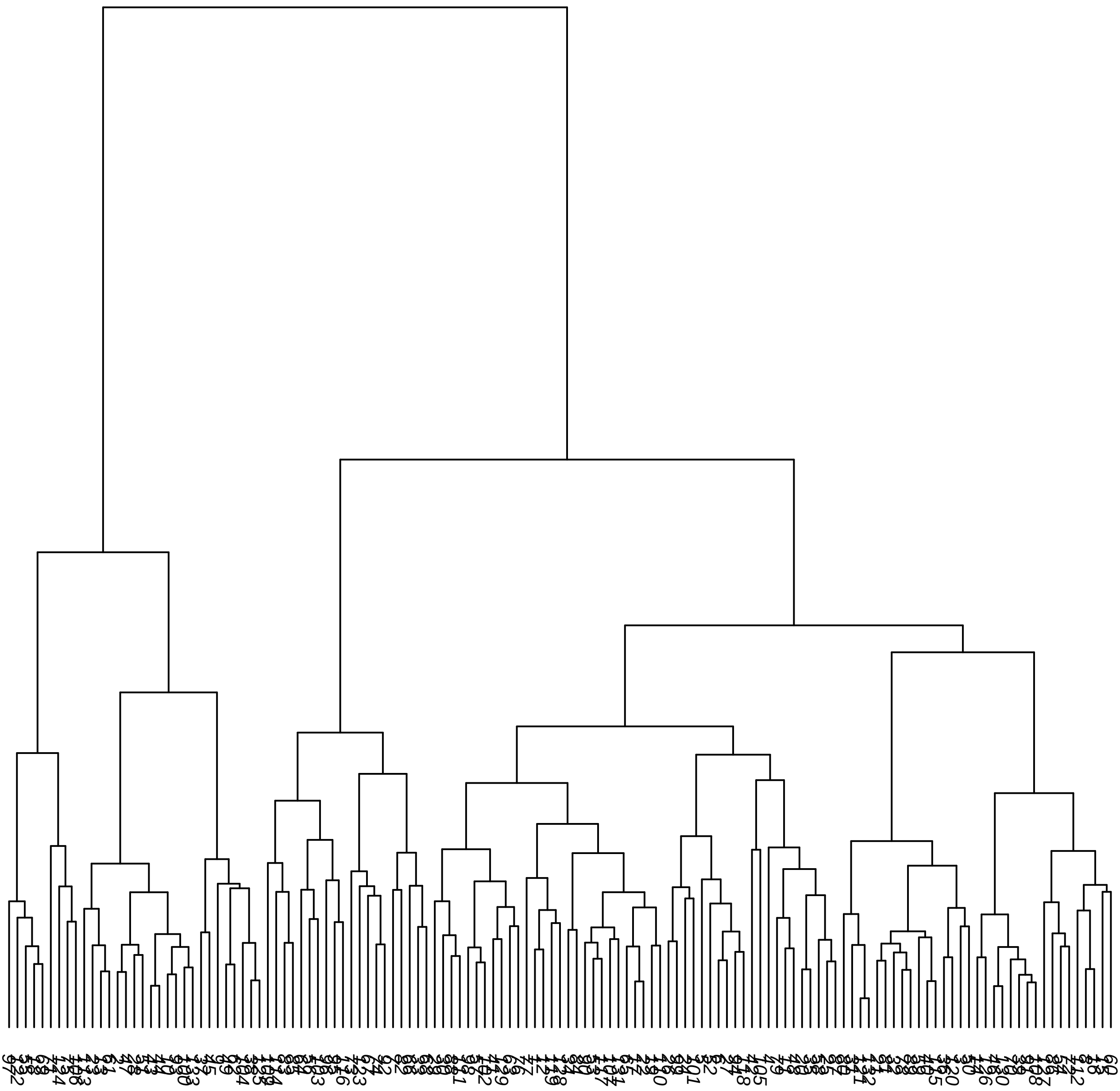

JPN Area 7 Unrooted Cluster dendrogram

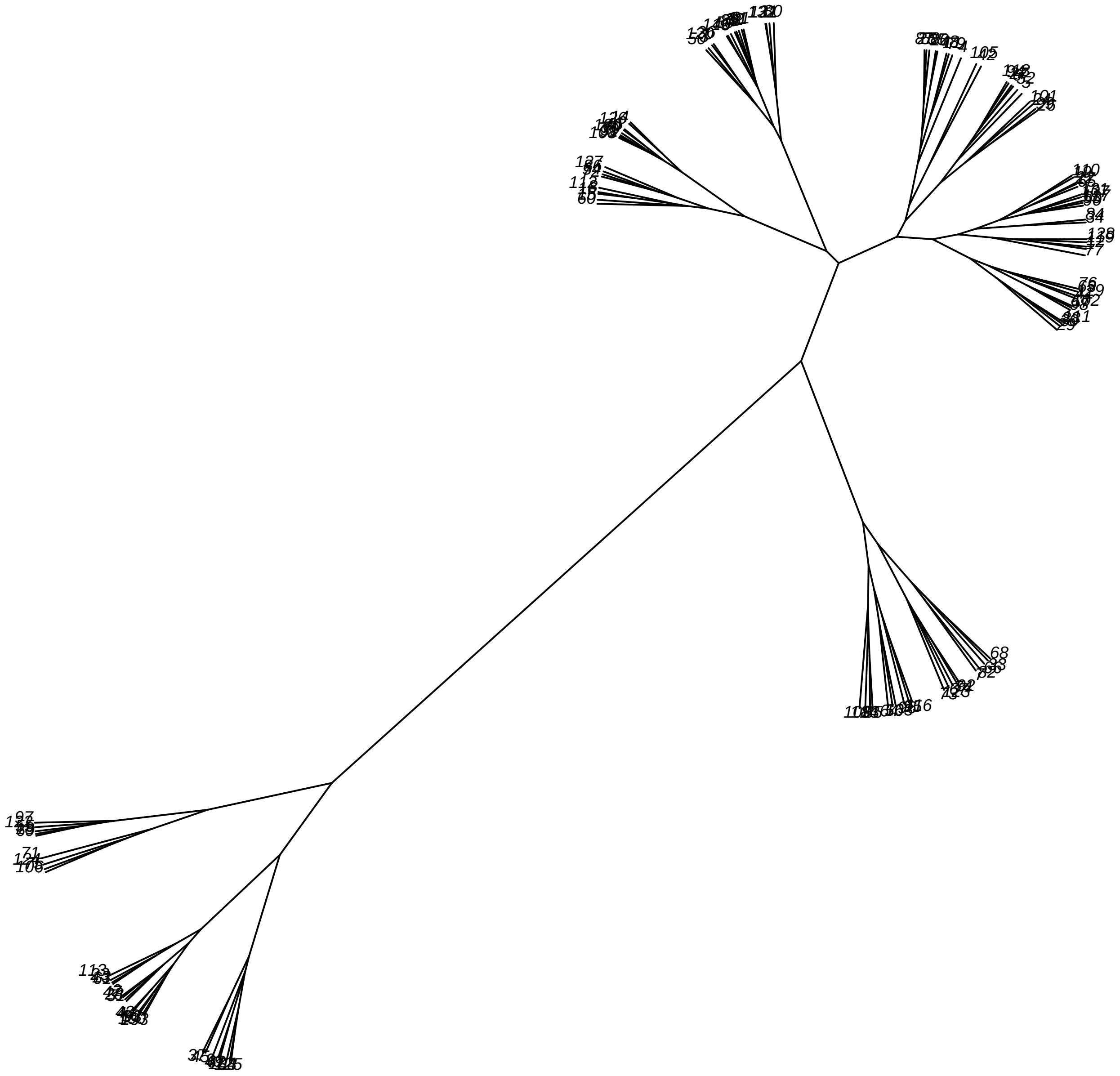

JPN Area 7 Fan Cluster dendrogram

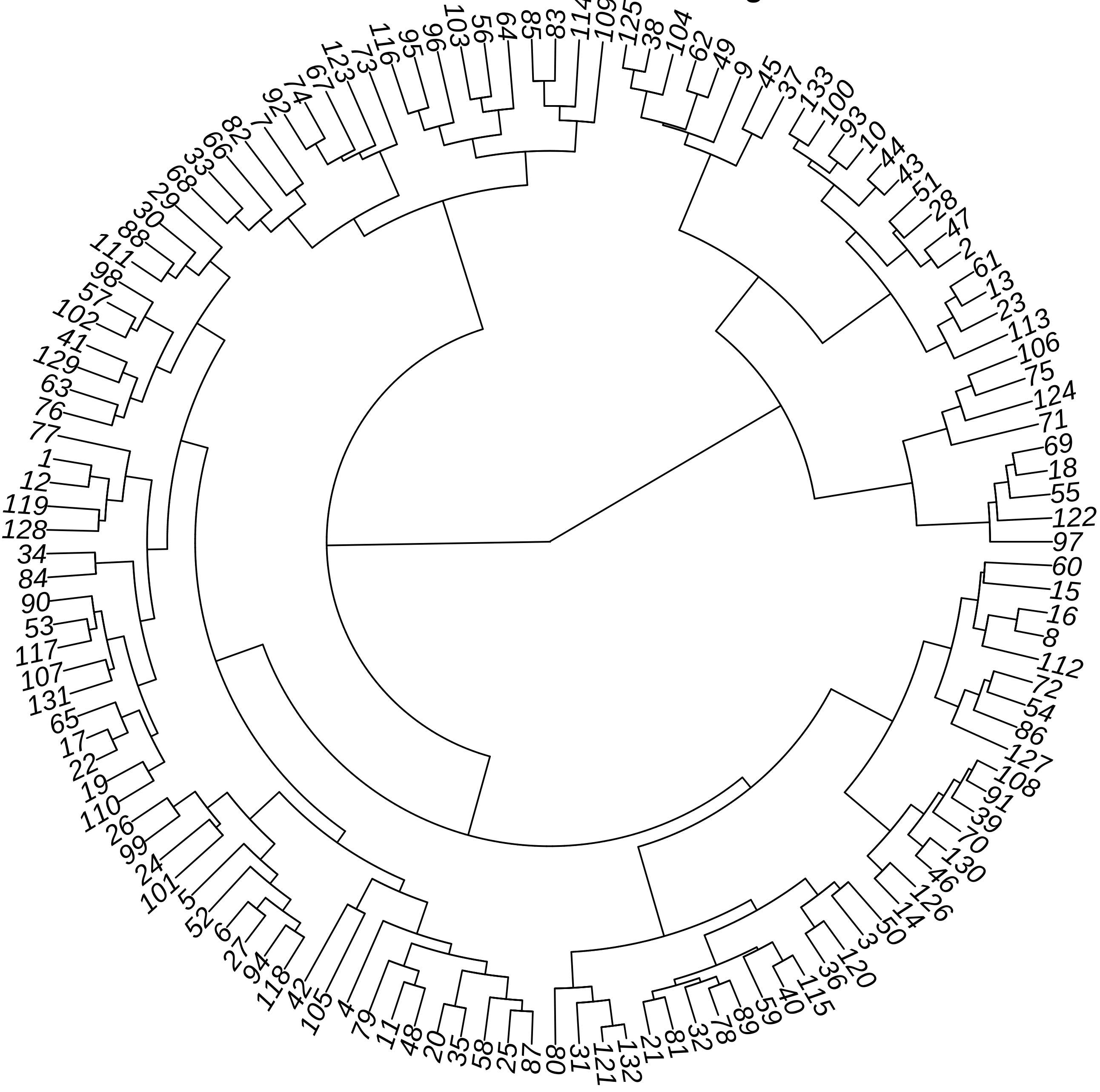

JPN Area 7 at h = 15 : Cluster dendrogram

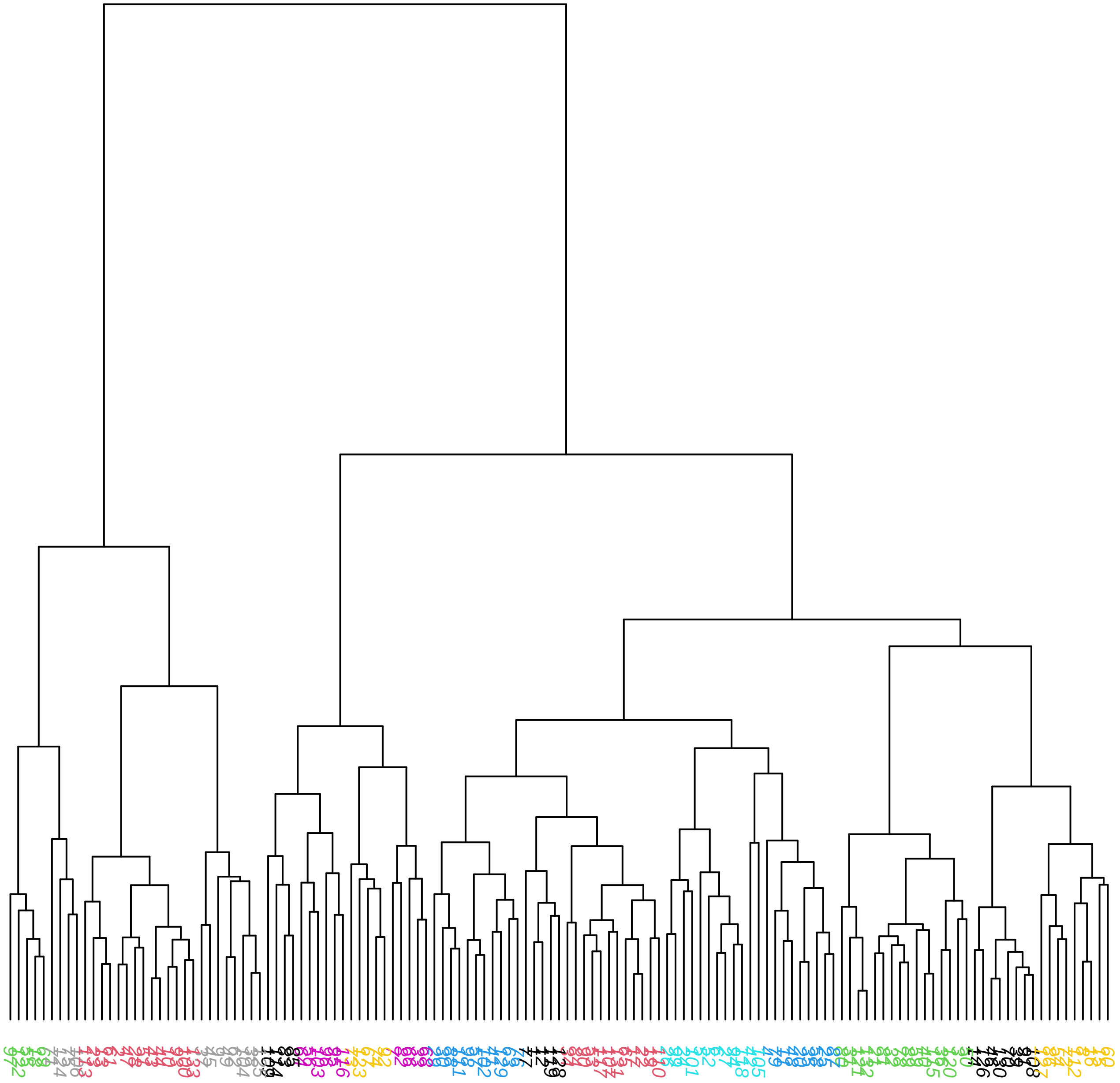

JPN Area 7 at h = 15 : Coloured Unrooted Cluster dendrogram

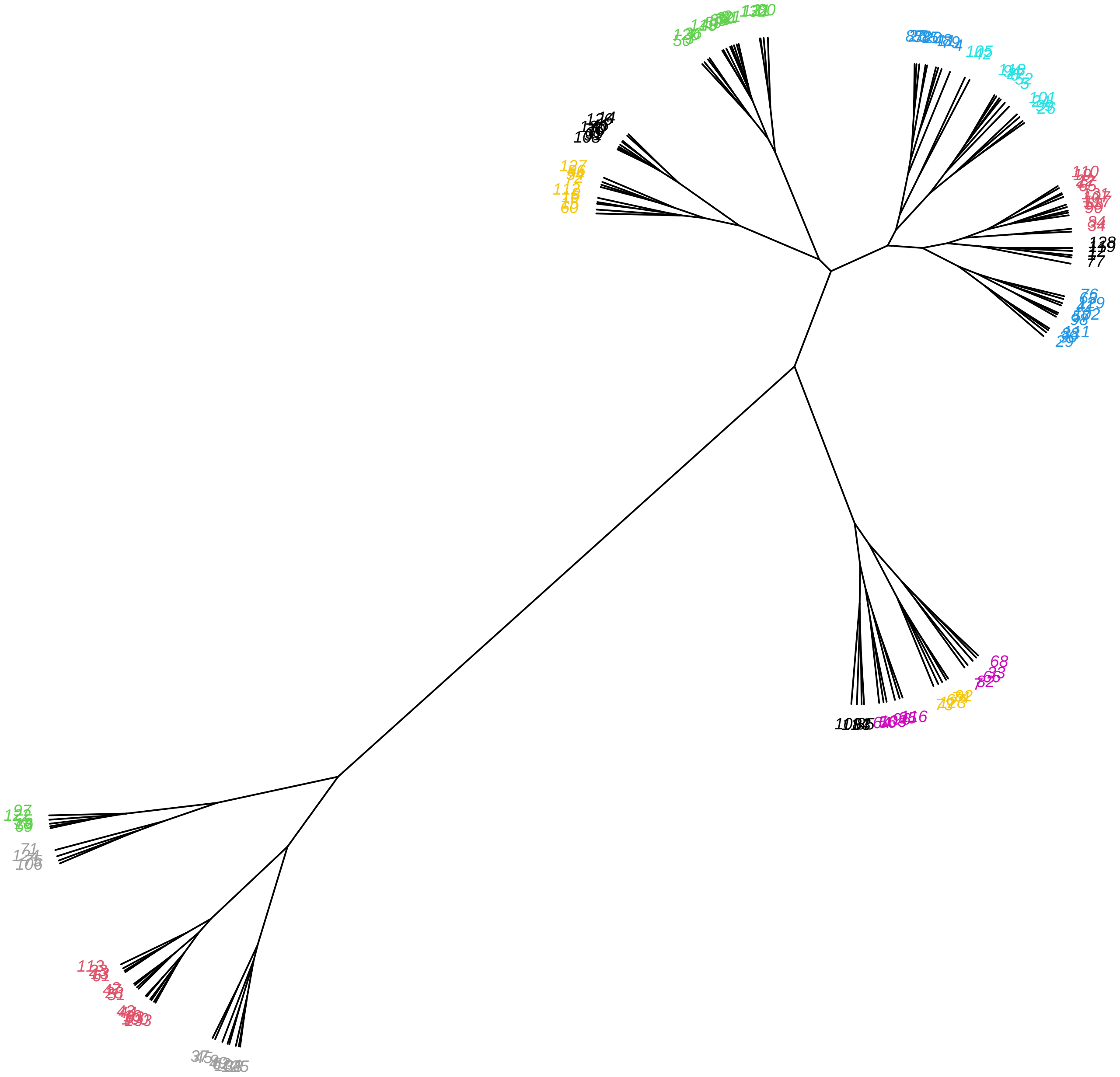

JPN Area 7 at h = 15 : Coloured Fan Cluster dendrogram

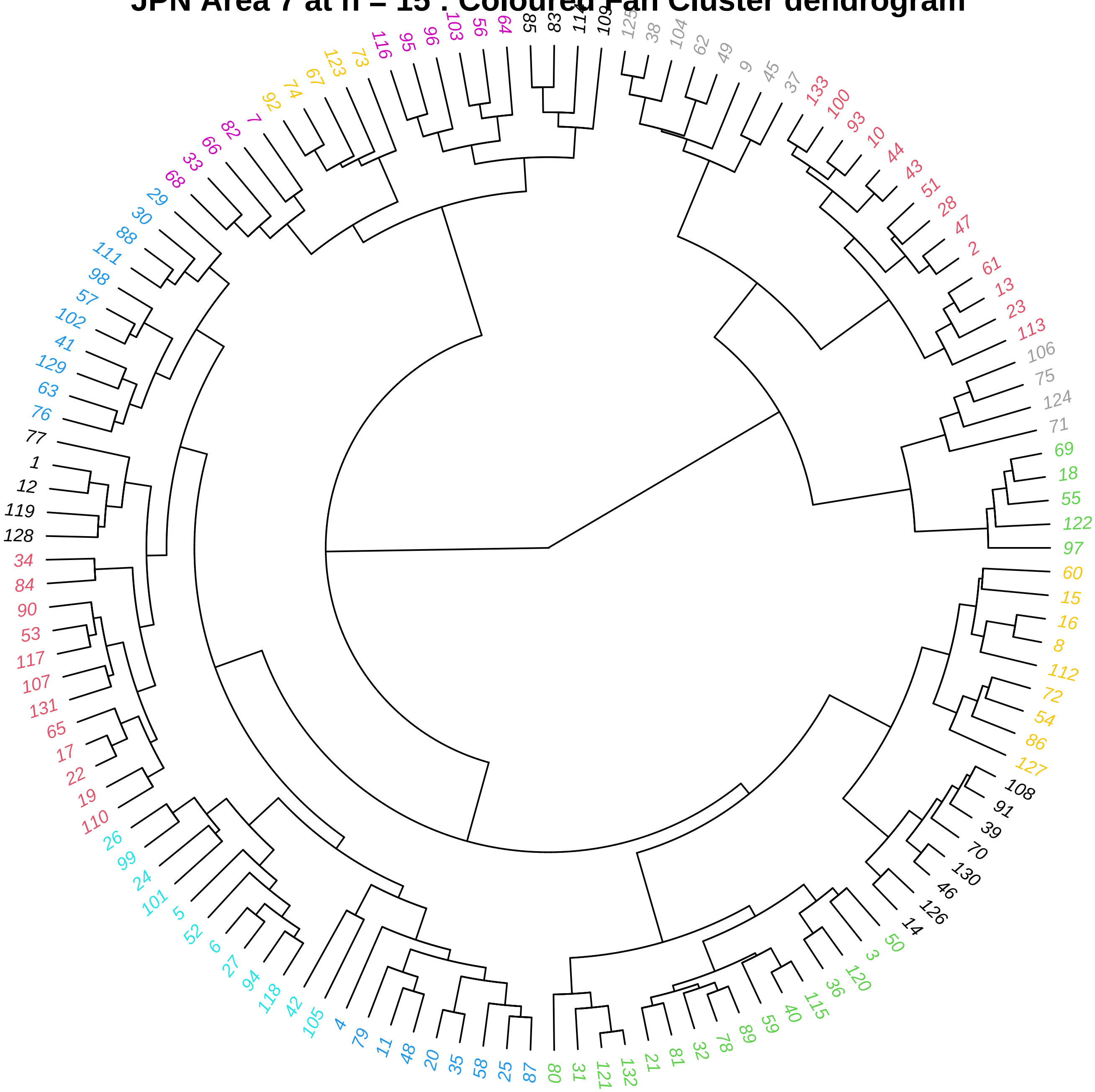

Supplement: S6 Appendix — (PDF) [file pone.0272848.s006.pdf]
