# Supplementary material for: Classifying ball trajectories in invasion sports using dynamic time warping: A basketball case study
Source: PLoS One. 2022 Oct 20;17(10):e0272848. doi: 10.1371/journal.pone.0272848 (PMC9584368; doi:10.1371/journal.pone.0272848)

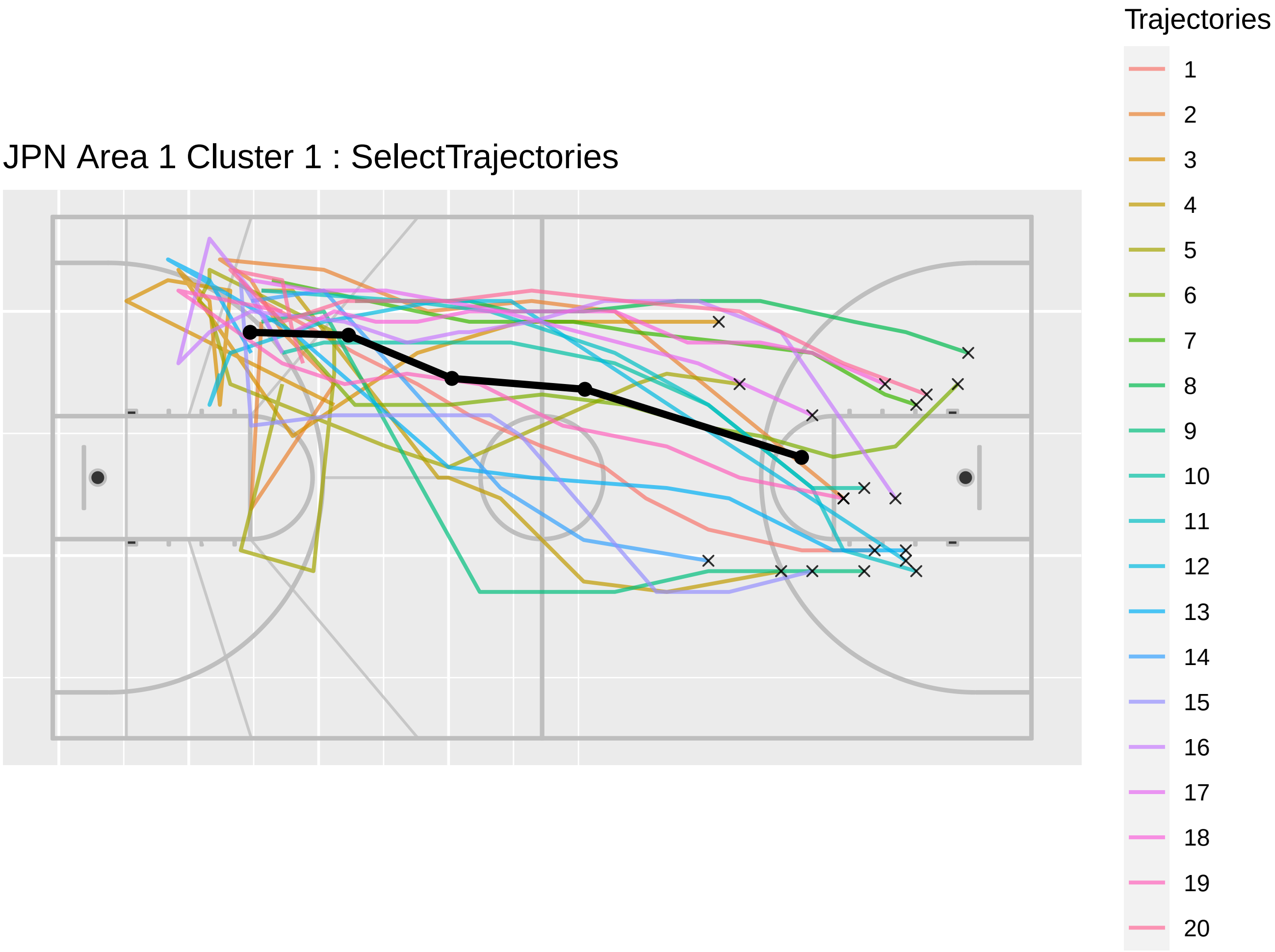
JPN Area 1 Cluster 1 : SelectTrajectories
Trajectories
1
2
3
4
5
6
7
8
9
10
11
12
13
14
15
16
17
18
19
20

JPN Area 1 Cluster 2 : SelectTrajectories

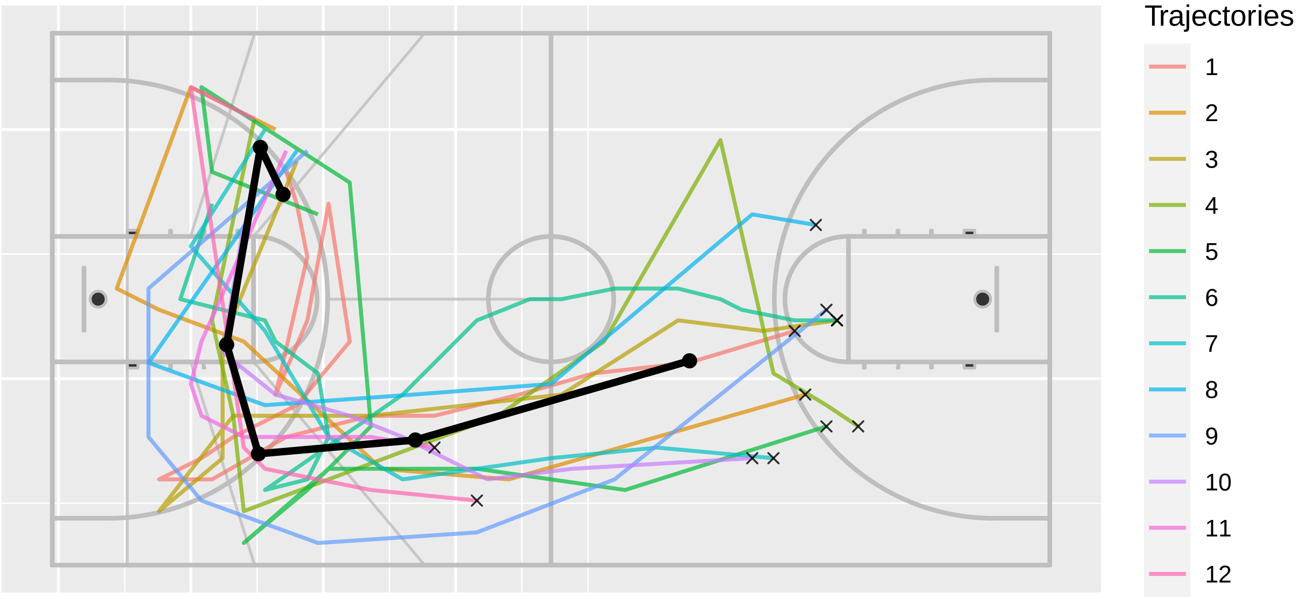

JPN Area 1 Cluster 3 : SelectTrajectories

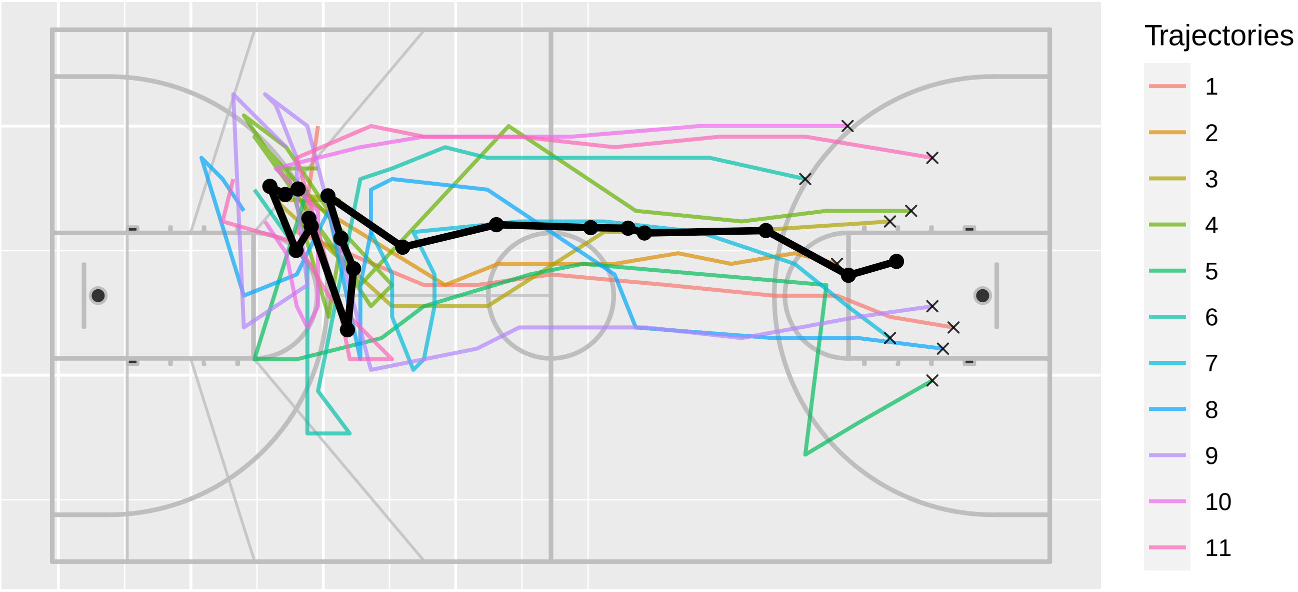

JPN Area 1 Cluster 4 : SelectTrajectories

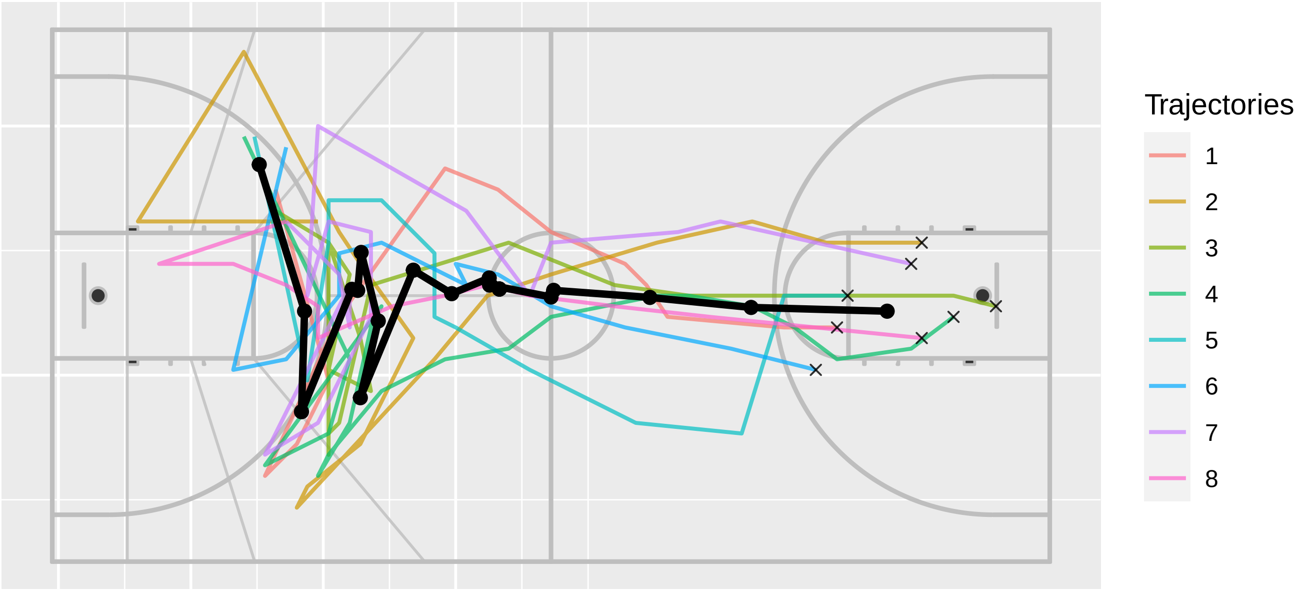

JPN Area 1 Cluster 5 : SelectTrajectories

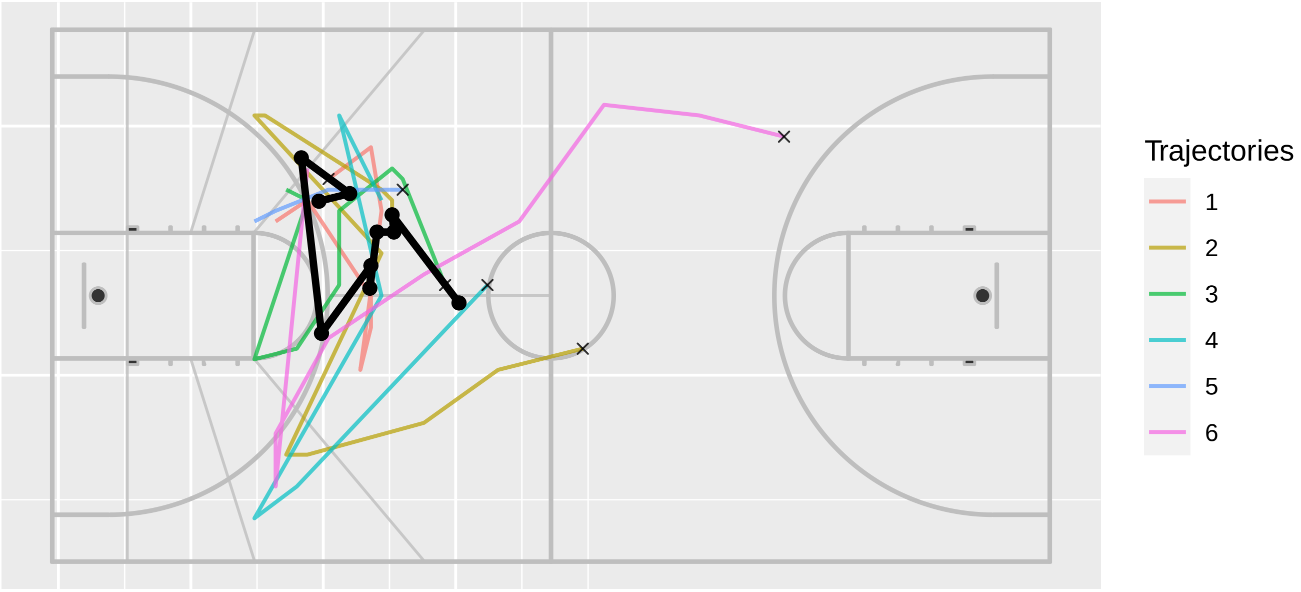

JPN Area 1 Cluster 6 : SelectTrajectories

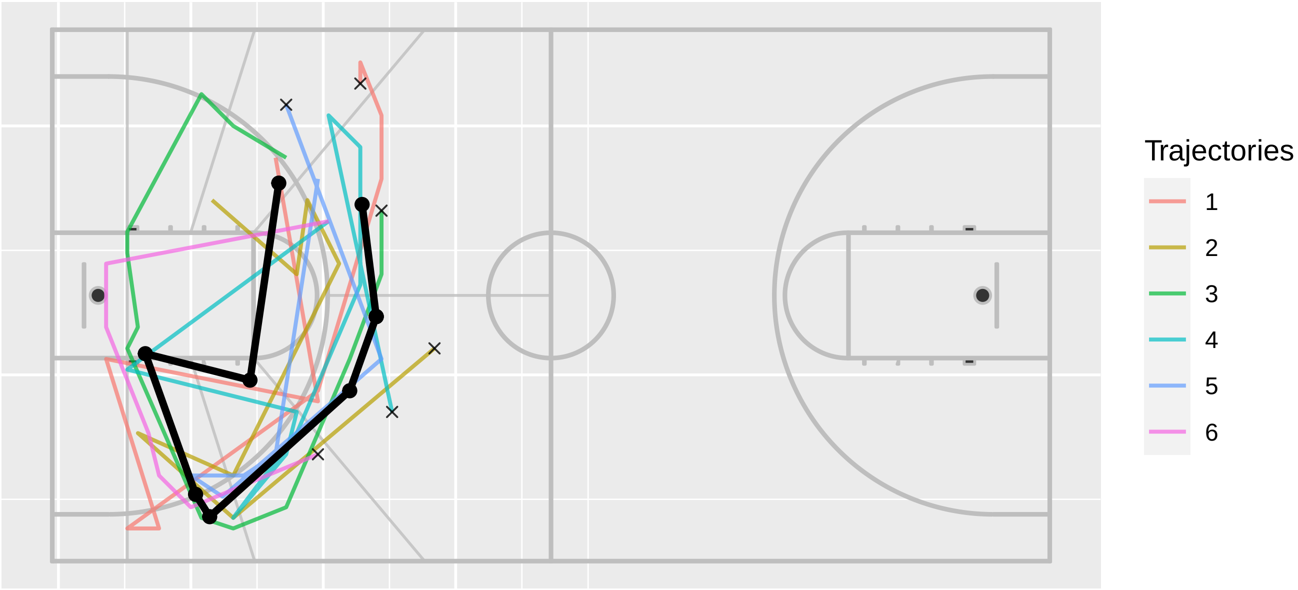

JPN Area 1 Cluster 7 : SelectTrajectories

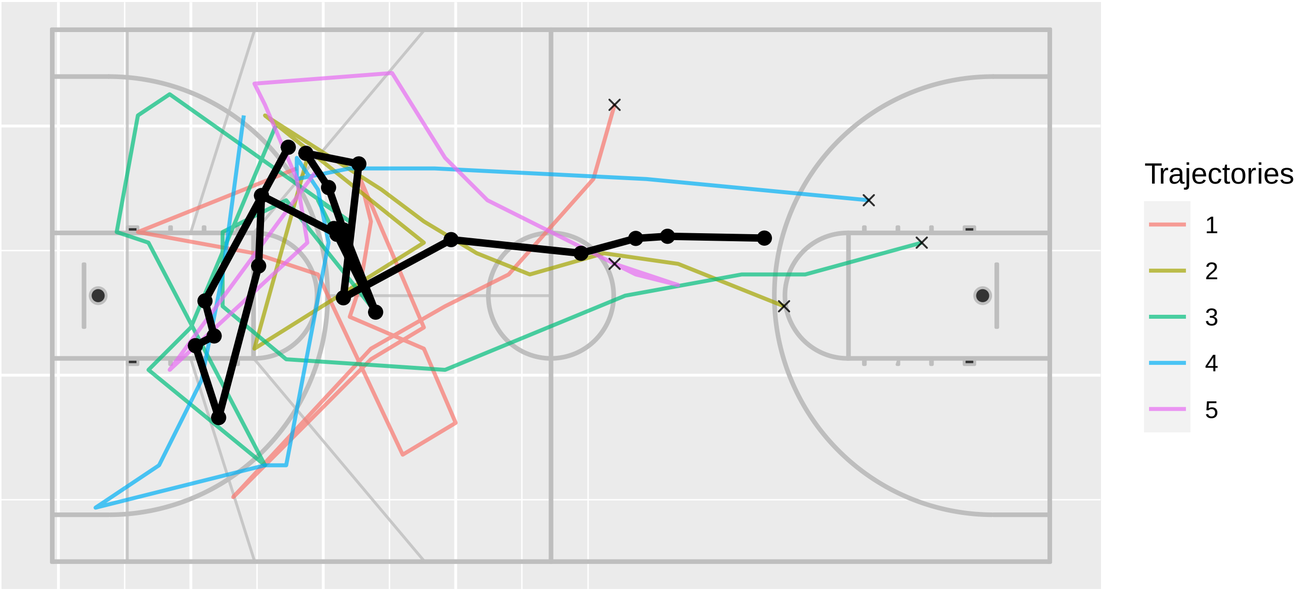

JPN Area 1 Cluster 8 : SelectTrajectories

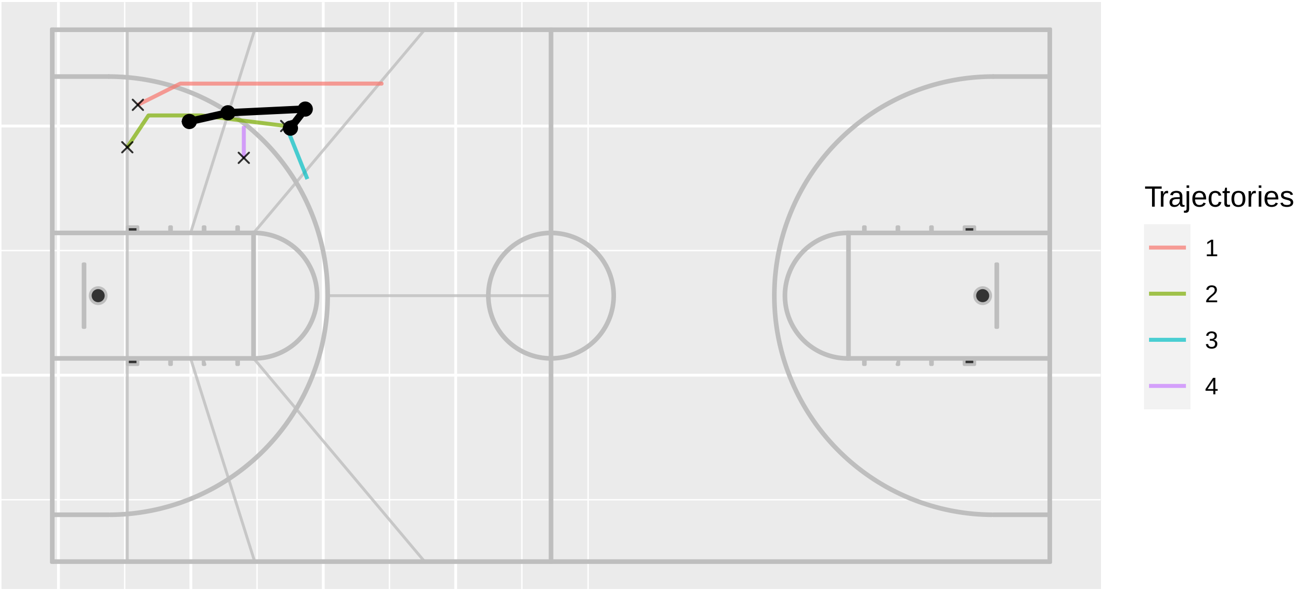

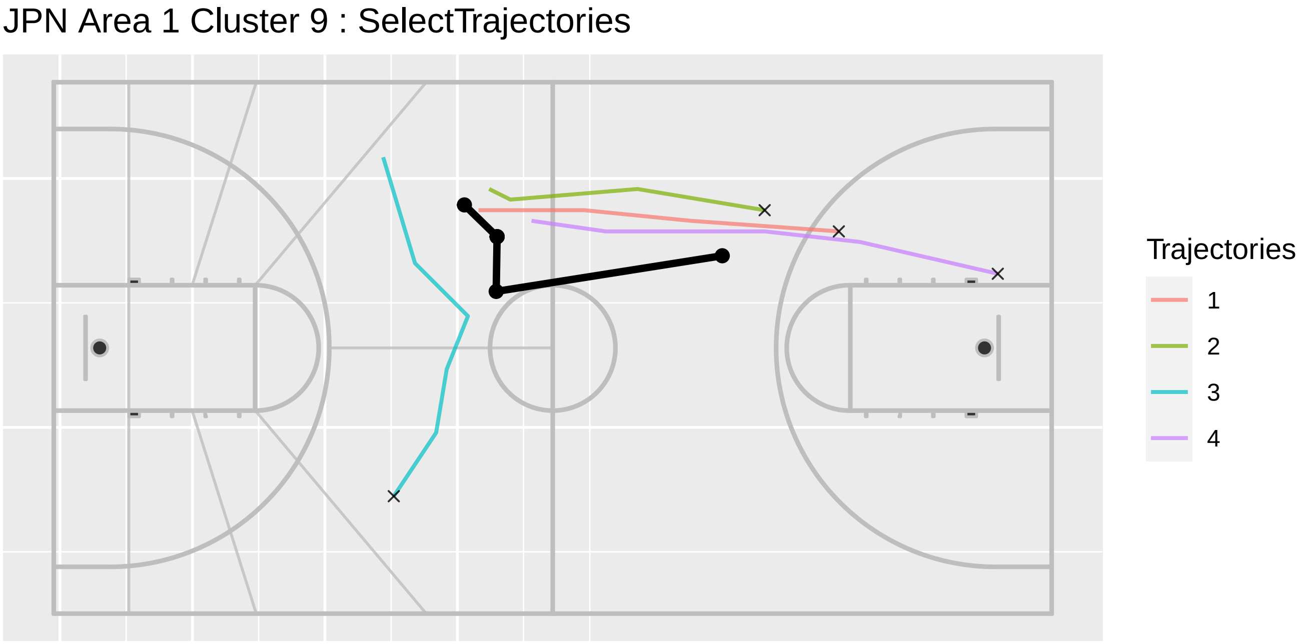
JPN Area 1 Cluster 9 : SelectTrajectories
Trajectories
1
2
3
4

JPN Area 1 Cluster 10 : SelectTrajectories

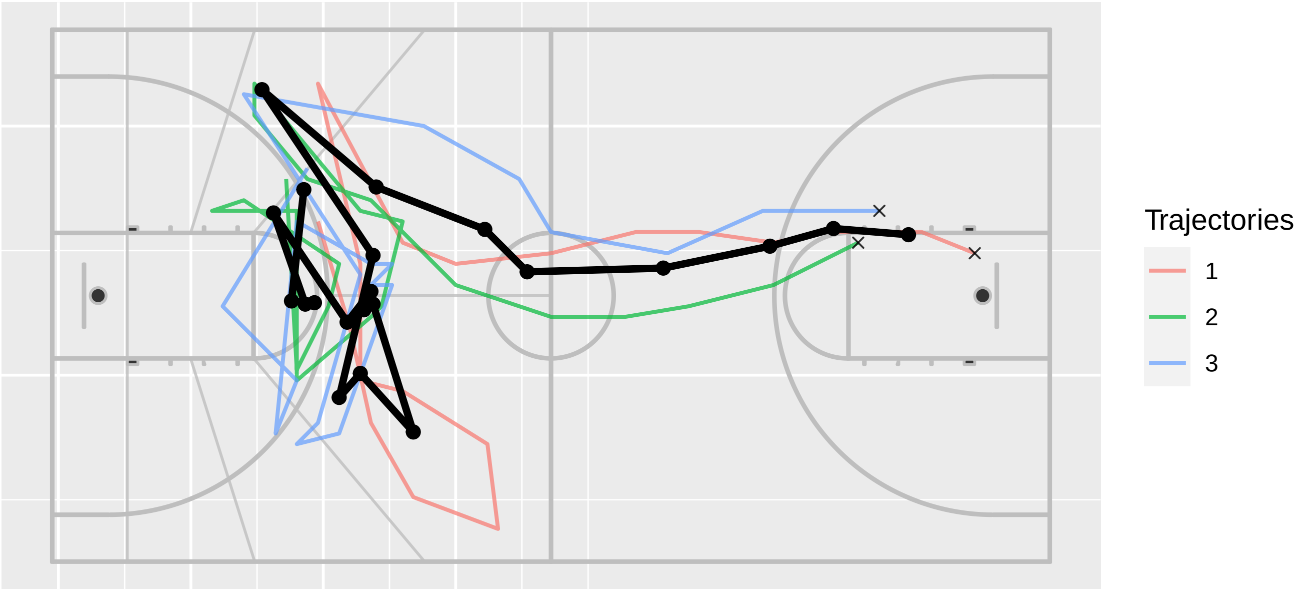

JPN Area 1 Cluster 11 : SelectTrajectories

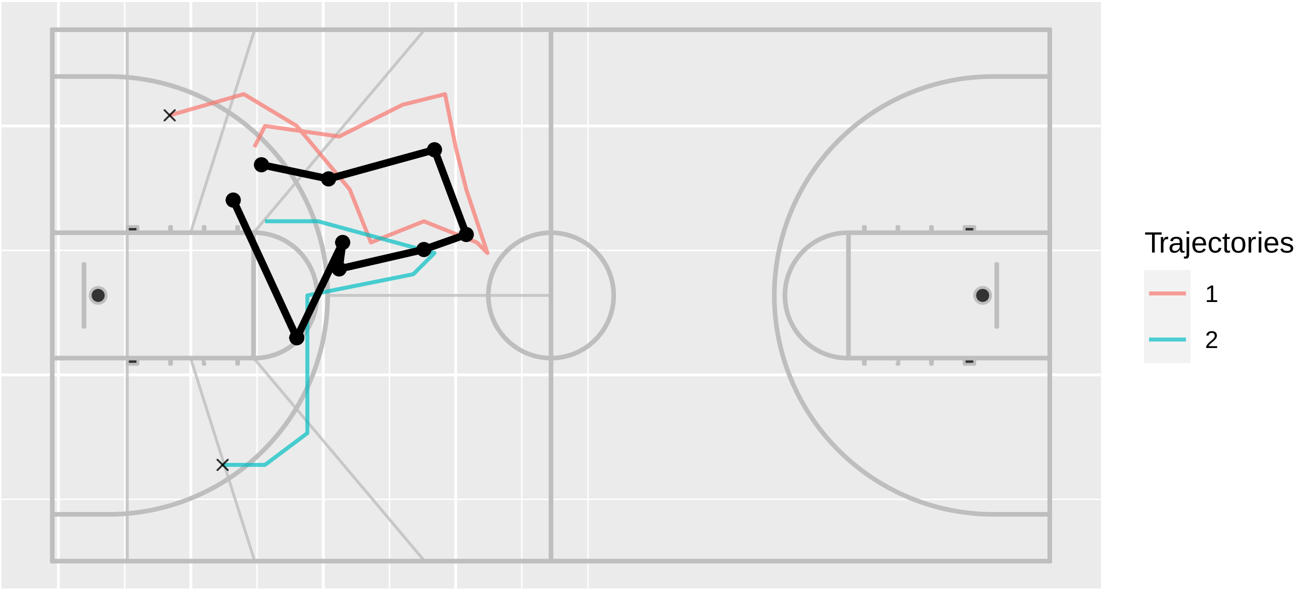

JPN Area 1 Cluster 12 : SelectTrajectories

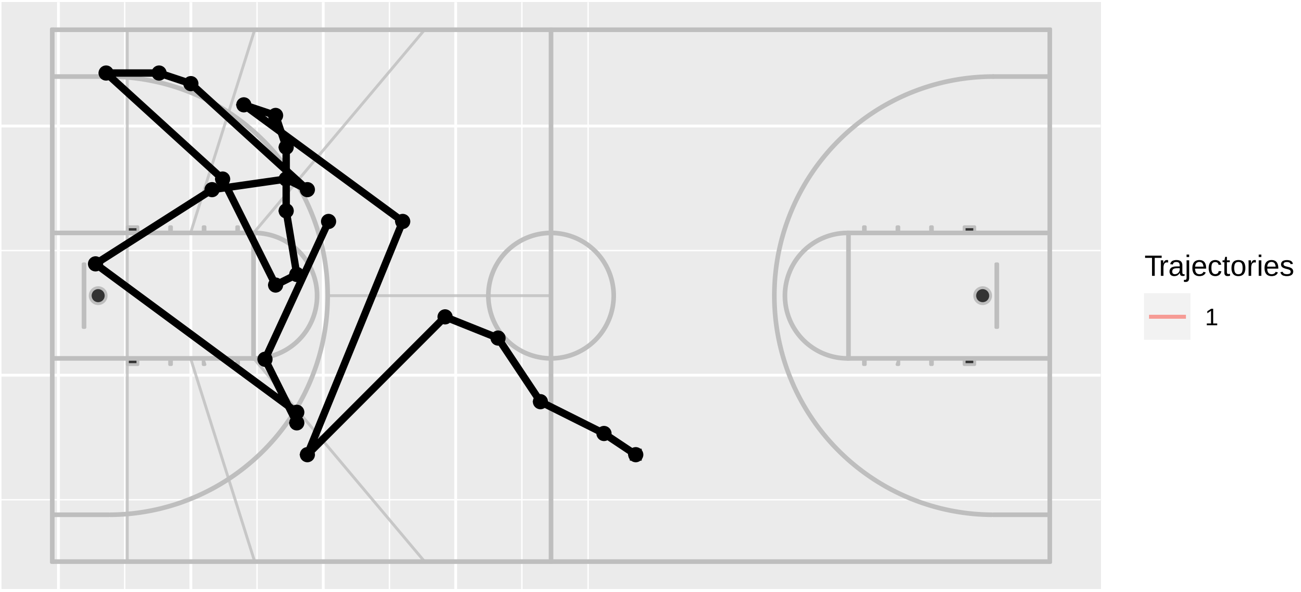

JPN Area 2 Cluster 1 : SelectTrajectories

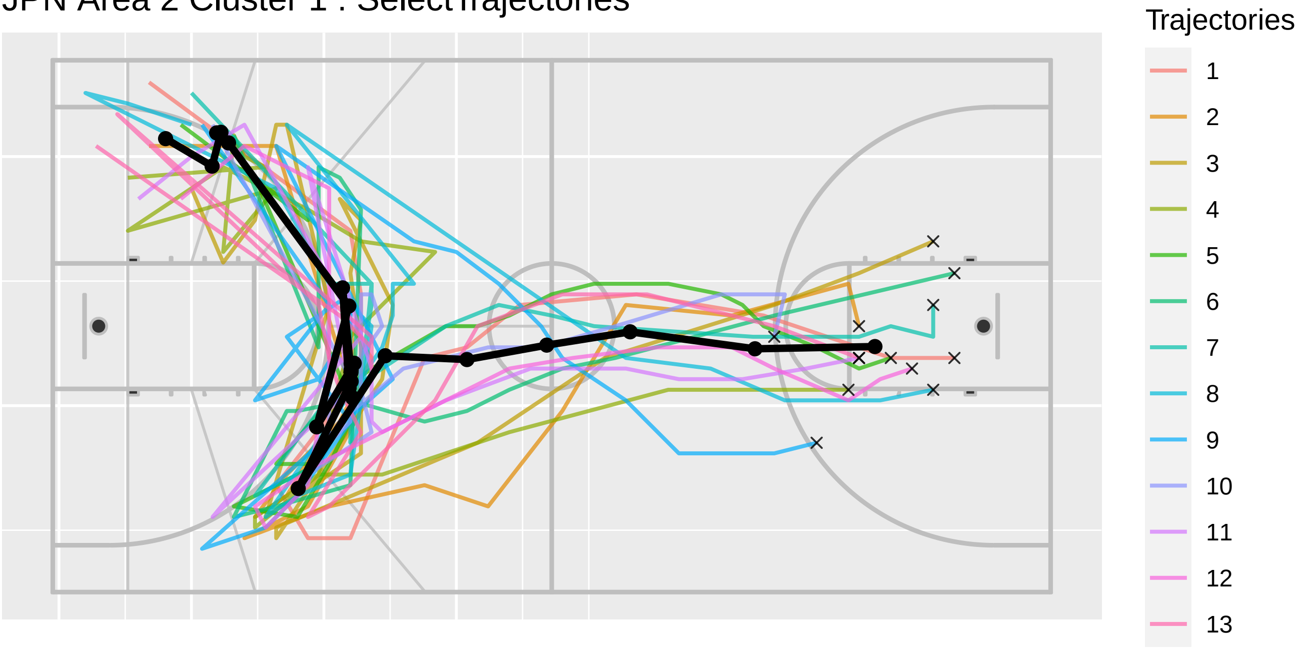

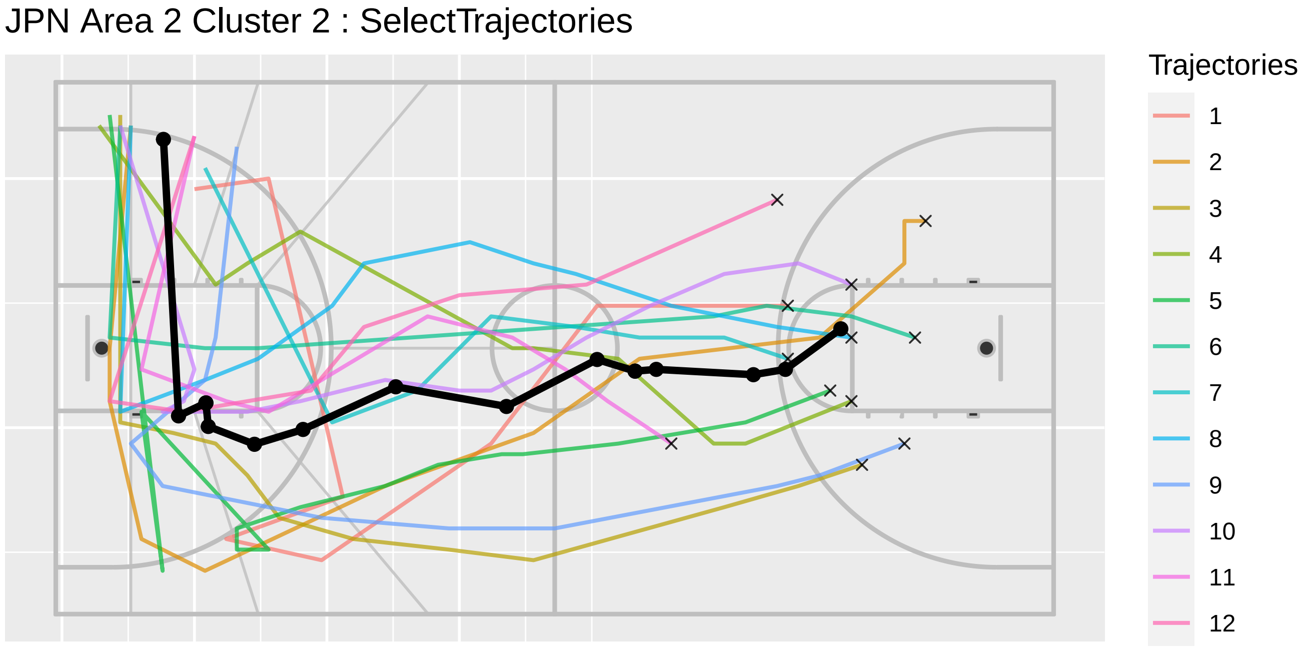
JPN Area 2 Cluster 2 : SelectTrajectories
Trajectories
1
2
3
4
5
6
7
8
9
10
11
12

JPN Area 2 Cluster 3 : SelectTrajectories

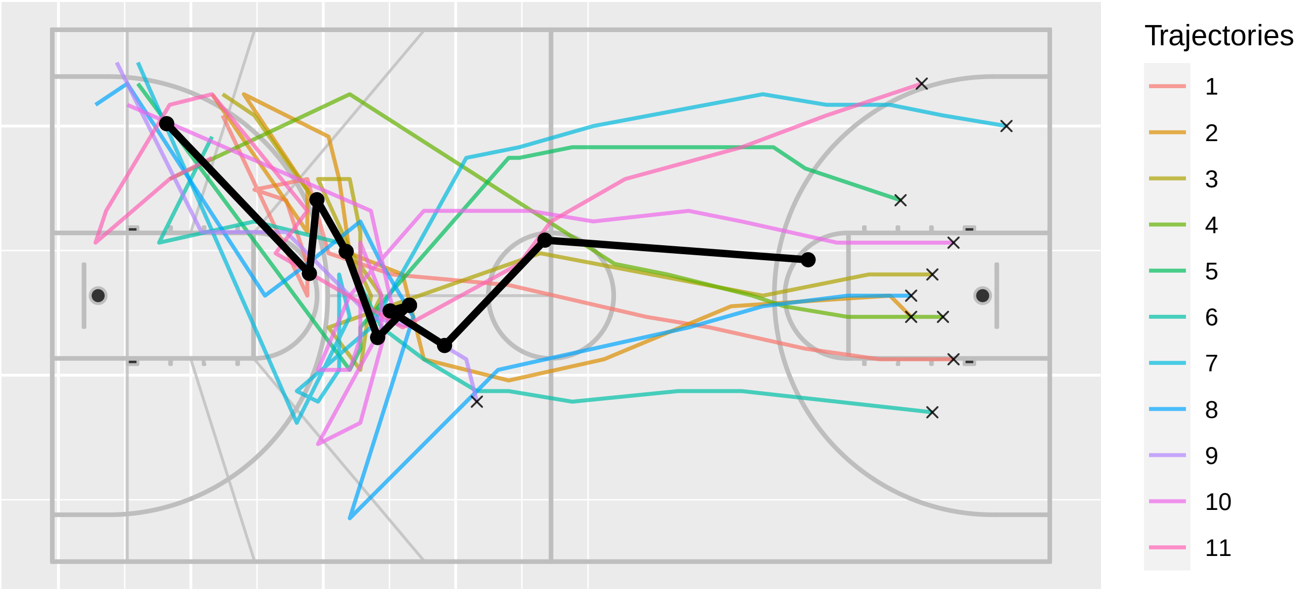

JPN Area 2 Cluster 4 : SelectTrajectories

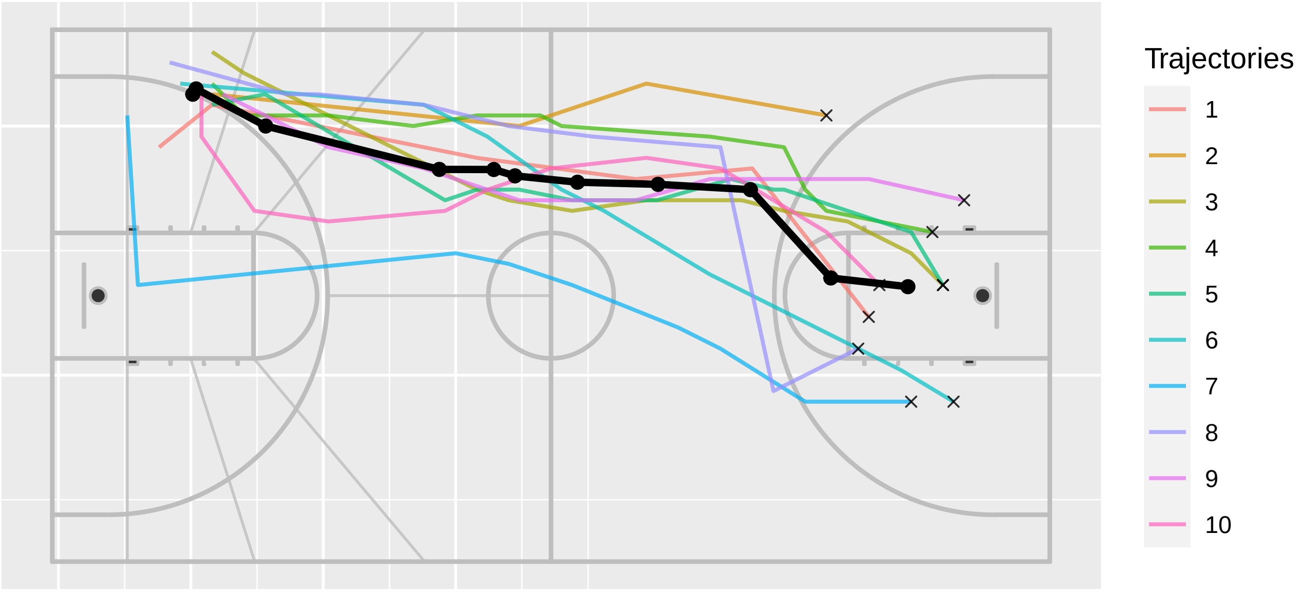

JPN Area 2 Cluster 5 : SelectTrajectories

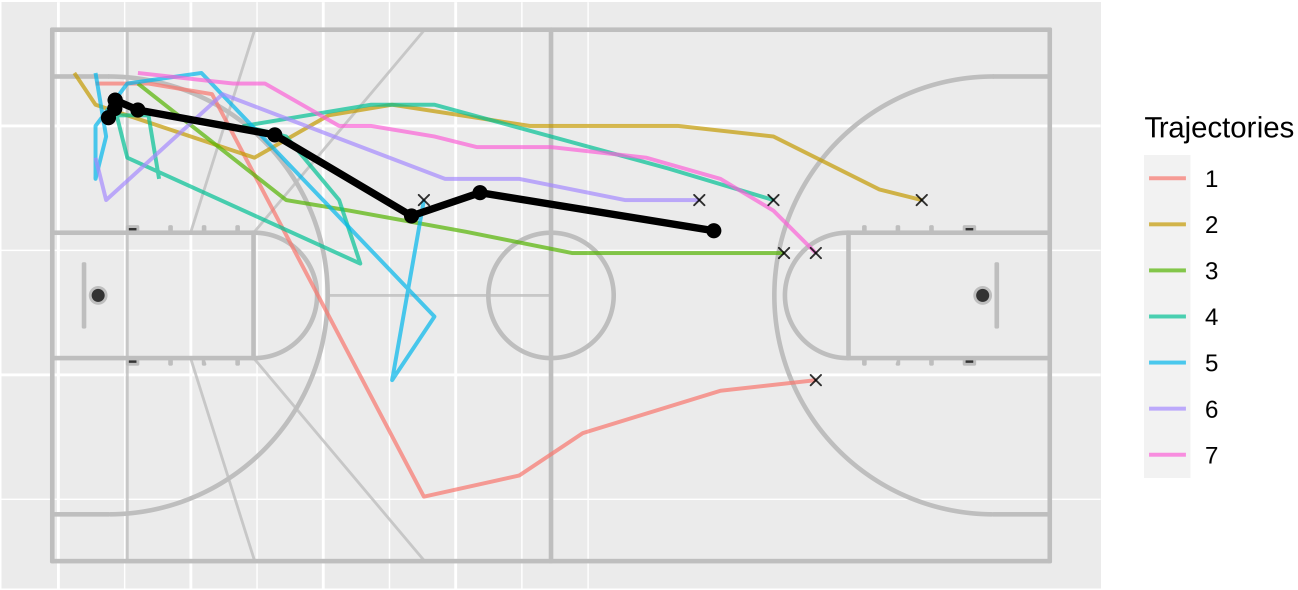

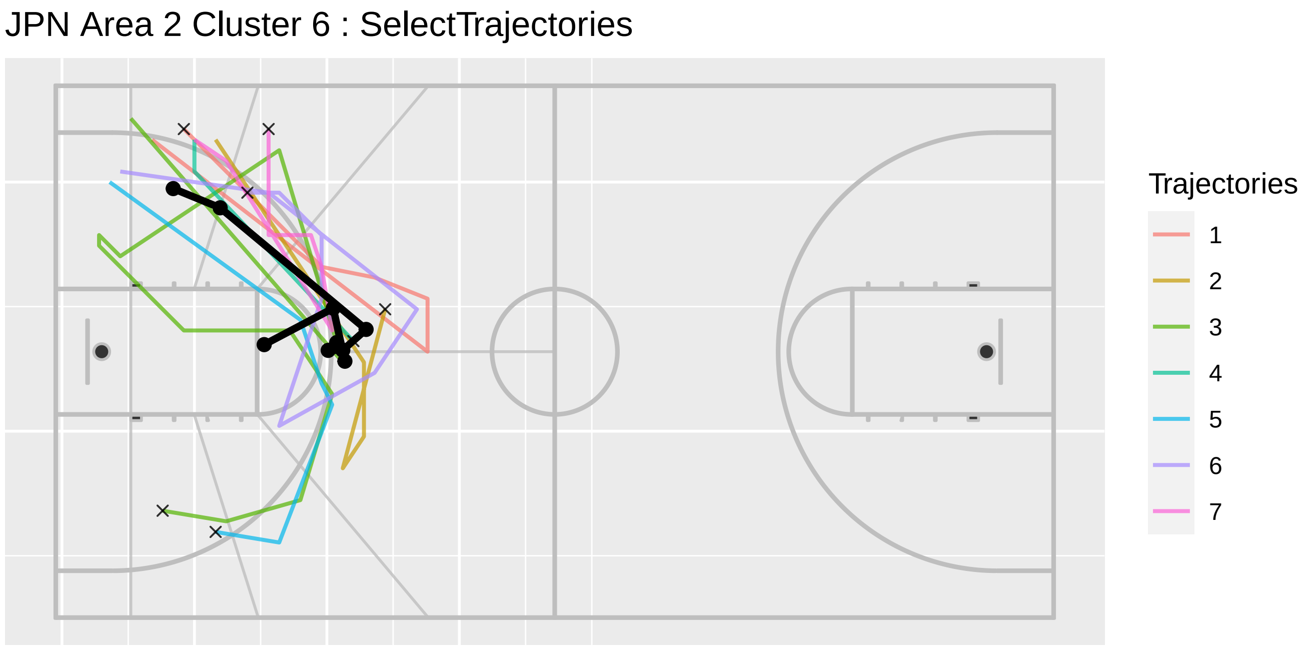
JPN Area 2 Cluster 6 : SelectTrajectories
Trajectories
1
2
3
4
5
6
7

JPN Area 2 Cluster 7 : SelectTrajectories

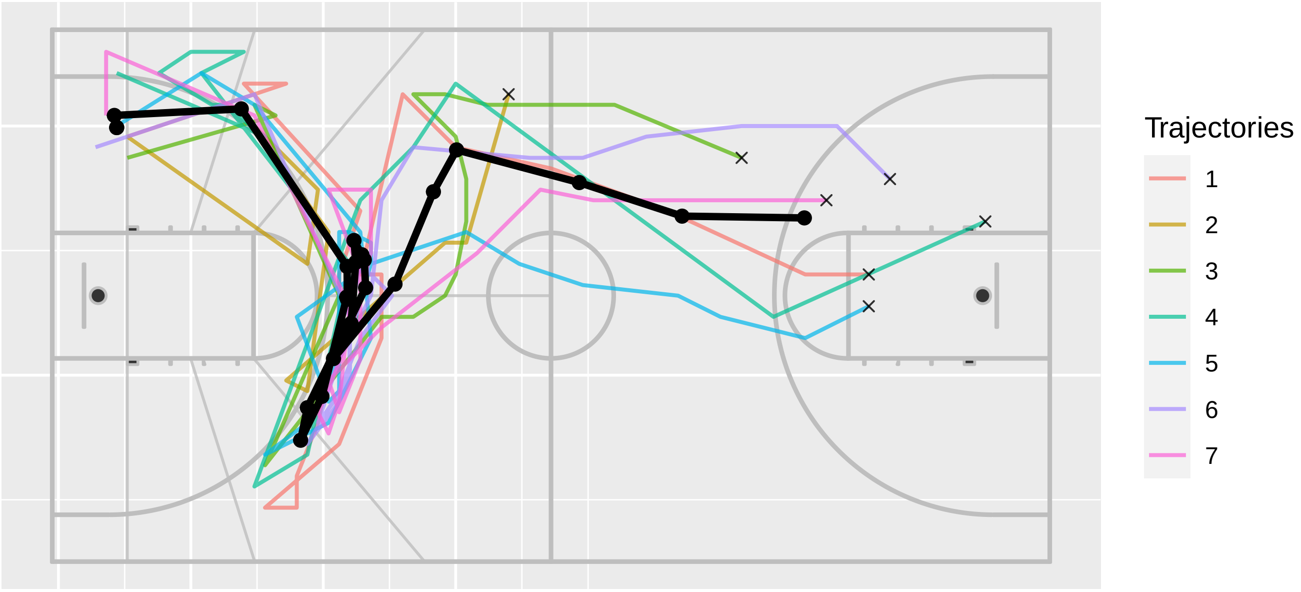

JPN Area 2 Cluster 8 : SelectTrajectories

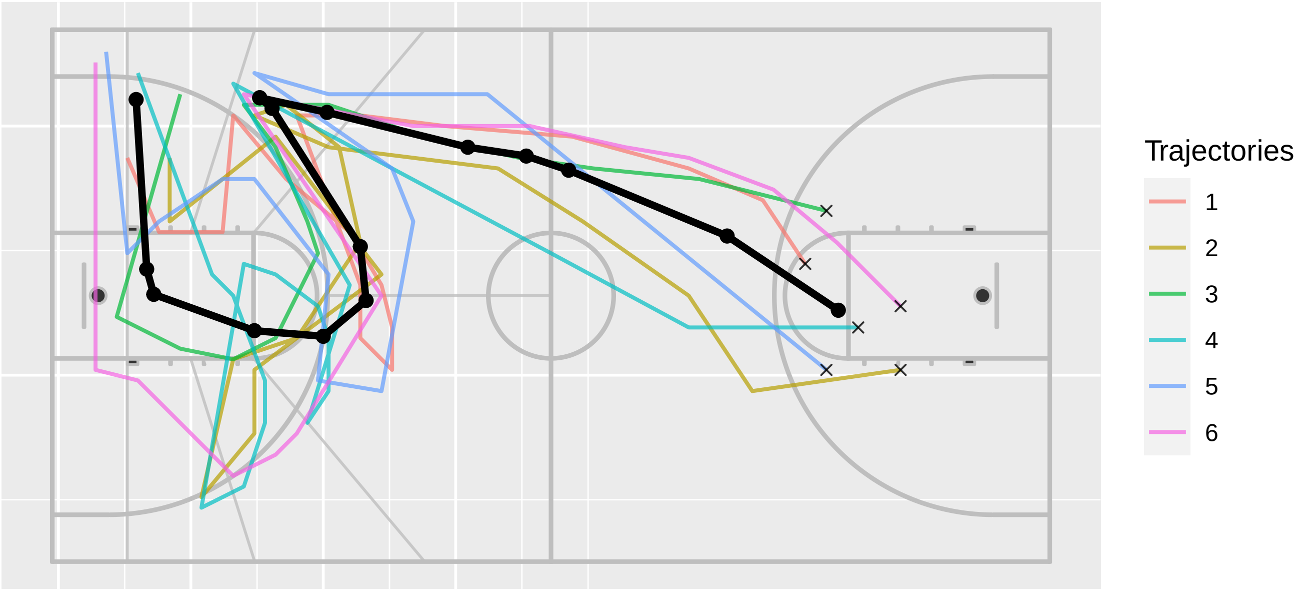

JPN Area 2 Cluster 9 : SelectTrajectories

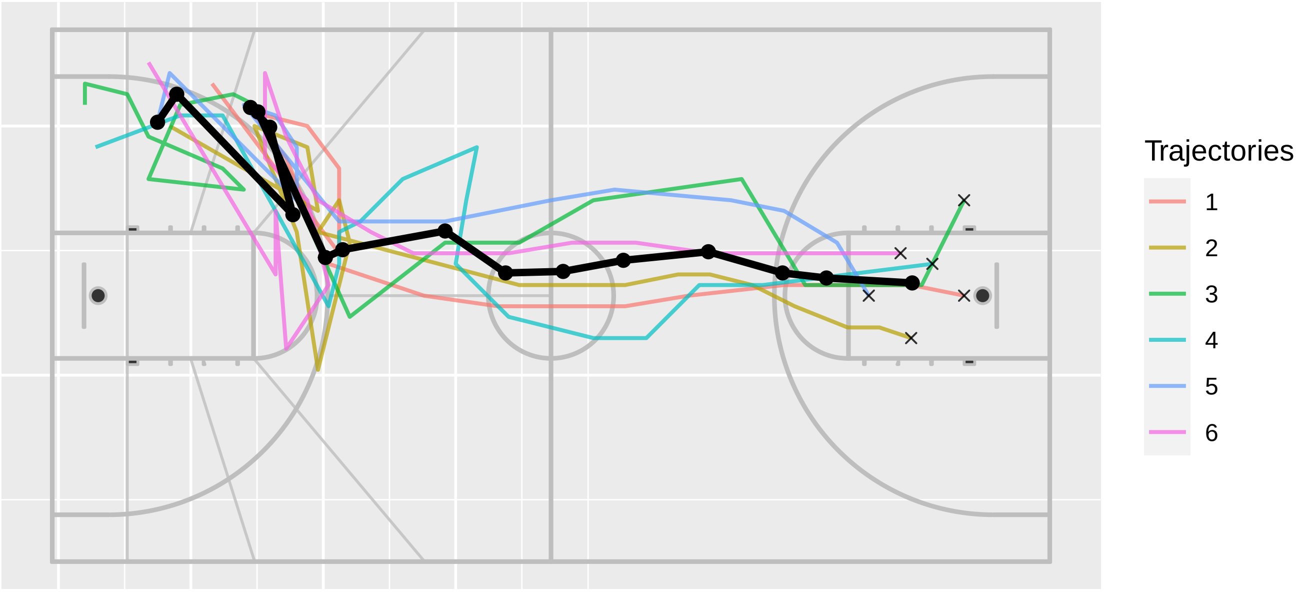

JPN Area 2 Cluster 10 : SelectTrajectories

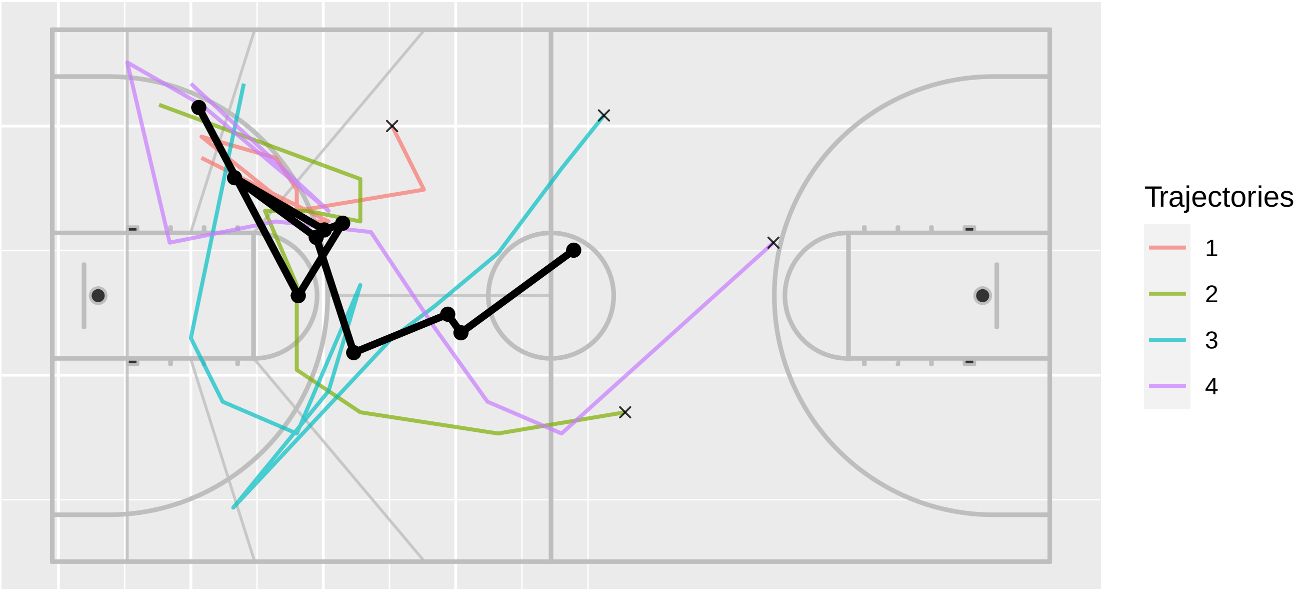

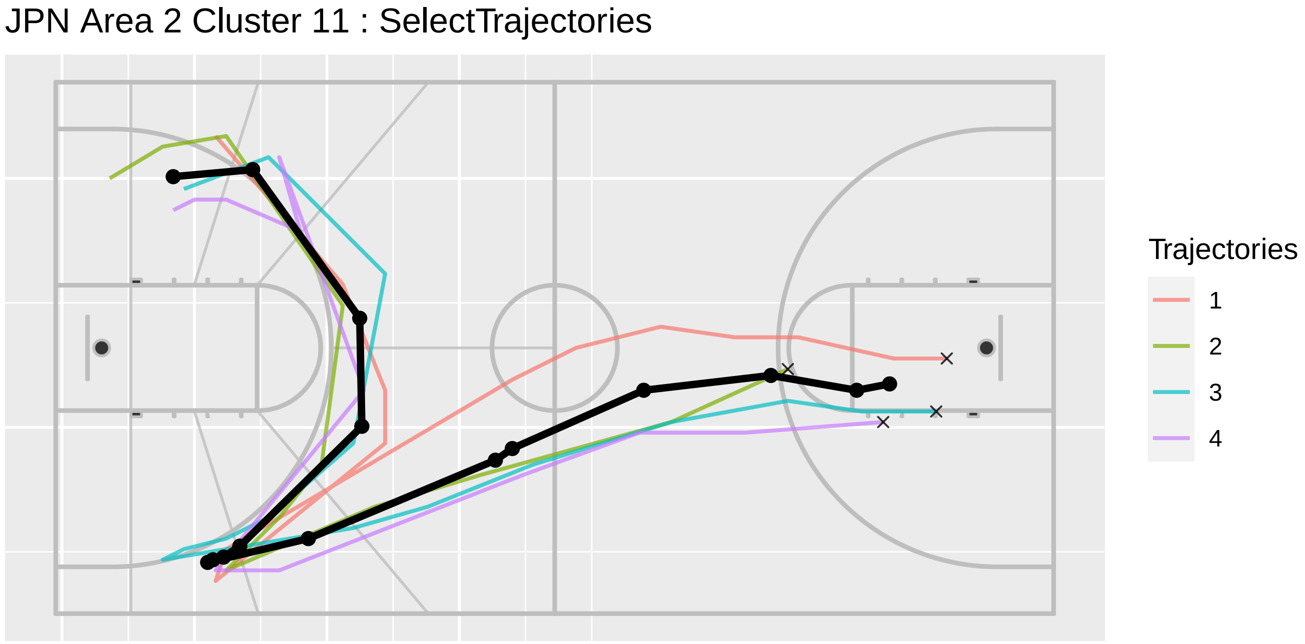
JPN Area 2 Cluster 11 : SelectTrajectories
Trajectories
1
2
3
4

JPN Area 2 Cluster 12 : SelectTrajectories

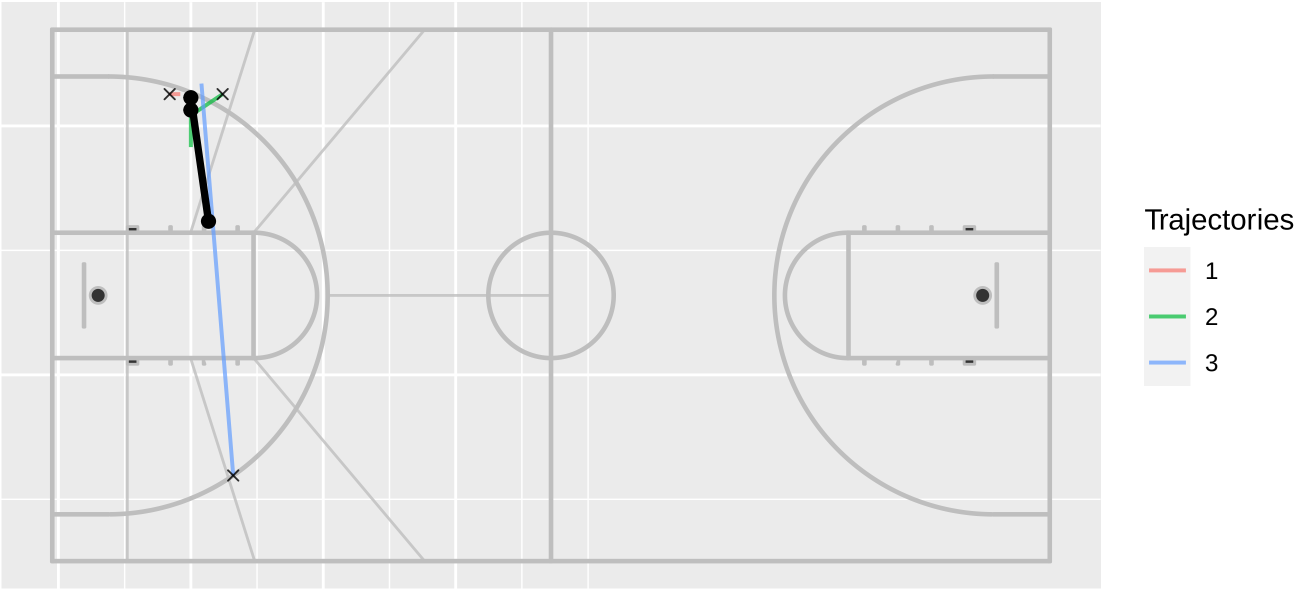

JPN Area 3 Cluster 1 : SelectTrajectories

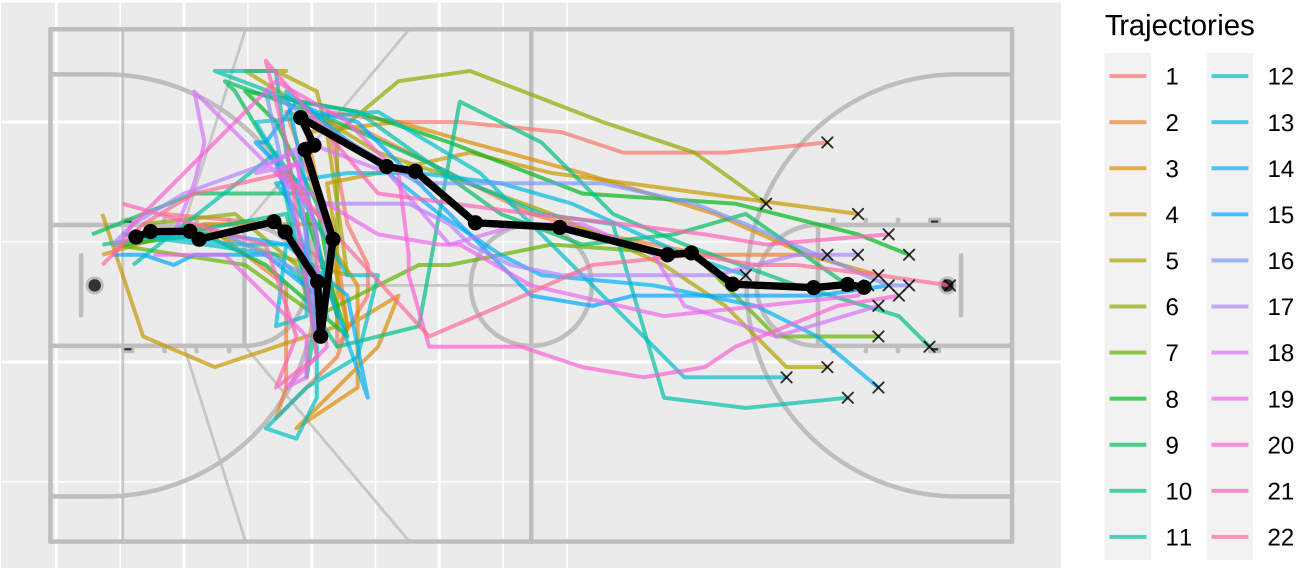

JPN Area 3 Cluster 2 : SelectTrajectories

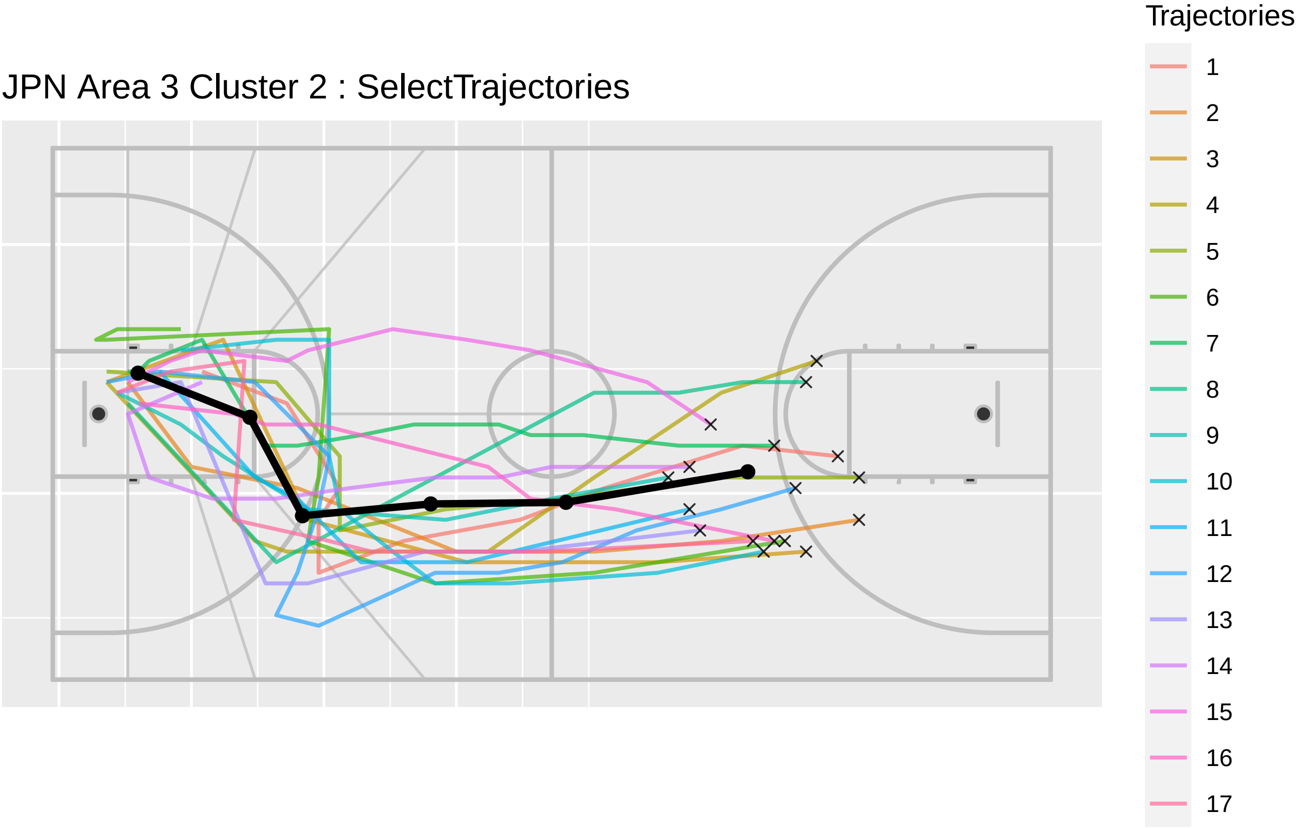

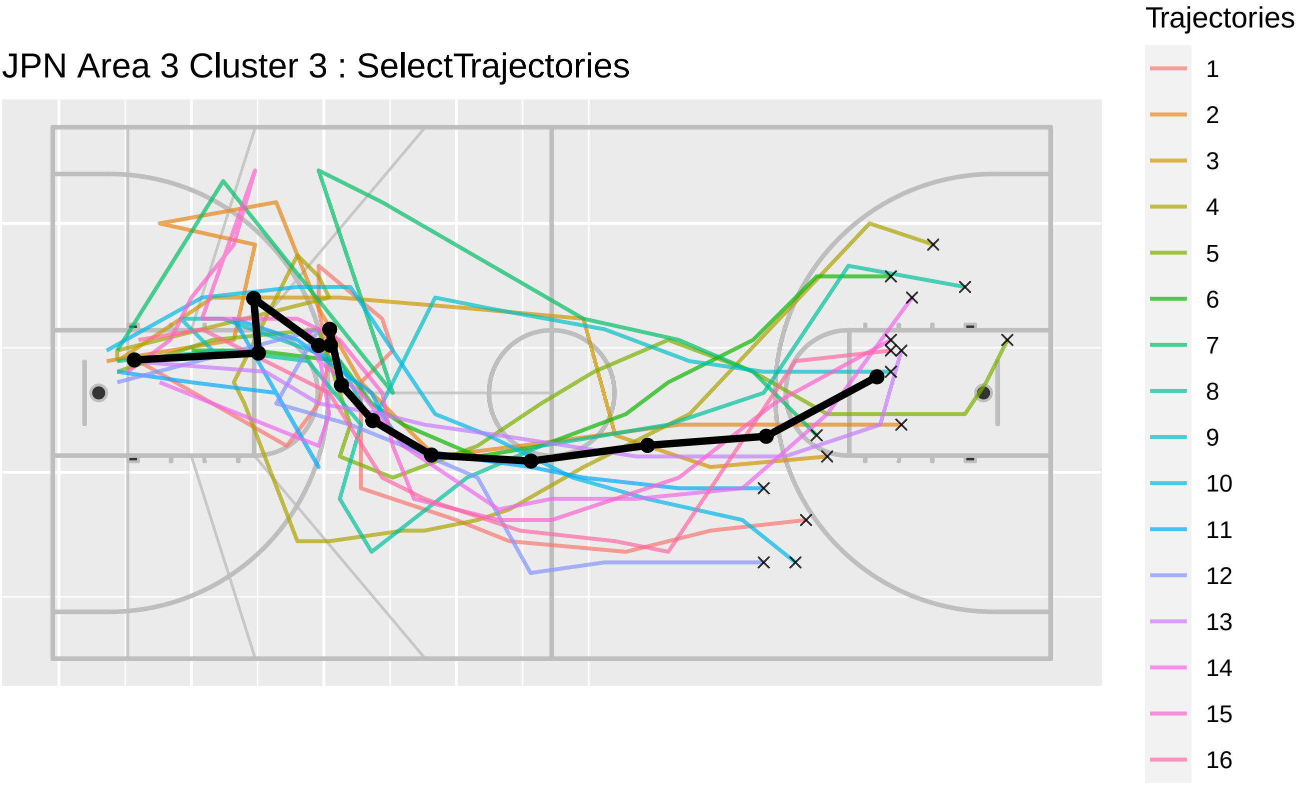
JPN Area 3 Cluster 3 : SelectTrajectories
Trajectories
1
2
3
4
5
6
7
8
9
10
11
12
13
14
15
16

JPN Area 3 Cluster 4 : SelectTrajectories

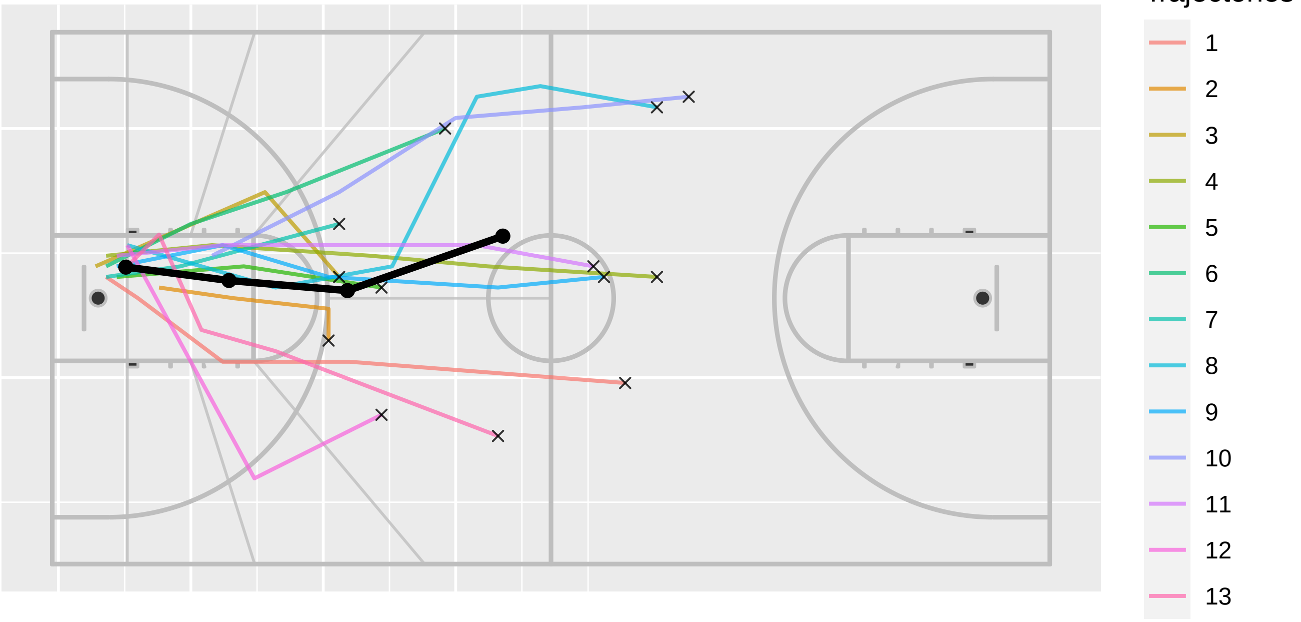

JPN Area 3 Cluster 5 : SelectTrajectories

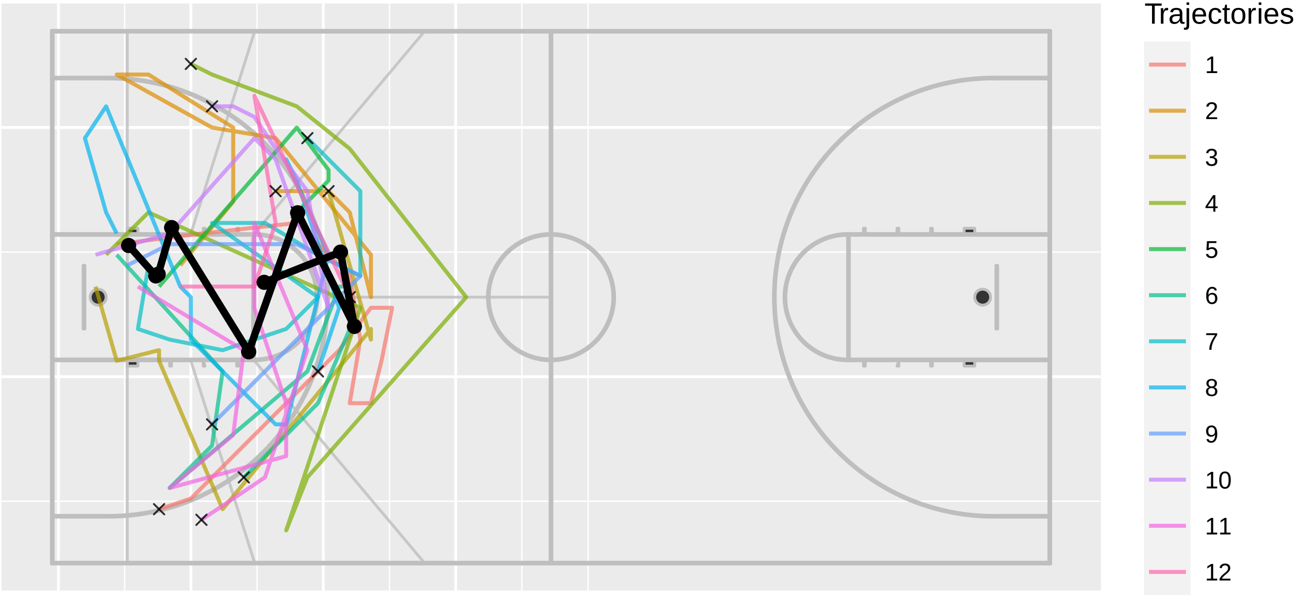

JPN Area 3 Cluster 6 : SelectTrajectories

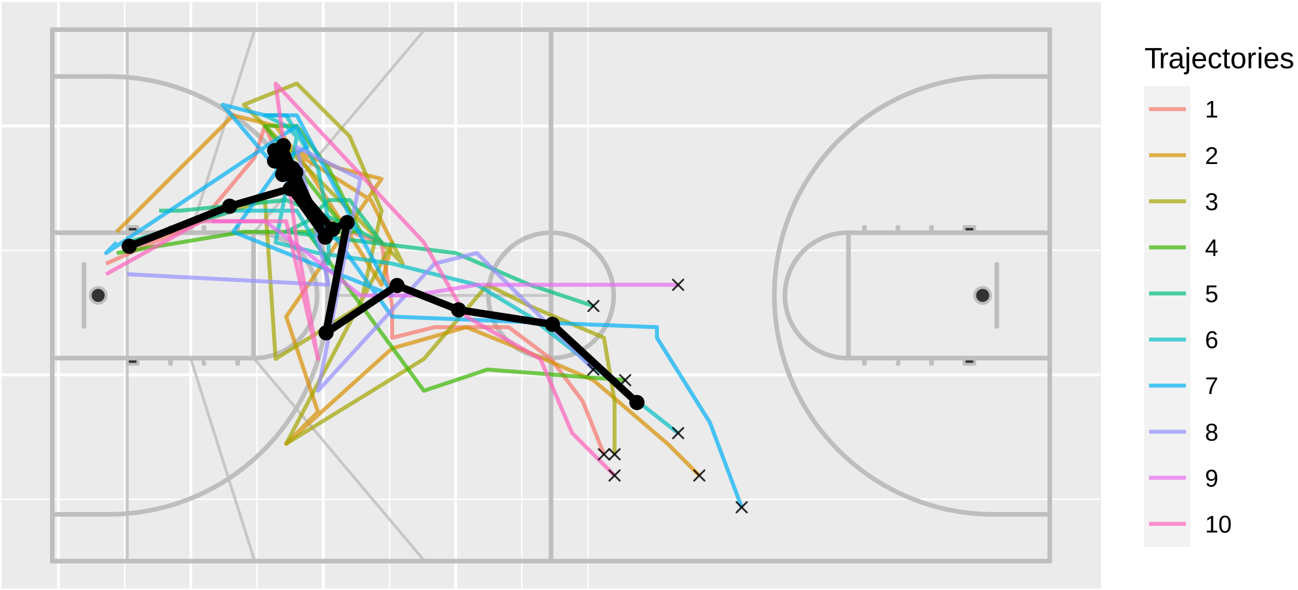

JPN Area 3 Cluster 7 : SelectTrajectories

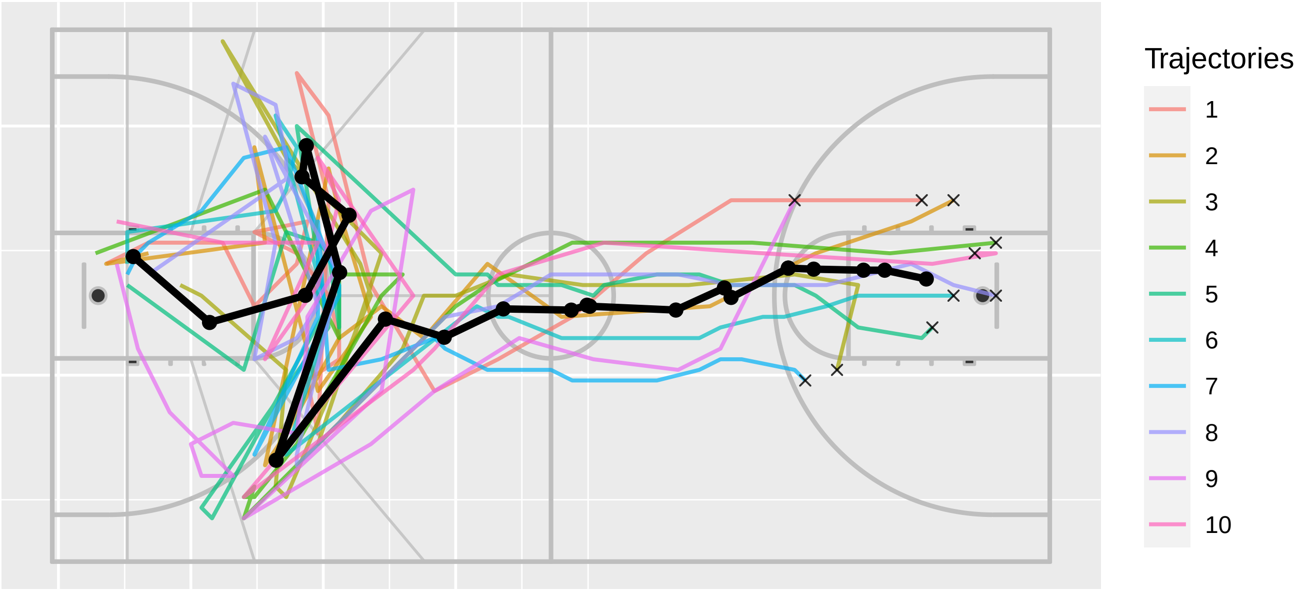

JPN Area 3 Cluster 8 : SelectTrajectories

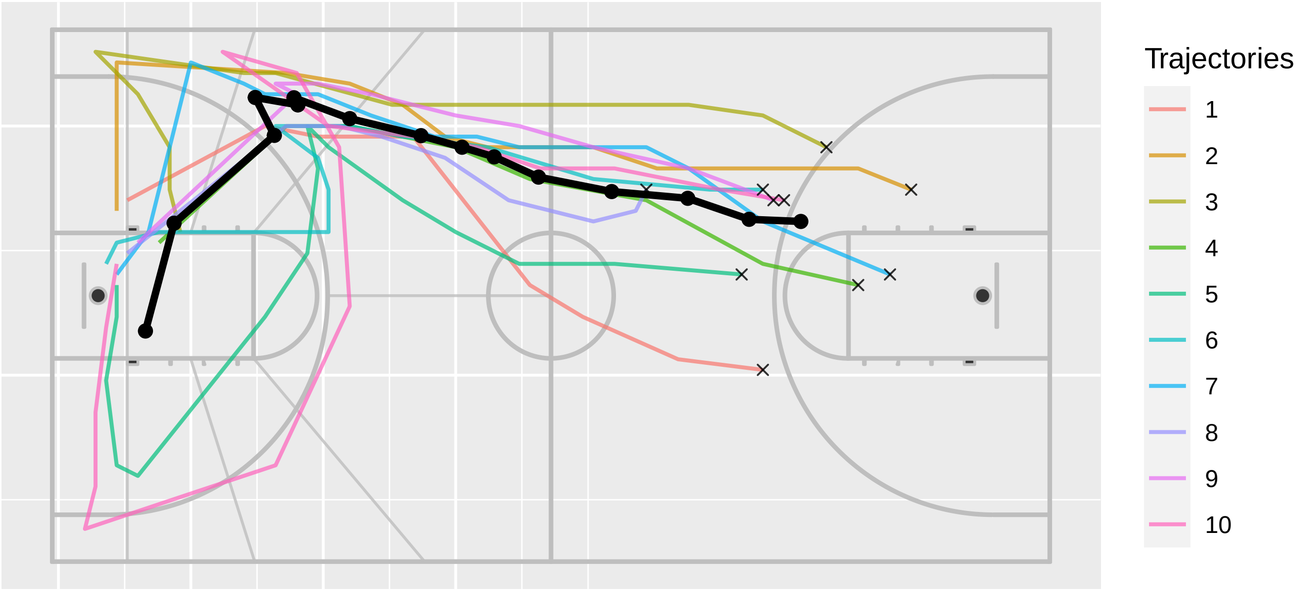

JPN Area 3 Cluster 9 : SelectTrajectories

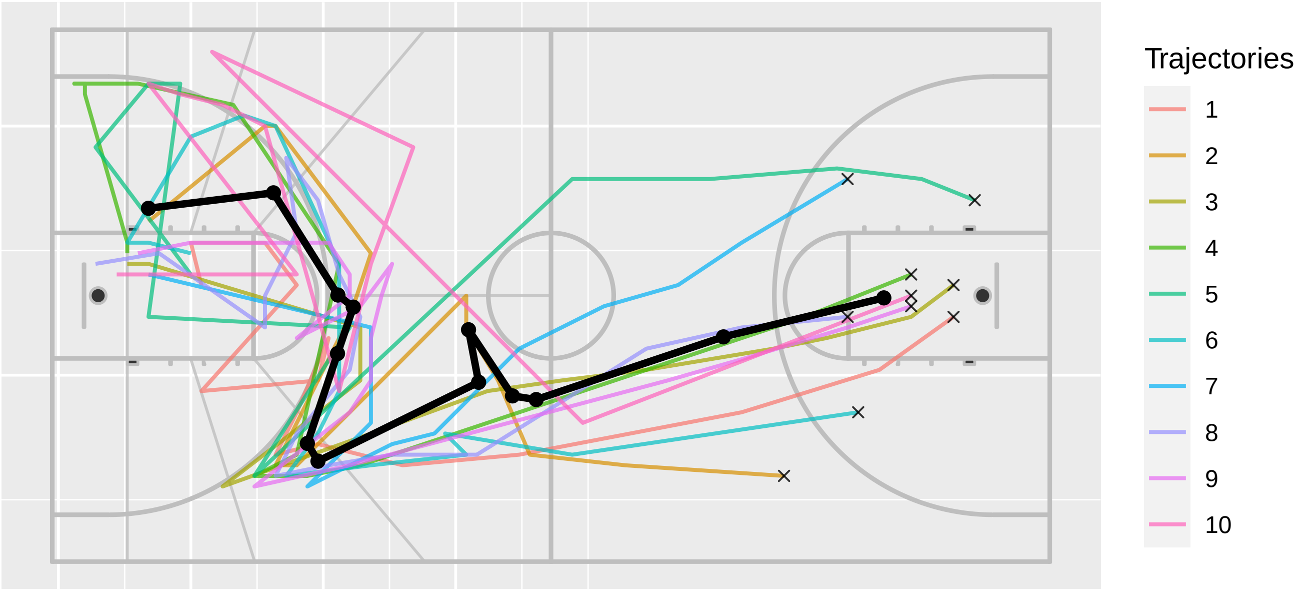

JPN Area 3 Cluster 10 : SelectTrajectories

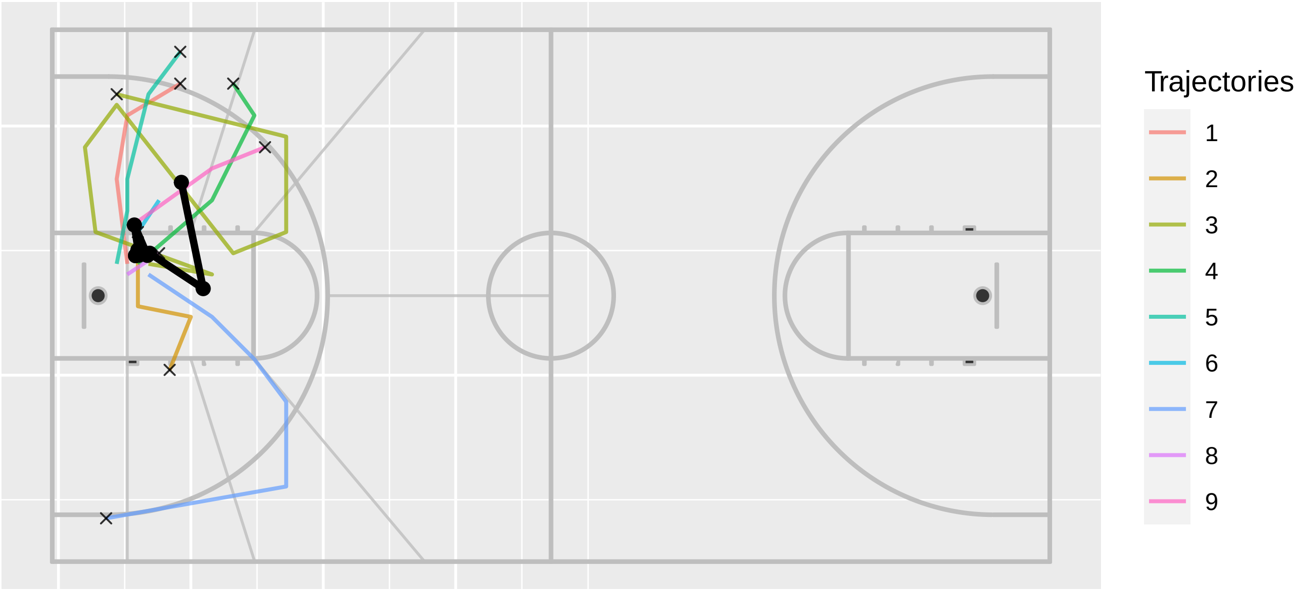

JPN Area 3 Cluster 11 : SelectTrajectories

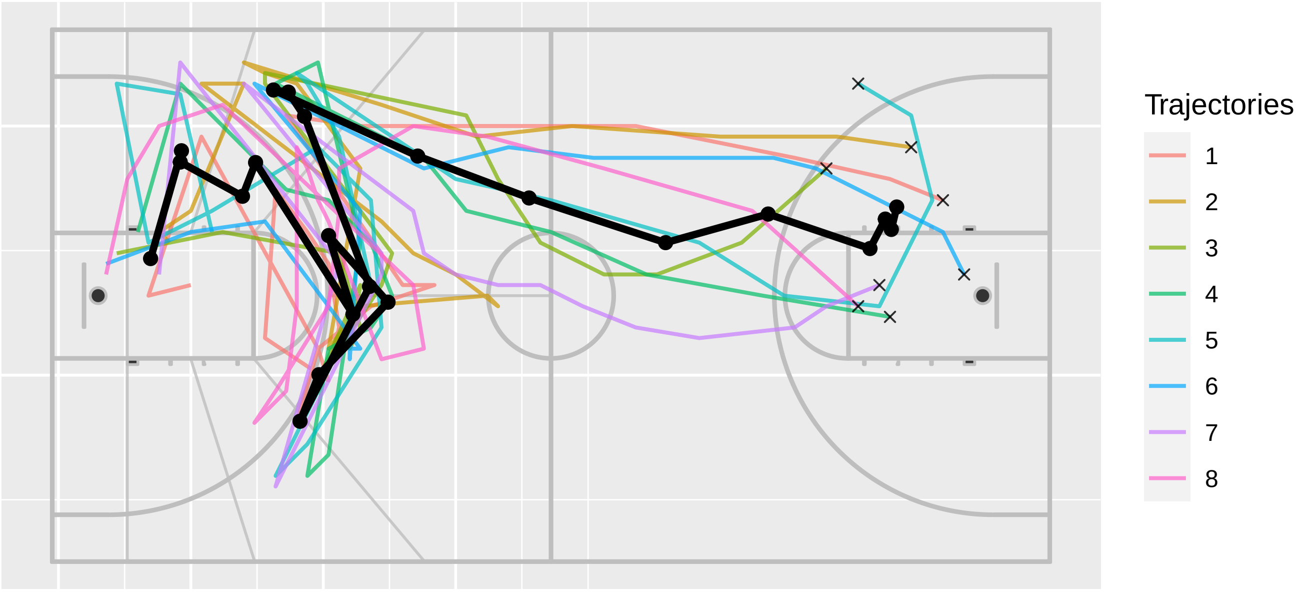

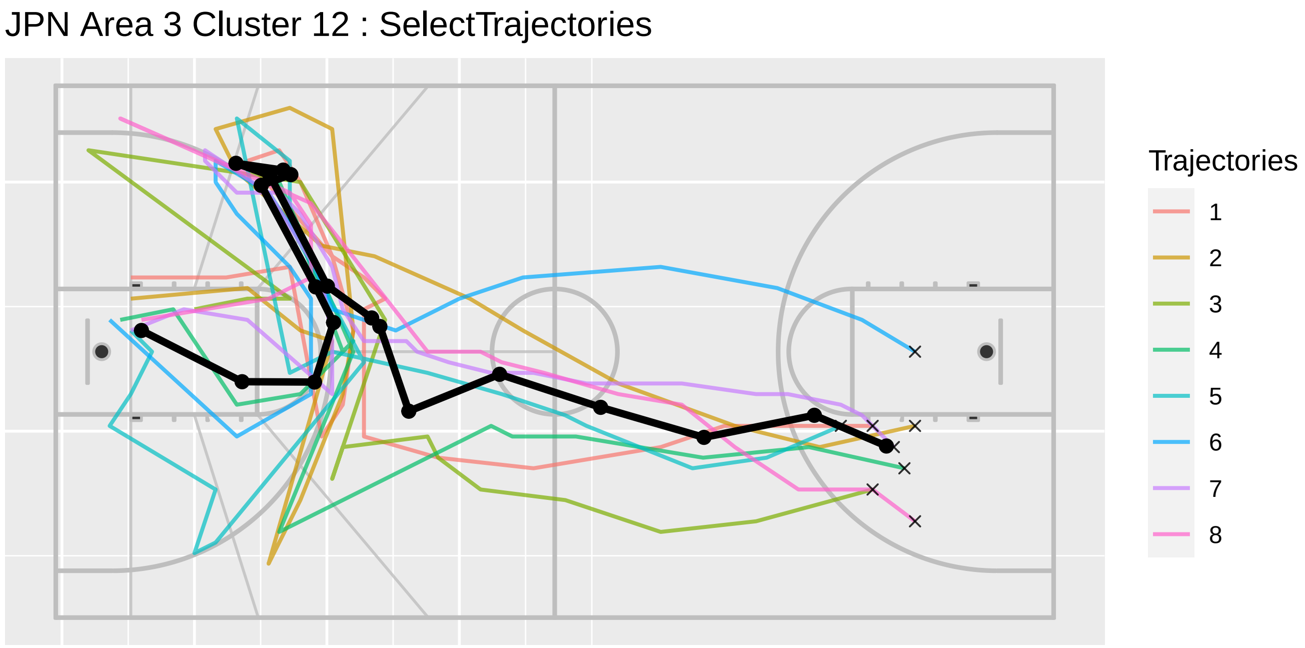
JPN Area 3 Cluster 12 : SelectTrajectories
Trajectories
1
2
3
4
5
6
7
8

JPN Area 3 Cluster 13 : SelectTrajectories

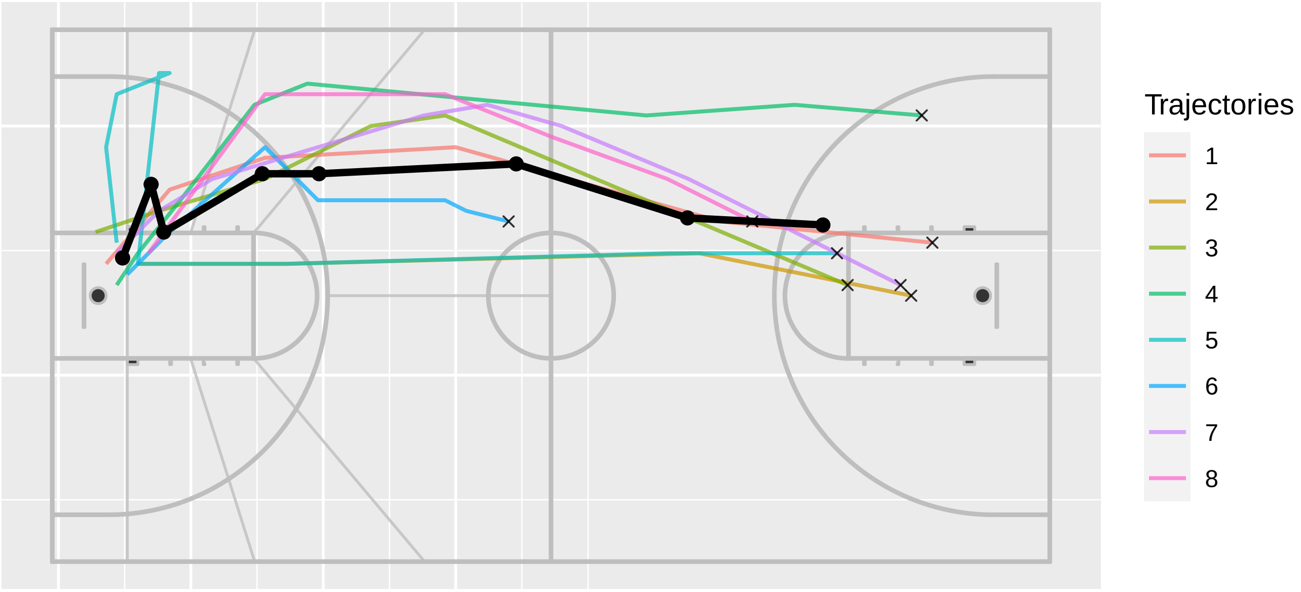

JPN Area 3 Cluster 14 : SelectTrajectories

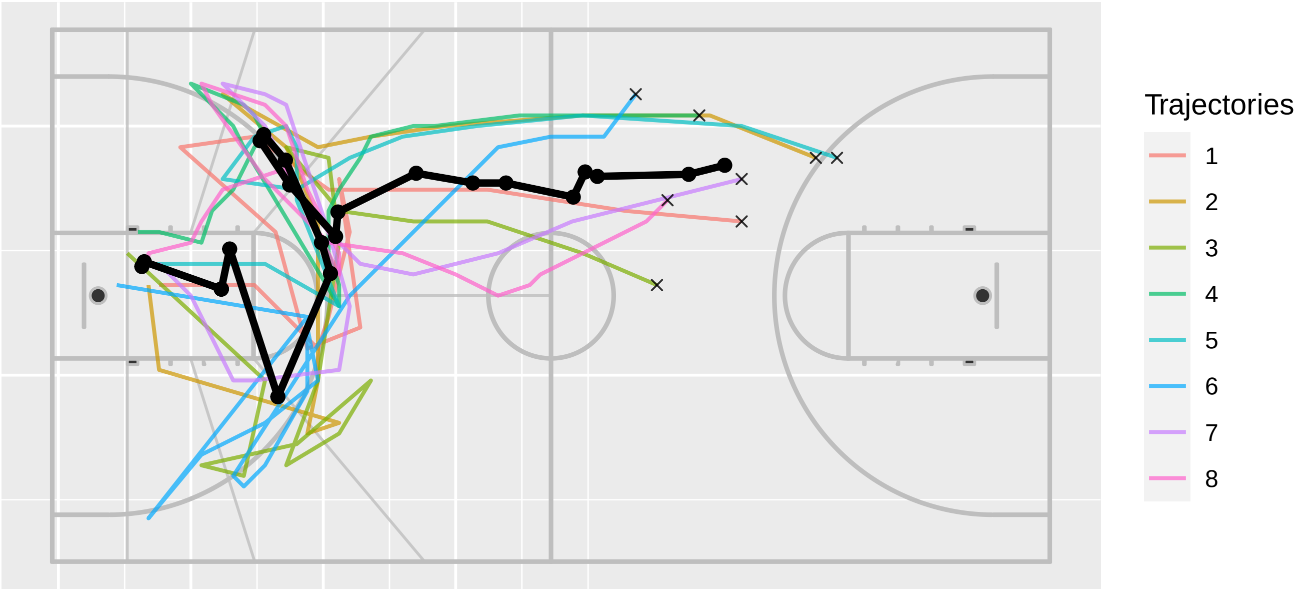

JPN Area 3 Cluster 15 : SelectTrajectories

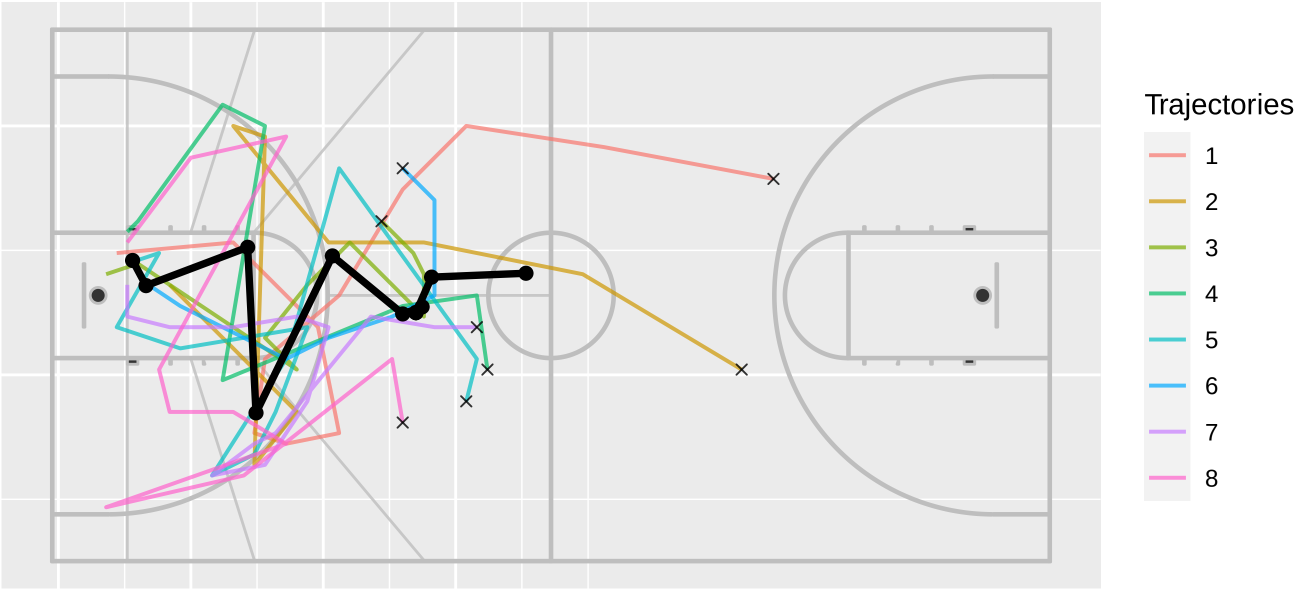

JPN Area 3 Cluster 16 : SelectTrajectories

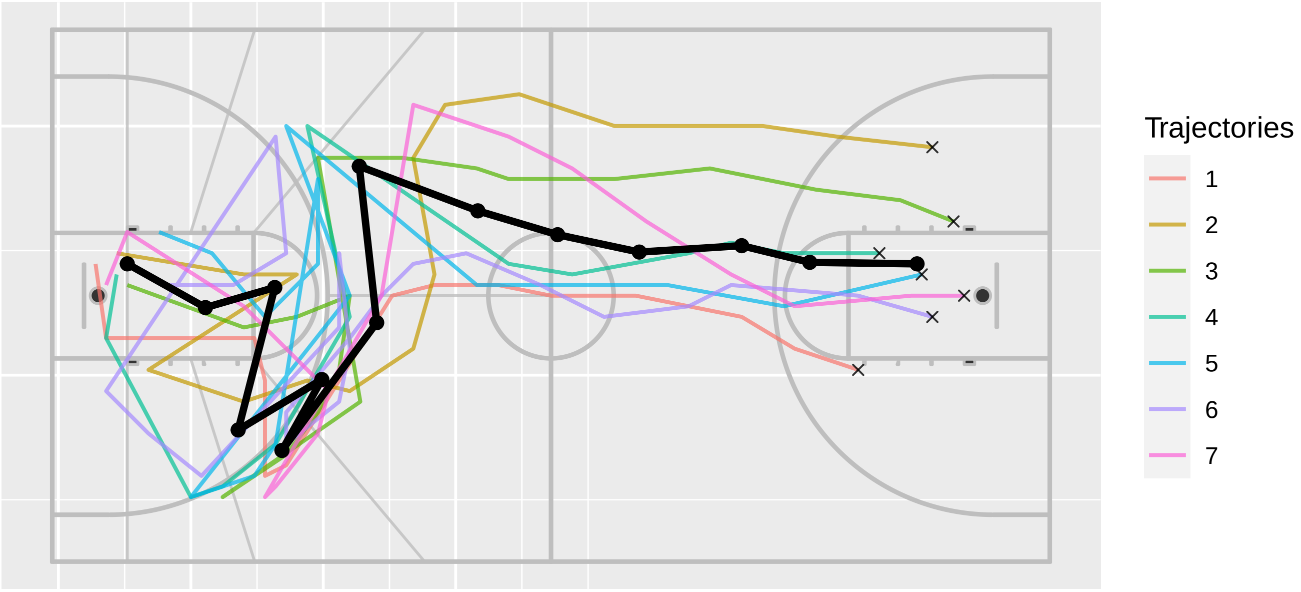

JPN Area 3 Cluster 17 : SelectTrajectories

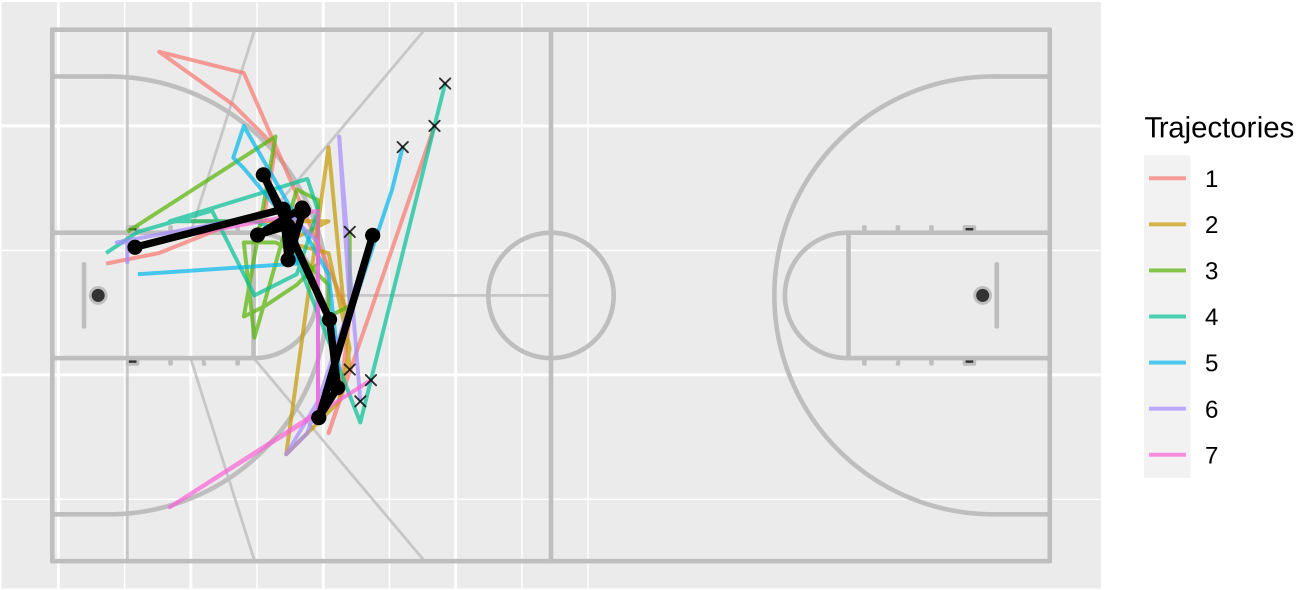

JPN Area 3 Cluster 18 : SelectTrajectories

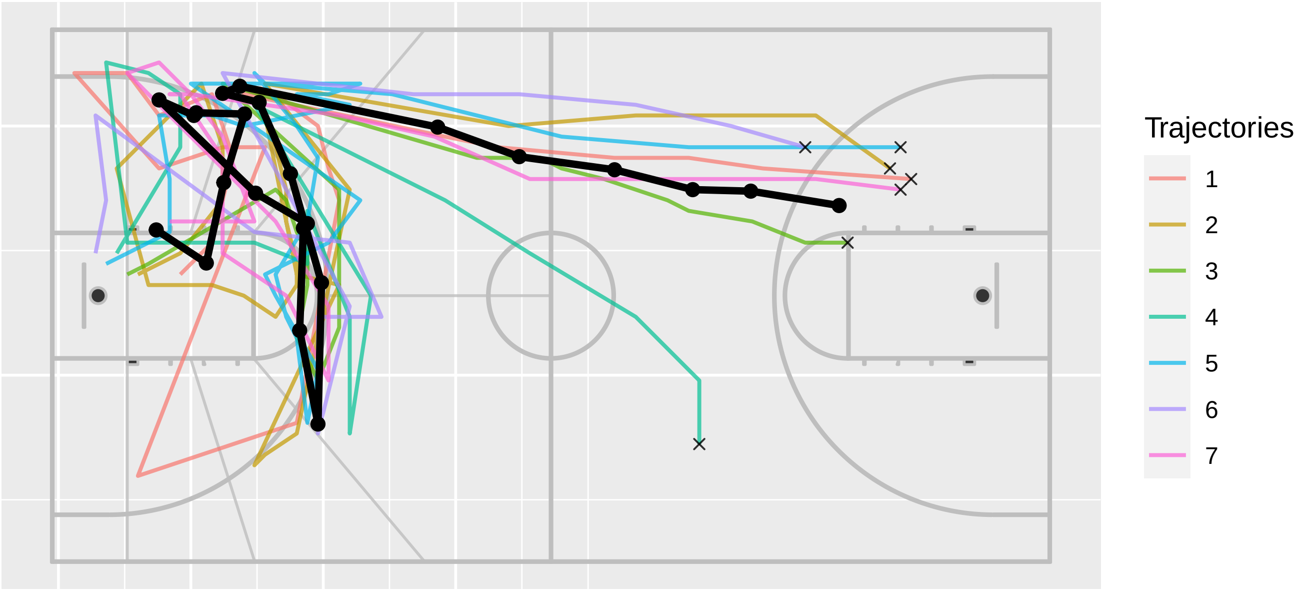

JPN Area 3 Cluster 19 : SelectTrajectories

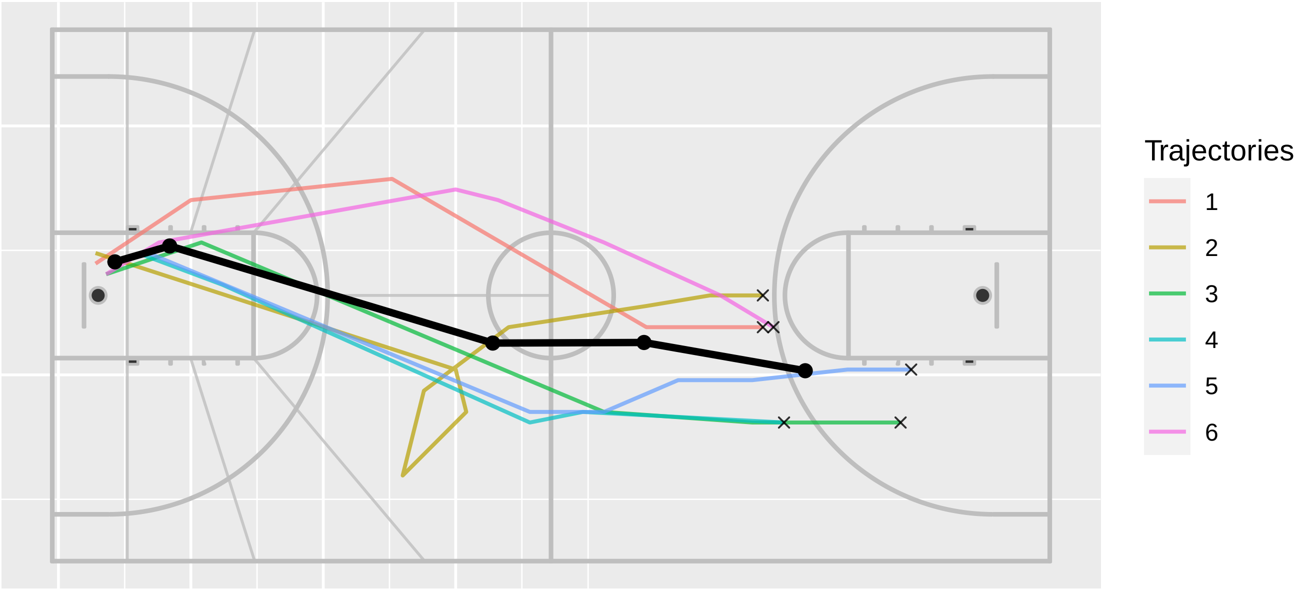

JPN Area 3 Cluster 20 : SelectTrajectories

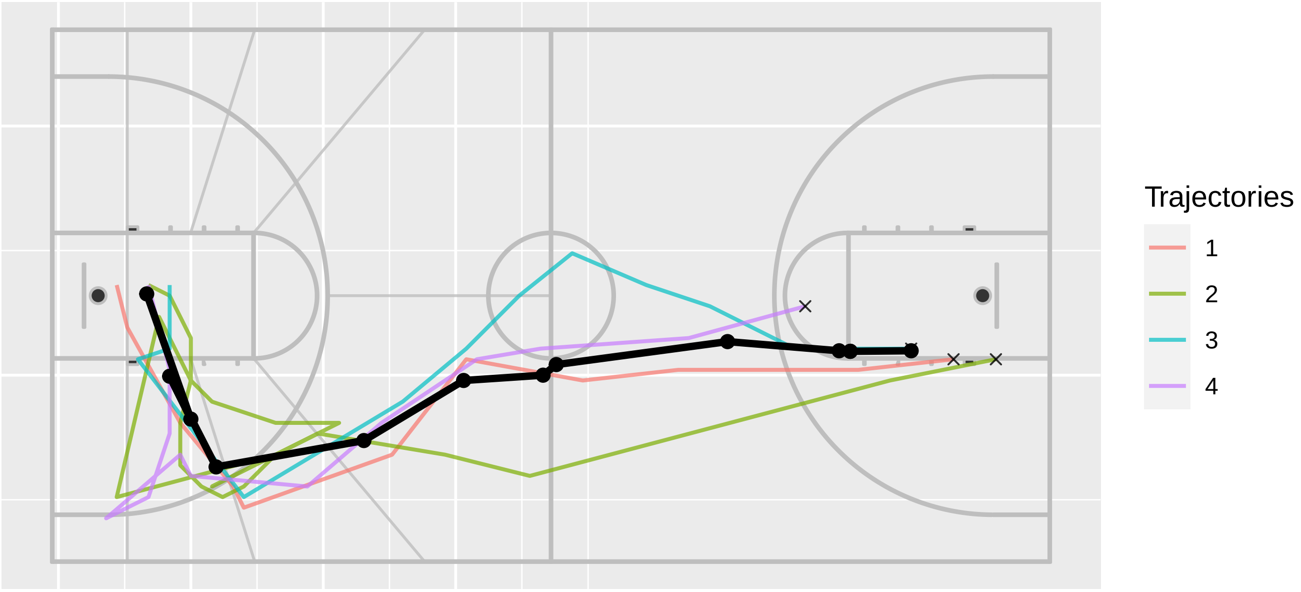

JPN Area 3 Cluster 21 : SelectTrajectories

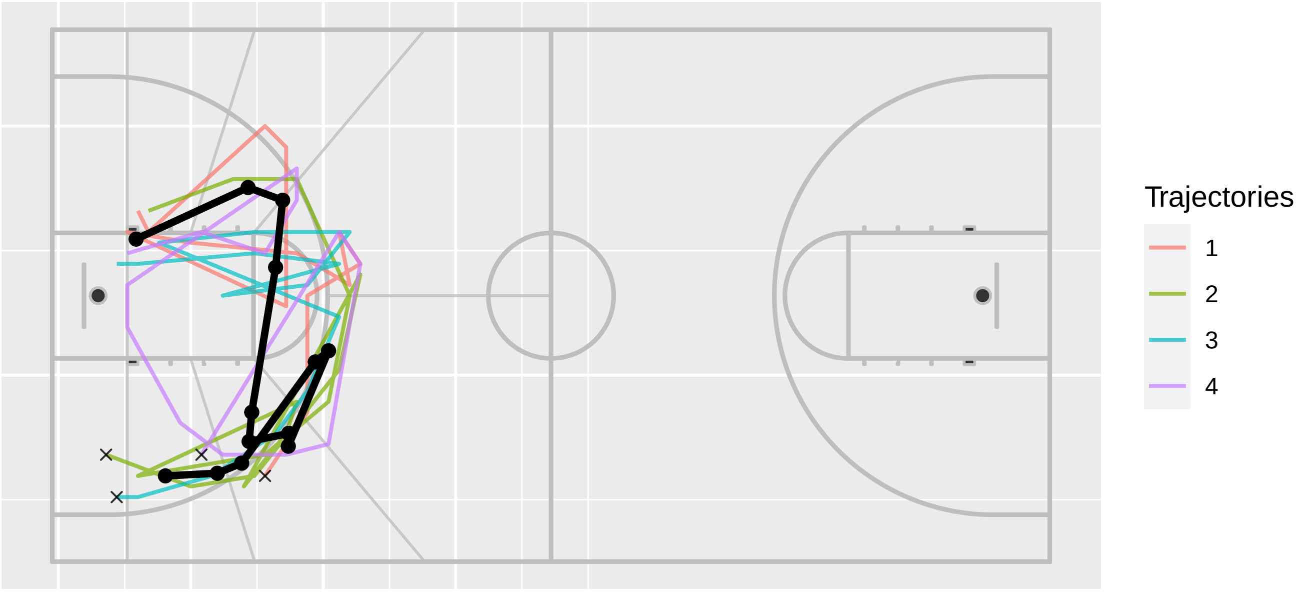

JPN Area 3 Cluster 22 : SelectTrajectories

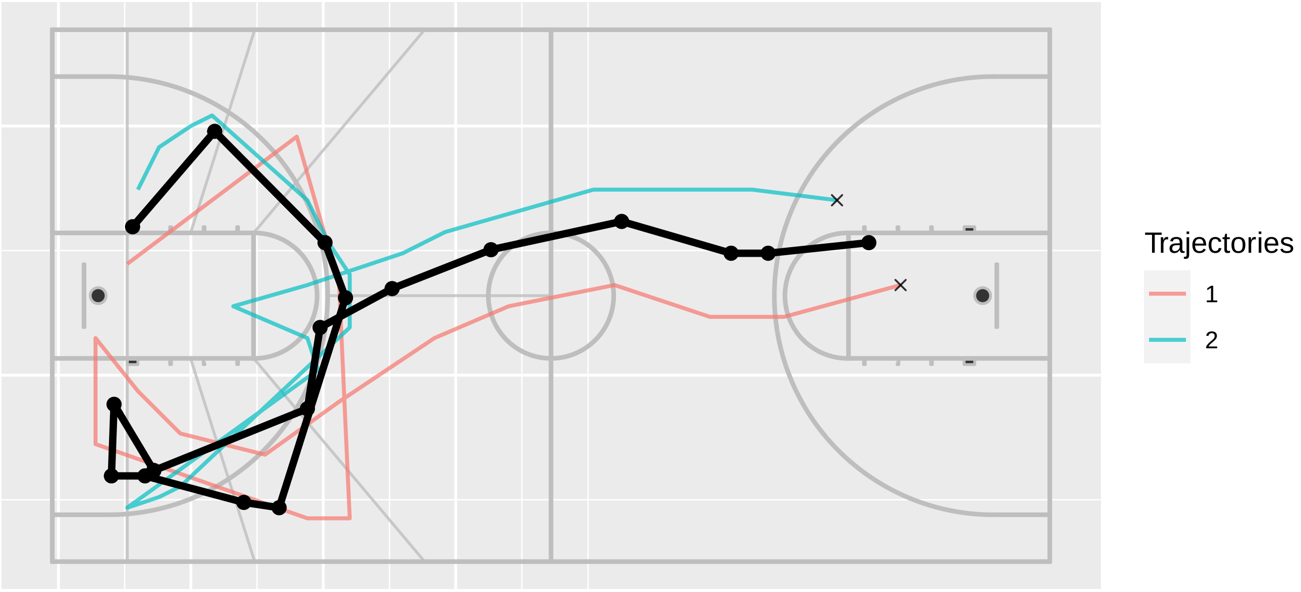

JPN Area 3 Cluster 23 : SelectTrajectories

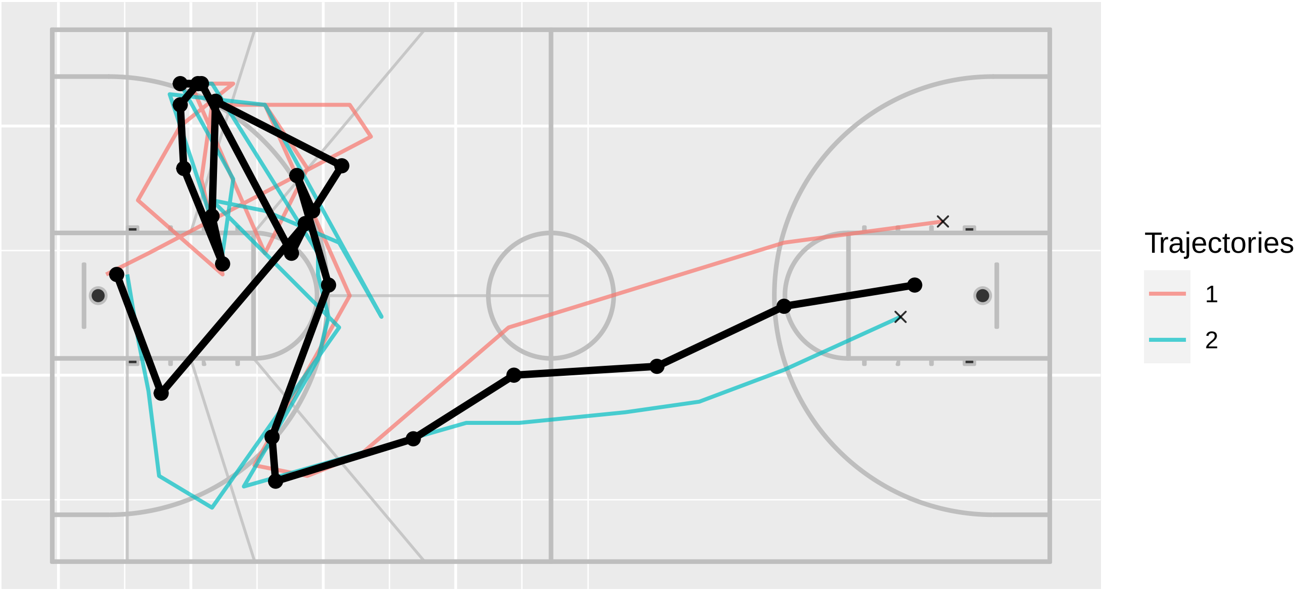

JPN Area 3 Cluster 24 : SelectTrajectories

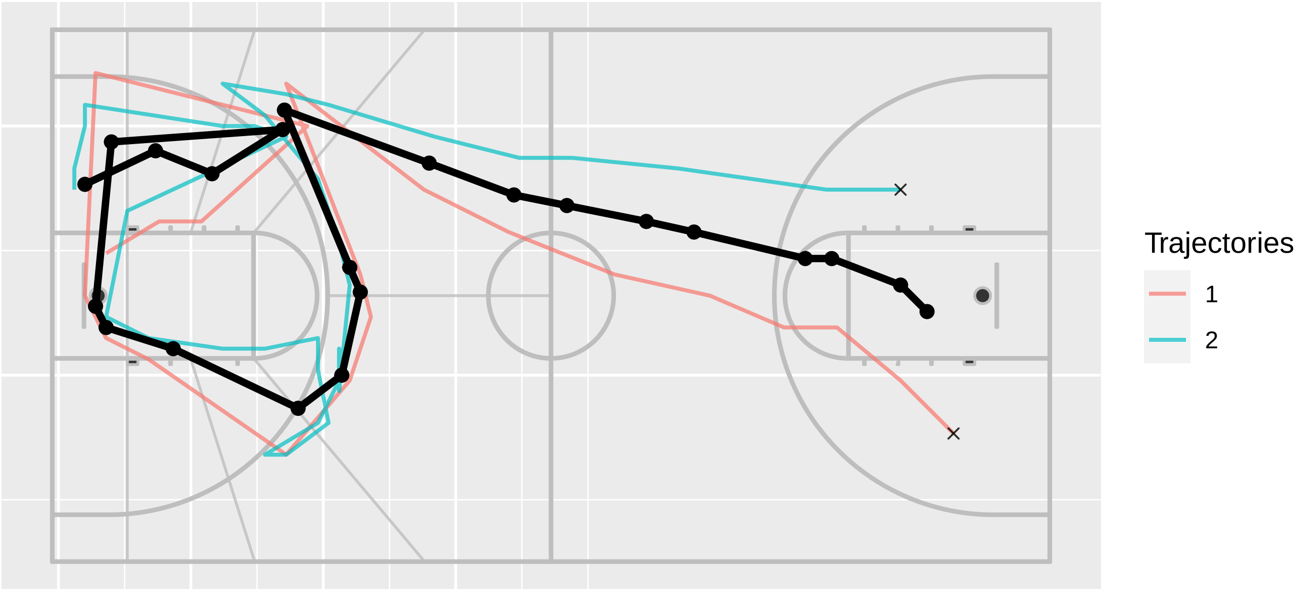

JPN Area 3 Cluster 25 : SelectTrajectories

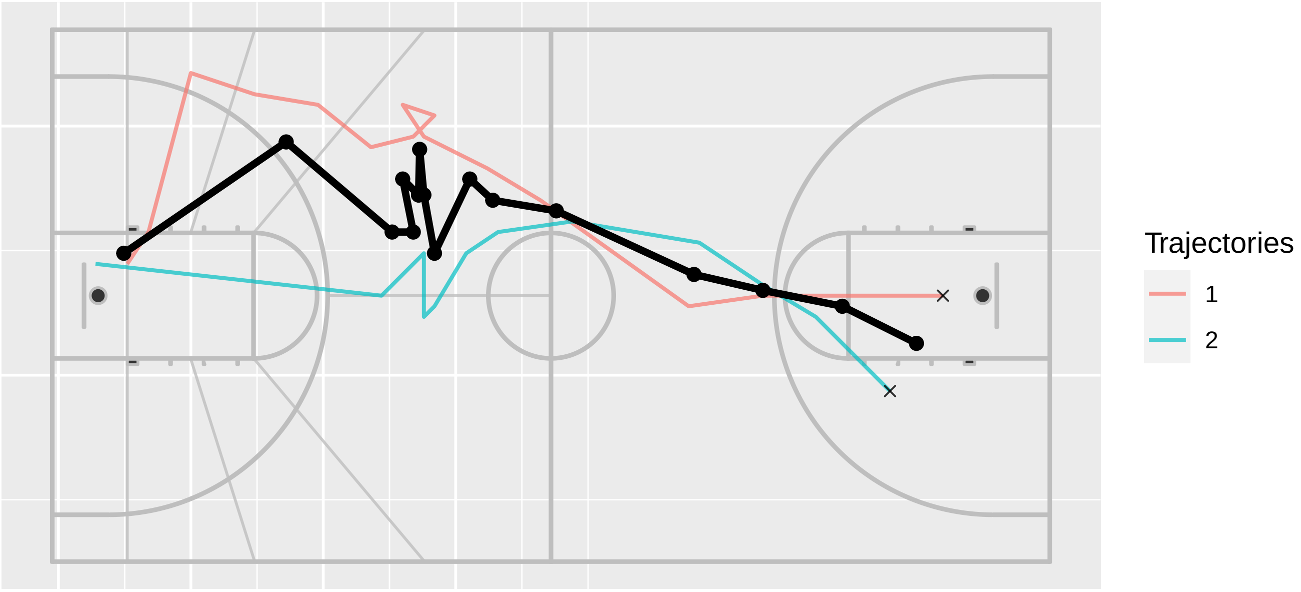

JPN Area 3 Cluster 26 : SelectTrajectories

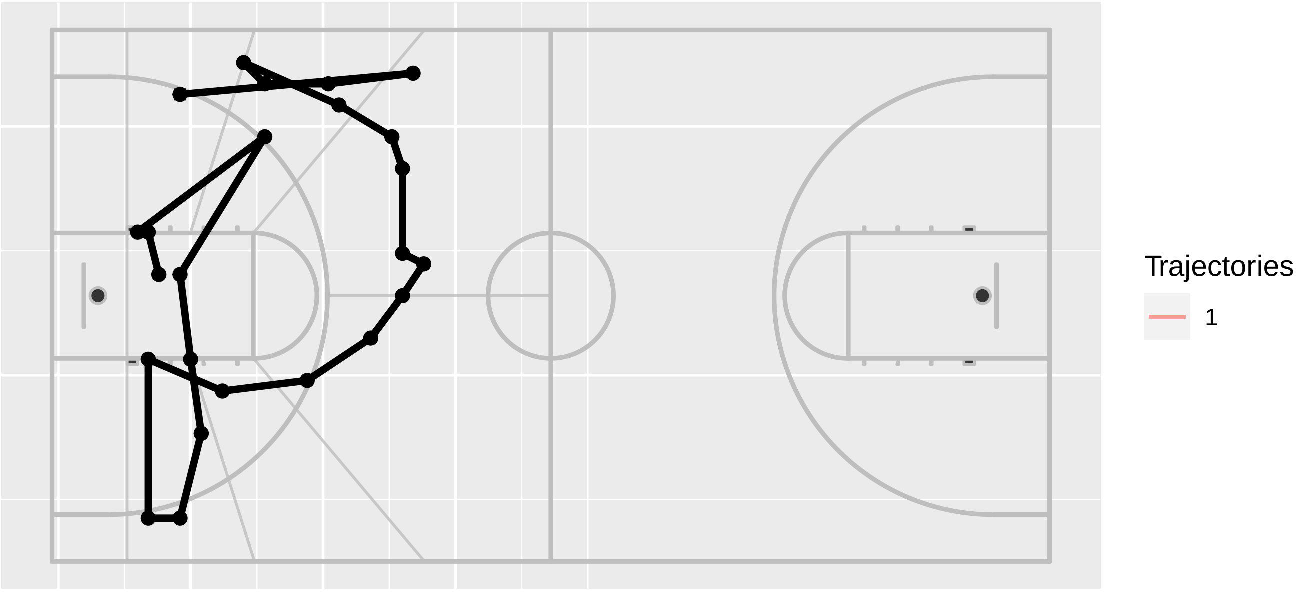

JPN Area 4 Cluster 1 : SelectTrajectories

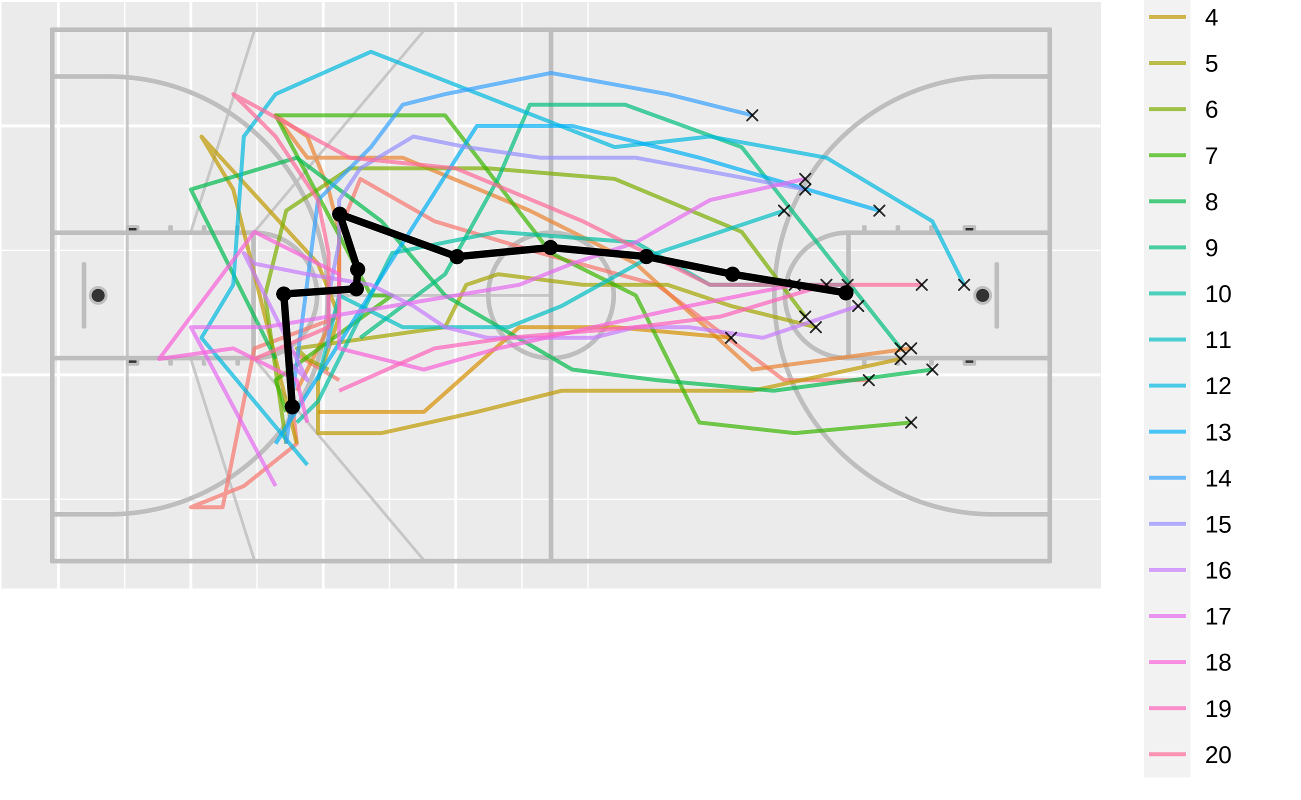

JPN Area 4 Cluster 2 : SelectTrajectories

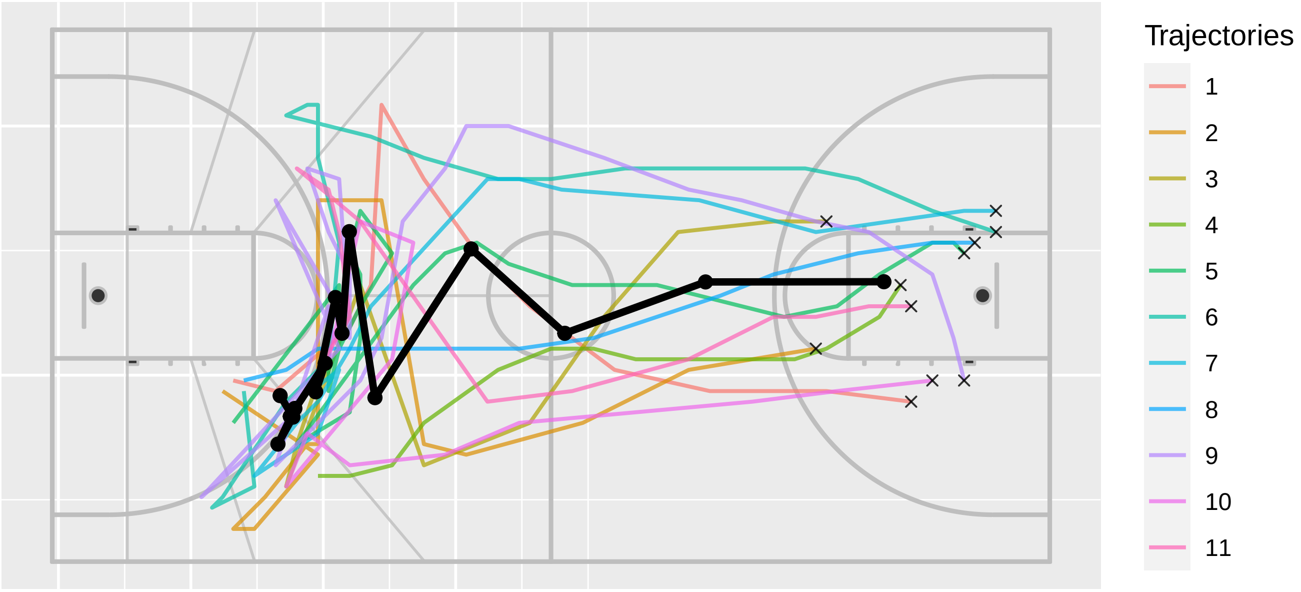

JPN Area 4 Cluster 3 : SelectTrajectories

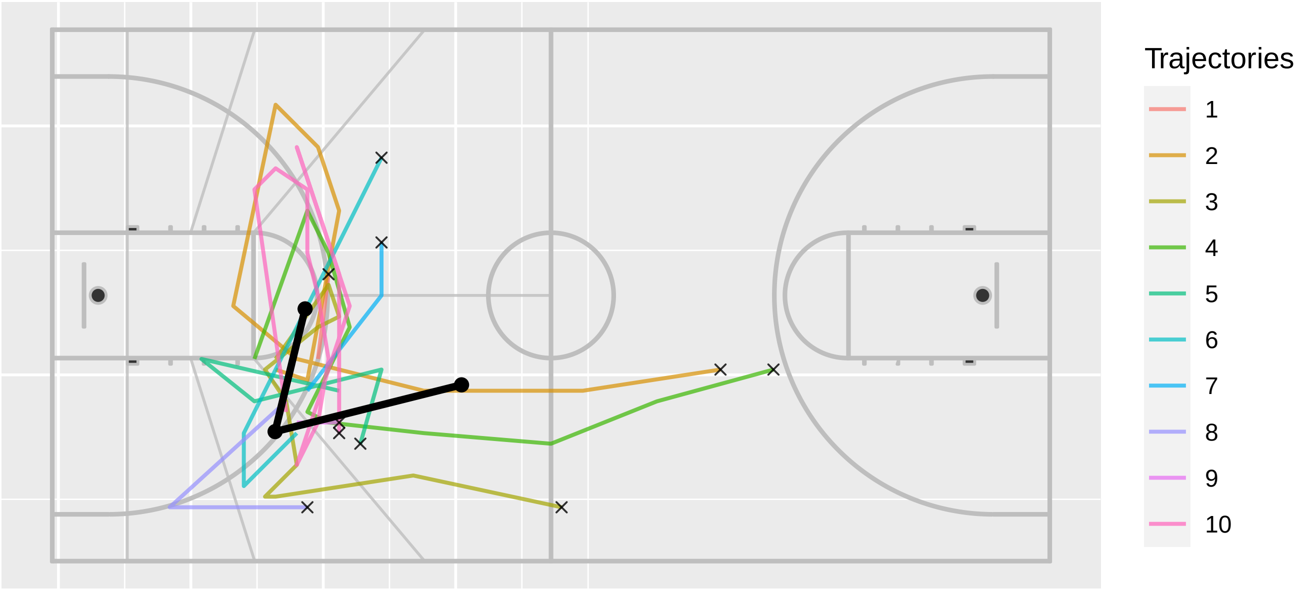

JPN Area 4 Cluster 4 : SelectTrajectories

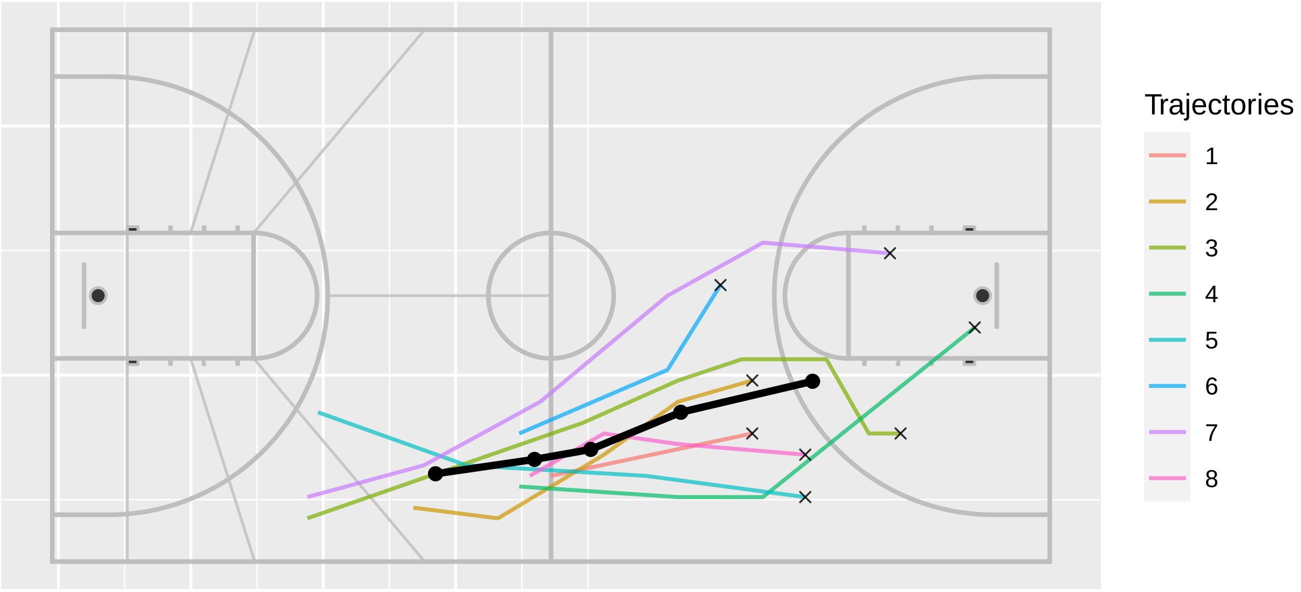

JPN Area 4 Cluster 5 : SelectTrajectories

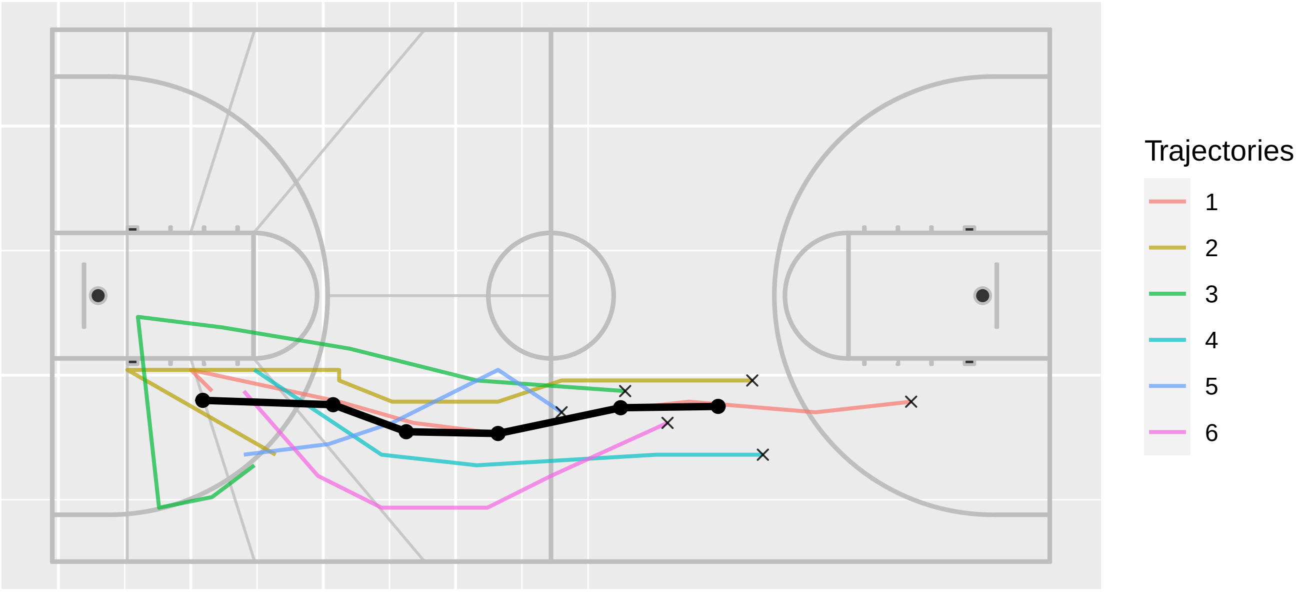

JPN Area 4 Cluster 6 : SelectTrajectories

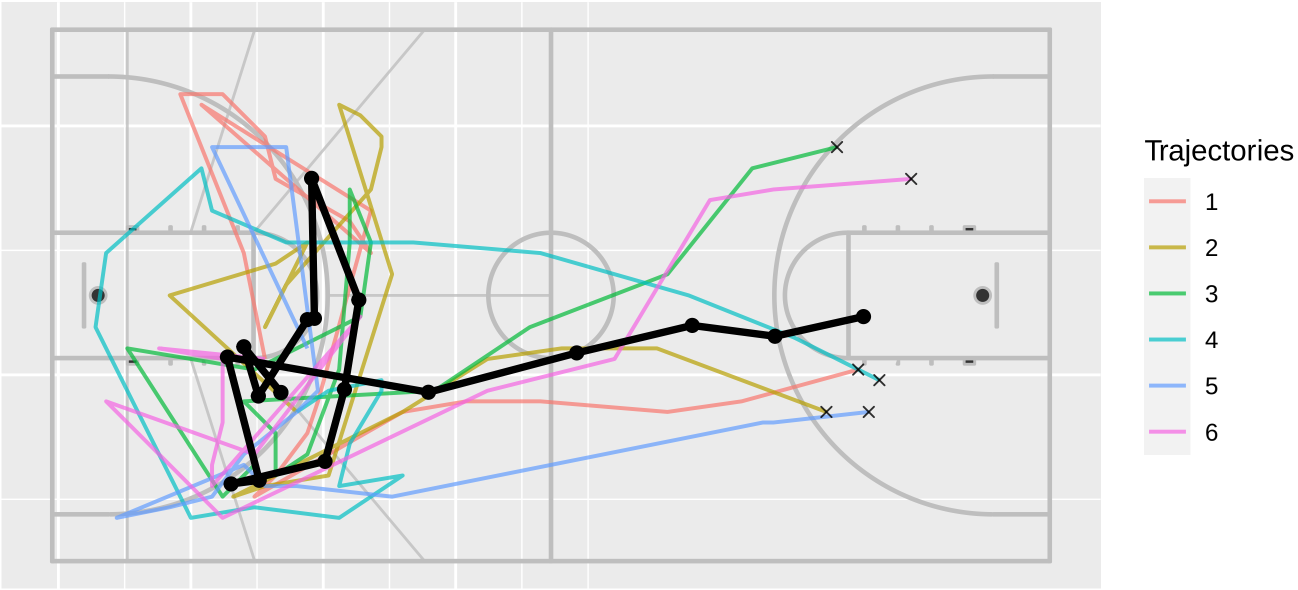

JPN Area 4 Cluster 7 : SelectTrajectories

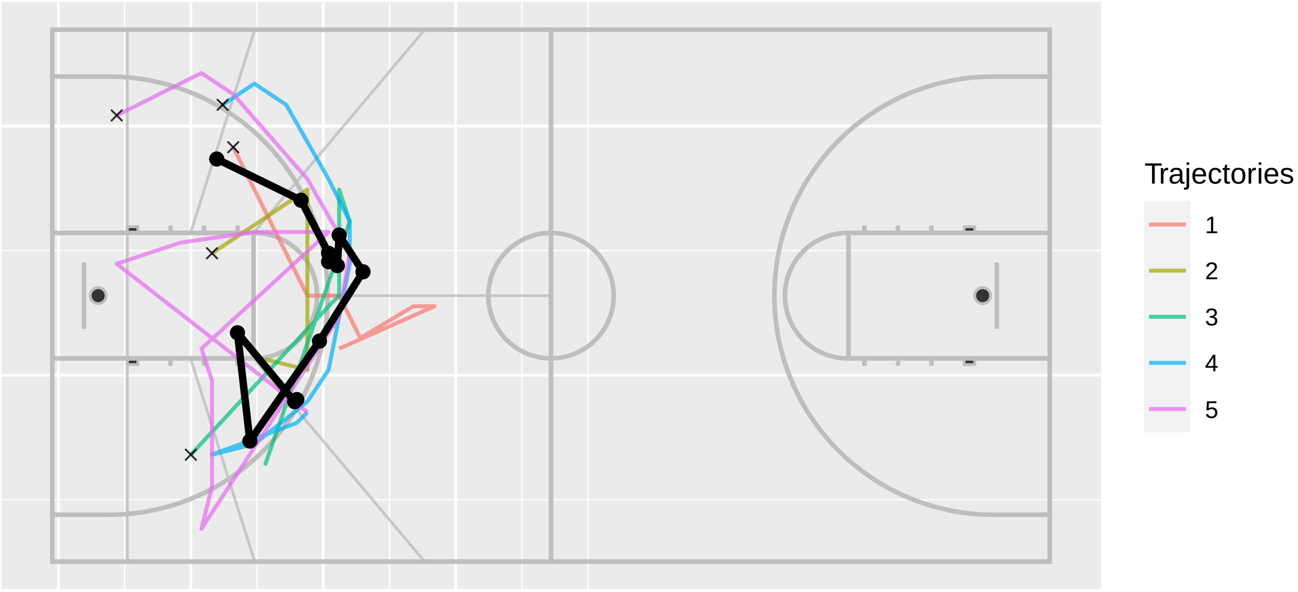

JPN Area 4 Cluster 8 : SelectTrajectories

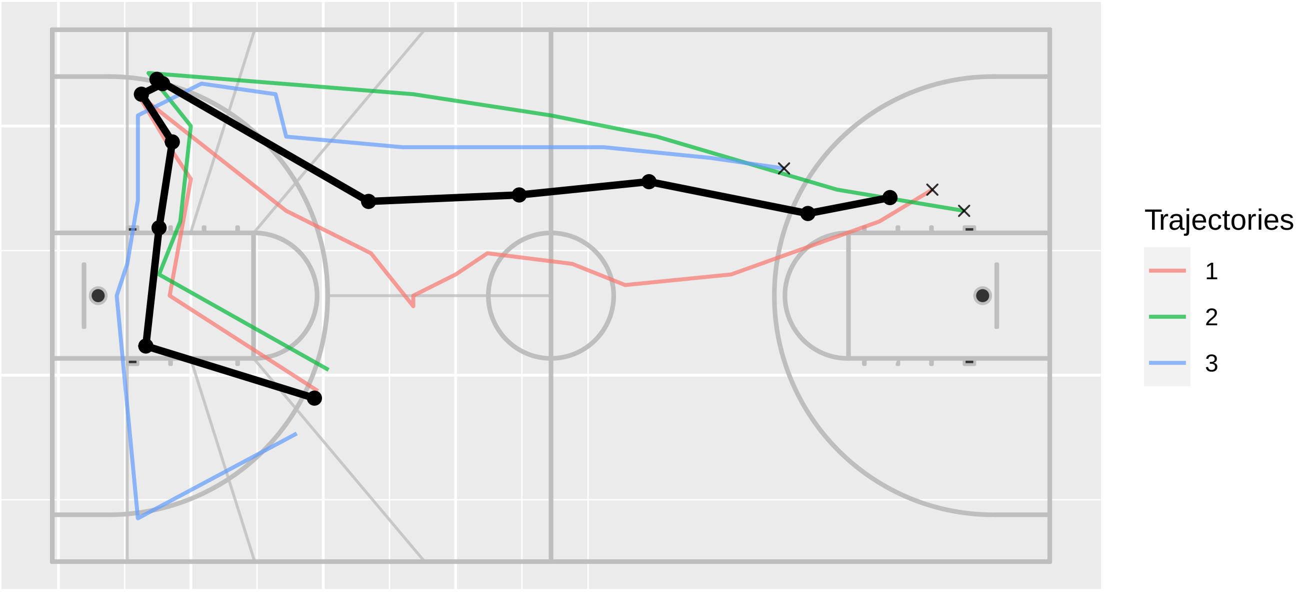

JPN Area 4 Cluster 9 : SelectTrajectories

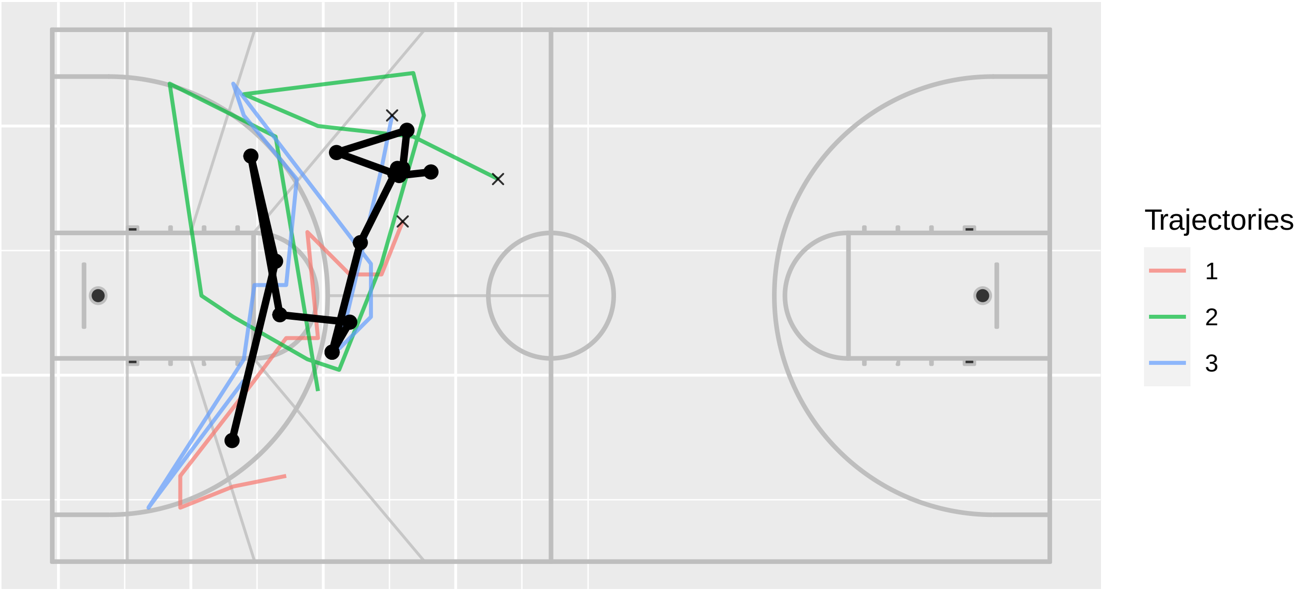

JPN Area 4 Cluster 10 : SelectTrajectories

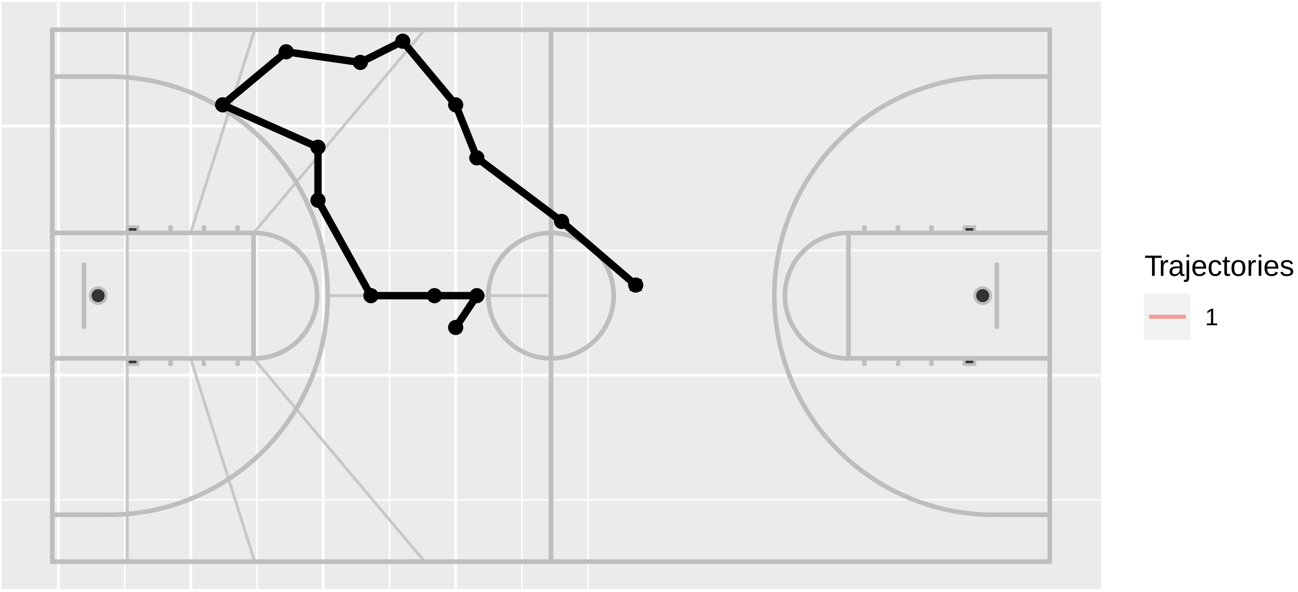

JPN Area 5 Cluster 1 : SelectTrajectories

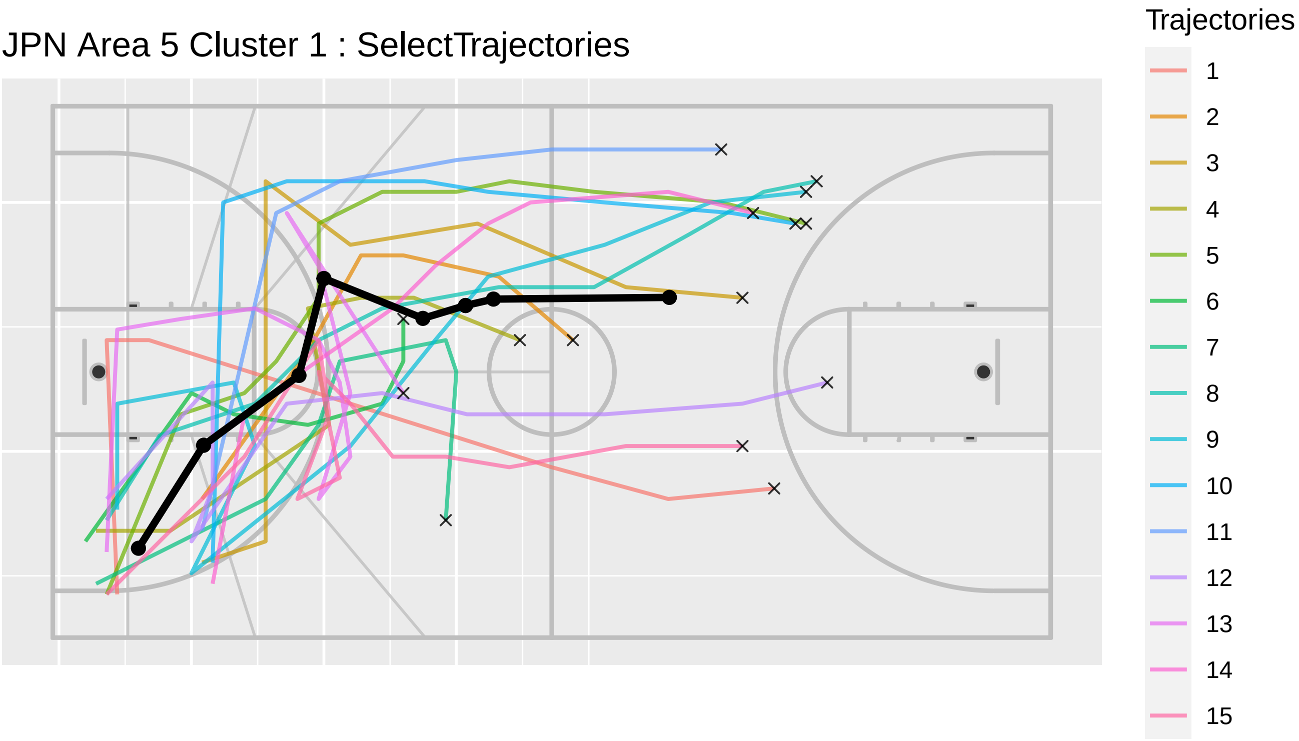

JPN Area 5 Cluster 2 : SelectTrajectories

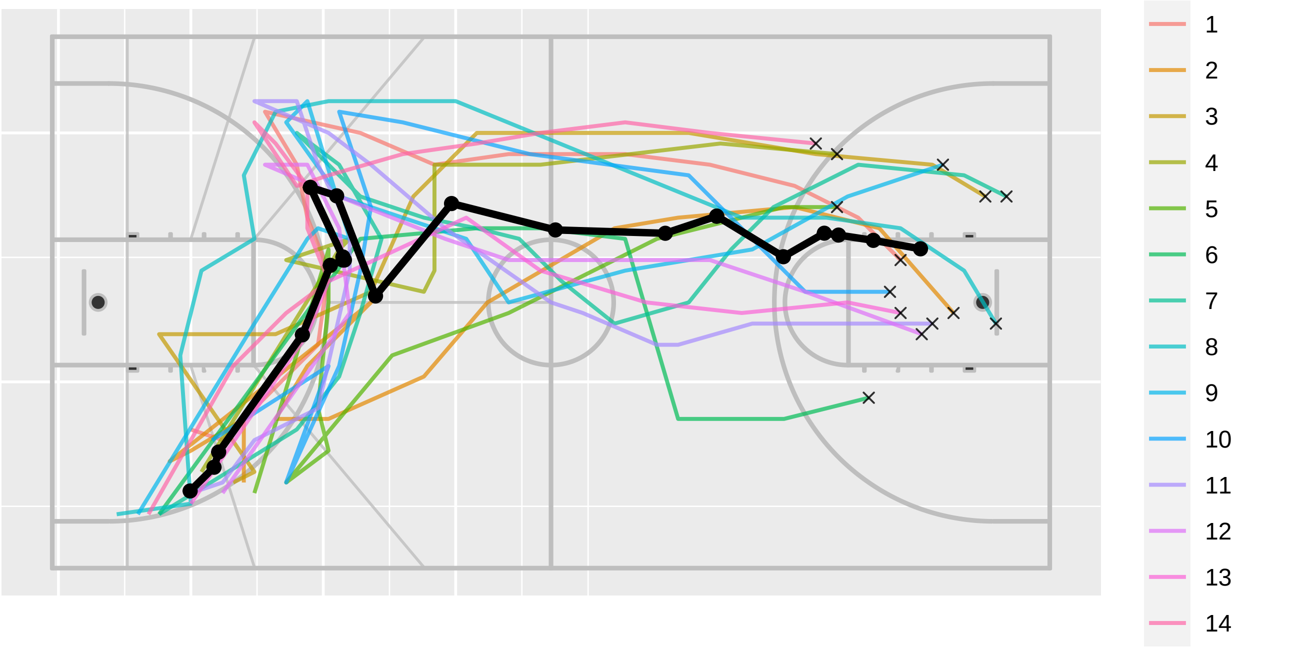

JPN Area 5 Cluster 3 : SelectTrajectories

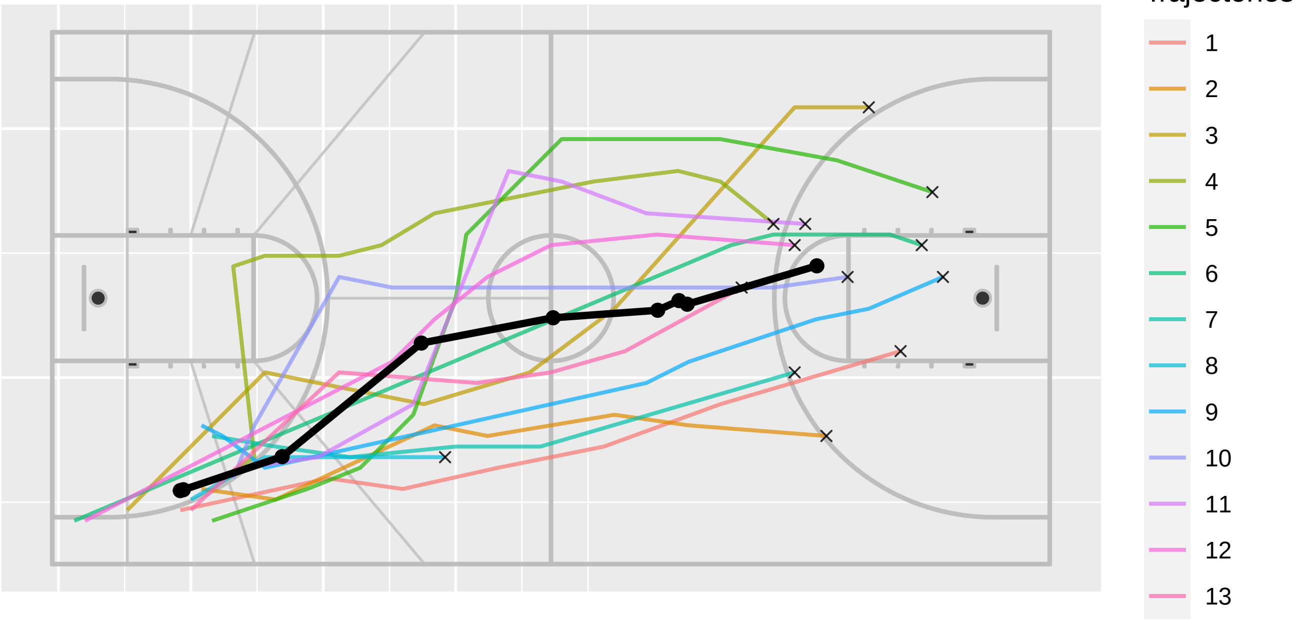

JPN Area 5 Cluster 4 : SelectTrajectories

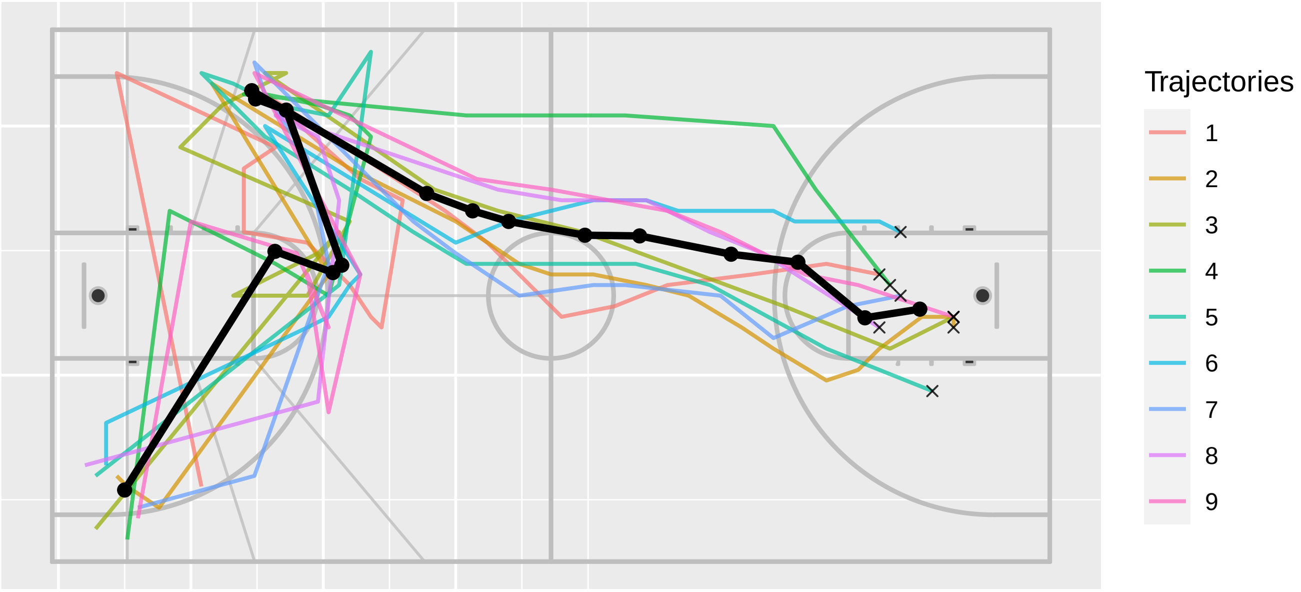

JPN Area 5 Cluster 5 : SelectTrajectories

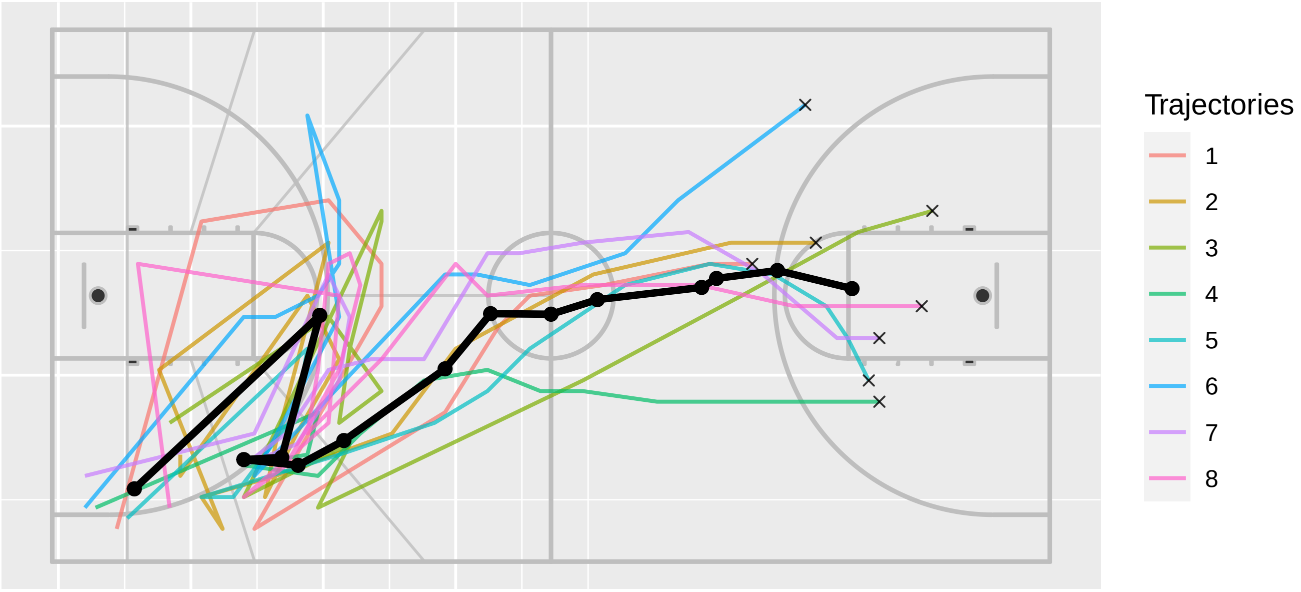

JPN Area 5 Cluster 6 : SelectTrajectories

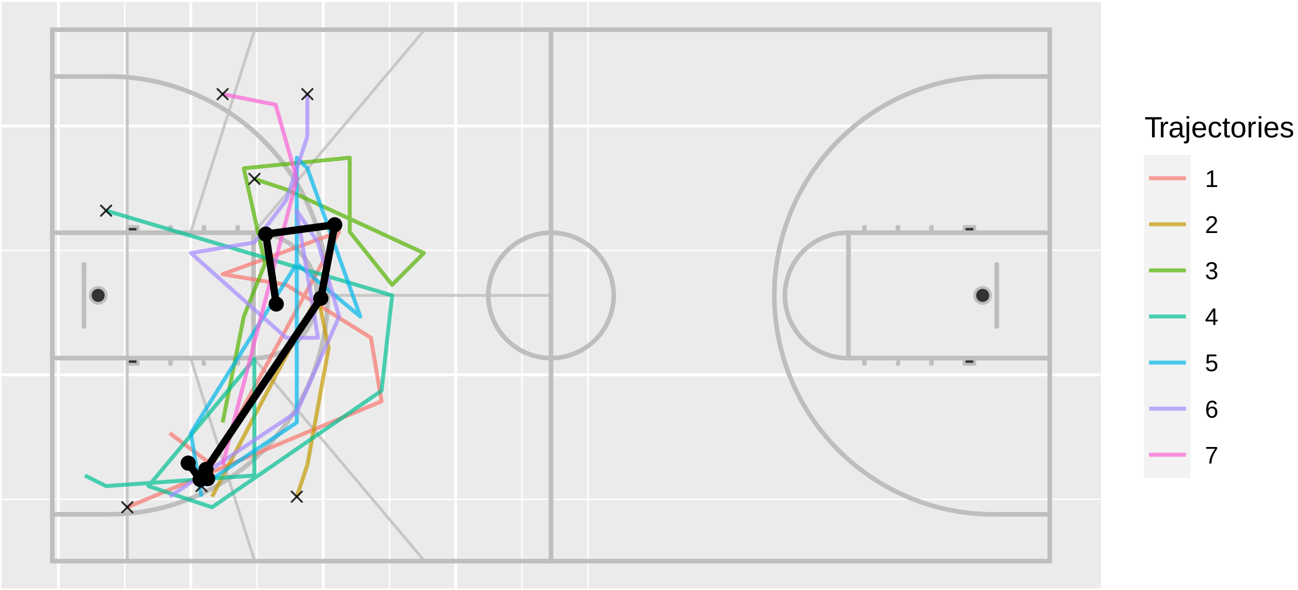

JPN Area 5 Cluster 7 : SelectTrajectories

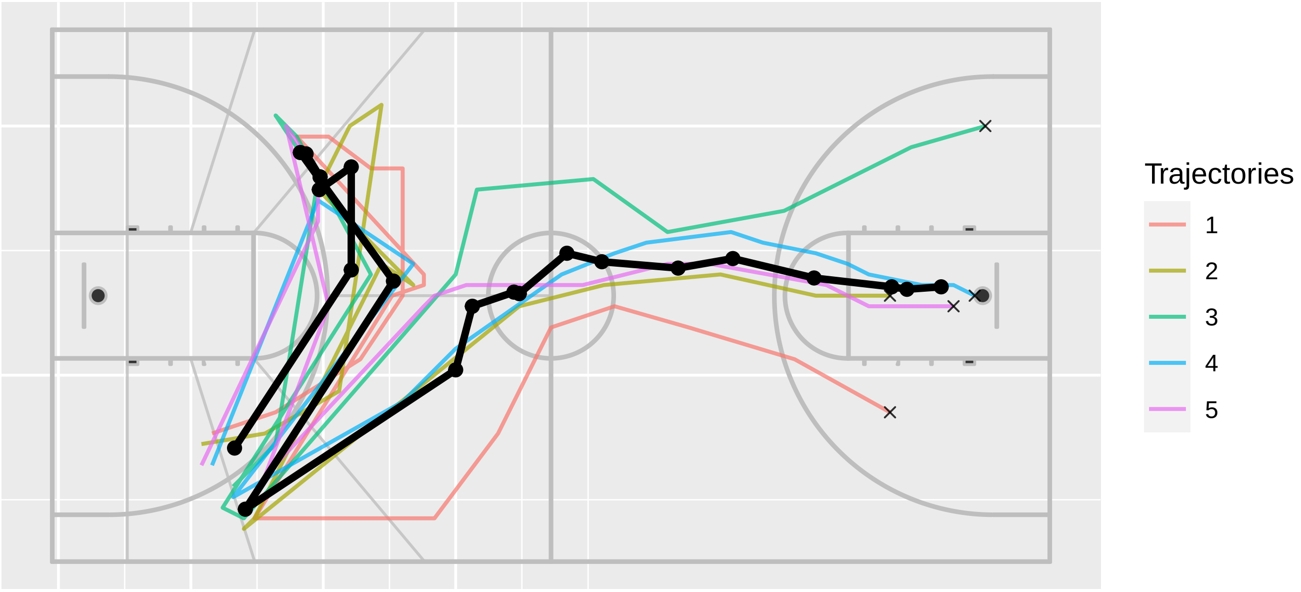

JPN Area 5 Cluster 8 : SelectTrajectories

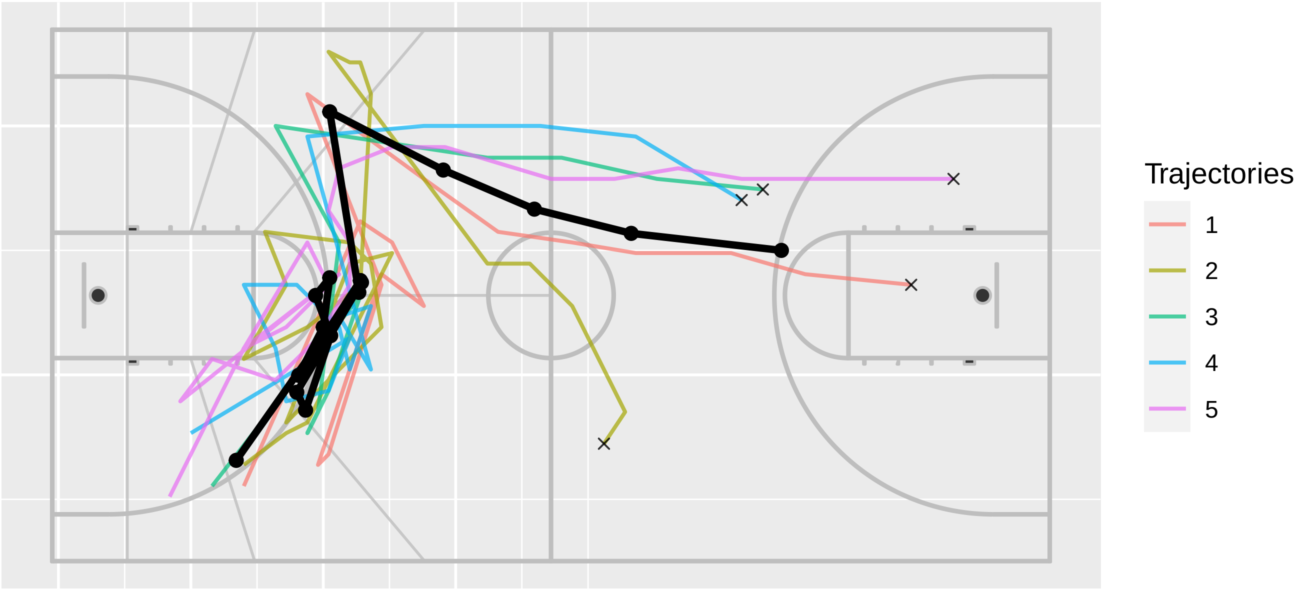

JPN Area 5 Cluster 9 : SelectTrajectories

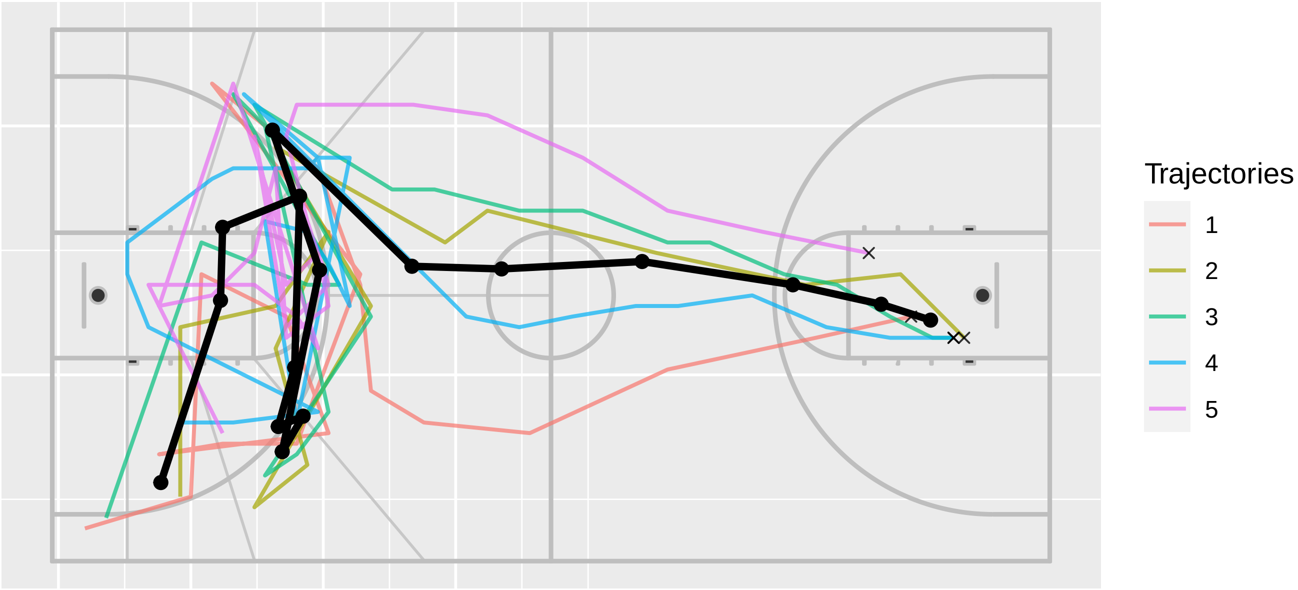

JPN Area 5 Cluster 10 : SelectTrajectories

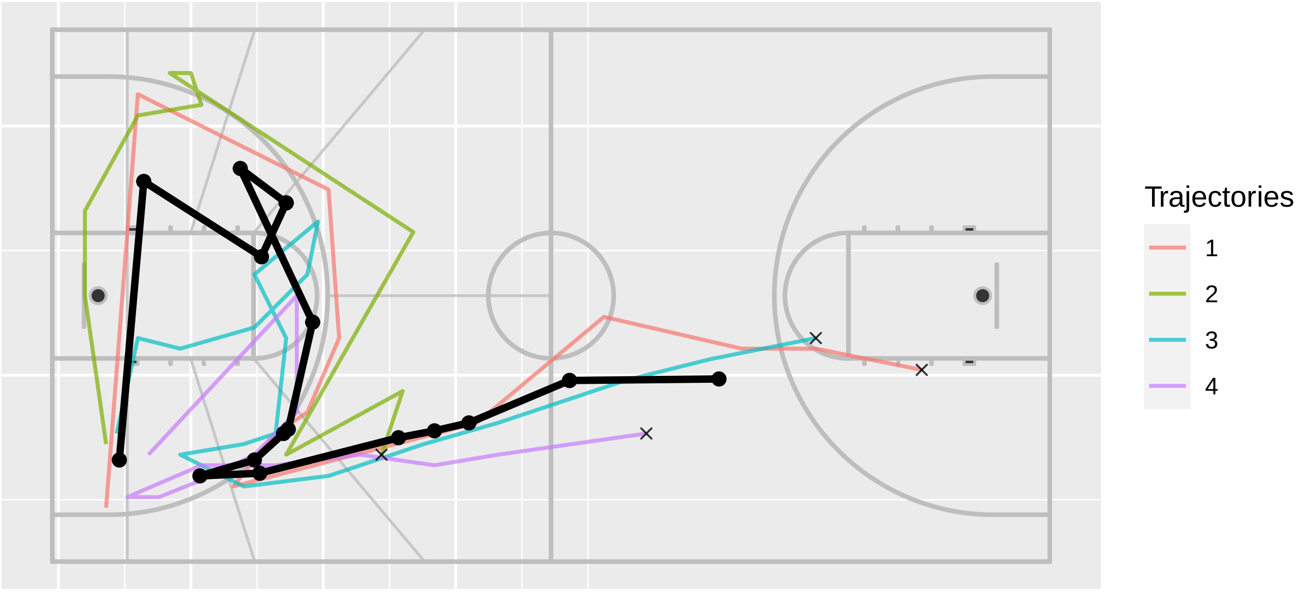

JPN Area 5 Cluster 11 : SelectTrajectories

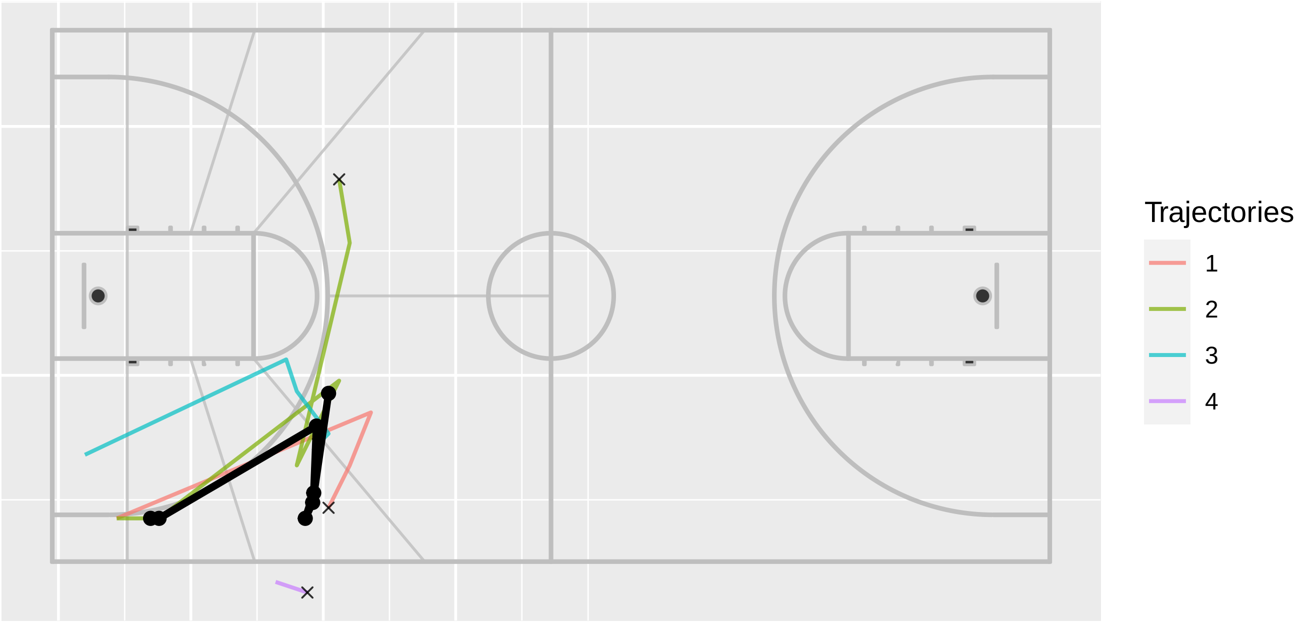

JPN Area 5 Cluster 12 : SelectTrajectories

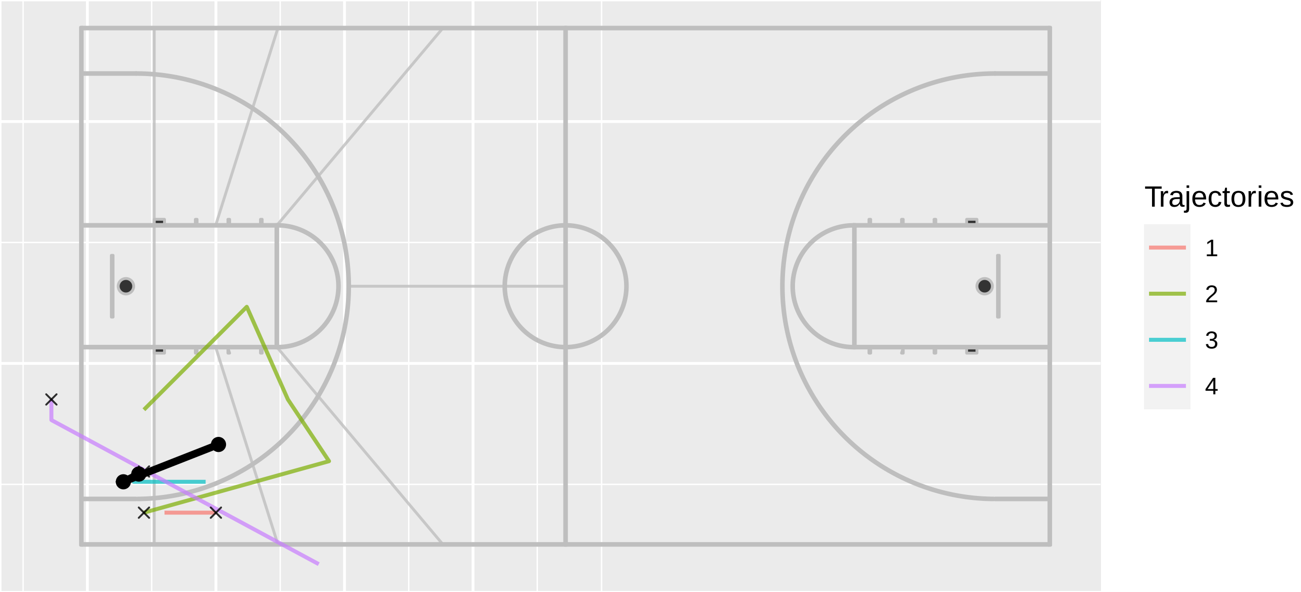

JPN Area 5 Cluster 13 : SelectTrajectories

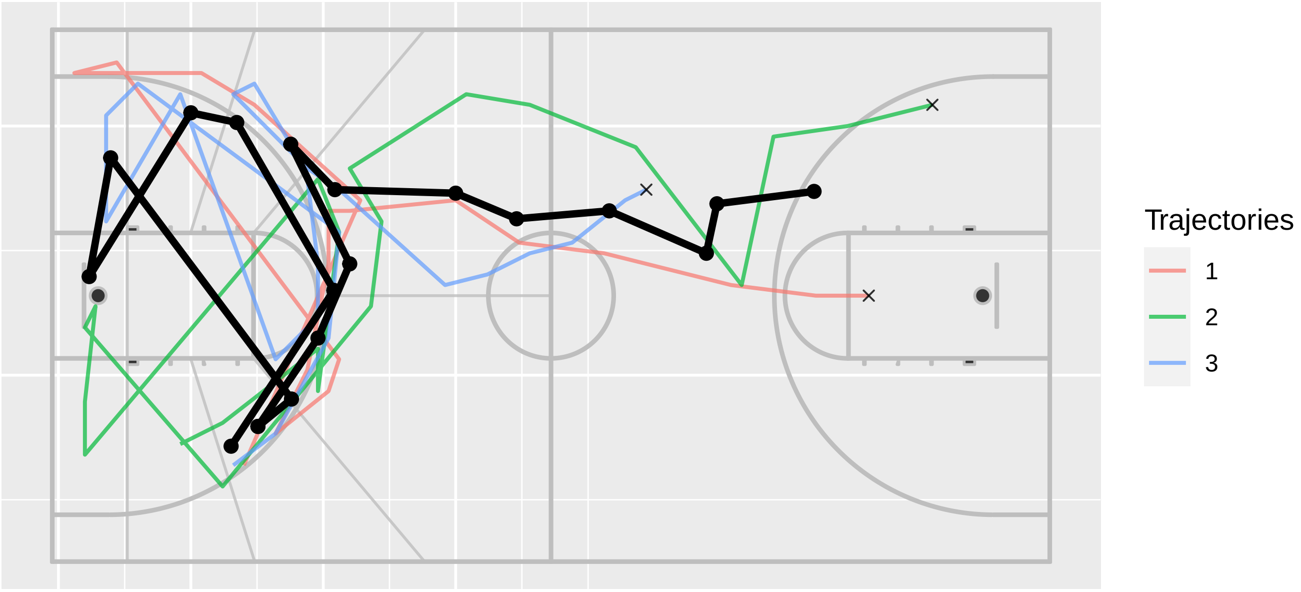

JPN Area 5 Cluster 14 : SelectTrajectories

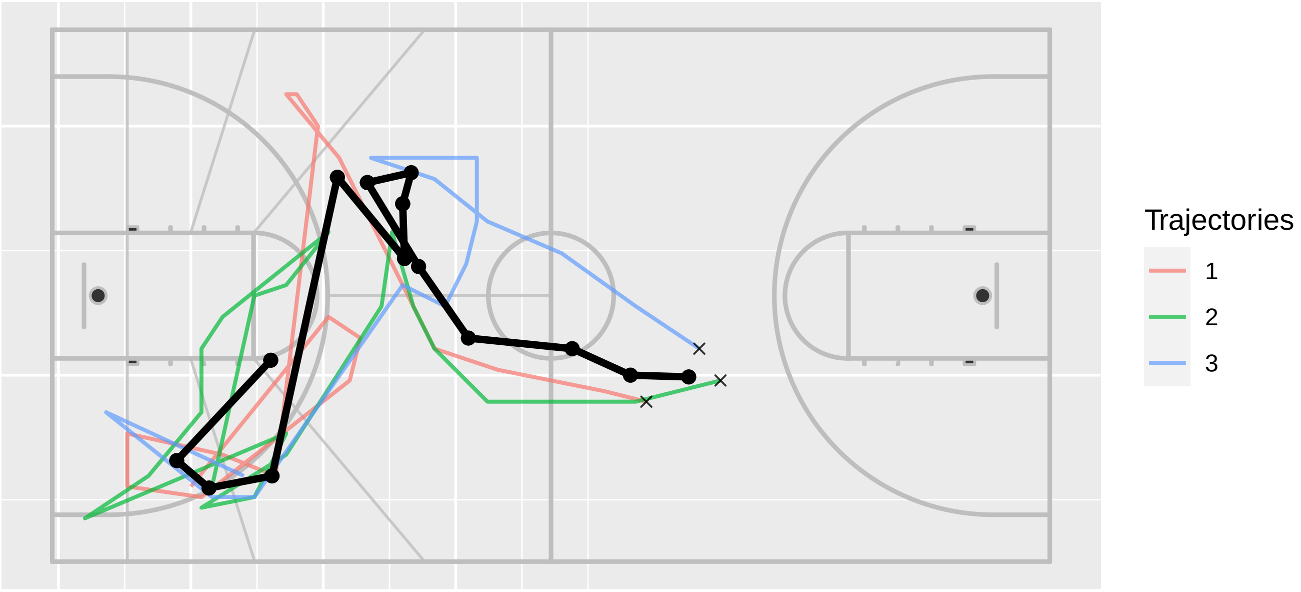

JPN Area 5 Cluster 15 : SelectTrajectories

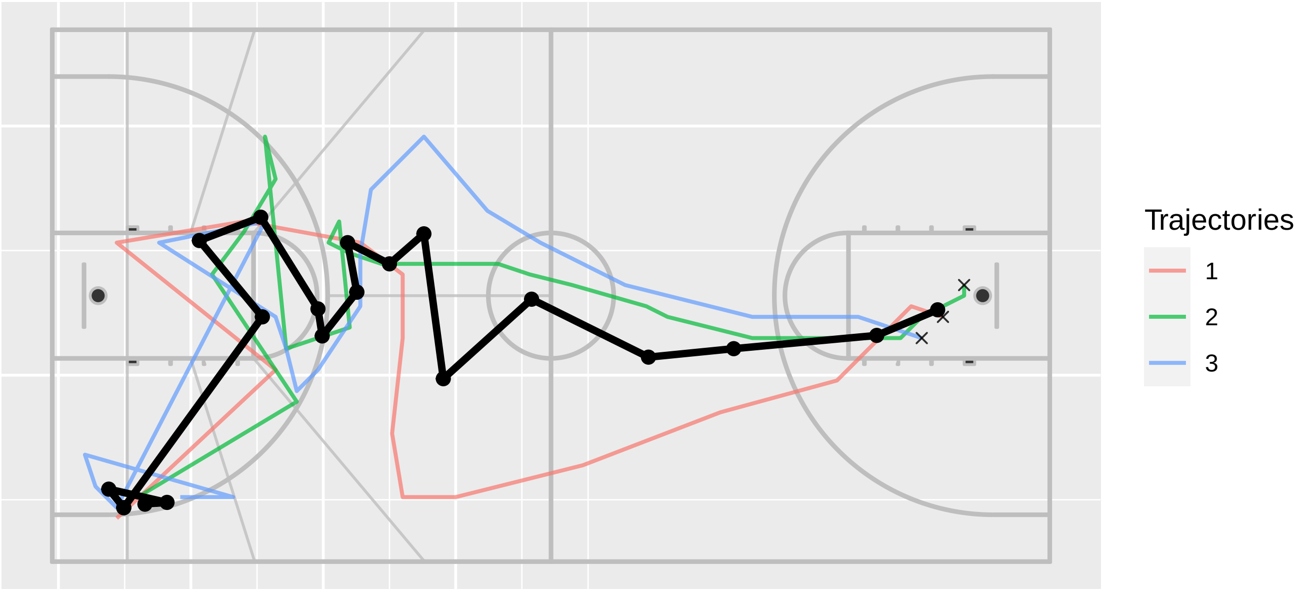

JPN Area 5 Cluster 16 : SelectTrajectories

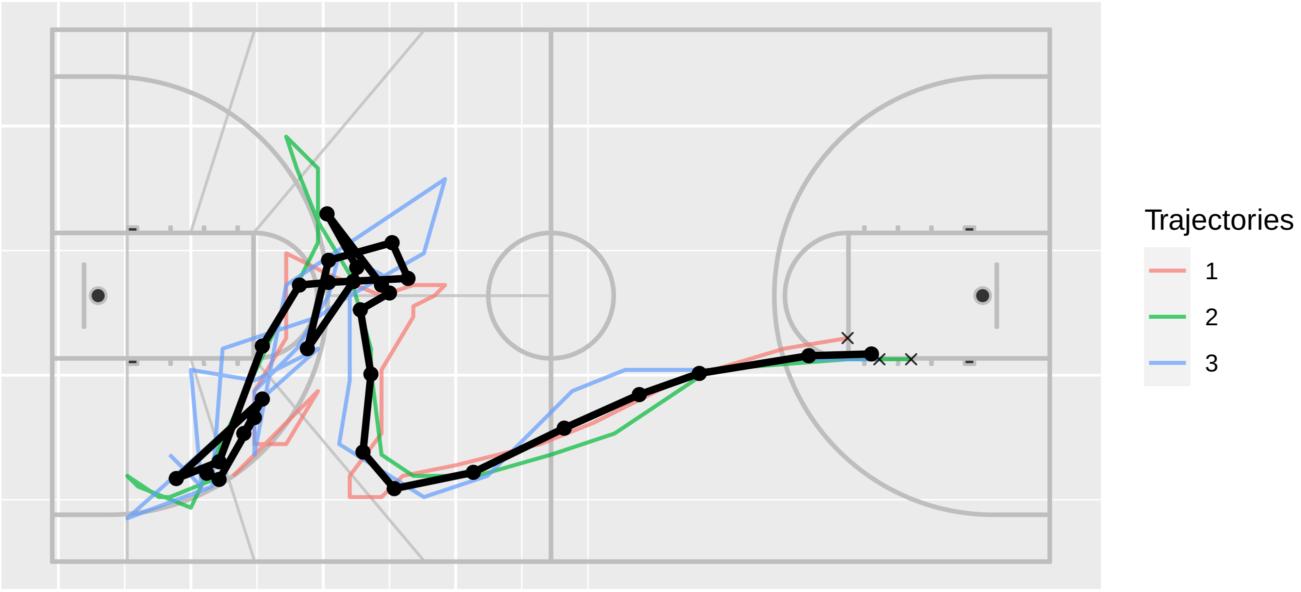

JPN Area 5 Cluster 17 : SelectTrajectories

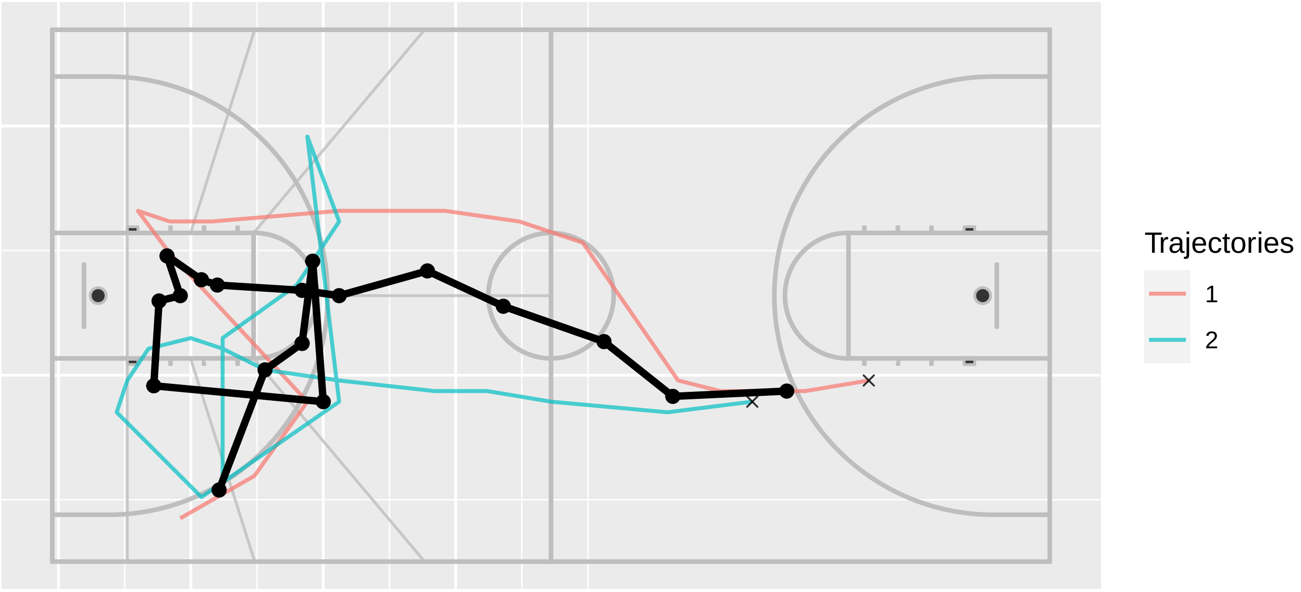

JPN Area 5 Cluster 18 : SelectTrajectories

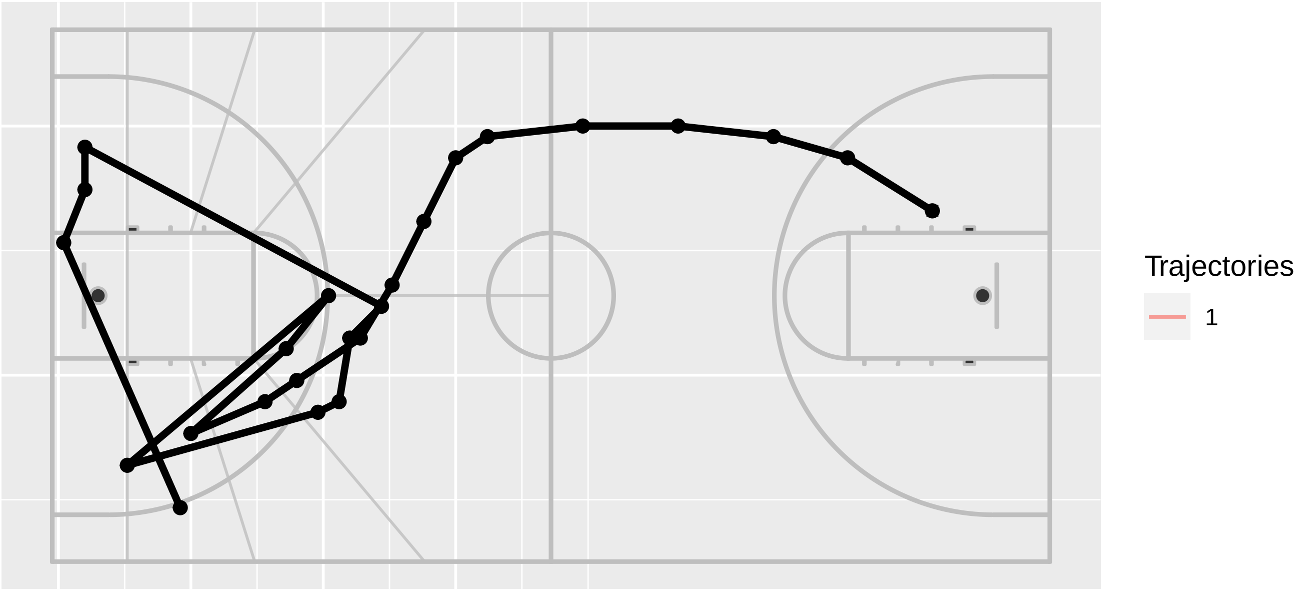

JPN Area 6 Cluster 1 : SelectTrajectories

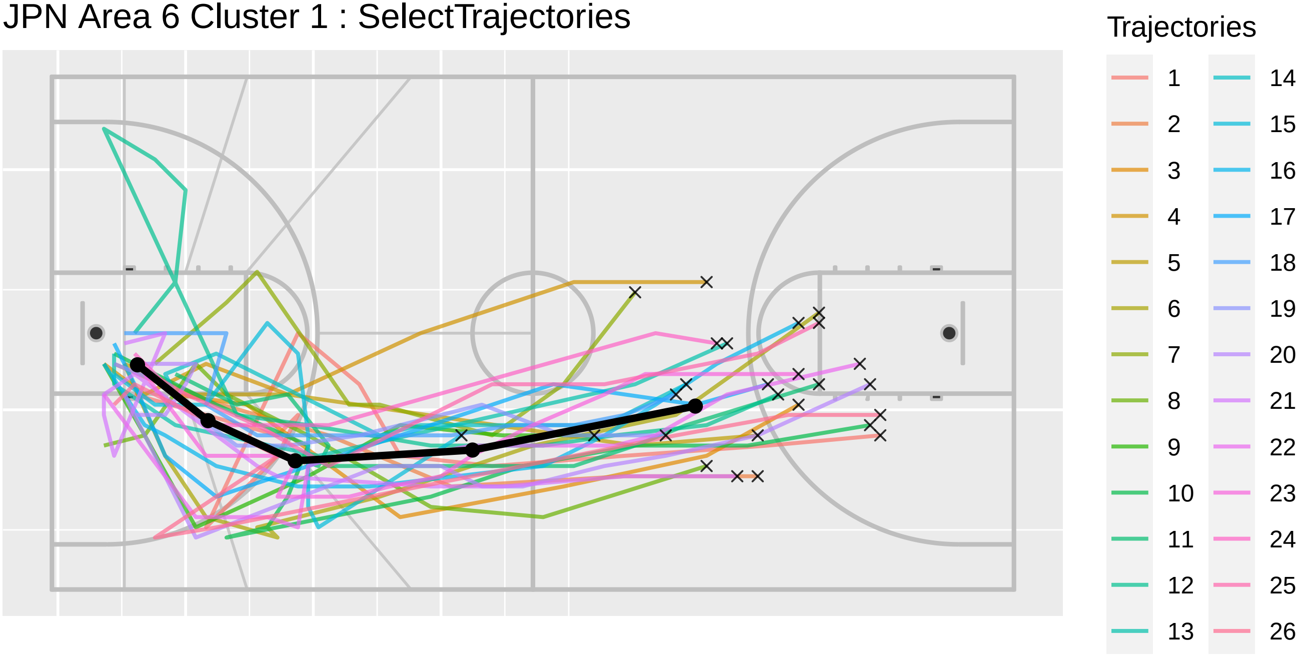

JPN Area 6 Cluster 2 : SelectTrajectories

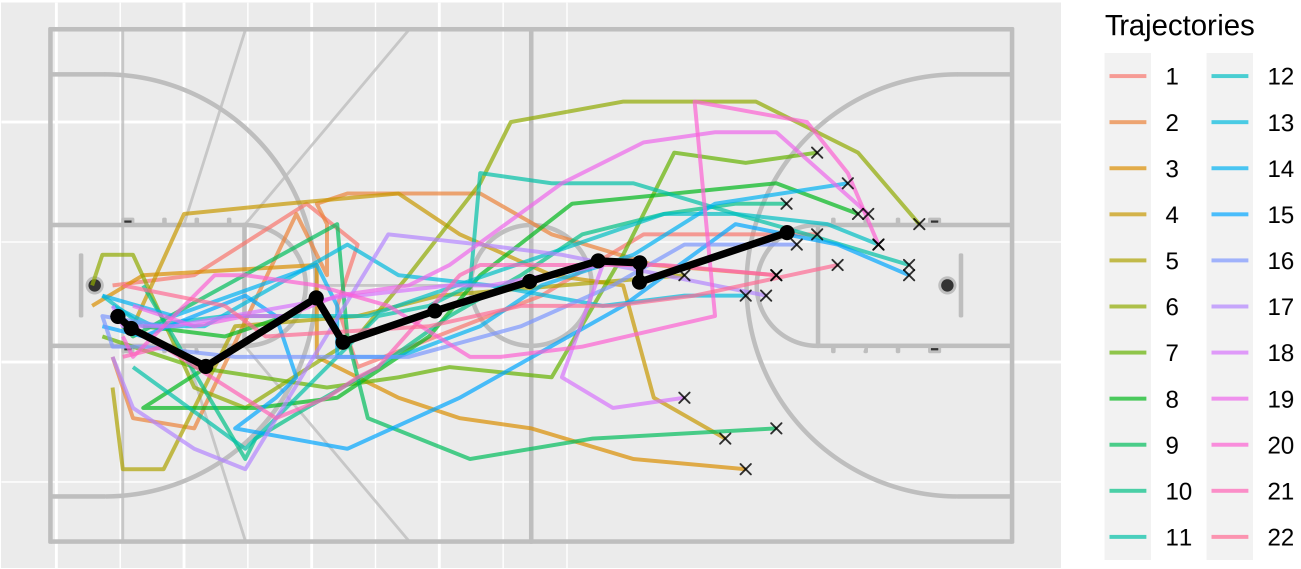

JPN Area 6 Cluster 3 : SelectTrajectories

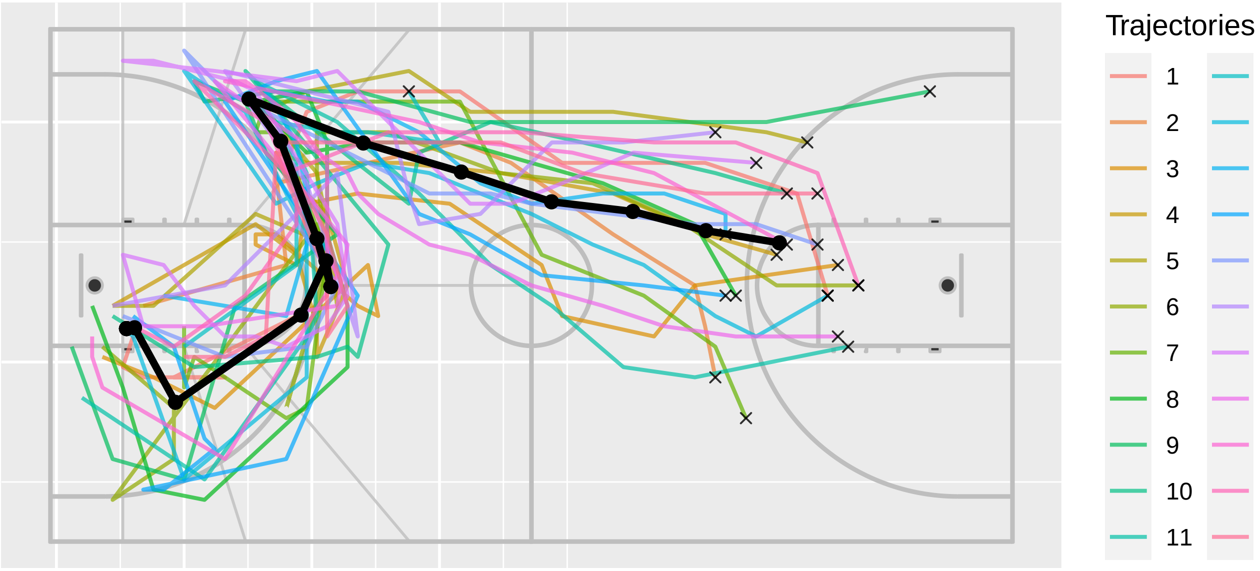

JPN Area 6 Cluster 4 : SelectTrajectories

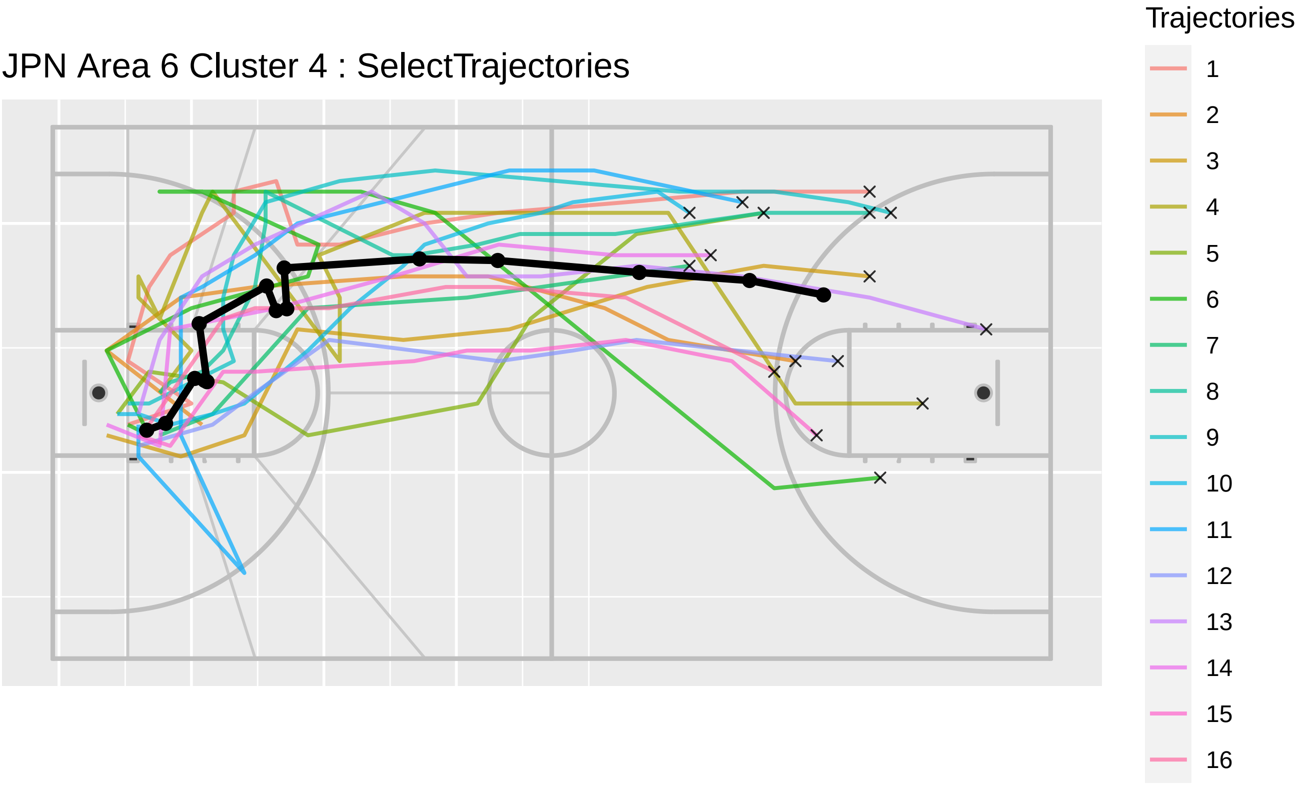

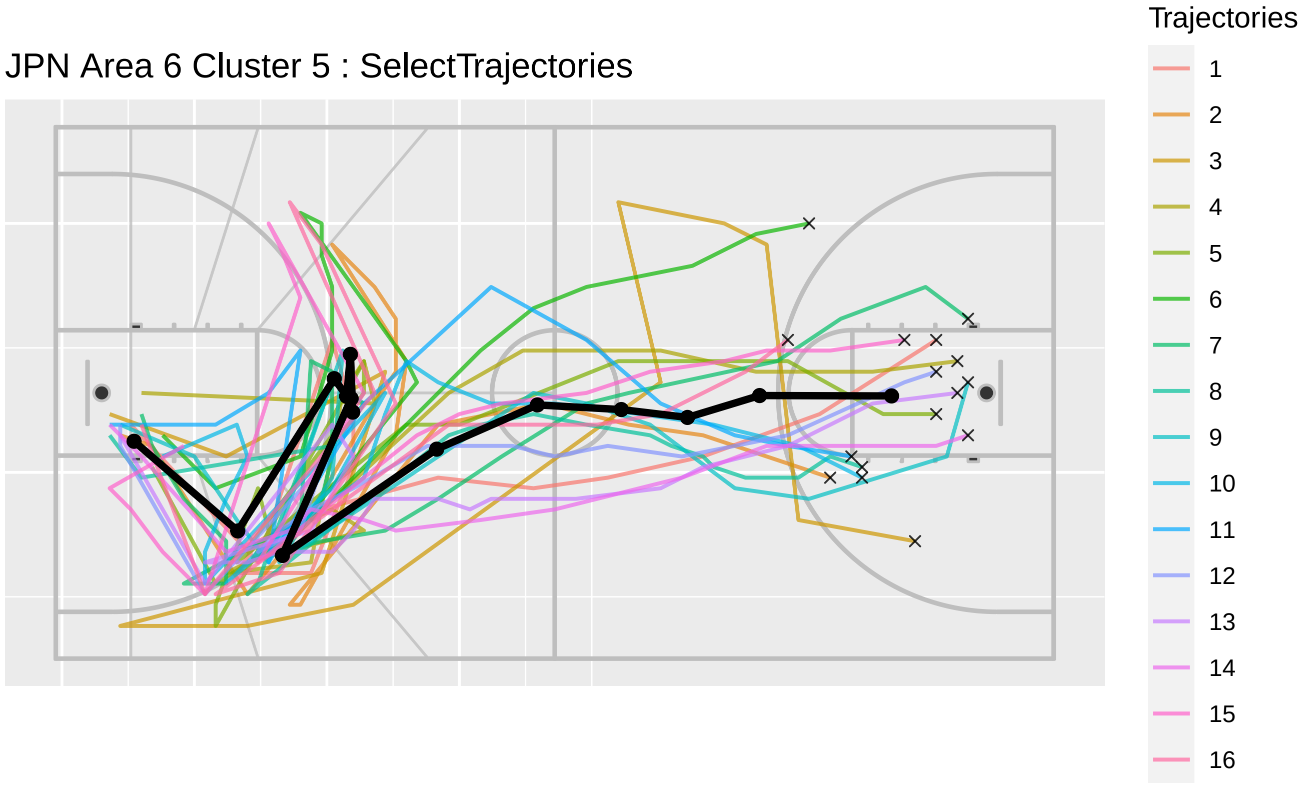
JPN Area 6 Cluster 5 : SelectTrajectories
Trajectories
1
2
3
4
5
6
7
8
9
10
11
12
13
14
15
16

JPN Area 6 Cluster 6 : SelectTrajectories

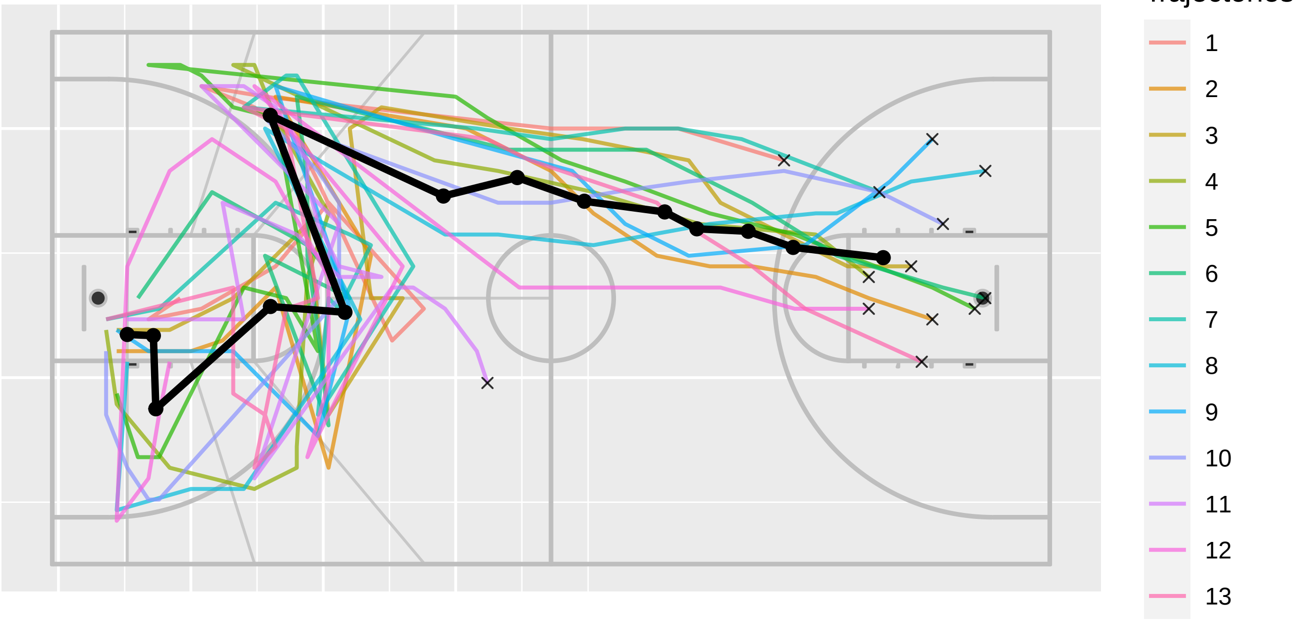

JPN Area 6 Cluster 7 : SelectTrajectories

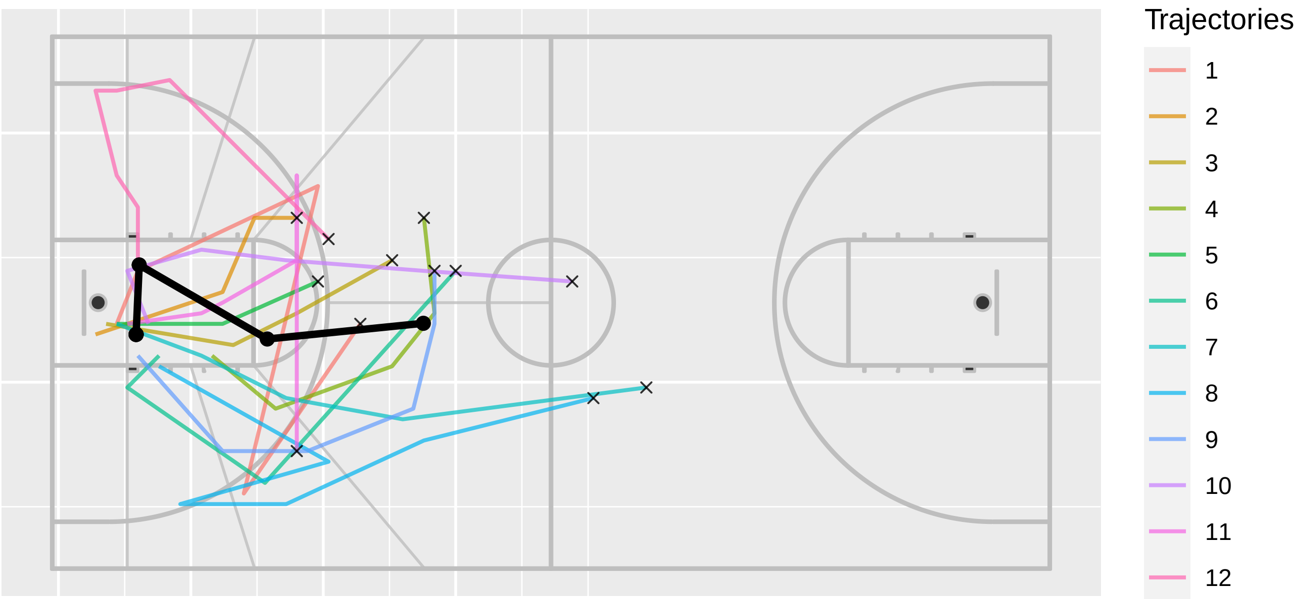

JPN Area 6 Cluster 8 : SelectTrajectories

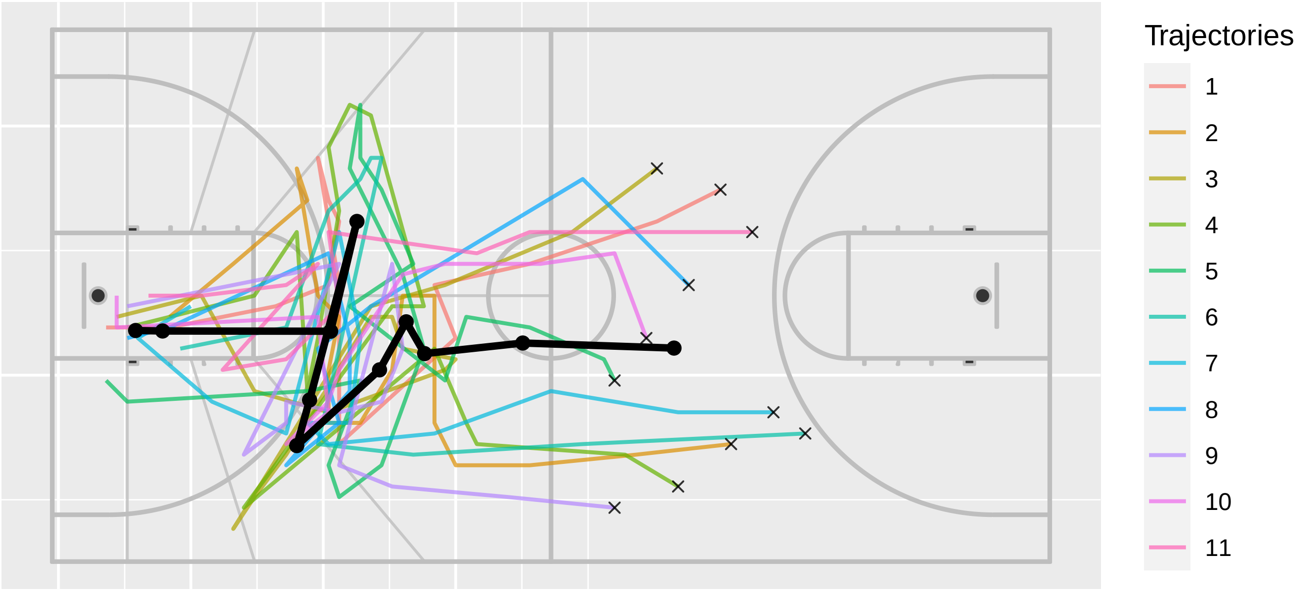

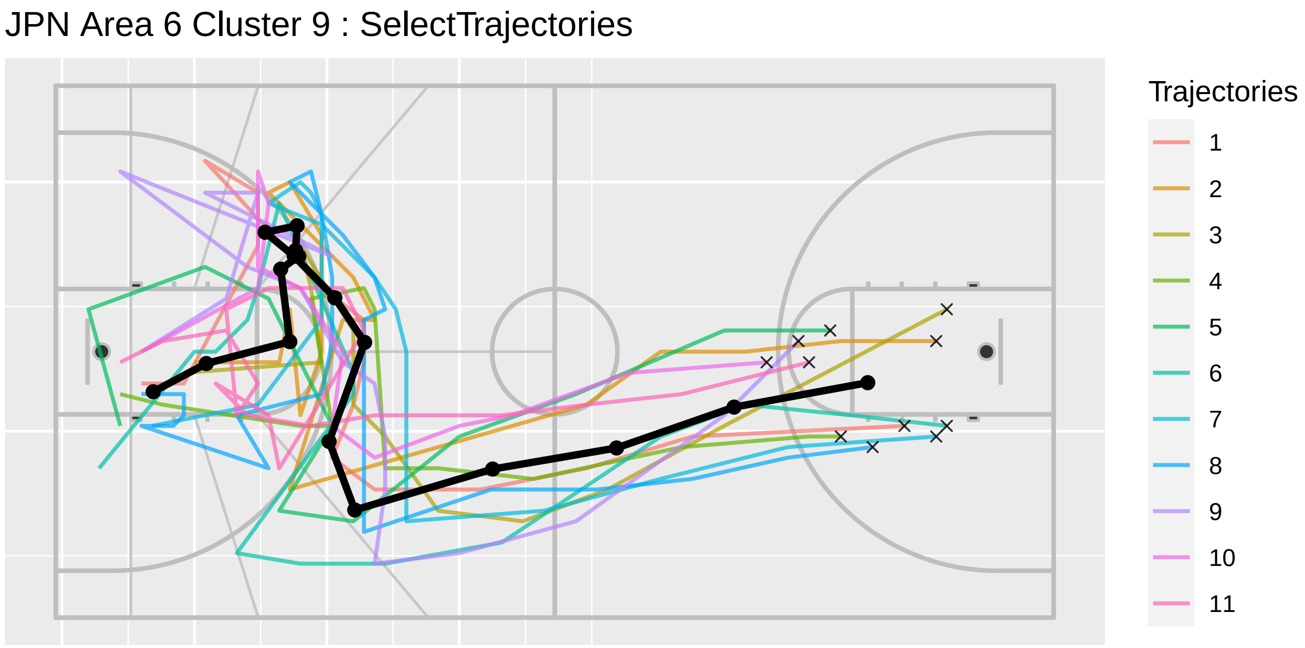

JPN Area 6 Cluster 9 : SelectTrajectories
Trajectories
1
2
3
4
5
6
7
8
9
10
11

JPN Area 6 Cluster 10 : SelectTrajectories

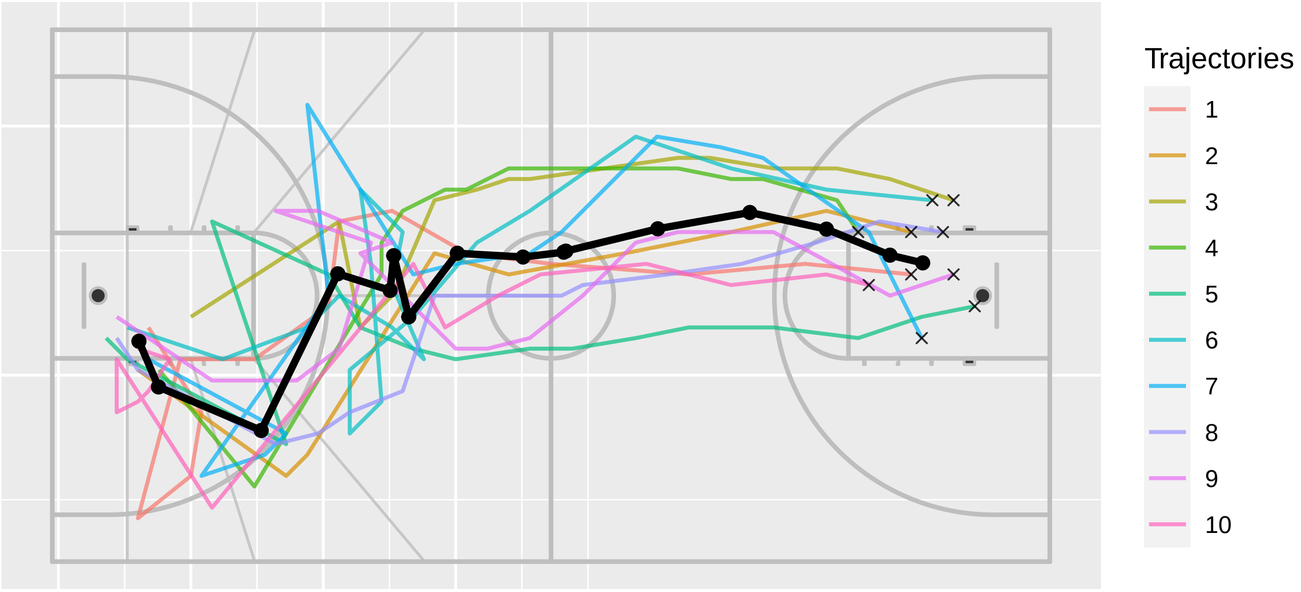

JPN Area 6 Cluster 11 : SelectTrajectories

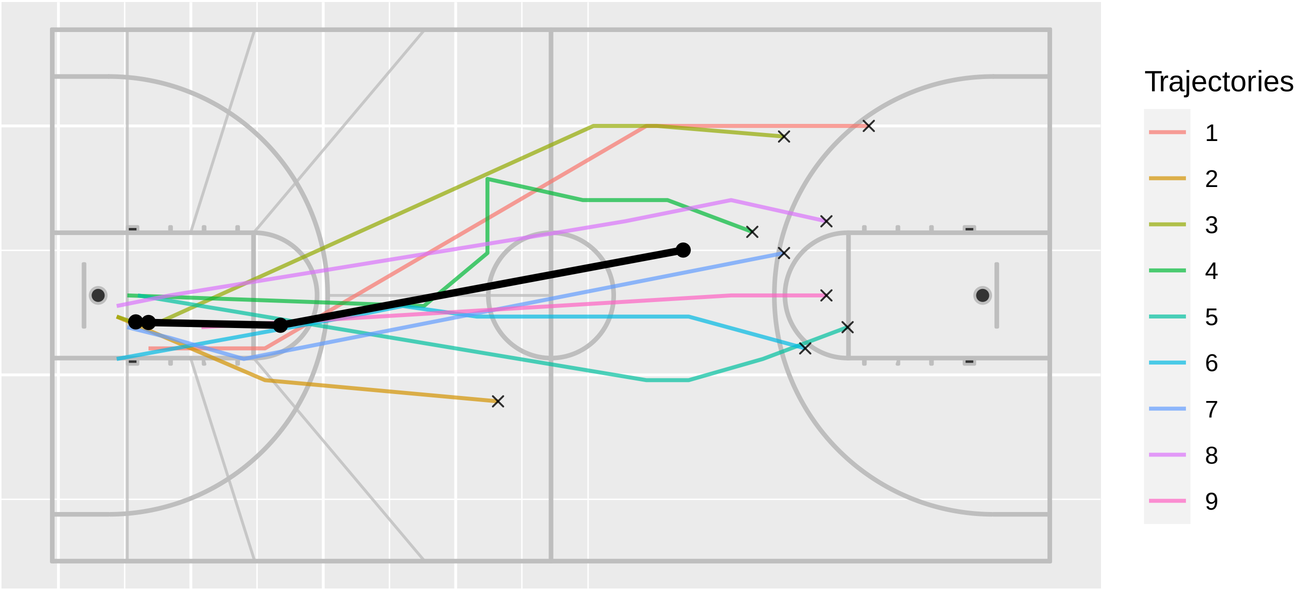

JPN Area 6 Cluster 12 : SelectTrajectories

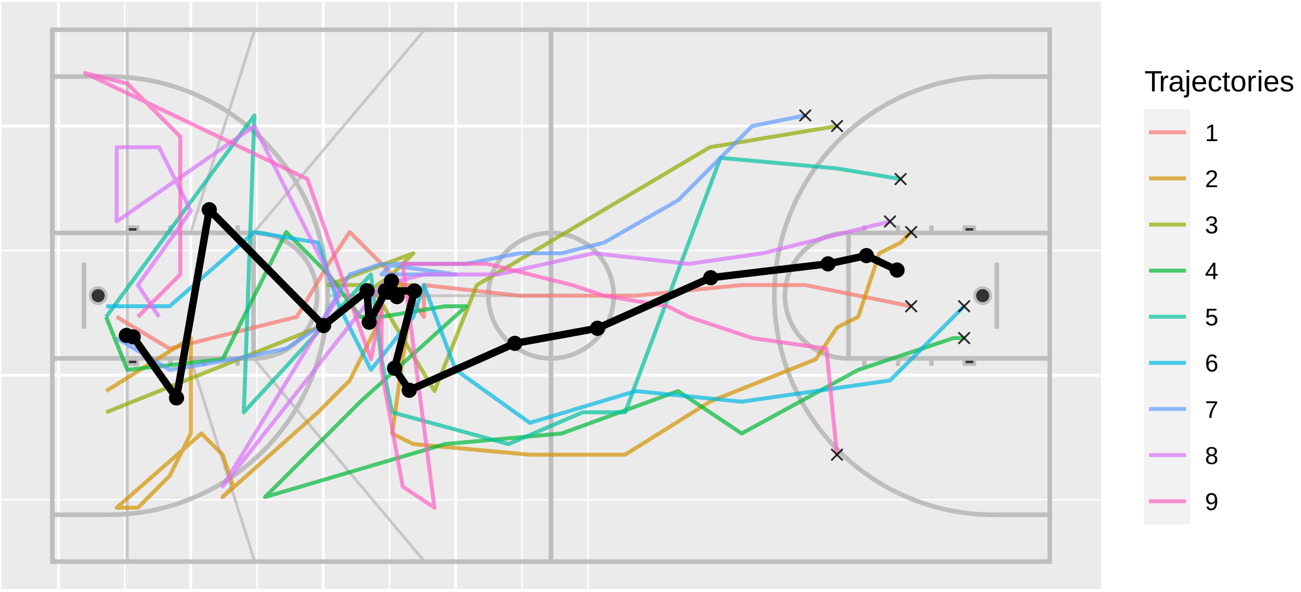

JPN Area 6 Cluster 13 : SelectTrajectories

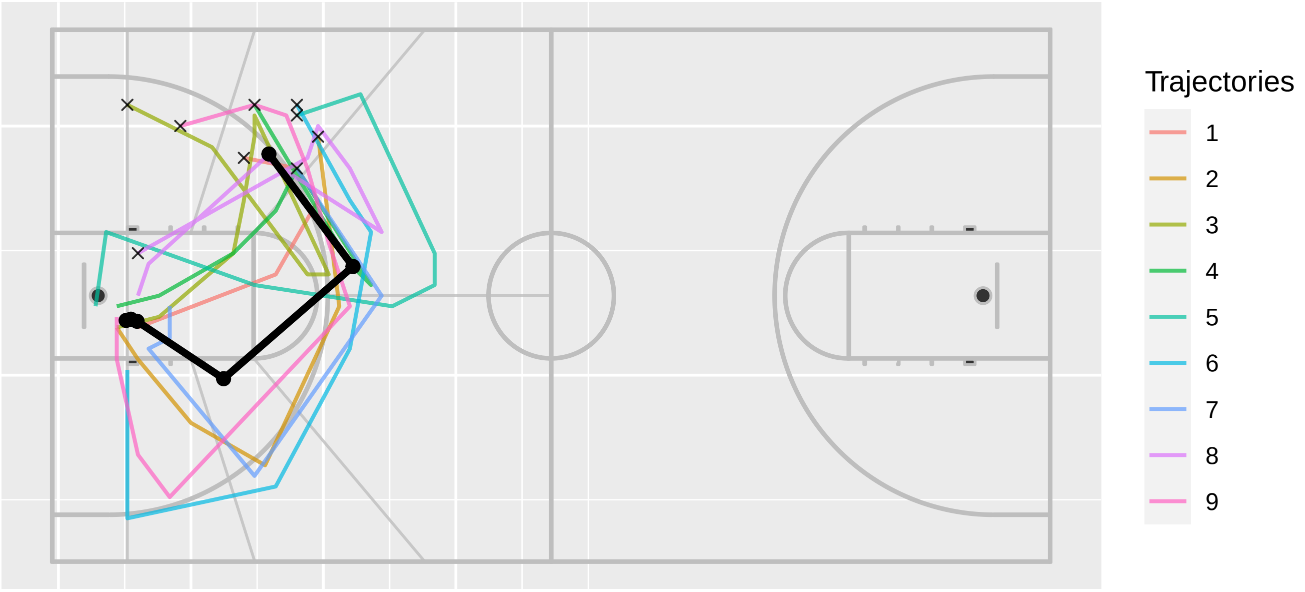

JPN Area 6 Cluster 14 : SelectTrajectories

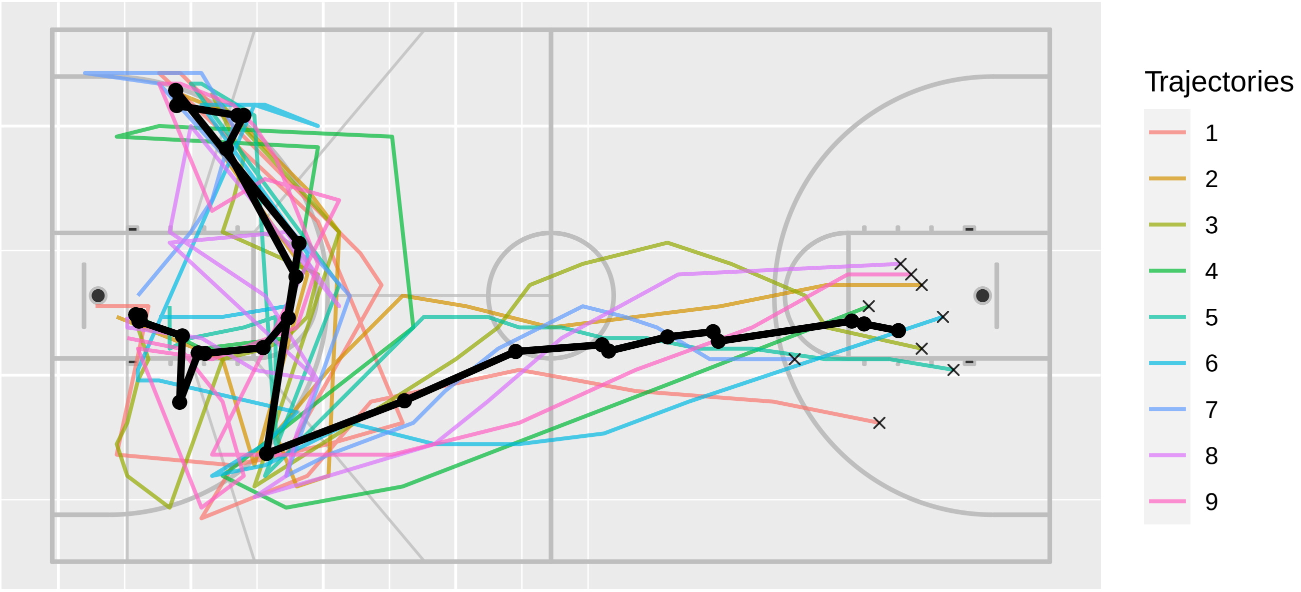

JPN Area 6 Cluster 15 : SelectTrajectories

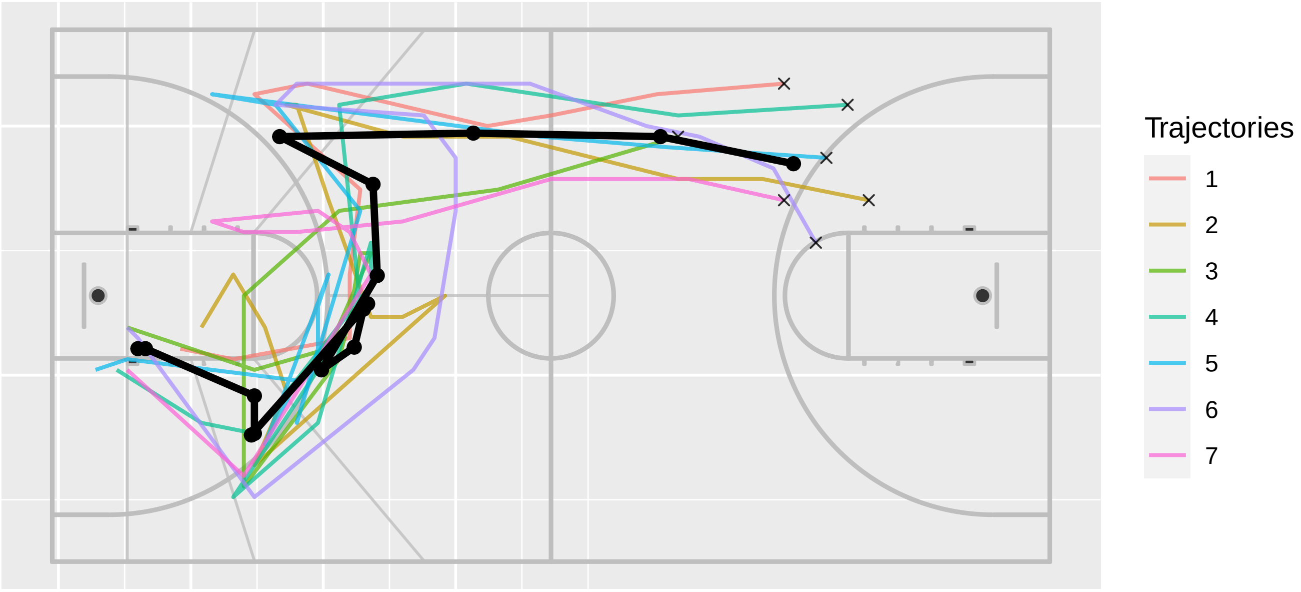

JPN Area 6 Cluster 16 : SelectTrajectories

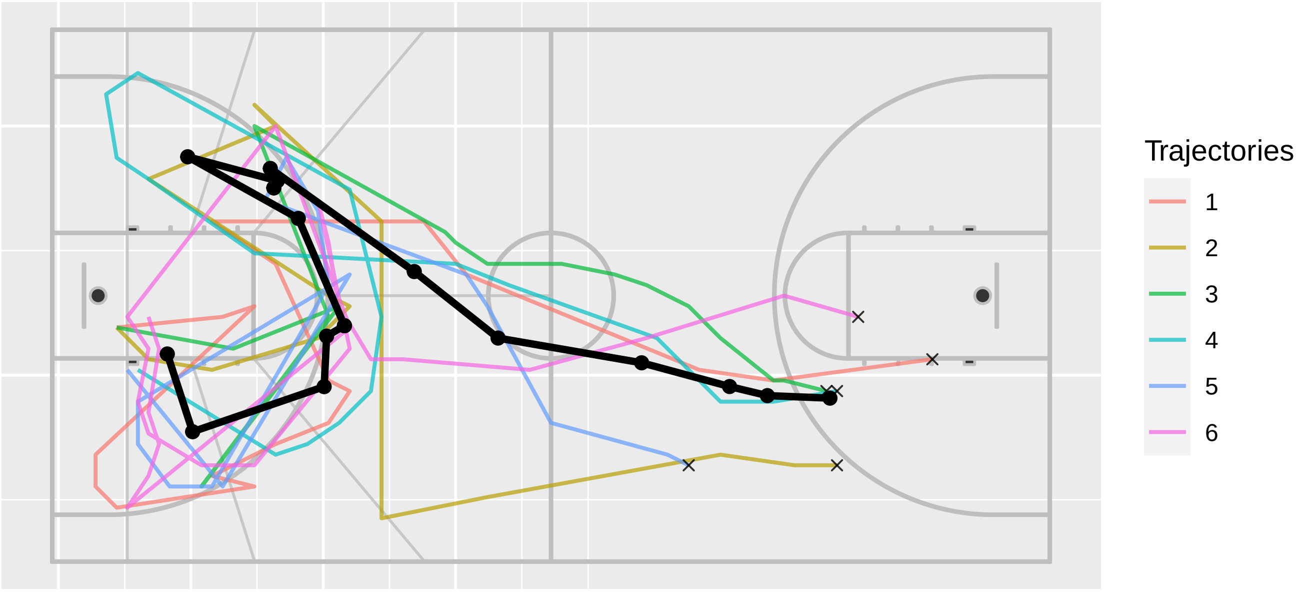

JPN Area 6 Cluster 17 : SelectTrajectories

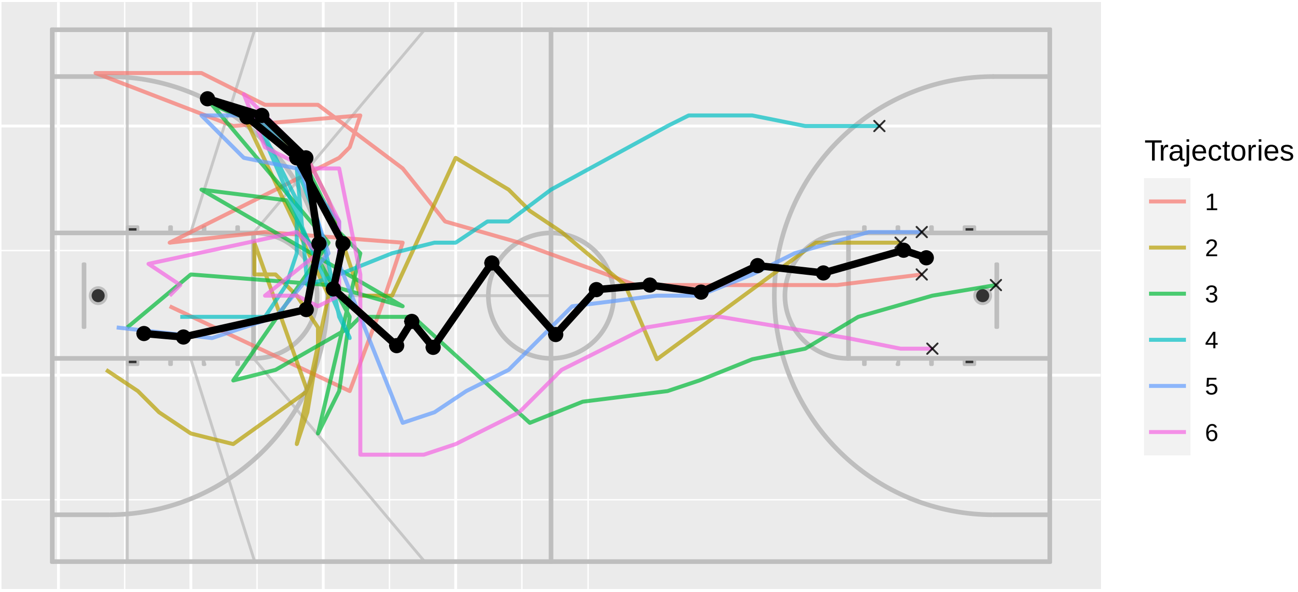

JPN Area 6 Cluster 18 : SelectTrajectories

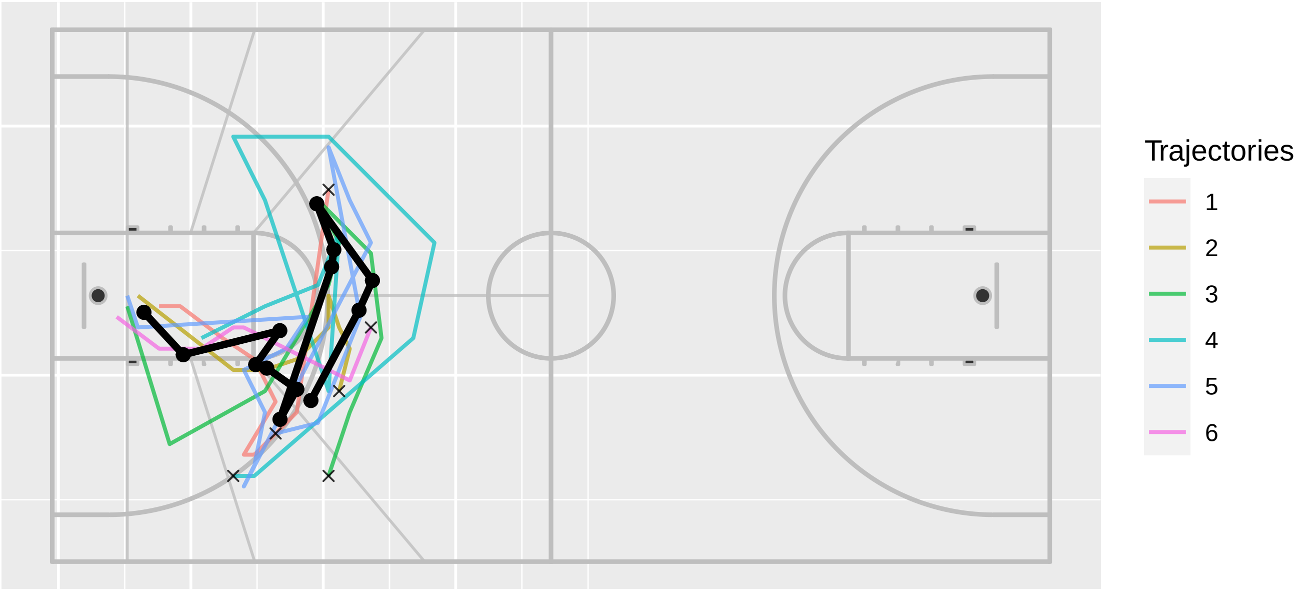

JPN Area 6 Cluster 19 : SelectTrajectories

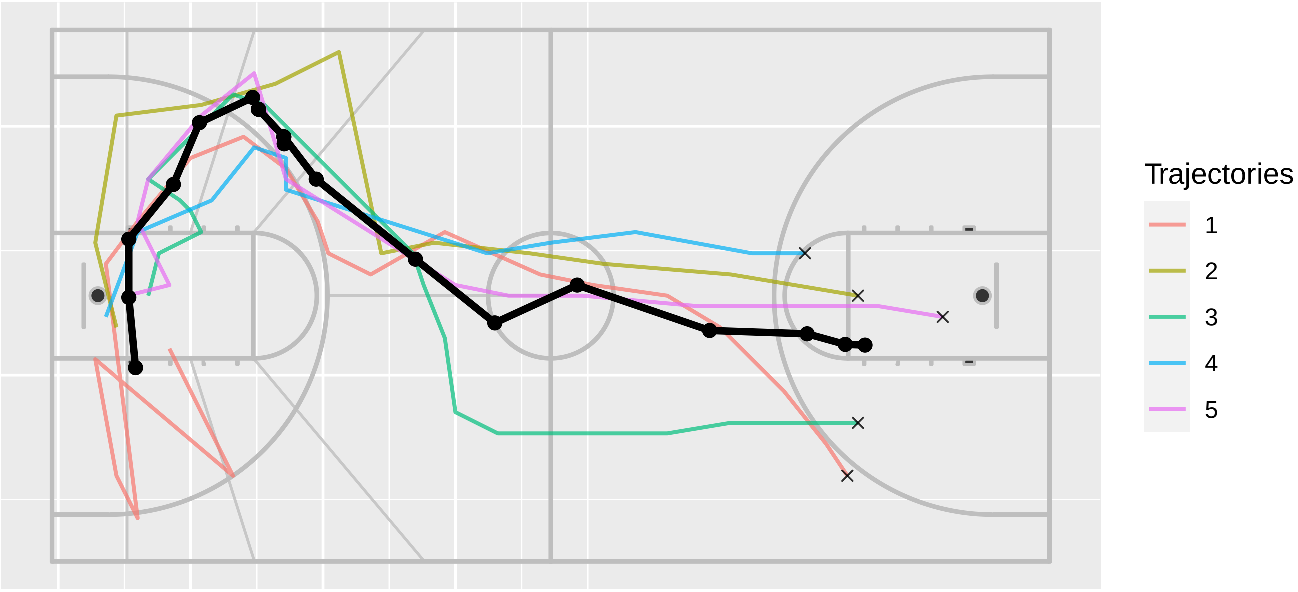

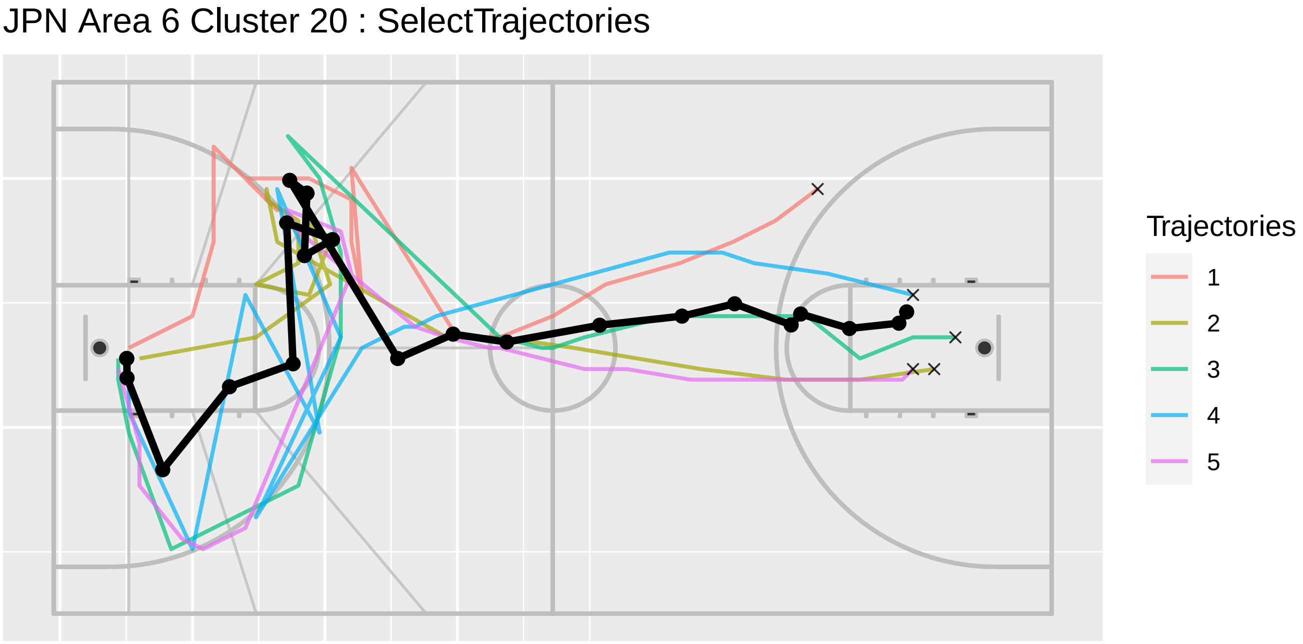
JPN Area 6 Cluster 20 : SelectTrajectories
Trajectories
1
2
3
4
5

JPN Area 6 Cluster 21 : SelectTrajectories

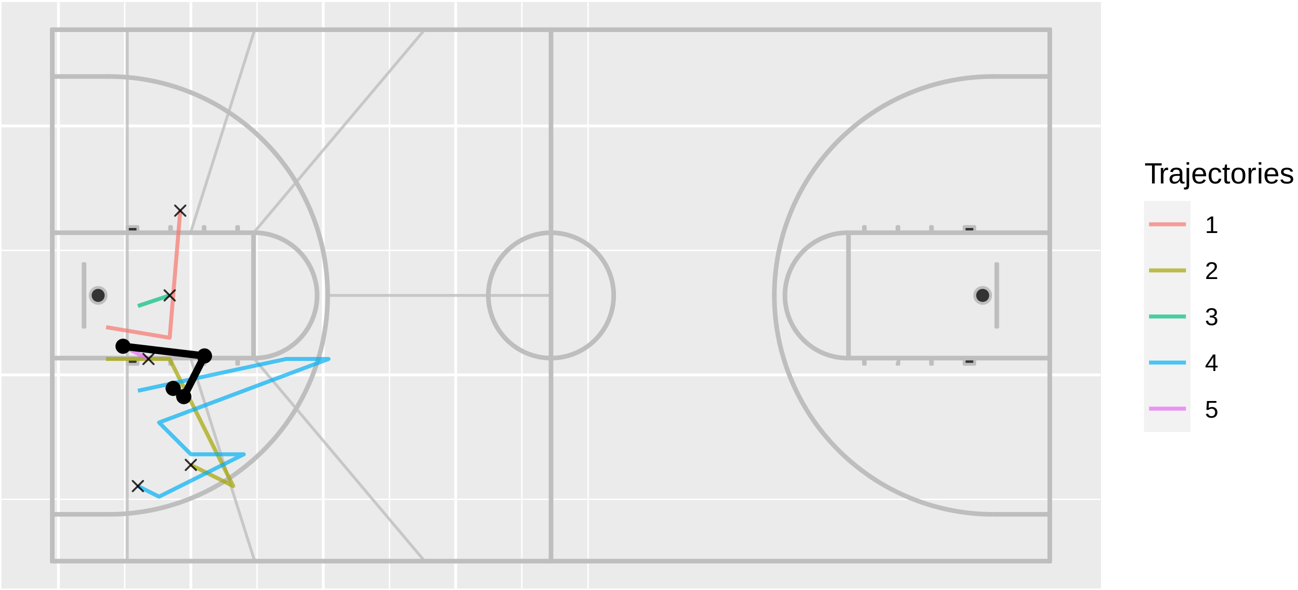

JPN Area 6 Cluster 22 : SelectTrajectories

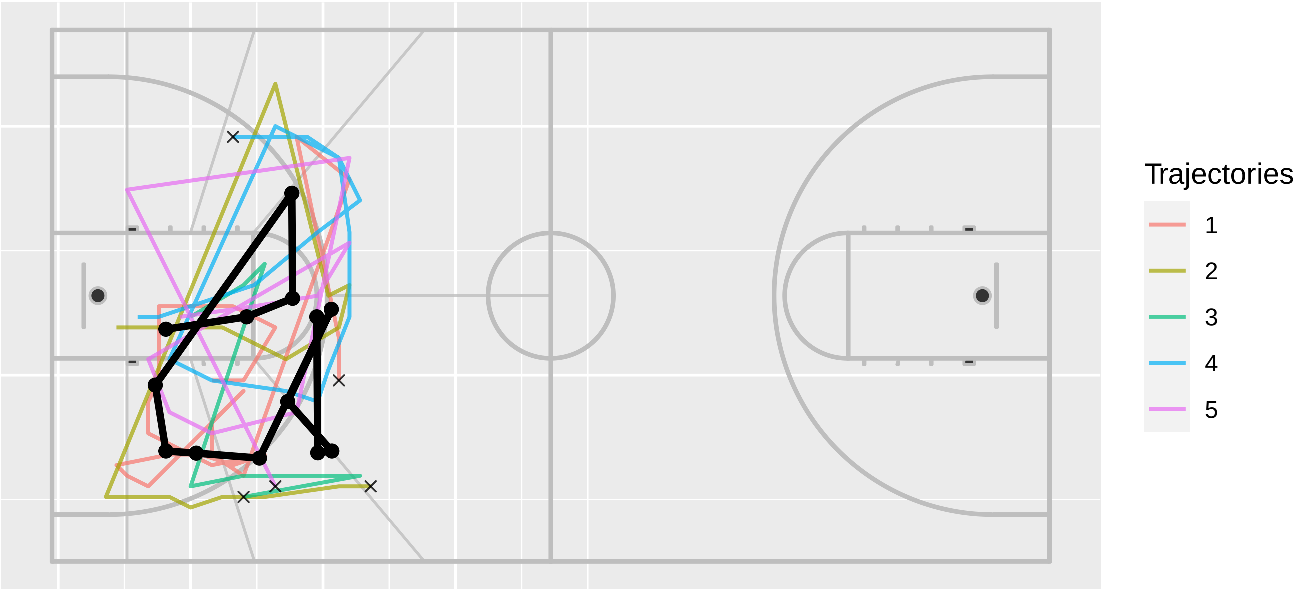

JPN Area 6 Cluster 23 : SelectTrajectories

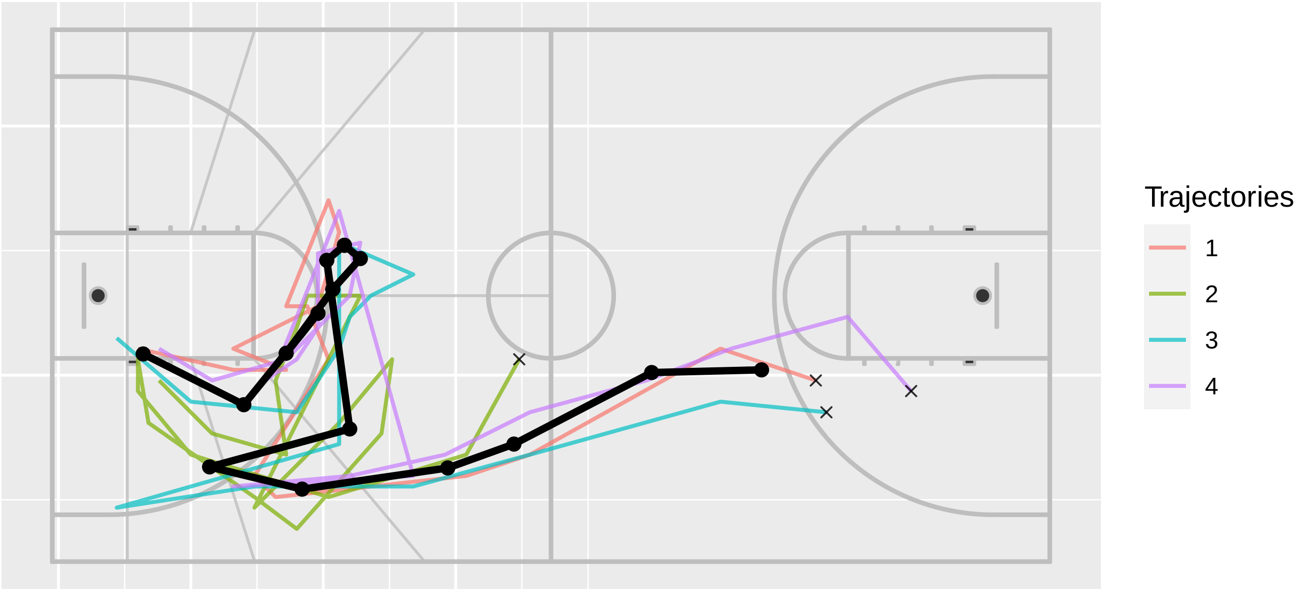

JPN Area 6 Cluster 24 : SelectTrajectories

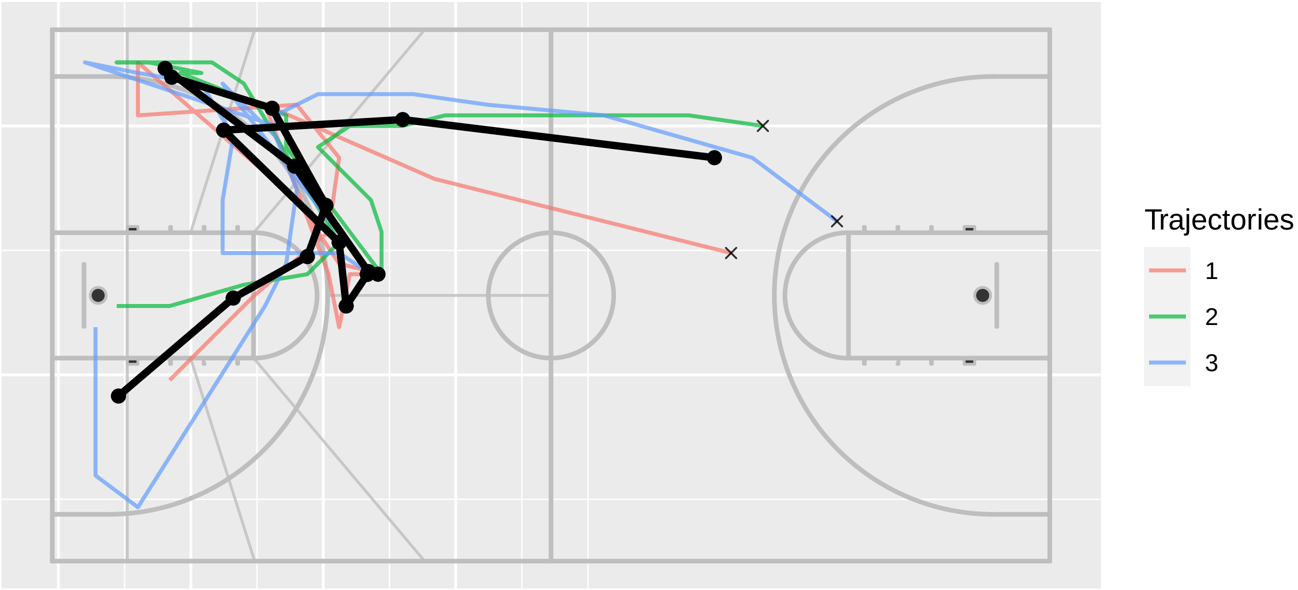

JPN Area 6 Cluster 25 : SelectTrajectories

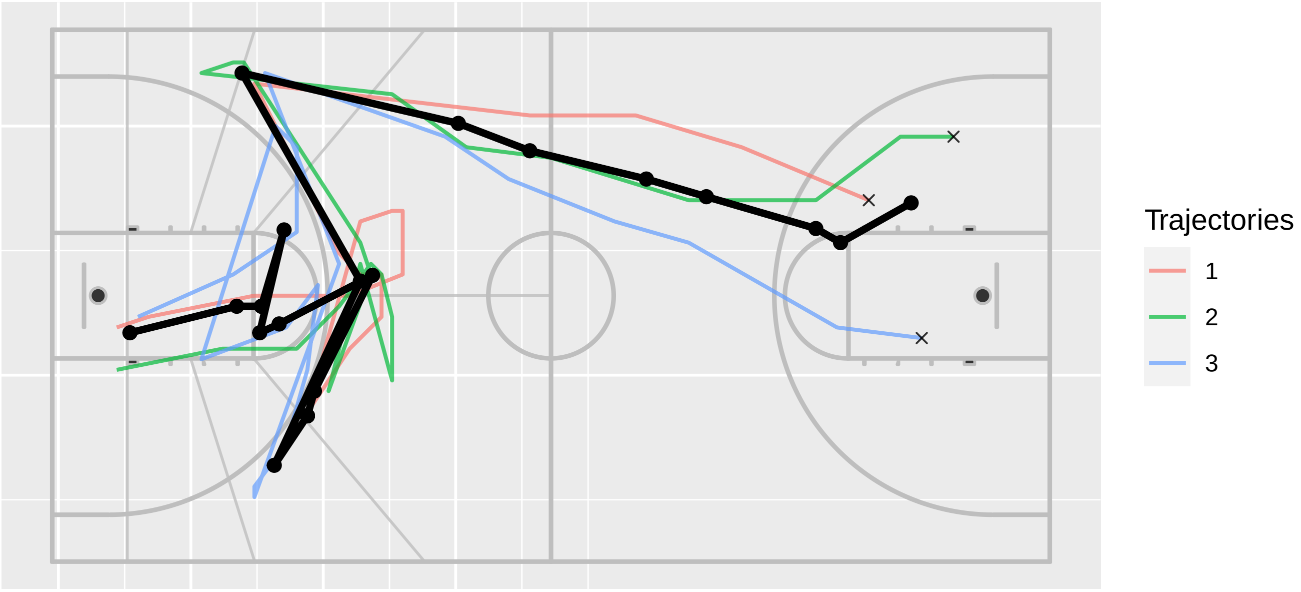

JPN Area 6 Cluster 26 : SelectTrajectories

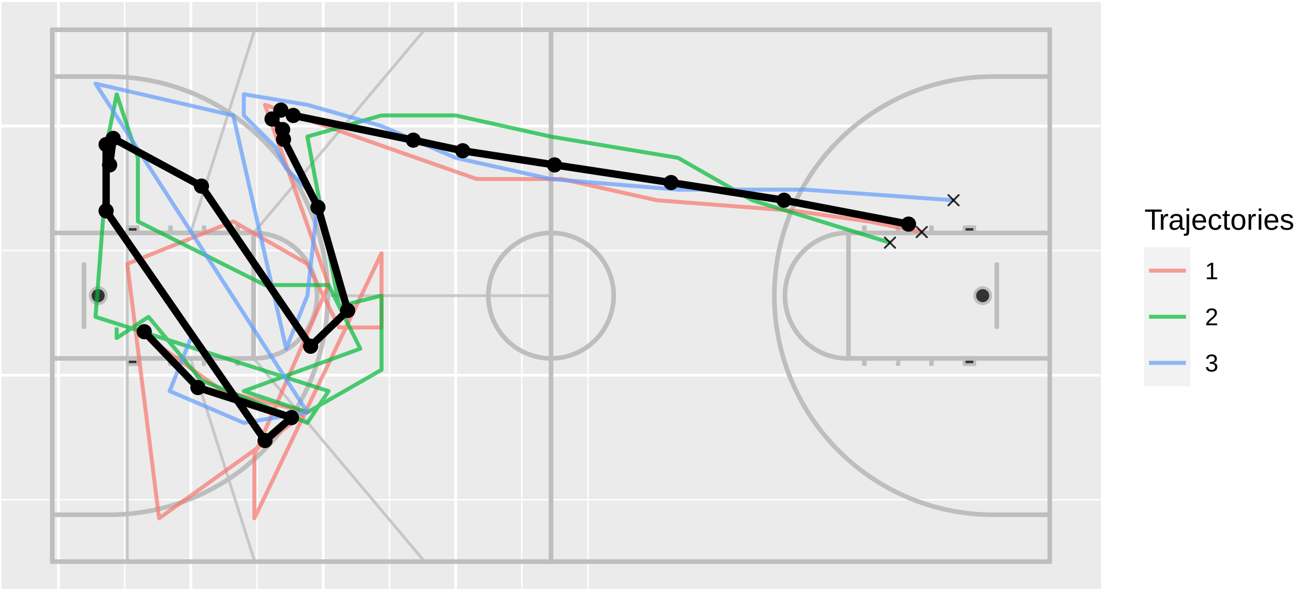

JPN Area 6 Cluster 27 : SelectTrajectories

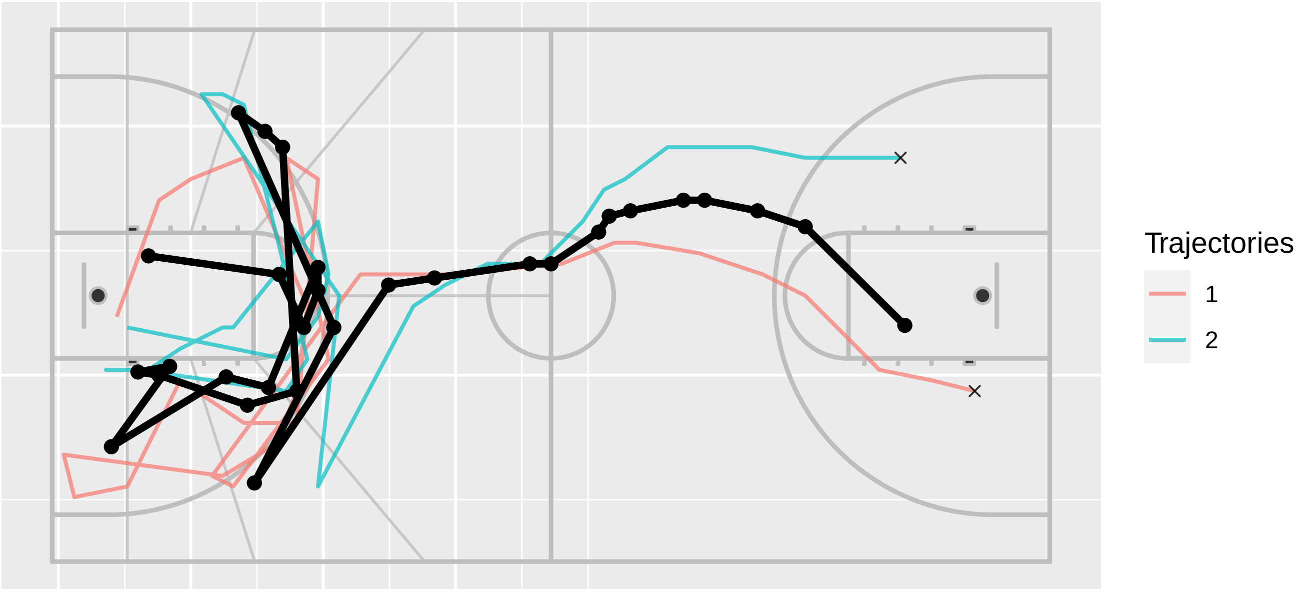

JPN Area 6 Cluster 28 : SelectTrajectories

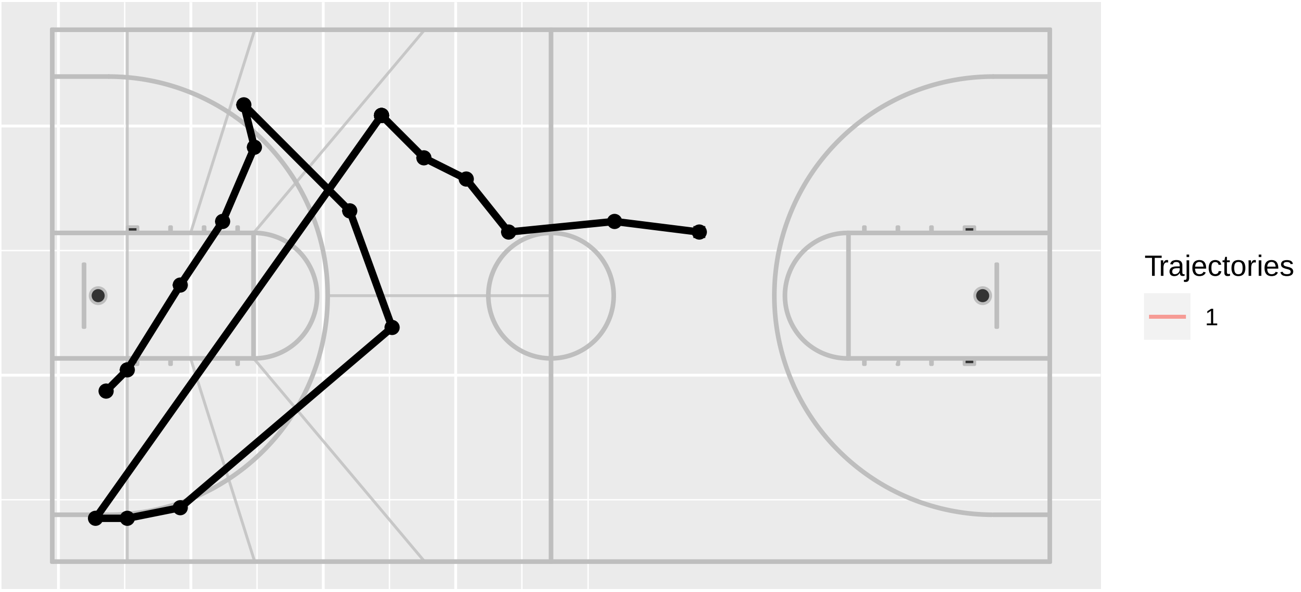

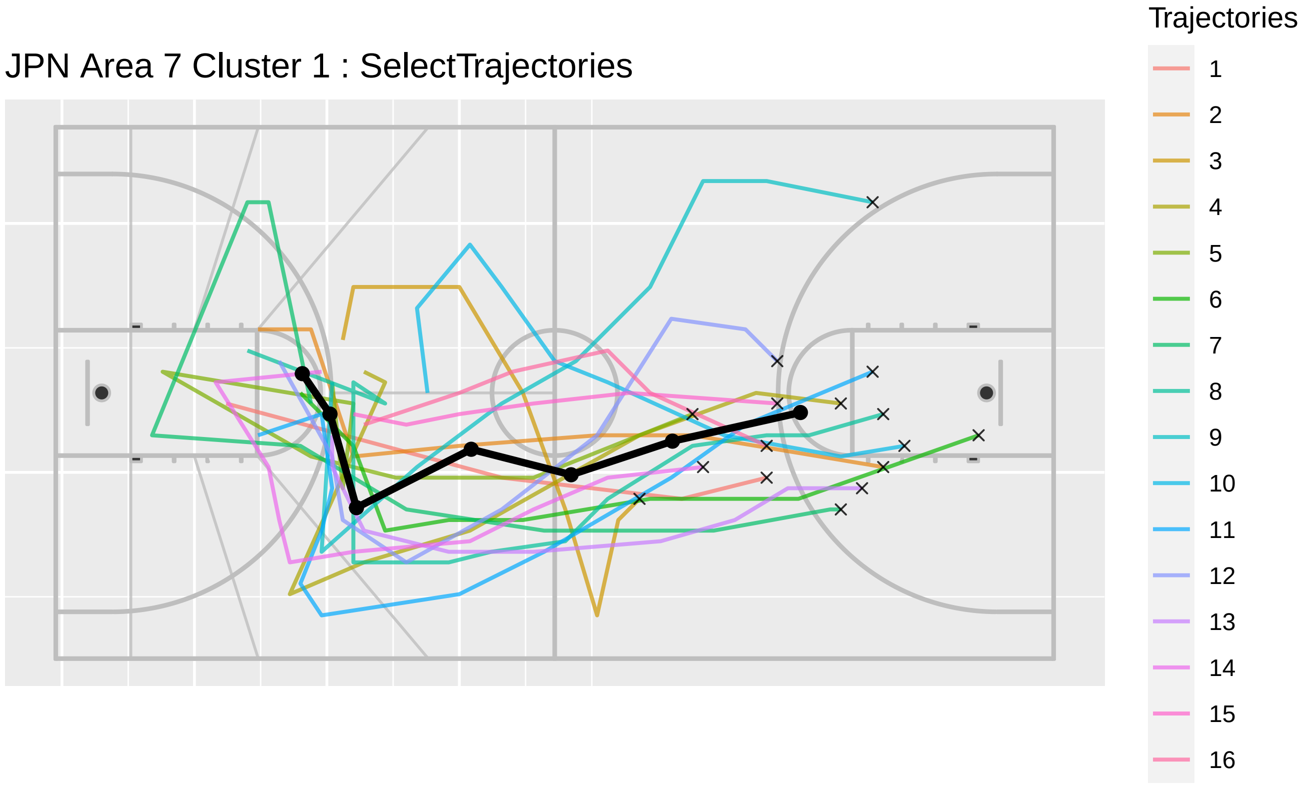
JPN Area 7 Cluster 1 : SelectTrajectories
Trajectories
1
2
3
4
5
6
7
8
9
10
11
12
13
14
15
16

JPN Area 7 Cluster 2 : SelectTrajectories

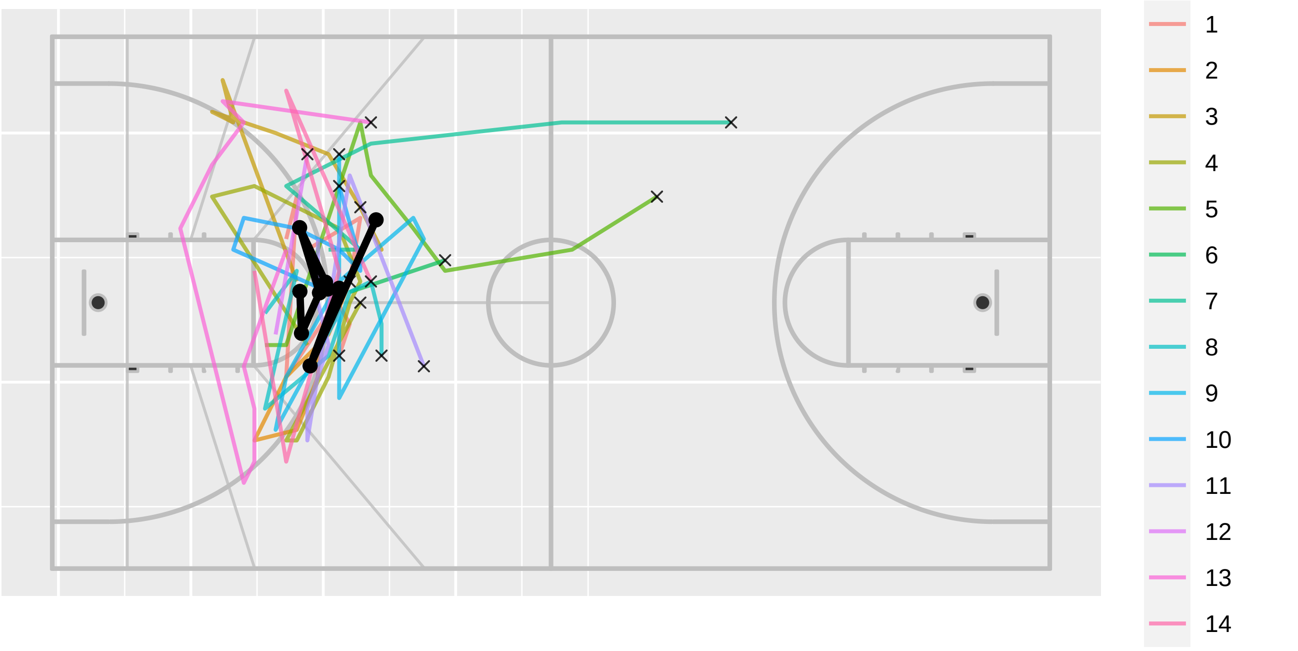

JPN Area 7 Cluster 3 : SelectTrajectories

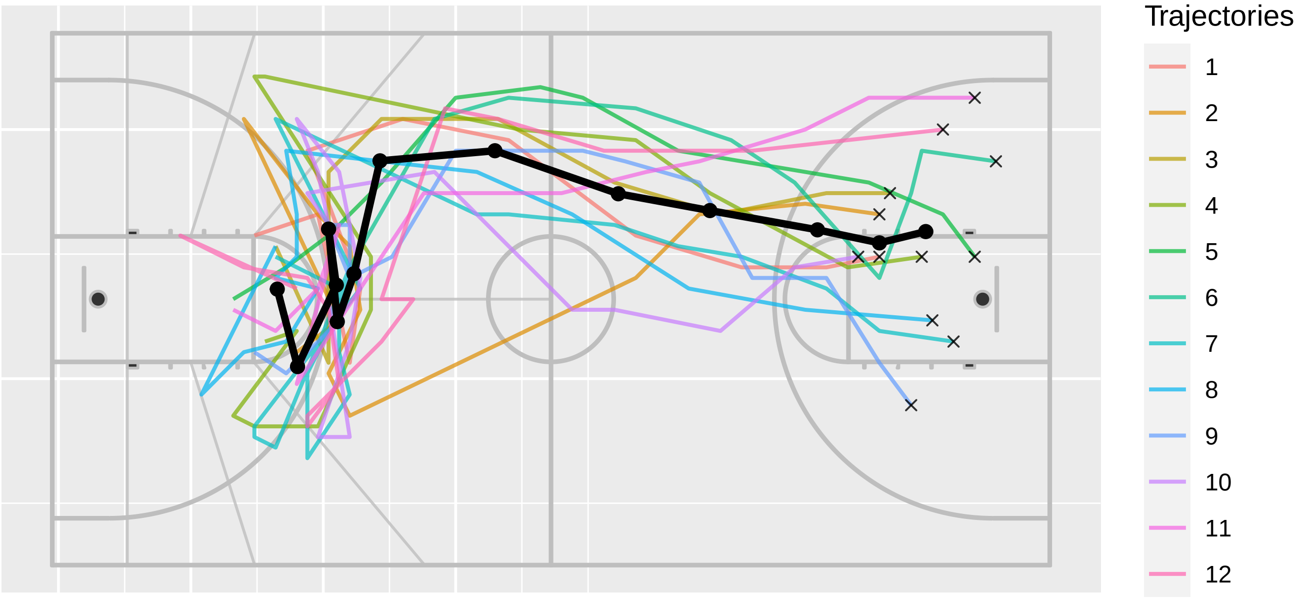

JPN Area 7 Cluster 4 : SelectTrajectories

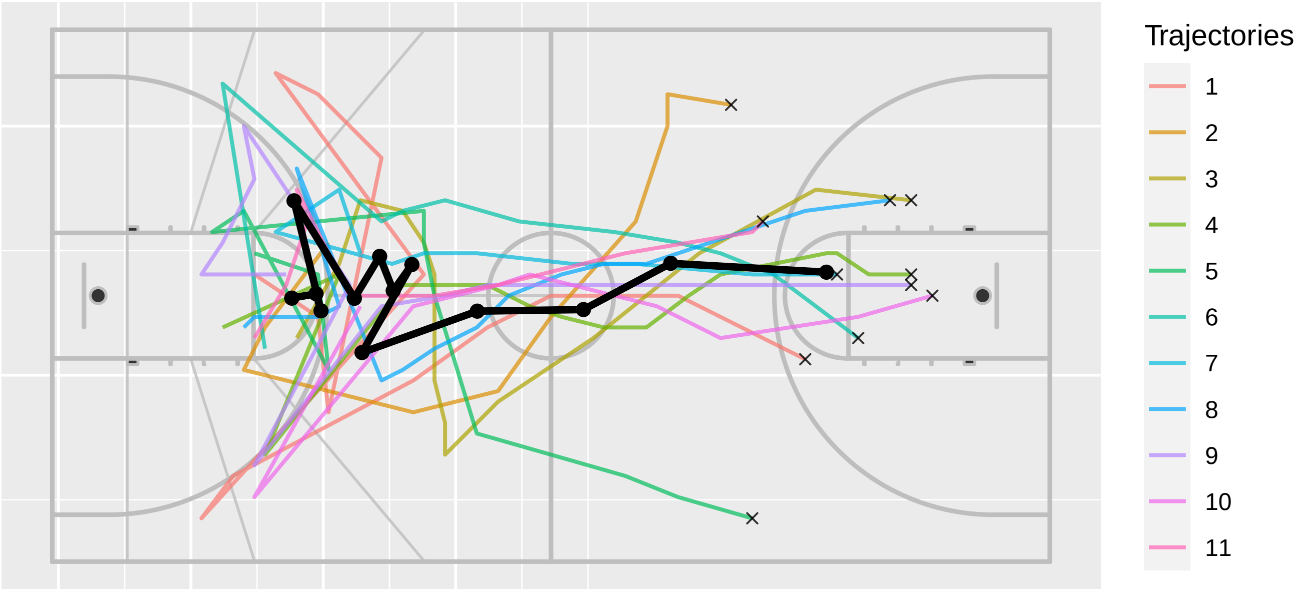

JPN Area 7 Cluster 5 : SelectTrajectories

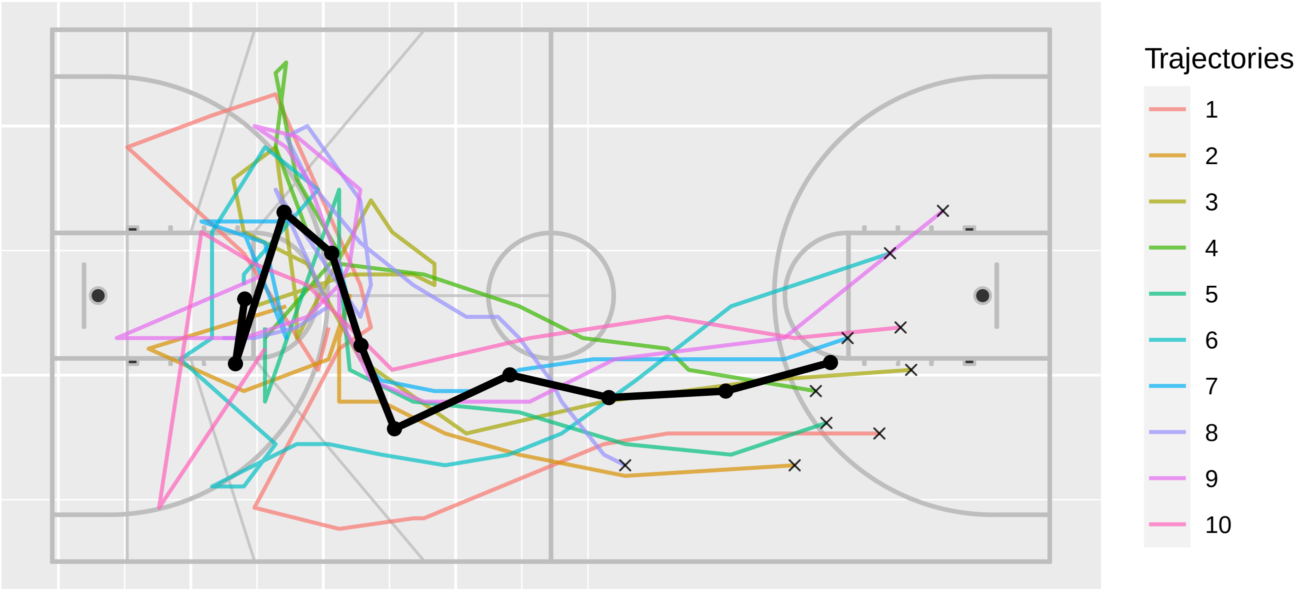

JPN Area 7 Cluster 6 : SelectTrajectories

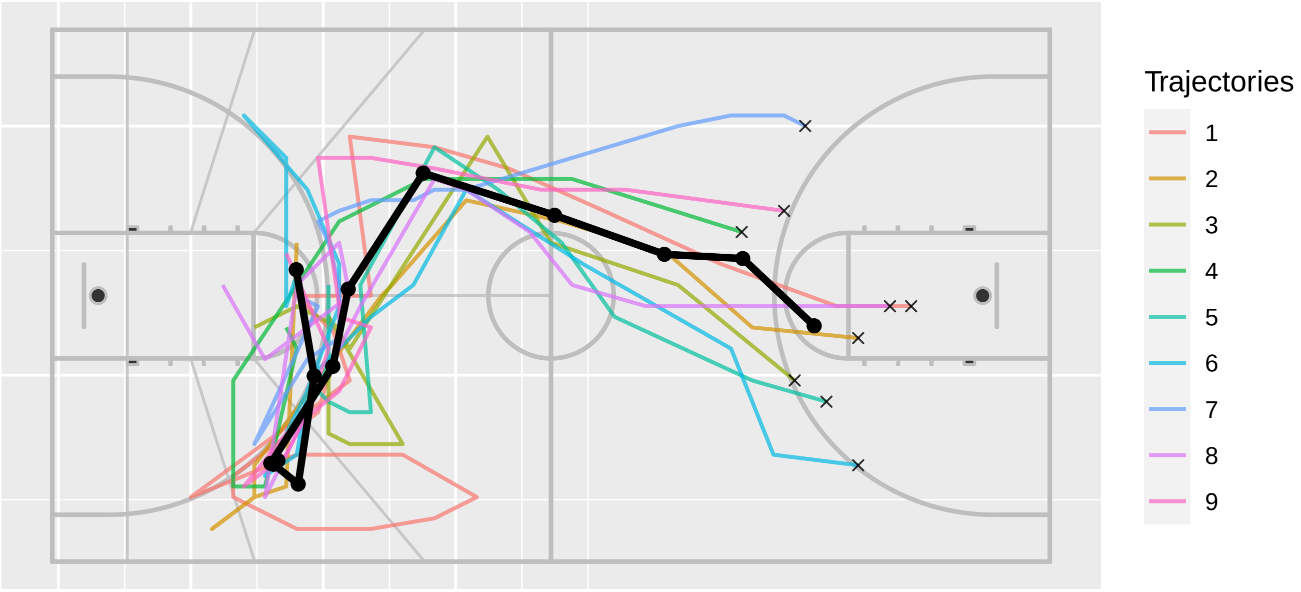

JPN Area 7 Cluster 7 : SelectTrajectories

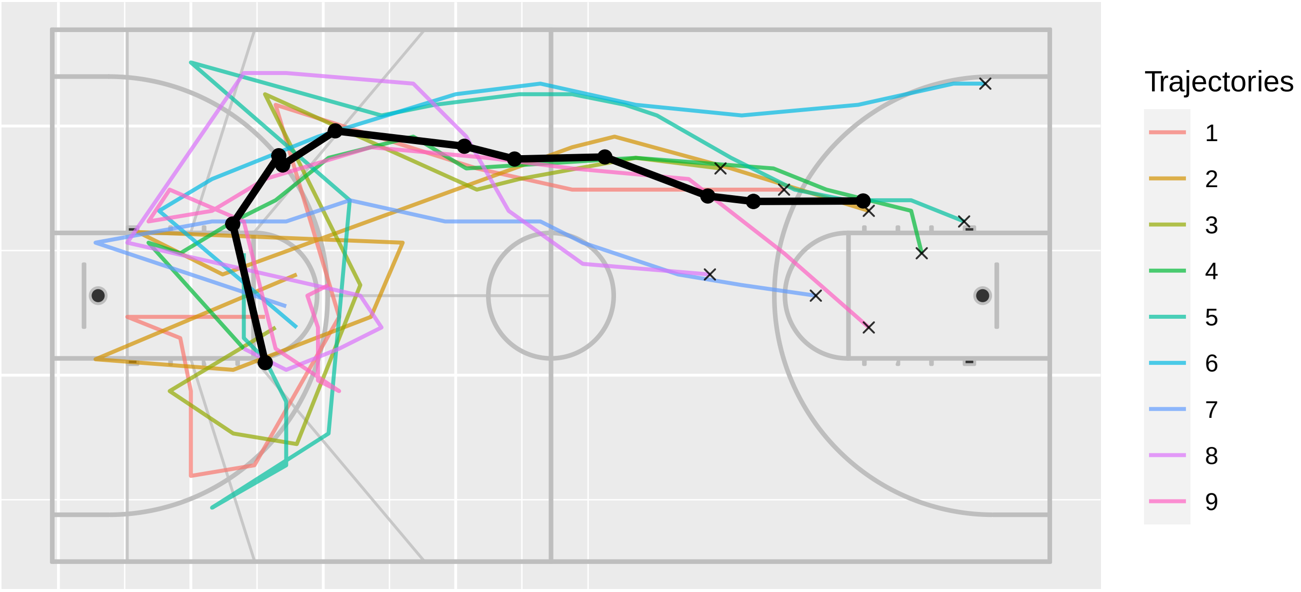

JPN Area 7 Cluster 8 : SelectTrajectories

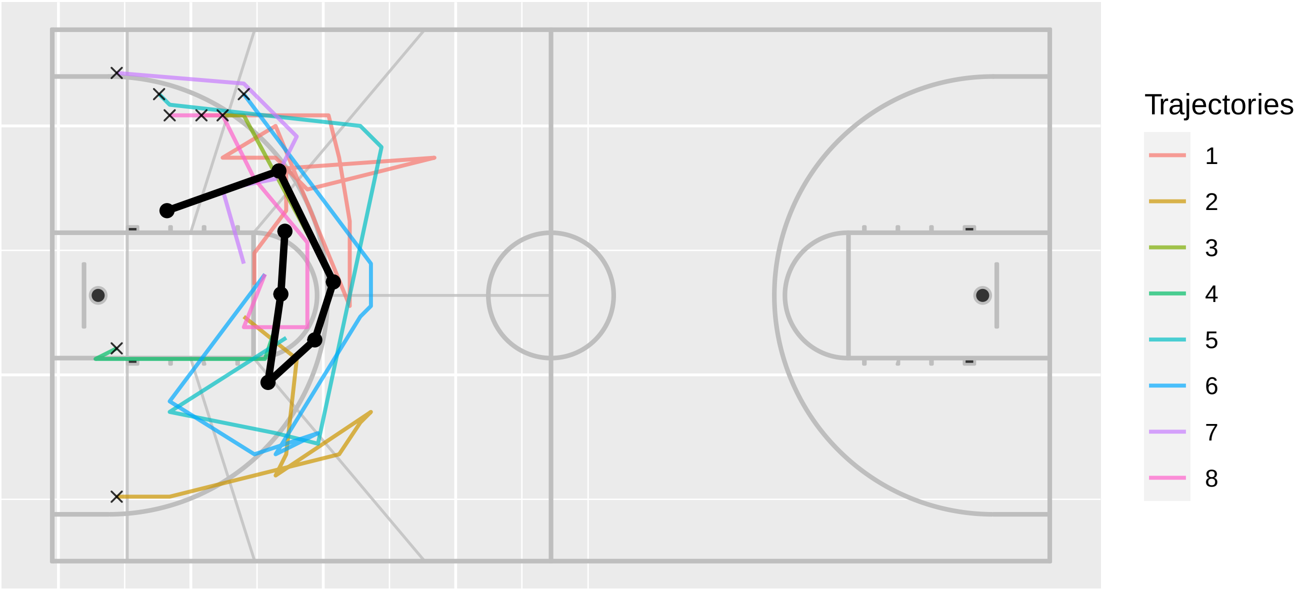

JPN Area 7 Cluster 9 : SelectTrajectories

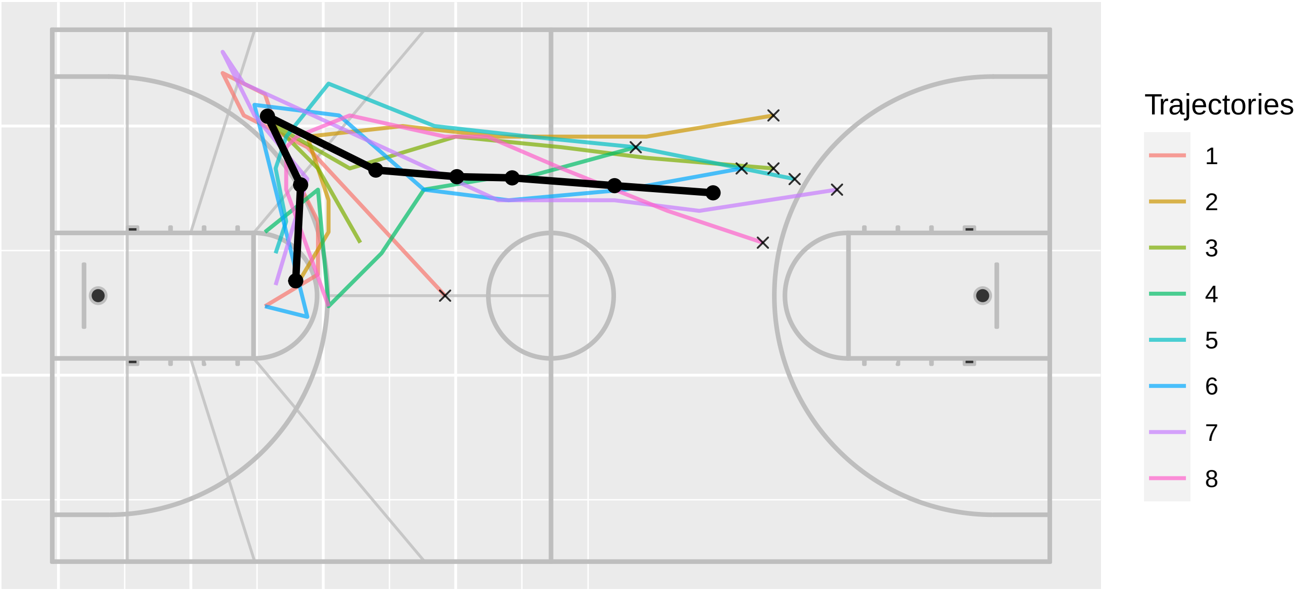

JPN Area 7 Cluster 10 : SelectTrajectories

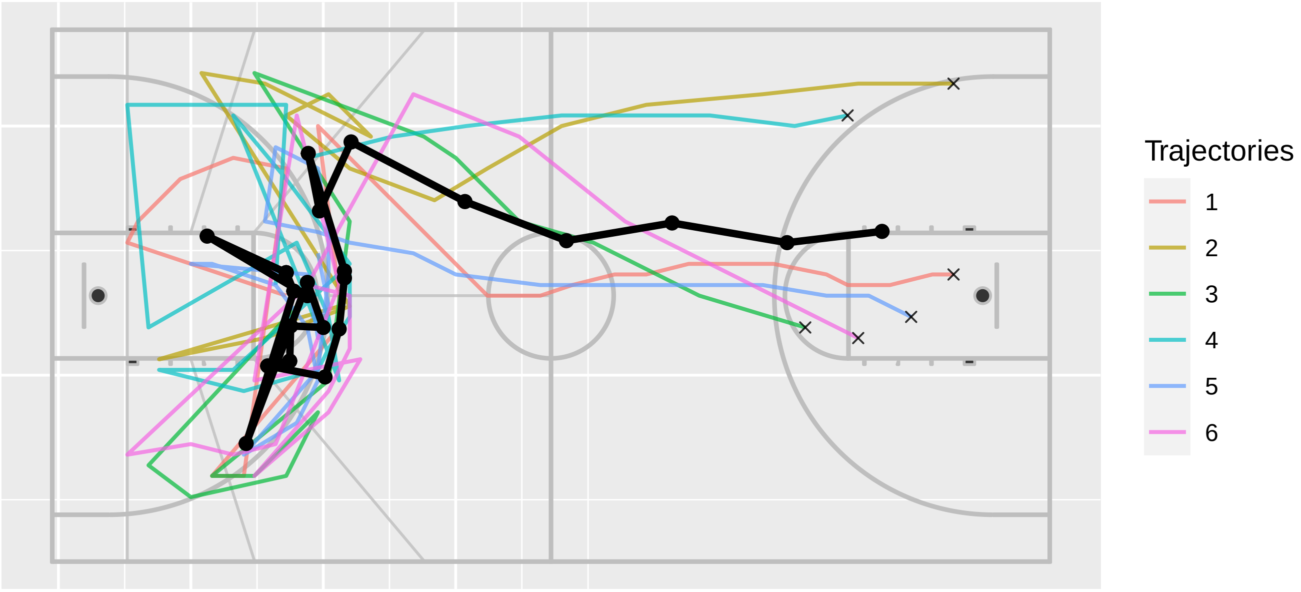

JPN Area 7 Cluster 11 : SelectTrajectories

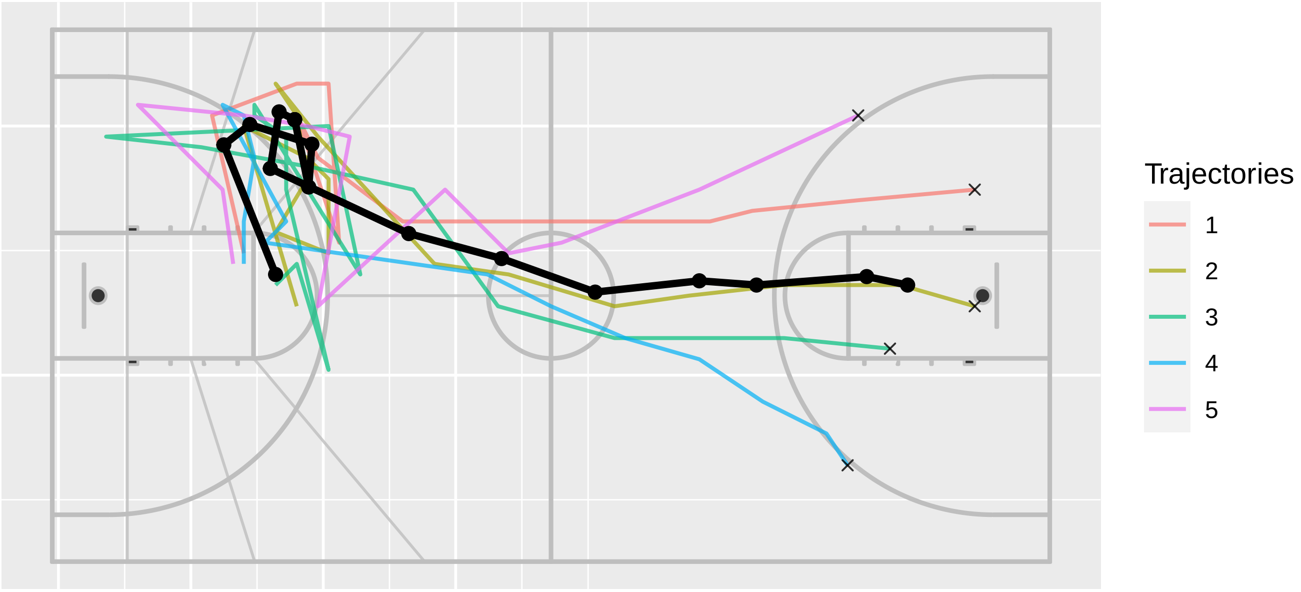

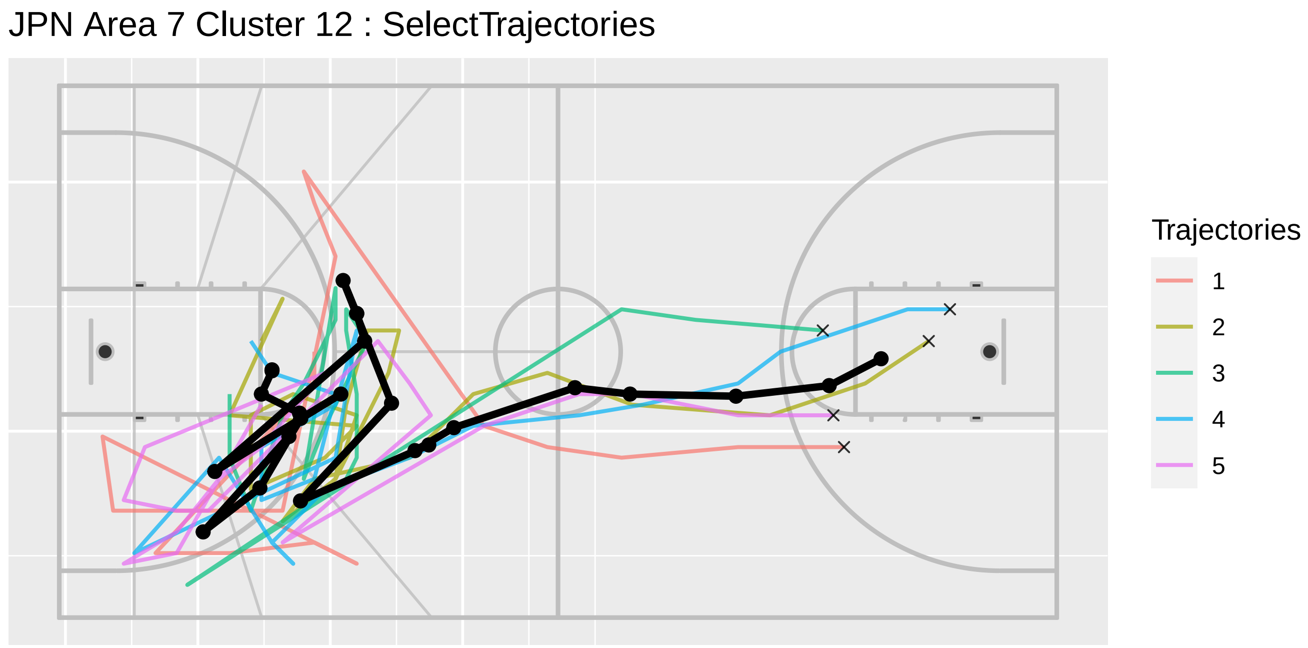
JPN Area 7 Cluster 12 : SelectTrajectories
Trajectories
1
2
3
4
5

JPN Area 7 Cluster 13 : SelectTrajectories

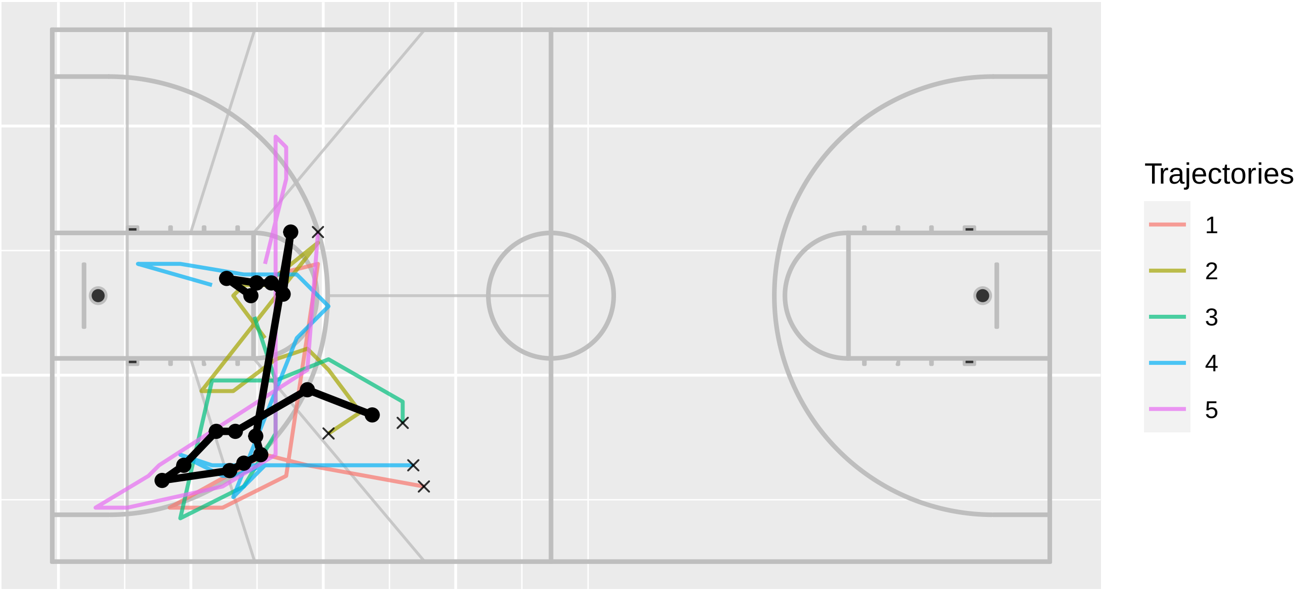

JPN Area 7 Cluster 14 : SelectTrajectories

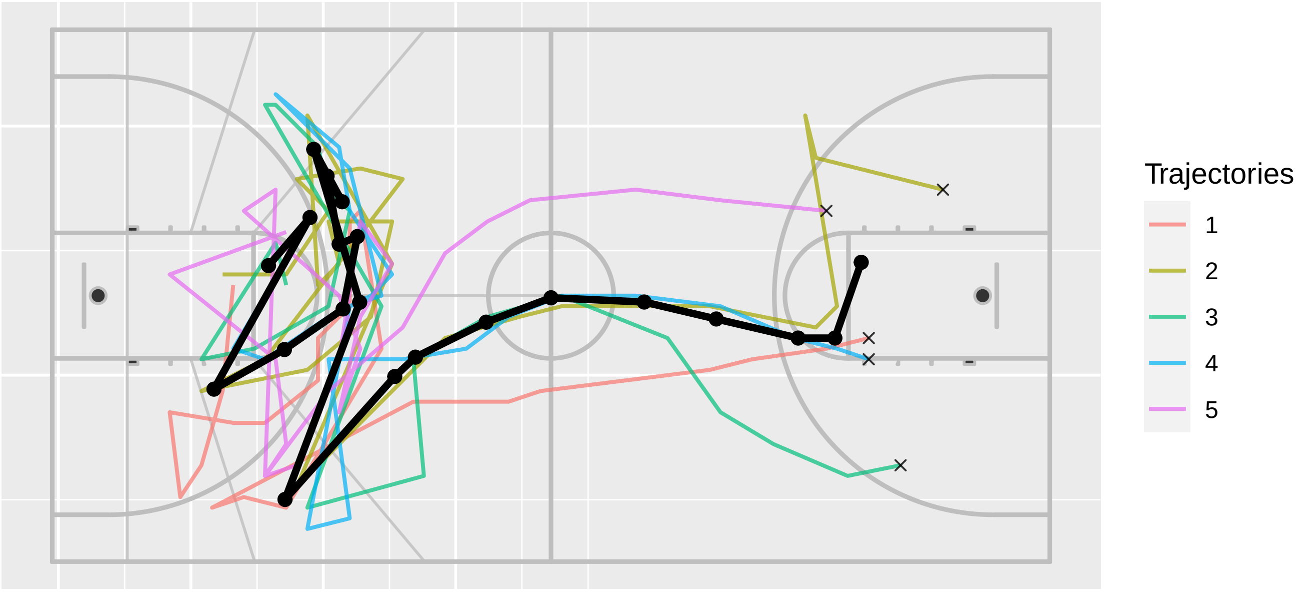

JPN Area 7 Cluster 15 : SelectTrajectories

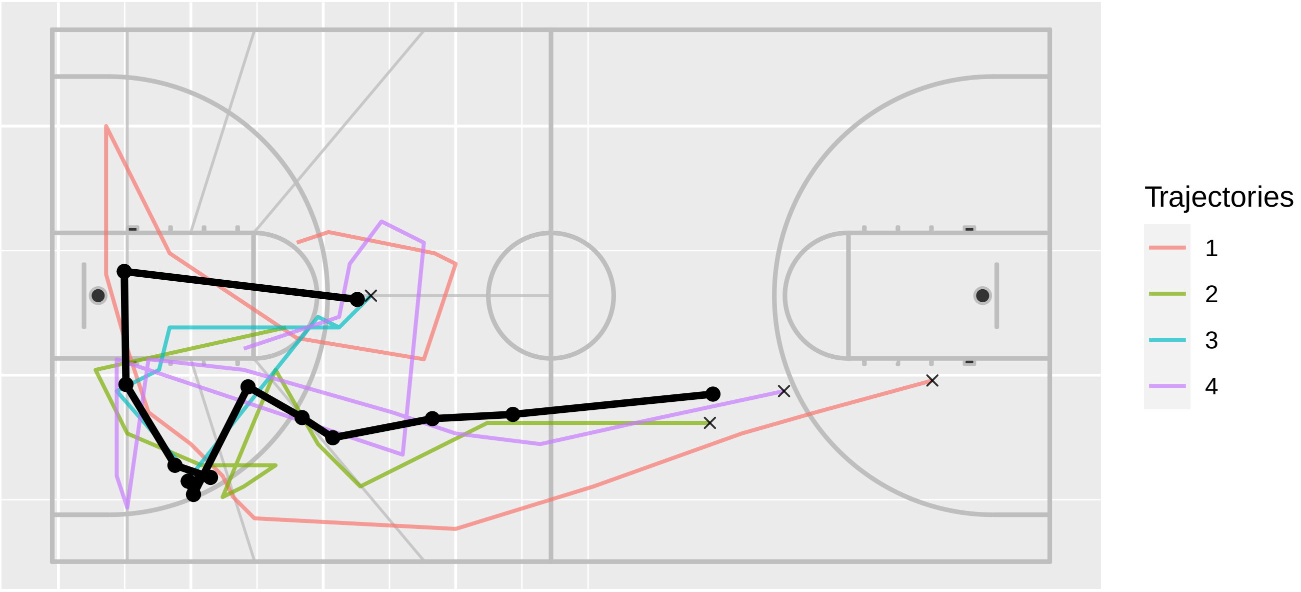

JPN Area 7 Cluster 16 : SelectTrajectories

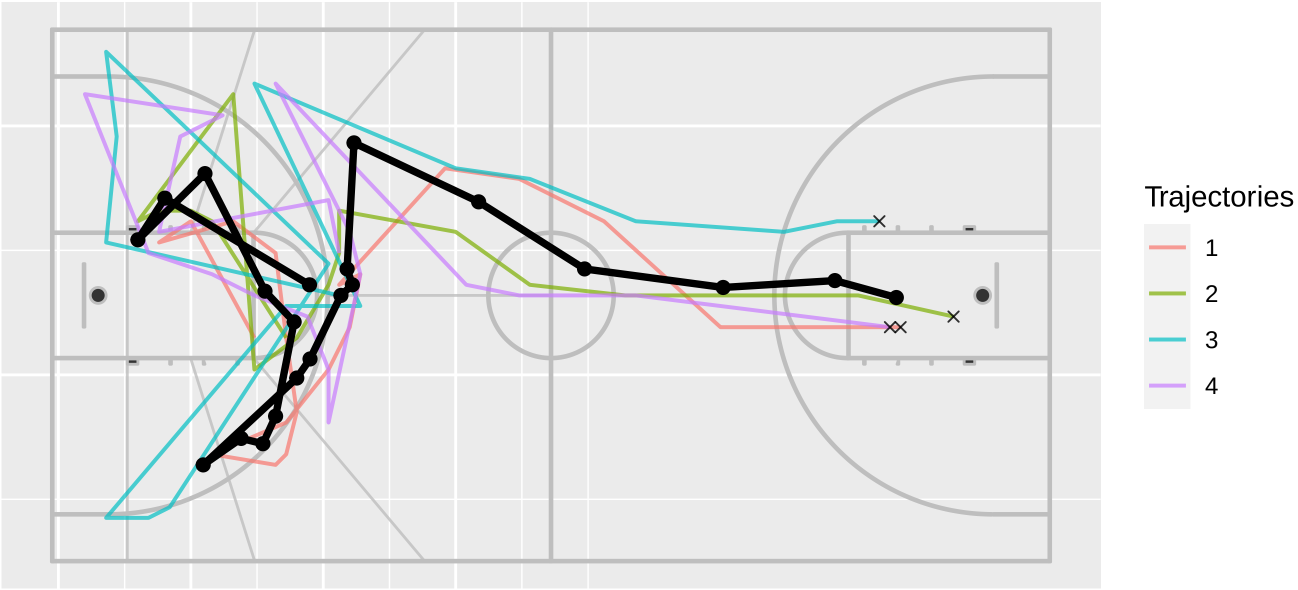

JPN Area 7 Cluster 17 : SelectTrajectories

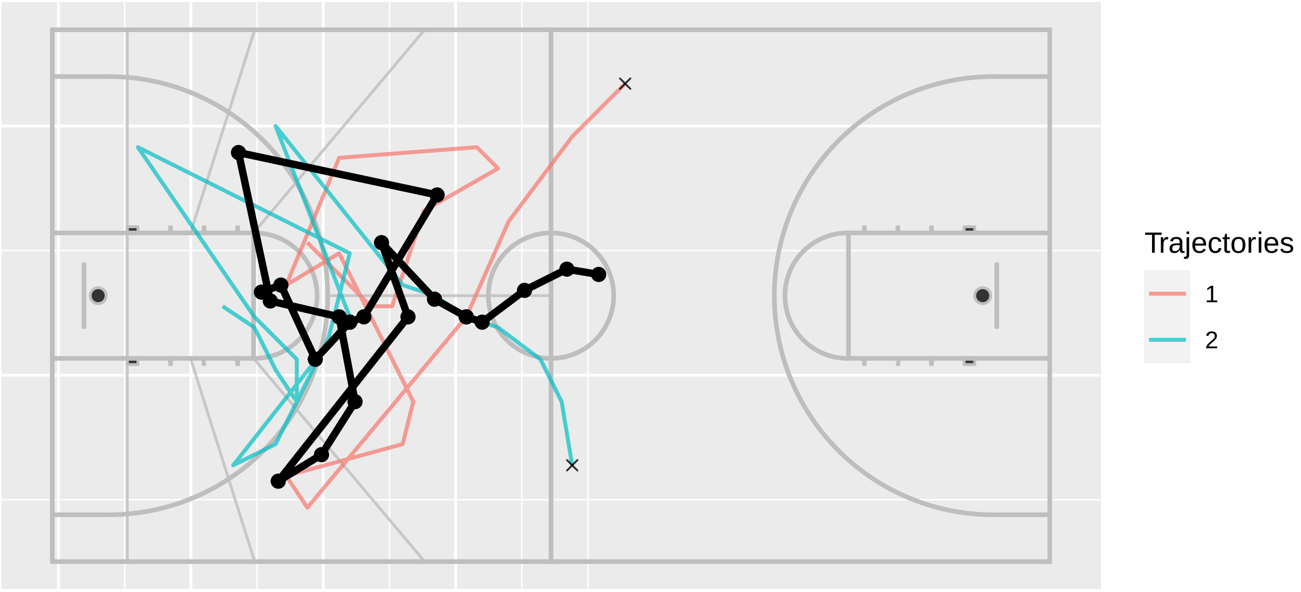

Supplement: S7 Appendix — (PDF) [file pone.0272848.s007.pdf]
