# Supplementary material for: Classifying ball trajectories in invasion sports using dynamic time warping: A basketball case study
Source: PLoS One. 2022 Oct 20;17(10):e0272848. doi: 10.1371/journal.pone.0272848 (PMC9584368; doi:10.1371/journal.pone.0272848)

USA Area 1 with cluster c(20, 20, 50, 50, 50, 20, 50) Cluster dendrogram

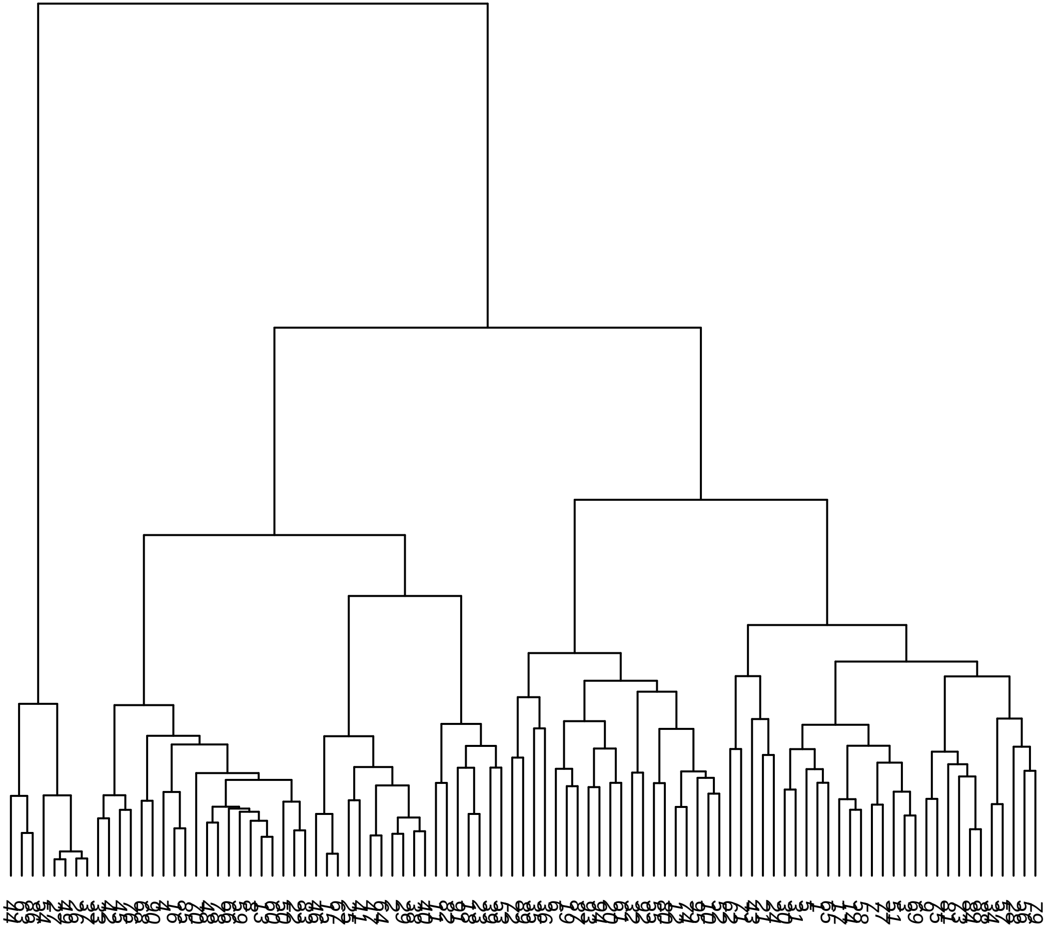

USA Area 1 Unrooted Cluster dendrogram

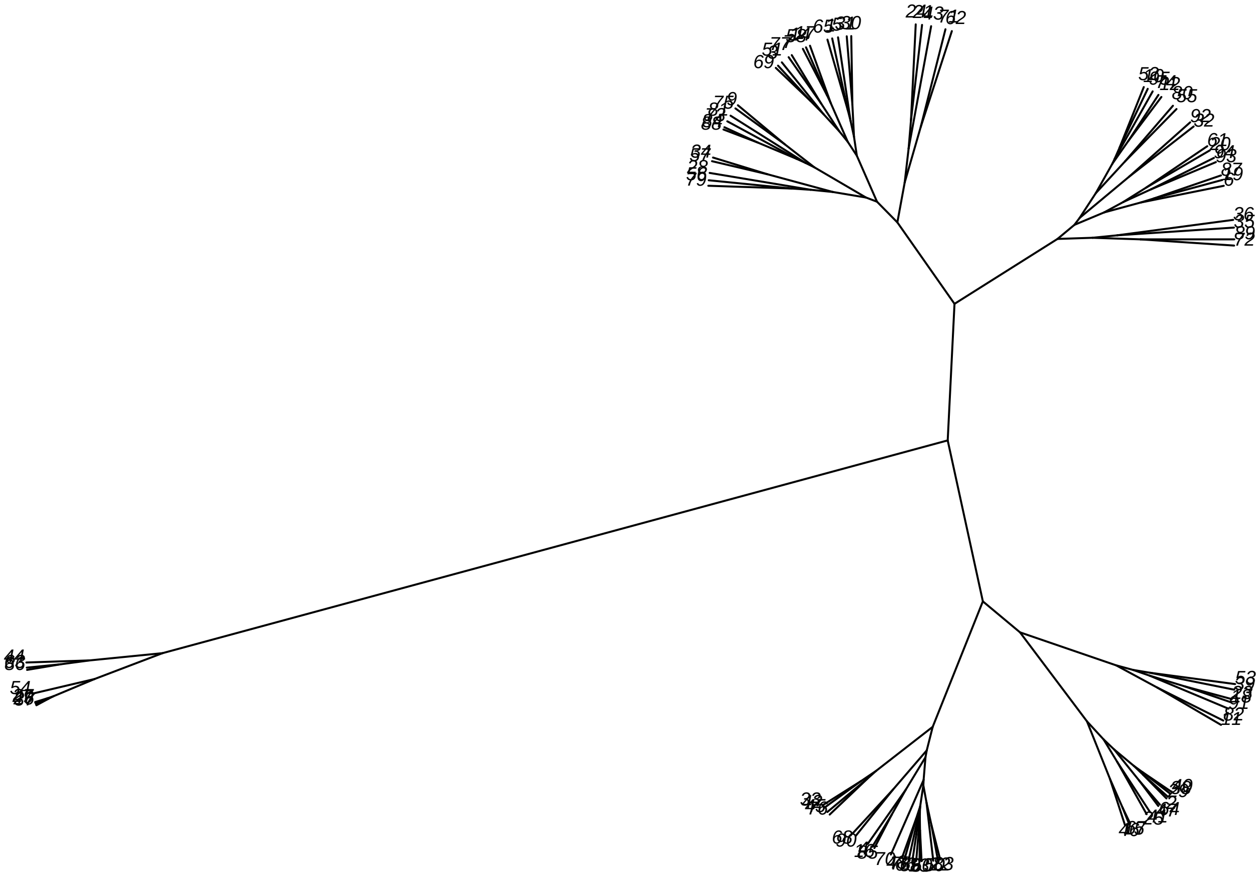

USA Area 1 Fan Cluster dendrogram

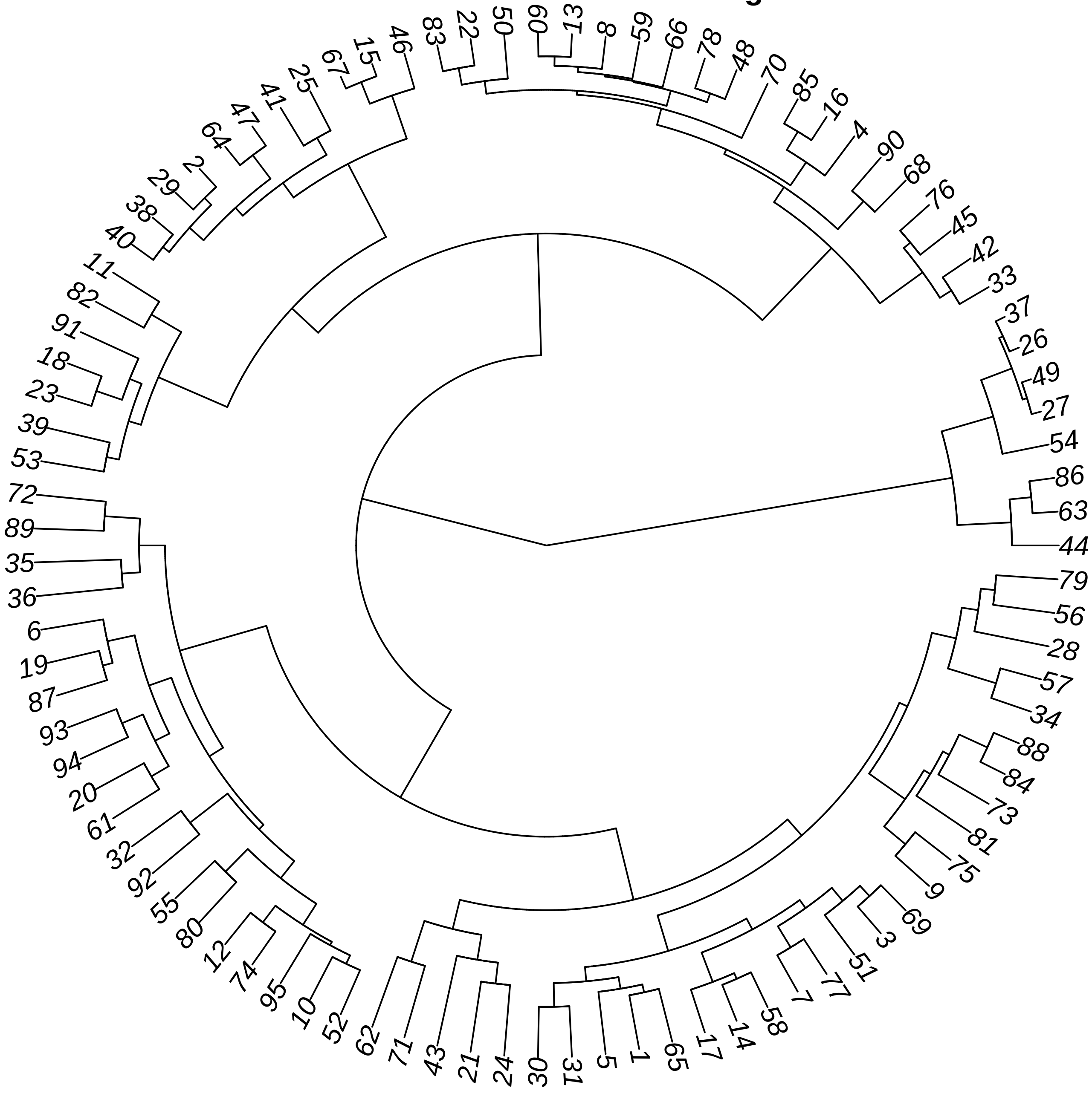

USA Area 1 at h = 15 : Cluster dendrogram

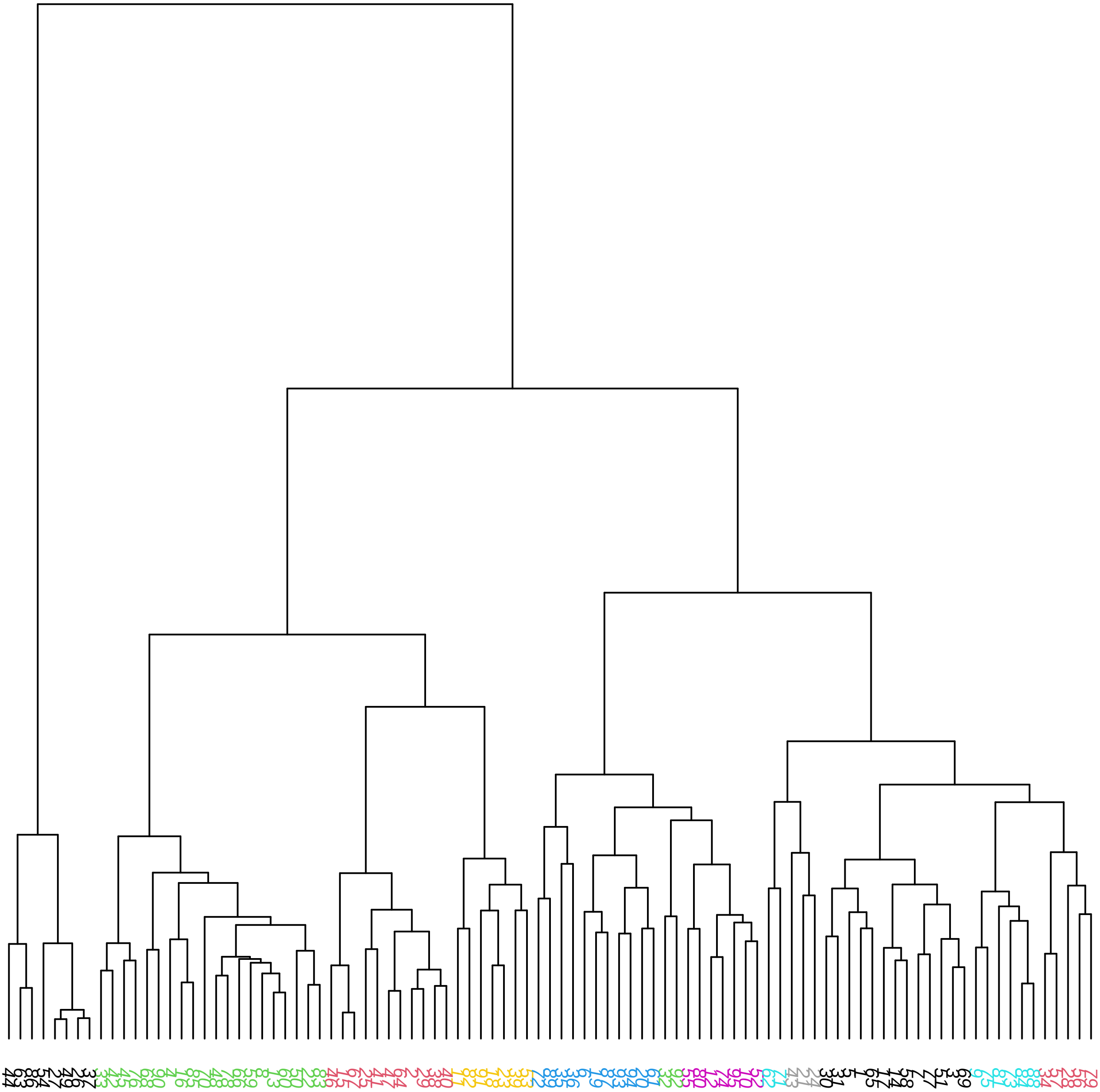

USA Area 1 at h = 15 : Coloured Unrooted Cluster dendrogram

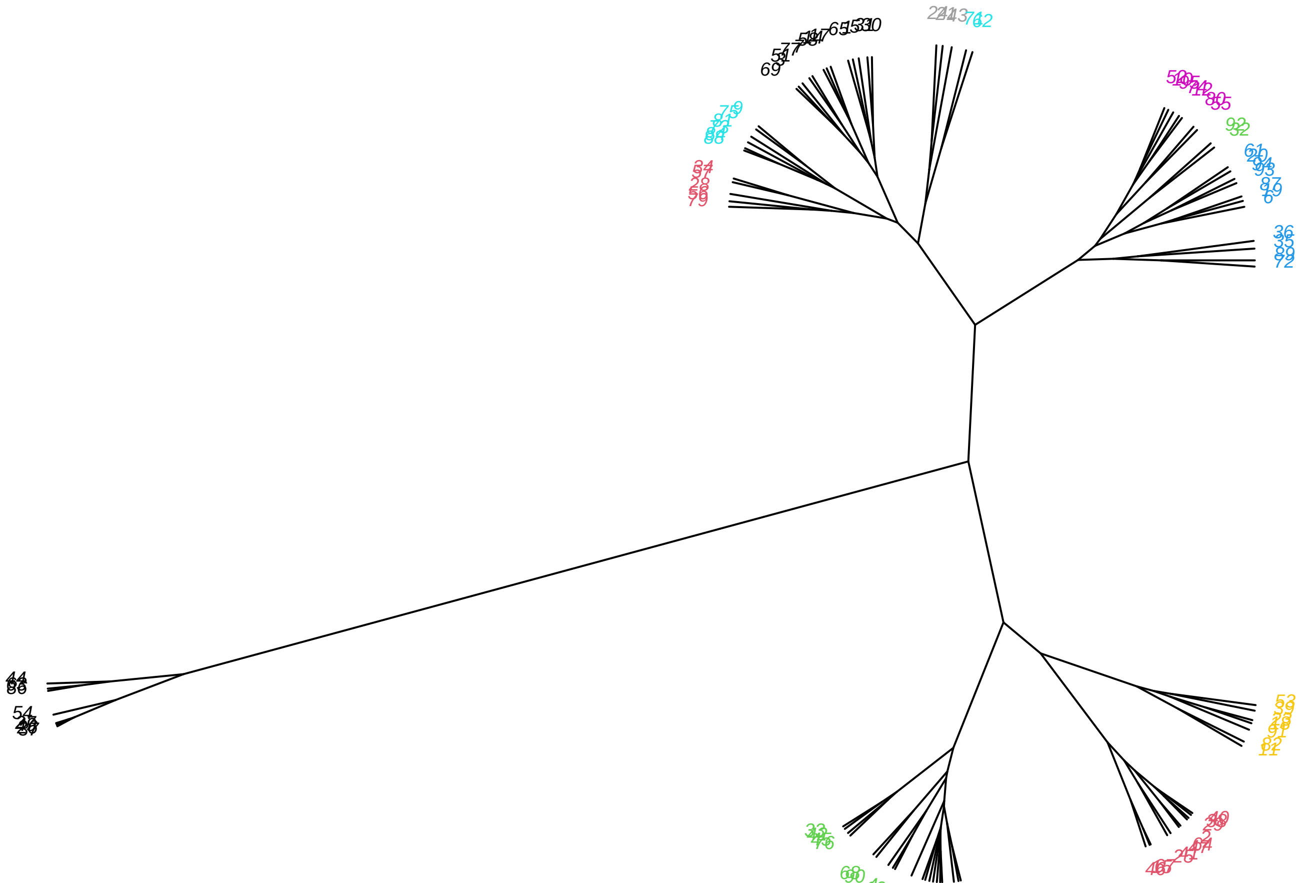

USA Area 1 at h = 15 : Coloured Fan Cluster dendrogram

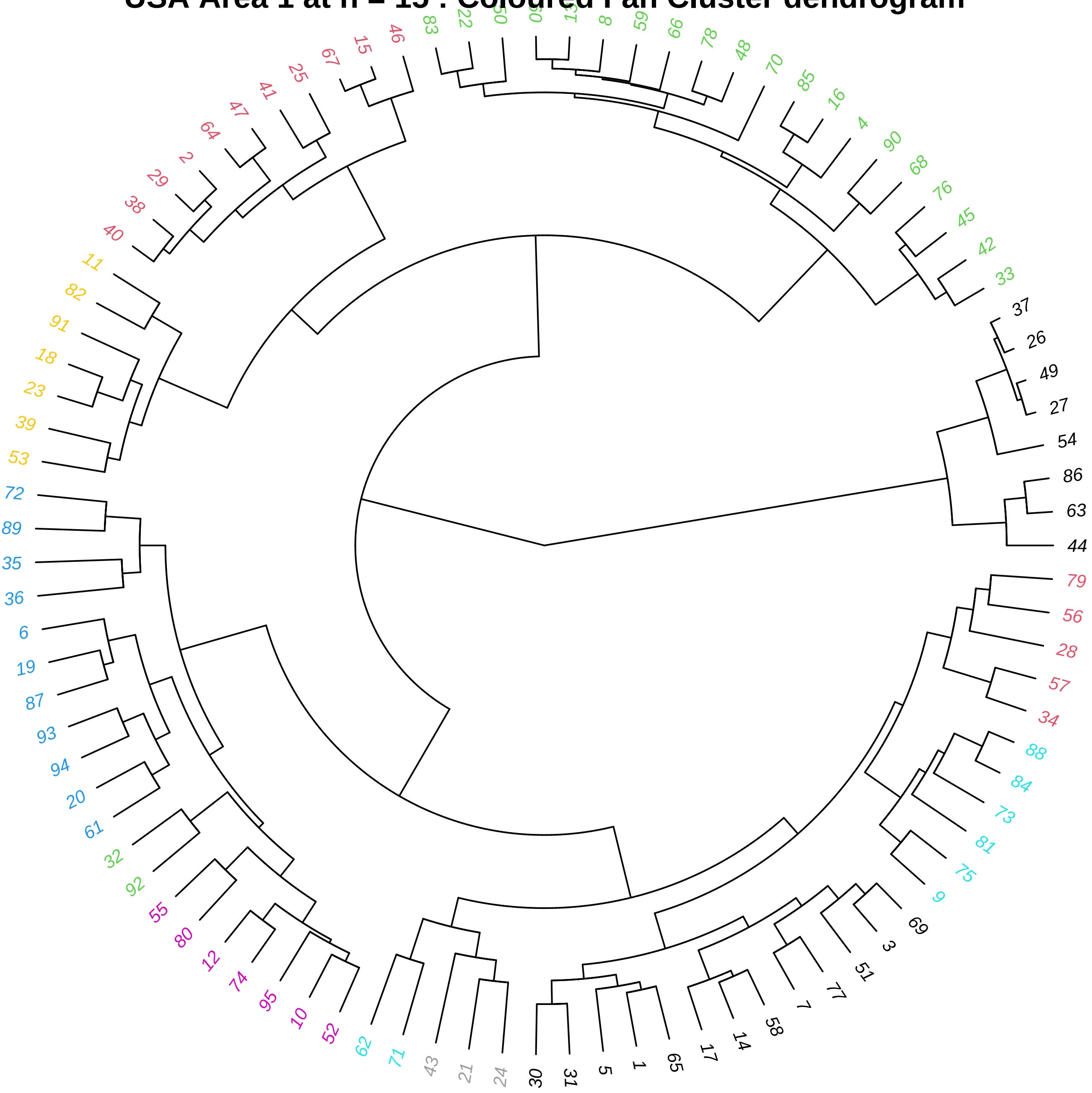

USA Area 2 with cluster c(20, 20, 50, 50, 50, 20, 50) Cluster dendrogram

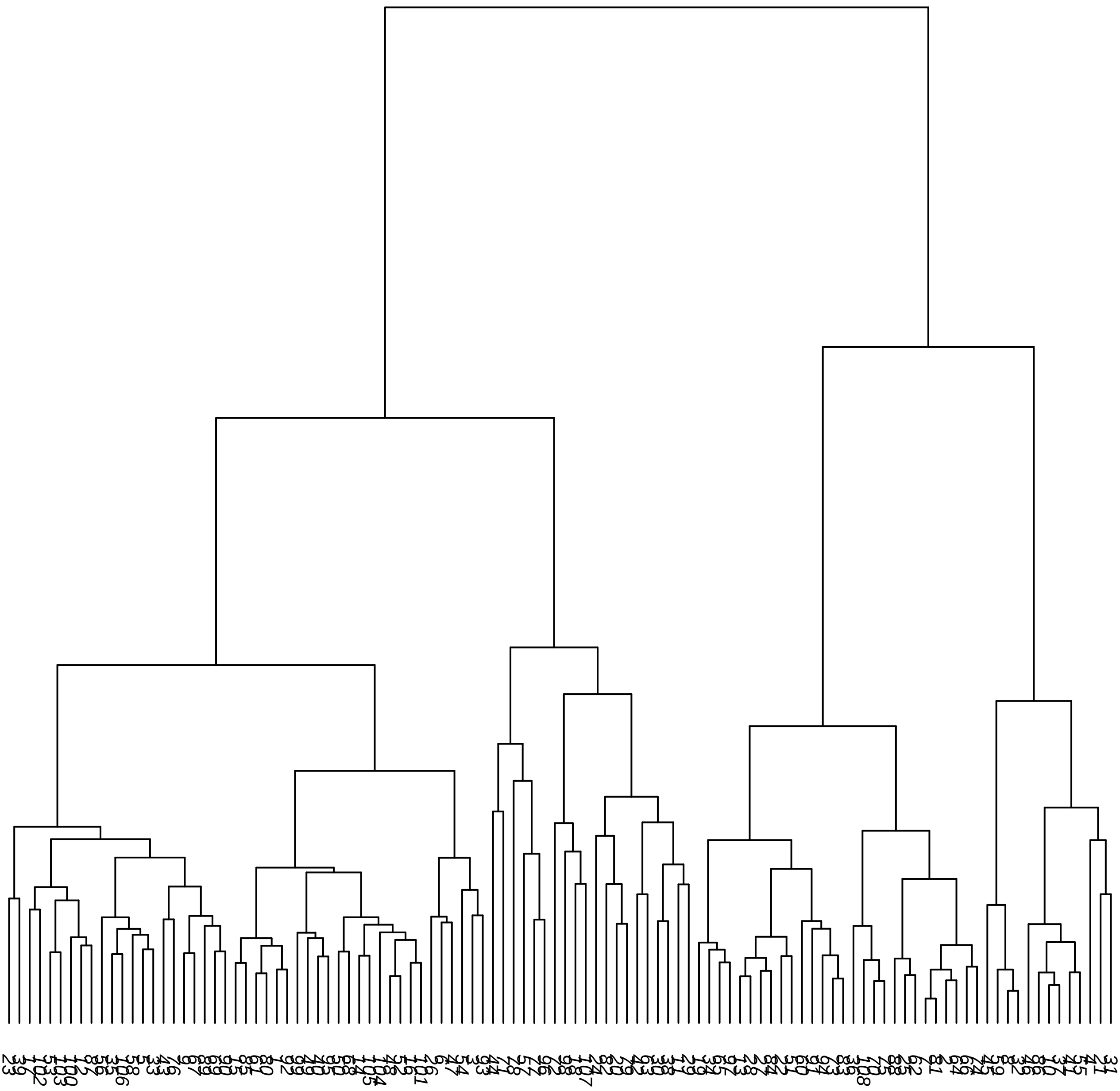

USA Area 2 Unrooted Cluster dendrogram

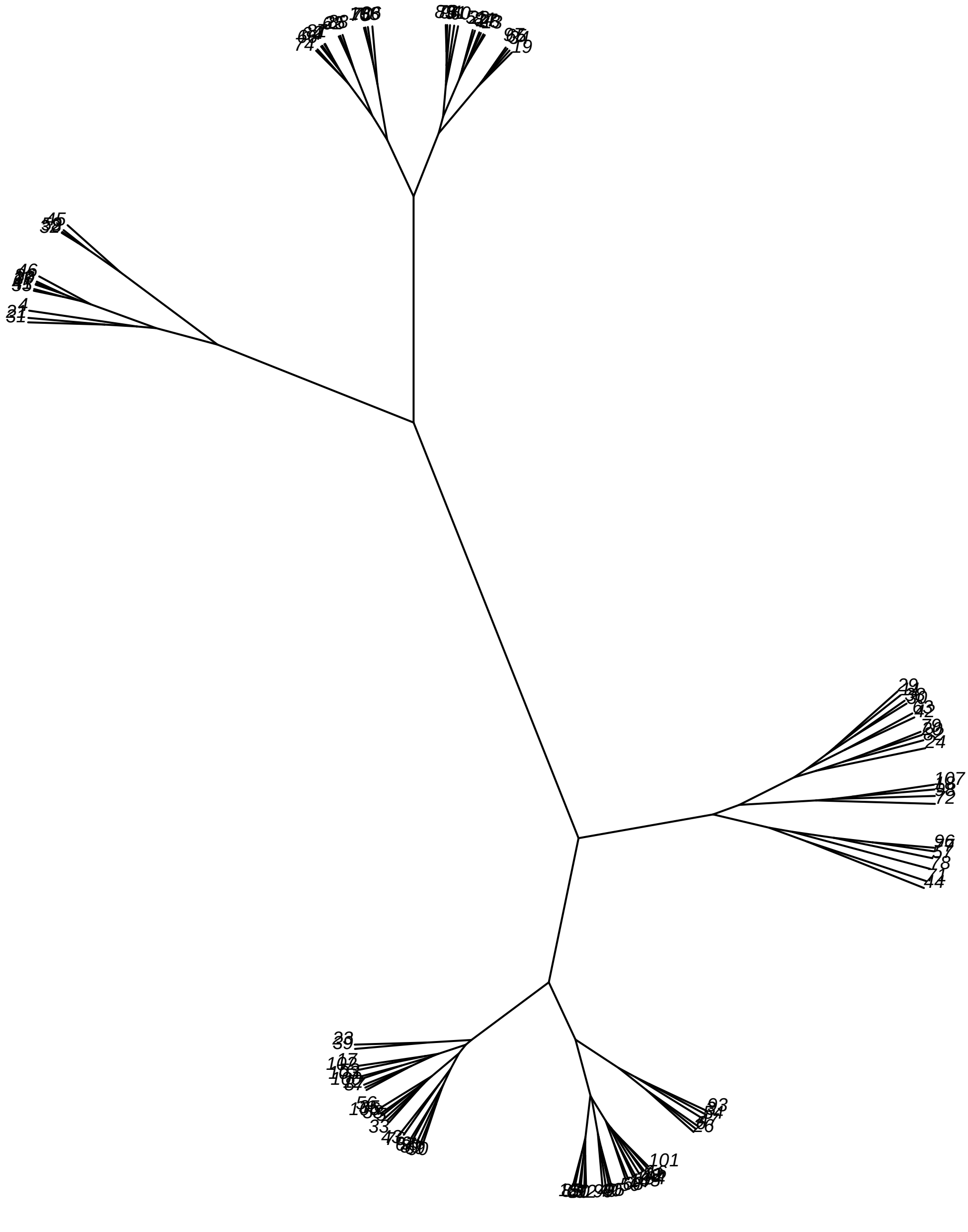

USA Area 2 Fan Cluster dendrogram

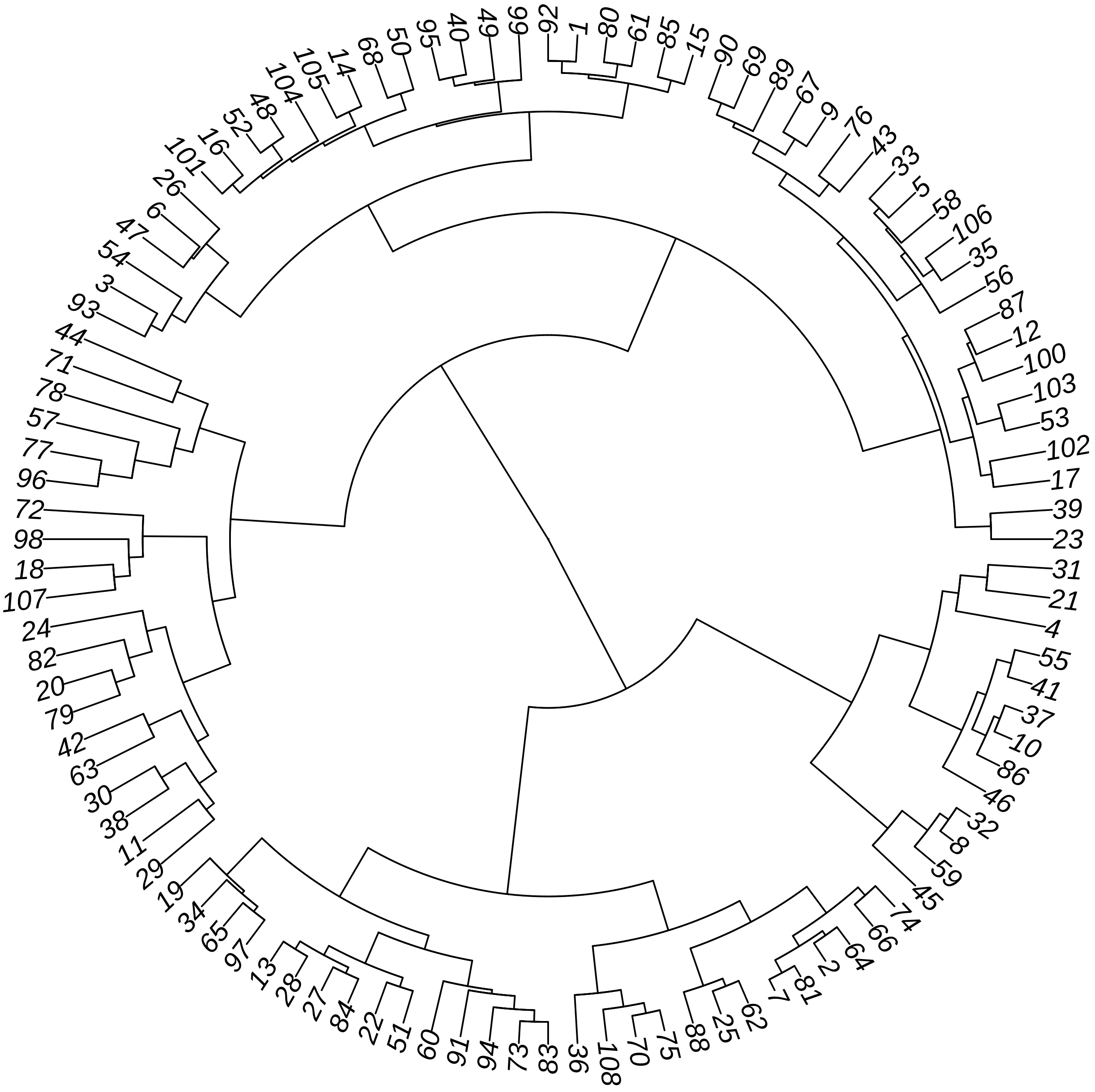

USA Area 2 at h = 15 : Cluster dendrogram

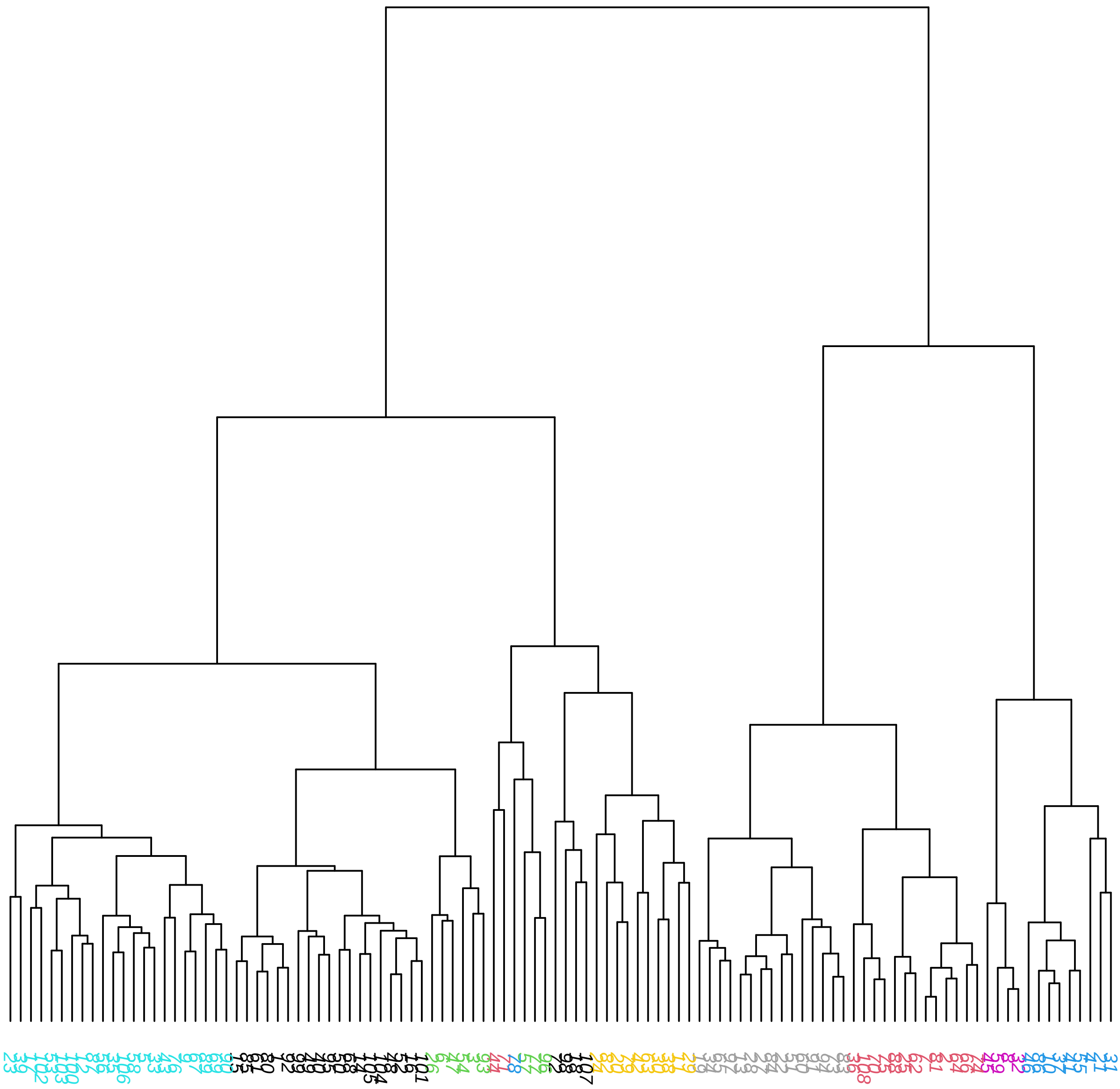

USA Area 2 at h = 15 : Coloured Unrooted Cluster dendrogram

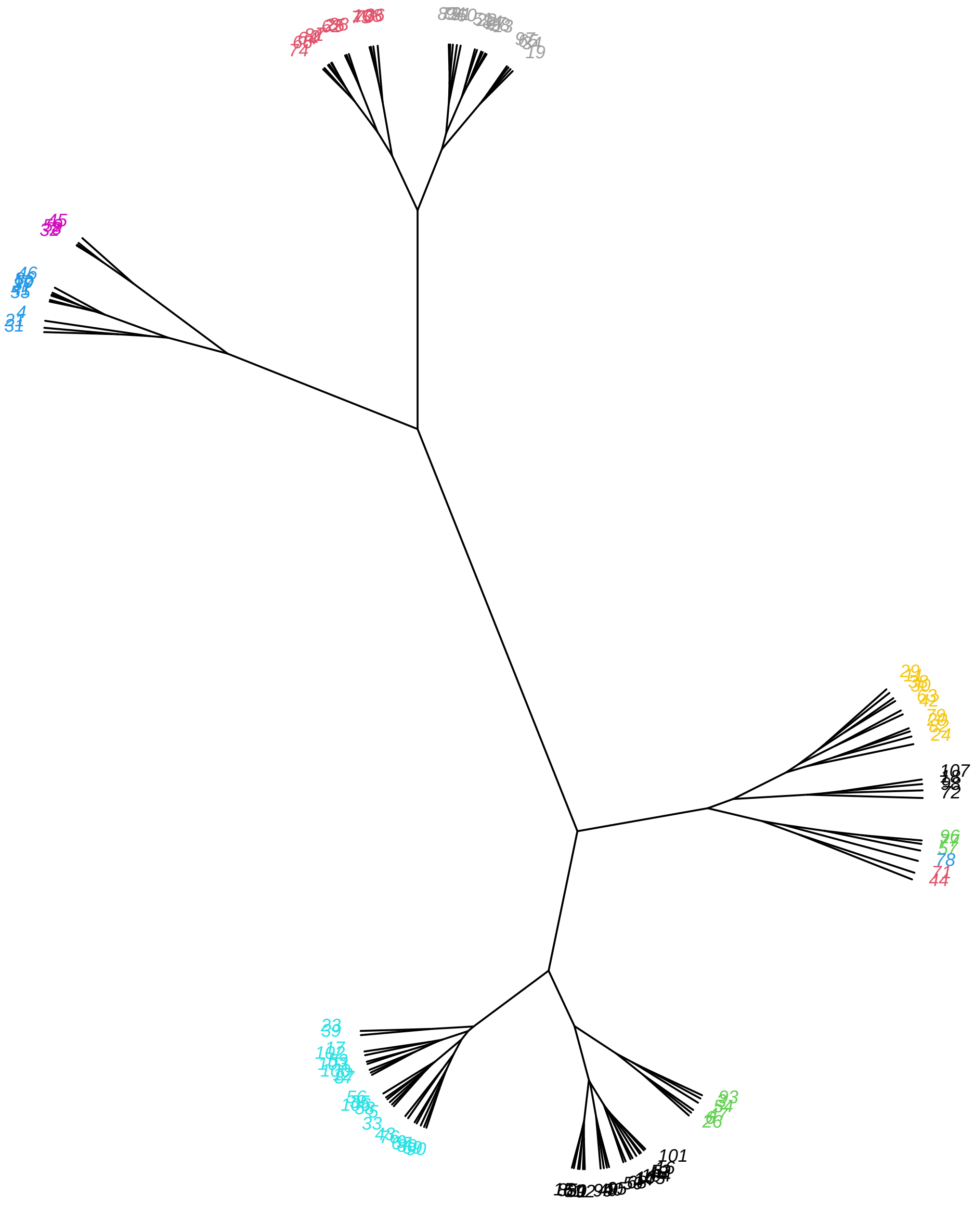

USA Area 2 at h = 15 : Coloured Fan Cluster dendrogram

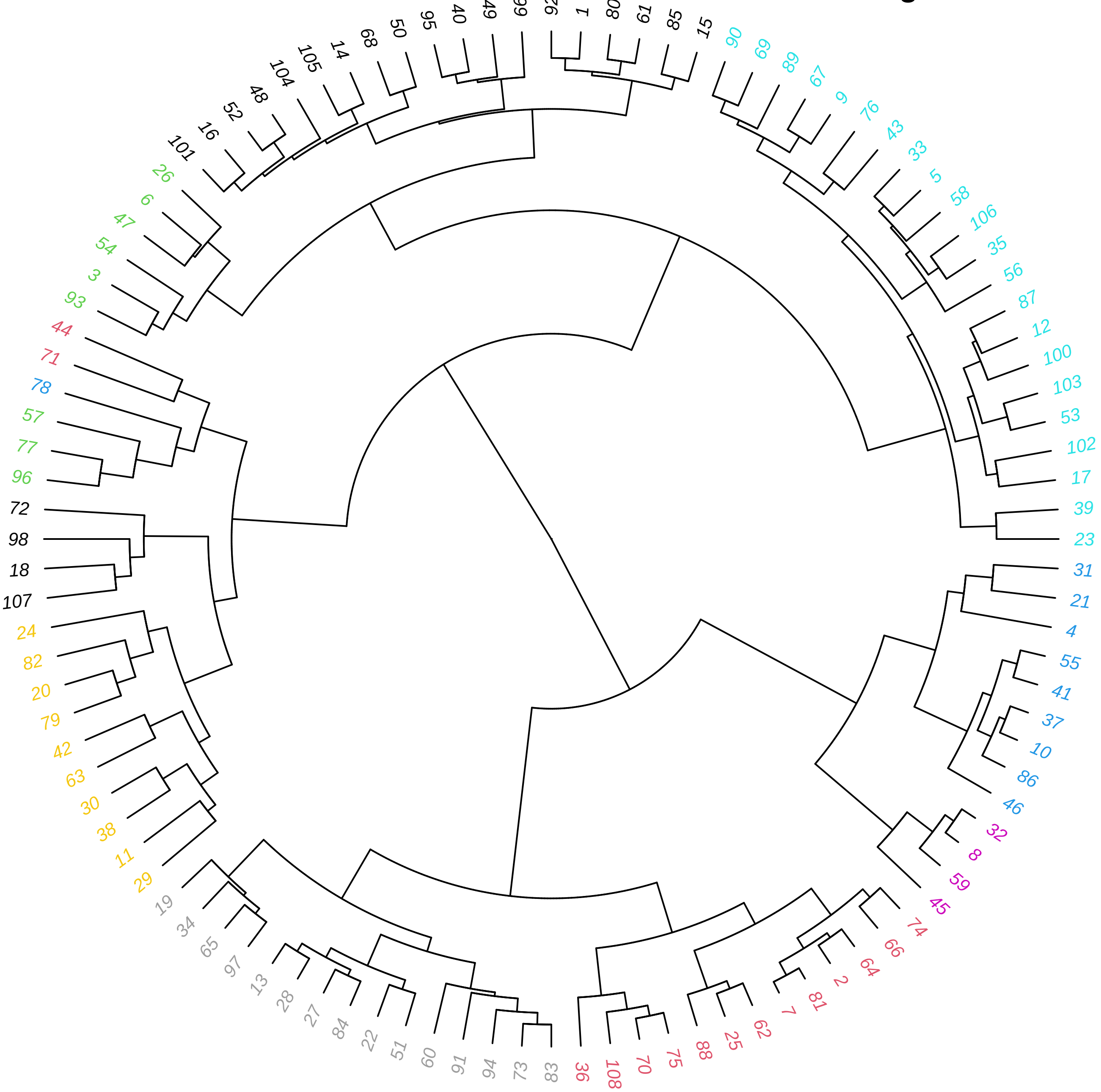

USA Area 3 with cluster c(20, 20, 50, 50, 50, 20, 50) Cluster dendrogram

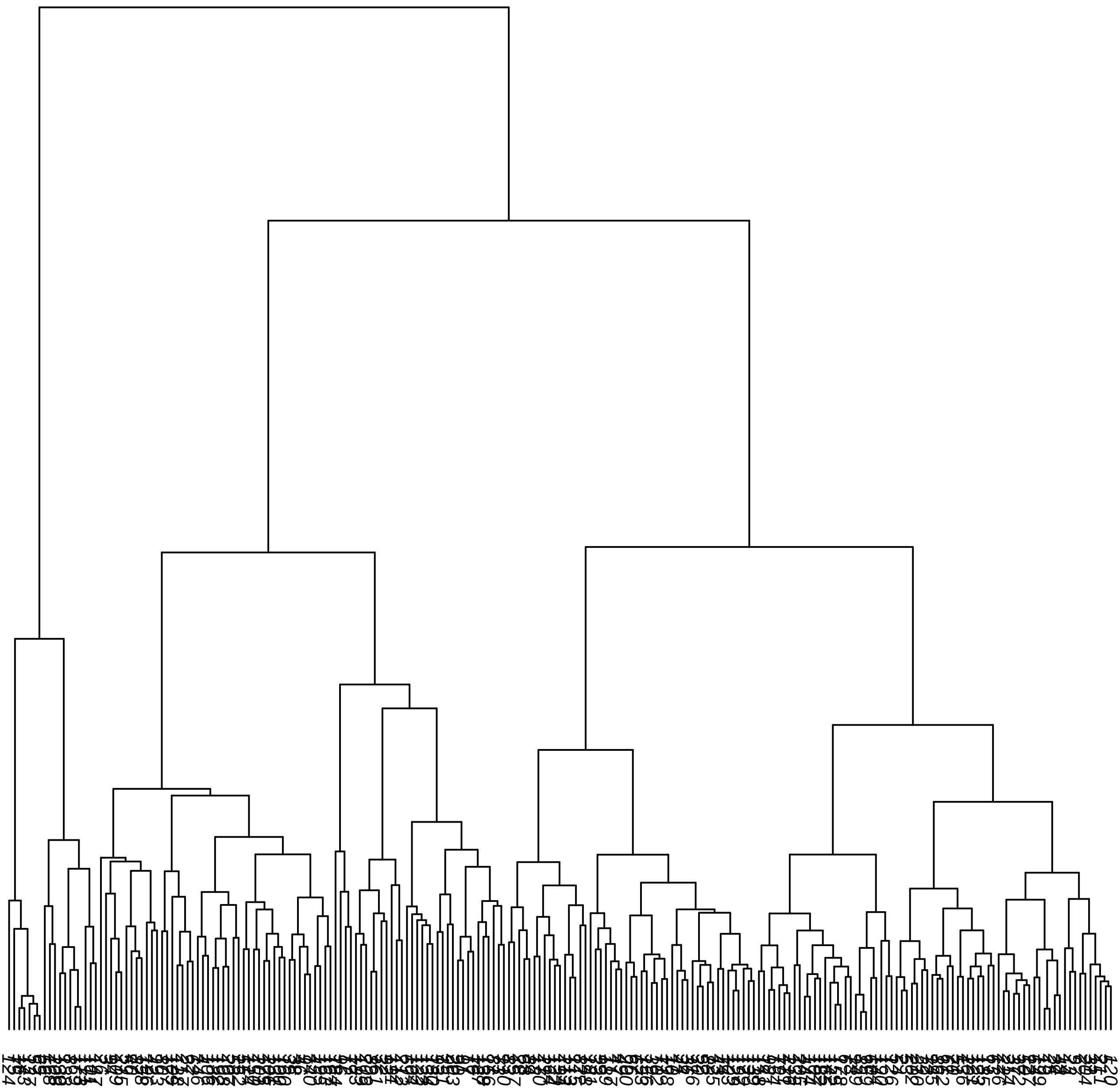

USA Area 3 Unrooted Cluster dendrogram

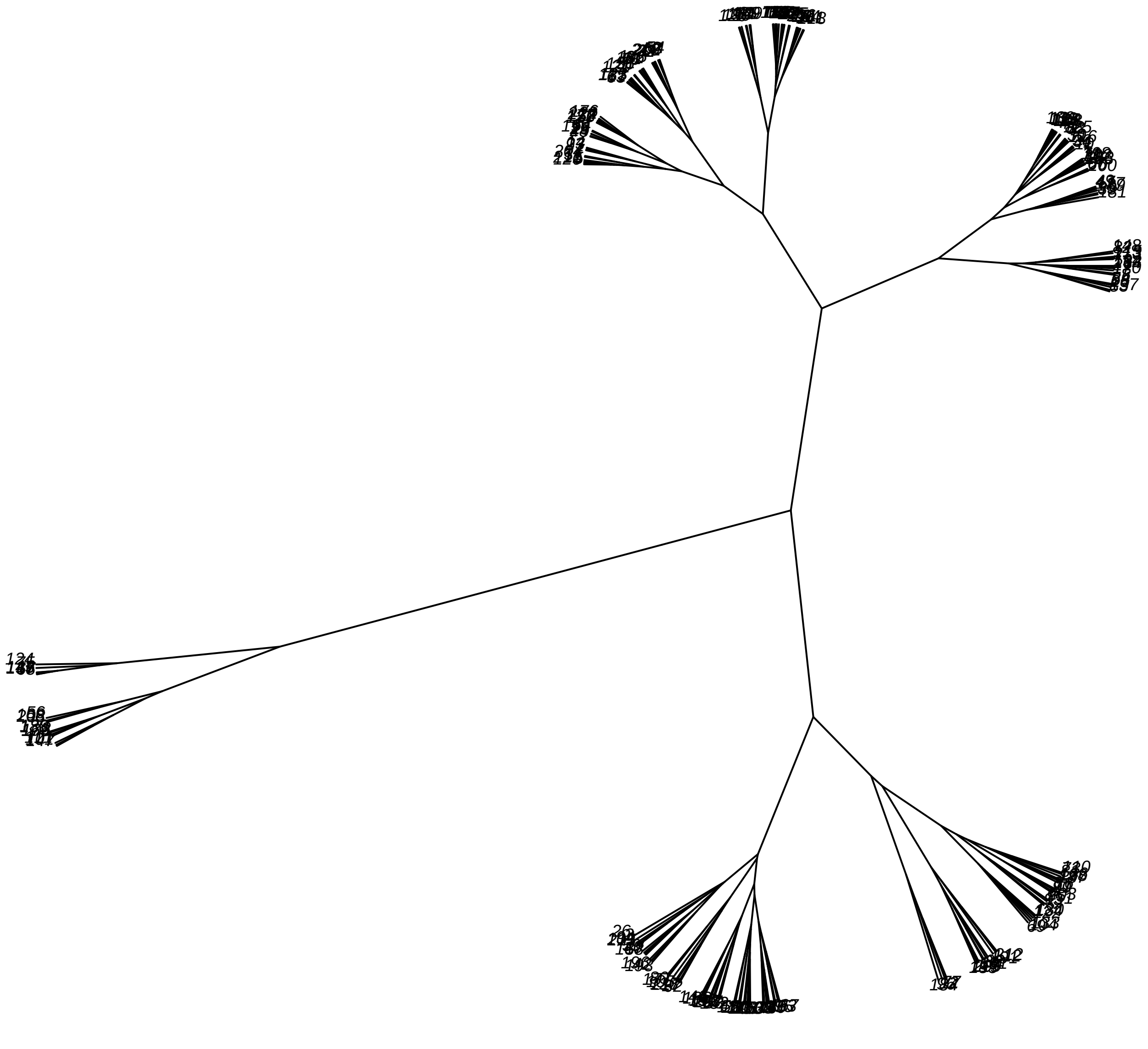

USA Area 3 Fan Cluster dendrogram

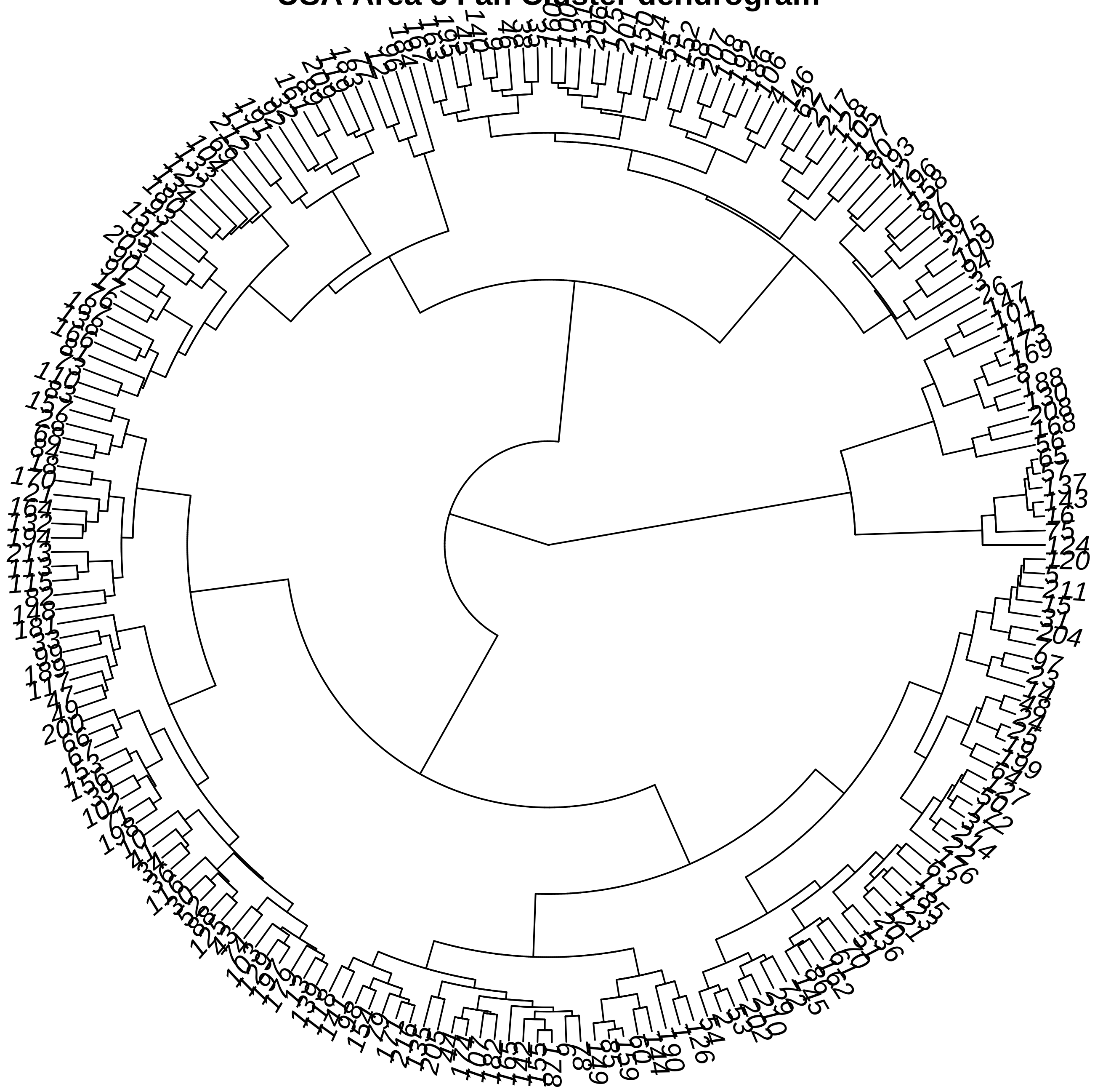

USA Area 3 at h = 15 : Cluster dendrogram

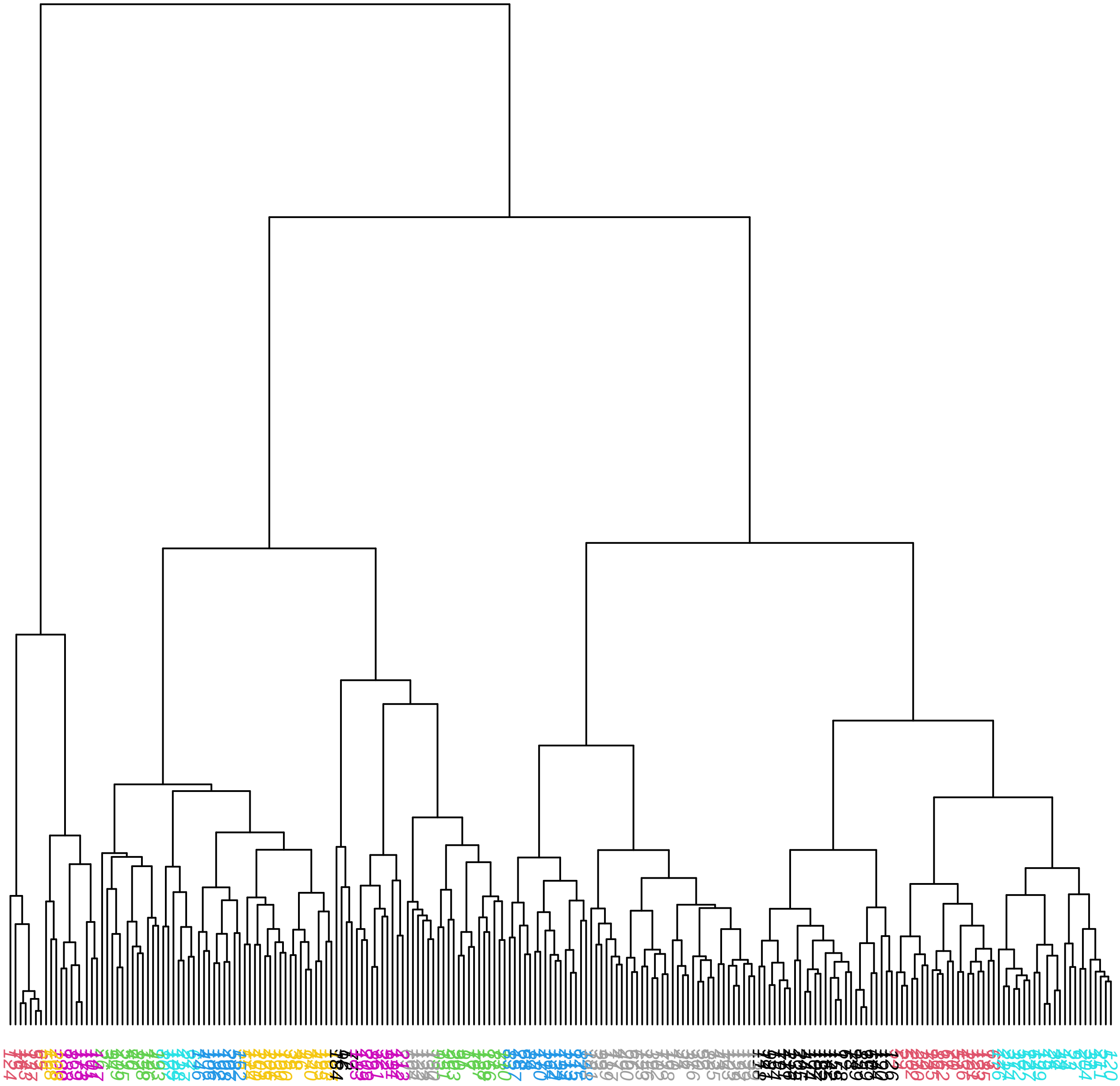

USA Area 3 at h = 15 : Coloured Unrooted Cluster dendrogram

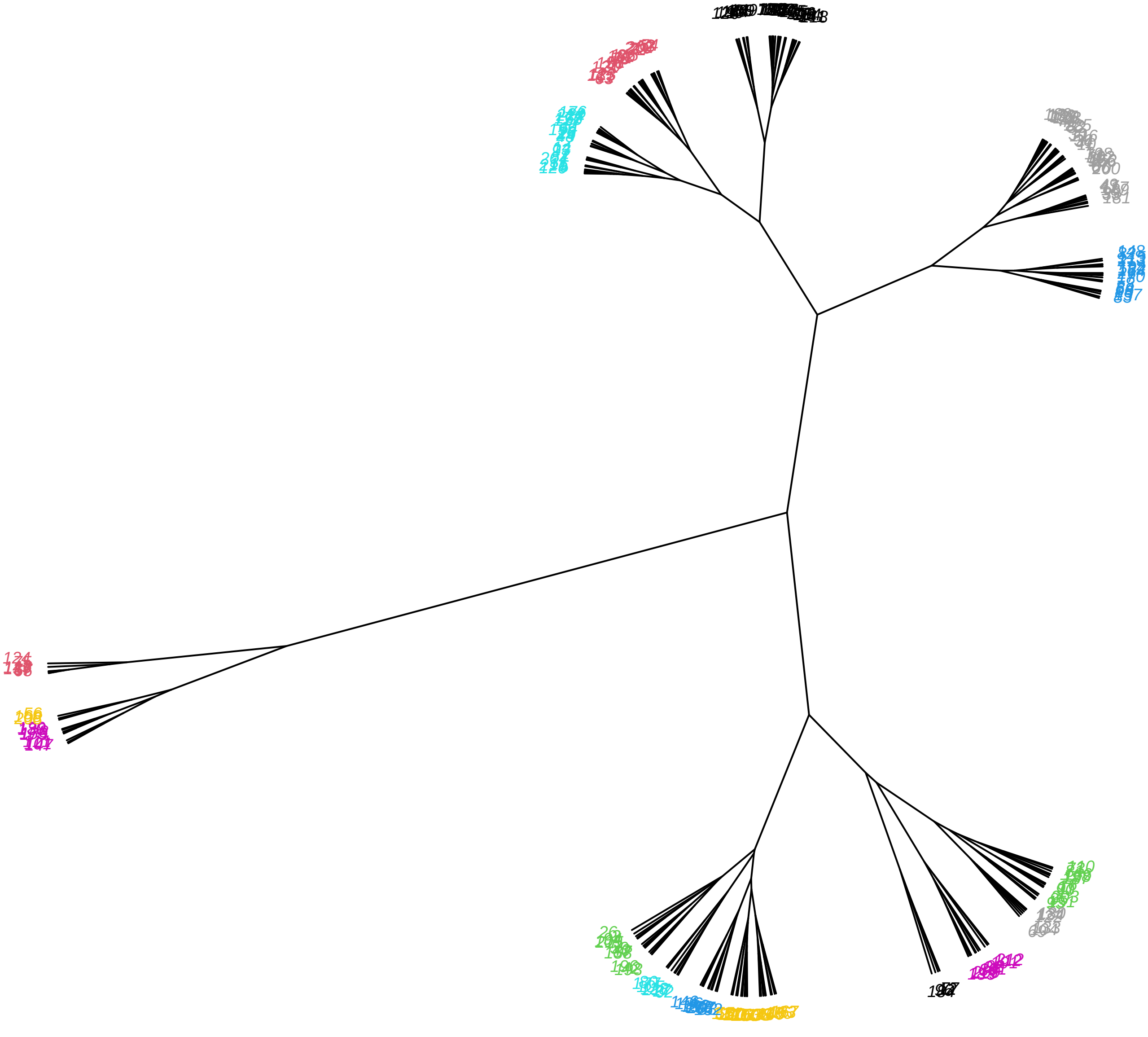

USA Area 3 at h = 15 : Coloured Fan Cluster dendrogram

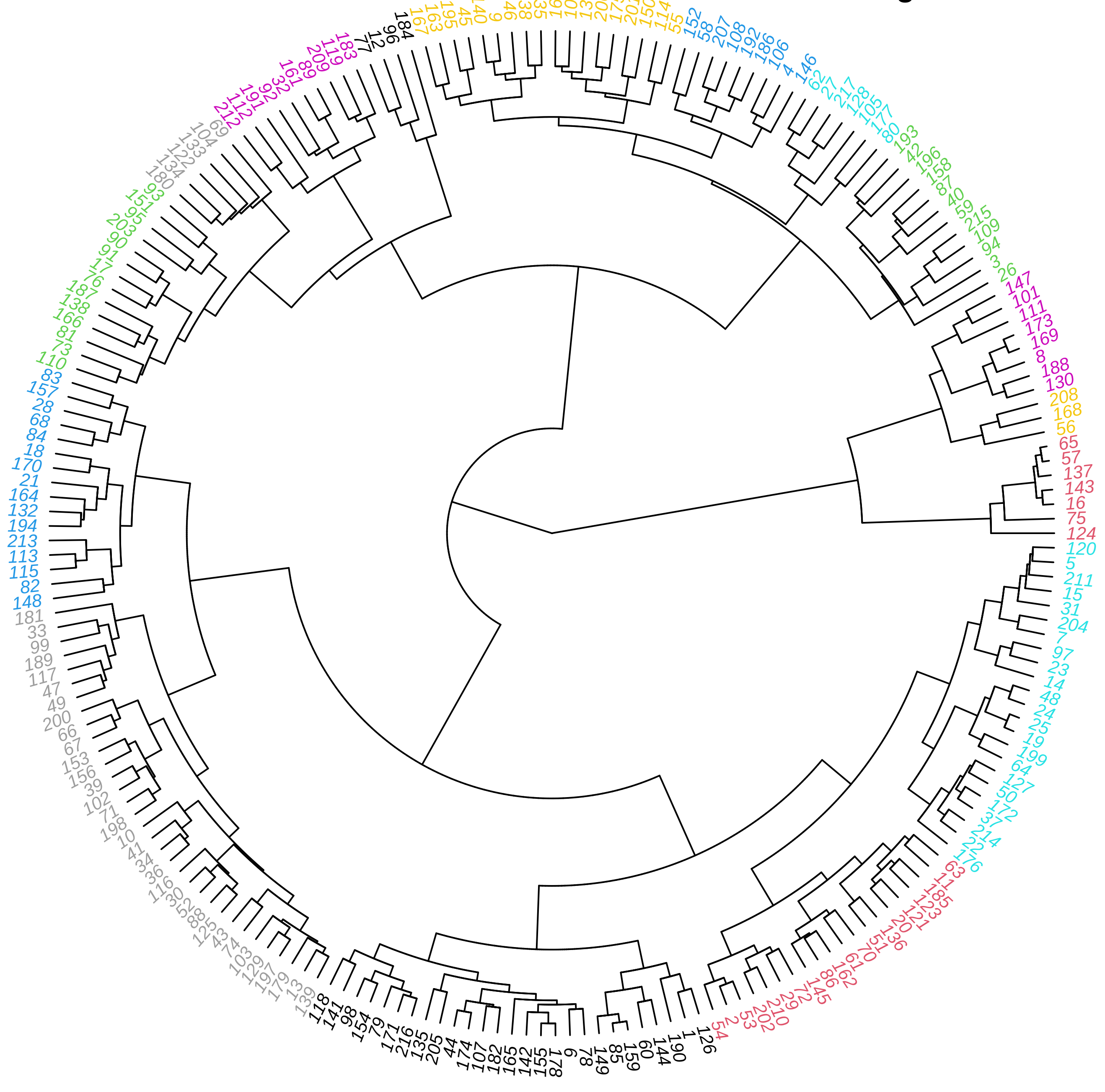

USA Area 4 with cluster c(20, 20, 50, 50, 50, 20, 50) Cluster dendrogram

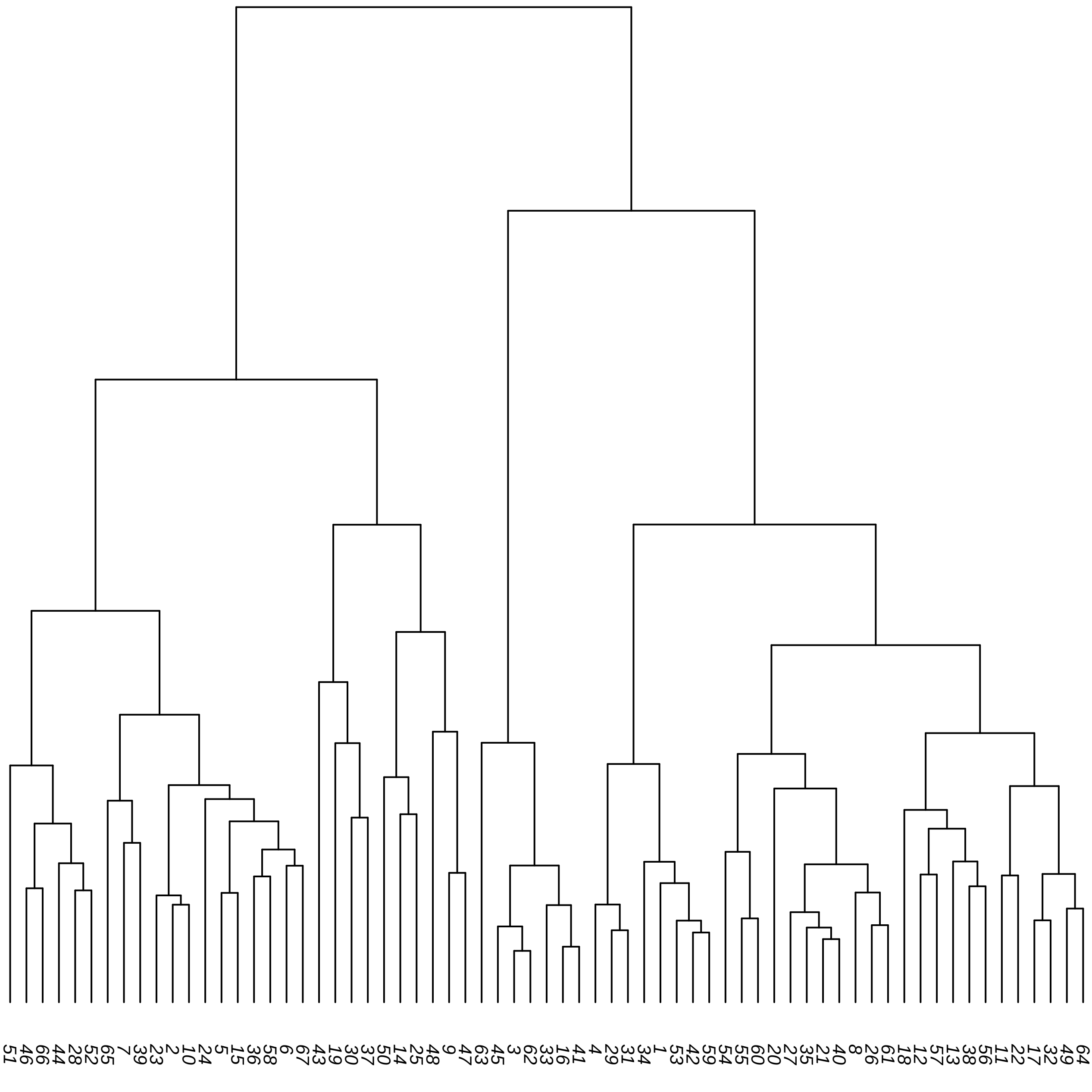

USA Area 4 Unrooted Cluster dendrogram

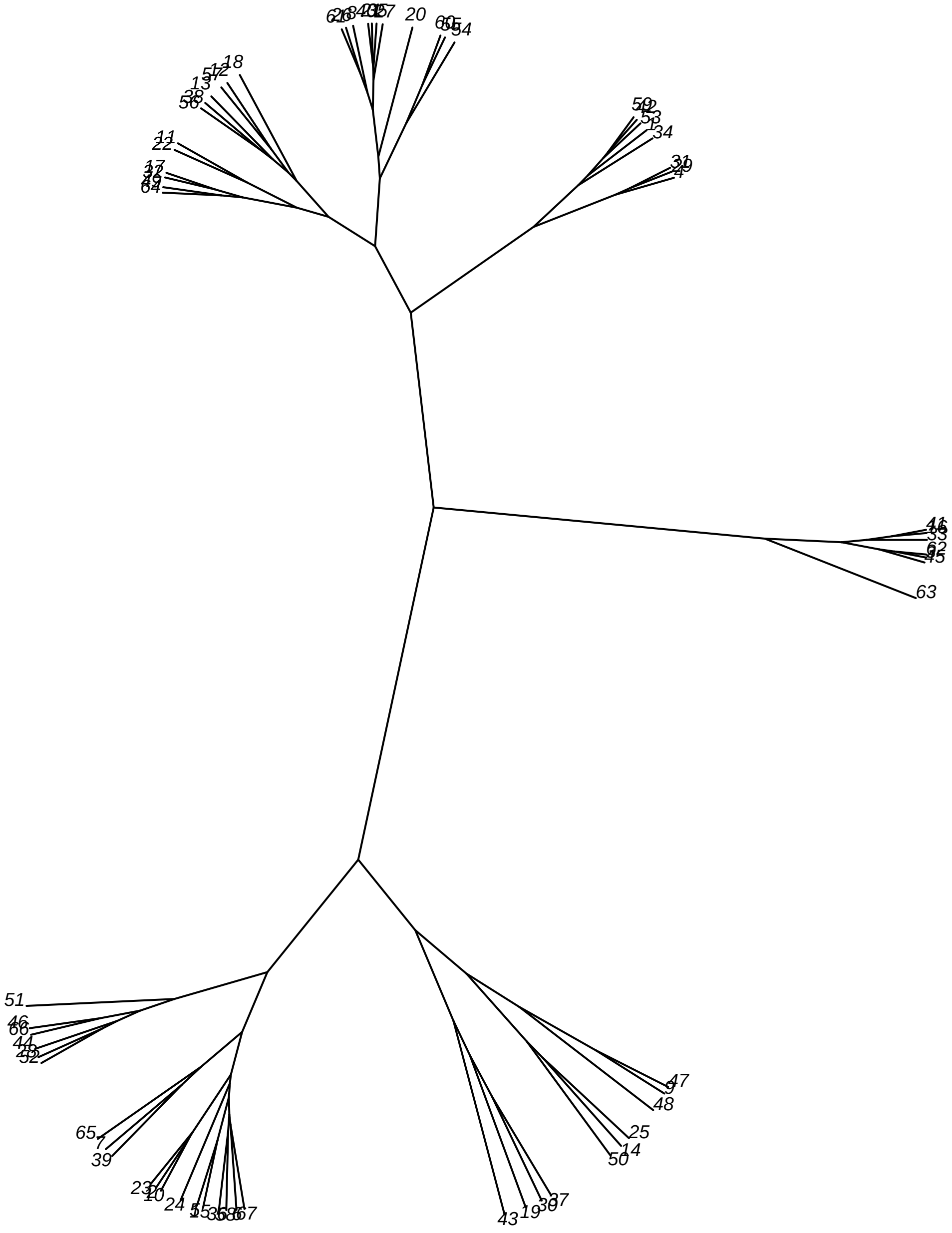

USA Area 4 Fan Cluster dendrogram

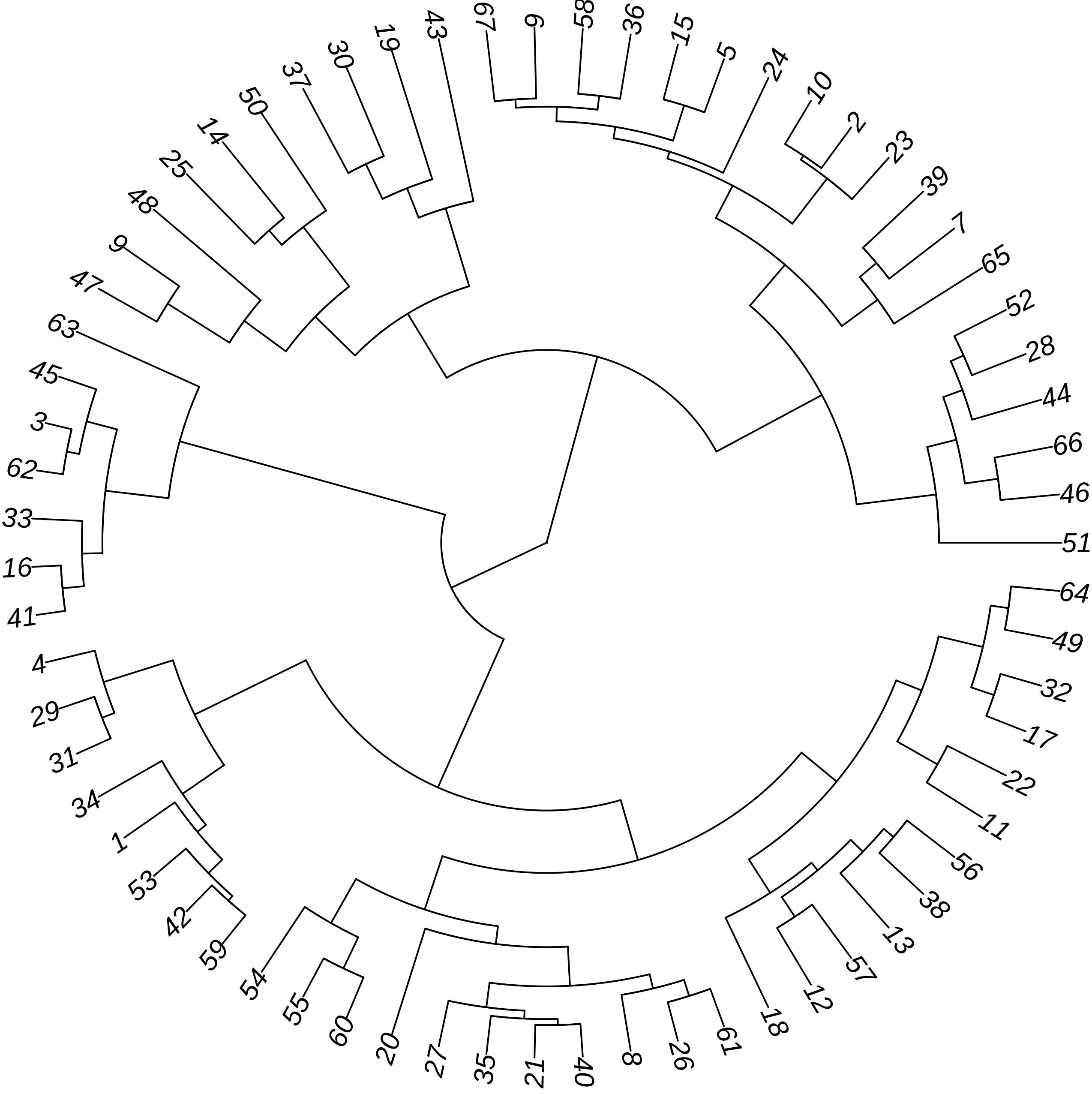

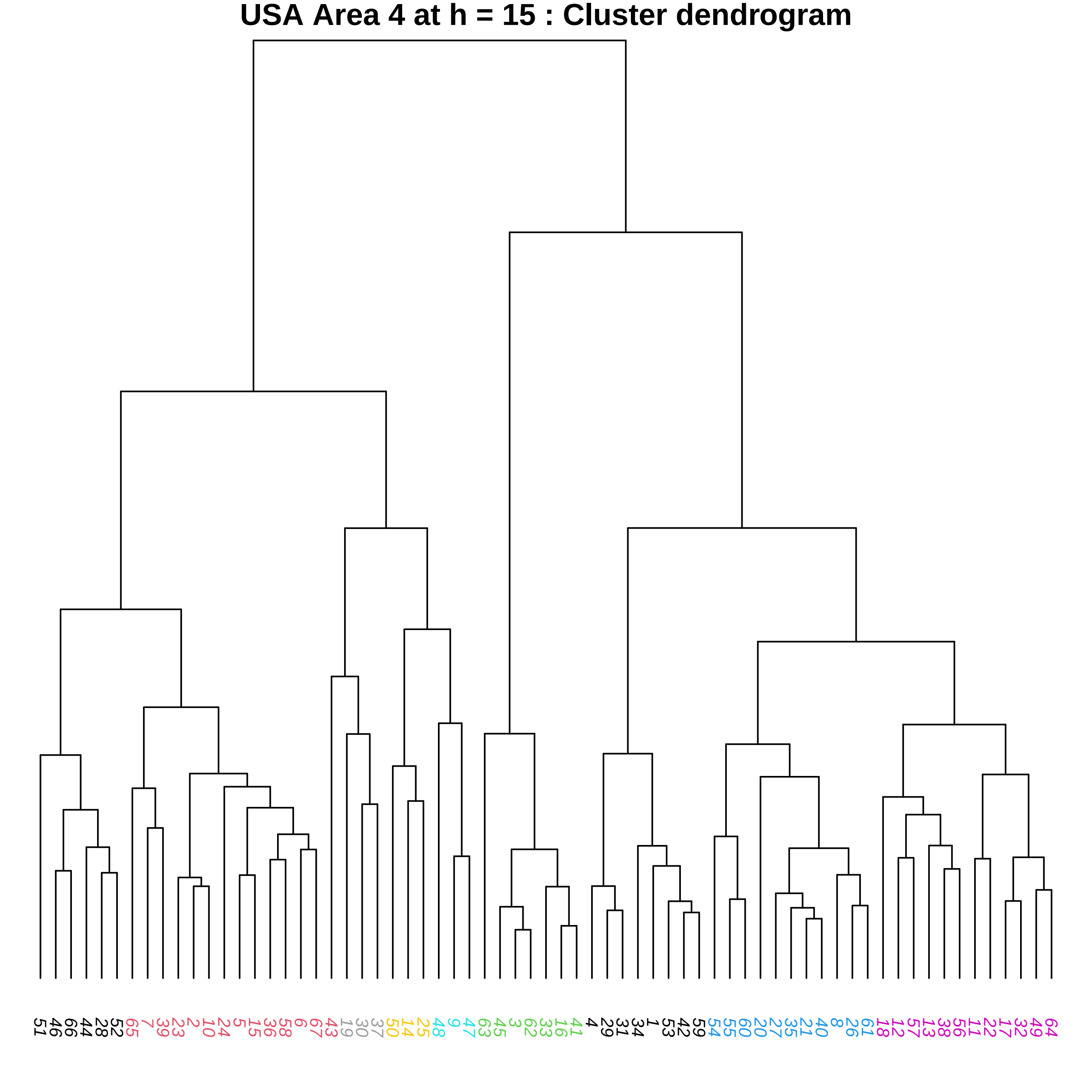
USA Area 4 at h = 15 : Cluster dendrogram
51 46 66 44 28 52 65 7 39 23 2 10 24 5 15 36 58 6 67 43 19 30 37 50 14 25 48 9 47 63 45 3 62 33 16 41 4 29 31 34 1 53 42 59 54 55 60 20 27 35 21 40 8 26 61 18 12 57 13 38 56 11 22 17 32 49 64

USA Area 4 at h = 15 : Coloured Unrooted Cluster dendrogram

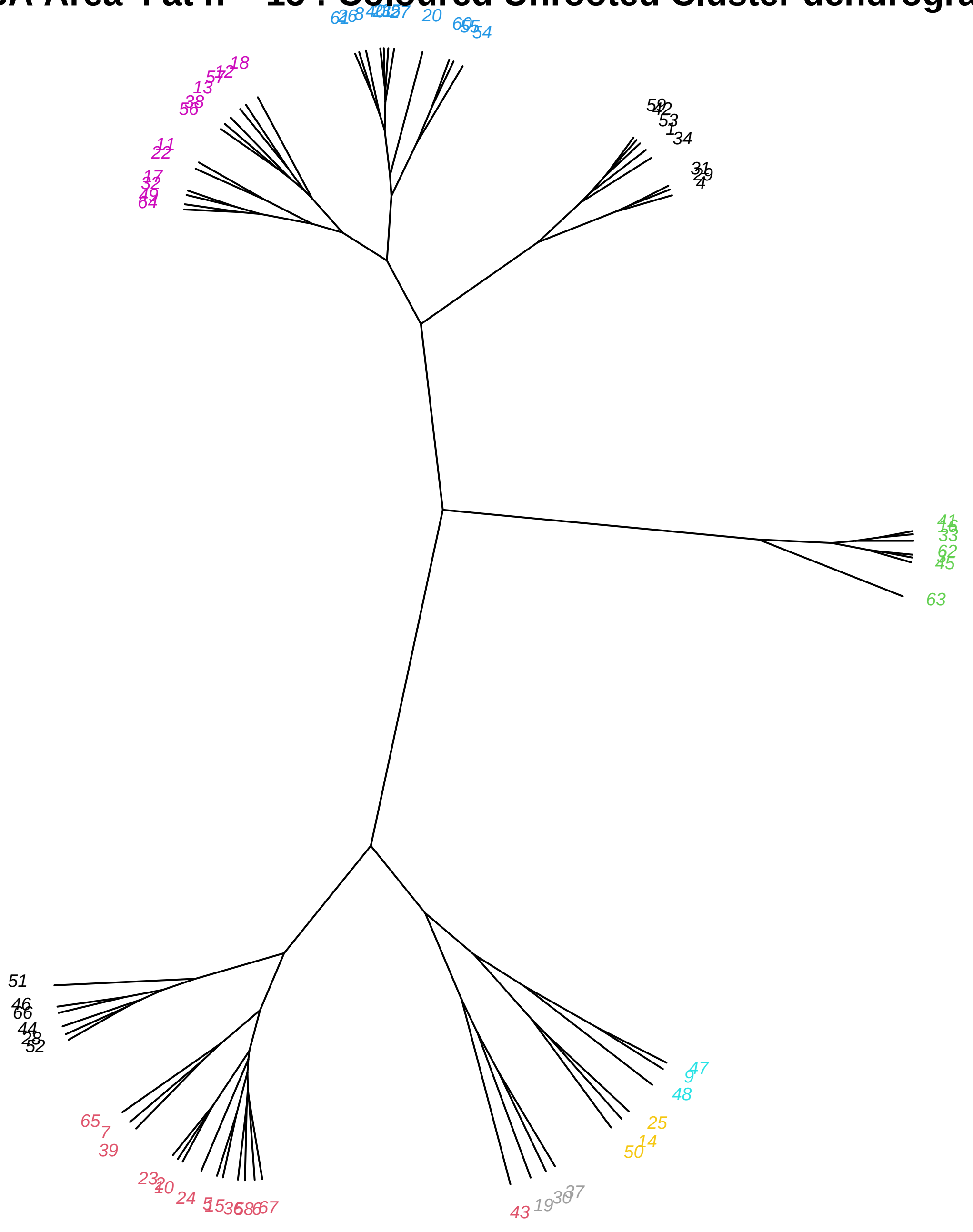

USA Area 4 at h = 15 : Coloured Fan Cluster dendrogram

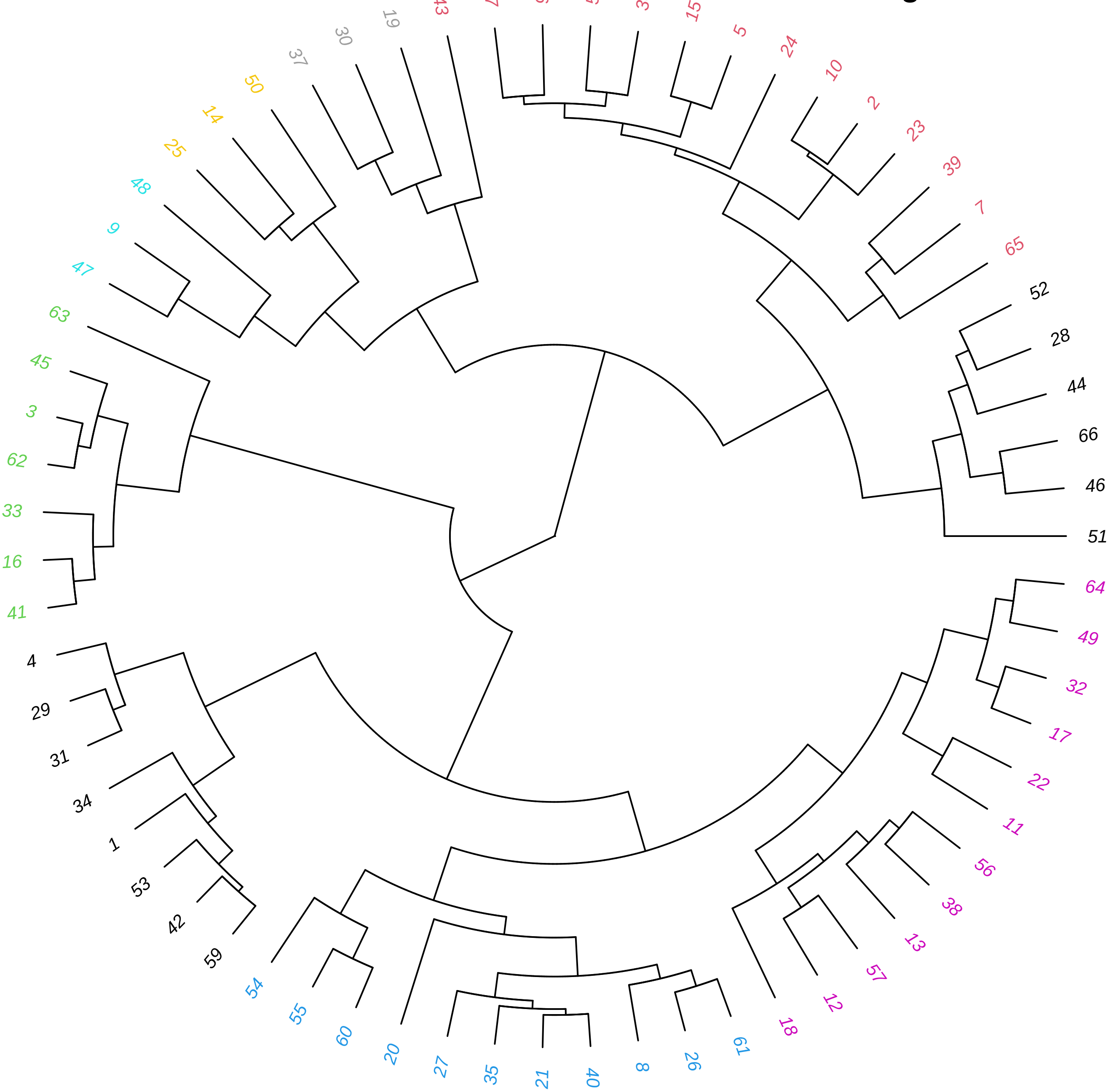

USA Area 5 with cluster c(20, 20, 50, 50, 50, 20, 50) Cluster dendrogram

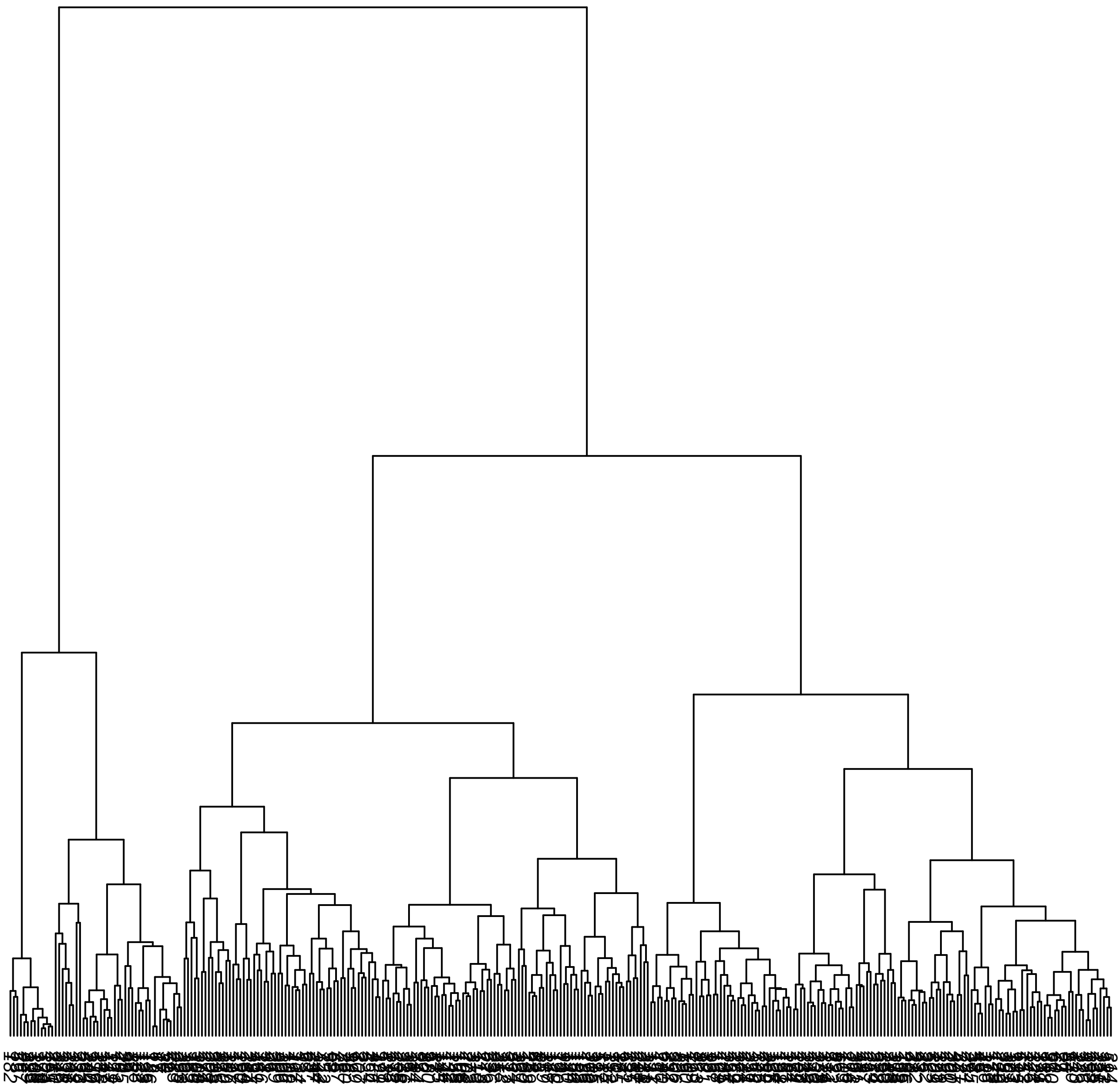

USA Area 5 Unrooted Cluster dendrogram

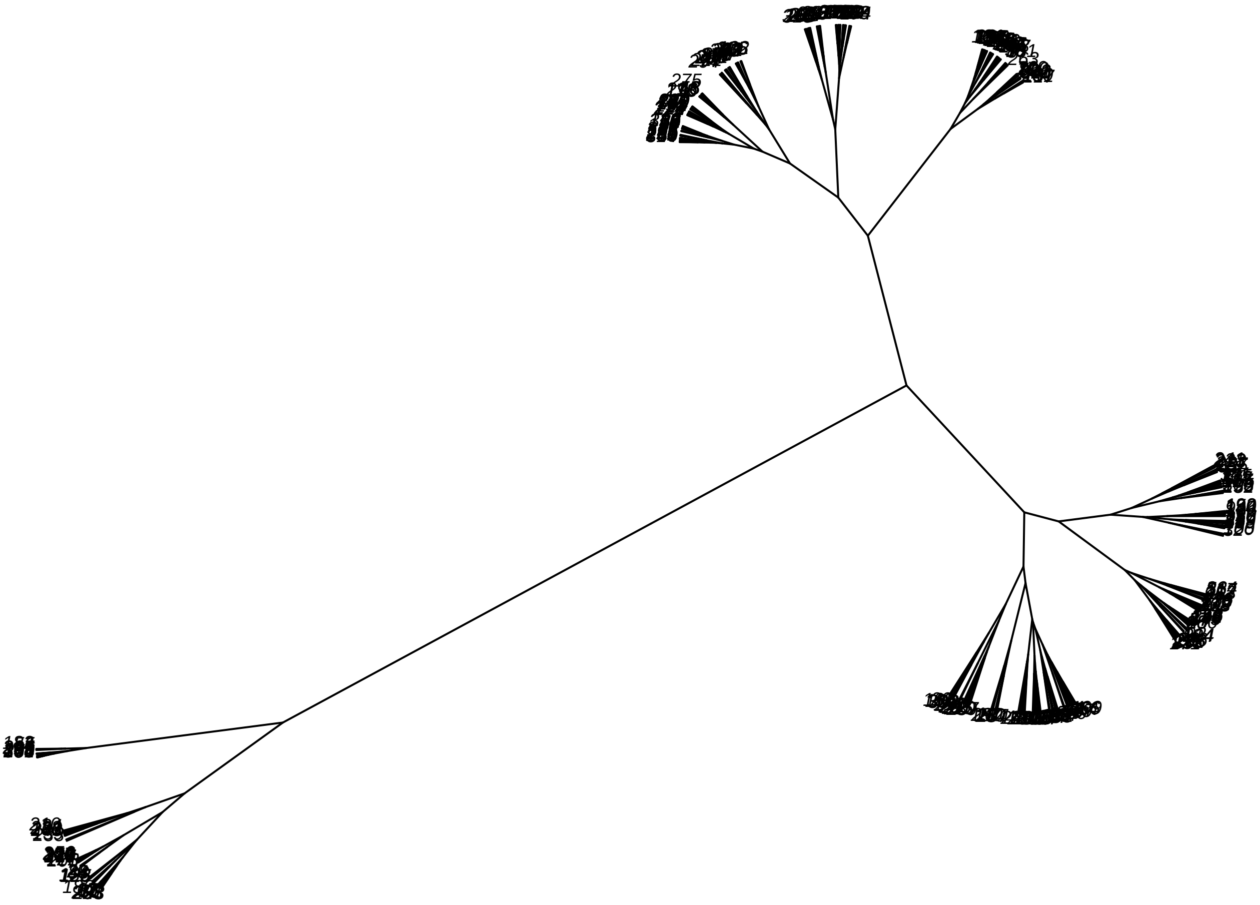

USA Area 5 Fan Cluster dendrogram

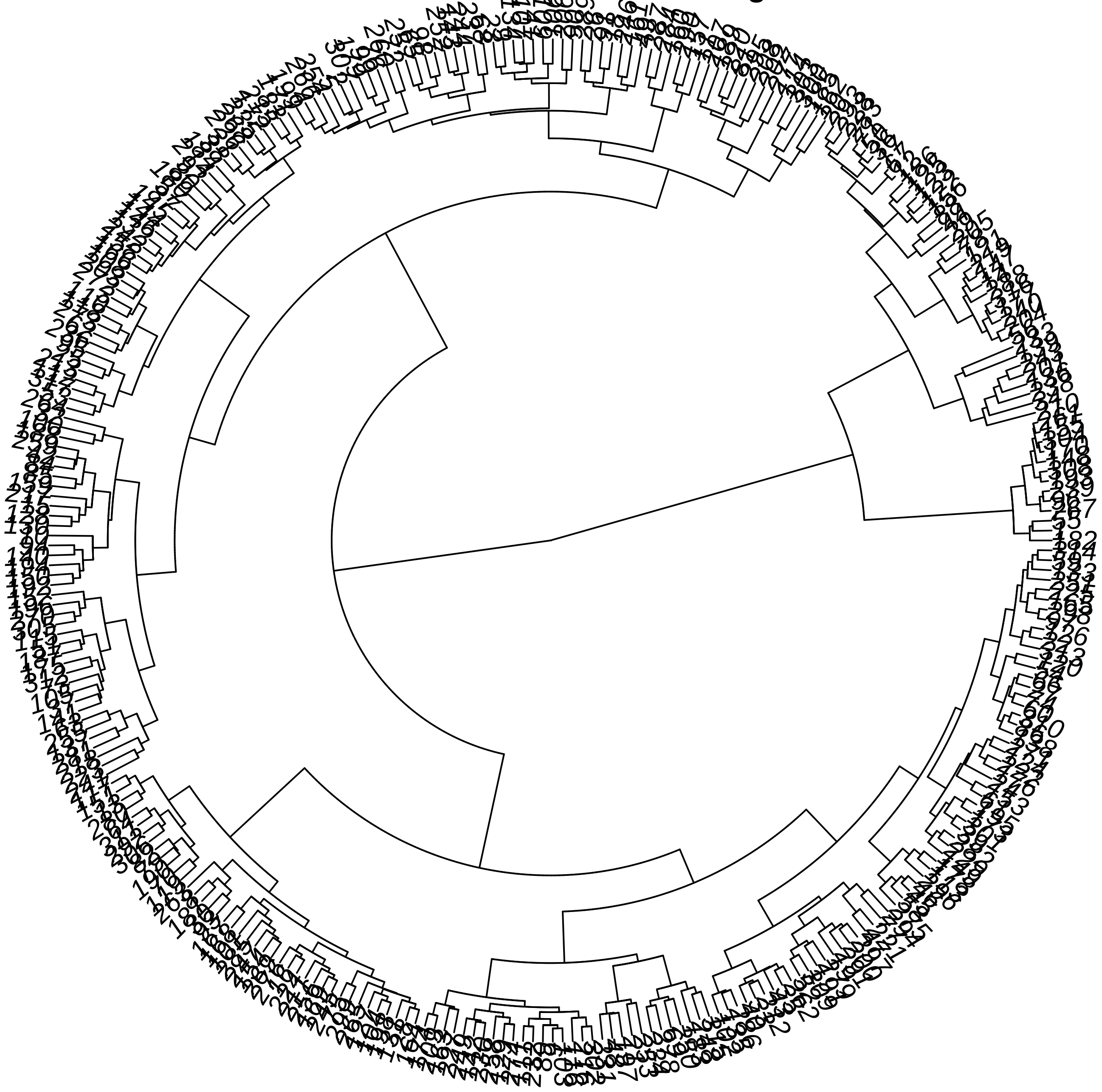

USA Area 5 at h = 15 : Cluster dendrogram

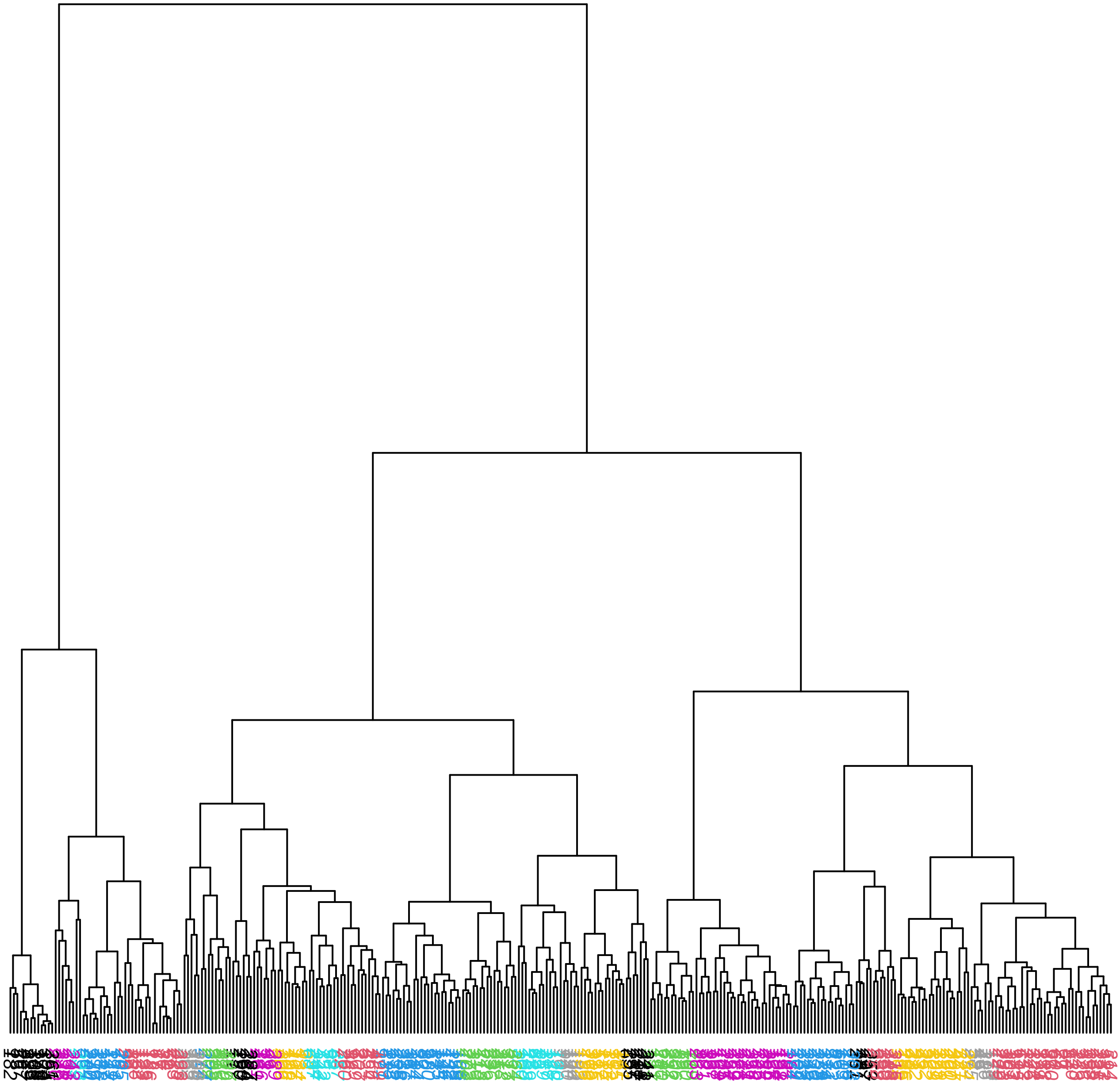

USA Area 5 at h = 15 : Coloured Unrooted Cluster dendrogram

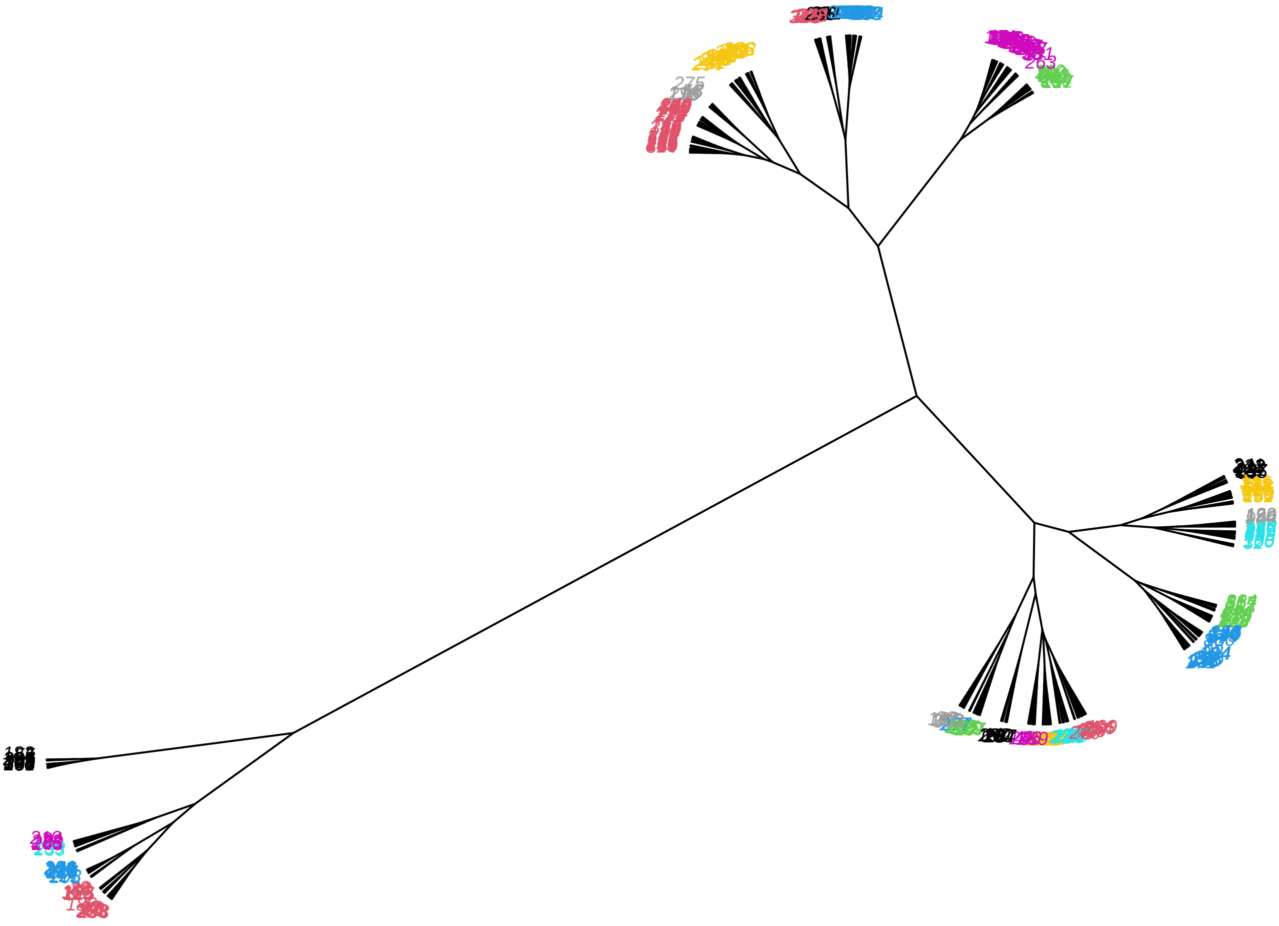

USA Area 5 at h = 15 : Coloured Fan Cluster dendrogram

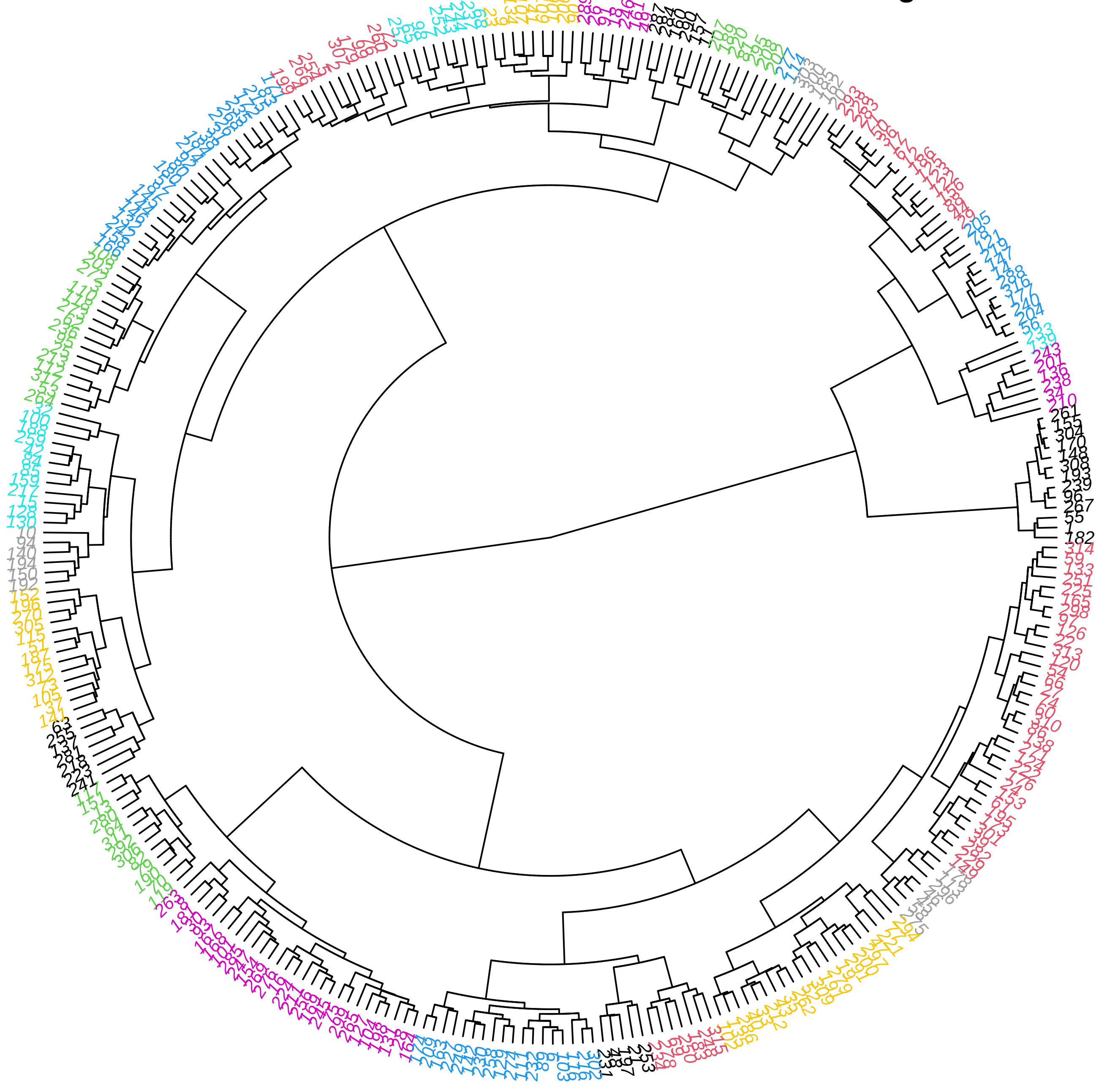

USA Area 6 with cluster c(20, 20, 50, 50, 50, 20, 50) Cluster dendrogram

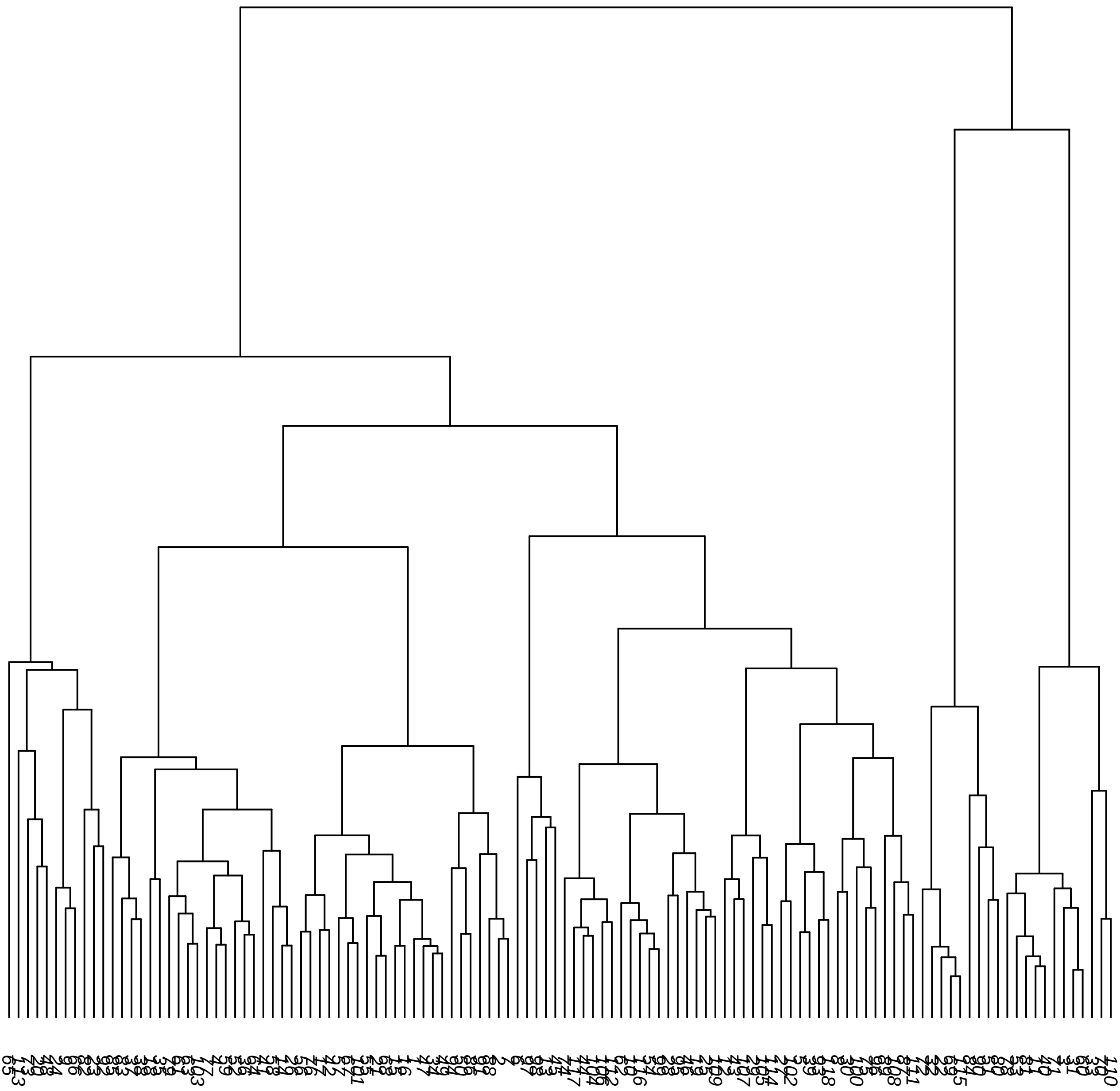

USA Area 6 Unrooted Cluster dendrogram

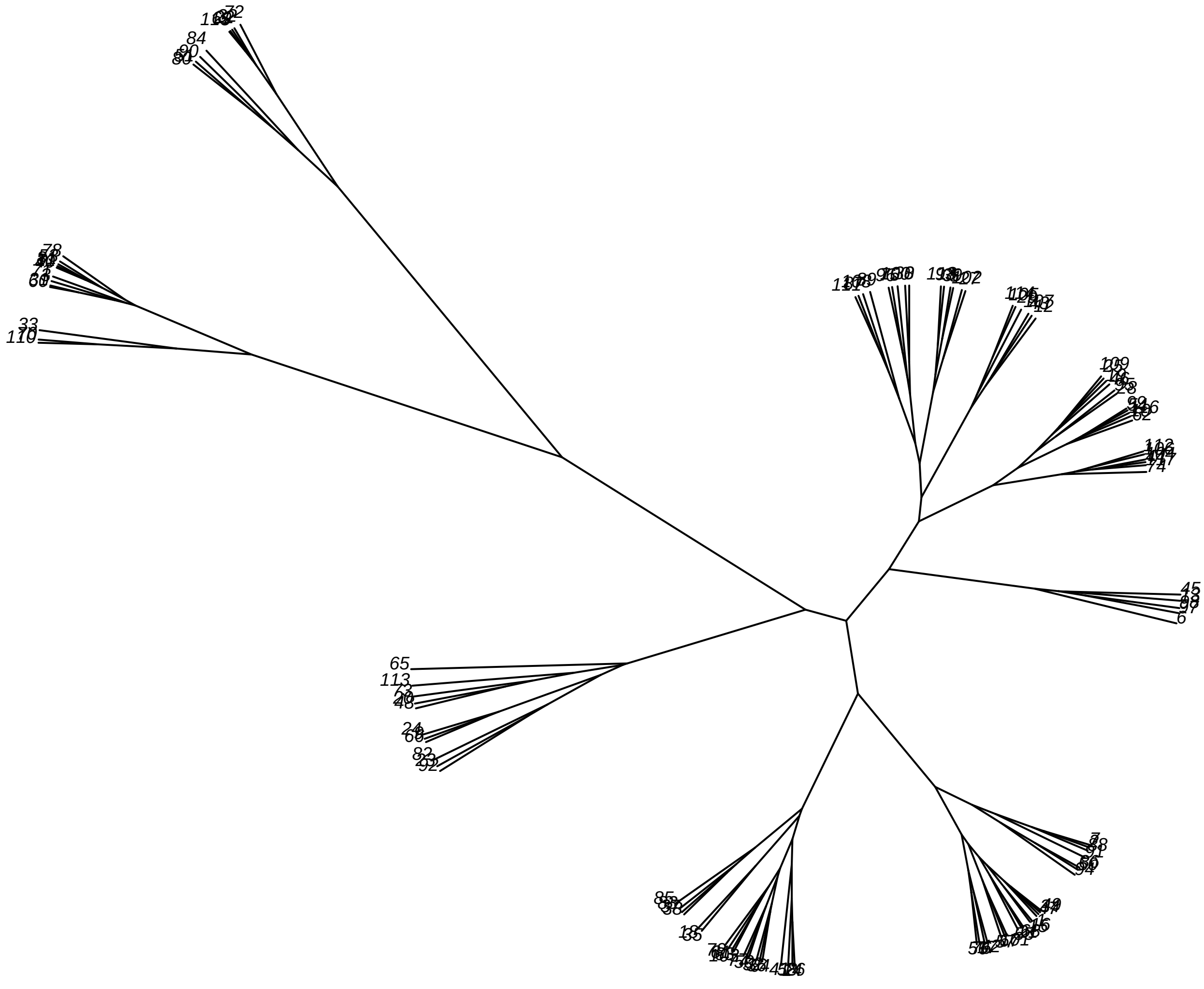

USA Area 6 Fan Cluster dendrogram

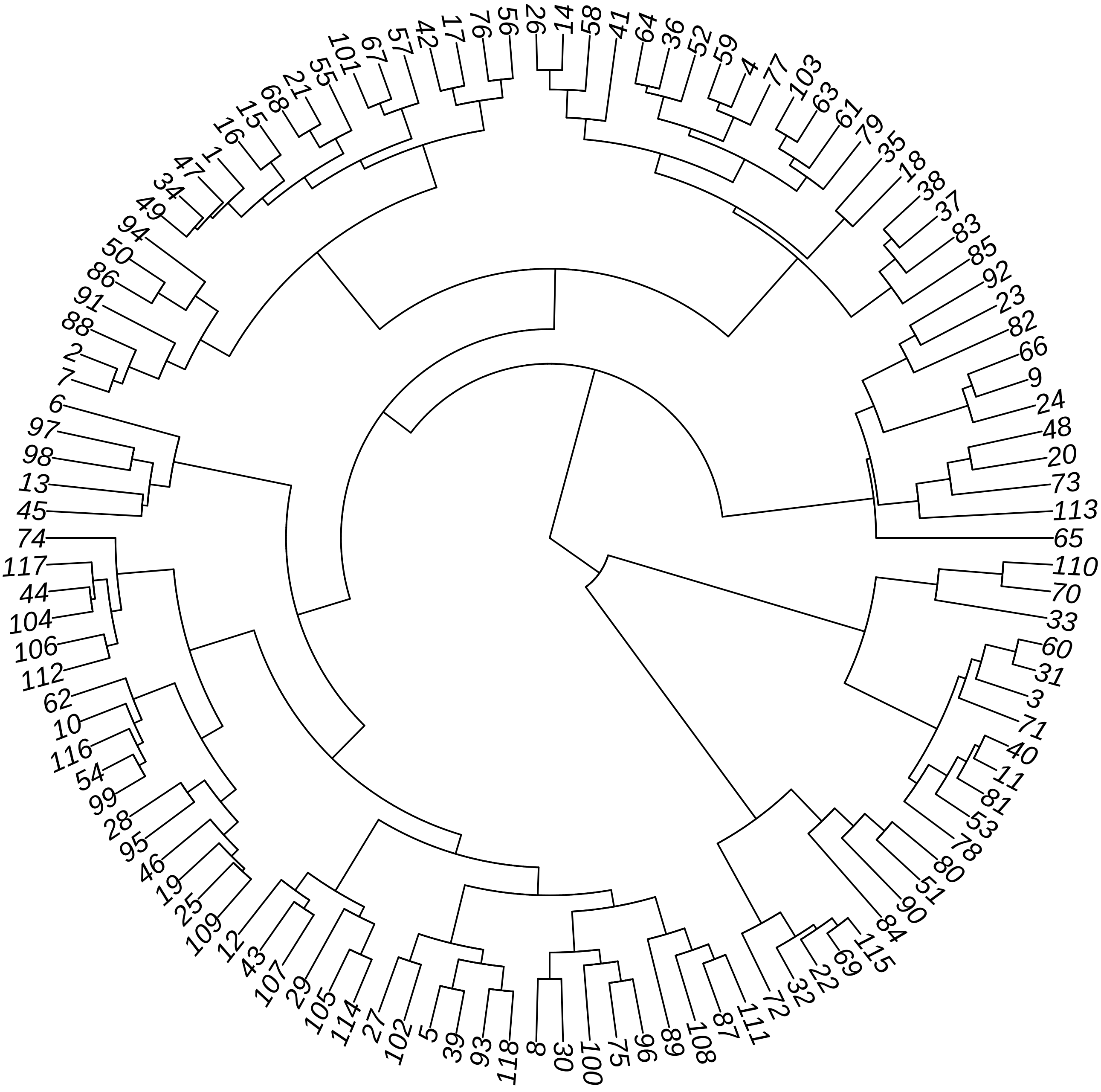

USA Area 6 at h = 15 : Cluster dendrogram

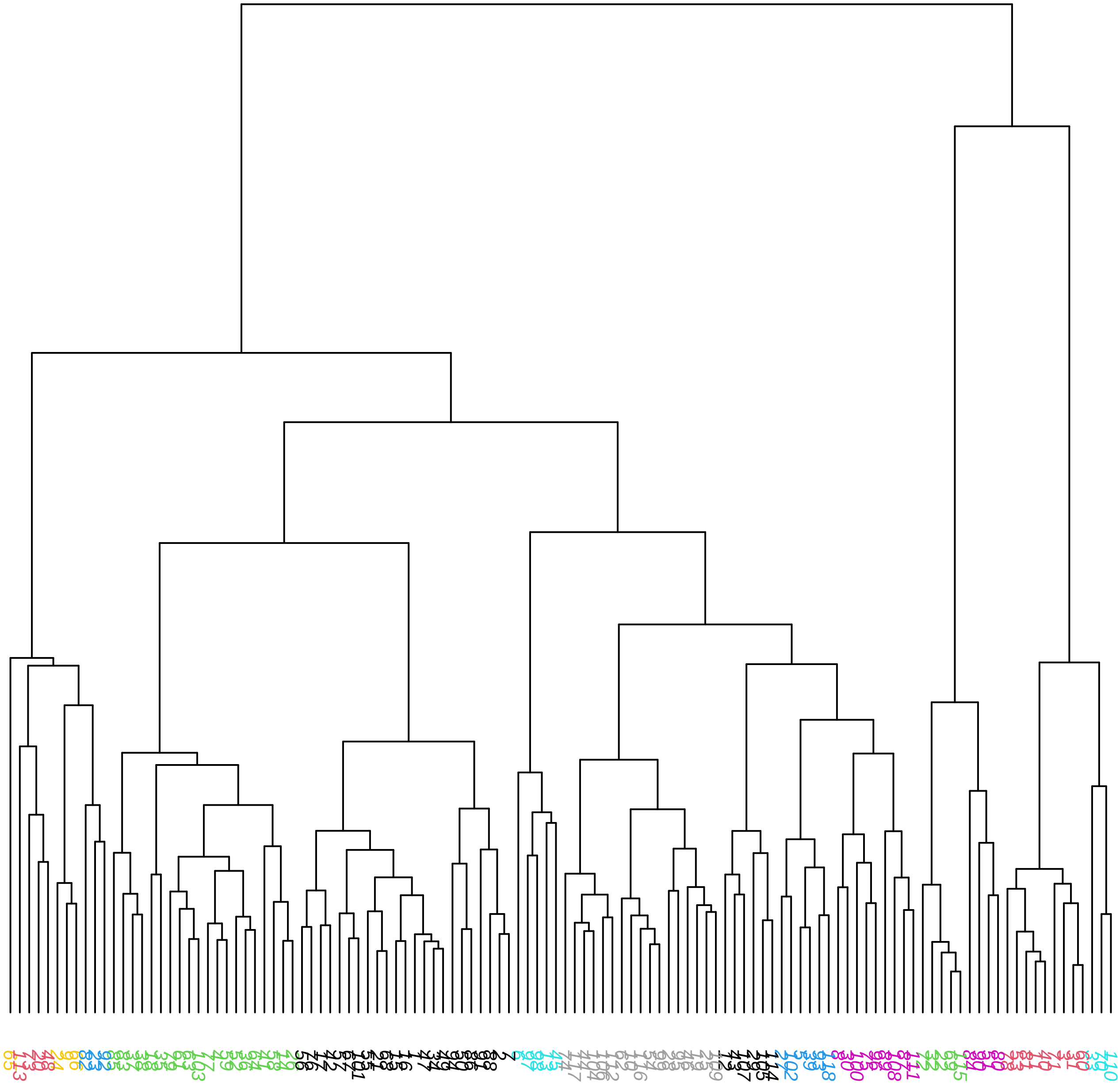

USA Area 6 at h = 15 : Coloured Unrooted Cluster dendrogram

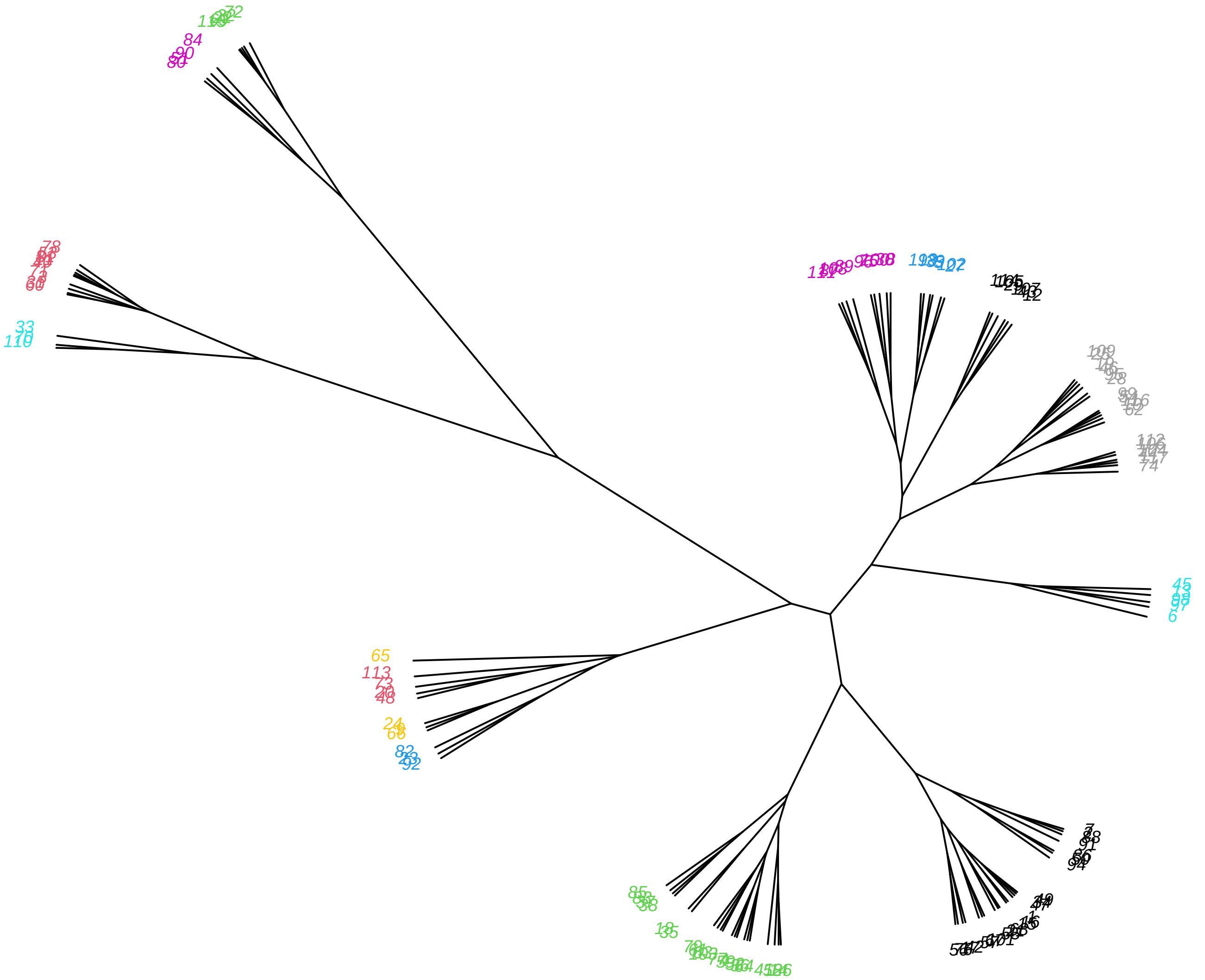

USA Area 6 at h = 15 : Coloured Fan Cluster dendrogram

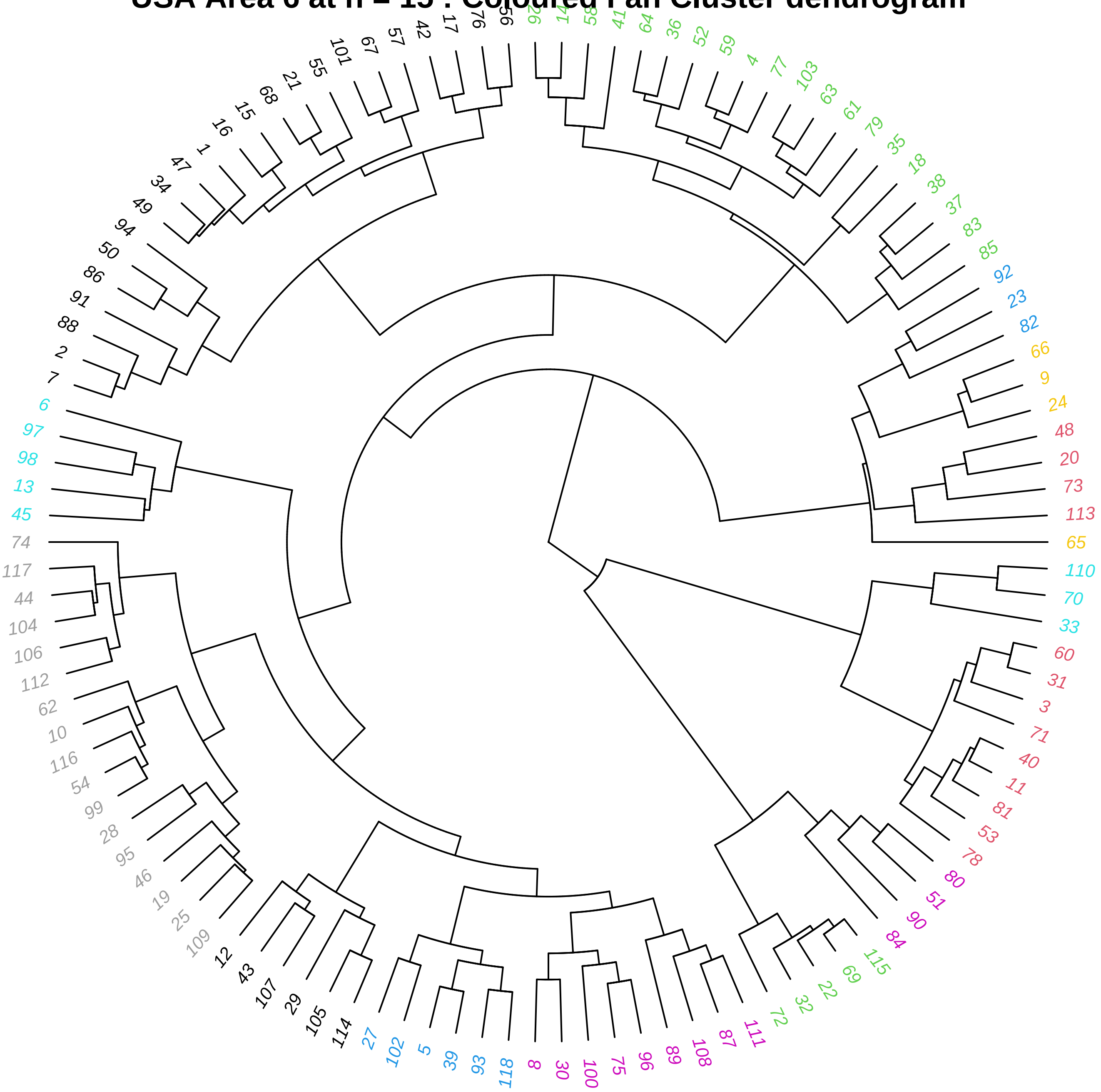

USA Area 7 with cluster c(20, 20, 50, 50, 50, 20, 50) Cluster dendrogram

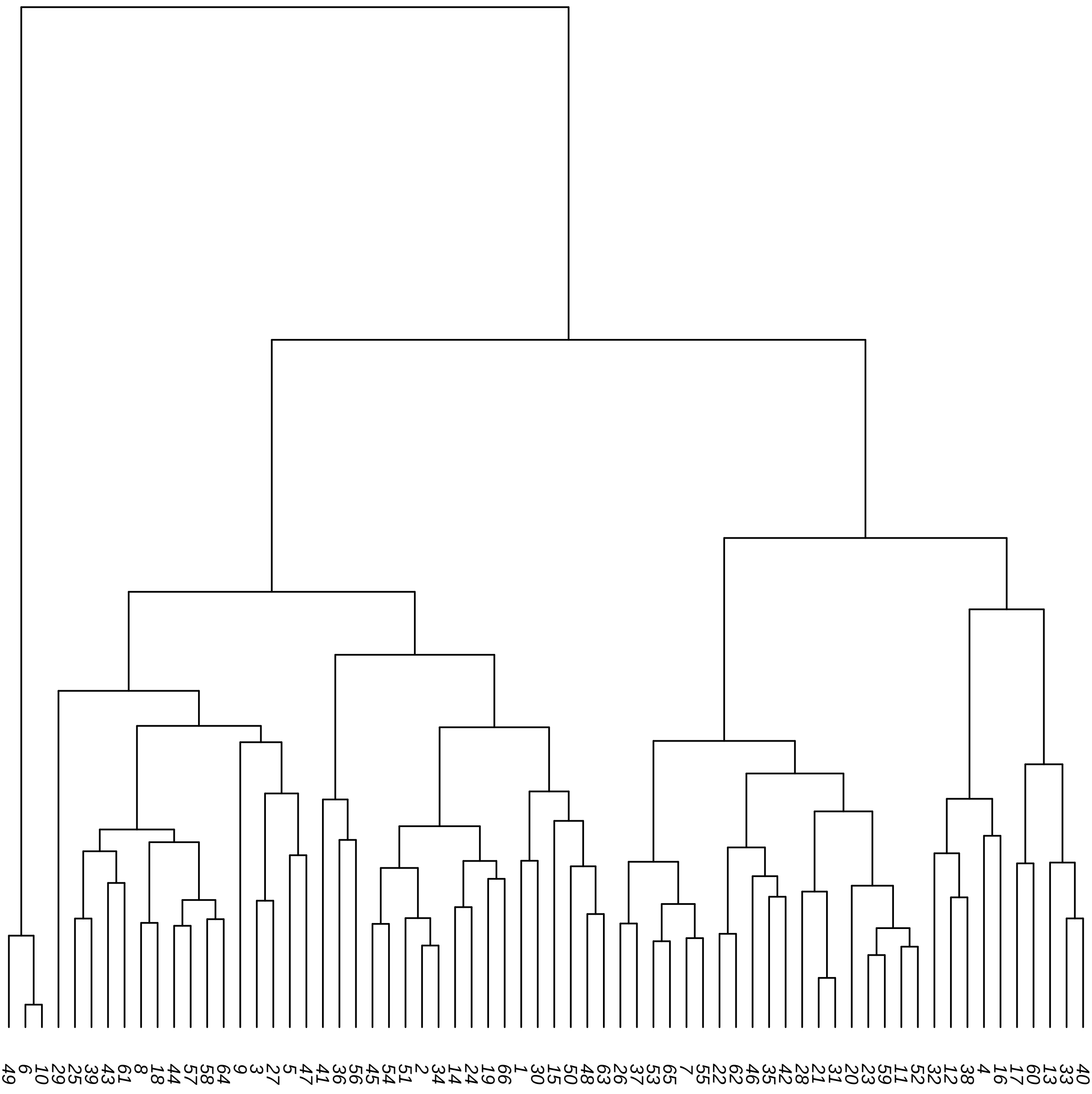

USA Area 7 Unrooted Cluster dendrogram

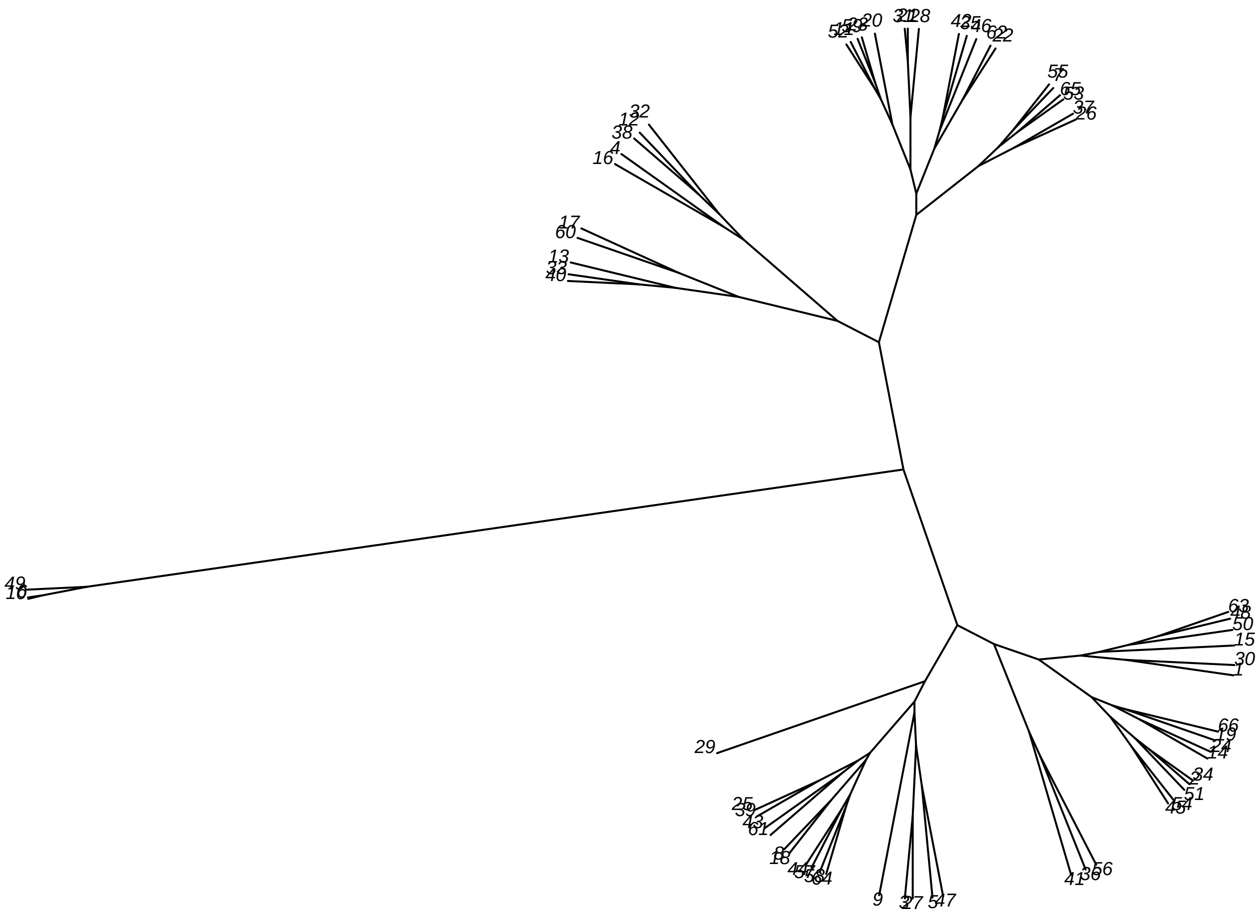

USA Area 7 Fan Cluster dendrogram

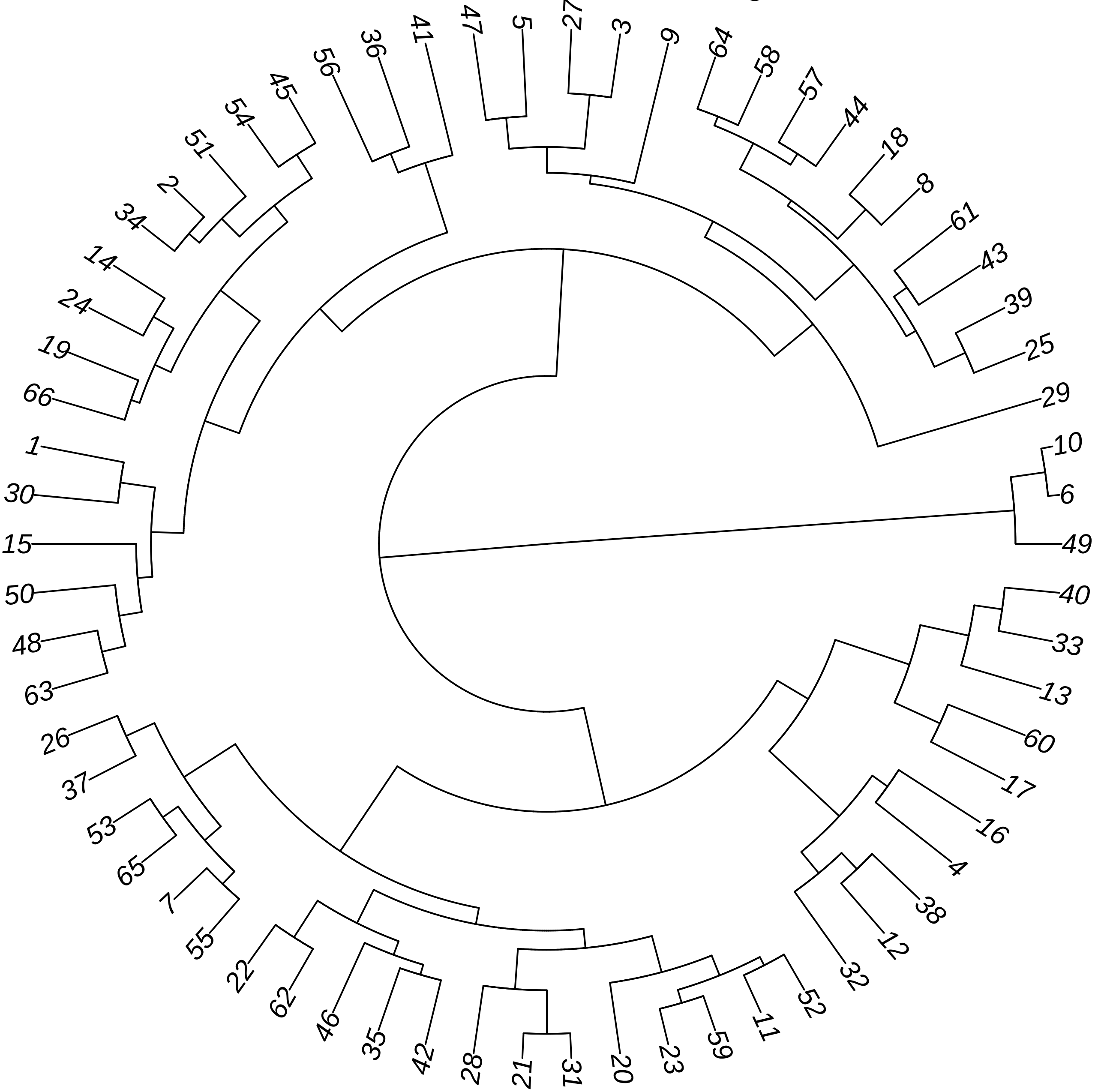

USA Area 7 at h = 15 : Cluster dendrogram

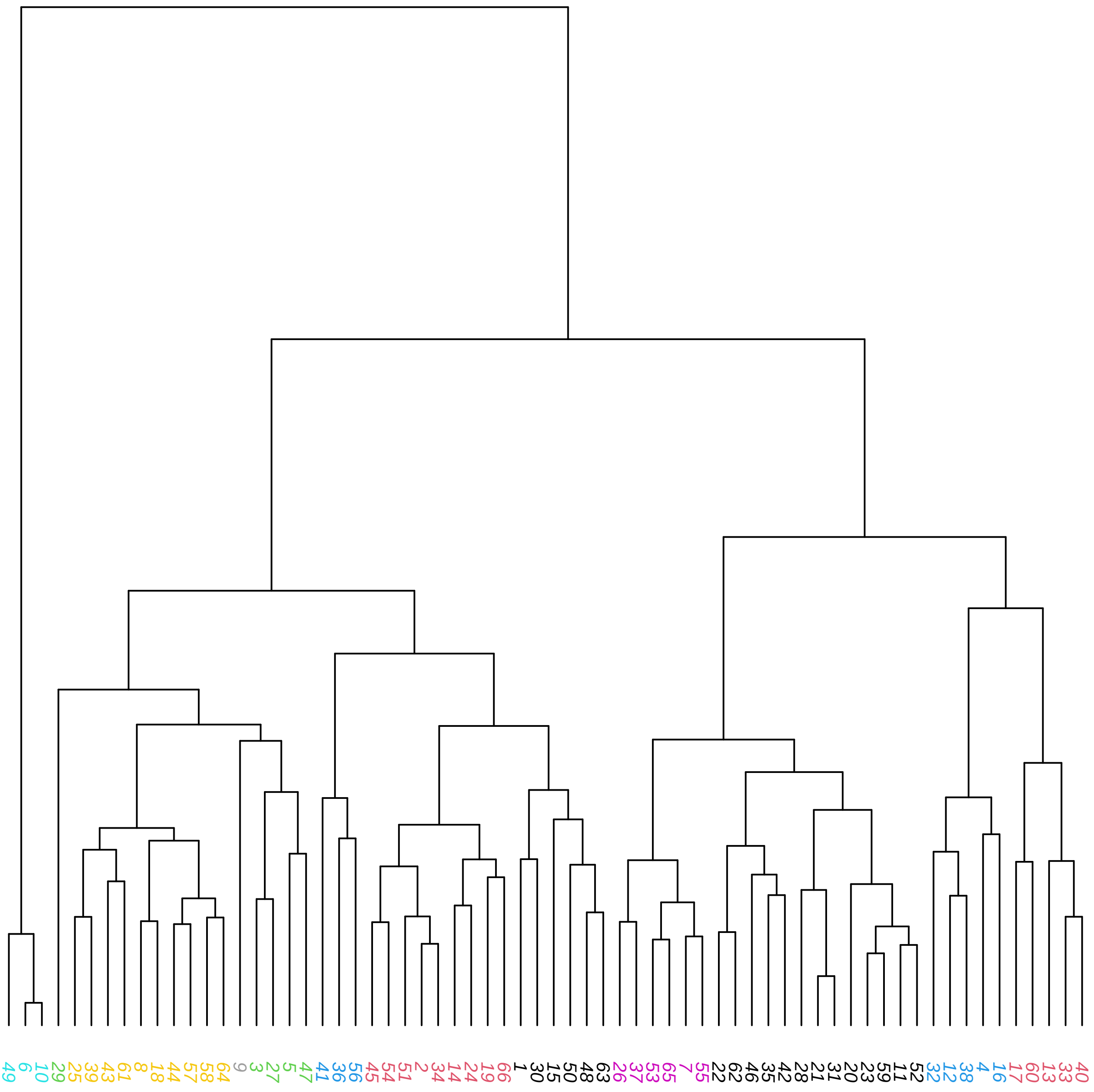

USA Area 7 at h = 15 : Coloured Unrooted Cluster dendrogram

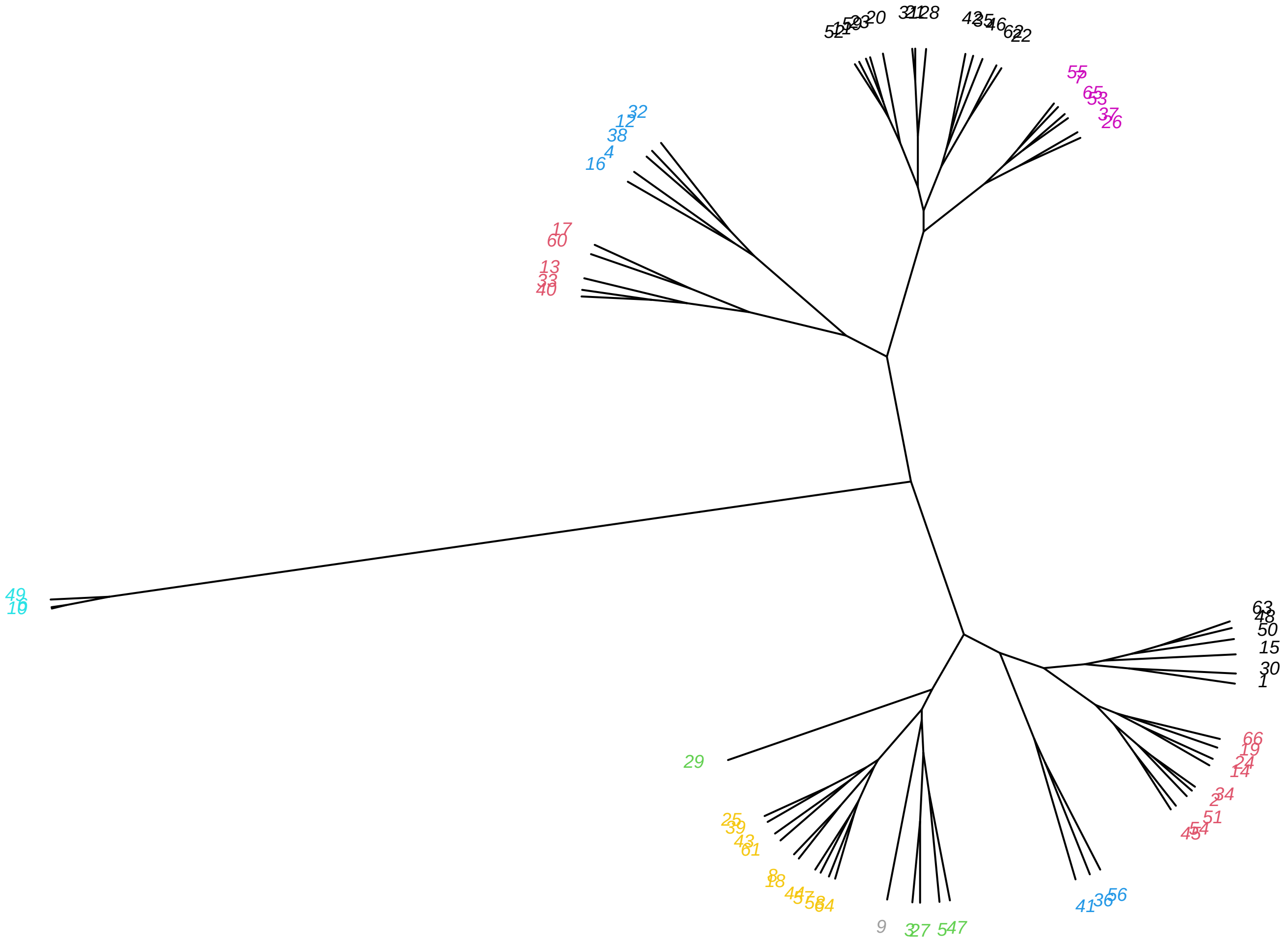

USA Area 7 at h = 15 : Coloured Fan Cluster dendrogram

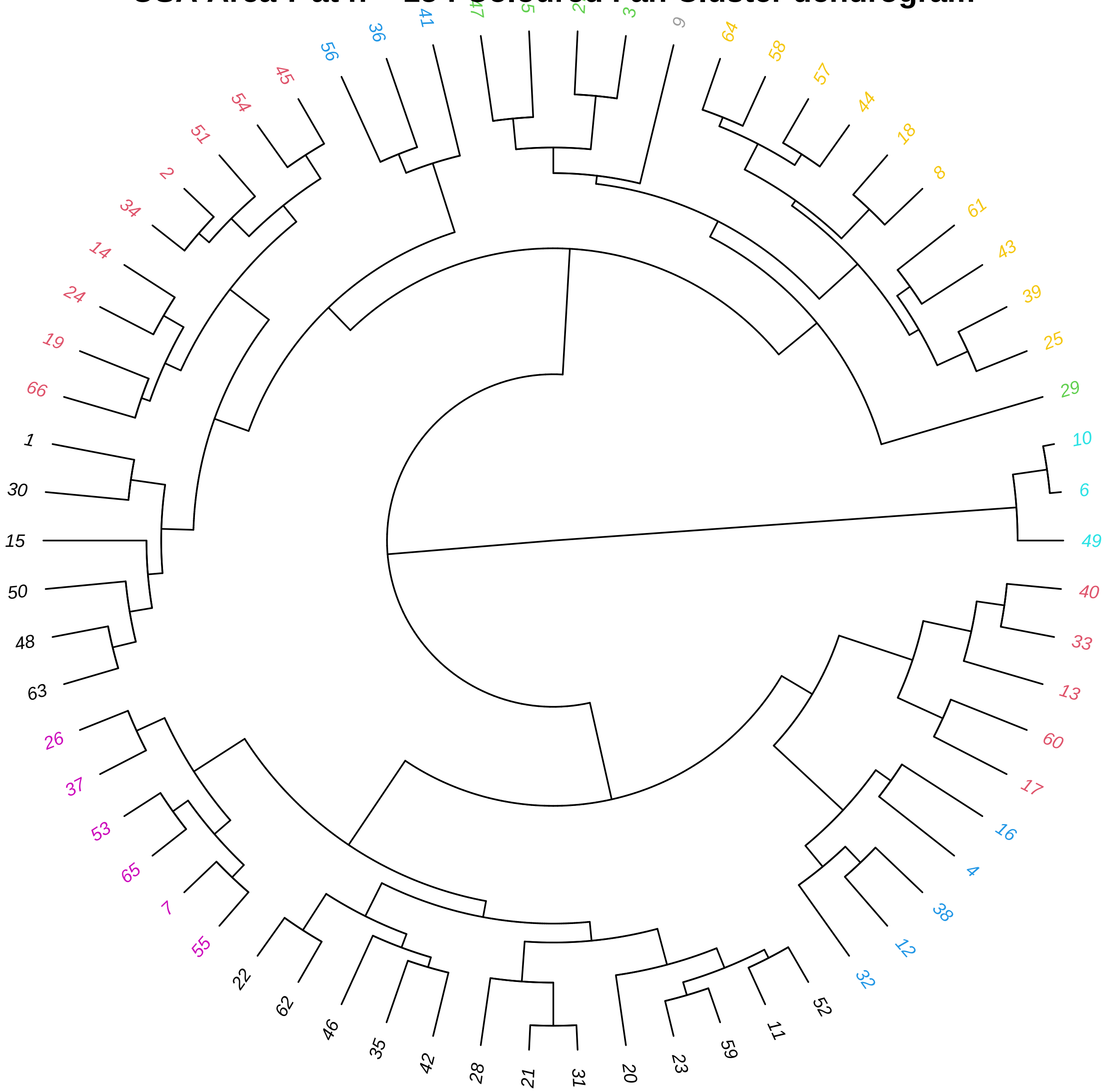

Supplement: S9 Appendix — (PDF) [file pone.0272848.s009.pdf]
