# Supplementary material for: Classifying ball trajectories in invasion sports using dynamic time warping: A basketball case study
Source: PLoS One. 2022 Oct 20;17(10):e0272848. doi: 10.1371/journal.pone.0272848 (PMC9584368; doi:10.1371/journal.pone.0272848)

USA Area 1 Cluster 1 : SelectTrajectories

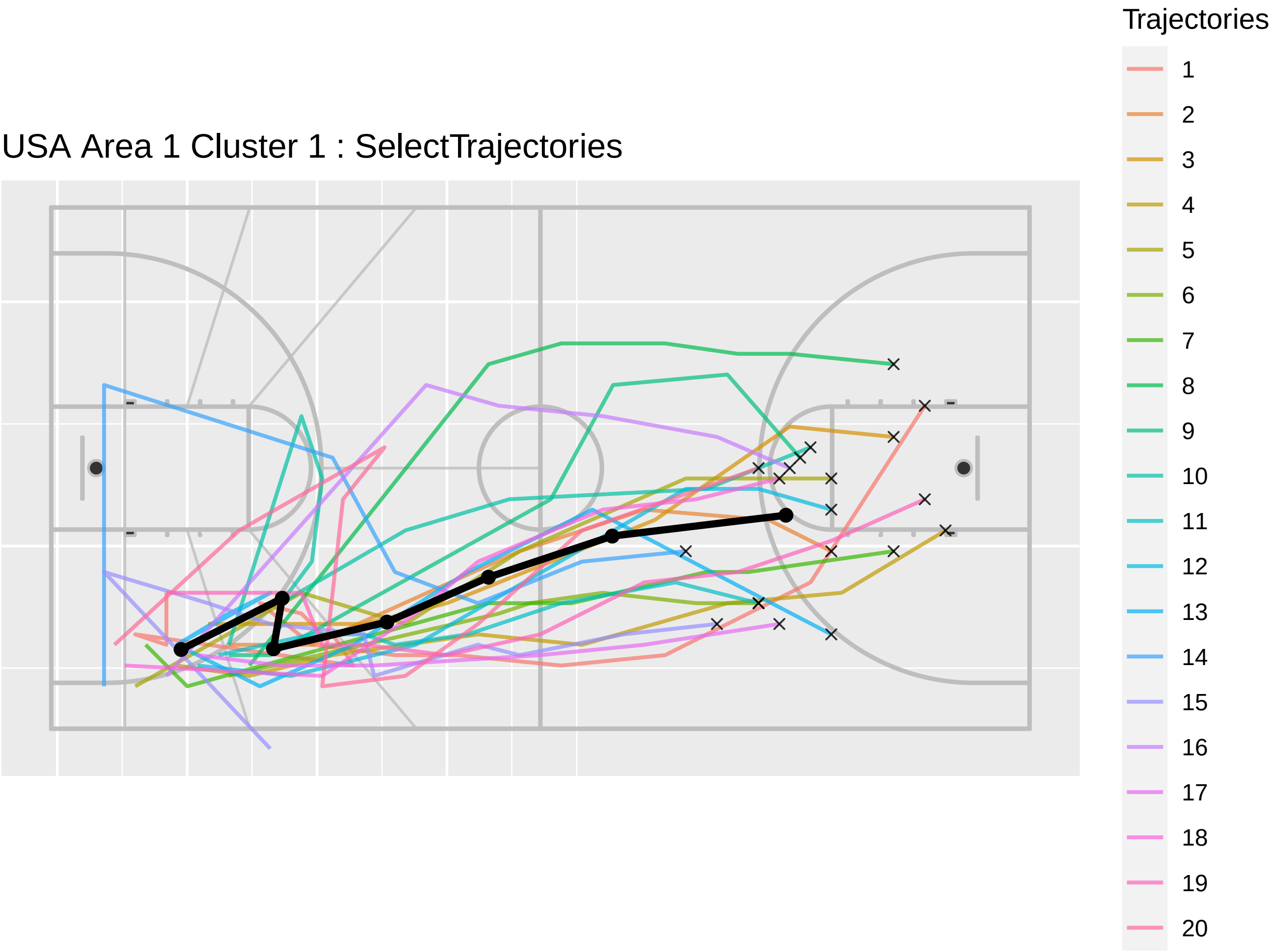

USA Area 1 Cluster 2 : SelectTrajectories

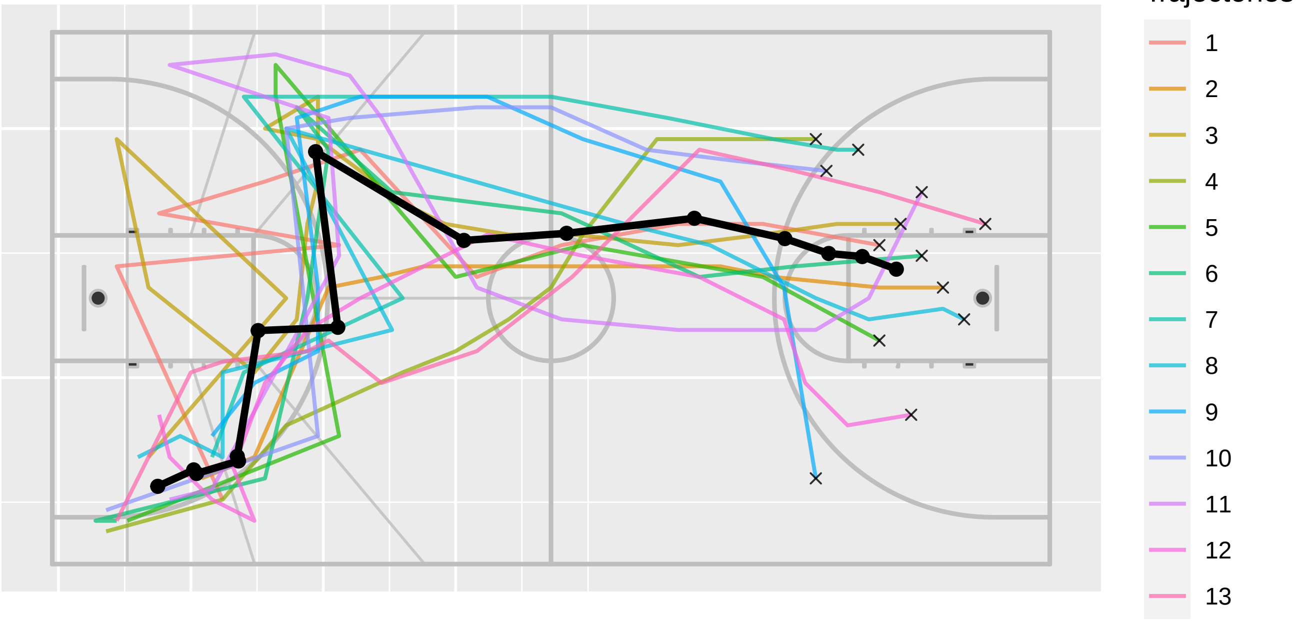

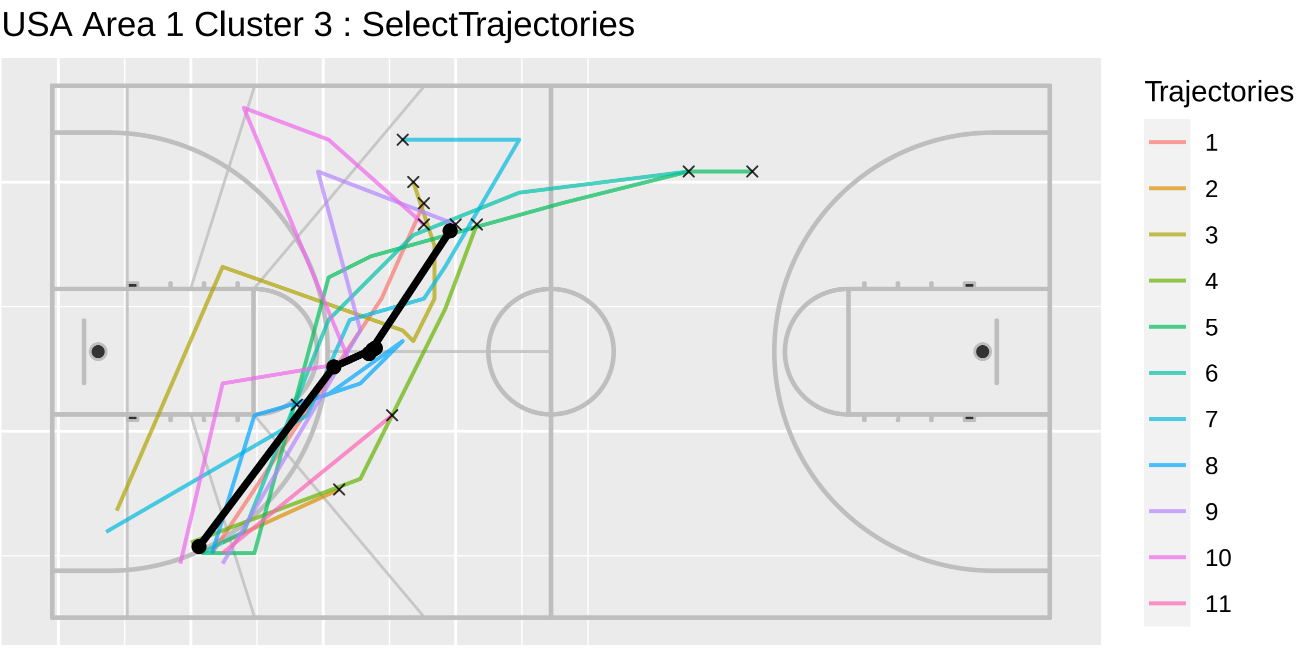
USA Area 1 Cluster 3 : SelectTrajectories
Trajectories
1
2
3
4
5
6
7
8
9
10
11

USA Area 1 Cluster 4 : SelectTrajectories

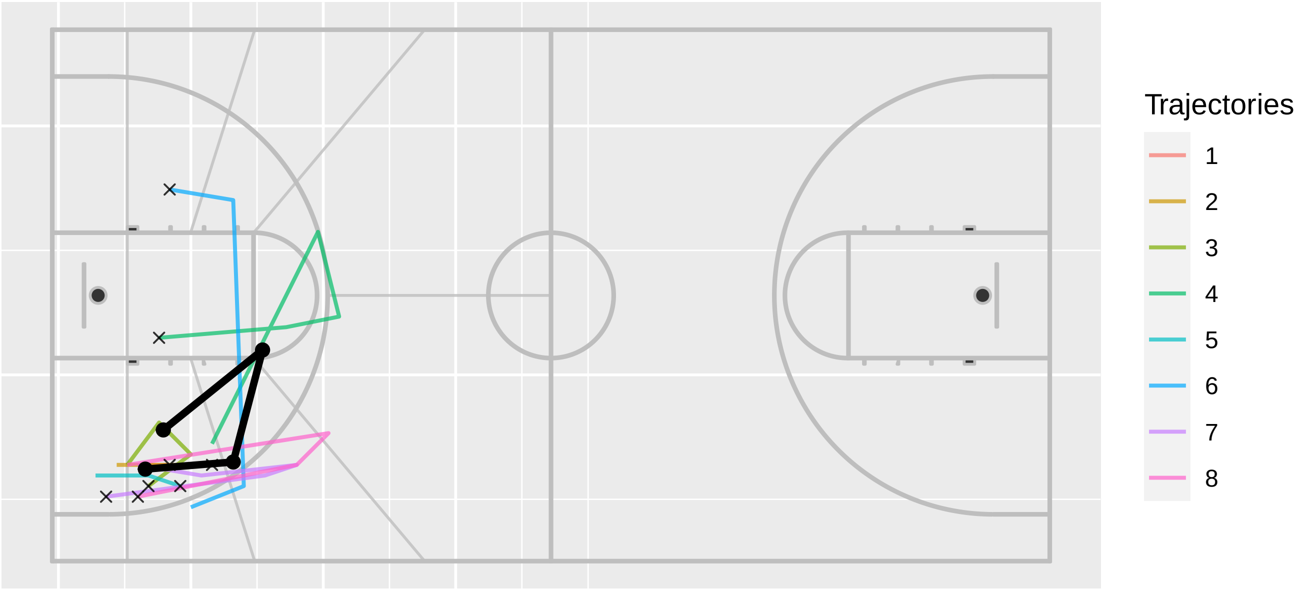

## USA Area 1 Cluster 5 : SelectTrajectories

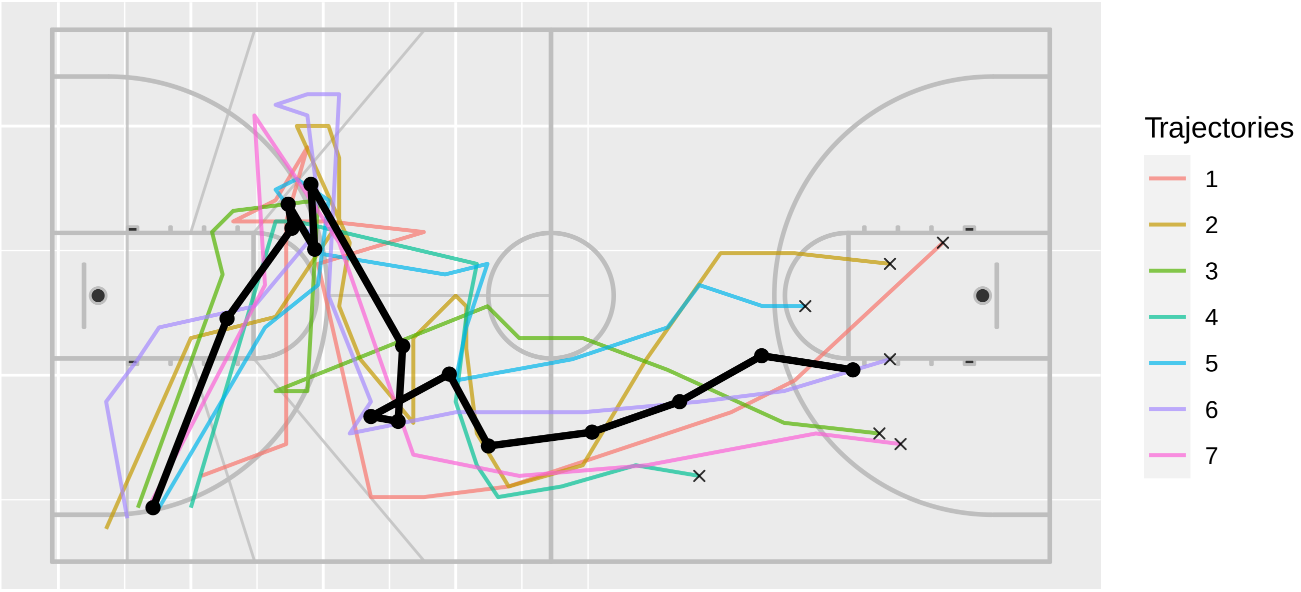

USA Area 1 Cluster 6 : SelectTrajectories

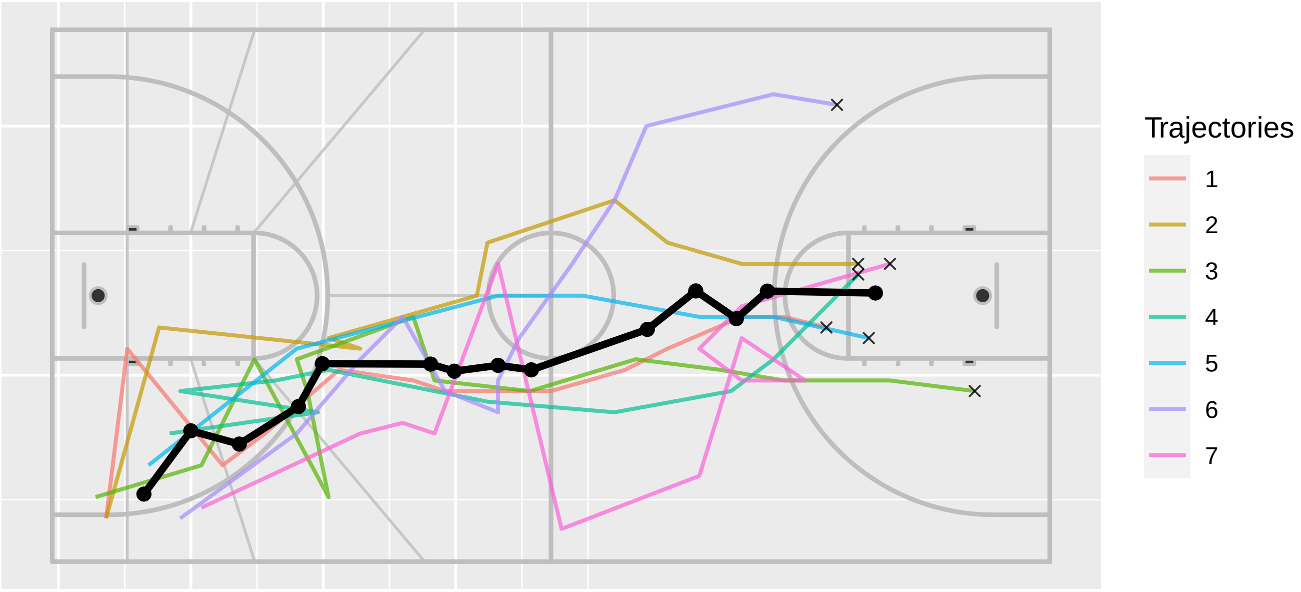

USA Area 1 Cluster 7 : SelectTrajectories

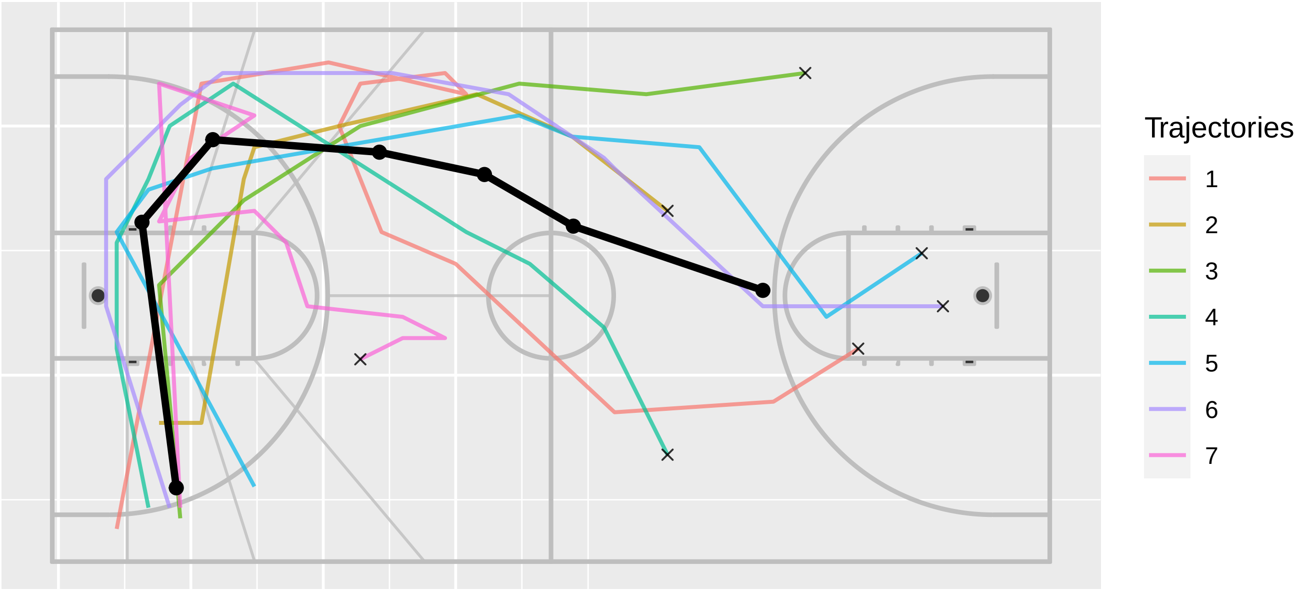

USA Area 1 Cluster 8 : SelectTrajectories

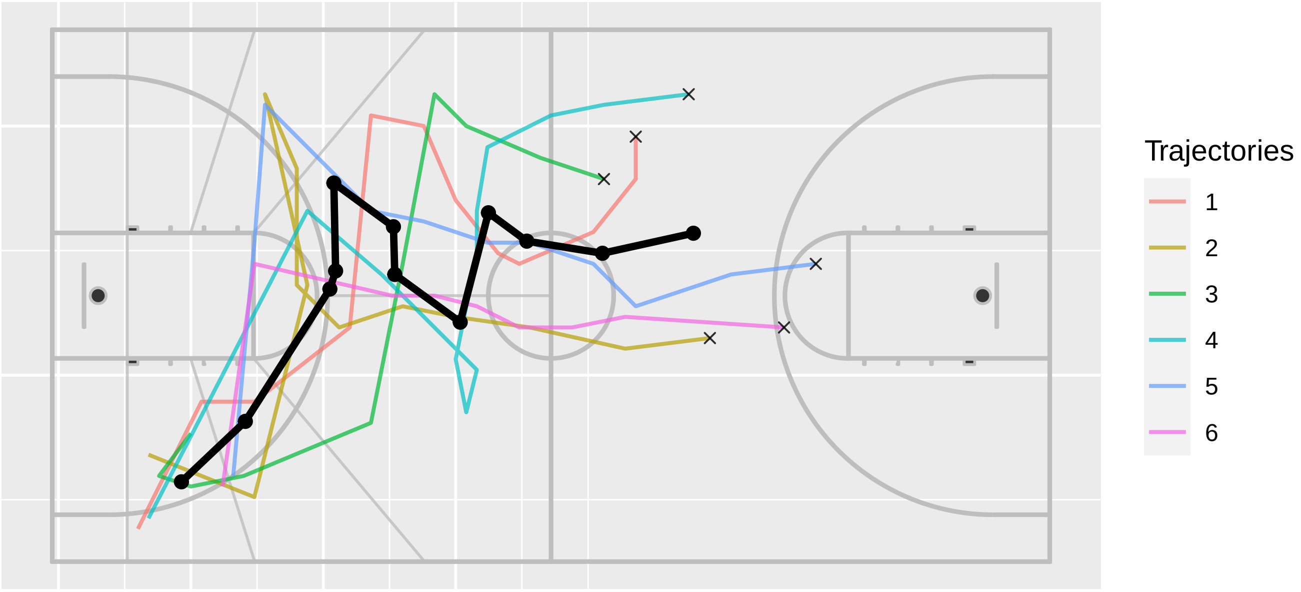

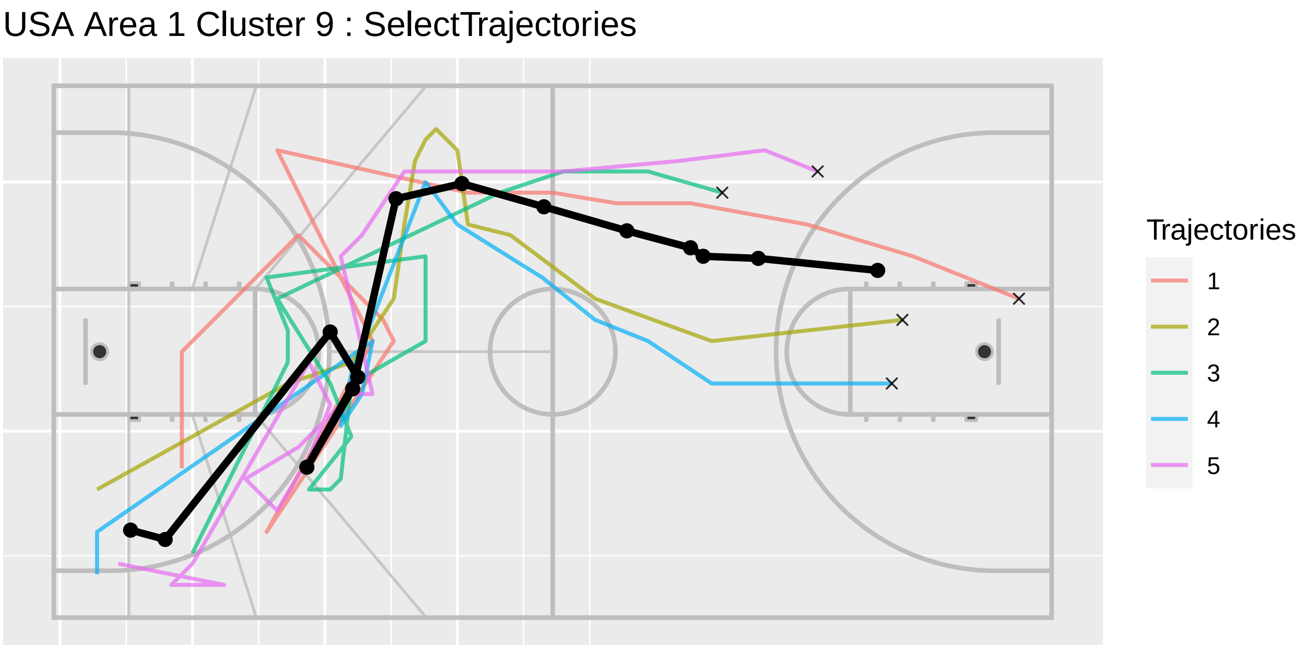
USA Area 1 Cluster 9 : SelectTrajectories
Trajectories
1
2
3
4
5

## USA Area 1 Cluster 10 : SelectTrajectories

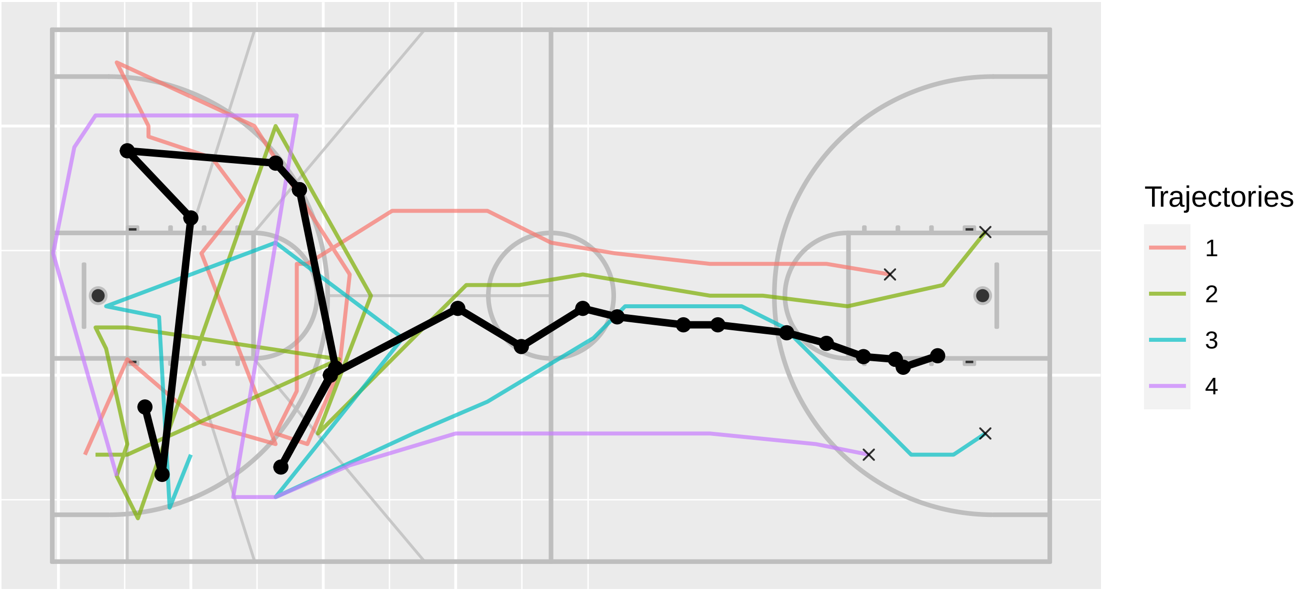

USA Area 1 Cluster 11 : SelectTrajectories

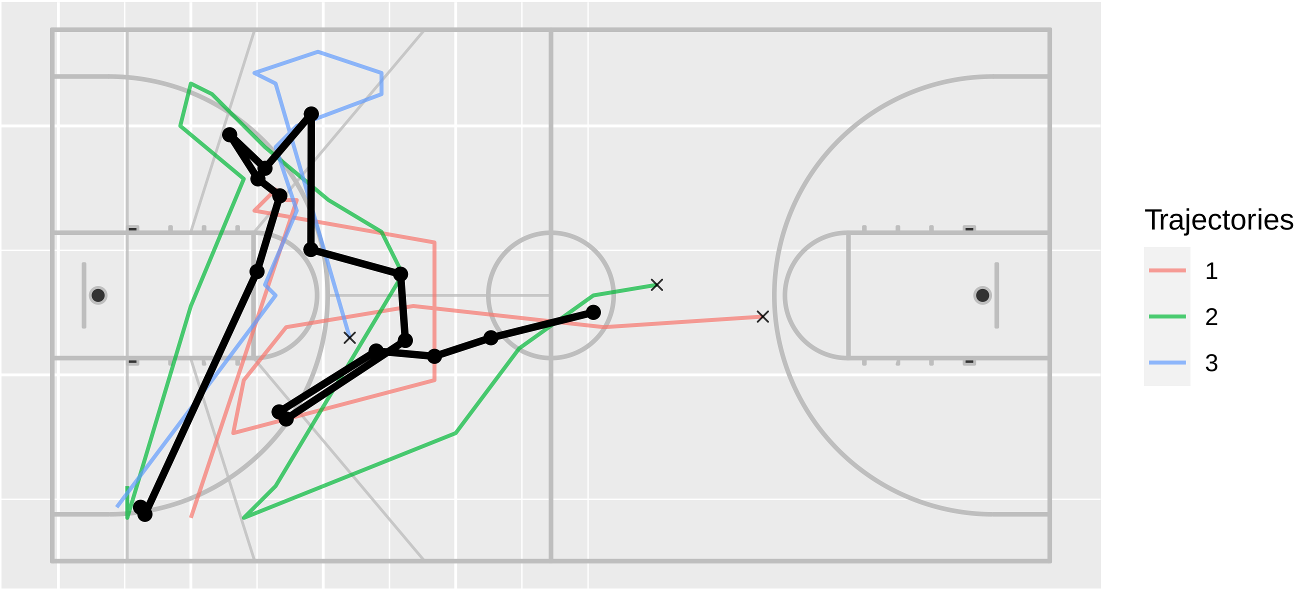

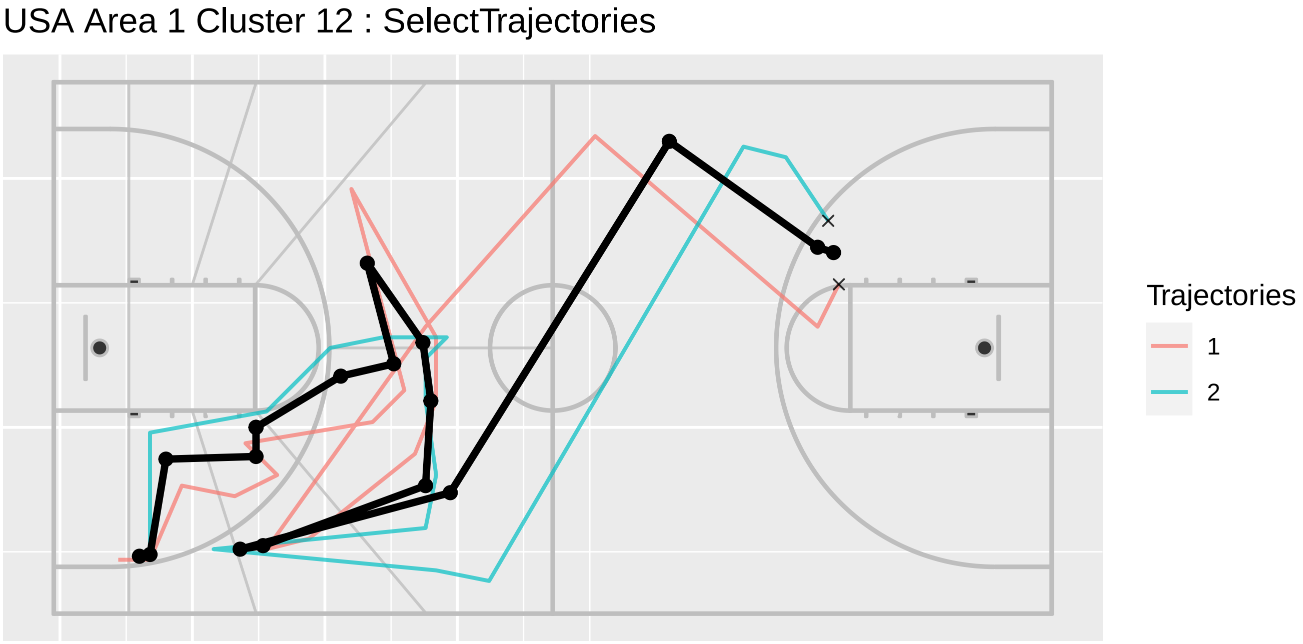
USA Area 1 Cluster 12 : SelectTrajectories
Trajectories
1
2

USA Area 1 Cluster 13 : SelectTrajectories

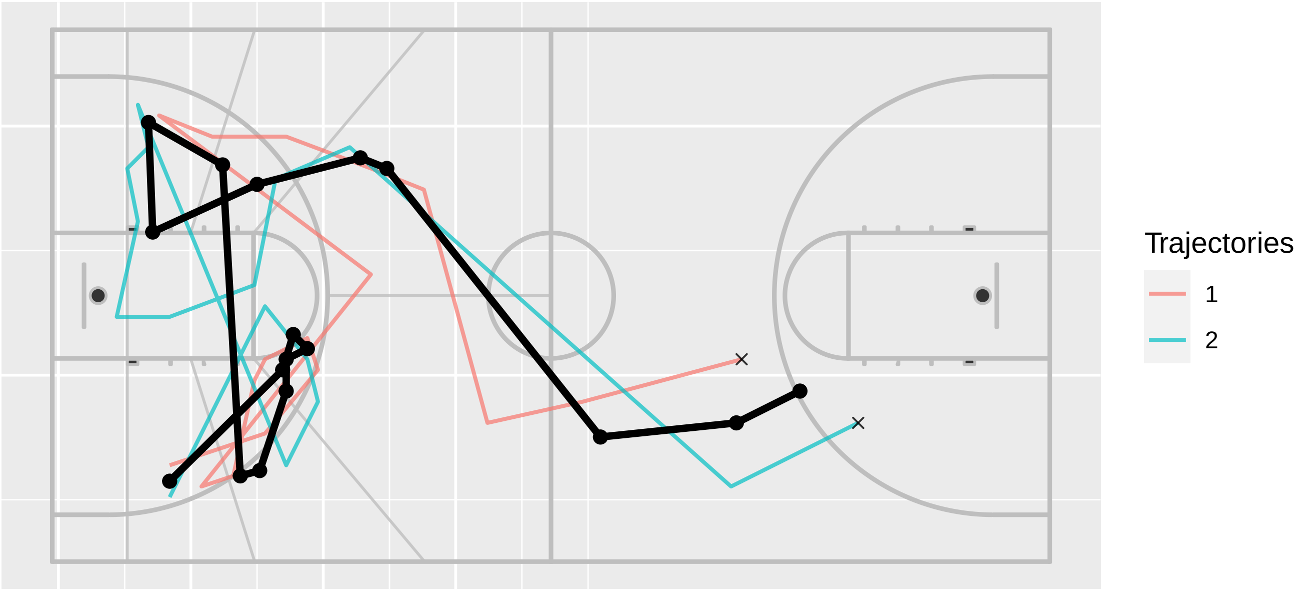

USA Area 2 Cluster 1 : SelectTrajectories

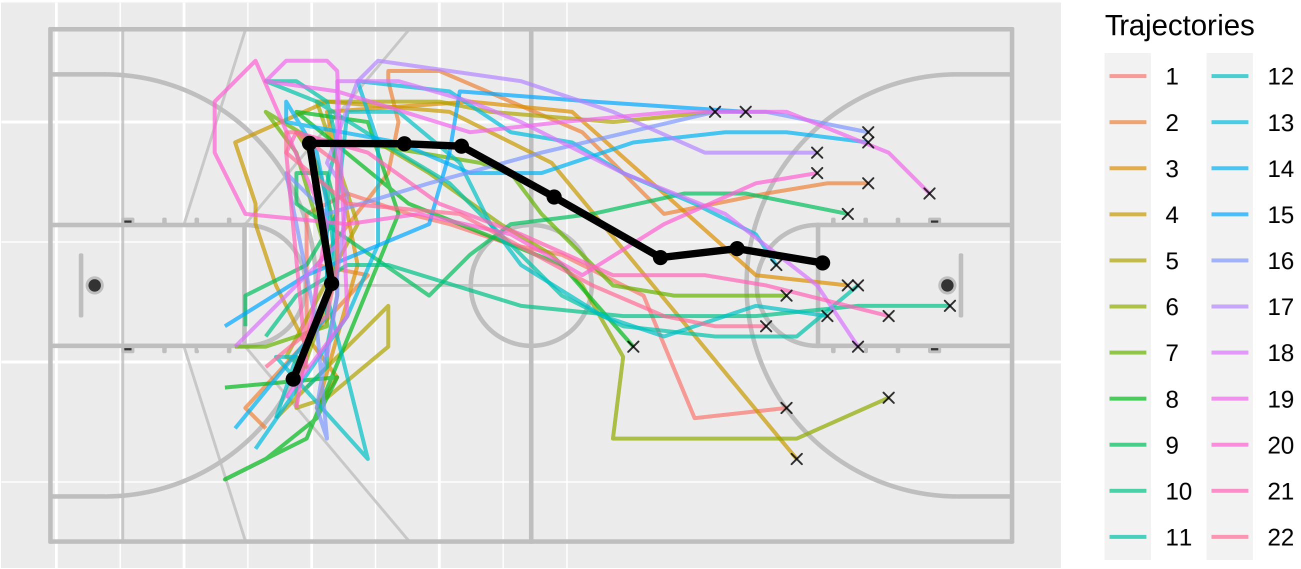

USA Area 2 Cluster 2 : SelectTrajectories

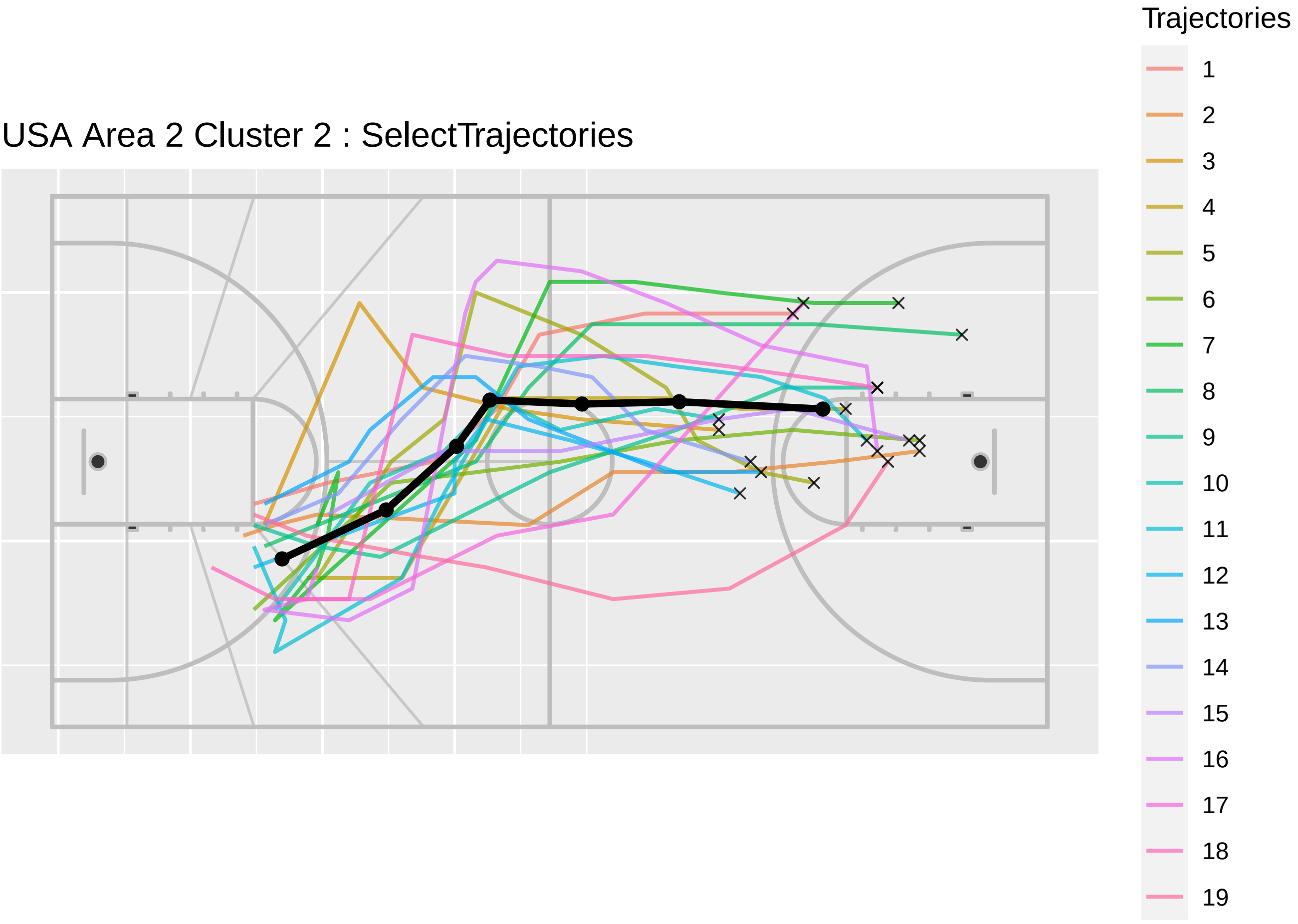

USA Area 2 Cluster 3 : SelectTrajectories

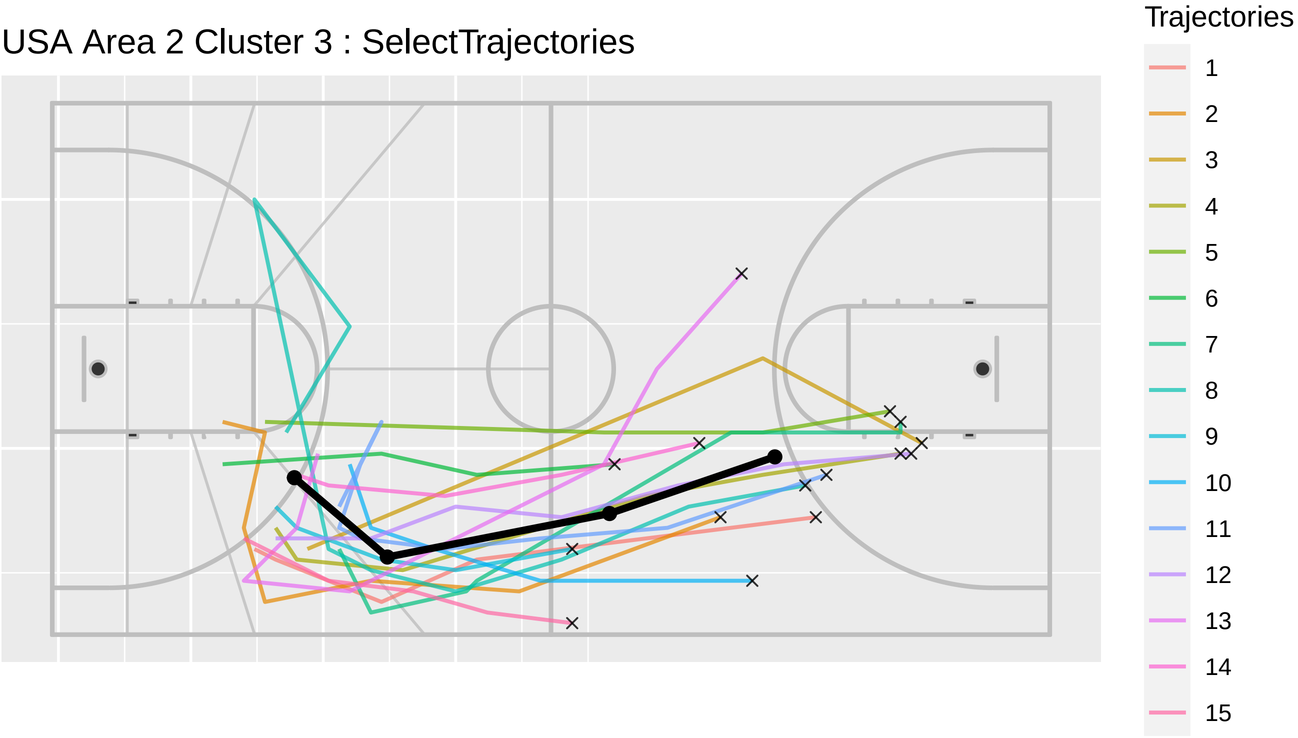

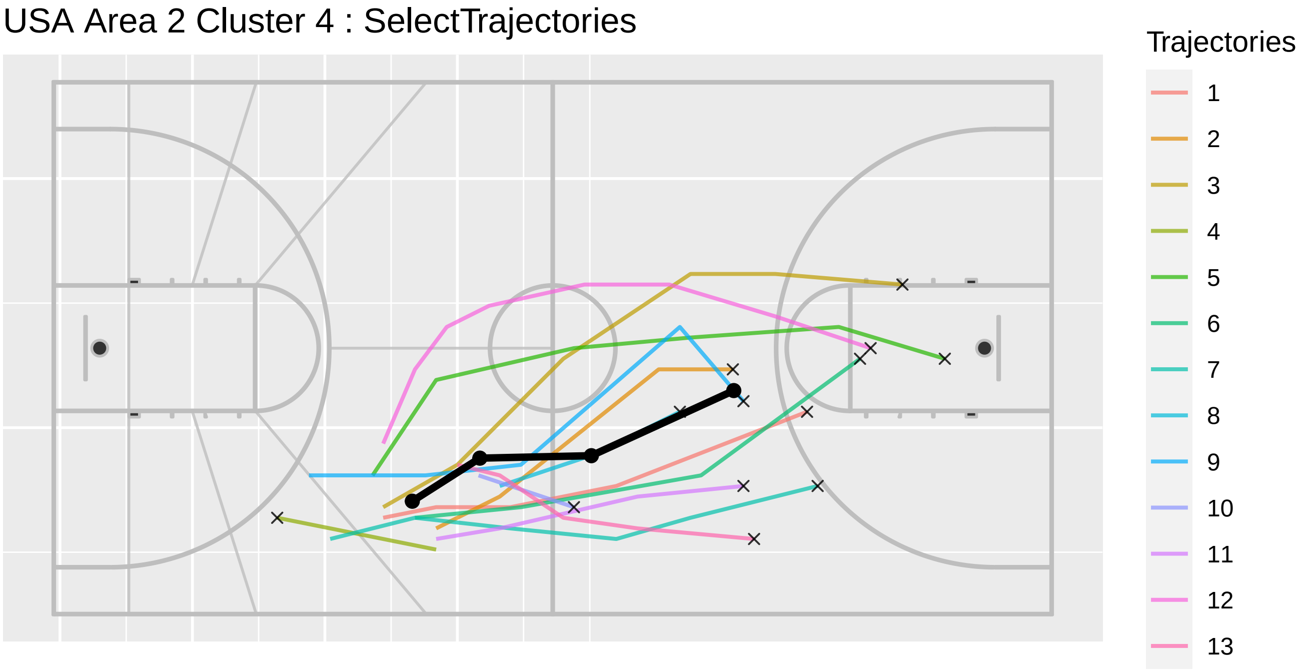
USA Area 2 Cluster 4 : SelectTrajectories
Trajectories
1
2
3
4
5
6
7
8
9
10
11
12
13

USA Area 2 Cluster 5 : SelectTrajectories

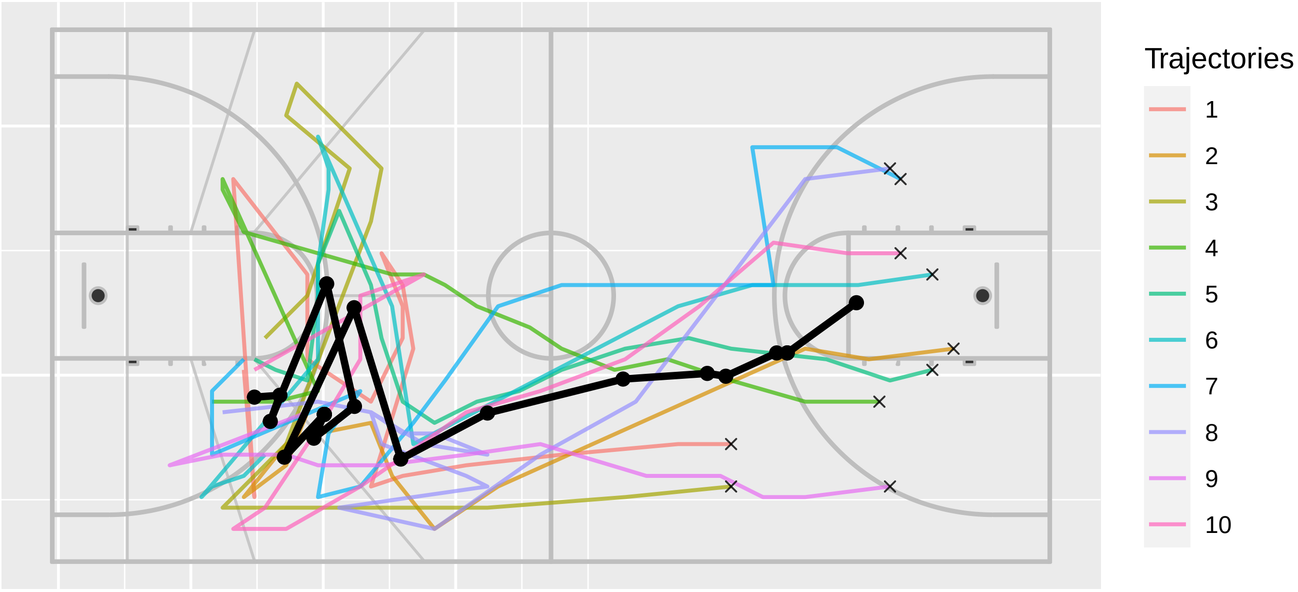

USA Area 2 Cluster 6 : SelectTrajectories

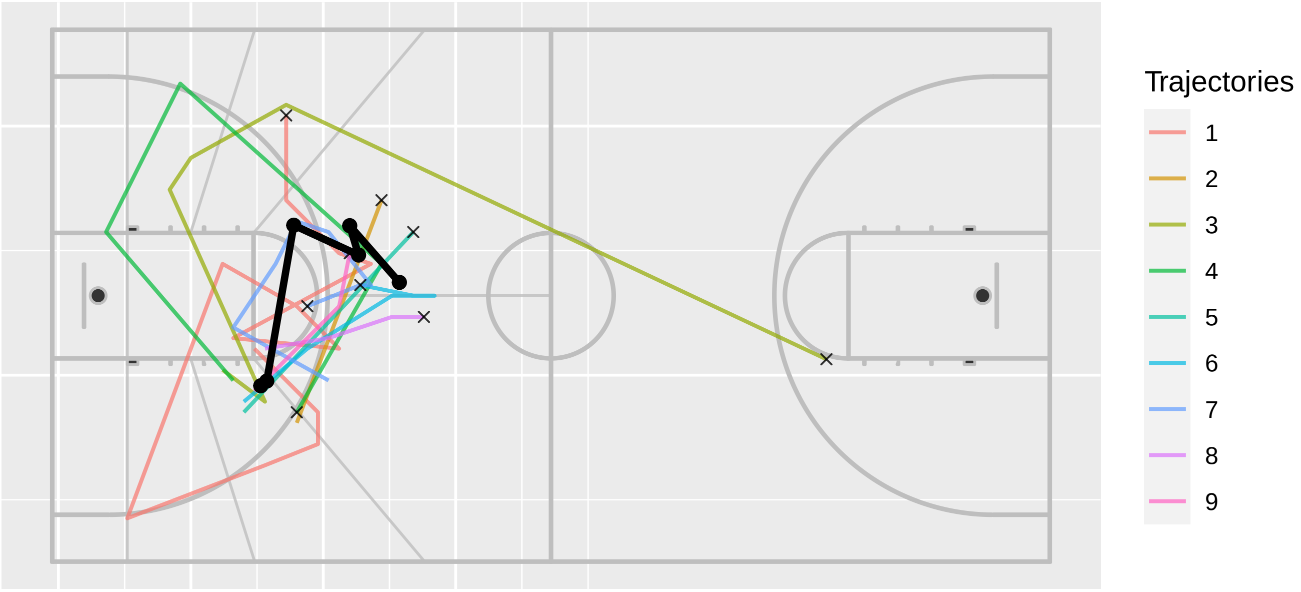

USA Area 2 Cluster 7 : SelectTrajectories

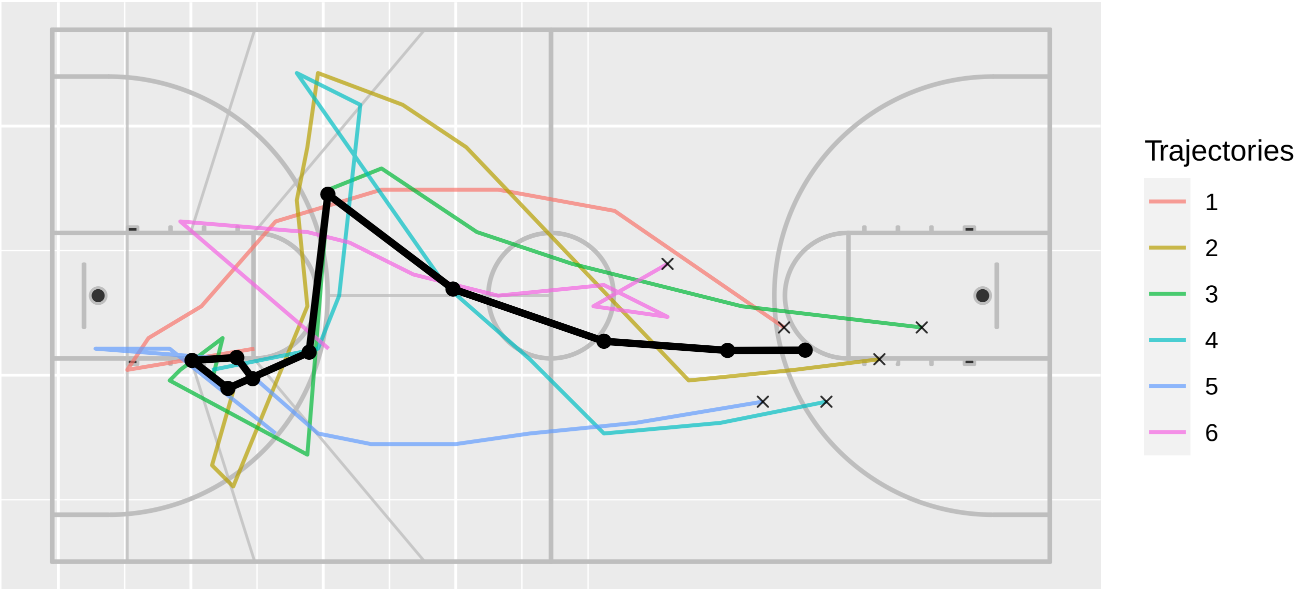

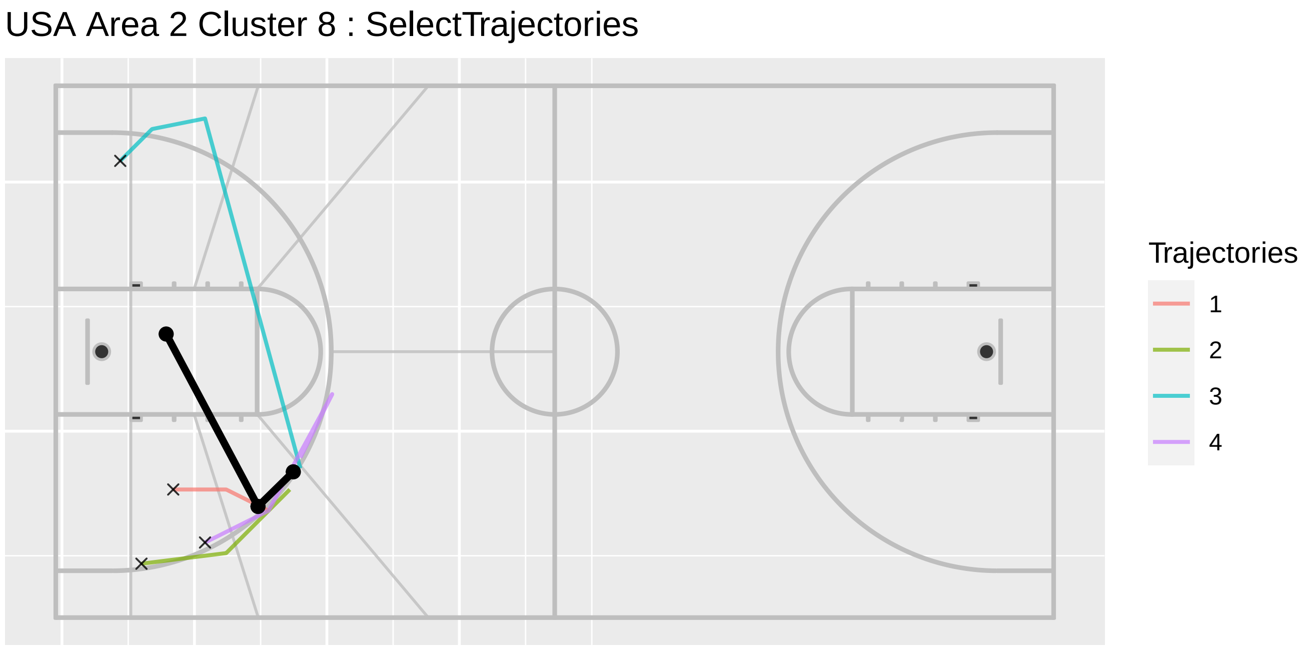
USA Area 2 Cluster 8 : SelectTrajectories
Trajectories
1
2
3
4

USA Area 2 Cluster 9 : SelectTrajectories

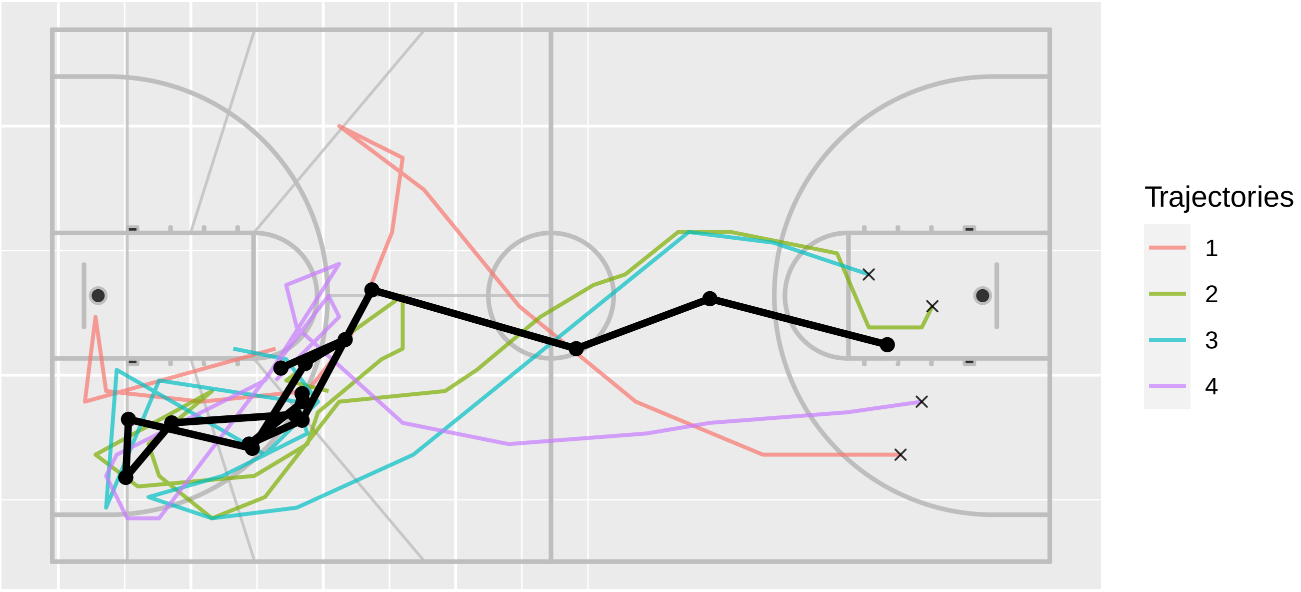

USA Area 2 Cluster 10 : SelectTrajectories

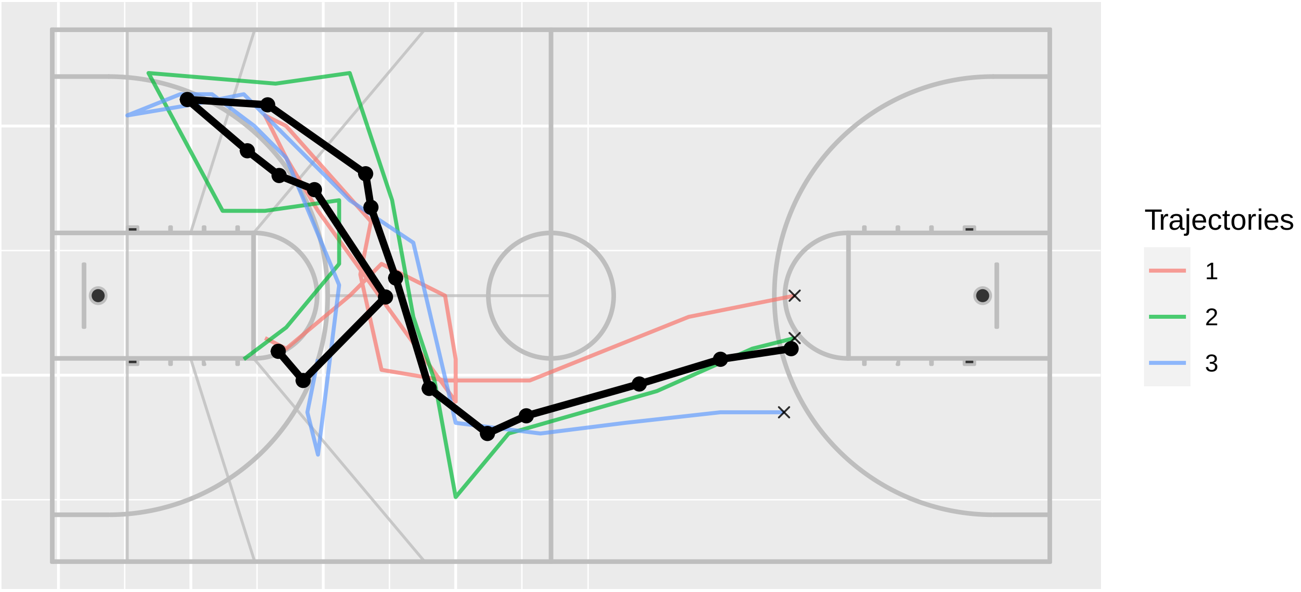

USA Area 2 Cluster 11 : SelectTrajectories

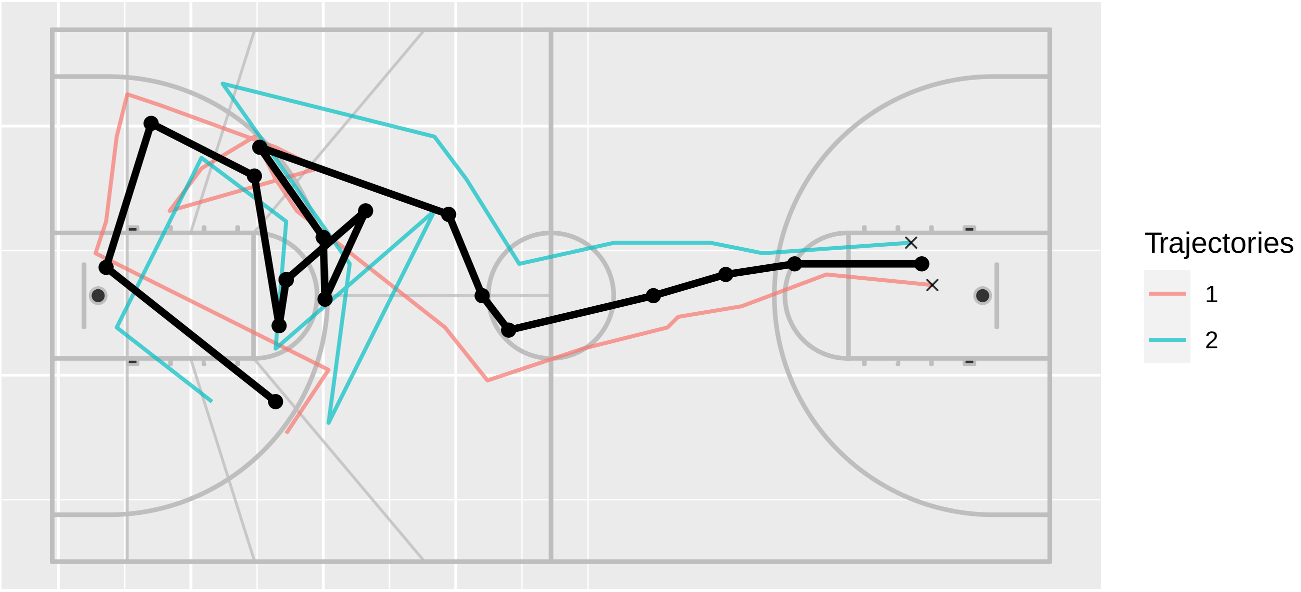

USA Area 2 Cluster 12 : SelectTrajectories

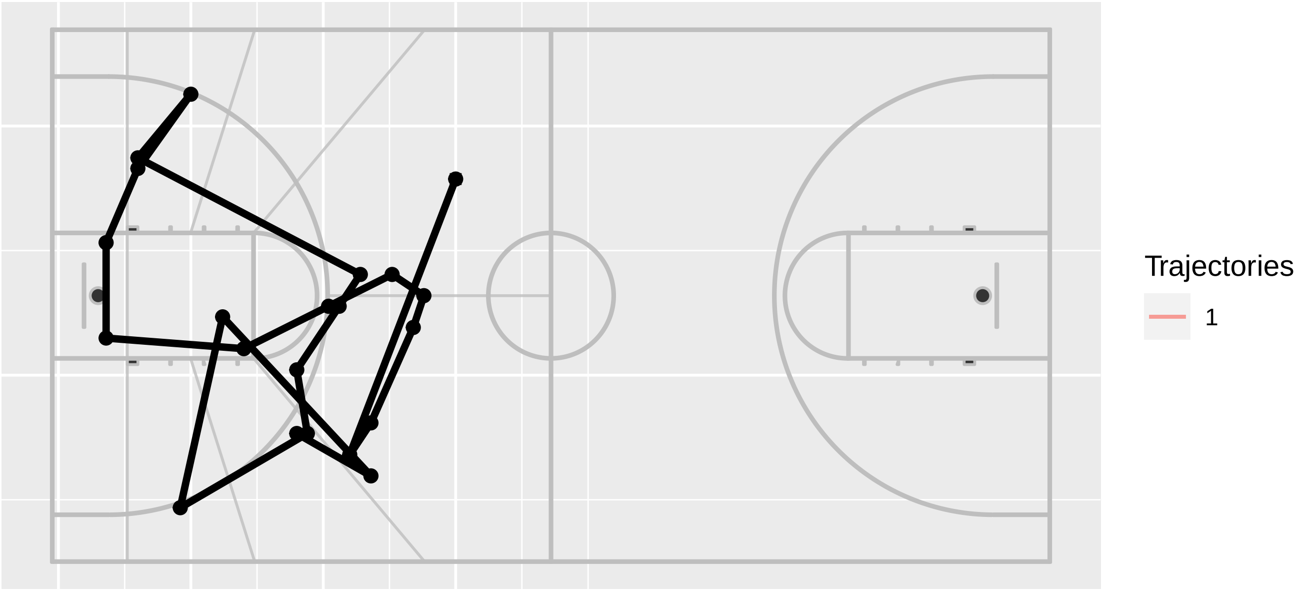

USA Area 3 Cluster 1 : SelectTrajectories

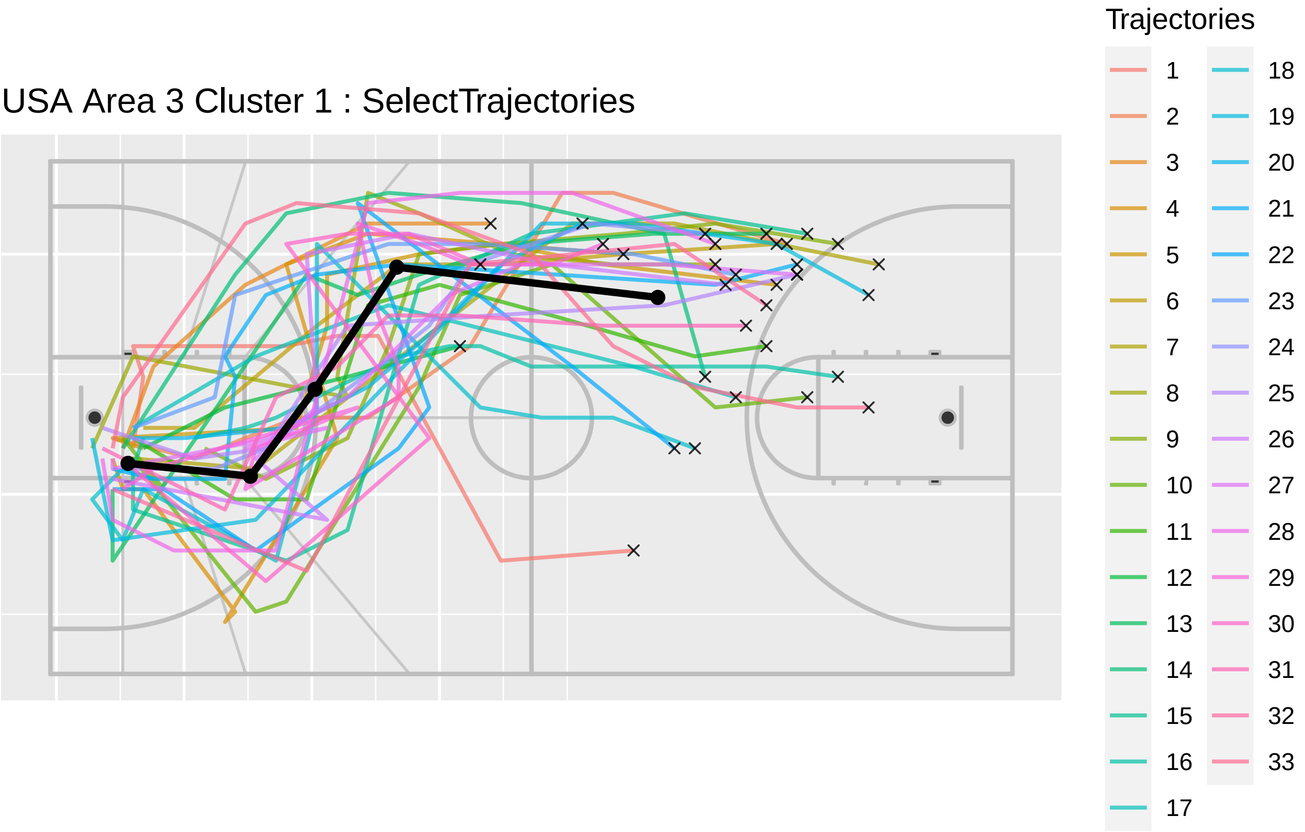

USA Area 3 Cluster 2 : SelectTrajectories

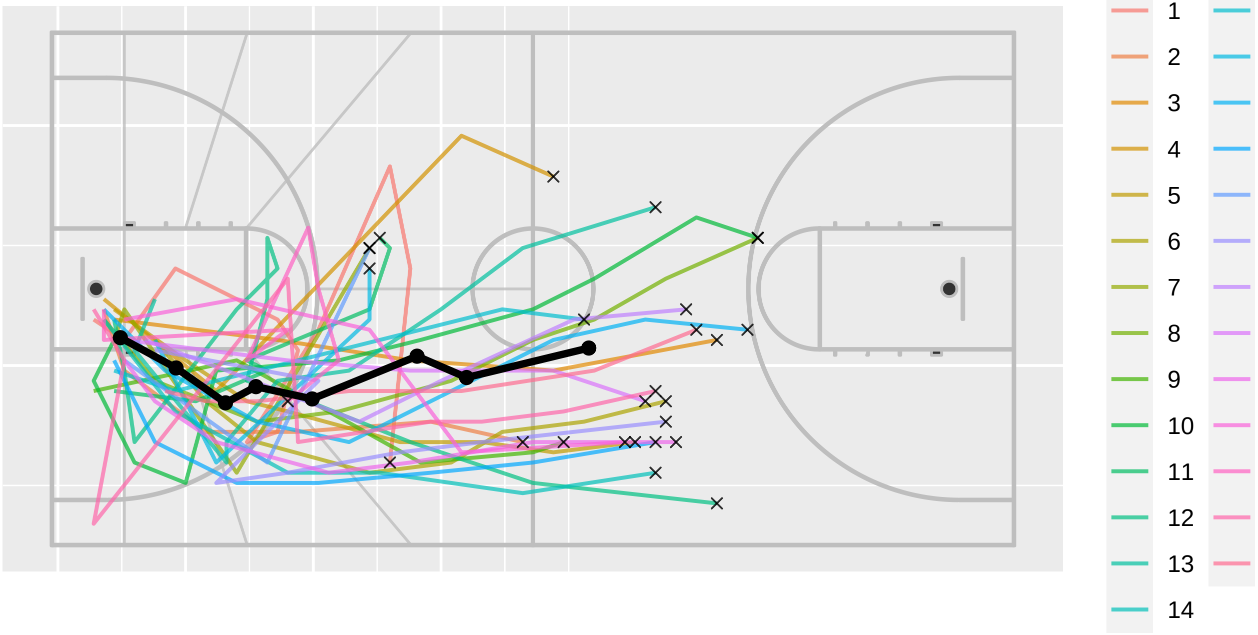

USA Area 3 Cluster 3 : SelectTrajectories

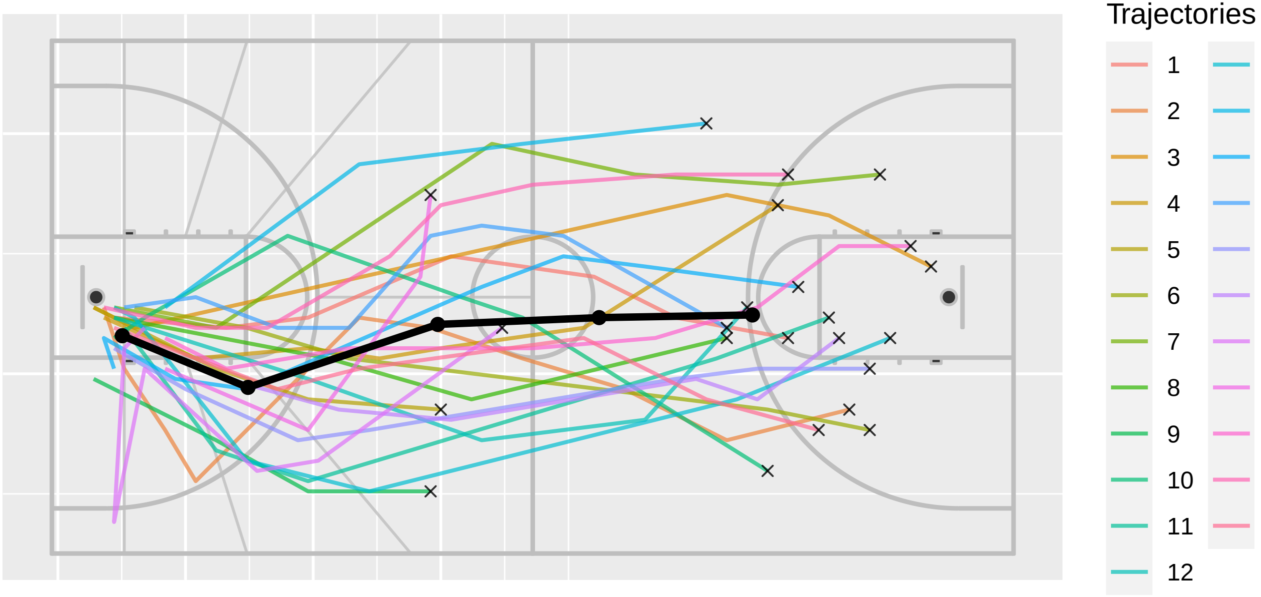

USA Area 3 Cluster 4 : SelectTrajectories

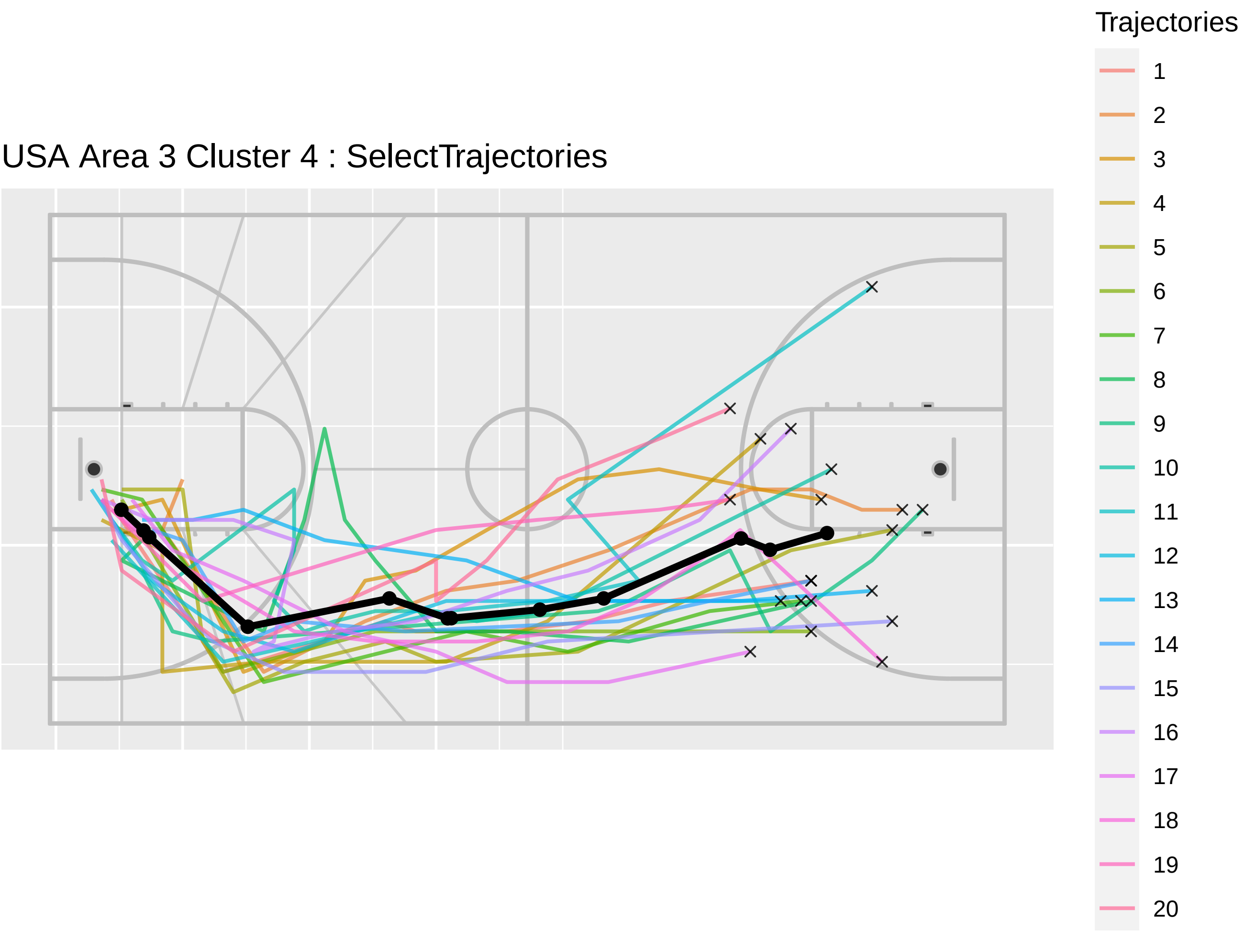

USA Area 3 Cluster 5 : SelectTrajectories

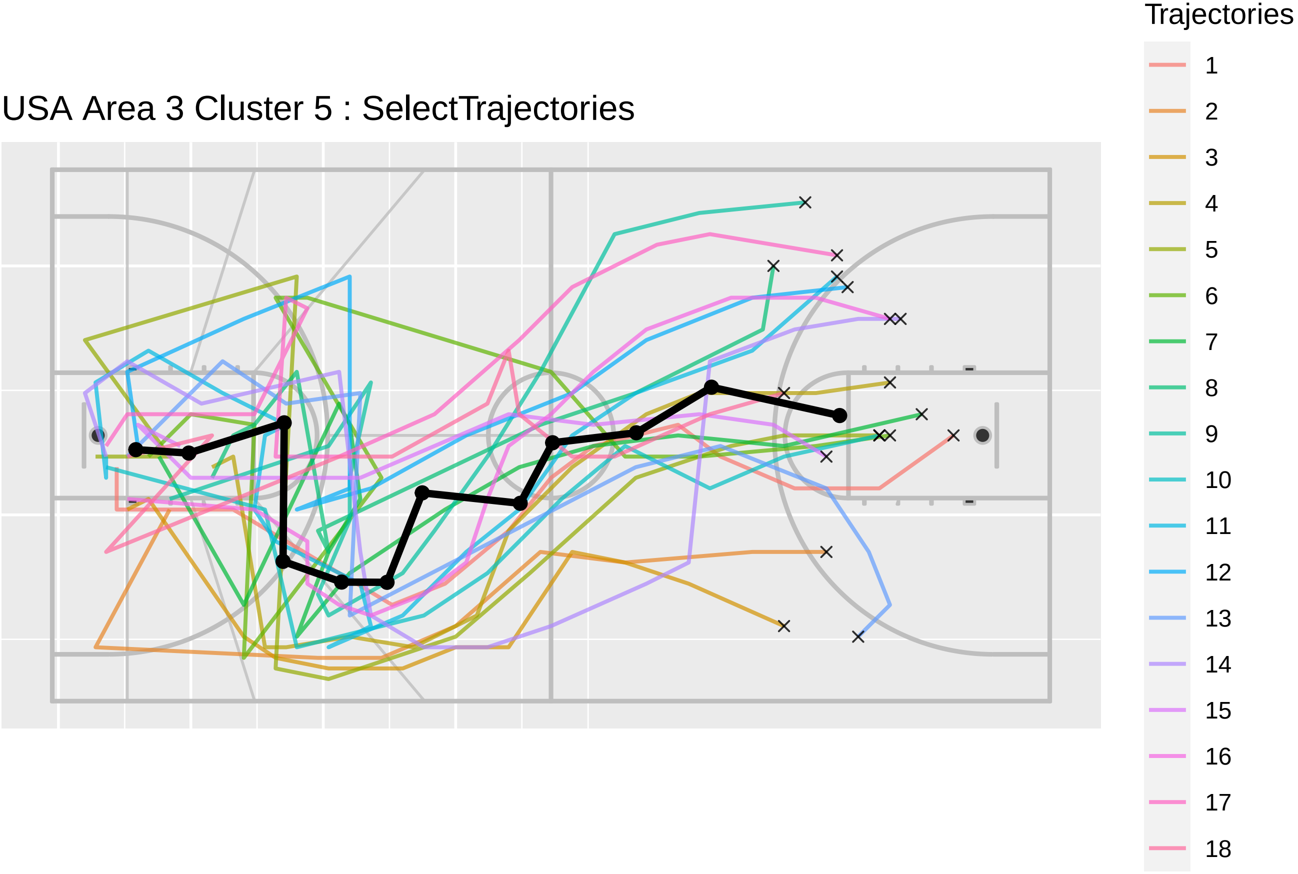

USA Area 3 Cluster 6 : SelectTrajectories

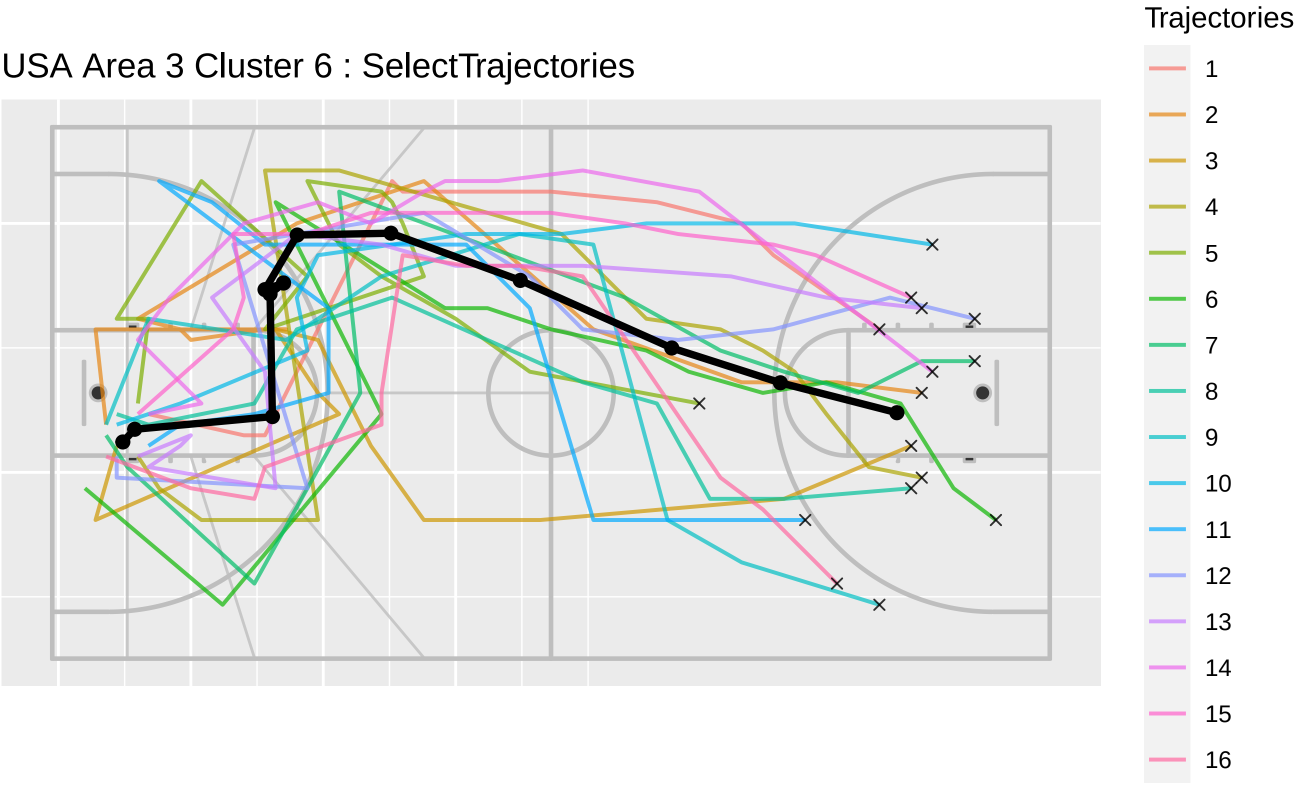

# USA Area 3 Cluster 7 : SelectTrajectories

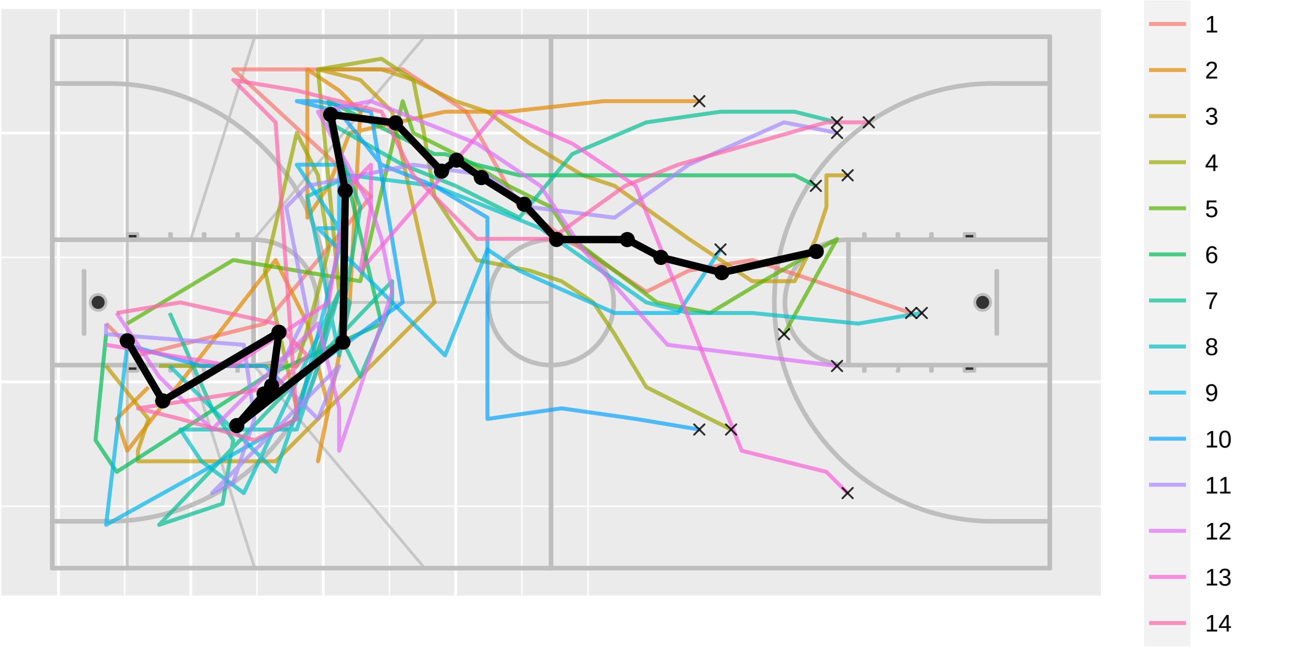

USA Area 3 Cluster 8 : SelectTrajectories

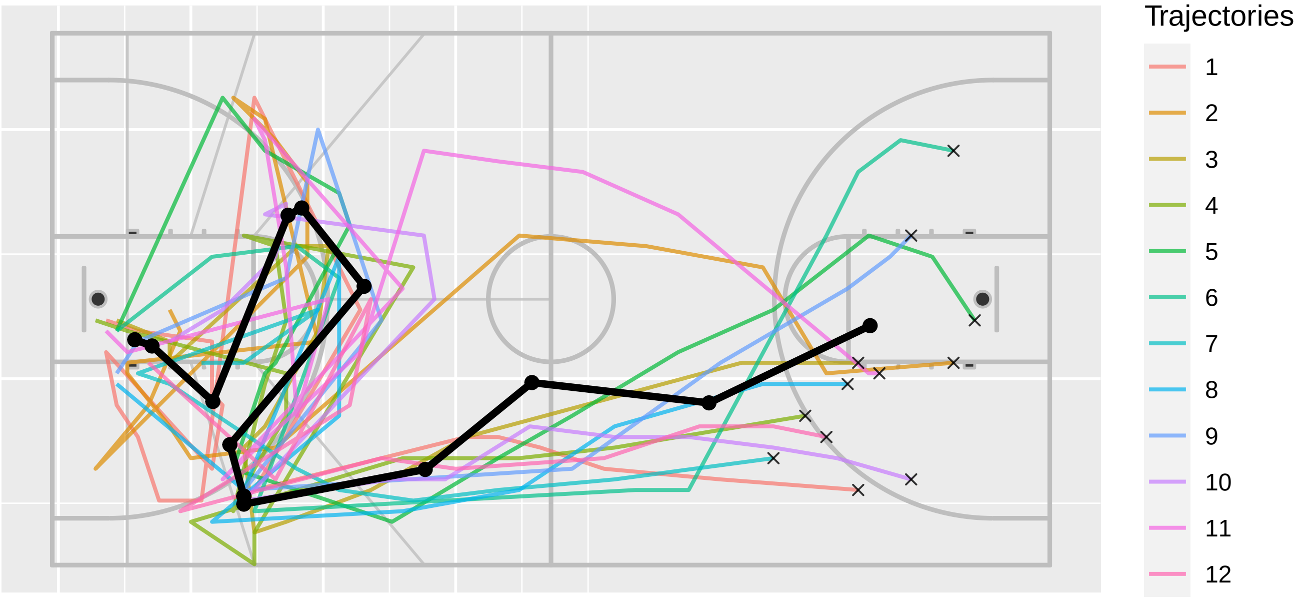

USA Area 3 Cluster 9 : SelectTrajectories

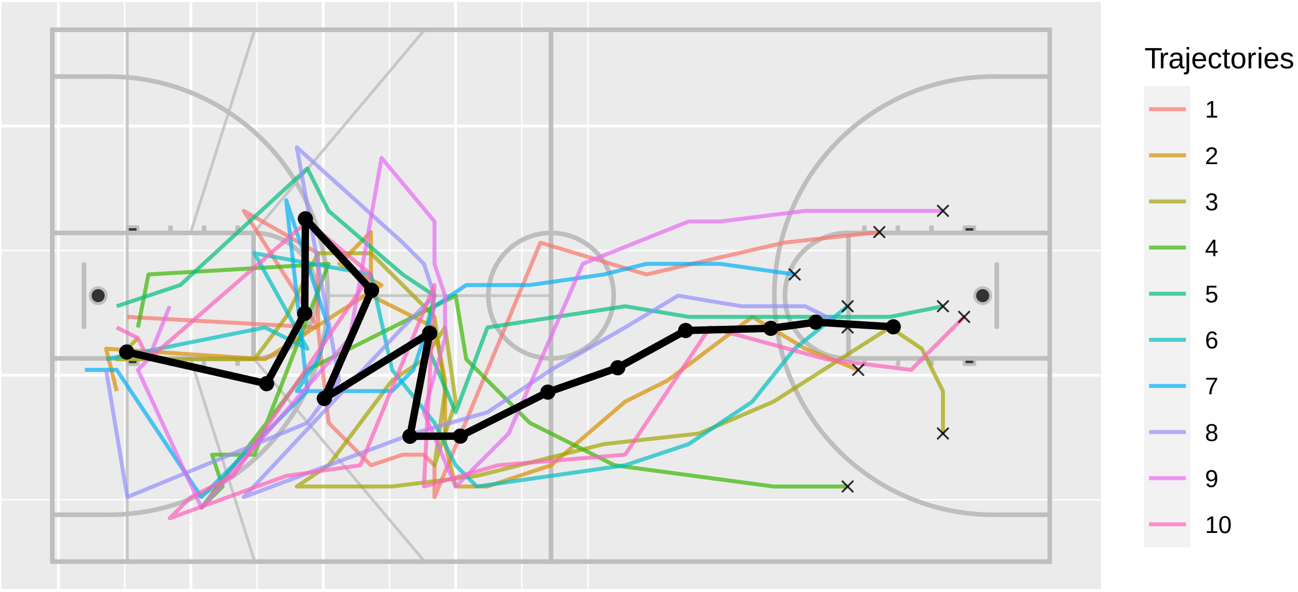

USA Area 3 Cluster 10 : SelectTrajectories

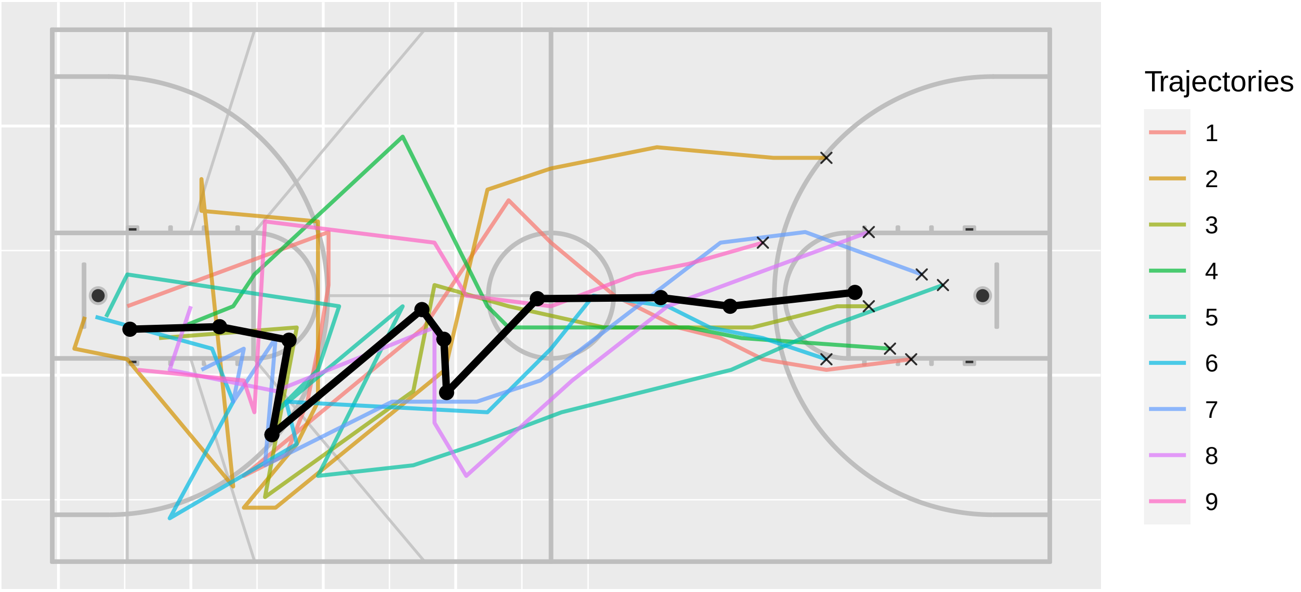

USA Area 3 Cluster 11 : SelectTrajectories

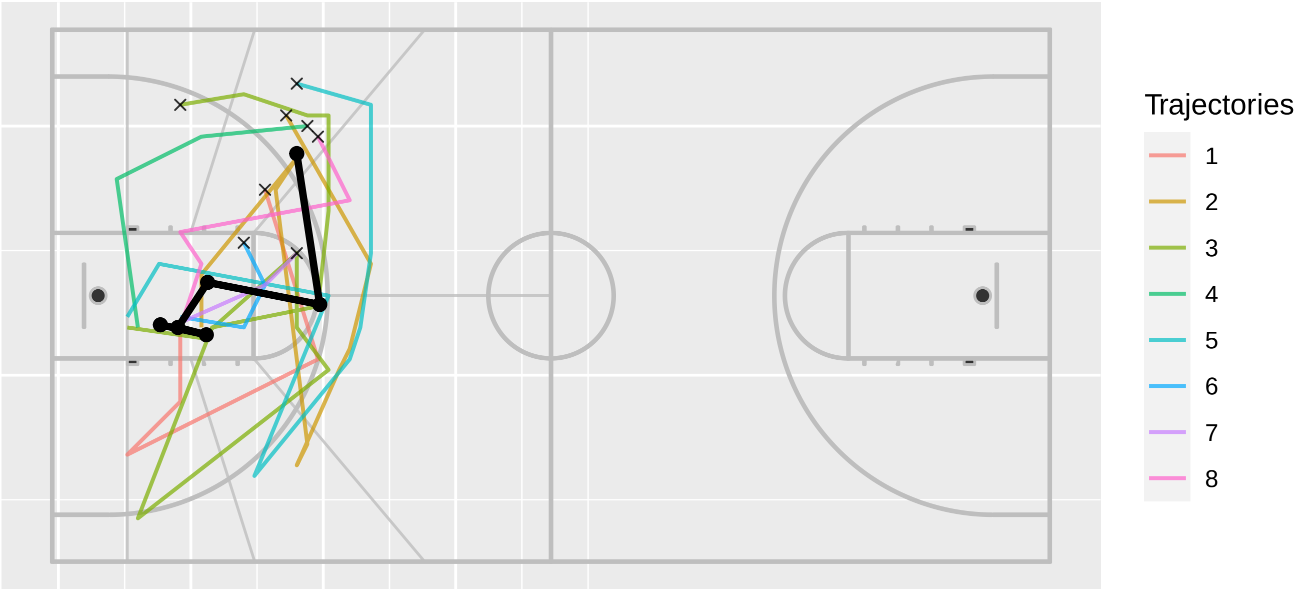

USA Area 3 Cluster 12 : SelectTrajectories

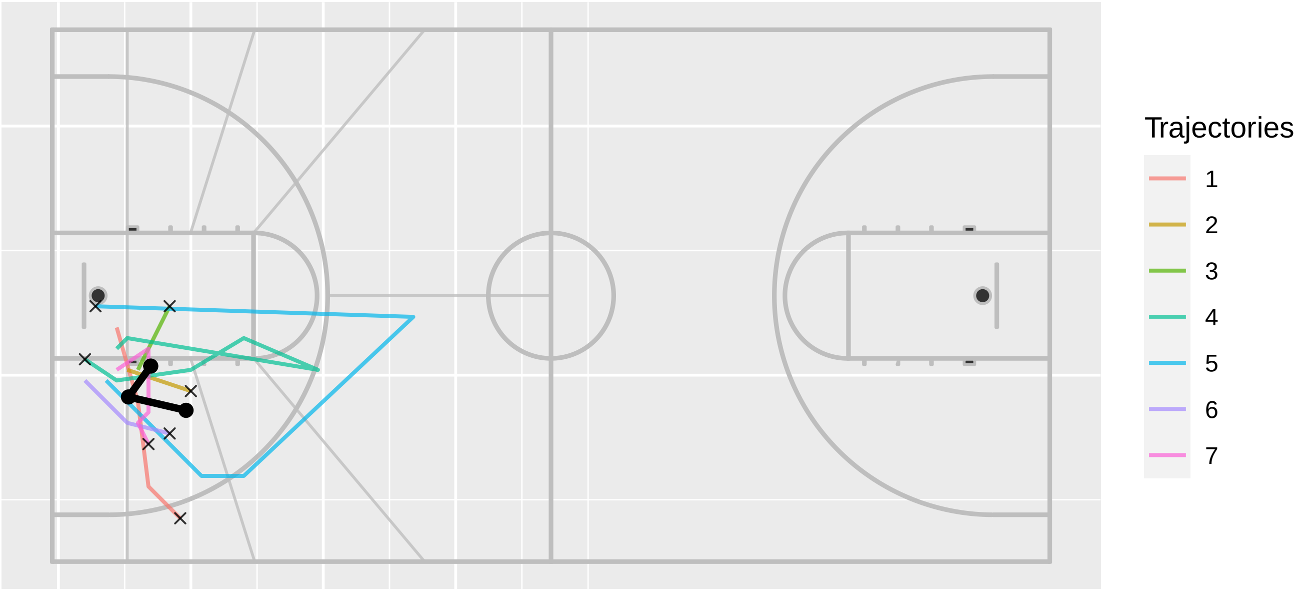

USA Area 3 Cluster 13 : SelectTrajectories

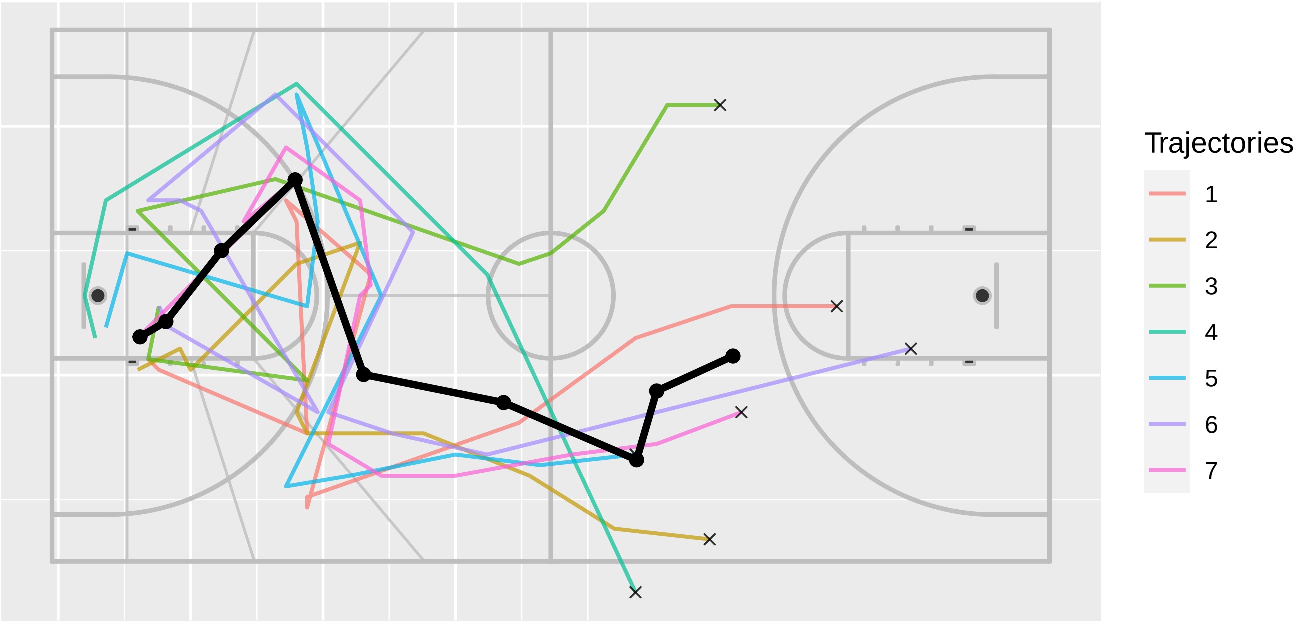

USA Area 3 Cluster 14 : SelectTrajectories

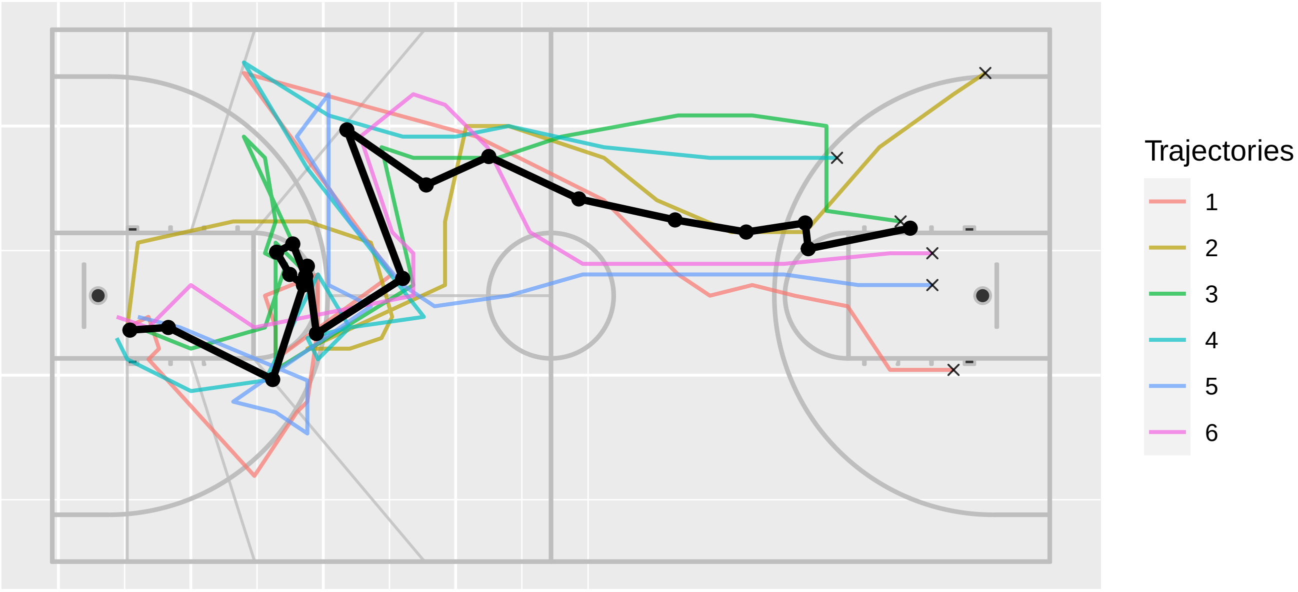

USA Area 3 Cluster 15 : SelectTrajectories

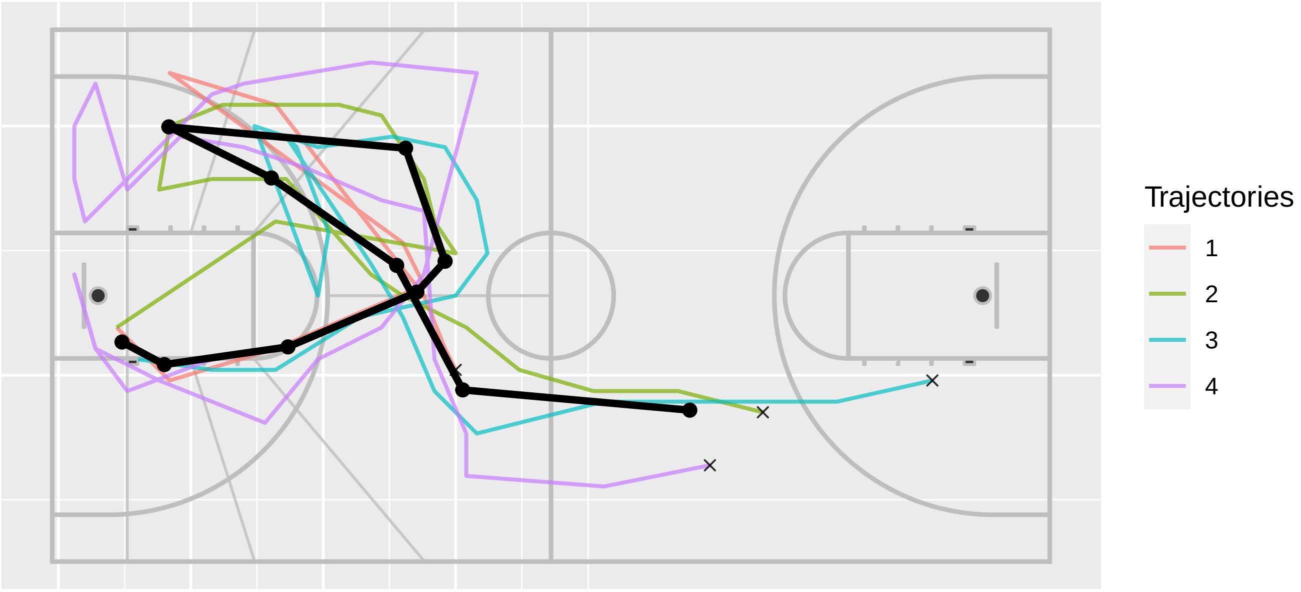

USA Area 3 Cluster 16 : SelectTrajectories

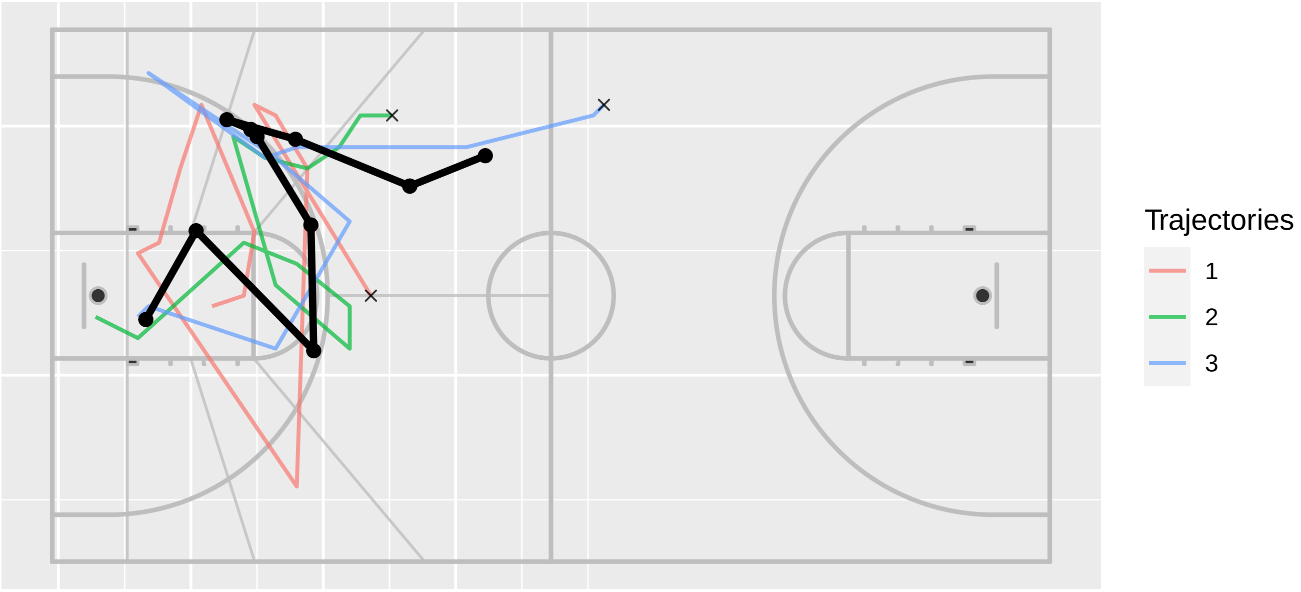

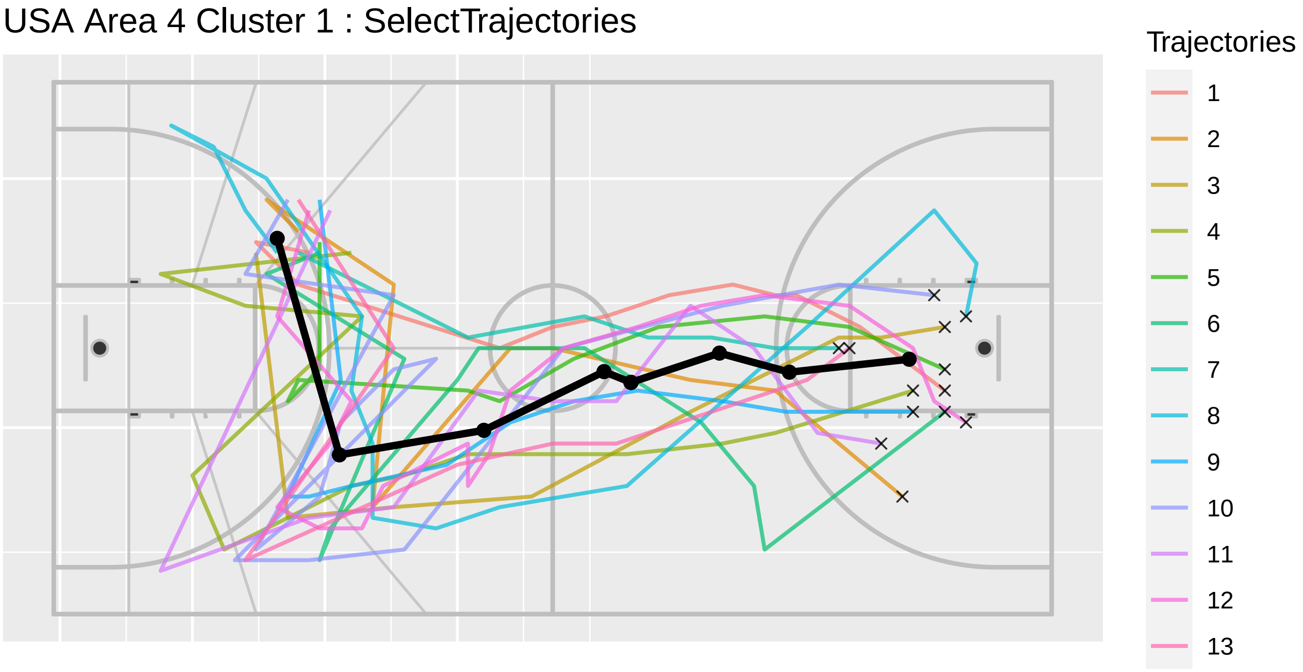

USA Area 4 Cluster 1 : SelectTrajectories
Trajectories
1
2
3
4
5
6
7
8
9
10
11
12
13

USA Area 4 Cluster 2 : SelectTrajectories

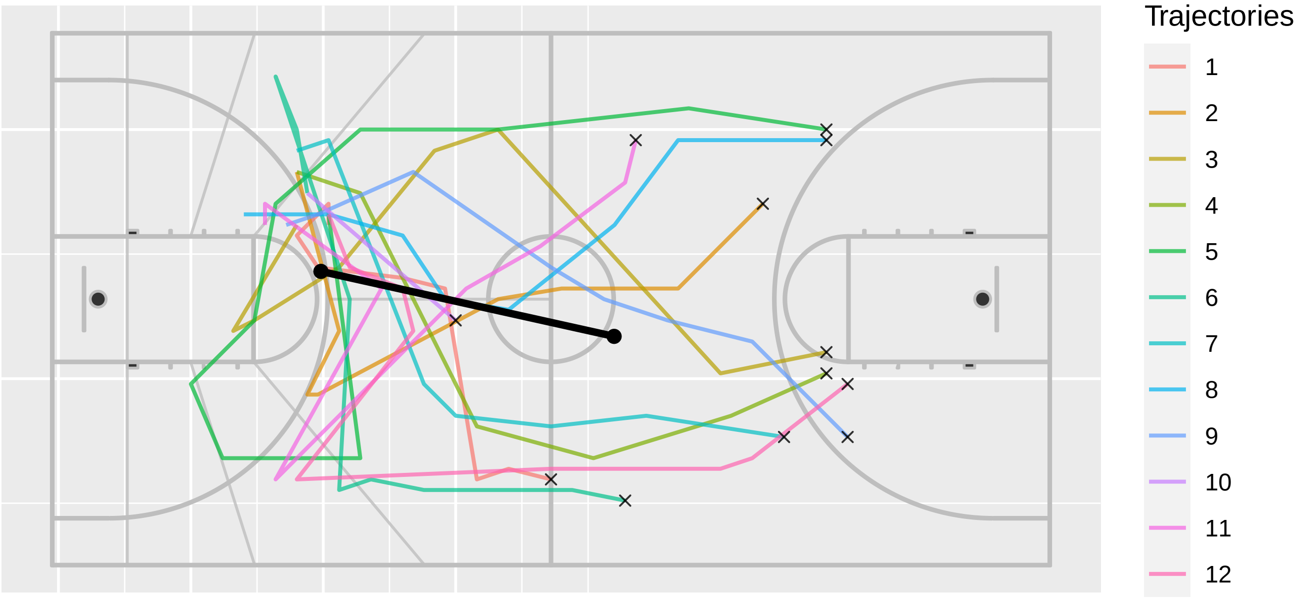

USA Area 4 Cluster 3 : SelectTrajectories

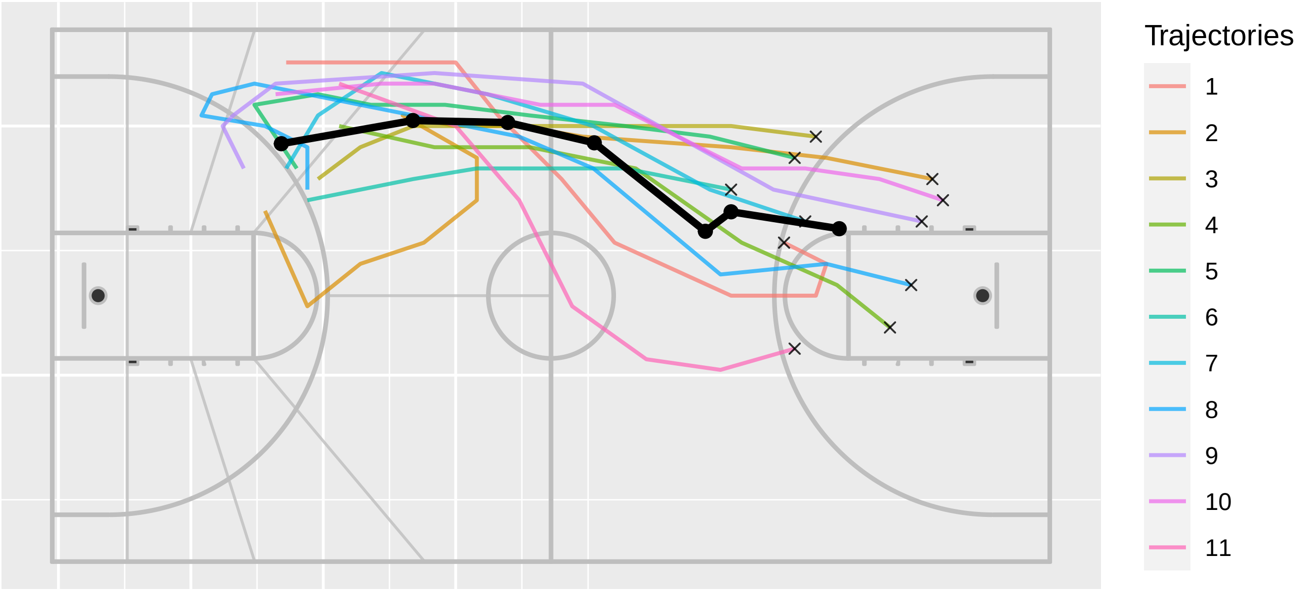

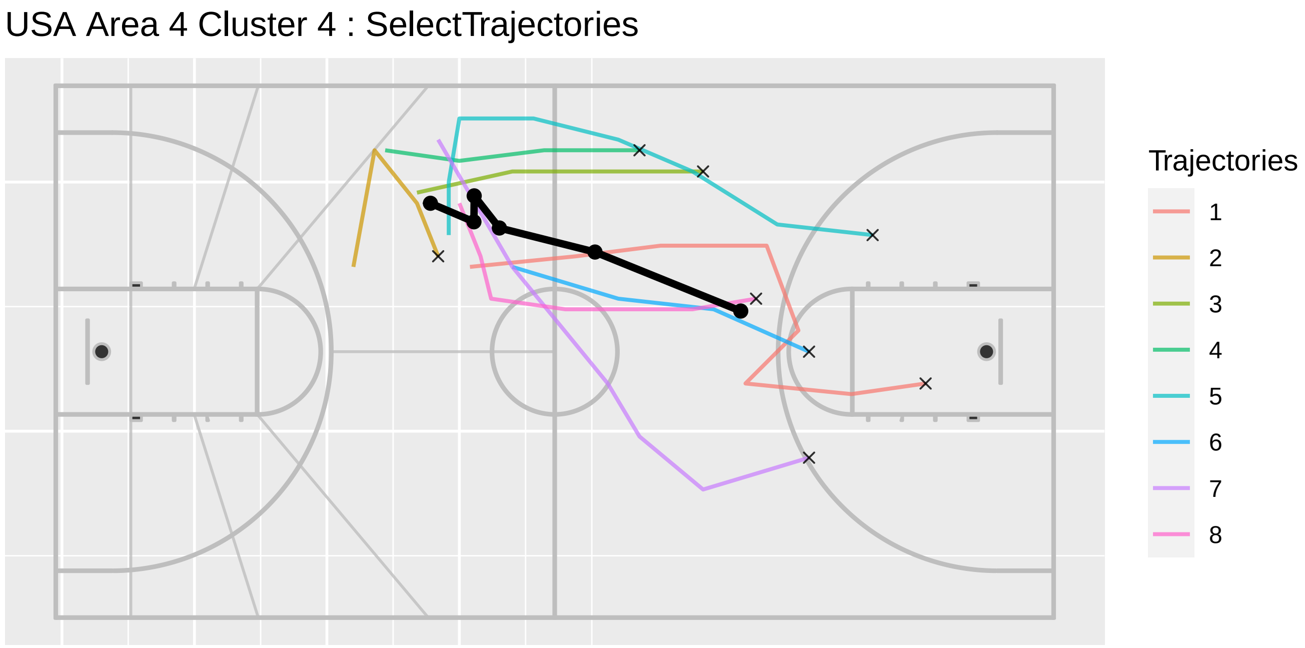
USA Area 4 Cluster 4 : SelectTrajectories
Trajectories
1
2
3
4
5
6
7
8

USA Area 4 Cluster 5 : SelectTrajectories

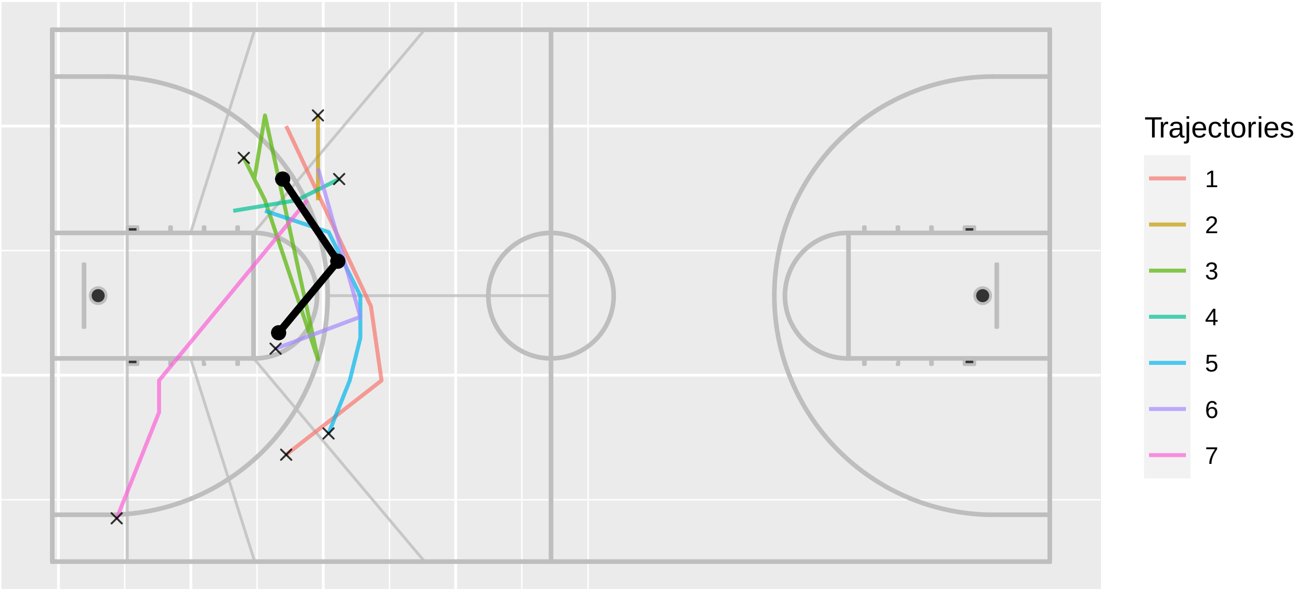

USA Area 4 Cluster 6 : SelectTrajectories

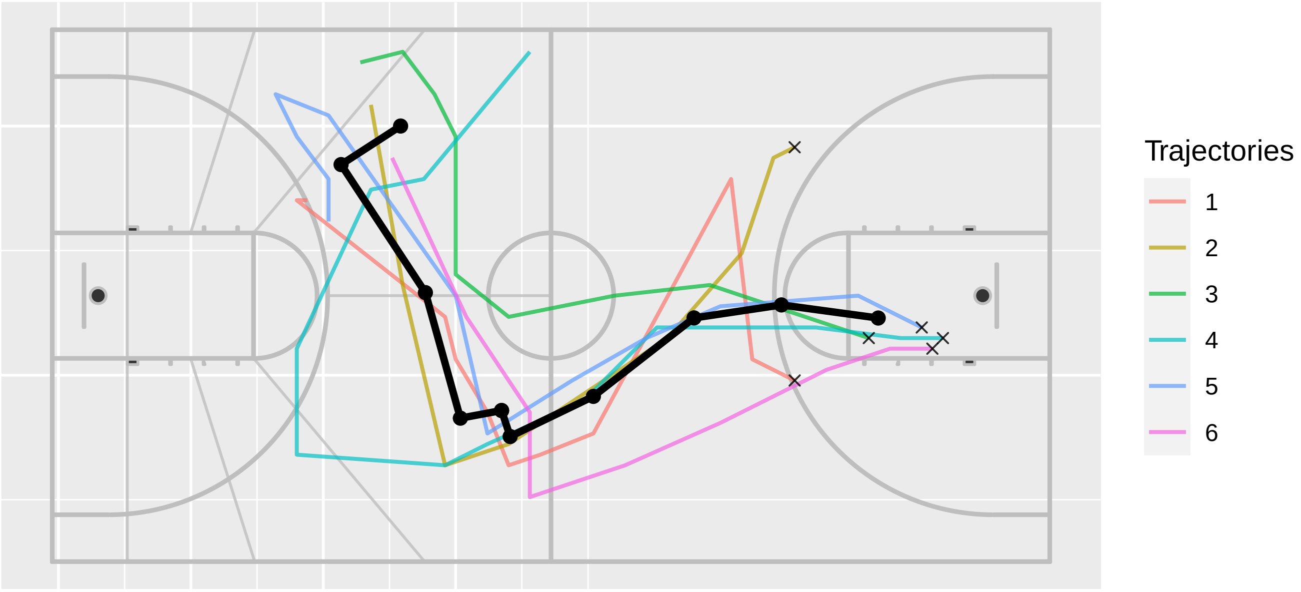

USA Area 4 Cluster 7 : SelectTrajectories

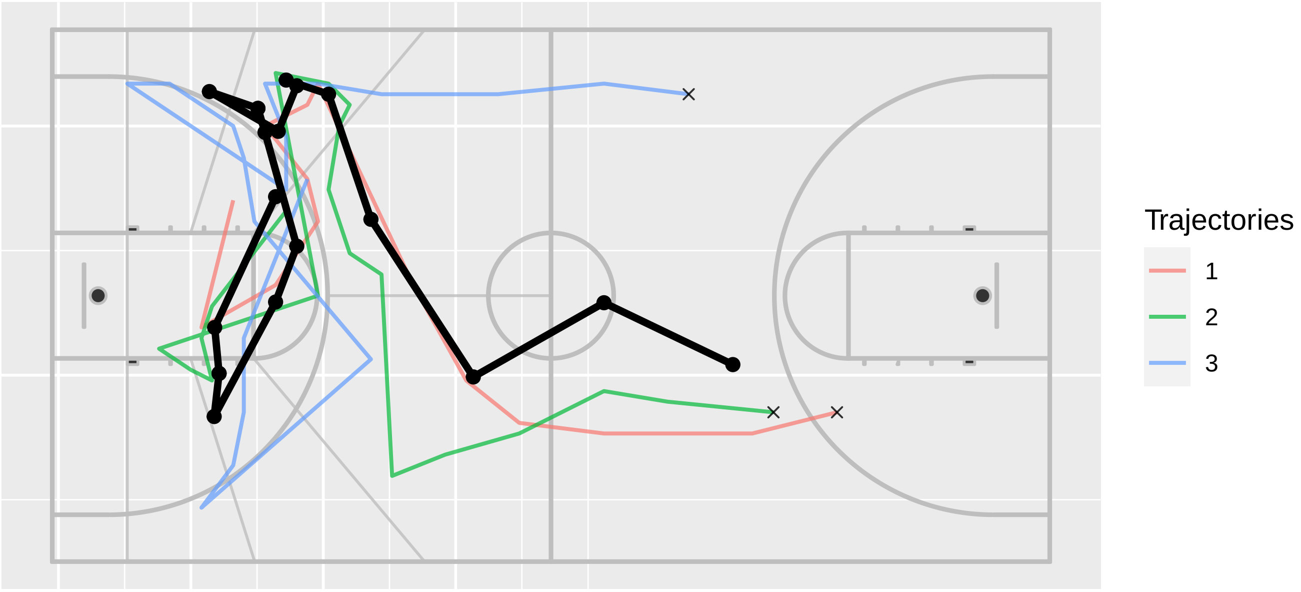

USA Area 4 Cluster 8 : SelectTrajectories

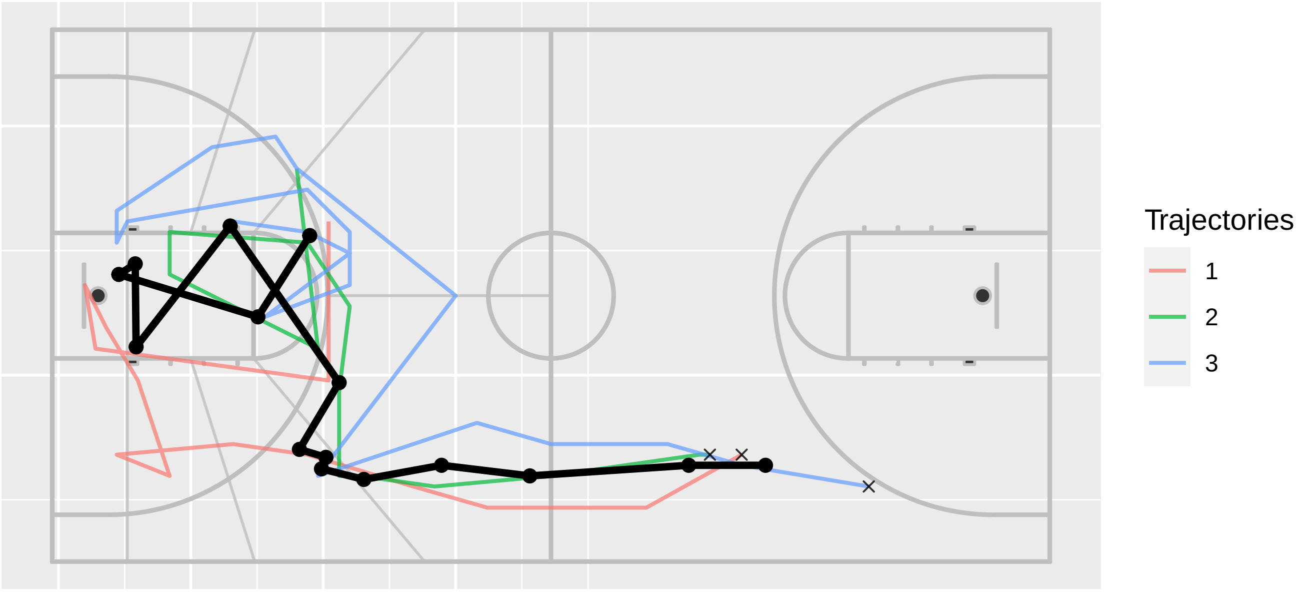

USA Area 4 Cluster 9 : SelectTrajectories

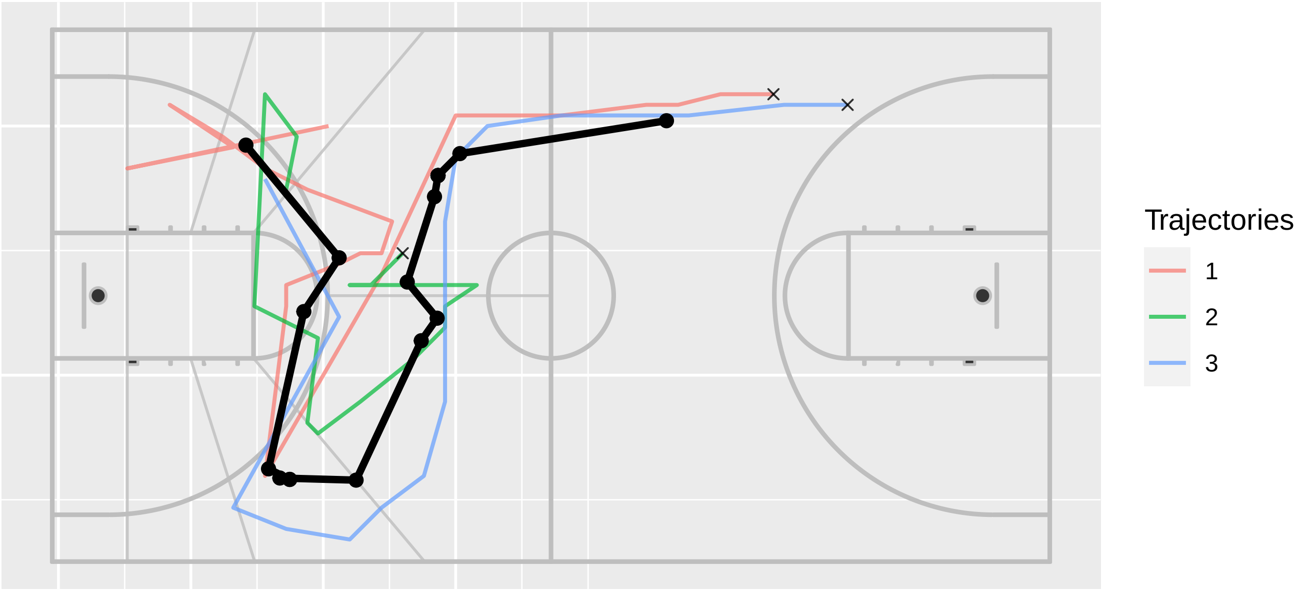

USA Area 4 Cluster 10 : SelectTrajectories

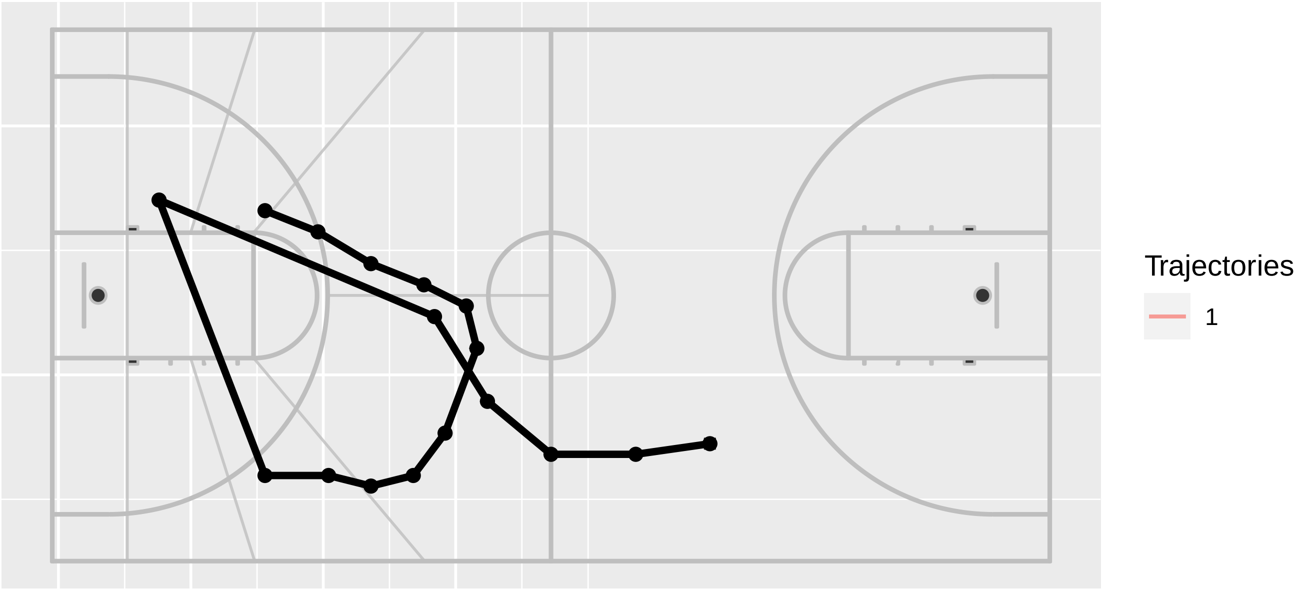

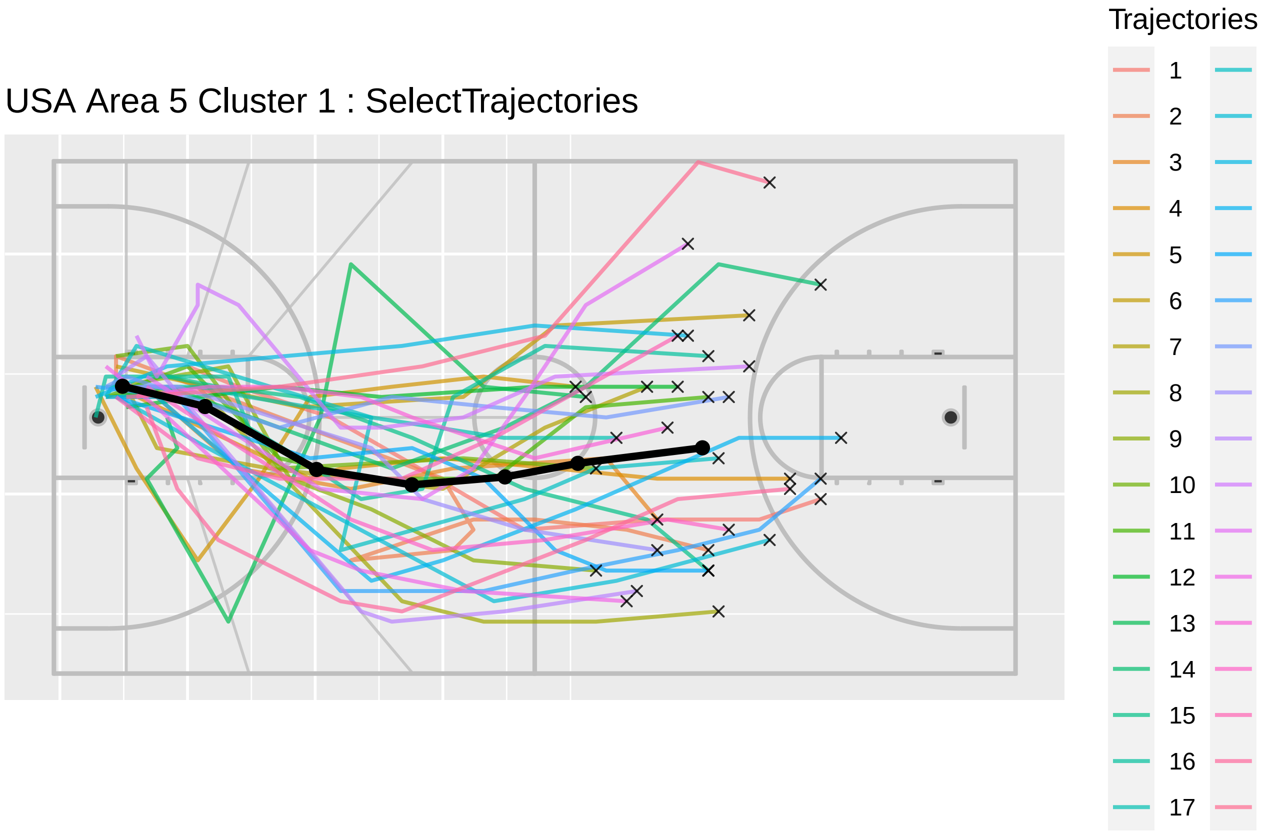
USA Area 5 Cluster 1 : SelectTrajectories
Trajectories
1
2
3
4
5
6
7
8
9
10
11
12
13
14
15
16
17
18
19
20
21
22
23
24
25
26
27
28
29
30
31
32
33
34

USA Area 5 Cluster 2 : SelectTrajectories

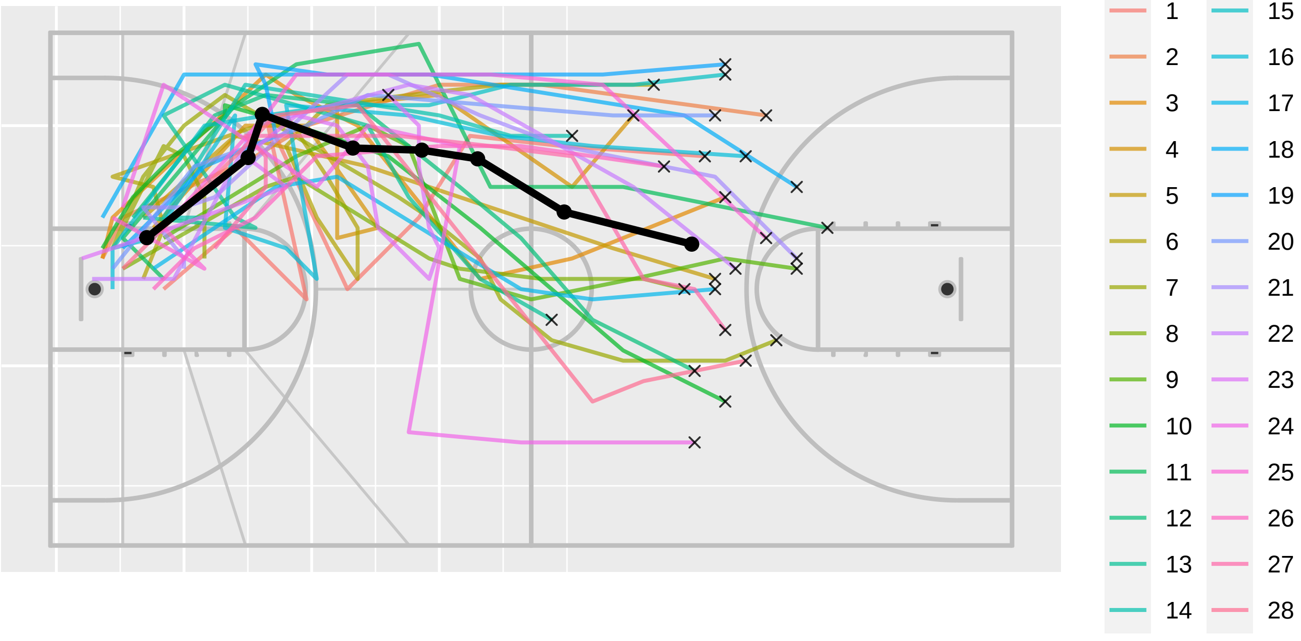

USA Area 5 Cluster 3 : SelectTrajectories

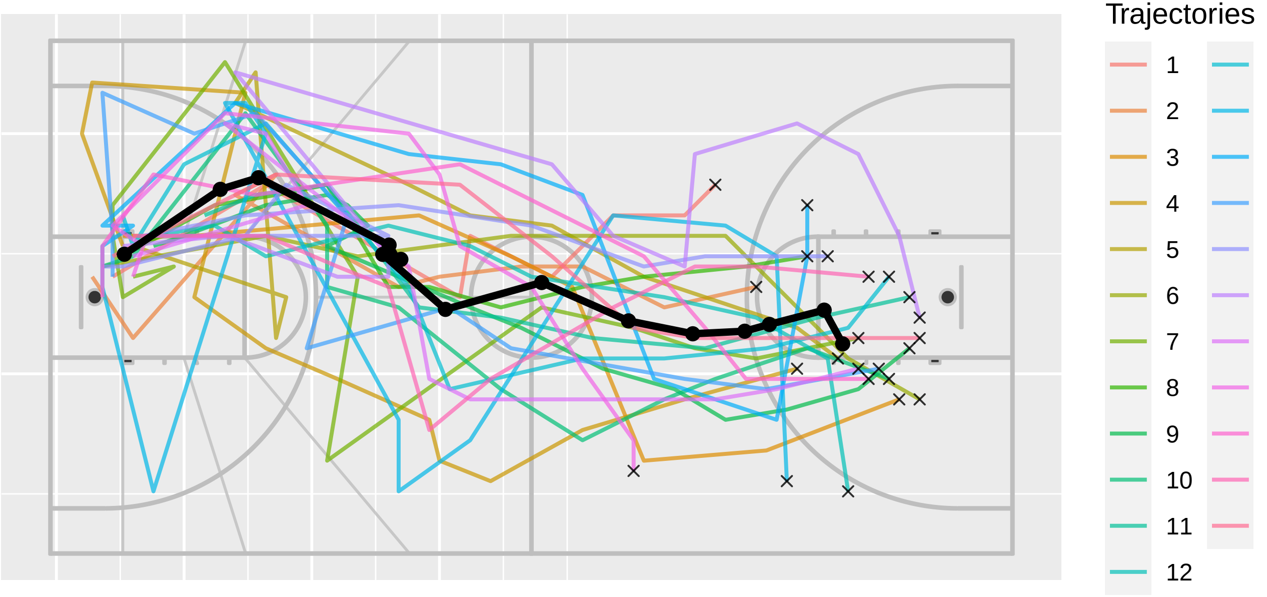

USA Area 5 Cluster 4 : SelectTrajectories

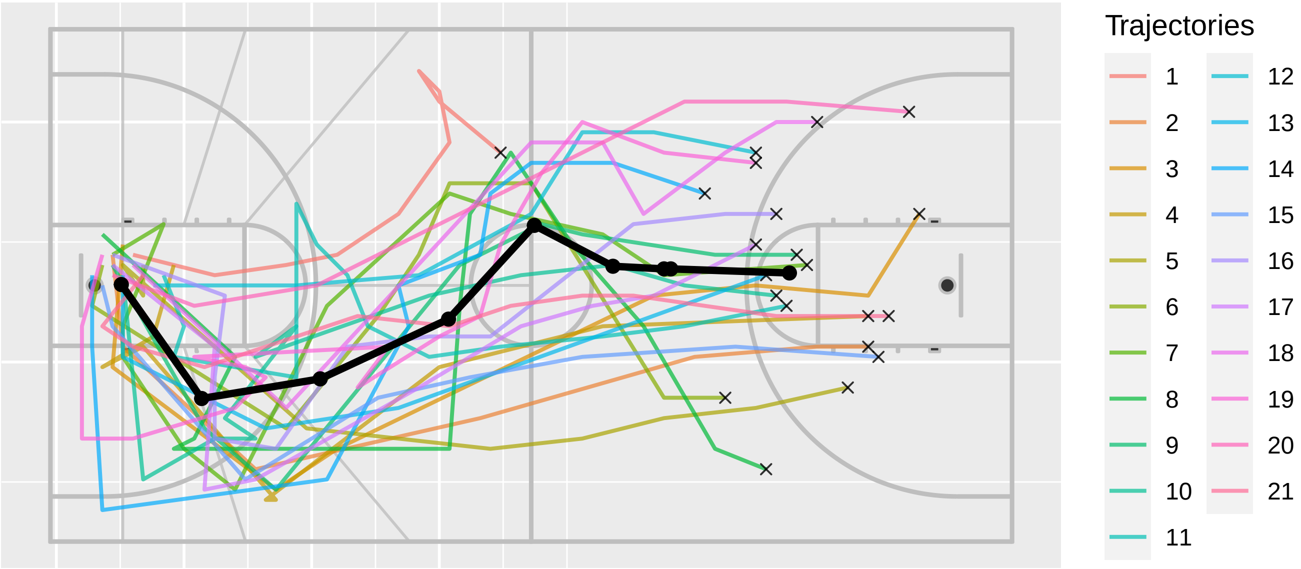

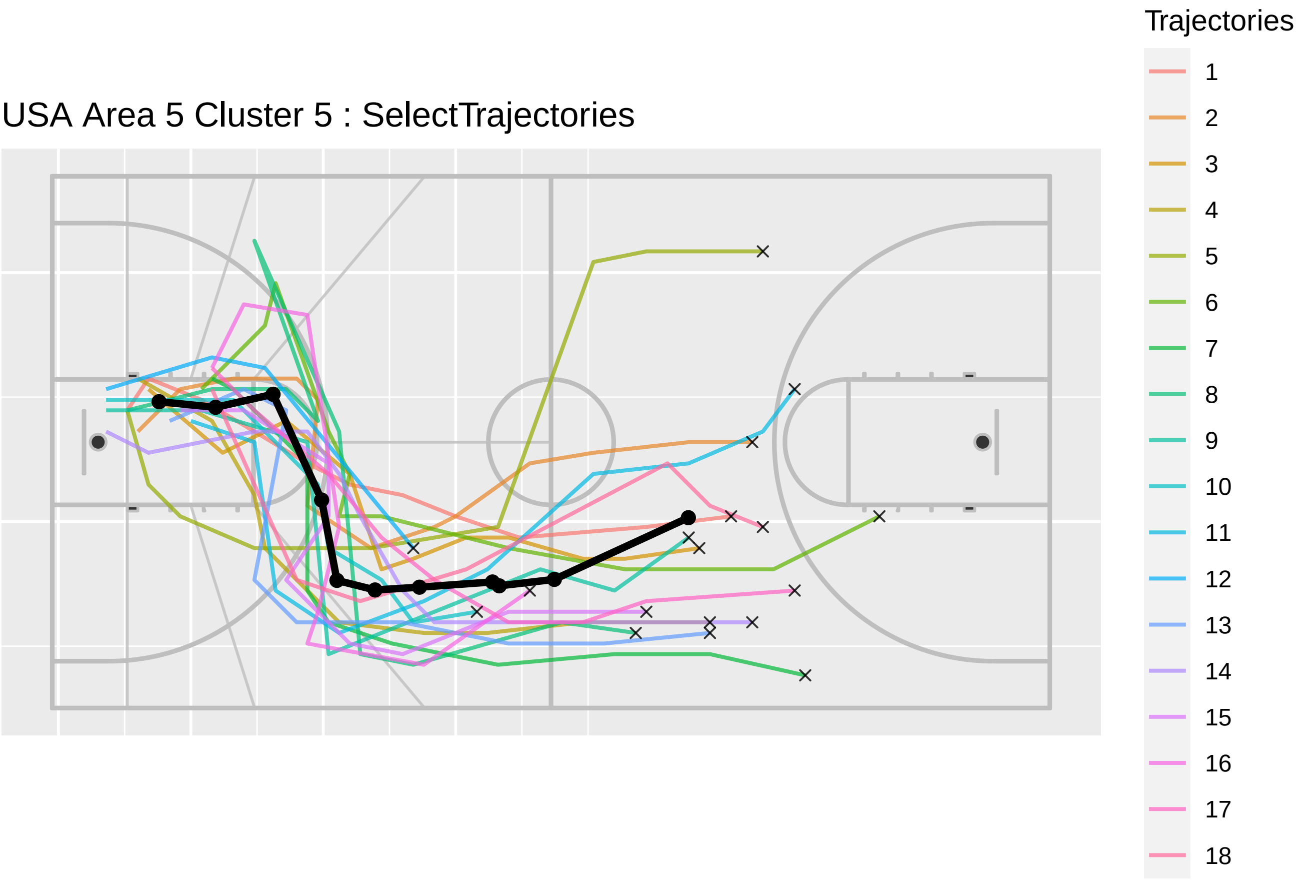
USA Area 5 Cluster 5 : SelectTrajectories
Trajectories
1
2
3
4
5
6
7
8
9
10
11
12
13
14
15
16
17
18

USA Area 5 Cluster 6 : SelectTrajectories

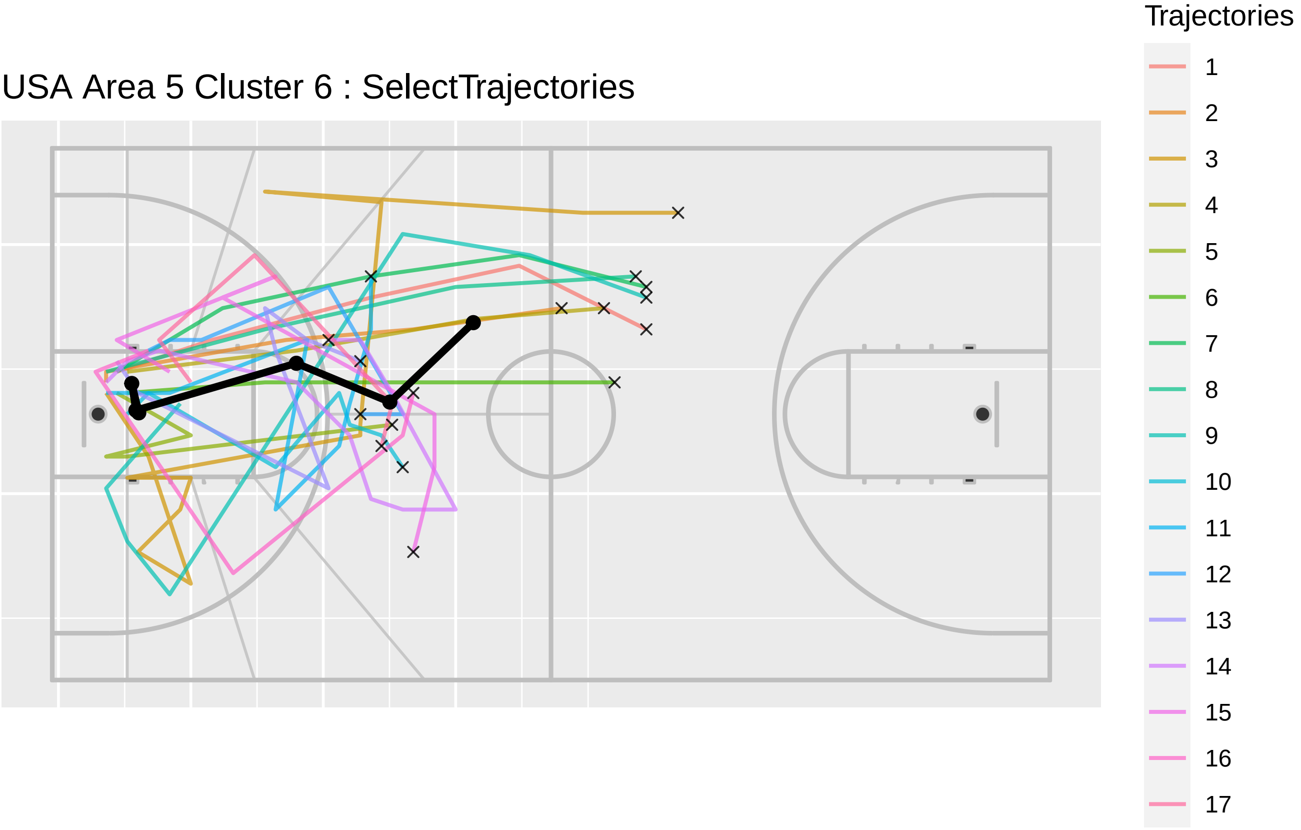

USA Area 5 Cluster 7 : SelectTrajectories

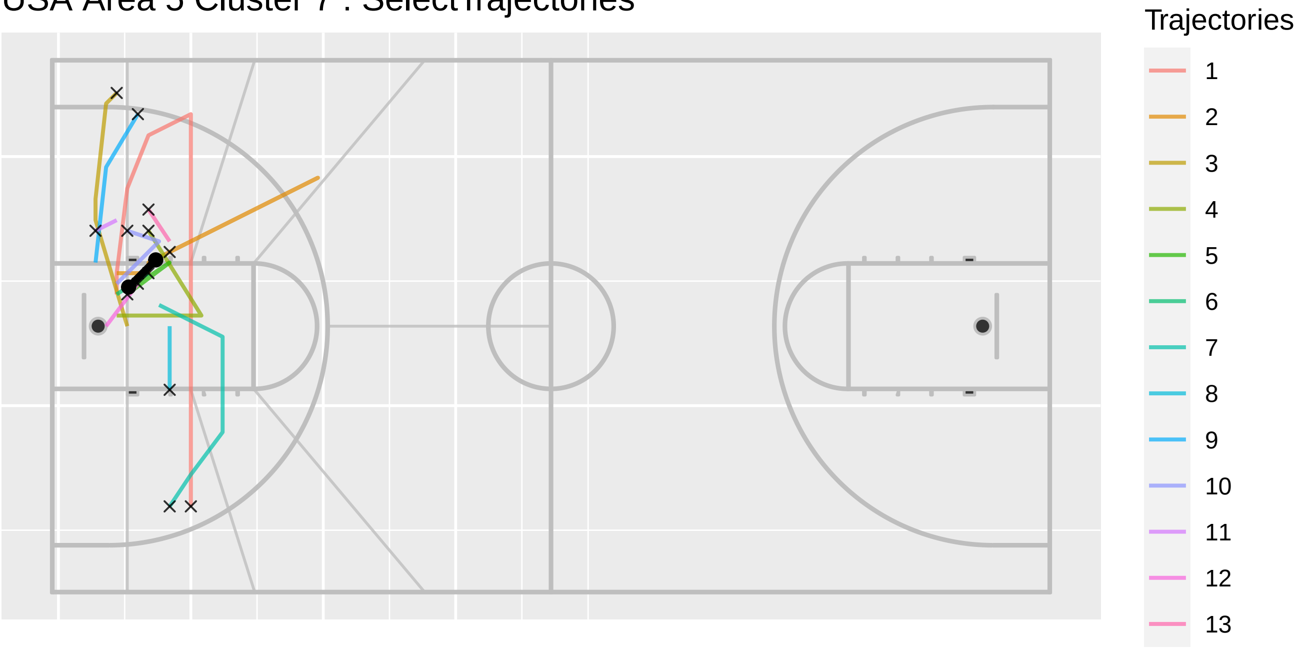

USA Area 5 Cluster 8 : SelectTrajectories

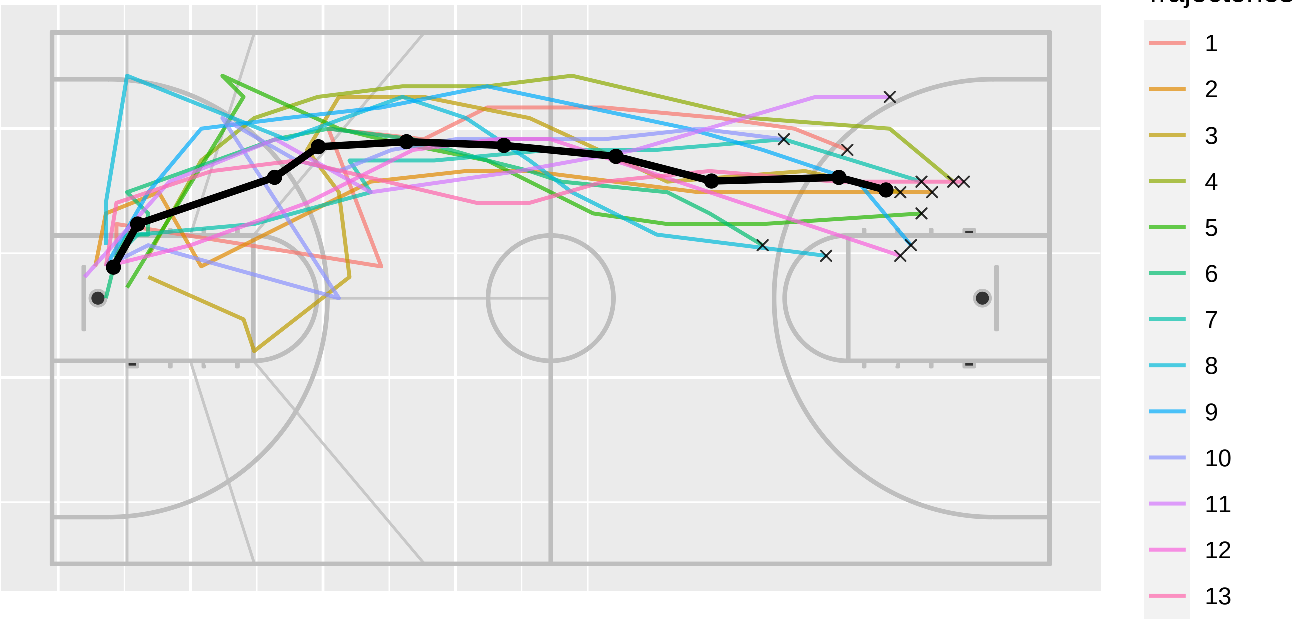

USA Area 5 Cluster 9 : SelectTrajectories

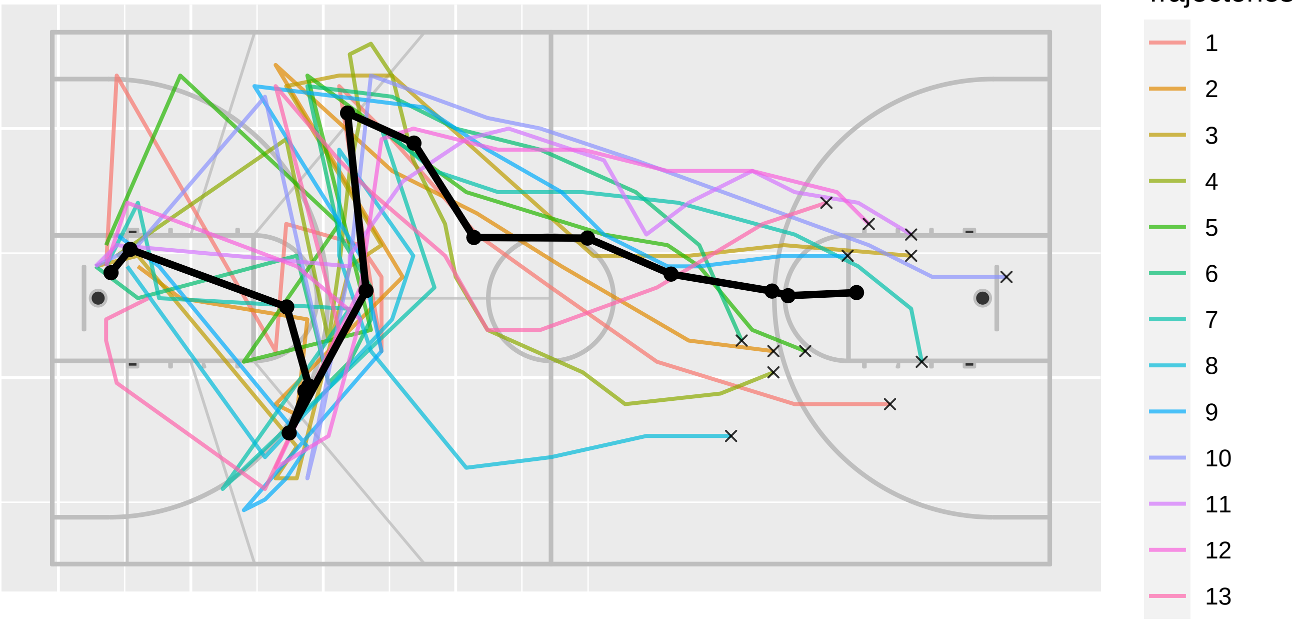

USA Area 5 Cluster 10 : SelectTrajectories

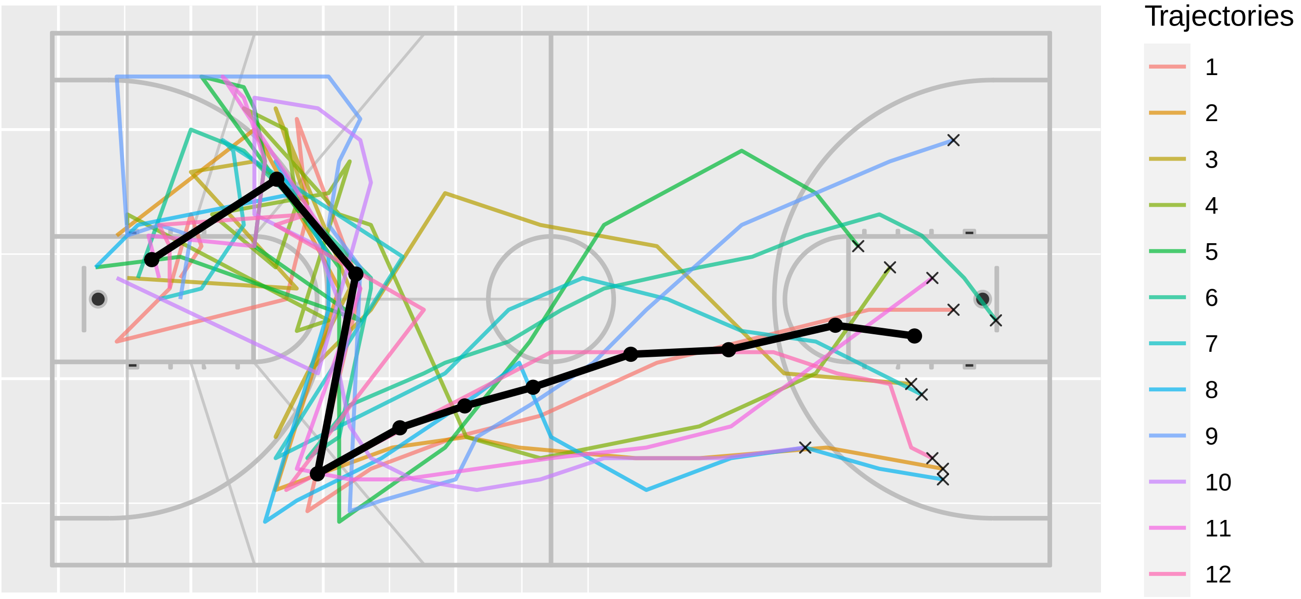

USA Area 5 Cluster 11 : SelectTrajectories

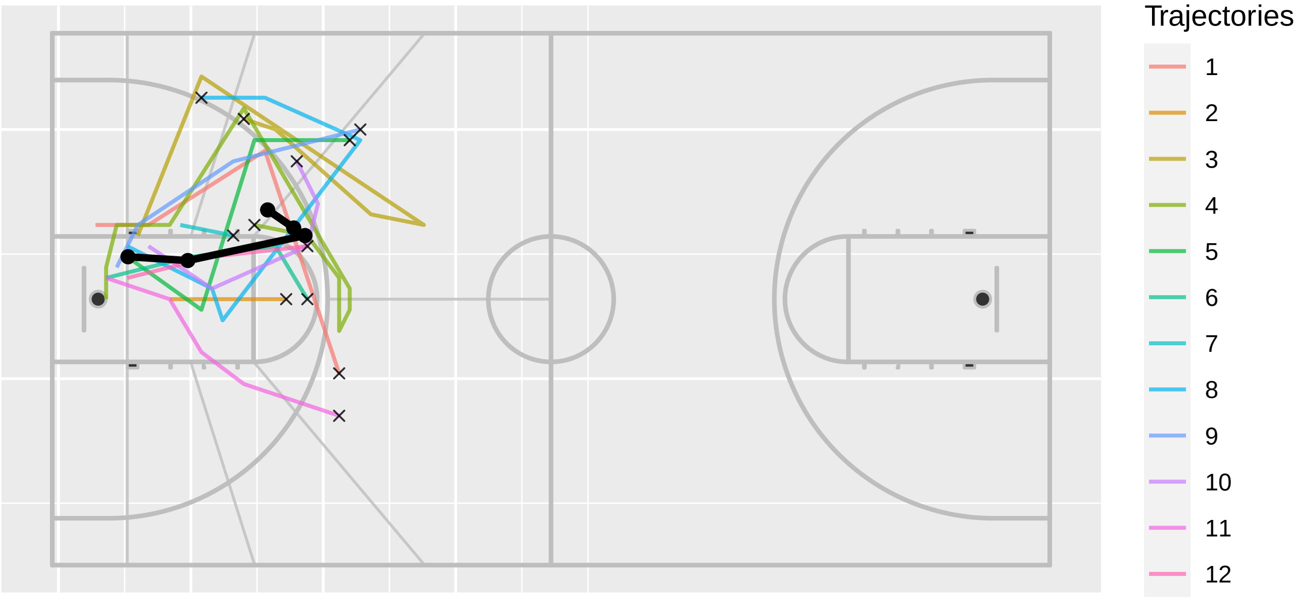

USA Area 5 Cluster 12 : SelectTrajectories

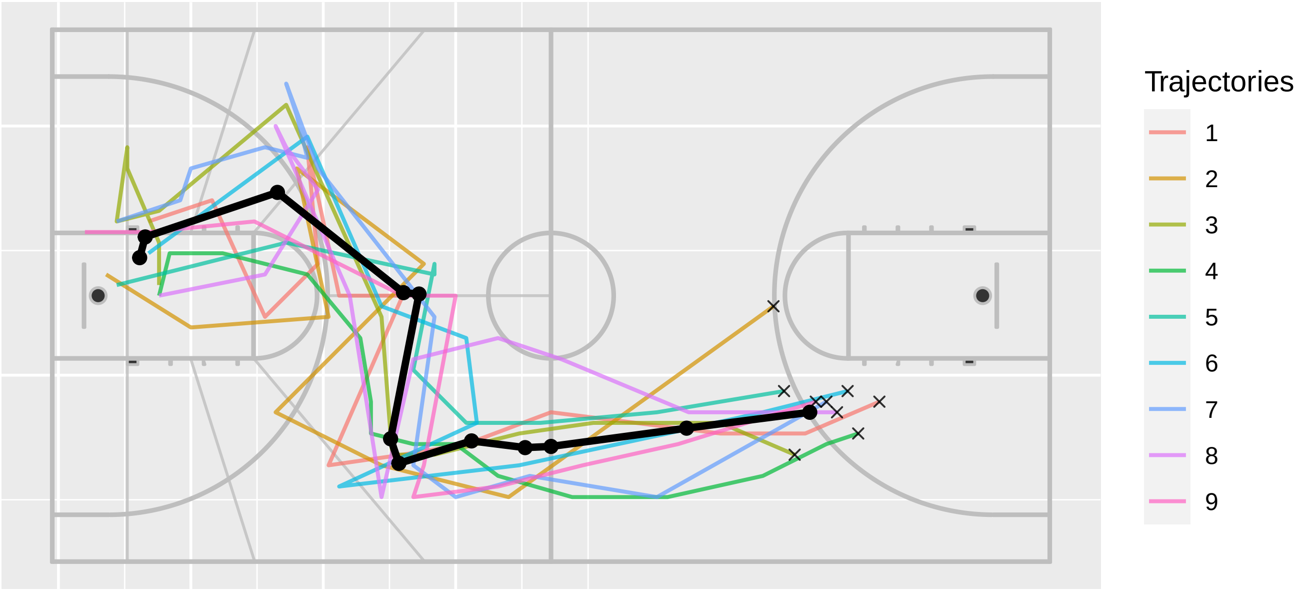

USA Area 5 Cluster 13 : SelectTrajectories

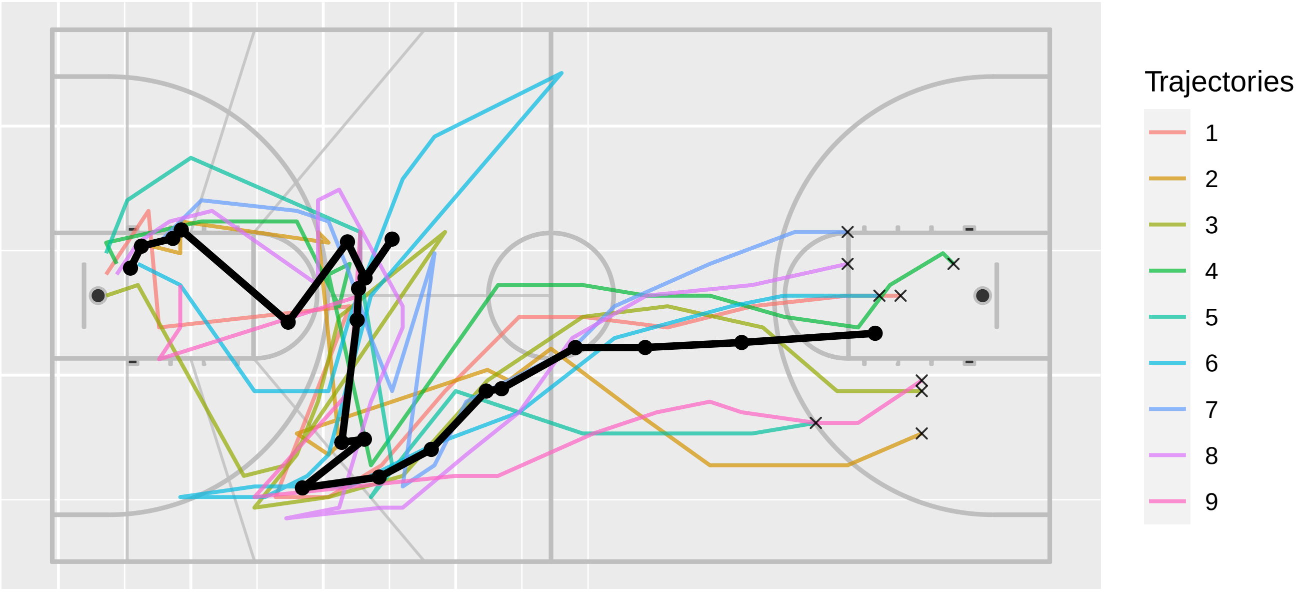

USA Area 5 Cluster 14 : SelectTrajectories

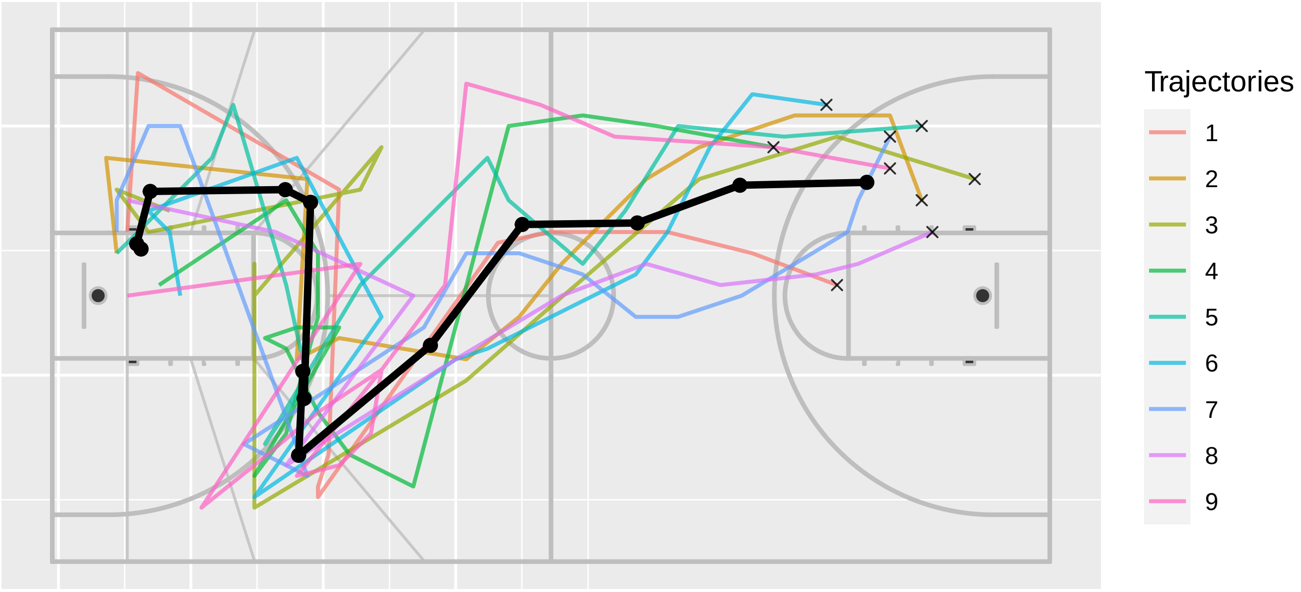

USA Area 5 Cluster 15 : SelectTrajectories

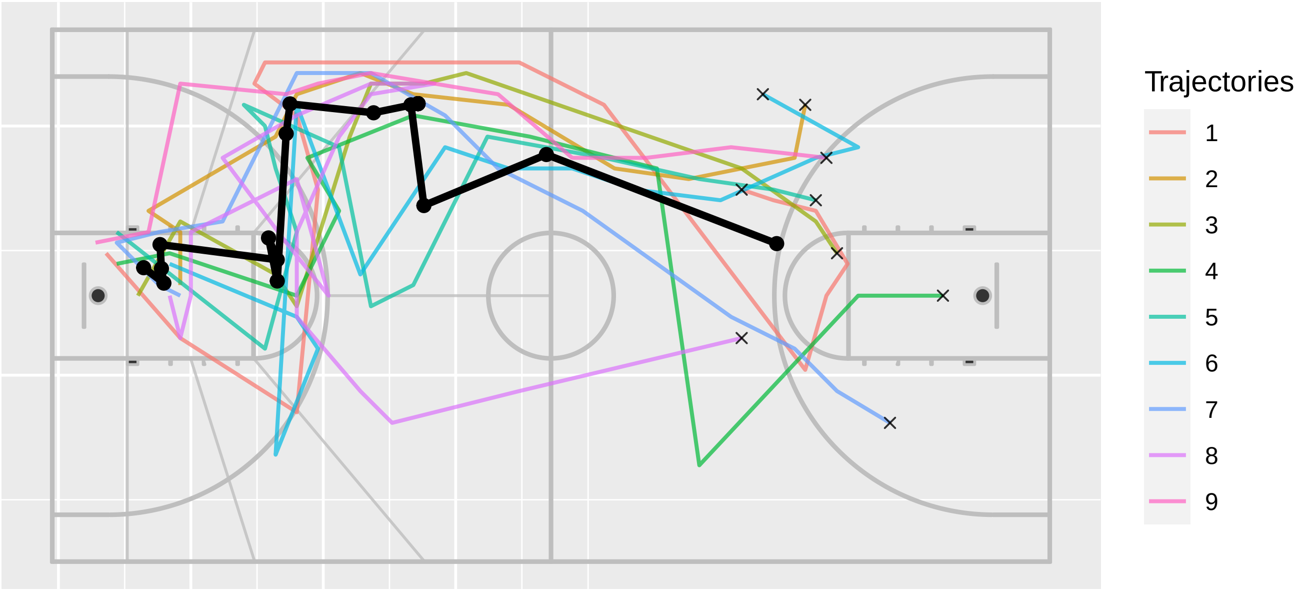

USA Area 5 Cluster 16 : SelectTrajectories

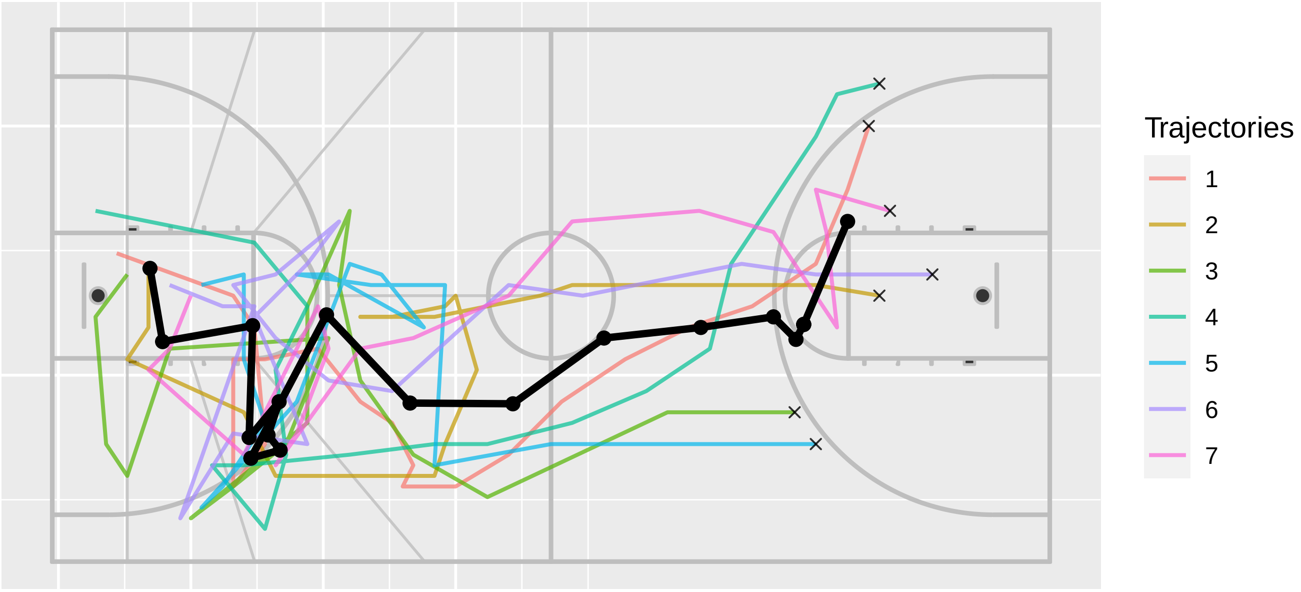

USA Area 5 Cluster 17 : SelectTrajectories

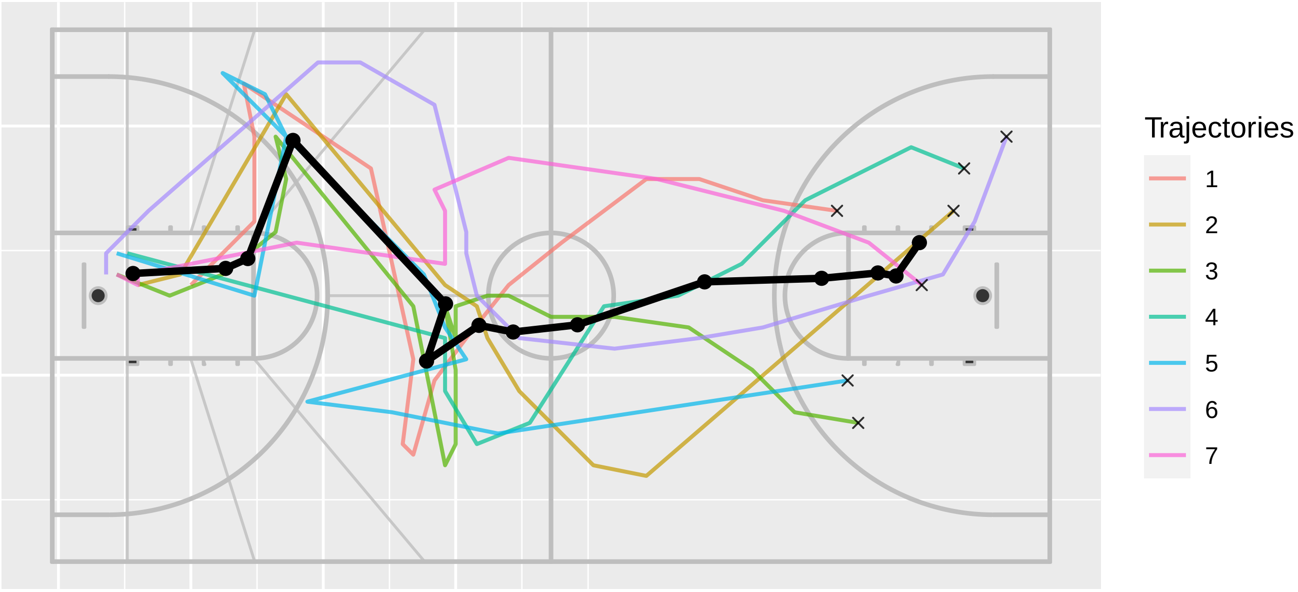

USA Area 5 Cluster 18 : SelectTrajectories

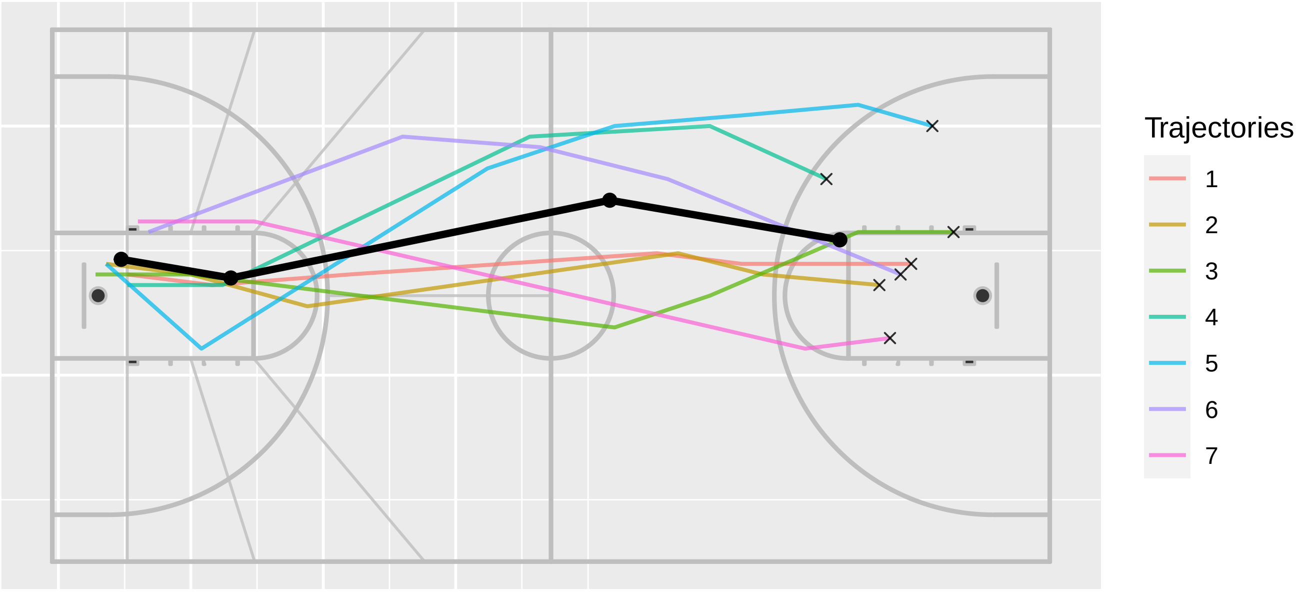

USA Area 5 Cluster 19 : SelectTrajectories

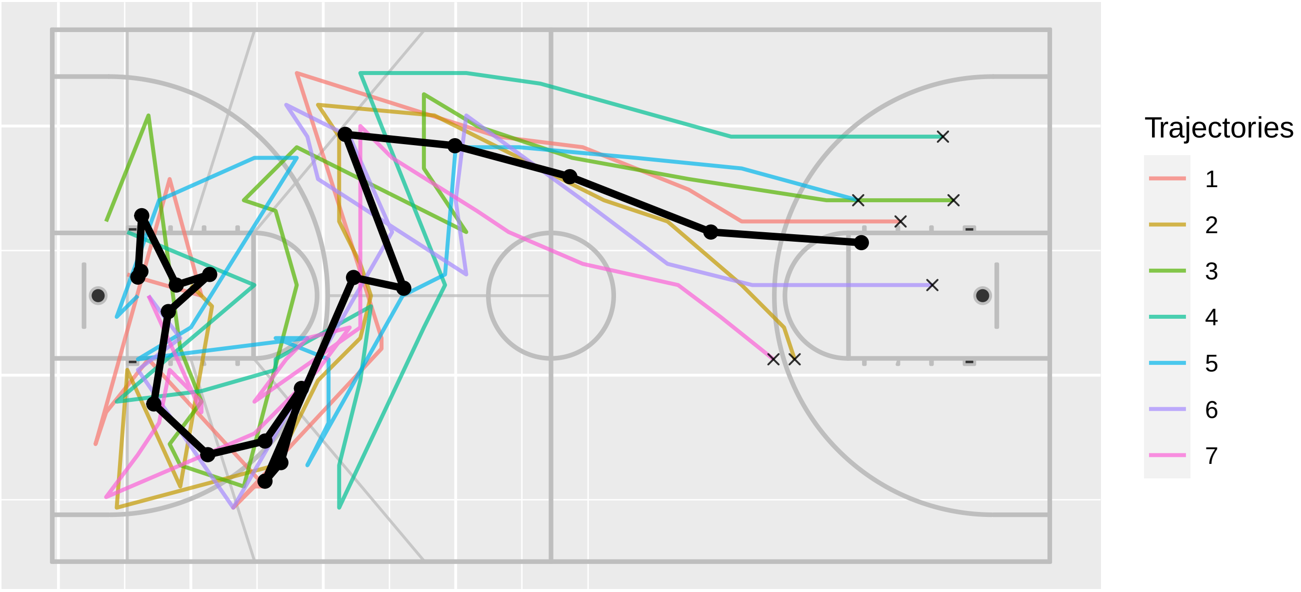

USA Area 5 Cluster 20 : SelectTrajectories

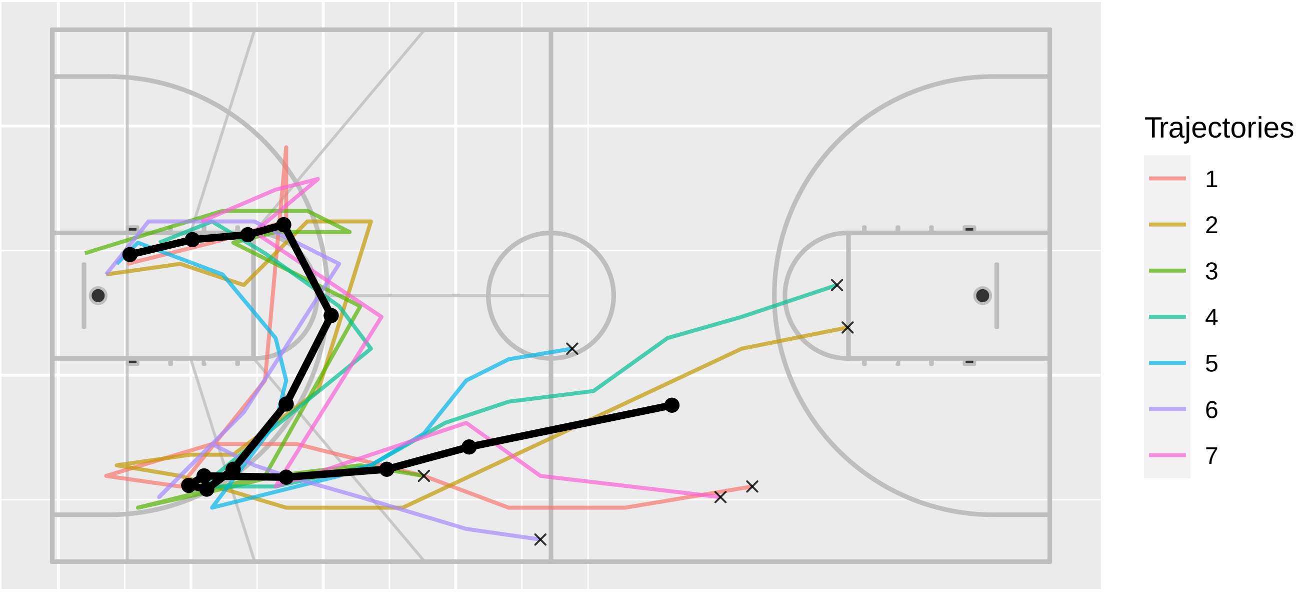

USA Area 5 Cluster 21 : SelectTrajectories

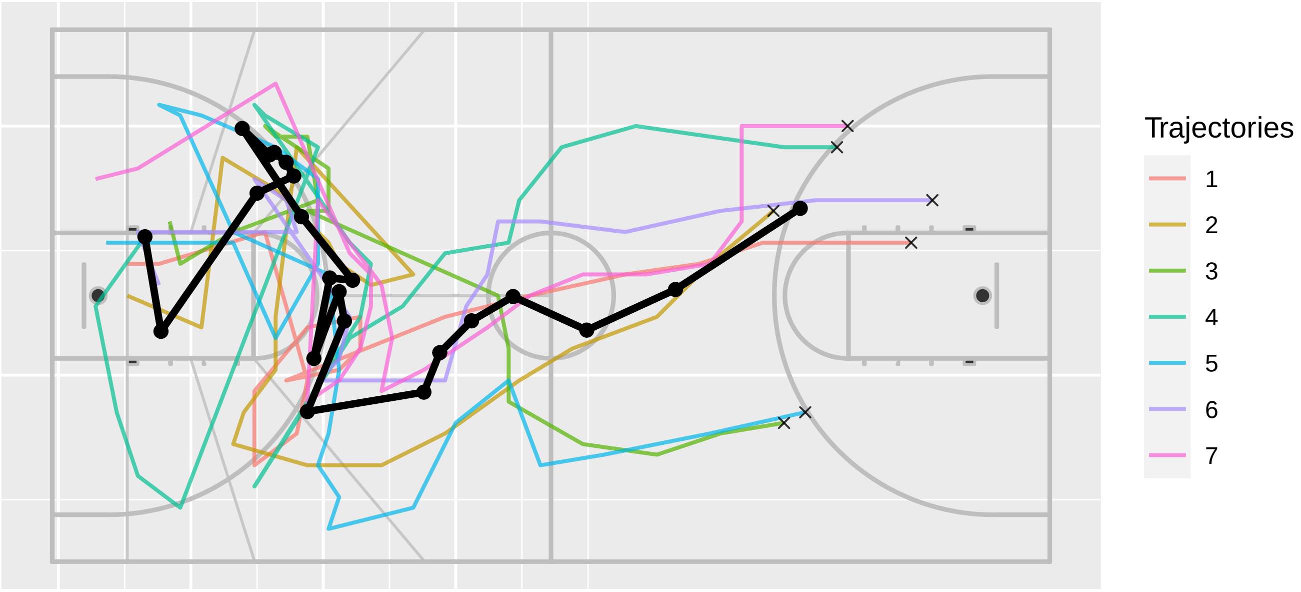

USA Area 5 Cluster 22 : SelectTrajectories

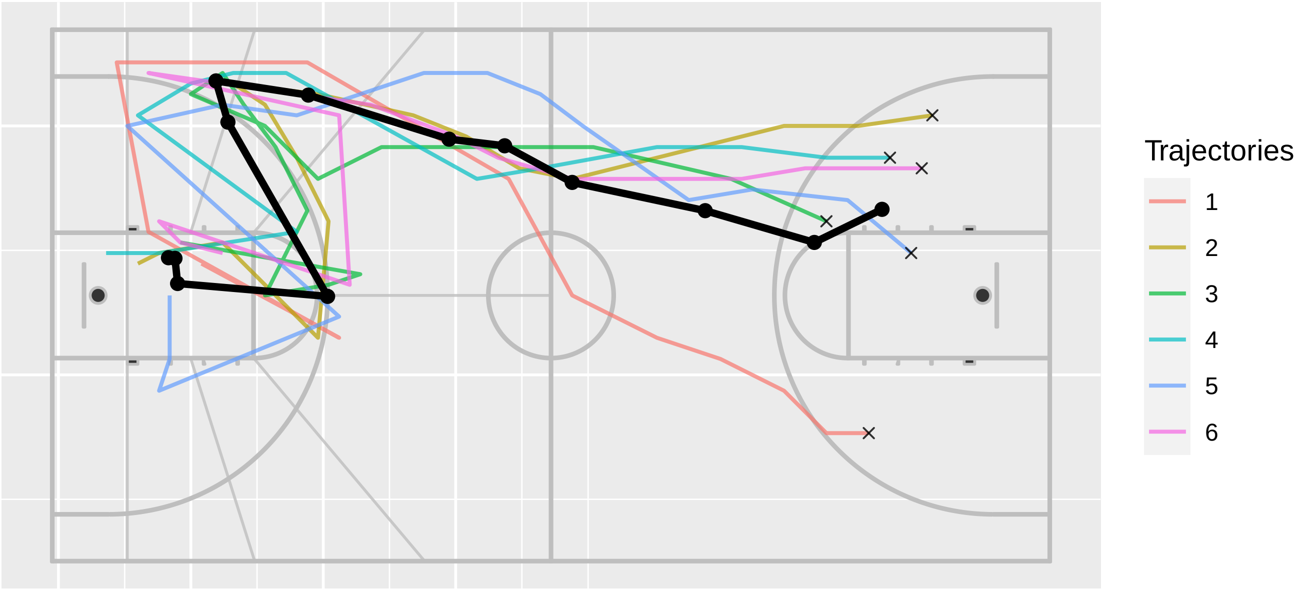

USA Area 5 Cluster 23 : SelectTrajectories

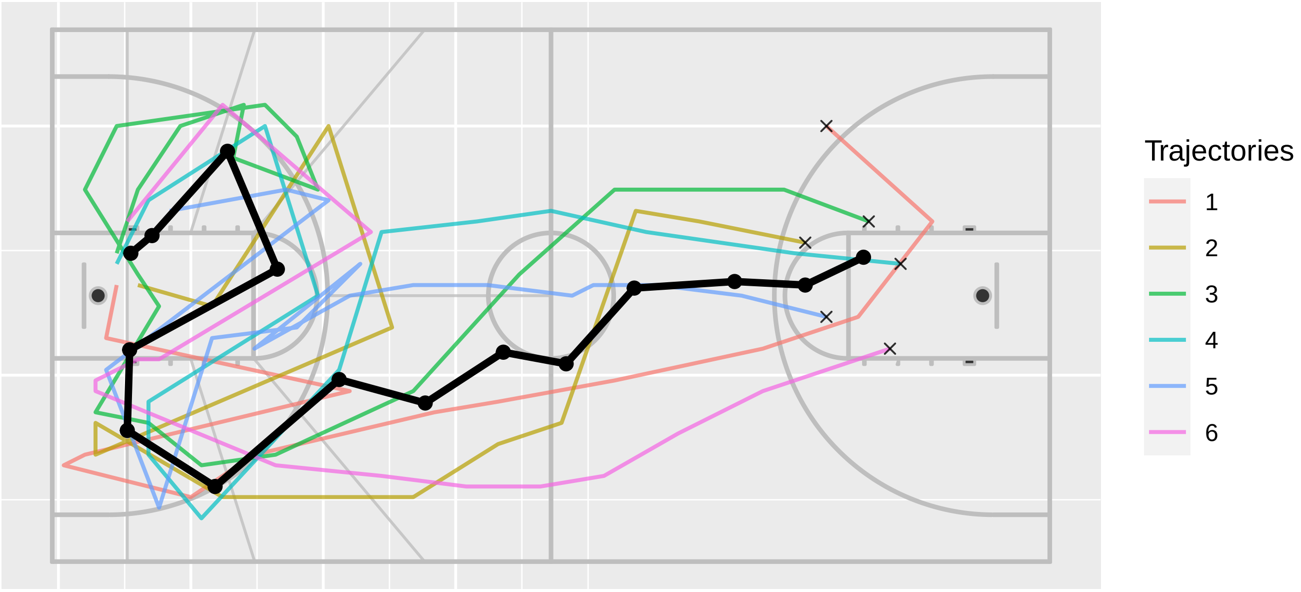

USA Area 5 Cluster 24 : SelectTrajectories

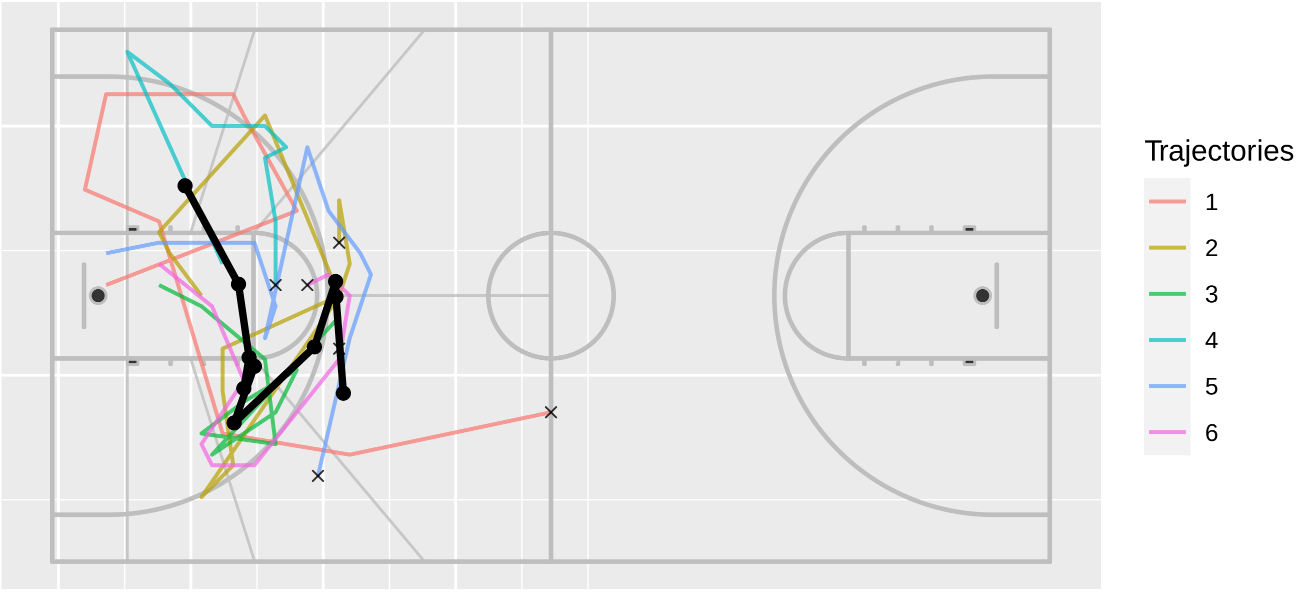

USA Area 5 Cluster 25 : SelectTrajectories

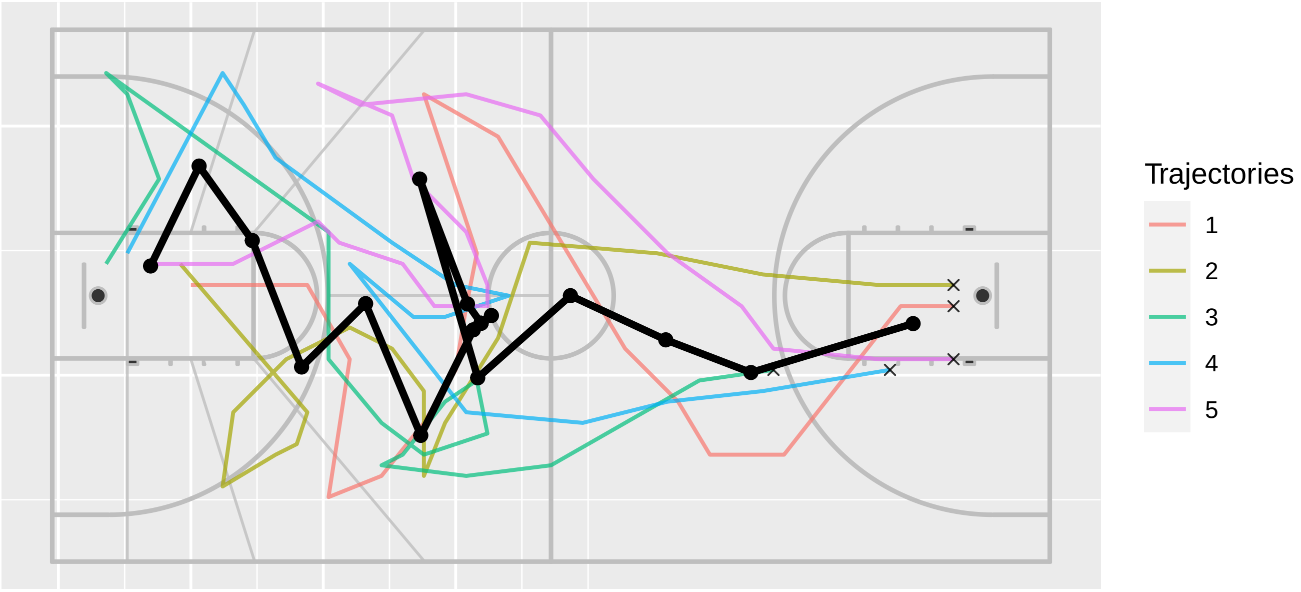

USA Area 5 Cluster 26 : SelectTrajectories

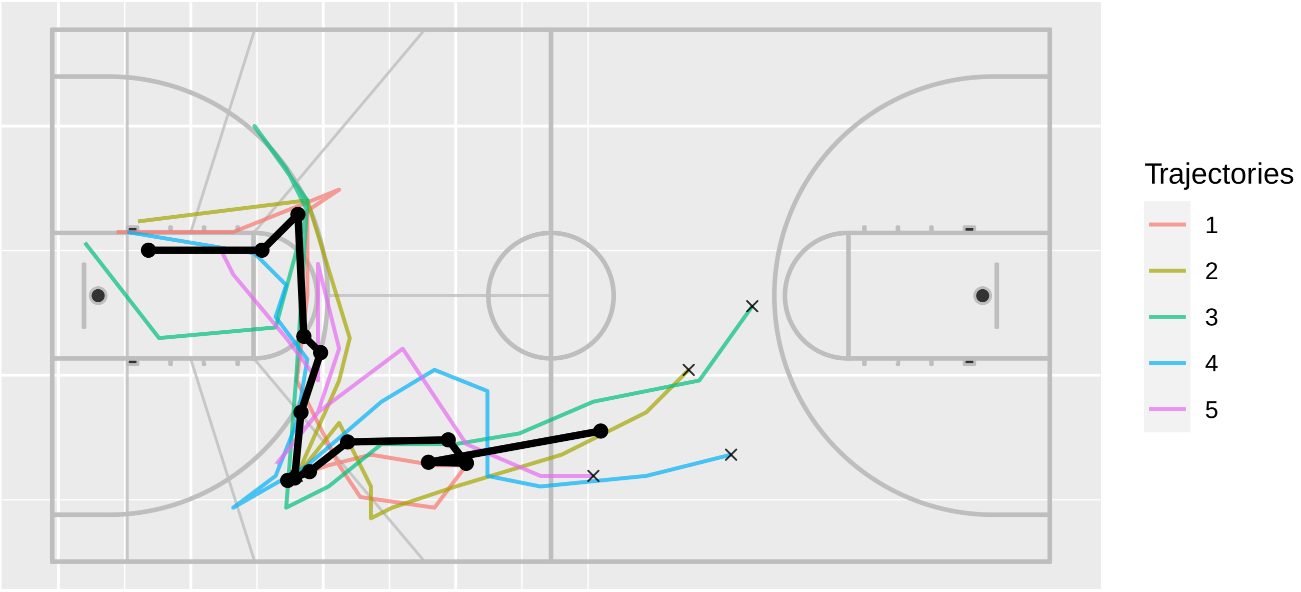

USA Area 5 Cluster 27 : SelectTrajectories

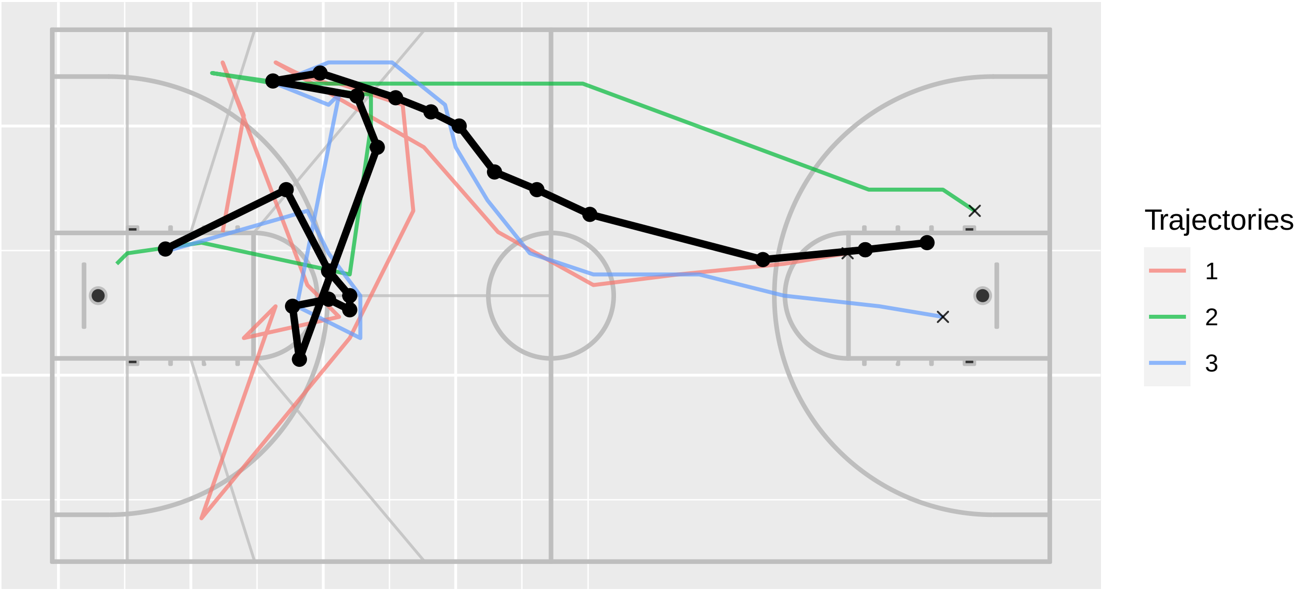

USA Area 5 Cluster 28 : SelectTrajectories

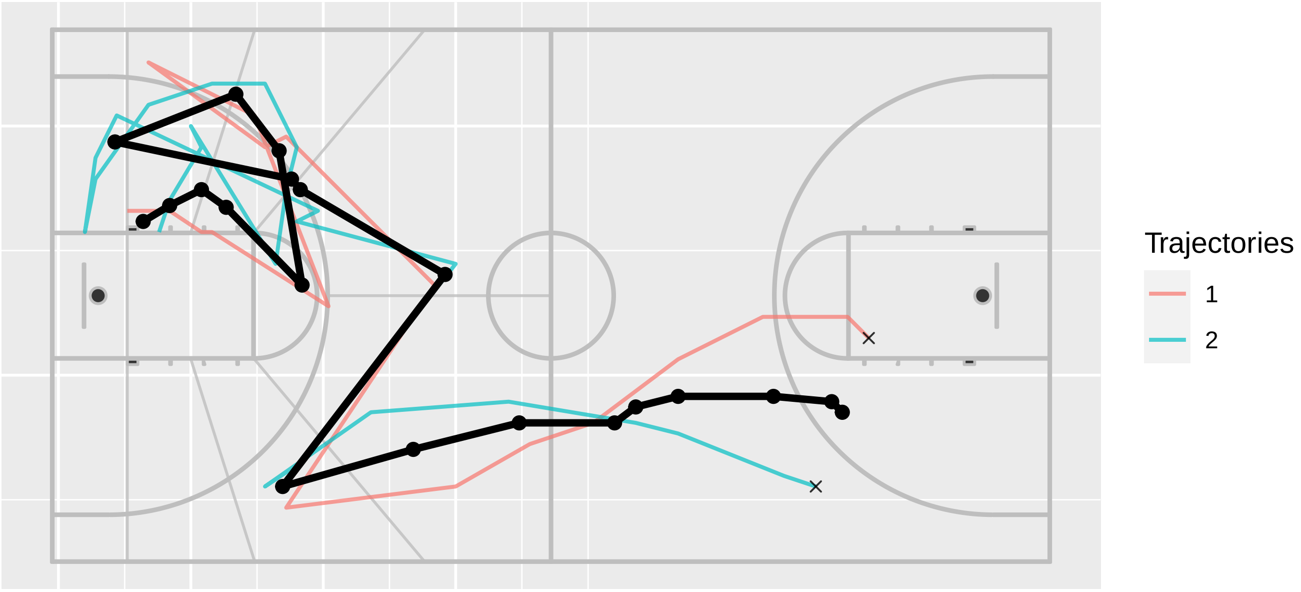

USA Area 5 Cluster 29 : SelectTrajectories

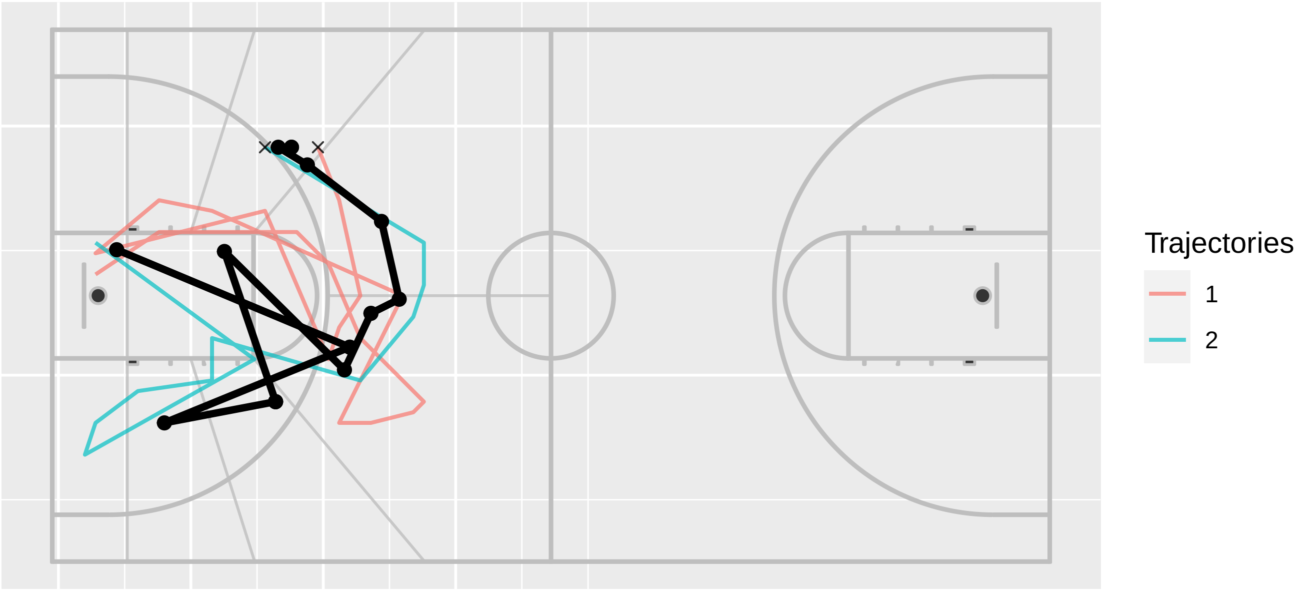

USA Area 6 Cluster 1 : SelectTrajectories

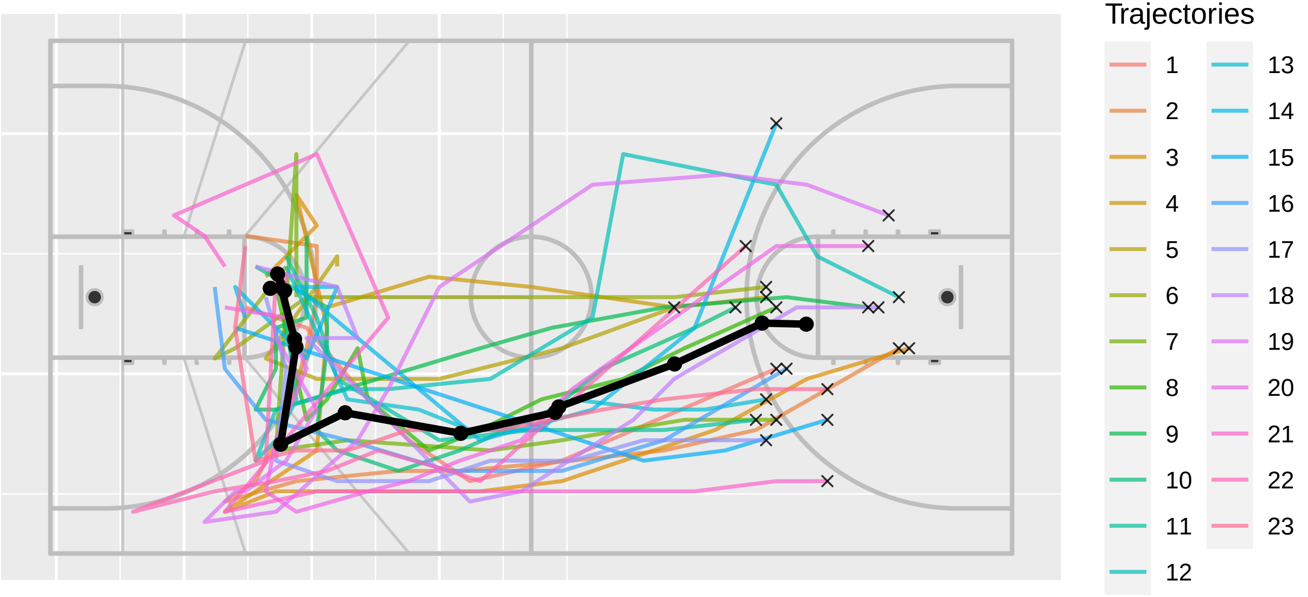

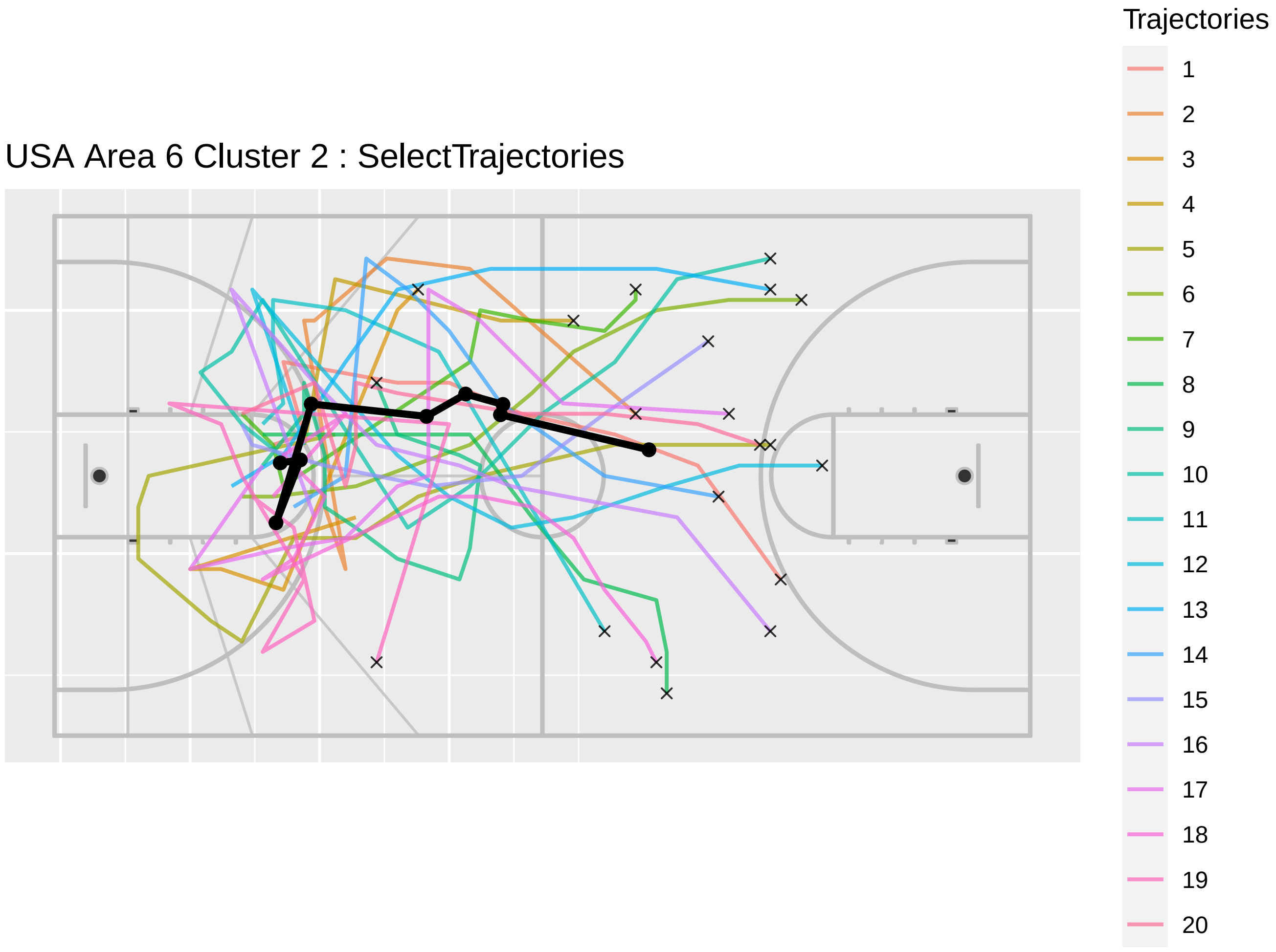
USA Area 6 Cluster 2 : SelectTrajectories
Trajectories
1
2
3
4
5
6
7
8
9
10
11
12
13
14
15
16
17
18
19
20

## USA Area 6 Cluster 3 : SelectTrajectories

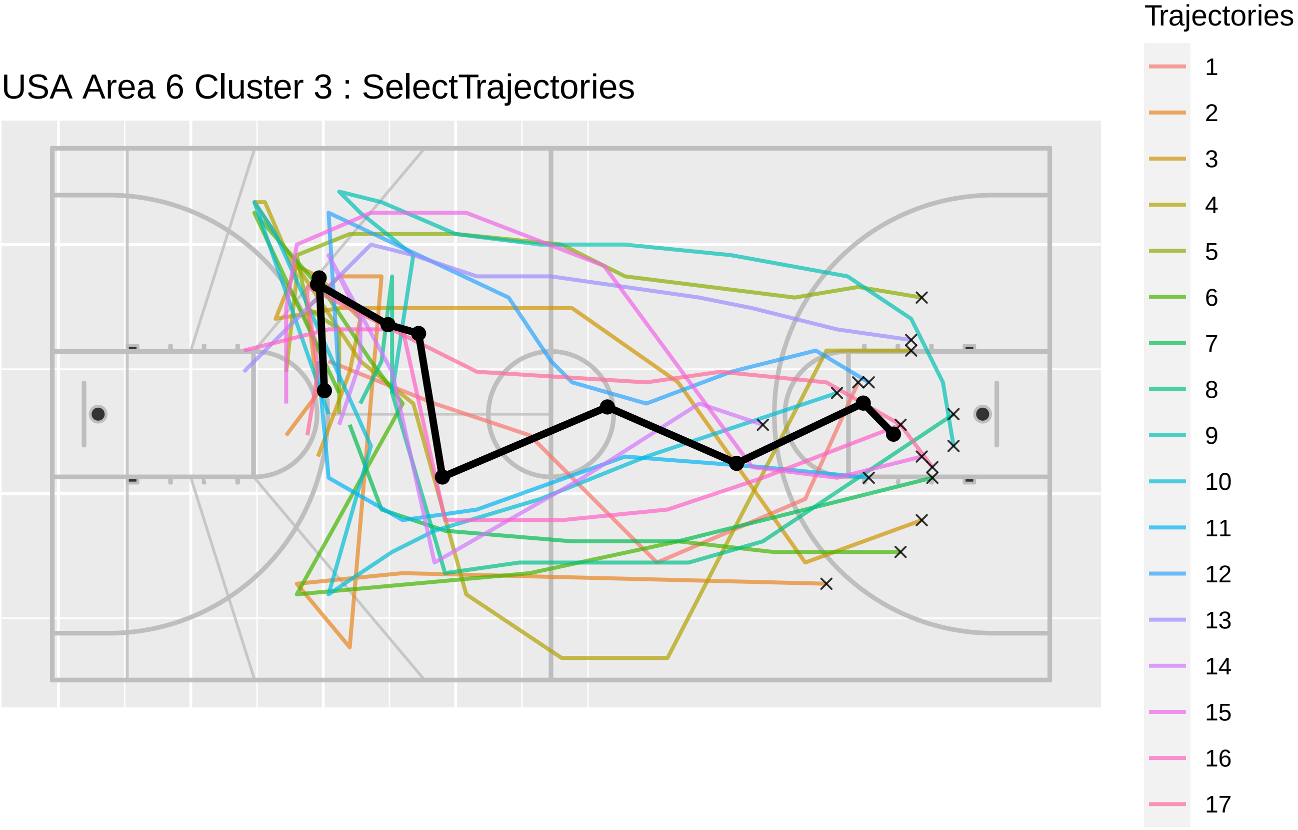

USA Area 6 Cluster 4 : SelectTrajectories

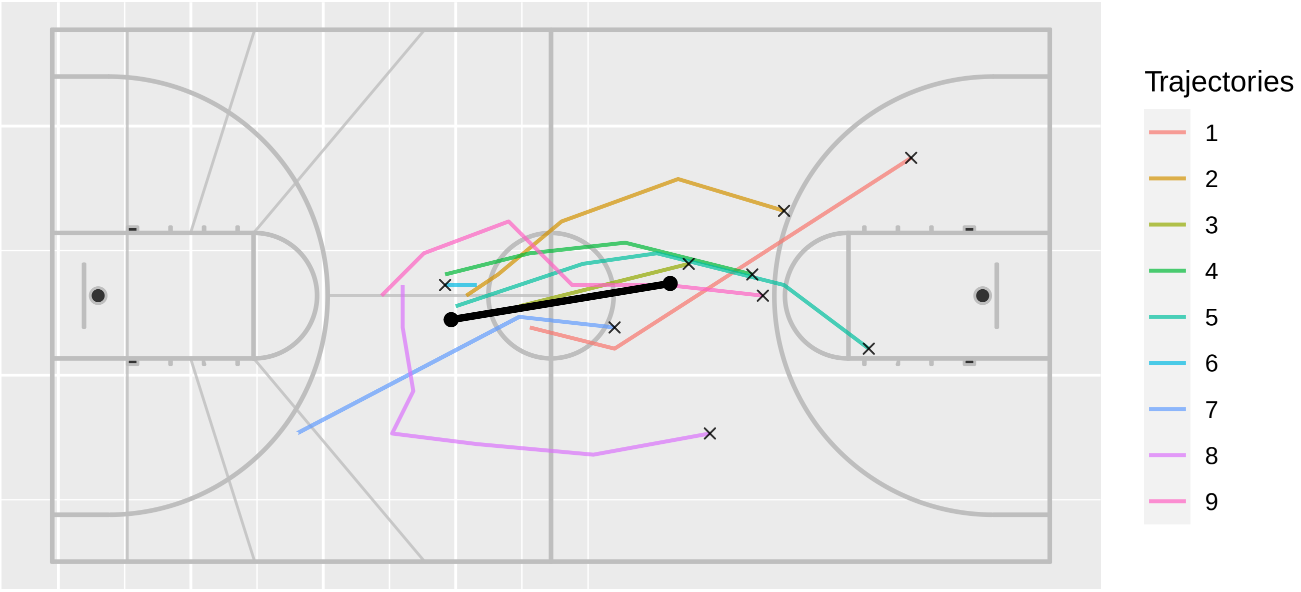

USA Area 6 Cluster 5 : SelectTrajectories

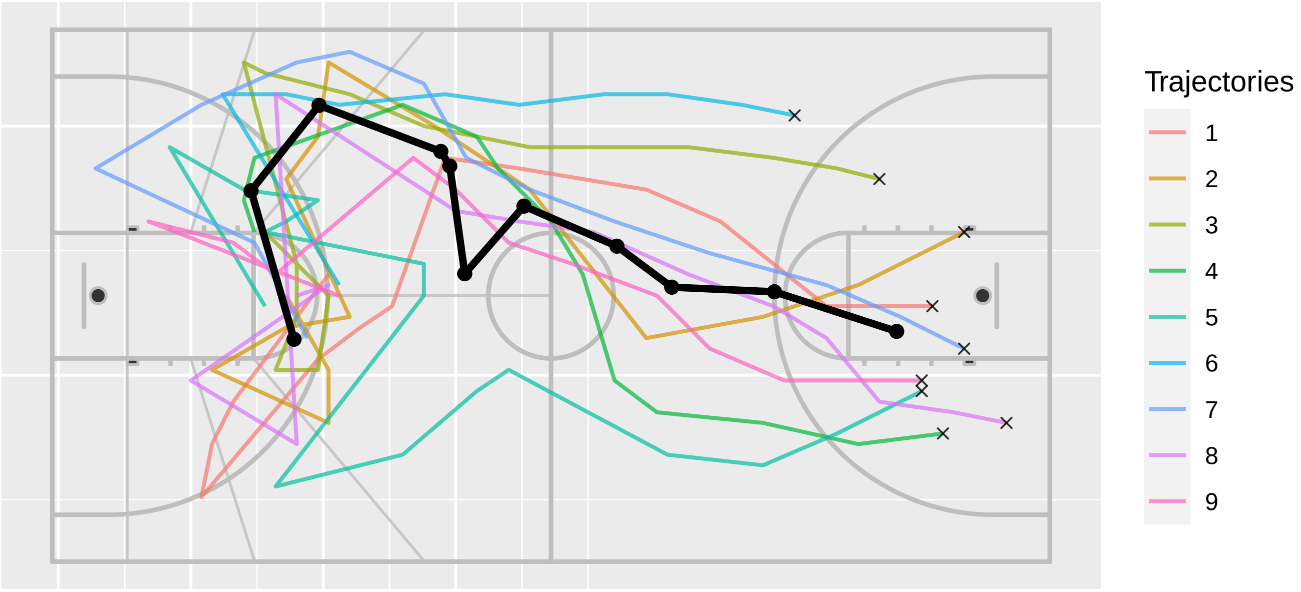

USA Area 6 Cluster 6 : SelectTrajectories

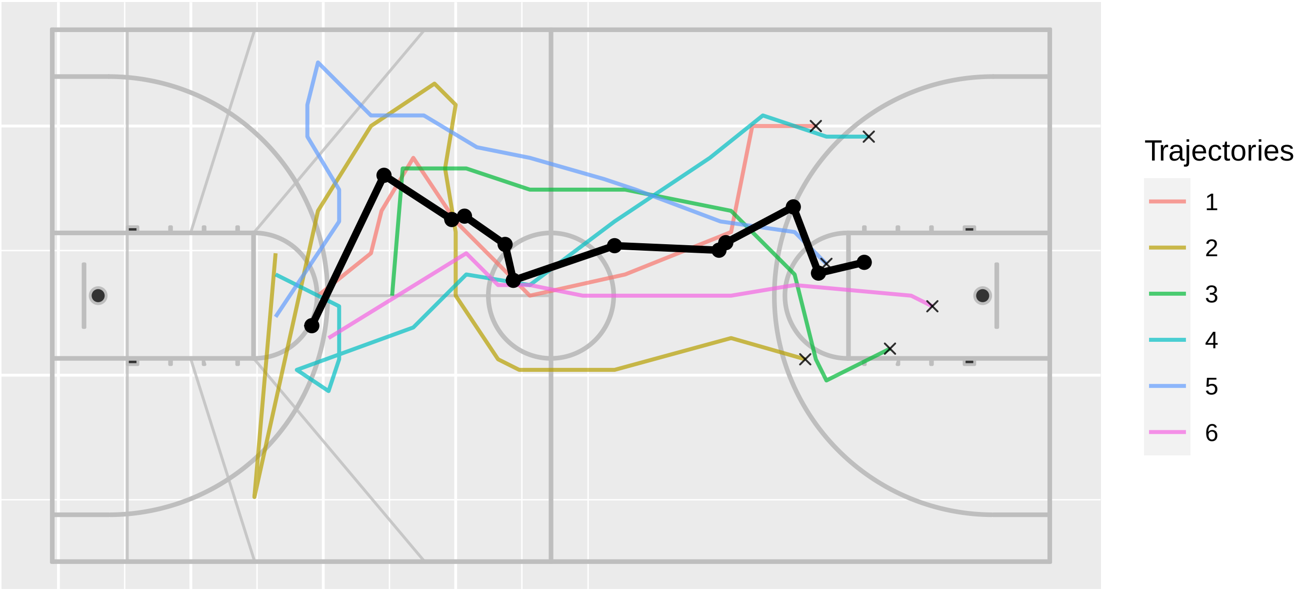

USA Area 6 Cluster 7 : SelectTrajectories

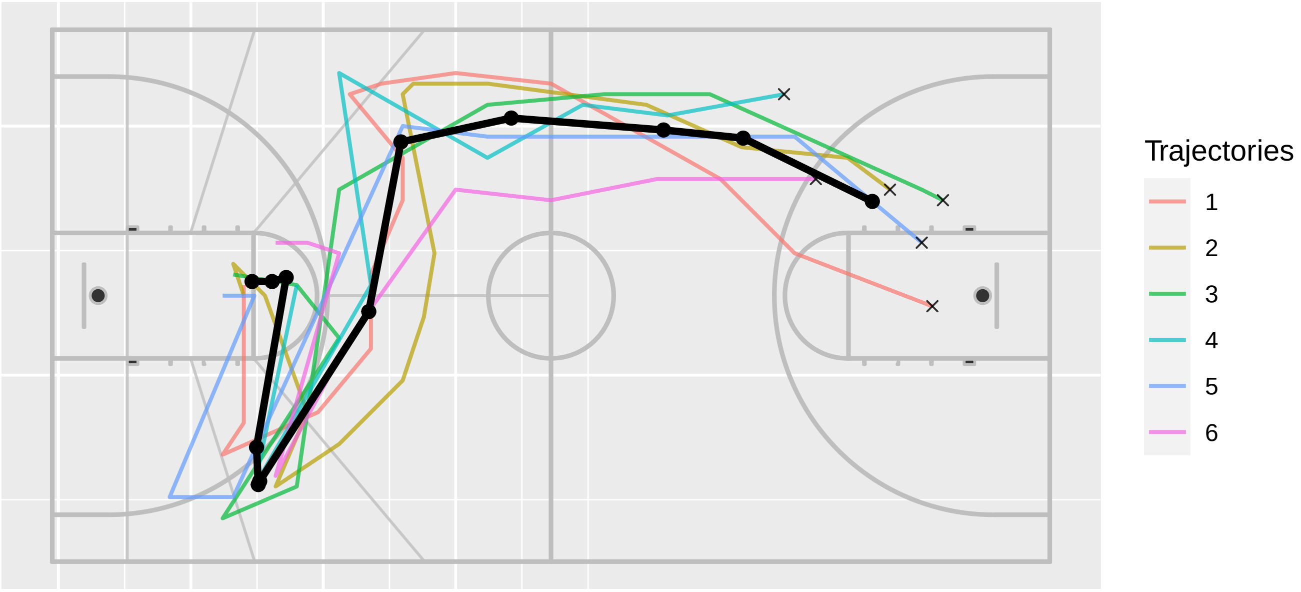

USA Area 6 Cluster 8 : SelectTrajectories

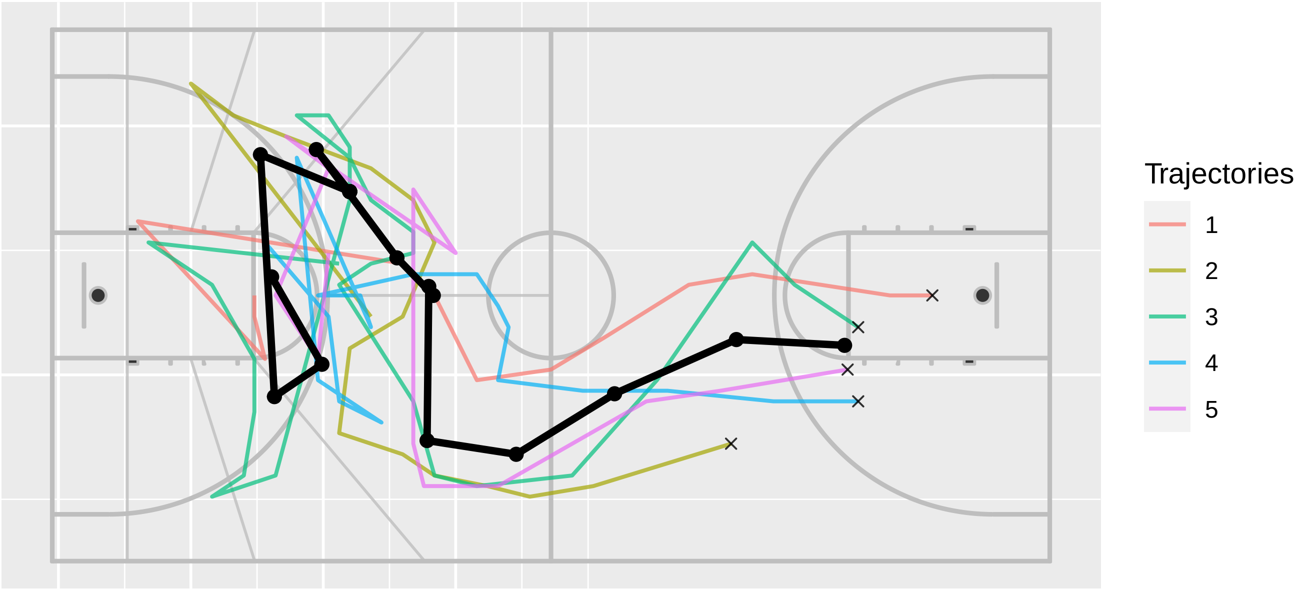

USA Area 6 Cluster 9 : SelectTrajectories

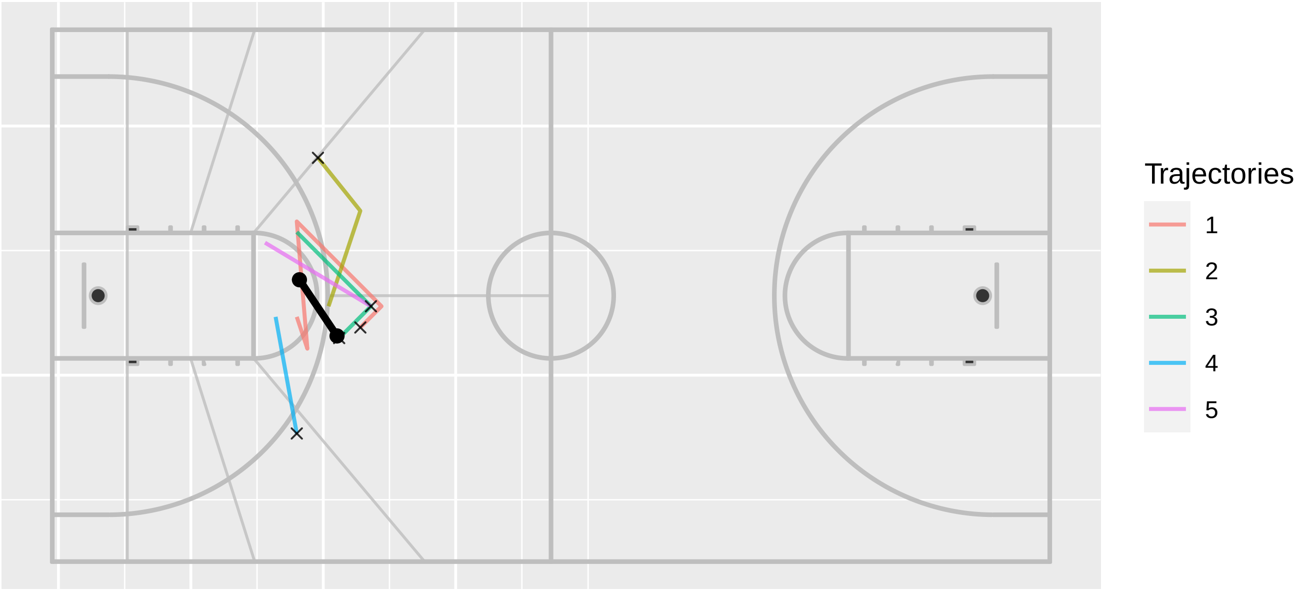

USA Area 6 Cluster 10 : SelectTrajectories

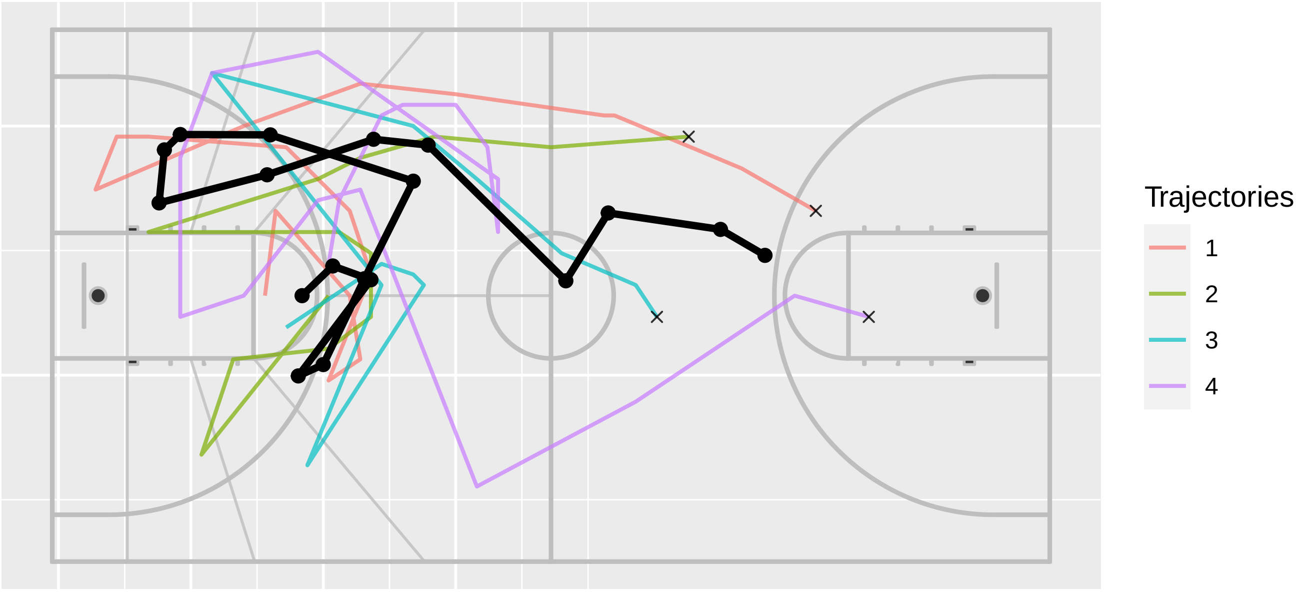

USA Area 6 Cluster 11 : SelectTrajectories

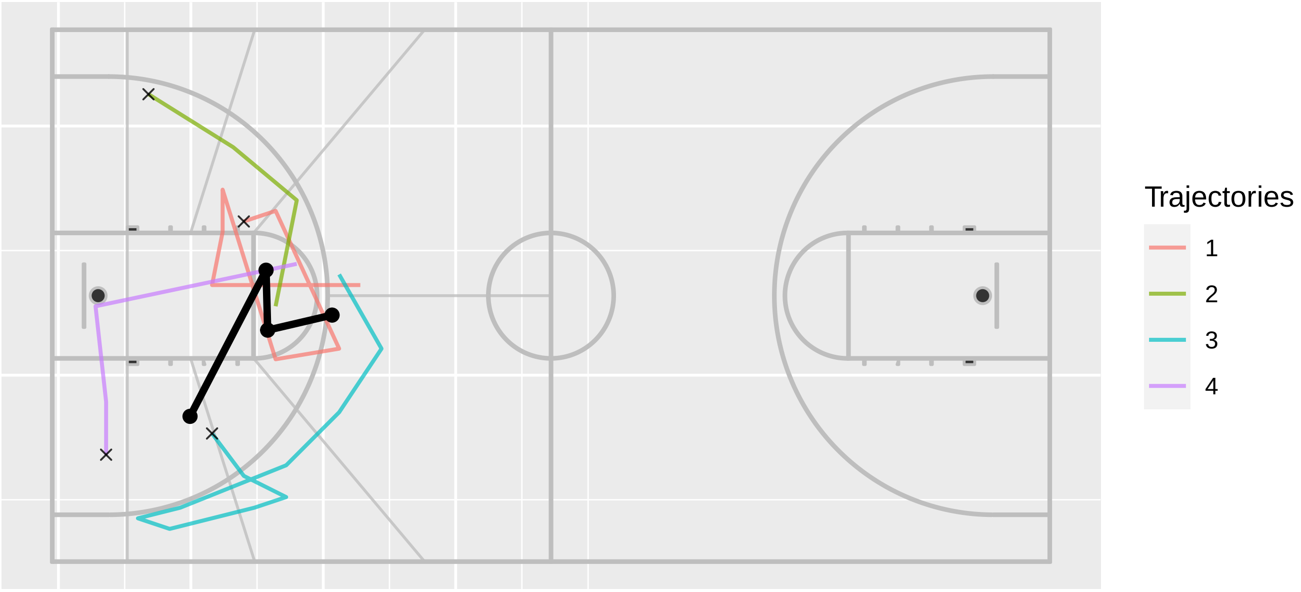

USA Area 6 Cluster 12 : SelectTrajectories

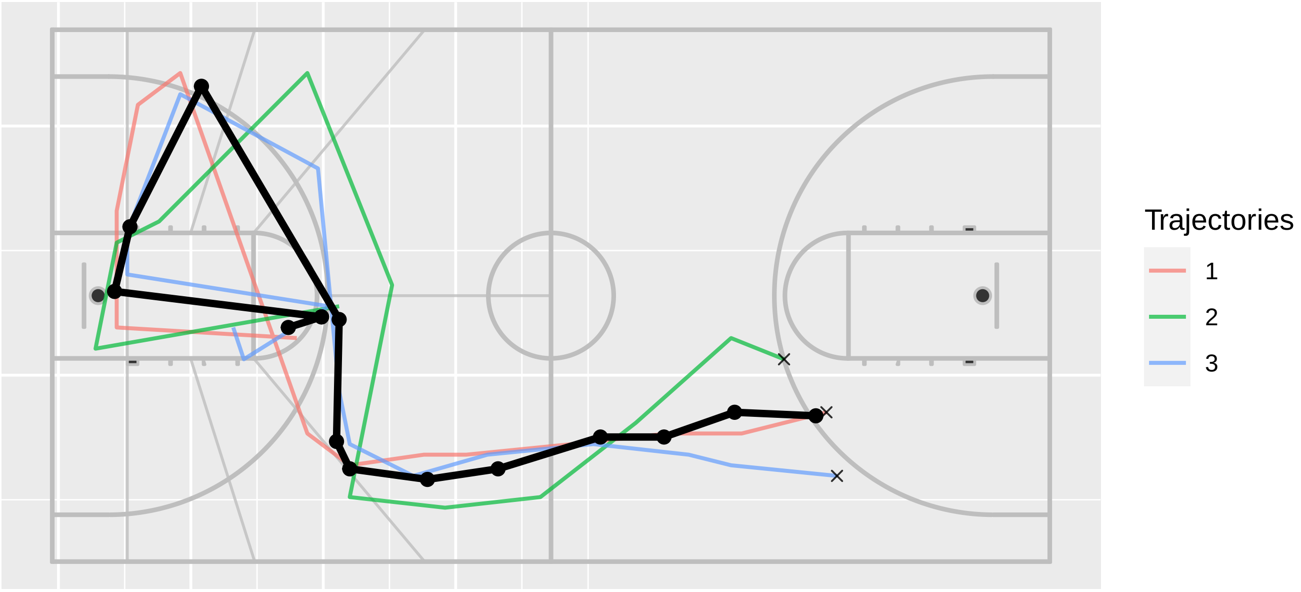

USA Area 6 Cluster 13 : SelectTrajectories

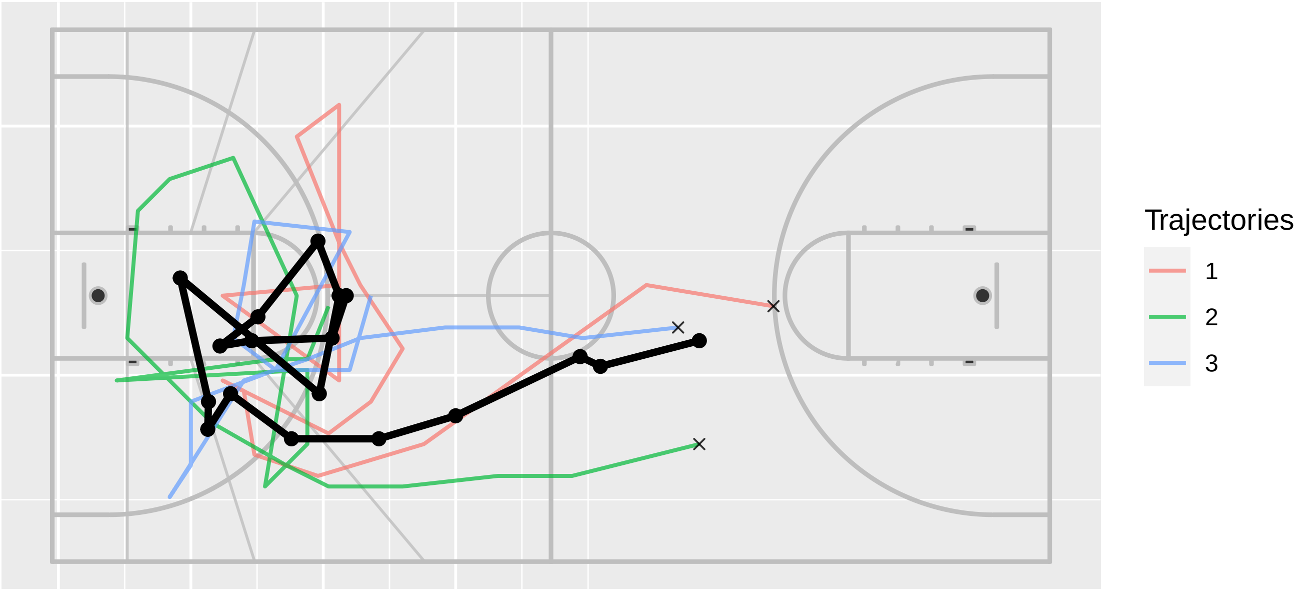

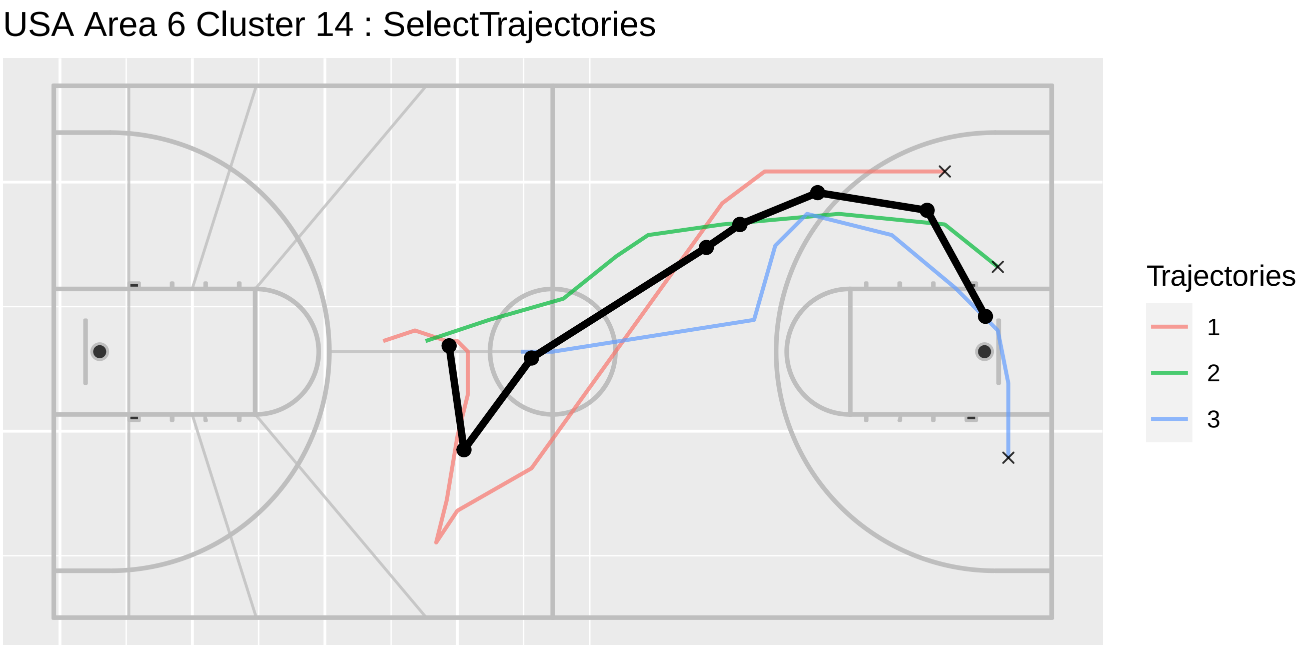
USA Area 6 Cluster 14 : SelectTrajectories
Trajectories
1
2
3

USA Area 6 Cluster 15 : SelectTrajectories

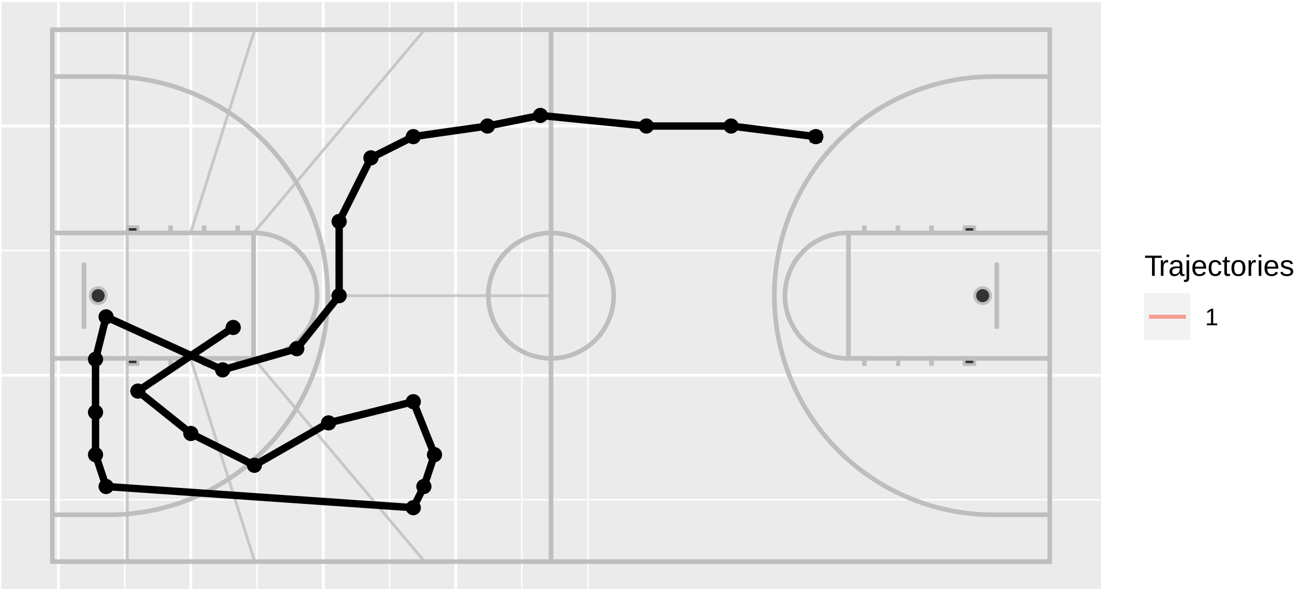

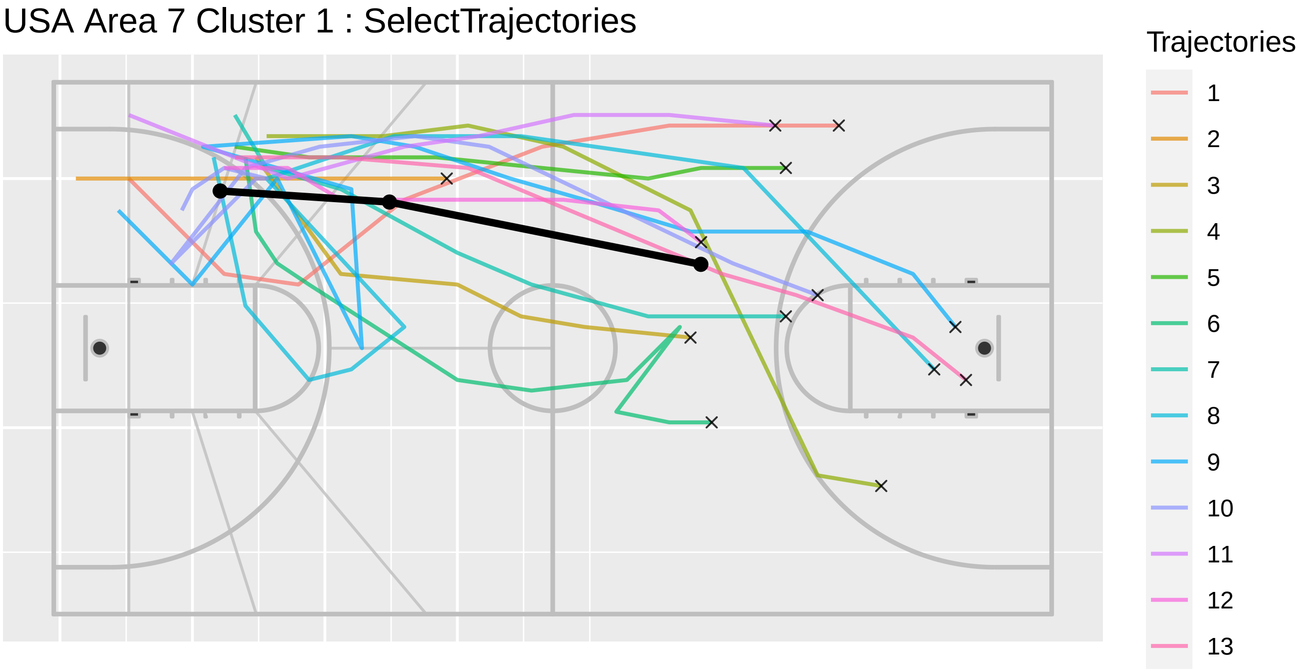
USA Area 7 Cluster 1 : SelectTrajectories
Trajectories
1
2
3
4
5
6
7
8
9
10
11
12
13

USA Area 7 Cluster 2 : SelectTrajectories

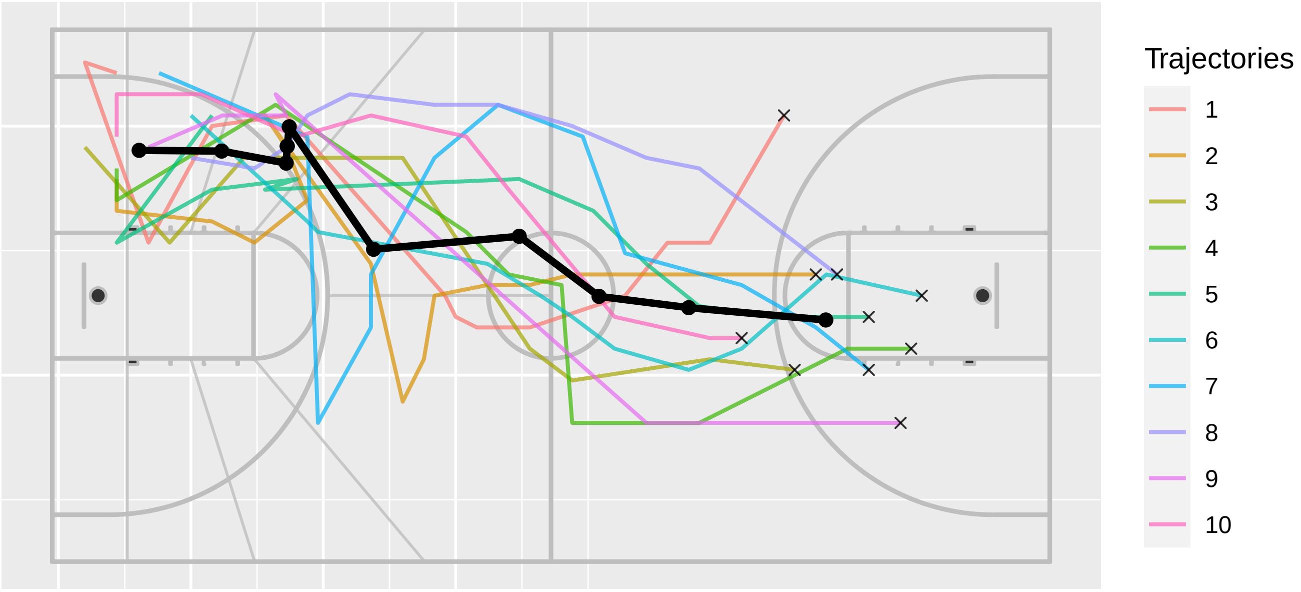

USA Area 7 Cluster 3 : SelectTrajectories

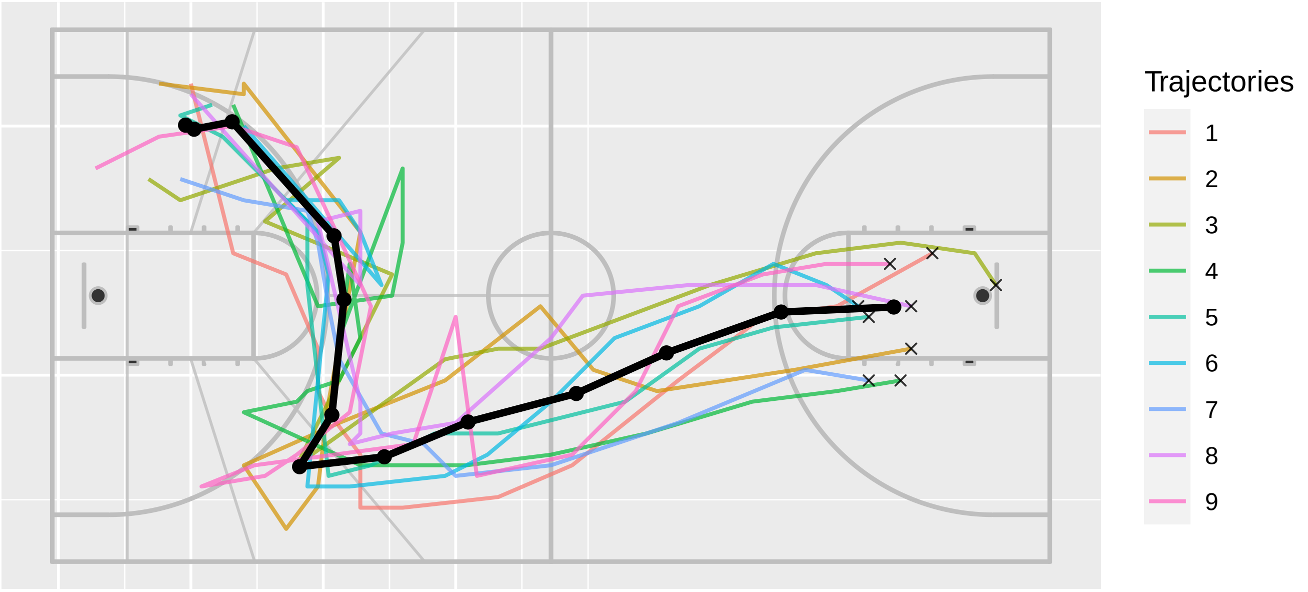

USA Area 7 Cluster 4 : SelectTrajectories

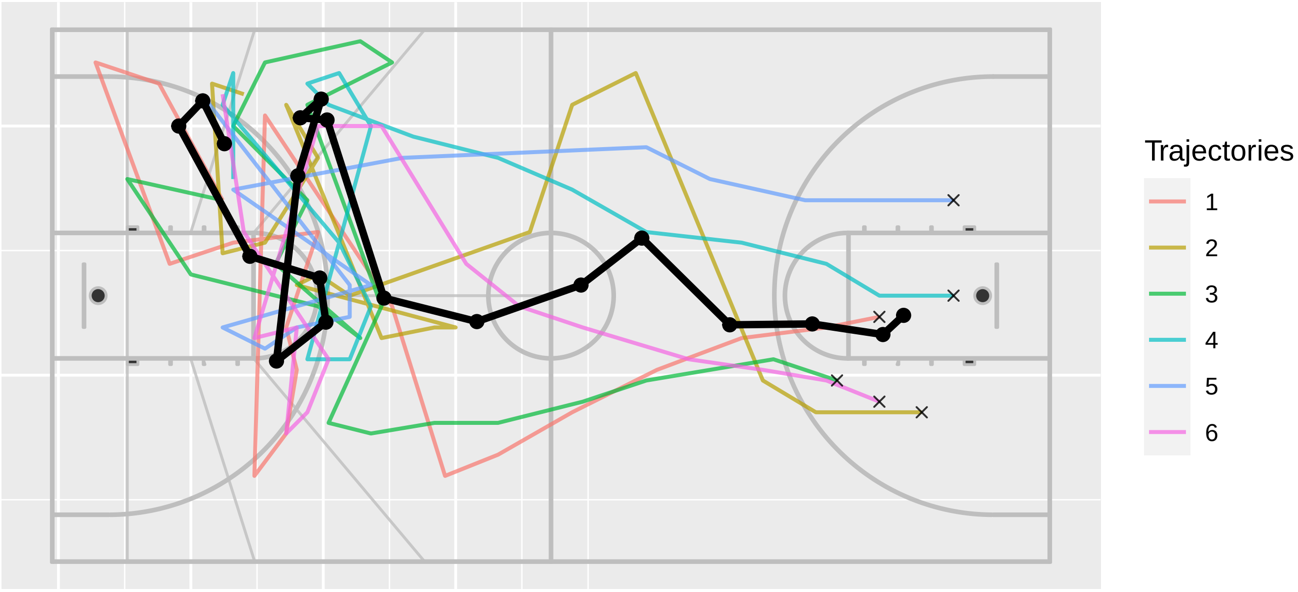

USA Area 7 Cluster 5 : SelectTrajectories

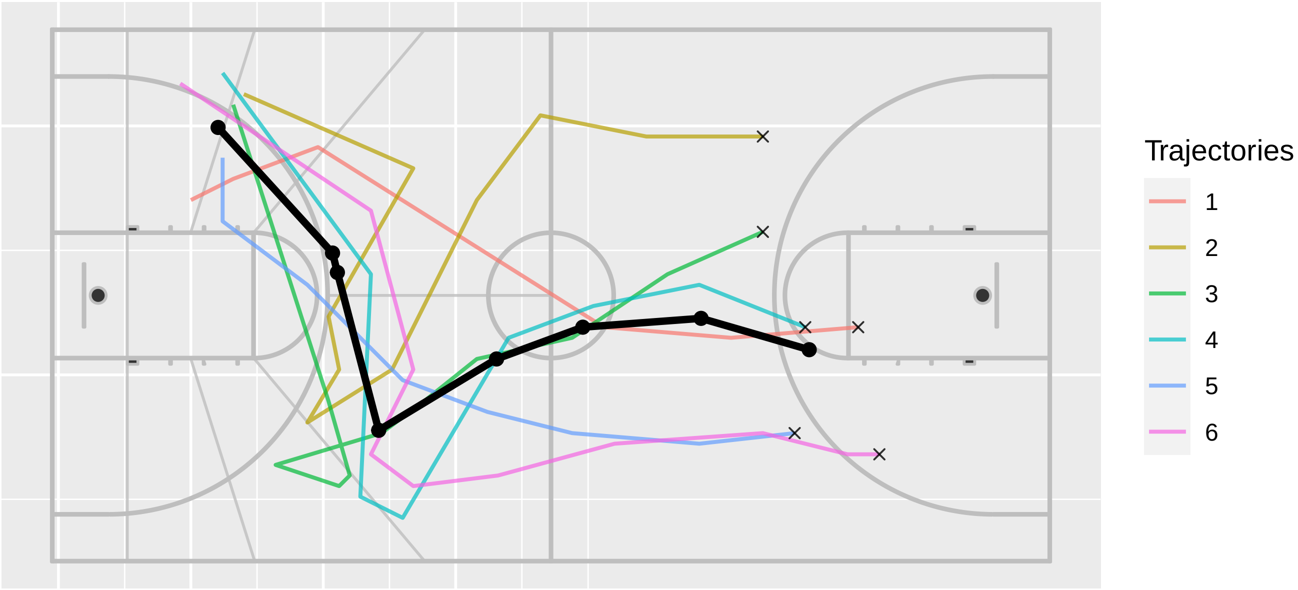

## USA Area 7 Cluster 6 : SelectTrajectories

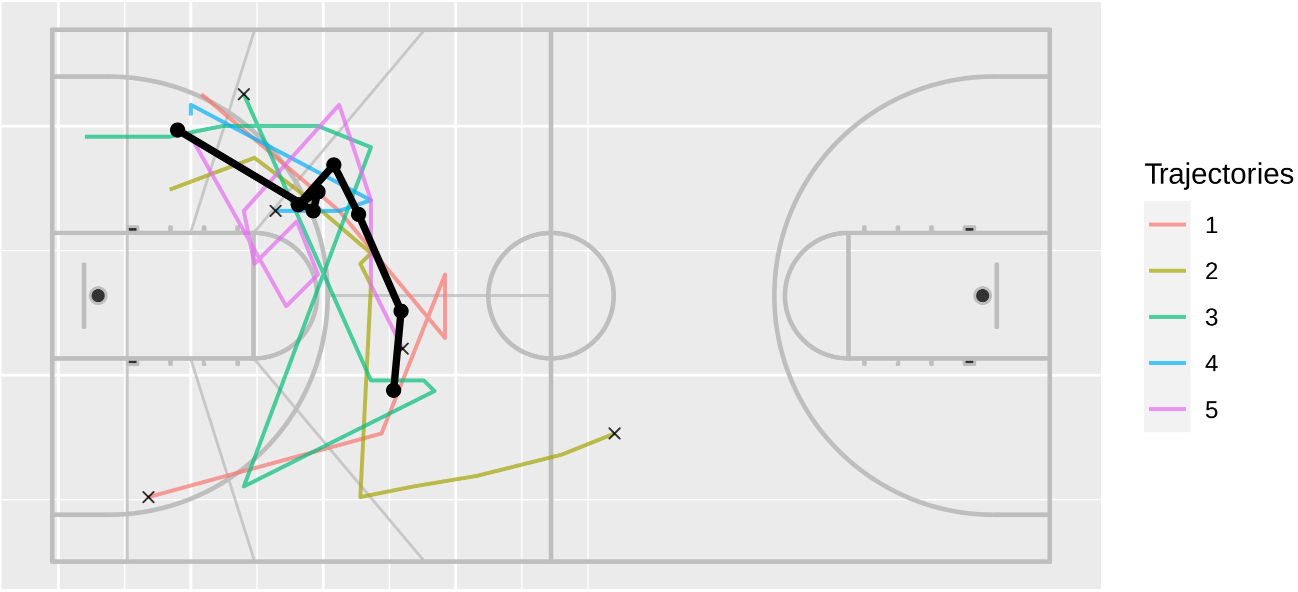

USA Area 7 Cluster 7 : SelectTrajectories

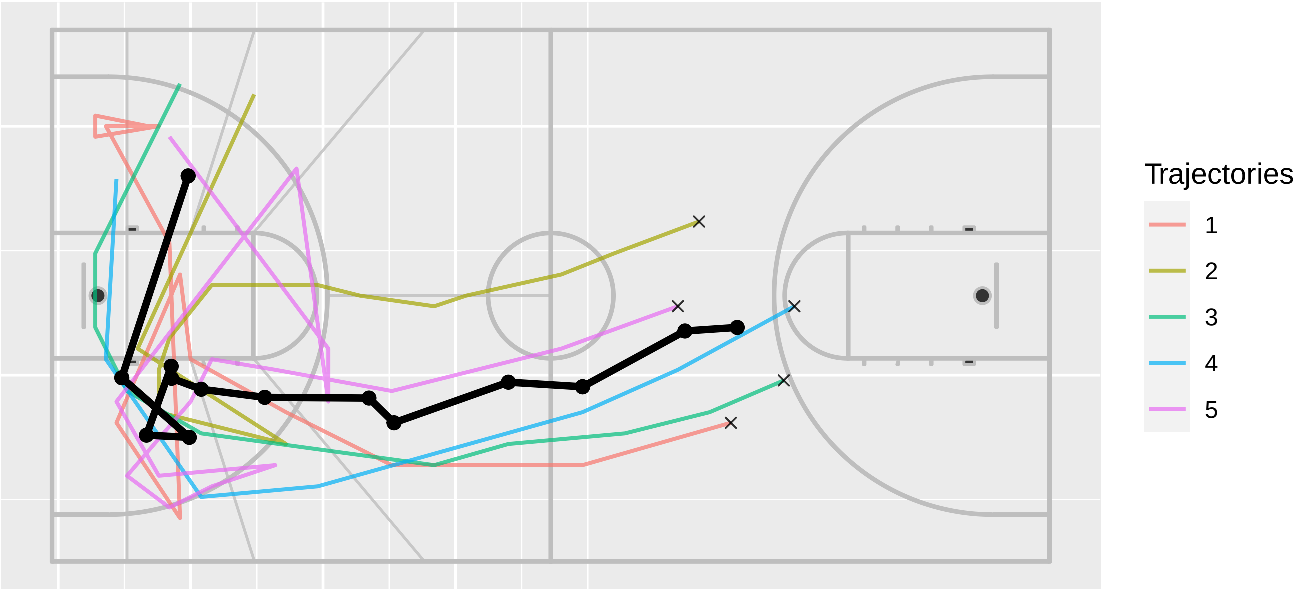

USA Area 7 Cluster 8 : SelectTrajectories

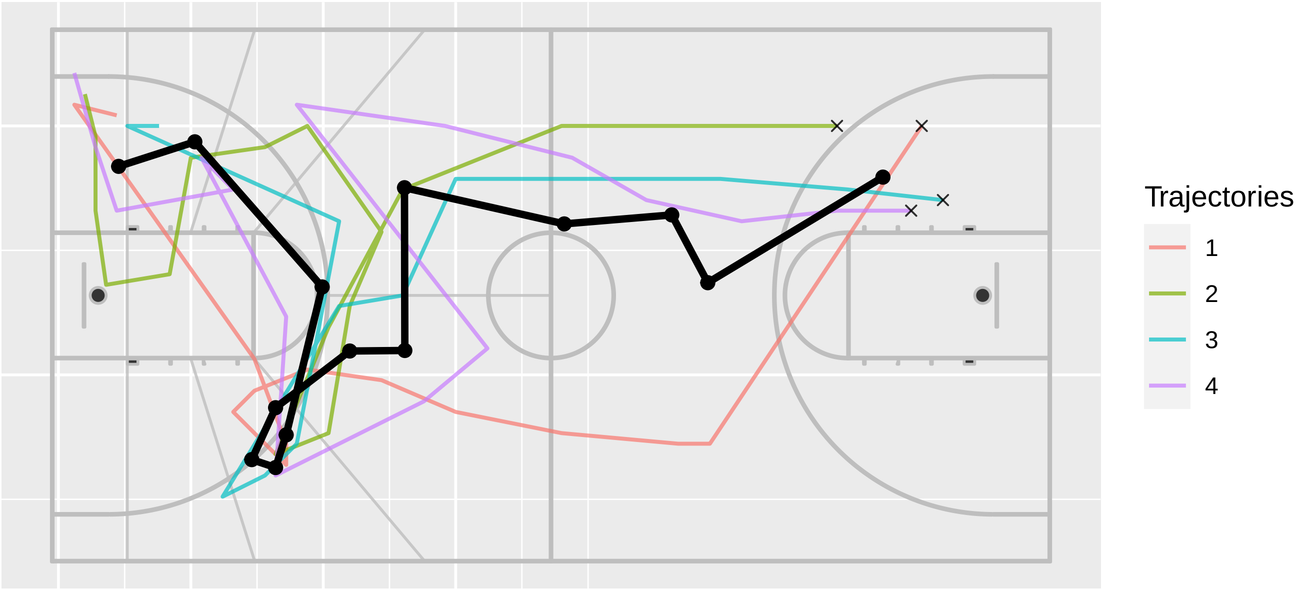

USA Area 7 Cluster 9 : SelectTrajectories

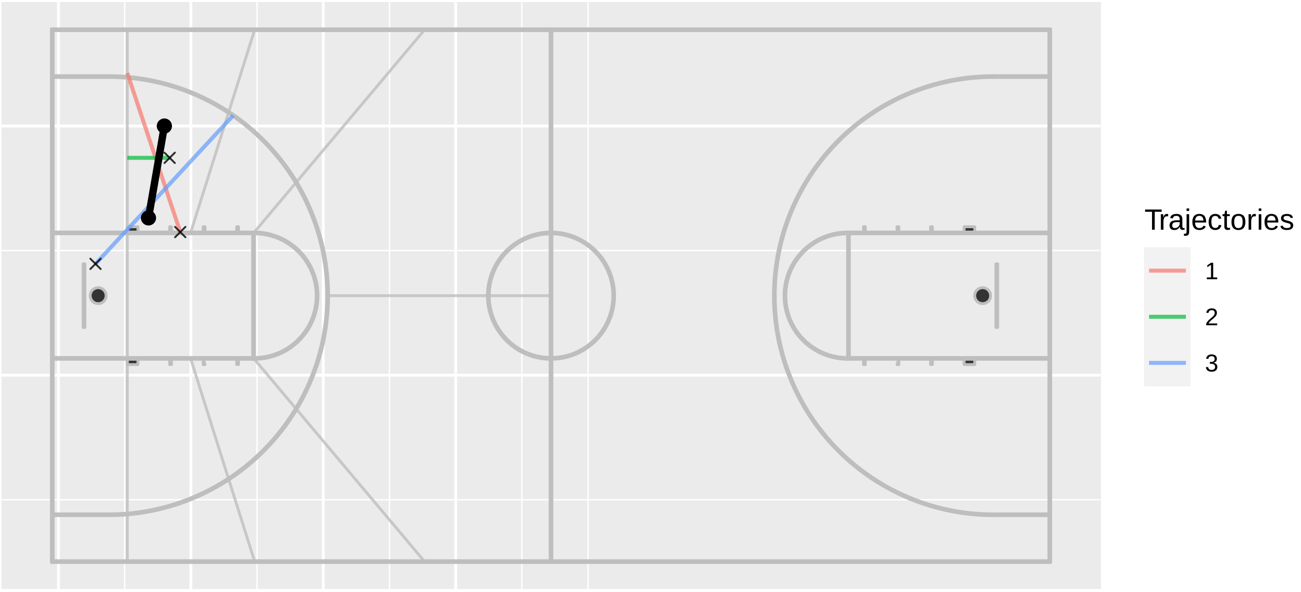

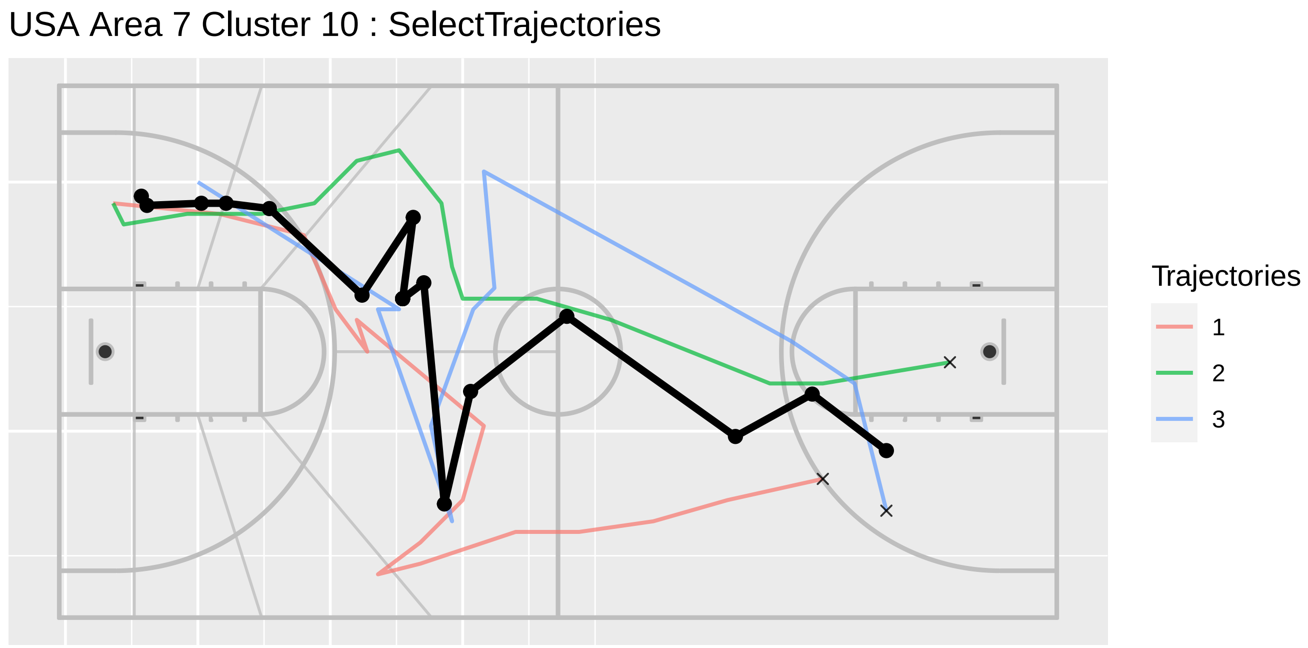
USA Area 7 Cluster 10 : SelectTrajectories
Trajectories
1
2
3

USA Area 7 Cluster 11 : SelectTrajectories

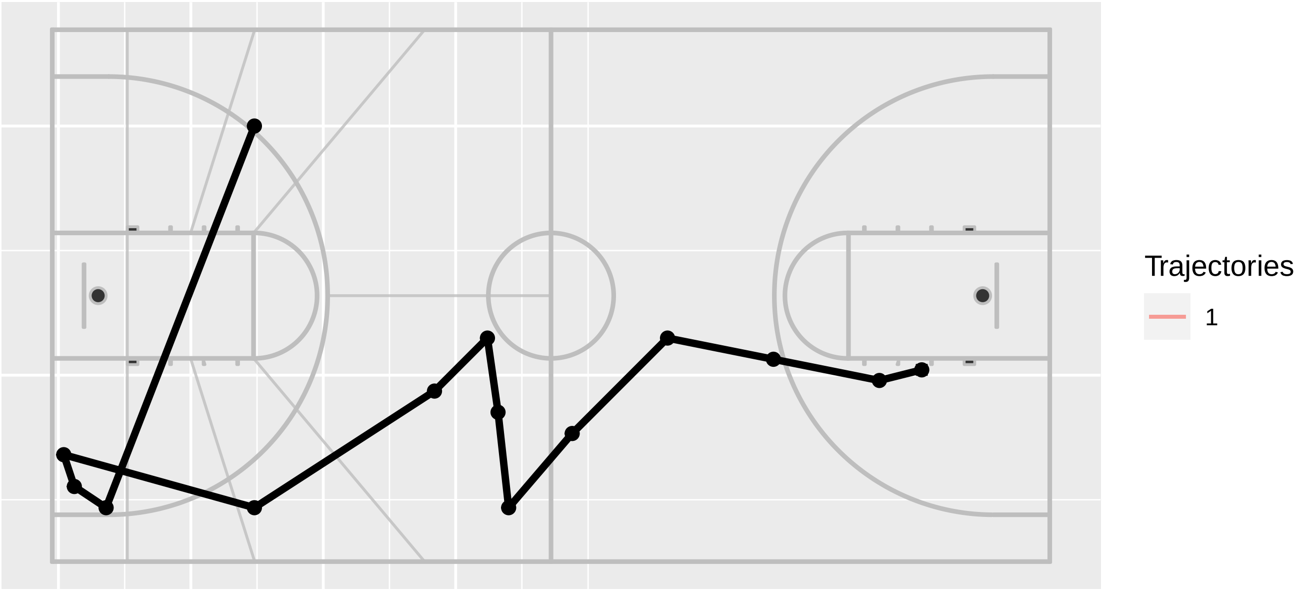

## USA Area 7 Cluster 12 : SelectTrajectories

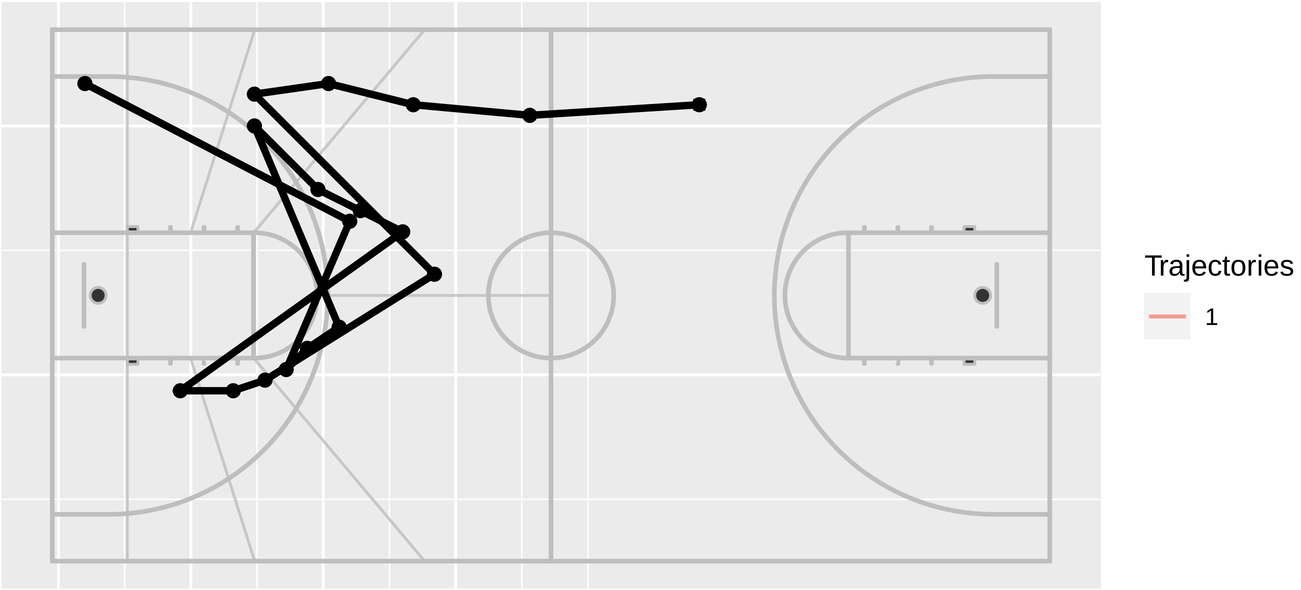

Supplement: S10 Appendix — (PDF) [file pone.0272848.s010.pdf]
