# Supplementary material for: Classifying ball trajectories in invasion sports using dynamic time warping: A basketball case study
Source: PLoS One. 2022 Oct 20;17(10):e0272848. doi: 10.1371/journal.pone.0272848 (PMC9584368; doi:10.1371/journal.pone.0272848)
Supplement: Attachment — Submitted filename: Review on Manuscript PONE-D-21-32152.pdf [file pone.0272848.s013.pdf]

Review on Manuscript: **"Classifying ball trajectories in invasion sports using Dynamic Time Warping: a basketball case study".**

In this manuscript, the authors presented a method to compare and to classify ball trajectories based on Dynamic Time Warping (DTW). The paper is well-written with some arguments on how DTW solved problem of measuring distance between two trajectories that were misaligned in time. The authors argued that DTW is useful for feature alignment in space and time. The idea of this research is interesting, however I am not really convinced by how the authors explained the details of the proposed method and how the proposed method contributes in the field of time series analysis based on machine learning.

Several comments regarding this paper:

1. What is the main contribution of this paper? The authors should explain contribution of the manuscript in the relevant field. In fact, Dynamic Time Warping (DTW) and K-Means clustering have been used widely in time series analysis across different research fields. In terms of time series analysis, DTW and K-Means clustering are not new methods.
2. Dynamic Time Warping is good for similarity measure between two time series data. However, this algorithm has a time and space complexity of $O(S_{n1}, S_{n2})$ where S_{n1} and S_{n2} are the lengths of the respective time series sequences between which DTW distance is to be calculated (see line 84-89). On the other hand, there are several other implementations of DTW that are faster in implementation, such as PrunedDTW[1], SparseDTW[2], FastDTW[3], and MultiscaleDTW[4]. Why did the authors choose original DTW to be used in this research instead of considering other faster version of DTW?
3. What is the size of the local cost Matrix M?
4. In addition to the chosen DTW method, the authors also choose K-Means clustering by justifying that K-Means is simple and efficient (line 159). However, K-Means clustering is well-known as a "lazy classifier" and it takes more computational time in classification process. Simplicity in implementation does not mean that this algorithm is appropriate to solve the problem. More justification is needed and please compare the proposed classification method with other classification method.
5. In line 143-144, the authors wrote: "parameters such as boundary condition, continuity condition, monotonic condition and warping window condition were left the same during the analysis." More detailed explanation on these parameters are needed to make this research reproducible.

---

[1] http://sites.labic.icmc.usp.br/prunedDTW/
[2] https://arxiv.org/abs/1201.2969
[3] https://cs.fit.edu/~pkc/papers/tdm04.pdf
[4] https://www.researchgate.net/publication/334413562_Iterative_Multiscale_Dynamic_Time_Warping_IMs-DTW_A_tool_for_rainfall_time_series_comparison

6. In this research, the authors used a dataset of 72 women's international basketball games ranging from the 2014 FIB world Championship to the 2016 Rio Olympic Games. Was there any specific justification in choosing this dataset? In fact, there are other datasets available in the internet, such as a dataset provided by Rajiv Shah[5].
7. More detailed discussions are needed, for instance:
    a. How does this research contribute in the field of machine learning, especially compared with previous works of time series analysis in sports data? Comparison with previous works is needed to see that the proposed method is better than current state-of-the-arts in the field.
    b. What are limitations of this research? What is the trade-off of implementing DTW and K-Means clustering?

*** EOF ***

[5] http://projects.rajivshah.com/blog/2016/04/02/sportvu_analysis/
